# Molecular Dynamics and Electron Density Topology Reveal Ligand-Specific Interaction Patterns at the Dopamine D_2_ Receptor

**DOI:** 10.3390/ijms27156572

**Published:** 2026-07-23

**Authors:** Gerardo Padilla-Bernal, Leonardo David Herrera-Zúñiga, Rubicelia Vargas

**Affiliations:** 1 Departamento de Química, Universidad Autónoma Metropolitana–Iztapalapa, Av. Ferrocarril San Rafael Atlixco 186, Col. Leyes de Reforma 1A Sección, Iztapalapa, Ciudad de México 09310, Mexico; gerapadillabernal@gmail.com; 2 HS Estudios Farmacoeconómicos, Torres Quintero 237, San Miguel, Iztapalapa, Ciudad de México 09360, Mexico; leonardo.herrera@hsestudiosfarmacoeconomicos.com.mx

**Keywords:** protein–ligand interactions, QTAIM, GPCR, molecular modeling, computational chemistry, molecular dynamics simulations

## Abstract

The dopamine D_2_ receptor (D2R) is one of the principal therapeutic targets for the treatment of schizophrenia and other neuropsychiatric disorders. Understanding how ligands with different pharmacological profiles interact with D2R is essential for the rational design of safer and more effective antipsychotic drugs. In this work, Molecular Dynamics (MD) simulations combined with Quantum Theory of Atoms in Molecules (QTAIM) analysis were employed to investigate the electronic nature of protein–ligand interactions in D2R embedded in a neuronal membrane environment. Representative agonists (dopamine and rotigotine) and antipsychotics from different generations (haloperidol, risperidone, and aripiprazole) were analyzed to identify interaction patterns associated with distinct pharmacological activities. The agonist-bound simulations revealed recurrent interactions involving the serine-rich region, whereas the antipsychotic-bound systems exhibited more persistent contacts within the central aromatic region of the binding pocket. These observations suggest ligand-associated interaction tendencies rather than universal determinants of agonism or antagonism. Furthermore, aripiprazole displayed a unique interaction profile characterized by enhanced coupling with the PIF connector, suggesting a distinct modulation of the TM6 toggle switch compared with other antipsychotics. The integration of MD and electron density topology revealed ligand-specific interaction networks associated with distinct pharmacological profiles at D2R. The interaction patterns identified in this study highlight characteristic interaction motifs associated with ligand-specific pharmacological profiles and provide mechanistic insights that may support the rational design of novel dopaminergic therapeutics.

## 1. Introduction

According to the World Health Organization, schizophrenia is a chronic disorder that affects around 1% of the global population [1]. To manage and alleviate its symptoms, various antipsychotic drugs have been developed, with the dopamine D_2_ receptor (D2R) being one of the primary pharmacological targets [2]. First-generation antipsychotics, such as haloperidol, were discovered around the same time as D2R identification and exhibit high selectivity for this receptor [3]. However, prolonged use of these drugs is associated with extrapyramidal symptoms (EPS), which manifest as movement disorders similar to those seen in Parkinson’s disease [4,5,6,7].

Second-generation antipsychotics, such as risperidone, have demonstrated a reduced incidence of EPS. Nevertheless, their use has been associated with an increased risk of metabolic side effects, including diabetes and hyperprolactinemia [4,5,6,7]. In this context, Jacques van Rossum [8] proposed that all antipsychotics act as antagonists of the D2R, an exception to this model is aripiprazole, which under certain physiological conditions functions as a partial agonist [9]. Due to this unique pharmacological profile, aripiprazole is often classified as a third-generation antipsychotic [8,9].

D2R belongs to the class of G-protein-coupled receptors (GPCRs), a large family of transmembrane receptors that regulate intracellular signaling cascades associated with key physiological processes. The pharmacological relevance of GPCRs is substantial, as approximately 30% of the currently available drugs target these receptors [10,11,12]. Structurally, GPCRs are characterized by a bundle of seven transmembrane α-helices (TM1–TM7), an intracellular helix (H8), three extracellular loops (ECL1–ECL3), and three intracellular loops (ICL1–ICL3). The N-terminus (N-ter) is oriented toward the extracellular space, while the C-terminus (C-ter) resides intracellularly.

The orthosteric binding pocket of GPCRs is typically located between the TM3, TM5, TM6, and TM7 helices. Given the high degree of structural conservation among GPCRs, the Ballesteros–Weinstein numbering system is commonly employed to standardize the identification and comparison of functionally important residues across different receptors [13].

The dopamine D_2_ receptor (D2R) is expressed primarily in cells of the central nervous system, where they modulate key functions such as locomotion, attention, sleep, memory, and learning, among others [14]. Due to their critical role in neuropsychiatric disorders, the activation mechanism of D2R has been extensively studied, leading to significant insights into its role within intracellular signaling pathways. However, as with other GPCRs, the precise molecular details of its activation process remain unclear.

To date, various studies have identified structural motifs associated with the general activation mechanism of GPCRs, commonly referred to as microswitches. These include the transmission motif (CWxP), which is involved in the outward displacement of TM6 during the transition from the inactive to the active state; the ionic motif (DRY), associated with the stabilization of the inactive conformation [15]; the tyrosine motif (NPxxY) [16]; and the PIF motif, a connector between the receptor’s two allosteric sites [17,18].

In addition, several conserved residues in D2R, often referred to as microdomains, have gained increasing attention due to their potential involvement in ligand recognition and binding [19]. The anionic domain centered on Asp114 (3.32 in the Ballesteros–Weinstein numbering scheme), where a salt bridge is formed between the basic amine group of the ligand and the acidic side chain of the receptor; the serine domain consisting of Ser193 (5.42), Ser194 (5.43), and Ser197 (5.46); which plays a role in the stabilization of dopamine-like agonists [20]; and the aromatic domain comprising Trp386 (6.48), Phe389 (6.51), Phe390 (6.52), and His393 (6.55), which is thought to contribute to the activation of the receptor via the toggle switch mechanism [20]. Recent findings have also highlighted the functional relevance of sodium ion (Na^+^) in GPCR activation. This ion interacts with a highly conserved region referred to as the Na^+^ pocket [21]. Many of these key motifs and microdomains are located within the orthosteric binding pocket.

The stabilization of the active and inactive conformations of GPCRs largely depends on the intermolecular interactions established between ligand molecules and the receptor’s binding pocket [22,23]. Therefore, the study of noncovalent interactions between these sites and therapeutically active drugs is particularly relevant for rational drug design. Understanding these interactions can help identify key residues involved in ligand recognition and activation, ultimately contributing to the development of safer and more effective antipsychotics targeting D2R.

From a theoretical chemistry perspective, Molecular Dynamics (MD) has become a widely employed computational tool due to its ability to simulate large-scale atomic systems over time. However, its scope is limited when it comes to accurately describing the electronic nature of noncovalent interactions that underpin protein structure and function. In this context, the Quantum Theory of Atoms in Molecules (QTAIM) [24,25] offers a valuable alternative. QTAIM provides a rigorous framework for classifying various types of chemical interactions, including covalent bonds, hydrogen bonds, and electrostatic interactions, through the analysis of the electron density topology and associated local energetic descriptors [26,27].

The aim of this study is to explore the relationship between the nature of intermolecular interactions in D2R–ligand complexes and the pharmacological activity of therapeutically relevant ligands, with the goal of generating insights for the rational design of antipsychotic drugs. Haloperidol, risperidone, and aripiprazole were selected as representative compounds of first-, second-, and third-generation antipsychotics, respectively. To contrast the interaction patterns of antipsychotics with those of agonists, the endogenous agonist dopamine and the antiparkinsonian agent rotigotine were included. Rotigotine has demonstrated efficacy in treating extrapyramidal symptoms at low doses [28] and was included as an additional agonist for comparative analysis.

To facilitate the interpretation of the computational results, Table 1 summarizes the experimentally reported binding affinities of the ligands analyzed in this study. The selected compounds represent distinct pharmacological profiles at the dopamine D2 receptor, including the endogenous agonist dopamine, the non-ergoline agonist rotigotine, the antagonists haloperidol and risperidone, and the partial agonist aripiprazole. To ensure consistency among the reported affinity values, all $K_{i}$ measurements were collected from studies employing [^3^H]spiperone as the radioligand.

As shown in Table 1, the ligand set encompasses agonist, antagonist, and partial agonist pharmacological profiles, providing a representative framework for exploring how distinct interaction networks emerge within the D2R binding pocket.

Our study combines Molecular Dynamics (MD) simulations with electron density topology analysis within the Quantum Theory of Atoms in Molecules (QTAIM) framework to obtain a comprehensive characterization of D2R–ligand interactions from both structural and electronic perspectives. Because the membrane environment plays an important role in GPCR conformational dynamics, a neuronal membrane model was constructed to simulate D2R under membrane-embedded conditions. This integrated approach enables the identification of interaction motifs associated with different pharmacological profiles and provides an electronic-level description of ligand recognition in D2R.

## 2. Results and Discussion

### 2.1. Docking Results

The structures ligand–D2R obtained from molecular docking showed a preferential orientation of the basic amine group toward TM6, with the exception of dopamine.

Haloperidol exhibited a binding pose that differed from the crystallographic structure of the same neuroreceptor (PDB ID: 6LUQ). In particular, the fluorine atom in the docking model was oriented outward, rather than forming interactions with the PIF motif as observed in the crystal structure [34]. For this reason, the crystallographic pose was selected as a reference for comparison with the docking-generated models.

Validation of the docking protocol against experimentally resolved D2R–ligand complexes showed that the method was able to reproduce the crystallographic binding orientations of risperidone within RMSD values of 2.1–5.7 Å. For haloperidol, RMSD values of 8.5–9.0 Å were obtained. Inspection of the corresponding poses revealed that these larger deviations did not arise from displacement of the ligand from the orthosteric binding pocket, but rather from an alternative orientation in which the fluorinated moiety was directed away from the PIF connector relative to the crystallographic structure. Consequently, the overall location of the ligand within the binding site remained similar, while the different orientation produced substantially larger RMSD values. Therefore, the crystallographic haloperidol pose was retained as an additional reference system and evaluated in parallel with the docking-generated conformations. This comparison allowed us to distinguish ligand orientations consistent with the available structural evidence from alternative binding modes within the same orthosteric pocket and to evaluate whether the alternative orientation identified by docking affected the interaction patterns observed during the subsequent analyses.

### 2.2. Molecular Dynamics Analysis

Once the docking procedure and MD simulation were completed and the binding poses were obtained, clustering analysis was performed to identify the predominant conformations. Results indicated that all antipsychotic-bound systems were distributed across three population clusters, each comprising approximately 30% of the total systems. In contrast, dopamine and rotigotine each exhibited a single dominant cluster. This observation aligns with the well-established notion that GPCRs do not adopt a single, fixed active or inactive conformation [22].

Based on the population distribution, three groups of antipsychotic-bound antagonist systems were identified and analyzed in this study. These were labeled as haloperidol-bound structures: HALO1, HALO2, HALO3, and HALO_CRYS_; risperidone-bound structures: RISP1, RISP2, and RISP3; and aripiprazole-bound structures: ARI1, ARI2, and ARI3. In addition, the dopamine-bound structure was labeled DOPA, and the rotigotine-bound structure was labeled ROTI.

Post-simulation analysis revealed the orientation of the amine group in the ligands relative to residue Asp114 in most antipsychotic-bound complexes, highlighting the crucial role of salt bridge formation in aminergic receptors such as D2R. This interaction serves as a key anchoring mechanism that stabilizes the ligand [35].

**Figure 1:**
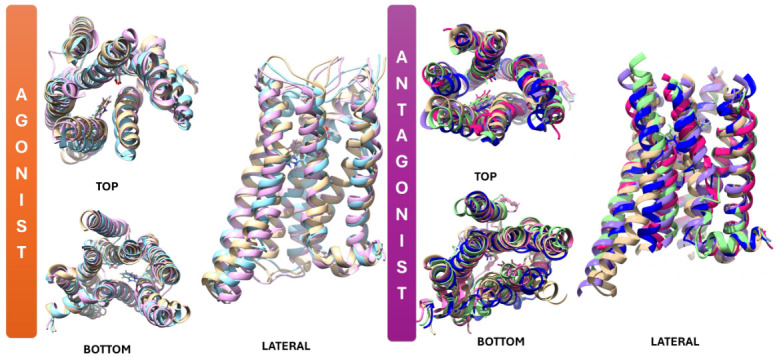
Superposition of D2R structures divided by agonist-bound and antagonist-bound forms. Principal changes are observed in TM6. Agonist-bound systems: APO–D2R (beige), D2R in complex with dopamine (cyan) and rotigotine (violet). Antagonist-bound systems: APO–D2R (beige), D2R in complex with haloperidol HALO1 (pink), haloperidol crystal pose HALO_CRYS_ (violet), risperidone RISP1 (green), and aripiprazole ARI1 (blue).

Figure 1 and Figure S2 of the Supporting Information (SI) display the superposition of the clustered structures. In agonist-bound systems, only transmembrane helix 6 (TM6) displays a marked outward displacement relative to the APO conformation, consistent with activation-associated rearrangements. In contrast, antagonist-bound systems show conformational changes primarily in TM5, TM6, and TM7. Previous studies have emphasized the pivotal role of TM6 in receptor activation, often described as a “toggle switch” due to its functional resemblance to such mechanisms [18,22,36]. Among the antipsychotic complexes, the aripiprazole-bound structures ARI1 and ARI3, along with the risperidone-bound structure RISP2, display subtle conformational differences. In these cases, TM6 appears to adopt an intermediate position between the APO structure and the other antipsychotic-bound conformations.

**Figure 2:**
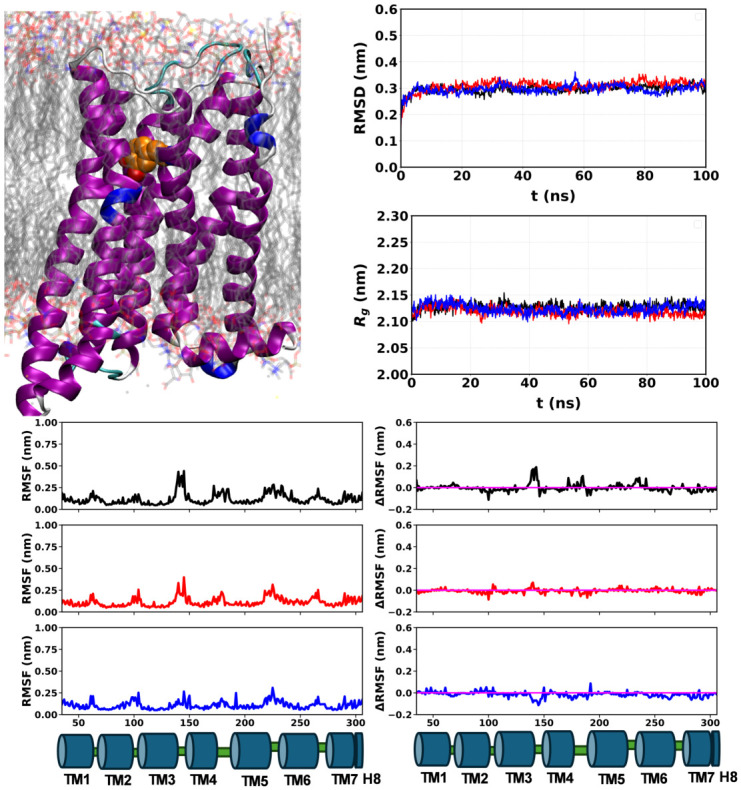
Summary of MD analysis for dopamine in a complex with D2R. From left to right, top to bottom: RMSD plot, radius of gyration, and RMSF plot. A schematic representation of the TM domains is included for reference. Black, red and blue are the MD indepent runs; the pink line referes to APO RMSF.

To explore conformational changes related to drug activity, C_α_–C_α_ distance maps, RMSD, RMSF, and radius of gyration analyses were conducted. Results are shown in Figure 2 for the dopamine-bound system and in Figures S3–S8 for the remaining systems.

The C_α_–C_α_ distance plots reveal marked differences between agonist- and antagonist-bound systems. In antagonist-bound complexes, a reduction in the distance between TM2 and TM6 is observed at the binding pocket level. Additionally, a closer proximity between the upper region of TM2 and ECL2 in the extracellular domain suggests a contraction of the binding site. On the intracellular side, TM3 and TM6, where the DRY microswitch is located, also shift closer together. In contrast, antagonist systems exhibit an increased distance between TM3 and TM7 on the intracellular face, indicating a distinct conformational pattern compared to agonist-bound systems.

RMSD analysis across all systems confirms that conformational stability is reached after 20 ns. Most antagonist-bound systems exhibit minimal fluctuations, whereas agonist-bound systems maintain relatively stable but slightly higher RMSD values throughout the simulation. Notably, one of the haloperidol-bound simulations displayed a distinct RMSD profile compared to the other replicates.

The radius of gyration measures the overall distribution of atoms around the center of mass and provides information about the compactness of the protein during the MD simulation. All systems exhibited an average value around 2.15 nm. Dopamine-bound structures showed a stable profile, comparable to the APO structure. In contrast, antipsychotic-bound systems presented alternating compression-expansion patterns, differing from the crystal pose structure, which remained stable throughout the simulation.

RMSF analysis quantifies the flexibility of individual residues, with loop regions showing the highest mobility, as typically observed in this type of receptor. Additionally, ΔRMSF values, calculated by comparing ligand-bound systems to the APO structure, highlight conformational differences between active and inactive states. Both dopamine- and rotigotine-bound D2R structures remained largely stable, though localized fluctuations were observed in intracellular loops ICL2 and ICL3, likely in response to ligand binding. Among antagonist-bound systems, aripiprazole complexes exhibited the lowest overall fluctuations. Conformational analysis indicates that the dopamine-bound system closely resembles the APO structure, whereas aripiprazole-bound systems tend to adopt an intermediate conformation between those of antagonist- and agonist-bound states.

The replica-resolved PLIP profiles indicated recurrent and replica-dependent interactions in the ligand-binding networks (Figures S9–S14). In the antipsychotic-bound systems a conserved central anchoring pattern was found around the anionic residue Asp114, where a salt-bridge interaction was discovered in all three replicates, albeit its relative frequency and the concomitant interactions differed among simulations. Also, the residues in the central aromatic area of the pocket, especially the ones surrounding Phe389 and Phe390, frequently appeared in the interaction profiles of haloperidol, risperidone and aripiprazole.

In the dopamine- and rotigotine-bound systems, interactions involving the serine-rich region, particularly residues Ser193, Ser194 and Ser197, were consistently identified across the three replicas. Rotigotine also engaged this region, although with a more variable interaction profile. Similar interactions were detected in some antipsychotic-bound simulations, but they were less consistently maintained across replicas. Thus, interactions involving the serine microdomain occurred in both agonist- and antipsychotic-bound systems; however, their recurrent involvement was more pronounced in the agonists examined in this study.

Interactions involving peripheral residues and the TM7 region exhibited greater variability, both in frequency and interaction type, across the different replicas. This variability suggests that individual residue contacts are unlikely to represent universal signatures of agonist or antagonist pharmacology. Instead, the results support the existence of ligand-associated interaction patterns involving broader receptor microdomains.

The replica-resolved PLIP analysis provides information about the recurrence and variability of protein–ligand contacts throughout the MD trajectories. The subsequent QTAIM analysis focuses on representative conformations extracted from these trajectories and provides an electronic-structure perspective of the interactions identified by PLIP. Consequently, the two approaches should be regarded as complementary rather than equivalent descriptions of ligand–receptor recognition.

### 2.3. Topological Analysis of Interactions Associated with Pharmacological Profiles

**Figure 3:**
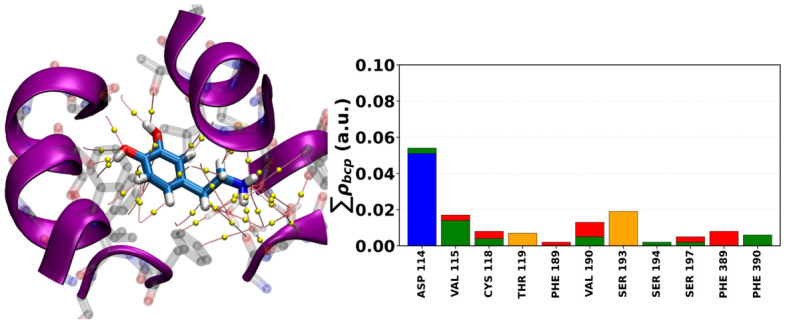
QTAIM analysis of dopamine in complex with D2R. On the left, the molecular graph is presented with bond critical points (yellow) and bond paths (pink) that indicate the presence of interactions. On the right, the intermolecular $\rho_{bcp}$ per residue is shown, highlighting the nature of the interactions: charged-assisted hydrogen bond (blue), conventional hydrogen bond (orange), non-conventional hydrogen bond (green) and dihydrogen bond (red).

To analyze the noncovalent interactions in antipsychotic-bound systems, binding pocket models of D2R were constructed based on representative structures obtained from clustering. A molecular interaction graph and corresponding bar plots depicting key residue interactions are shown in Figure 3 and Figures S15–S19 for the remaining systems.

The majority of predicted interactions correspond to non-conventional hydrogen bonds (CH⋯X) and dihydrogen interactions (CH⋯HC). Among these, CH⋯π interactions involving aromatic residues exhibited the highest interaction values. Although the relationship between aromaticity and QTAIM descriptors remains under investigation [37,38,39], initial findings suggest that such interactions play a significant role in ligand stabilization.

One of the key advantages of QTAIM lies in its ability to determine the existence of interactions between atoms through the molecular graph, which also allows for the identification of specific residue–ligand interactions. This approach provides results that are consistent with those obtained from other methods for predicting protein–ligand interactions [40,41]. The main interactions identified across all clusters are summarized in Figure S20.

The Asp114 residue, which most frequently interacts with the ligands, typically exhibits the highest $\rho_{bcp}$ values among all ligand–residue interactions; this trend is consistent with observations reported in Ref. [42]. As shown in Table 2, this salt bridge is associated with positive values of $\nabla^{2}\rho_{bcp}$ in all systems, indicating a closed-shell interaction according to QTAIM criteria. Nevertheless, several complexes display $|V_{bcp}|/G_{bcp}$ values close to or slightly above unity together with negative $H_{bcp}$ and $H_{bcp}/\rho_{bcp}$ values, suggesting a non-negligible degree of electron sharing. Interestingly, the positive bond-degree values are observed for systems in which the protonated amine interacts with both oxygen atoms of the Asp114 carboxylate group, suggesting that bifurcation of the salt bridge may reduce the degree of electron sharing associated with each individual contact. Therefore, this interaction is more appropriately described as a charge-assisted hydrogen bond with varying degrees of covalent contribution rather than as a purely electrostatic or fully covalent interaction.

Among all ligands, dopamine exhibited the fewest interactions, both in terms of the number of atomic contacts (22) and interacting residues (11), likely due to its smaller molecular size compared to the antipsychotics. Nonetheless, the strength of its interaction with Asp114 is comparable to that observed with antipsychotic ligands (Figure 3).

Additionally, the distribution of electron density at bond critical points ($\rho_{bcp}$) in specific interaction regions provides important details regarding the ligand–receptor interactions. QTAIM critical point analysis can complement the geometrical description provided by other methodologies based on geometric criteria; previous work by our group has shown that QTAIM complements the interaction profile compared to those derived from PLIP [43].

Figure S21 illustrates the electron density associated with protein–ligand interactions, including those mediated by water molecules. Among the systems analyzed, haloperidol, considered a selective antipsychotic, showed the fewest water-mediated interactions, while risperidone exhibited the highest number, marking a clear difference between the two within a 5.0 Å cutoff.

**Figure 4:**
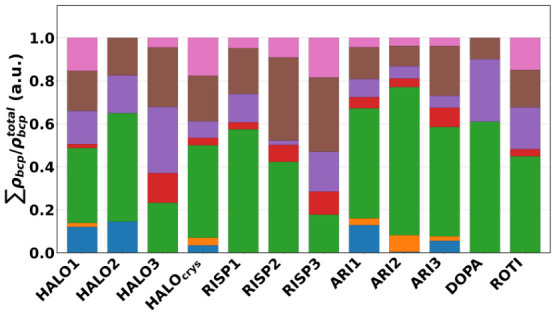
Analysis of fractional $\sum \rho_{bcp}$ distribution of principal D2R domains within a 5.0 Å cutoff from the ligand: TM2 (blue), ECL1 (orange), TM3 (green), ECL2 (red), TM5 (violet), TM6 (brown), and TM7 (pink).

Figure 4 presents the electron density grouped by structural domains. In all systems where a salt bridge with Asp114 was present, transmembrane helix 3 (TM3) displayed the highest number of interactions, followed by TM6. Only the antipsychotic-bound systems showed interactions involving the PIF connector.

Interactions involving the TM2 and ECL1 domains were observed exclusively in the haloperidol-bound systems (both poses) and in aripiprazole. Interestingly, dopamine stood out among all ligands by interacting with three distinct domains: TM3, TM5, and TM6, demonstrating a unique binding profile compared to the other compounds.

**Figure 5:**
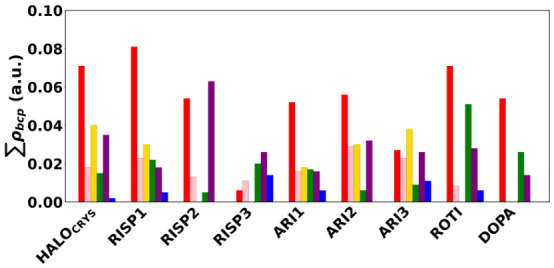
$\sum \rho_{bcp}$ of some residues related to the activation mechanism of D2R interacting with antipsychotics within a 5.0 Å cutoff from the ligand: Asp114 (red), Trp386 (pink), PIF connector (yellow), serine domain (green), Phe389–Phe390 from aromatic domain (purple), and Tyr416 (blue). For haloperidol, only the crystal pose is presented. The dopamine molecule is included for comparison.

The total sum of $\rho_{bcp}$ values across all intermolecular interactions was used as a comparative descriptor of the ligand–receptor interaction network. In previous QTAIM studies, summed electron density values at bond critical points have been employed to evaluate the collective contribution of multiple intermolecular contacts within supramolecular assemblies [42]. Consistent with this interpretation, $\sum \rho_{bcp}$ is considered here as a descriptor of the extent and organization of the interaction network rather than as a direct measure of binding affinity or interaction energy. Figure 5 shows the summed electron density at bond critical points ($\rho_{bcp}$) for several key microdomains relevant to aminergic GPCR function. These include Asp114, the serine microdomain, and the aromatic microdomain, which comprises Trp386 from the CWxP motif (analyzed separately in this study for clarity). Tyr416 (7.43) [44] and the PIF connector [19] were also considered.

Asp114 consistently emerged as a critical interaction site across nearly all systems, supporting its role as the primary anchoring point for aminergic ligands in D2R. However, interactions involving other microdomains displayed a stronger dependence on ligand pharmacology. In the replica-resolved PLIP profiles, repeated interactions between dopamine or rotigotine and the serine microdomain were consistently observed. Contacts involving this region were also detected in some antipsychotic-bound simulations, although they were less consistently conserved across replicas. Together, these observations suggest a ligand-associated tendency for more persistent participation of the serine microdomain in the agonist-bound systems analyzed in this study, rather than an exclusive marker of agonist activity. Although Zell et al. [19] proposed that interactions with the serine microdomain are not decisive for ligand mode of action, the present results indicate that this region may be associated with interaction patterns characteristic of agonist-bound systems.

Conversely, residues within the central aromatic region of the binding pocket, including Trp386 were recurrently involved in the interaction patterns observed for the antipsychotic-bound systems. Interactions involving Tyr416 were less consistently conserved across replicas. Together, these observations suggest ligand-dependent differences in the participation of TM6 and TM7, while indicating that individual residue interactions are unlikely to represent universal signatures of agonism or antagonism. Among the antipsychotics investigated, aripiprazole exhibited the strongest interaction with the PIF connector, whereas risperidone showed little involvement with this region. This finding is consistent with the distinct pharmacological profile of aripiprazole and suggests that interactions with the PIF connector may contribute to the modulation of receptor conformational dynamics.

**Figure 6:**
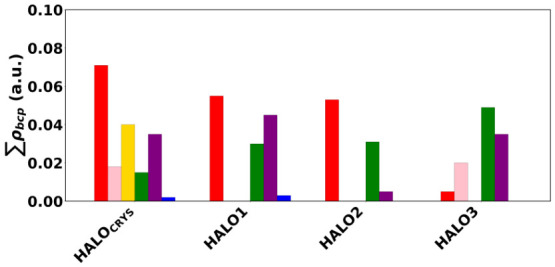
$\sum \rho_{bcp}$ of some residues related to activation mechanism of D2R interacting with haloperidol within a 5.0 Å cutoff from the ligand: Asp114 (red), Trp386 (pink), PIF connector (yellow), serine domain (green), Phe389–Phe390 from aromatic domain (purple), and Tyr416 (blue).

Ligand orientation also influences the interaction profile. Figure 6 compares two haloperidol binding poses, corresponding to the crystallographic and docking-derived conformations. Notable differences are observed between the two, including the strength of the salt bridge with Asp114, the extent of interaction with the PIF connector, and the electron density distribution in the serine microdomain. These differences are particularly evident when the chlorine atom is oriented outward, toward the extracellular space. These results illustrate how subtle changes in ligand orientation can alter the interaction network established within the binding pocket.

Overall, the replica-resolved PLIP profiles and representative QTAIM models indicate that Asp114 acts as a recurrent anchoring site, while the serine microdomain, Trp386, Tyr416, and the PIF connector participate differently depending on the ligand and the sampled conformation. These patterns are interpreted as ligand-associated interaction tendencies within the systems investigated. In particular, dopamine showed the strongest preference for the serine microdomain, in agreement with previous studies involving dopaminergic receptors [45]. Additional agonist ligands should be examined to further assess the role of this region in ligand recognition and selectivity.

The interaction patterns identified through QTAIM analysis are consistent with the known pharmacological profiles of the investigated ligands. Dopamine and rotigotine, which exhibit agonist activity, showed a greater participation of the serine microdomain, whereas antagonist ligands displayed enhanced interactions with residues associated with inactive receptor conformations. Aripiprazole displayed an intermediate interaction pattern, consistent with its partial agonist properties. Although no direct quantitative relationship between the QTAIM descriptors and ligand affinity is proposed, these results suggest that distinct efficacy classes are associated with different interaction networks within the D2R binding pocket.

## 3. Materials and Methods

The first D2R–ligand complexes for the ensuing molecular dynamics (MD) simulations were generated by means of molecular docking. The receptor models were built with experimentally determined D2R structures from the RCSB protein data bank. For the agonist-bound systems, the structure of the active-state D2R in complex with bromocriptine (PDB ID: 6VMS) was utilized as the structural template while for the antipsychotic-bound systems, the structure of the inactive-state D2R in combination with risperidone (PDB ID: 6CM4) was used as the structural template. No full-length D2R homology model was built. Since the 6CM4 structure did not contain the intracellular loop 3 (ICL3), just the missing loop segment was modeled using the loop-modeling protocol implemented in Modeller 10.6 [46], using the 6VMS structure as a structural reference for linking the TM5 and TM6 transmembrane helices. Titratable D2R residues were protonated with PROPKA at pH 7.4 [47,48].

The APO–D2R model was generated as a single reference system in a ligand-free state from the experimentally determined active-state D2R–bromocriptine structure (PDB ID: 6VMS). The co-crystallized ligand was removed while preserving the receptor conformation. No de novo modeling or full-length homology modeling was performed. The ligand-free receptor was prepared following the same methodology as the ligand-bound systems, including PROPKA protonation-state assignment at pH 7.4, membrane embedding, solvation, ion placement, equilibration, and subsequent MD simulation.

**Figure 7:**
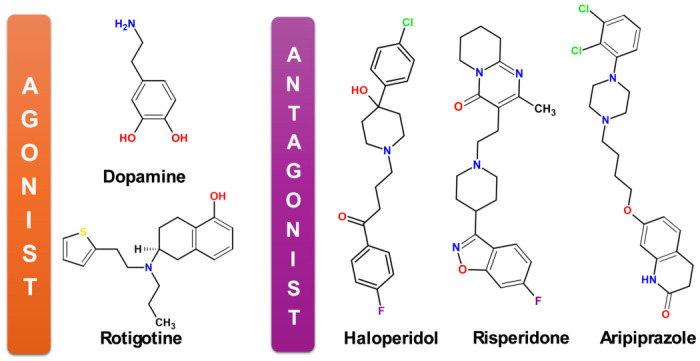
Ligands used in this work: Haloperidol, risperidone, and aripiprazole are antipsychotic drugs used as antagonists, dopamine (endogenous agonist) and rotigotine (antiparkinsonian agonist) were included as representative agonists. These molecules are related to schizophrenia treatment.

To investigate how different generations of antipsychotics interact with the D2R, the following representative molecules were selected: haloperidol (first-generation), risperidone (second-generation), and aripiprazole (third-generation). For comparison purposes, dopamine and the D2R agonist rotigotine (Figure 7) were included as well. All ligands were geometry optimized before docking at the PBE0-D3/6-311G** level of theory [49,50,51,52,53,54] and the protonated state of the basic amine group was examined in each case, based on the p$K_{a}$ reported in Table 1. The PBE0-D3/6-311G** level of theory was selected because it provides a balanced description of electron density distributions and noncovalent interactions while maintaining a computational cost compatible with the size of the receptor–ligand clusters considered in this study. This methodology has previously been employed in topological analyses of intermolecular interactions and has been shown to provide reliable electron density descriptors for QTAIM investigations [55,56].

The orthosteric binding pocket was defined based on the position of the co-crystallized ligand in the experimental D2R structures. Each ligand was subsequently docked independently into the corresponding prepared receptor model using AutoDock4 (v.1.5.7), AutoDock Vina (v.1.2.7), and Vinardo at AutoDock Vina [57,58,59]. This procedure was applied to all compounds, including risperidone and haloperidol, which were redocked rather than directly transferred from their crystallographic complexes.

For each ligand–receptor system, 500 docking runs were performed with each docking engine. The resulting poses were subsequently rescored using Vina to allow comparison within a common scoring framework. In total, 1500 docking calculations were carried out for each ligand–receptor system.

Docking poses were clustered using a 2 Å RMSD cutoff. The most populated cluster was selected as the representative binding mode for each ligand, which corresponds to the most favorable scoring energy and was subsequently employed as a starting D2R–ligand complex in the MD simulations. For haloperidol, the crystallographic D2R–haloperidol complex was retained exclusively as an external reference to assess the ability of the docking protocol to reproduce the experimentally determined binding orientation. Thus, the crystallographic binding mode of haloperidol was not used to replace the docking procedure or to impose a predefined binding mode, but rather was preserved as an external reference for evaluating the agreement between the docking-derived poses and the experimental structure. Predicted poses that were inconsistent with the experimental data were interpreted with caution and were further evaluated based on their stability during the MD simulations and their subsequent QTAIM interaction patterns.

Docking validation was carried out for ligand–receptor systems for which experimentally resolved binding modes are available in the Protein Data Bank. Co-crystallized ligands were removed from the receptor structures and independently redocked into the corresponding orthosteric binding pockets using the same protocol applied to all ligands analyzed in this study. The predicted poses were then compared with the crystallographic orientations after structural alignment of the receptor backbone. Validation was assessed through the heavy-atom RMSD between the redocked and experimental ligand poses (Figure S1).

Risperidone and haloperidol were selected for validation because experimentally determined D2R-bound structures are available for both ligands. The redocked risperidone pose showed a heavy-atom RMSD of 2.2 Å relative to the crystallographic pose in PDB ID 6CM4, whereas the redocked haloperidol pose showed a heavy-atom RMSD of 9.0 Å relative to the corresponding experimental structure, due to the different orientation. The docking protocol reproduced the crystallographic orientation of risperidone, whereas the haloperidol redocking yielded an alternative orientation within the orthosteric binding pocket. Consequently, the docking results are interpreted as providing reasonable starting conformations for the MD simulations rather than a general validation of reproducing crystallographic ligand orientations.

No experimentally resolved D2R–aripiprazole structure is currently available in the Protein Data Bank. Therefore, a direct crystallographic validation analogous to that performed for risperidone and haloperidol could not be carried out. The aripiprazole binding mode was consequently evaluated through docking consistency, molecular dynamics stability, and interaction pattern analysis.

To mimic the environment of the D2R receptor, an atomistic neuronal membrane was modeled using the Membrane Builder tool available on the CHARMM-GUI server. The receptor was embedded in a rectangular water box, and a 0.15 M concentration of NaCl was added to maintain physiological ionic strength.

Ligand parameters were created with the CHARMM General Force Field (CGenFF) [60] through the ParamChem/CGenFF procedure, which is compatible with the CHARMM36m force field utilized for the receptor and membrane components. CGenFF was chosen because it was especially created to expand the CHARMM additive all-atom force field to organic and drug-like compounds in biological settings. The generated CGenFF topology and parameter files were inspected, including the charge and bonded-parameter penalty scores reported by ParamChem. All penalty scores were below 50, although moderate values were observed for selected charge and bonded terms, particularly for risperidone. Since none of the scores reached the range typically associated with poor parameter analogy requiring extensive validation or reoptimization, no additional quantum-mechanical reparameterization was deemed necessary. The complete ParamChem/CGenFF penalty scores for all ligands are provided in Table S1 of the Supplementary Information.

The ligands haloperidol, risperidone, aripiprazole, dopamine, and rotigotine were modeled in their protonated forms with a net charge of +1, consistent with the expected protonation state of their basic amine groups under physiological conditions. No further quantum-mechanical reparametrization was performed and the default CGenFF-assigned atom types, parameters, and partial charges were used. The resulting ligand topologies were evaluated for chemically plausible atom type and were then included in the CHARMM36m-based simulation systems. All ligand-containing complexes passed the energy minimization and equilibration with no obvious structural instabilities before the production MD.

The TIP3P [61] water model was used, with a water layer thickness of 40 Å on both sides of the membrane. The lipid composition was derived from the neuronal membrane model reported in Ref. [62]. During membrane construction, CHARMM-GUI introduced minor adjustments in the number of lipid molecules required for bilayer generation while preserving the target lipid distribution. The final lipid composition employed in the simulations is summarized in Table 3, together with the corresponding values reported in Ref. [62]. Each resulting system contained approximately 200,000 atoms. During system preparation, the OPM-based orientation option provided by CHARMM-GUI was employed to preserve this arrangement during lipid bilayer construction.

MD simulations were performed using GROMACS 2020 [63] with the CHARMM36m force field [64]. Three independent simulations of 100 ns were conducted per system under the following conditions: Initial atomic velocities were assigned according to a Maxwell–Boltzmann distribution at 311.6 K using randomized velocity seeds. The leapfrog Verlet algorithm was used to integrate Newton’s equations of motion with a time step of 2 fs, which allowed the SHAKE algorithm to maintain all bonds involving hydrogen atoms. Long-range electrostatic interactions were computed using the Particle Mesh Ewald method, with a cut-off radius of 10 Å for noncovalent interact ions and a non-bonded pair-list distance of 15 Å. The equilibration protocol followed the standard CHARMM-GUI Membrane Builder scheme, which employs a stepwise reduction of positional and dihedral restraints on protein backbone and side chains, lipids, water molecules, and ions [65]. The equilibration consisted of 50 ps of NVT equilibration (two steps of 25 ps each) followed by 325 ps of semiisotropic NPT equilibration distributed over four successive stages (25 ps, 100 ps, 100 ps, and 100 ps). Berendsen temperature coupling was used throughout all equilibration stages at 311.6 K, while pressure was maintained at 1 bar.

Production simulations were carried out at 311.6 K and 1.013 bar using the Nosé–Hoover thermostat and the Parrinello–Rahman barostat. The selected temperature reflects the physiological conditions of the human brain [66]. The simulation time (100 ns) was used solely to allow structural relaxation under biochemical conditions. This timescale has been used in similar studies [45].

After the MD simulations, conformational clustering was carried out using the GROMOS clustering method based on Ref. [67] to identify the dominant conformations with an RMSD cutoff of 2.0 Å. To ensure equilibration, only frames from 20 to 100 ns of each production trajectory were included in the analysis. The initial equilibration period was not considered.

Binding pocket models were generated from the final cluster structure by selecting all residues with at least one atom within 5.0 Å of the ligand. The resulting fragments were capped at the N- and C-termini via methylation to preserve peptide bond continuity. This methodology has been successfully applied in previous studies [35,55,56].

To examine whether the interactions detected in the typical cluster structures, were also observed in the independent MD simulations, protein–ligand interaction profiles were constructed individually for each of the three replicas by means of PLIP [68]. Interactions were classified according to the geometric criteria adopted in PLIP, including hydrogen bonds, hydrophobic contacts, π-stacking interactions, and salt bridges. The residue-level profiles obtained were compared descriptively between replicates to find reproducible interaction areas and contacts that were limited to particular simulations. This analysis was not intended to be an inferential statistical comparison, but rather an assessment of the repeatability and conformational variability of the interaction patterns. The replica-resolved profiles are shown in Figures S9–S14.

The Quantum Theory of Atoms in Molecules (QTAIM) provides a robust framework for visualizing and classifying noncovalent interactions through the analysis of bond critical points in the electron density ($\rho_{bcp}$), where *bcp* indicates bond critical point, using both topological and energetic criteria. According to QTAIM, an interaction is considered to exist if a *bcp* is present between two nuclear attractors (i.e., atomic nuclei). Additionally, within this theoretical framework, bond paths are defined as conceptual lines of maximum electron density that connect two nuclei through a critical point. Therefore, QTAIM provides complementary information on molecular interactions that goes beyond traditional geometric parameters.

The $\rho_{bcp}$ is used as a measure of the electronic concentration in the interaction region, with larger values generally indicating stronger interactions. The Laplacian of the electron density ($\nabla^{2}\rho_{bcp}$) reflects local electron density concentration or depletion and is frequently employed to distinguish shared-shell (negative values) from closed-shell (positive values) interactions. The local potential energy density ($V_{bcp}$) and kinetic energy density ($G_{bcp}$) provide complementary information on the energetic nature of the interaction. In particular, the $\left|V_{bcp}\right|/G_{bcp}$ ratio has been widely used to classify bonding interactions, with values smaller than 1 generally associated with noncovalent closed-shell interactions, whereas values exceeding 1 indicate increasing covalent character. The bond degree descriptor, defined as $BD=H_{bcp}/\rho_{bcp}$ where $H_{bcp}$ = $G_{bcp}+V_{bcp}$, provides additional information regarding the covalent or noncovalent character of the interaction [26,27]. A summarize of these QTAIM descriptors can be seen at Table S2.

In this study, QTAIM analysis of noncovalent interactions was carried out as follows: single-point energy calculations and wavefunction generation were performed for all binding pocket models using the PBE0-D3/6-311G** level of theory. The resulting wavefunctions were then used to compute electron density scalar fields with the Graphics Processing Units for Atoms and Molecules (GPUAM) project [69,70,71]. QTAIM topological analysis was subsequently performed using the same code to identify *bcp*s. Noncovalent interactions, primarily hydrogen bonds, were characterized based on both electron density and energetic criteria. The QTAIM descriptors reported throughout this work are expressed in atomic units (a.u.). Only bcps meeting the condition ρ(bcp)>0.001a.u. were considered.

**Figure 8:**
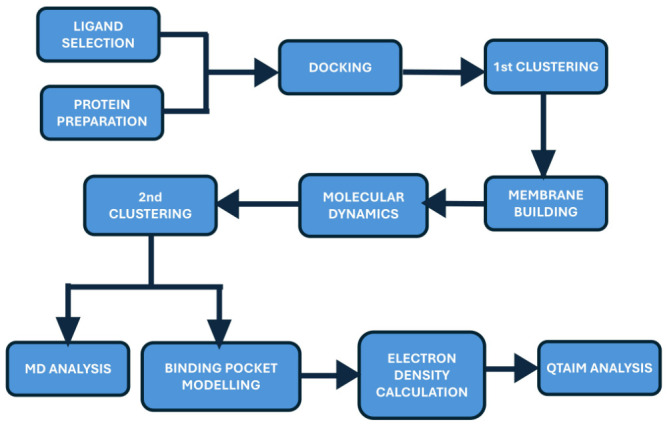
Schematic representation of the workflow employed in this study.

All the workflow is described in Figure 8.

## 4. Conclusions

This study investigated the relationship between ligand–receptor interactions and the pharmacological profiles of therapeutically relevant D2R ligands through the combined use of Molecular Dynamics (MD) simulations and Quantum Theory of Atoms in Molecules (QTAIM) analysis. MD simulations revealed recurring, yet replica-dependent, differences in interaction patterns and conformational behavior among the ligand-bound systems studied. Although variability was observed across independent simulations, conserved interaction regions were identified within each system, supporting the use of representative conformations for the subsequent QTAIM analysis.

QTAIM analysis identified Asp114 as the principal interaction site across nearly all systems, supporting its role as a conserved anchoring point for aminergic ligands. Beyond this common interaction, ligand-dependent differences were observed in other receptor microdomains. Dopamine and rotigotine exhibited recurrent interactions involving the serine microdomain, whereas the antipsychotic-bound systems more frequently involved residues within the central aromatic region of the binding pocket. However, the identity and persistence of individual residue interactions varied across replicas, particularly for TM7-associated contacts. These observations suggest ligand-associated interaction tendencies within the ligand set analyzed rather than definitive molecular determinants of agonist or antagonist pharmacology.

Among the antipsychotics studied, aripiprazole exhibited the most persistent interactions involving the PIF connector, distinguishing it from haloperidol and risperidone. Together with its intermediate conformational behavior, this finding is consistent with its reported partial agonist profile and highlights the possibility that different receptor microdomains contribute differently to ligand-specific interaction patterns. Nevertheless, the limited number of ligands analyzed in this study precludes broad generalizations regarding D2R activation or inhibition mechanisms.

Overall, the results indicate that, beyond the conserved anchoring role of Asp114, the serine microdomain, Trp386, and the PIF connector participate differently depending on the ligand and the sampled receptor conformation. These observations support the existence of ligand-associated interaction tendencies within the systems analyzed rather than universal determinants of agonist or antagonist pharmacology. The combination of MD simulations and QTAIM analysis provides complementary structural and electronic perspectives on ligand–receptor recognition, contributing to a more detailed understanding of D2R binding interactions and their relationship with ligand pharmacological profiles.

## Figures and Tables

**Table 1:** Pharmacological properties of the investigated ligands.

Ligand	Functional Efficacy	$K_{i}$ (nM) ^a^	p$K_{a}$ ^b^
Haloperidol	Antagonist	2.8 ± 0.4 [29]	8.05
Risperidone	Antagonist	4.5 ± 1.2 [29]	8.76
Aripiprazole	Partial agonist	0.52 ± 0.17 [30]	7.46
Rotigotine	Agonist	13.5 [31]	10.97
Dopamine	Agonist	260 ± 88.3 [32]	9.2

^a^ Binding data reported in this table were collected from studies that employed [^3^H]spiperone as the radioligand. ^b^ Data obtained from DrugBank [33].

**Table 2:** QTAIM bond critical point descriptors for Asp114–ligand interaction (N^+^H⋯O^−^).

Cluster System	$\rho_{\mathrm{bcp}}$	$\nabla^{2}\rho_{\mathrm{bcp}}$	$|V_{\mathrm{bcp}}|/G_{\mathrm{bcp}}$	$H_{\mathrm{bcp}}/\rho_{\mathrm{bcp}}$ ^a^
	(a.u.)	(a.u.)		
HALO1	0.039	0.126	1.03	−0.03
HALO2	0.037	0.133	1.01	0
HALO3	–	–	–	–
HALO_*crys*_	0.051	0.150	1.14	−0.12
RISP1	0.028	0.086	0.97	0.02
	0.020	0.073	0.89	0.09
RISP2	0.038	0.136	1.01	−0.01
RISP3	–	–	–	–
ARI1	0.043	0.134	1.08	−0.07
ARI2	0.047	0.130	1.13	−0.11
ARI3	–	–	–	–
DOPA	0.051	0.149	1.15	−0.13
ROTI	0.031	0.106	0.98	0.017
	0.013	0.037	0.85	0.085

^a^ $H_{bcp}$ normalized by $\rho_{bcp}$ to facilitate comparison. This quantity is also known as the bond degree [27].

**Table 3:** Lipid composition of the neuronal membrane model proposed in this study. Percentages are reported relative to the total lipid content of the membrane.

Lipid	Outer Surface			Inner Surface		
	Number	% ^a^	% ^b^	Number	% ^a^	% ^b^
Cho	66	13.52	13.03	121	24.80	25.67
POPC	26	5.33	5.19	50	10.25	10.22
SOPE	25	5.12	5.00	49	10.04	9.83
POPS	4	0.82	0.78	8	1.64	1.55
POPI	5	1.02	1.05	11	2.25	2.06
SSM	4	0.82	0.82	8	1.64	1.61
GalCer	86	17.62	18.00	–	–	–
Sulf	17	3.48	3.64	–	–	–
GM1a	4	0.82	1.50	–	–	–
GD1a	4	0.82		–	–	–

^a^ This work. ^b^ Reference [62].

## Data Availability

The original contributions presented in this study are included in the article/Supplementary Materials. Further inquiries can be directed to the corresponding author.

## Supplementary Materials

The following supporting information can be downloaded at: https://www.mdpi.com/article/10.3390/ijms27156572/s1.

## Abbreviations

The following abbreviations are used in this manuscript:

GPCR	G Protein-Coupled Receptor
D2R	Dopamine 2 Receptor
QTAIM	Quantum Theory of Atoms in Molecules
MD	Molecular Dynamics
