# Supplementary material for: Molecular Dynamics and Electron Density Topology Reveal Ligand-Specific Interaction Patterns at the Dopamine D2 Receptor
Source: Int J Mol Sci. 2026 Jul 23;27(15):6572. doi: 10.3390/ijms27156572 (PMC13465887; doi:10.3390/ijms27156572)
Supplement: Supplementary file 1 [file ijms-27-06572-s001.zip › ijms-4383018-supplementary.pdf]

Supporting Information: Molecular Dynamics and Electron Density Topology Reveal Ligand-Specific Interaction Patterns at the Dopamine D_2 Receptor

Gerardo Padilla-Bernal,[†] Leonardo David Herrera-Zúñiga,[‡] and Rubicelia Vargas[*,†]

†*Departamento de Química, División de Ciencias Básicas e Ingeniería, Universidad Autónoma Metropolitana - Iztapalapa, San Rafael Atlixco 186, Col. Vicentina, C.P. 09340 Iztapalapa, CDMX, México*

‡*Investigador y Coordinador, HS Estudios Farmacoeconómicos. Torres Quintero 237, San Miguel, Iztapalapa, 09360 Ciudad de México, CDMX, Mexico*

E-mail: rvargas@izt.uam.mx

In this section, the validation of the docking assays is presented alongside the parameterization and quality assessment of the studied ligands. Specifically, the maximum parameter and charge penalty scores derived from the CGenFF server are reported to verify the reliability of the generated force field parameters.

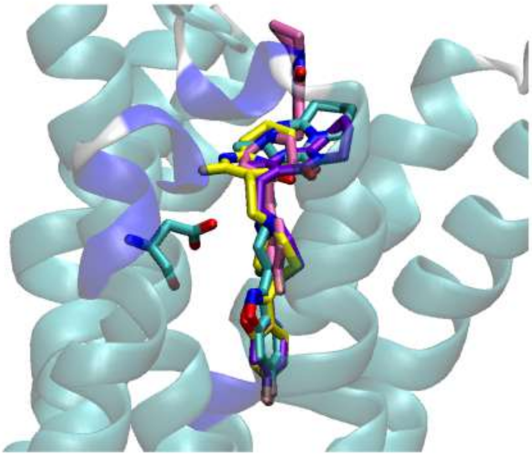

Risperidone	RMSD (Å)	$Energy_{scoring}$ (Kcal/mol)
Pose 1	2.17	-11.087
Pose 2	5.73	-9.951
Pose 3	2.93	-10.572

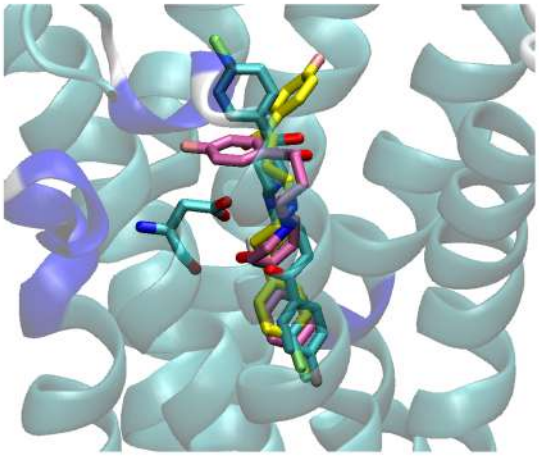

Haloperidol	RMSD Å	$Energy_{scoring}$ (Kcal/mol)
Pose 1	8.979	-9.679
Pose 2	8.491	-7.712

Figure S.1: Validation of the docking protocol using the available crystallographic structures. Comparison between the crystallographic ligand pose (carbon atoms shown in cyan) and the representative docked poses obtained from clustering analysis. The root-mean-square deviation (RMSD) relative to the crystallographic pose and the corresponding docking scores are reported.

Table S1: Penalty score for each ligand during parameterization obtained from CGenFF. Values below 10 indicate highly reliable parameters, scores between 10 and 50 recommend basic validation, and values above 50 mandate extensive optimization. All studied ligands fall within acceptable thresholds

**Ligand**	**Maximum parameter penalty**	**Maximum charge penalty**
Haloperidol	24.6	34.8
Risperidone	46.5	46.7
Aripiprazole	34.7	27.8
Dopamine	1.5	45.1
Rotigotine	2.1	6.7

In this section, protein structural superpositions and C_α–C_α distance matrices are presented to analyze conformational relationships within the systems. Additionally, RMSD, radius of gyration, and RMSF / Δ RMSF are shown for each system. The RMSF plots include a domain scheme to facilitate the interpretation of the results. For the D2R systems docked with antipsychotics, three clusters were identified.

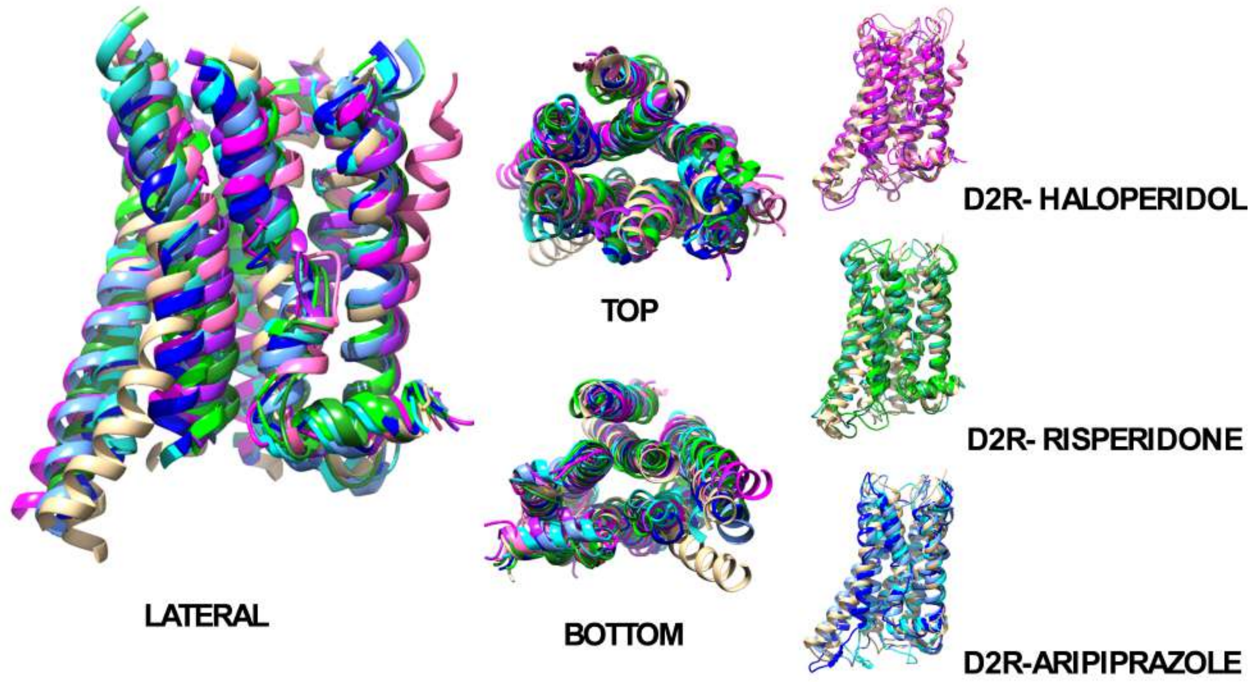

Figure S.2: Superposition of D2R structures from antagonist-bound forms. Significant changes are observed in TM6 for the following structures: APO-D2R (beige), D2R complexed with haloperidol (pink shades), D2R complexed with risperidone (green shades), and D2R complexed with aripiprazole (blue shades). Each drug is represented by three clusters, with color gradients indicating differences across them, from cluster one (darker) to cluster three (lighter).

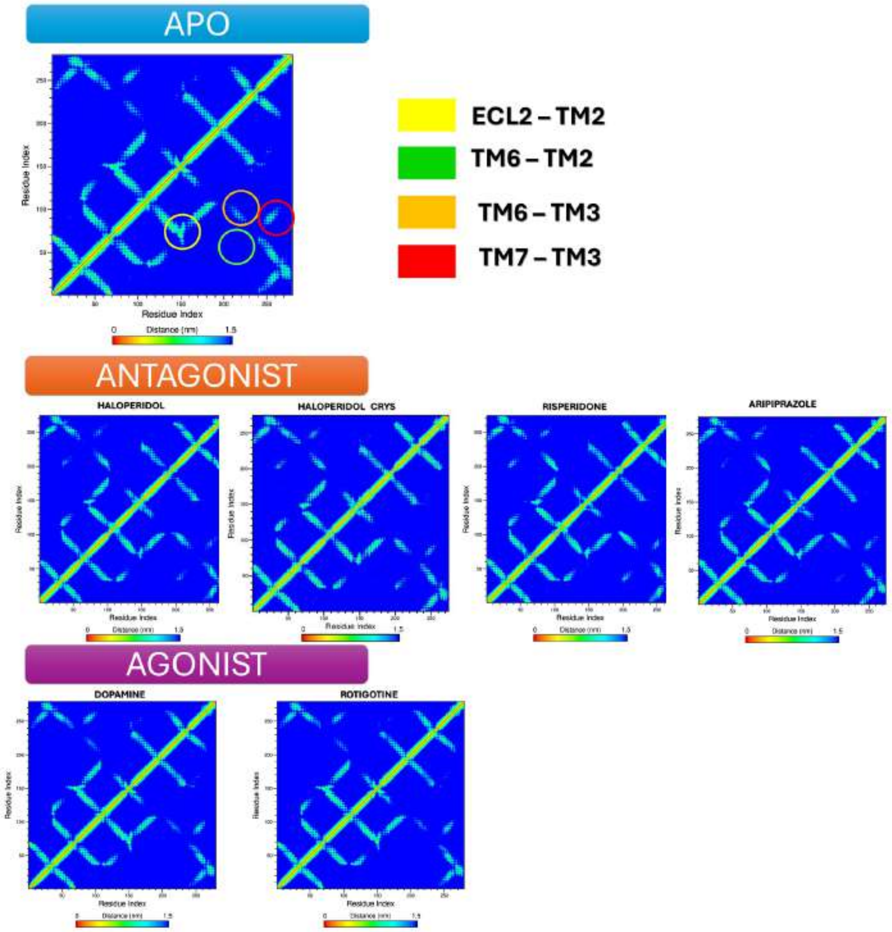

Residues	Domanin
1-30	TM1
31-36	ICL1
37-65	TM2
66-74	ECL1
75-106	TM3
107-111	ICL2
112-137	TM4
138-152	ECL2
153-189	TM5
190-198	ICL3
198-229	TM6
230-235	ECL3
236-270	TM7

Figure S.3: C_α-C_α distance matrix analysis. Cyrcles in colors in APO conformation point out principal changes between antagonist and agonist systems. Residue indexes shown in the table are added to locate D2R domains.

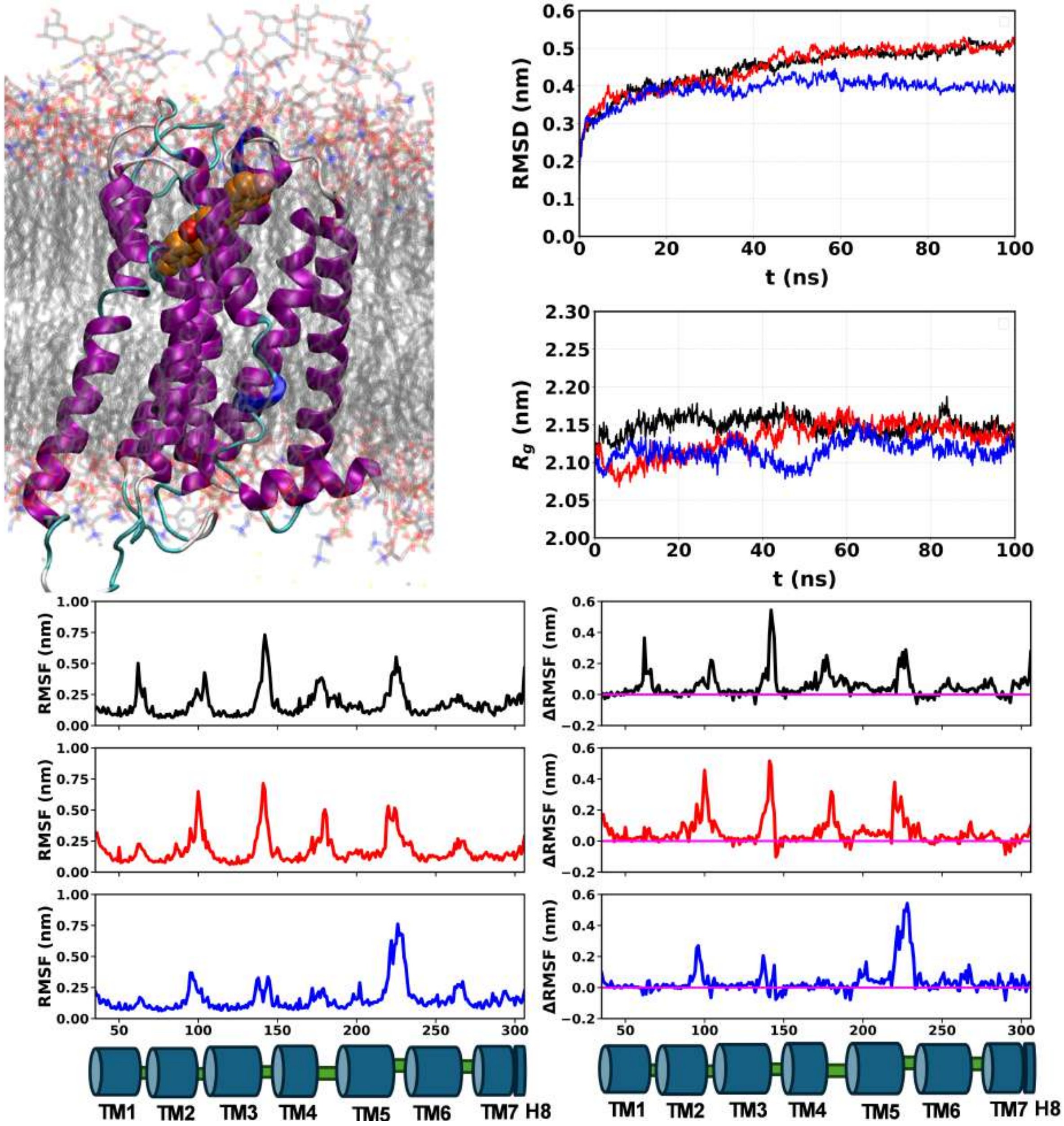

Figure S.4: MD analysis of haloperidol in complex with D2R. From left to right, top to bottom: RMSD plot, radius of gyration, and RMSF plot. A schematic representation of the TM domains is included for reference. Each MD run is represented by a different color: run 1 (black), run 2 (red), and run 3 (blue).

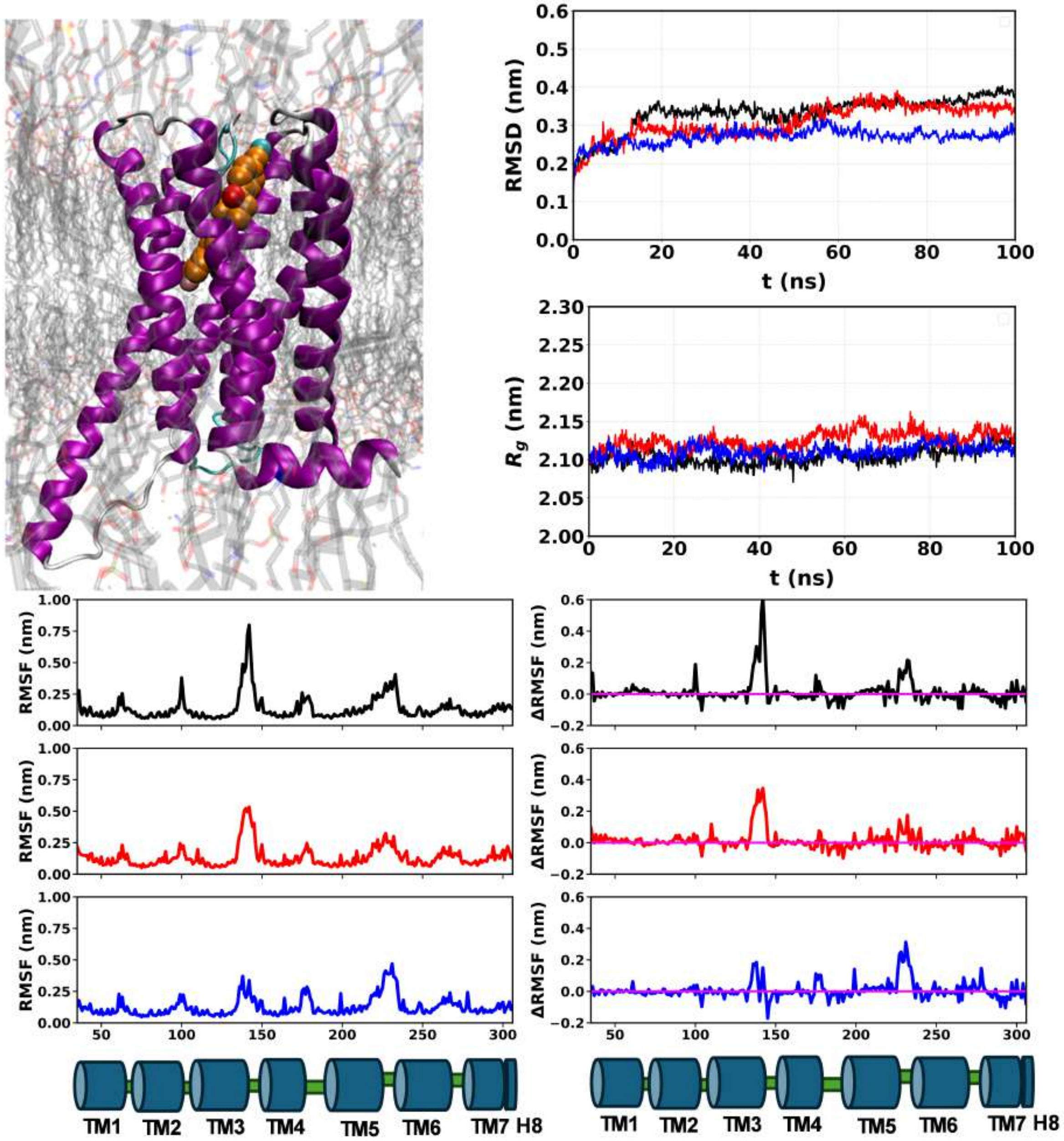

Figure S.5: MD analysis of haloperidol (crystal pose) in complex with D2R. From left to right, top to bottom: RMSD plot, radius of gyration, and RMSF plot. A schematic representation of the TM domains is included for reference. Each MD run is represented by a different color: run 1 (black), run 2 (red), and run 3 (blue).

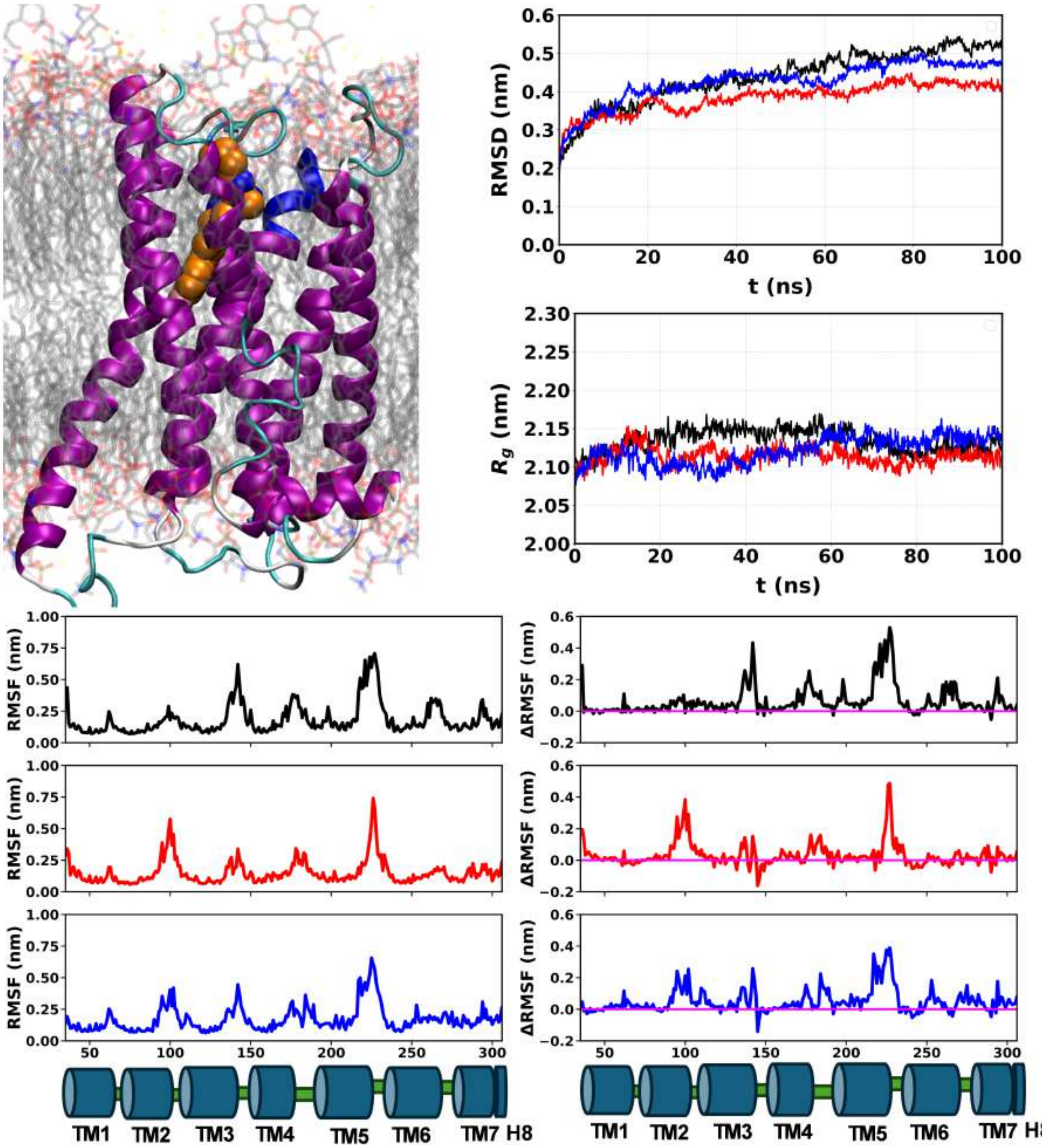

Figure S.6: MD analysis of risperidone in complex with D2R. From left to right, top to bottom: RMSD plot, radius of gyration, and RMSF plot. A schematic representation of the TM domains is included for reference. Each MD run is represented by a different color: run 1 (black), run 2 (red), and run 3 (blue).

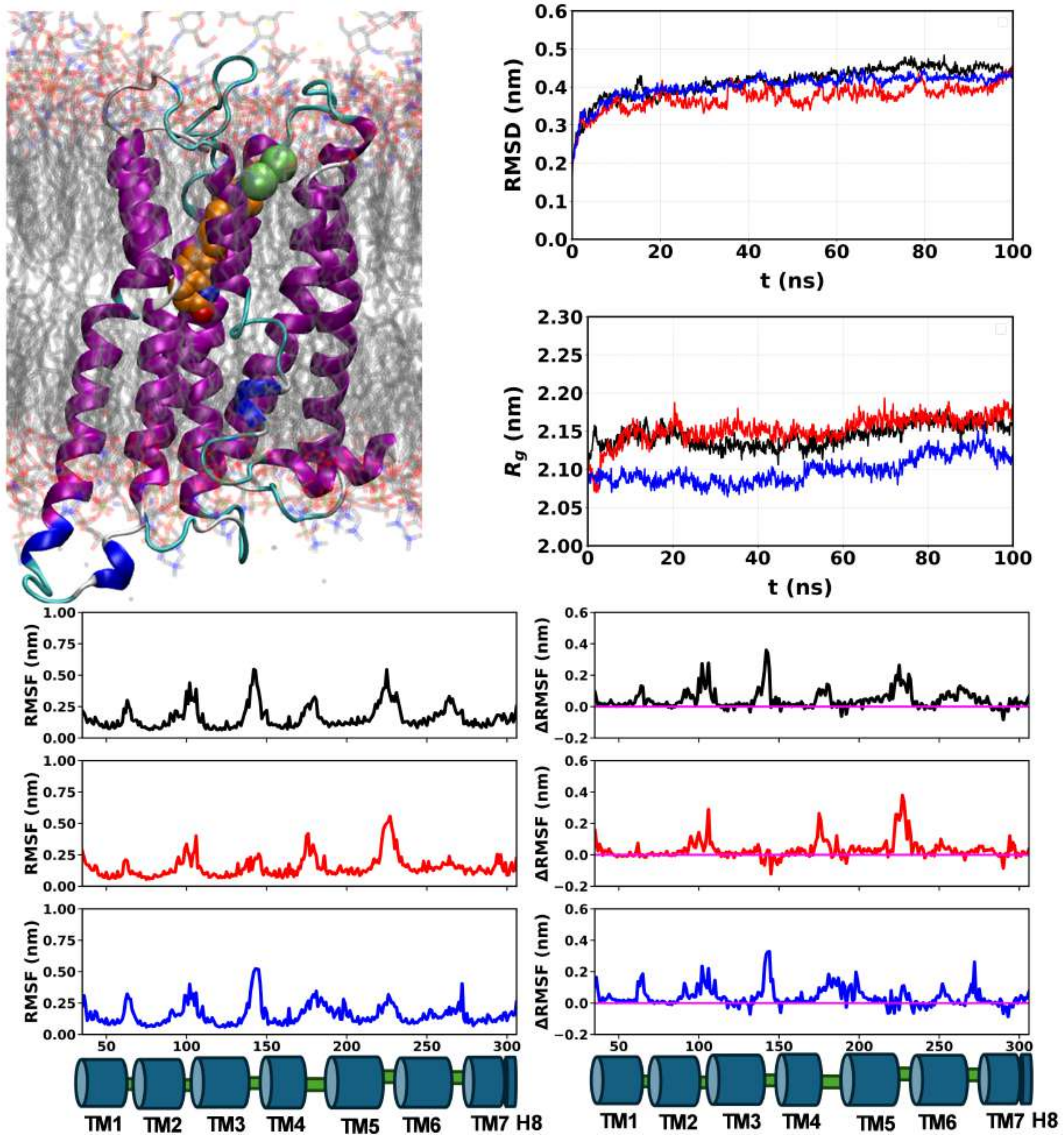

Figure S.7: MD analysis of aripiprazol in complex with D2R. From left to right, top to bottom: RMSD plot, radius of gyration, and RMSF plot. A schematic representation of the TM domains is included for reference. Each MD run is represented by a different color: run 1 (black), run 2 (red), and run 3 (blue)

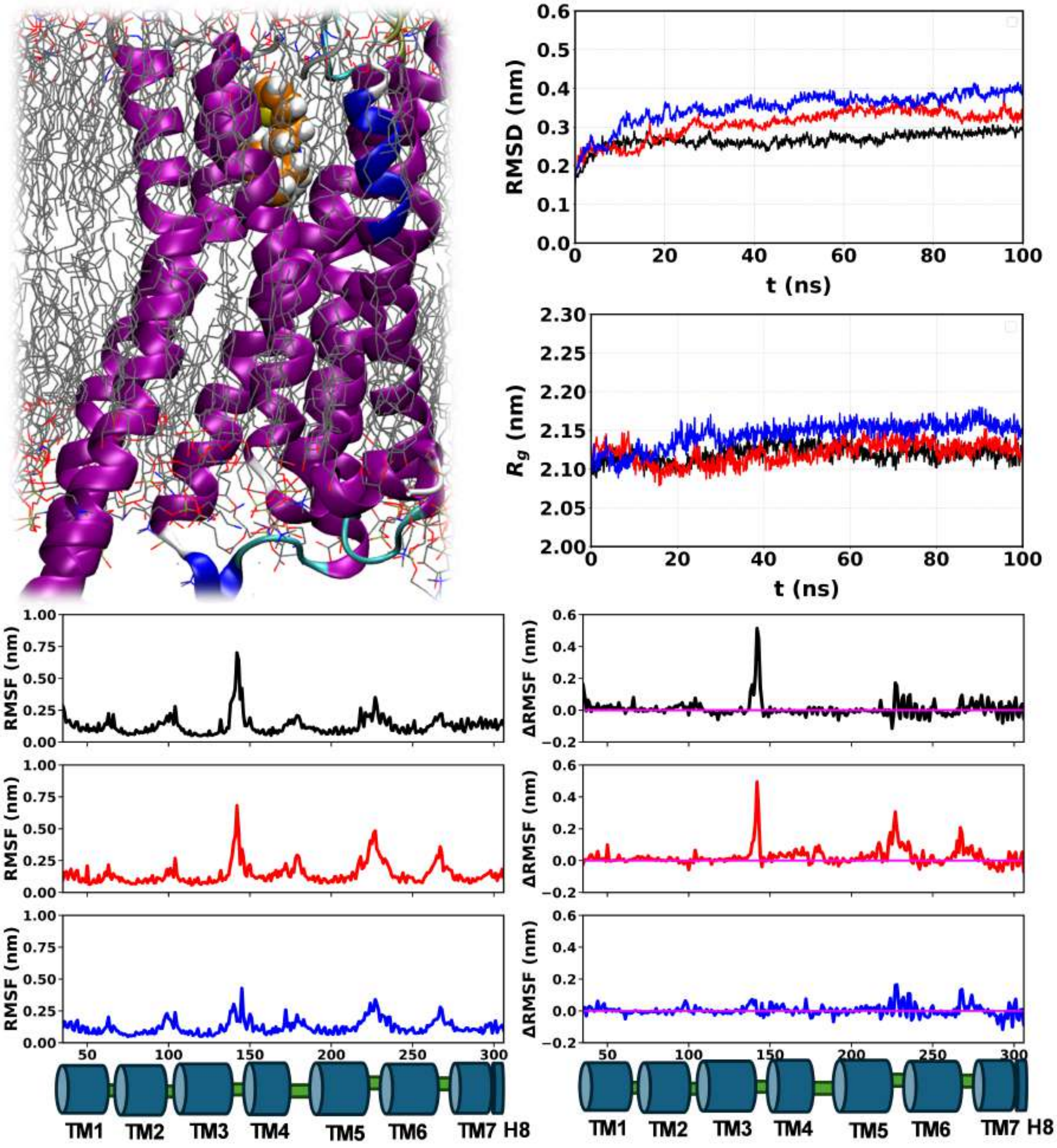

Figure S.8: MD analysis of rotigotine in complex with D2R. From left to right, top to bottom: RMSD plot, radius of gyration, and RMSF plot. A schematic representation of the TM domains is included for reference. Each MD run is represented by a different color: run 1 (black), run 2 (red), and run 3 (blue)

In this section, replica-resolved interaction patterns of PLIP for D2R-ligand complexes in molecular-dynamics simulations are shown. The analysis of the protein-ligand interactions was performed for each of the three MD replicates of D2R bound to haloperidol in the docking-derived posture, haloperidol in the crystallographic pose, risperidone, aripiprazole, dopamine and rotigotine, respectively. Each row of panels corresponds to one independent MD replica. The stacked bars indicate the presence of hydrogen bonds, hydrophobic contacts, π-stacking interactions and salt bridges identified by PLIP for each interacting residue. The profiles were compared descriptively to discover reproducible interaction areas and contacts with replica dependent variations. The vertical axis indicates the proportion of interactions.

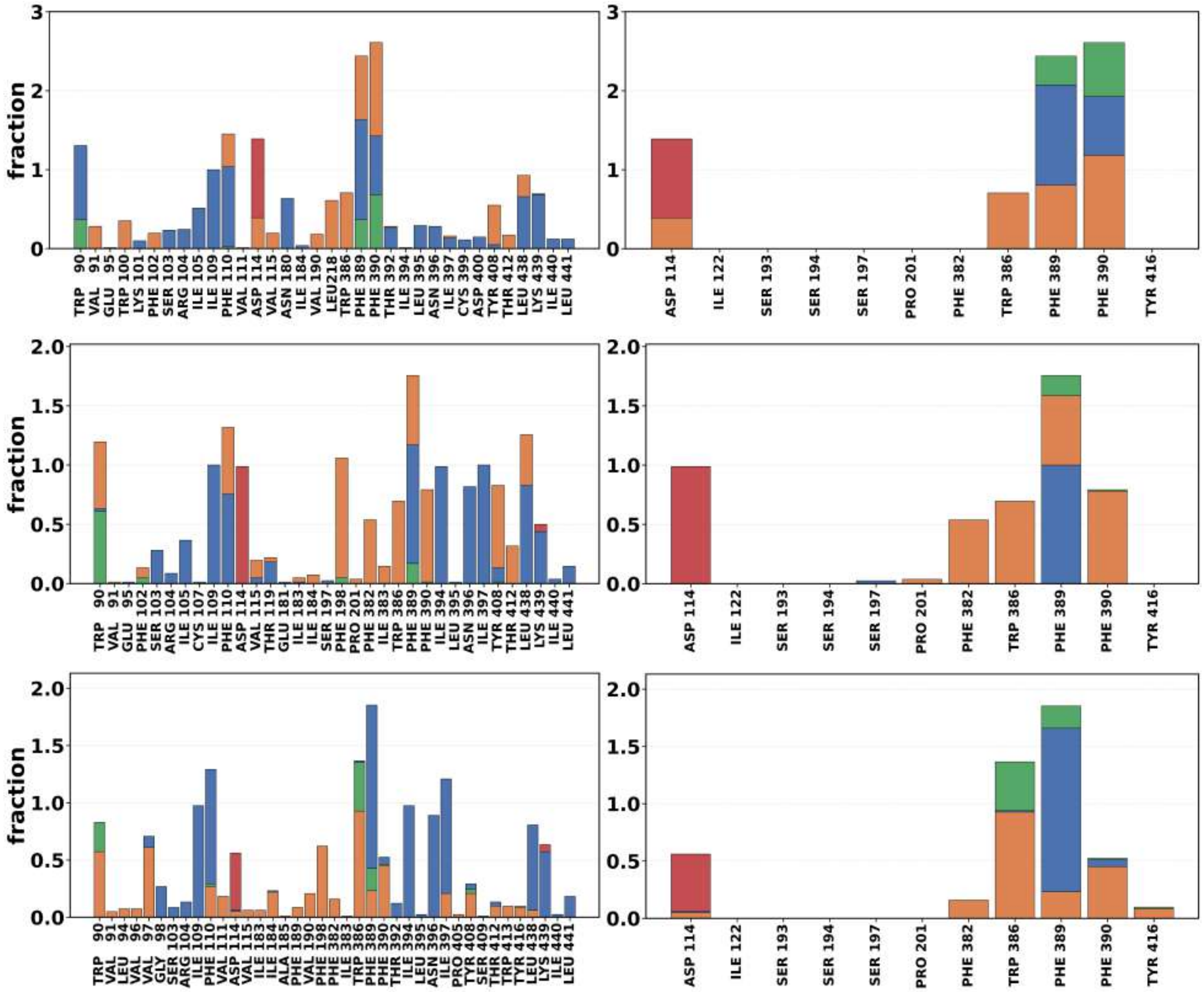

Figure S.9: PLIP interaction patterns resolved for D2R-haloperidol complex via molecular-dynamics simulations. Each row corresponds to an independent MD run, arranged from run 1 (top) to run 3 (bottom).The left column displays all receptor residues involved in ligand interactions. The right column highlights residues belonging to functional microdomains: ionic microdomain (Asp114$^{3.32}$), the PIF connector (Ile122$^{3.40}$), the serine microdomain (Ser193$^{5.42}$, Ser194$^{5.43}$, and Ser197$^{5.46}$), the aromatic microdomain (Trp386$^{6.48}$, Phe389$^{6.51}$, Phe390$^{6.52}$, and His393$^{6.55}$), and Tyr416$^{7.43}$. Colors indicate the interaction type: hydrogen bonds (blue), salt bridges (red), hydrophobic interactions (orange), and π-stacking interactions (green).

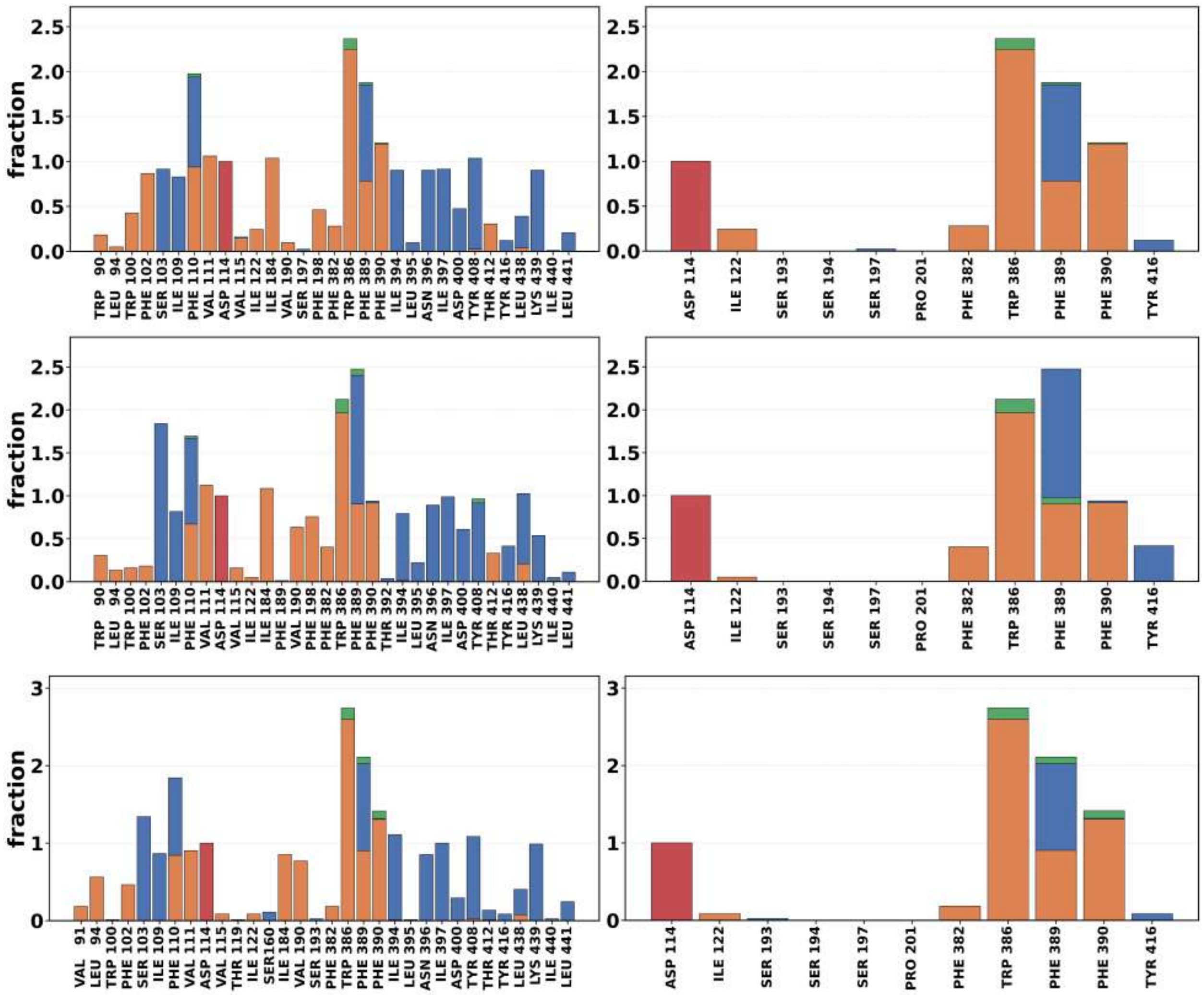

Figure S.10: PLIP interaction patterns resolved for D2R-haloperidol complex (crystal pose) via molecular-dynamics simulations. Each row corresponds to an independent MD run, arranged from run 1 (top) to run 3 (bottom). The left column displays all receptor residues involved in ligand interactions. The right column highlights residues belonging to functional microdomains: ionic microdomain (Asp114$^{3.32}$), the PIF connector (Ile122$^{3.40}$), the serine microdomain (Ser193$^{5.42}$, Ser194$^{5.43}$, and Ser197$^{5.46}$), the aromatic microdomain (Trp386$^{6.48}$, Phe389$^{6.51}$, Phe390$^{6.52}$, and His393$^{6.55}$), and Tyr416$^{7.43}$. Colors indicate the interaction type: hydrogen bonds (blue), salt bridges (red), hydrophobic interactions (orange), and π-stacking interactions (green).

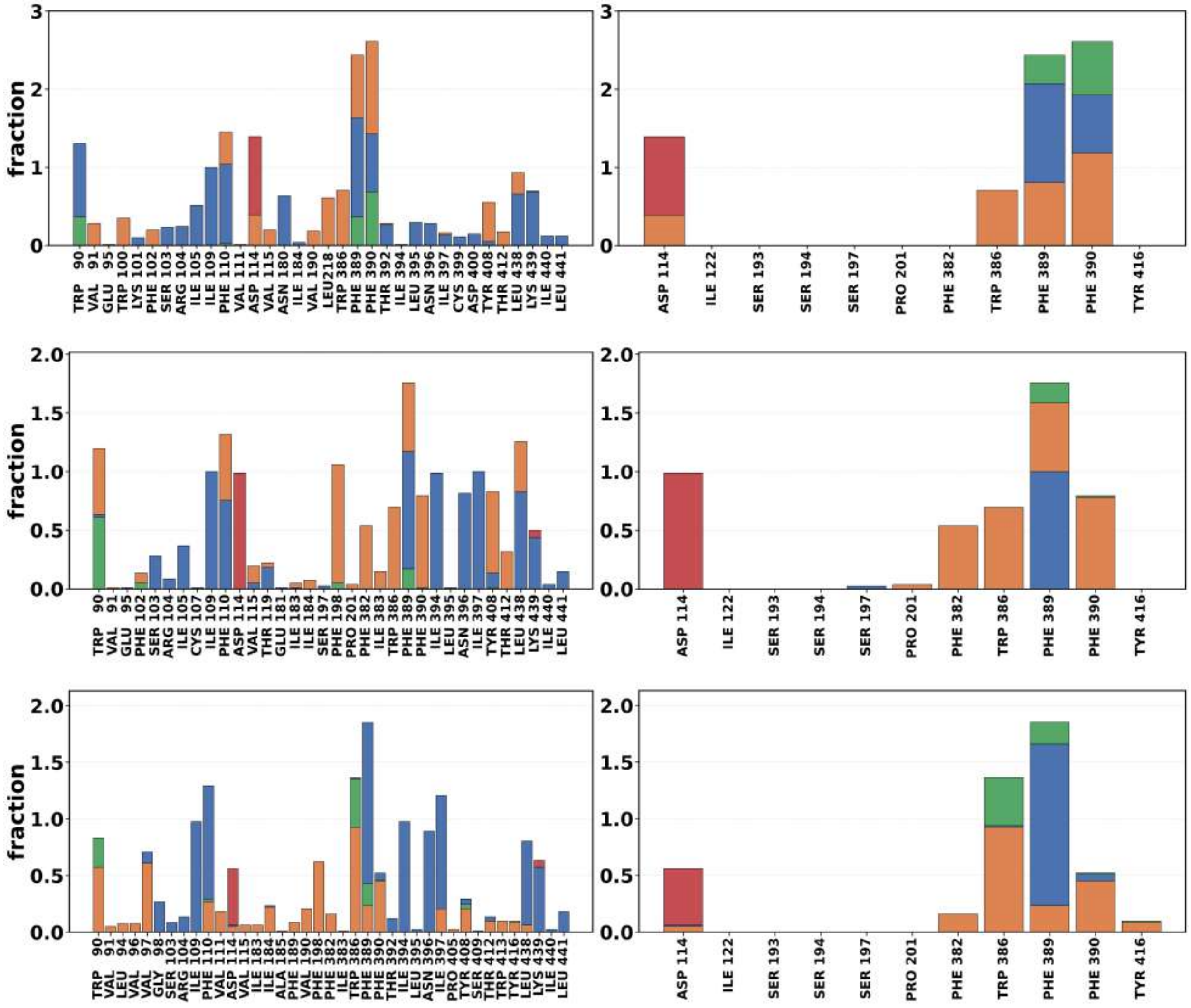

Figure S.11: PLIP interaction patterns resolved for D2R-risperidone complex via molecular-dynamics simulations. Each row corresponds to an independent MD run, arranged from run 1 (top) to run 3 (bottom). The left column displays all receptor residues involved in ligand interactions. The right column highlights residues belonging to functional microdomains: ionic microdomain (Asp114$^{3.32}$), the PIF connector (Ile122$^{3.40}$), the serine microdomain (Ser193$^{5.42}$, Ser194$^{5.43}$, and Ser197$^{5.46}$), the aromatic microdomain (Trp386$^{6.48}$, Phe389$^{6.51}$, Phe390$^{6.52}$, and His393$^{6.55}$), and Tyr416$^{7.43}$. Colors indicate the interaction type: hydrogen bonds (blue), salt bridges (red), hydrophobic interactions (orange), and π-stacking interactions (green).

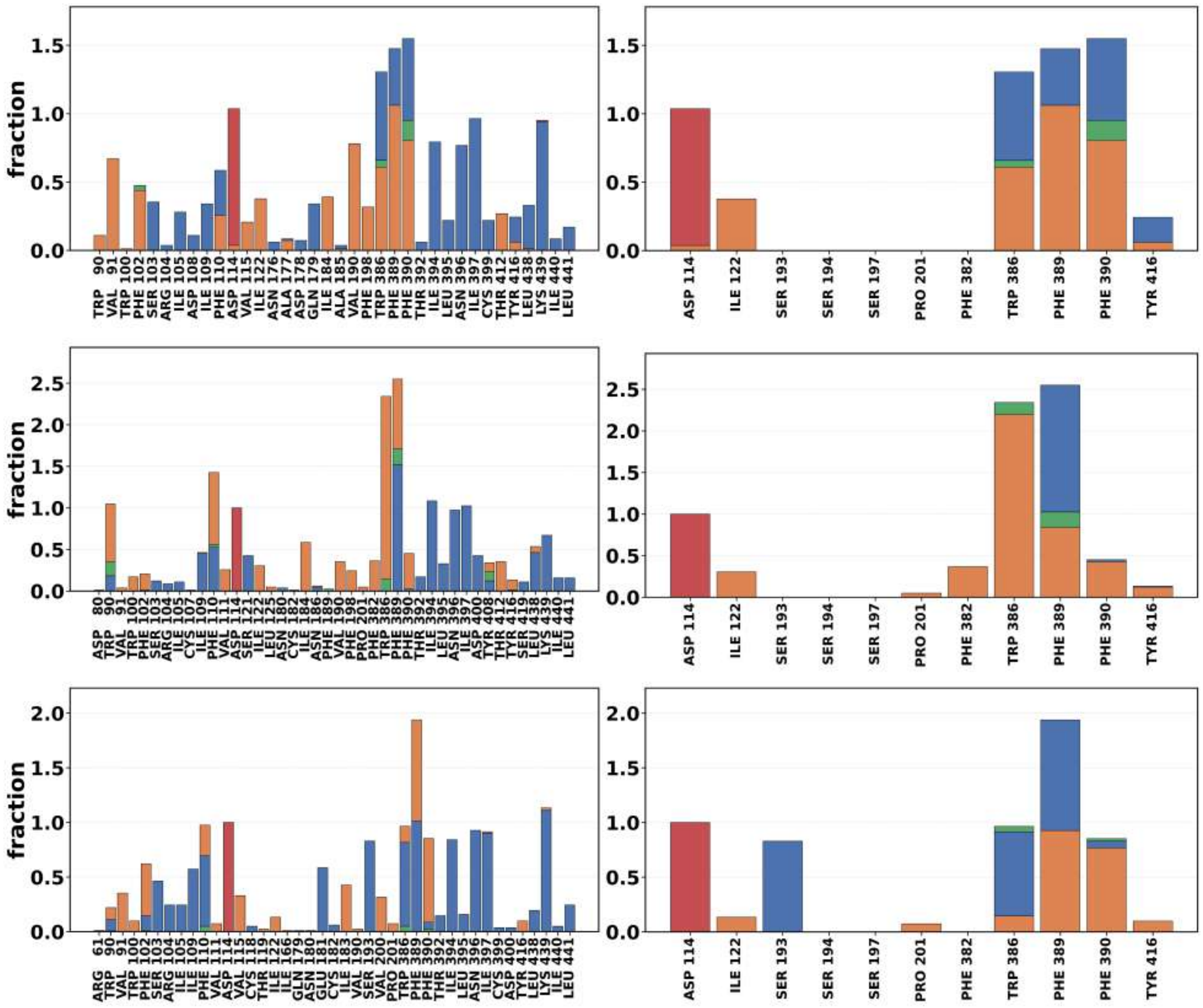

Figure S.12: PLIP interaction patterns resolved for D2R-aripiprazol complex via molecular-dynamics simulations. Each row corresponds to an independent MD run, arranged from run 1 (top) to run 3 (bottom). The left column displays all receptor residues involved in ligand interactions. The right column highlights residues belonging to functional microdomains: ionic microdomain (Asp114$^{3.32}$), the PIF connector (Ile122$^{3.40}$), the serine microdomain (Ser193$^{5.42}$, Ser194$^{5.43}$, and Ser197$^{5.46}$), the aromatic microdomain (Trp386$^{6.48}$, Phe389$^{6.51}$, Phe390$^{6.52}$, and His393$^{6.55}$), and Tyr416$^{7.43}$. Colors indicate the interaction type: hydrogen bonds (blue), salt bridges (red), hydrophobic interactions (orange), and π-stacking interactions (green).

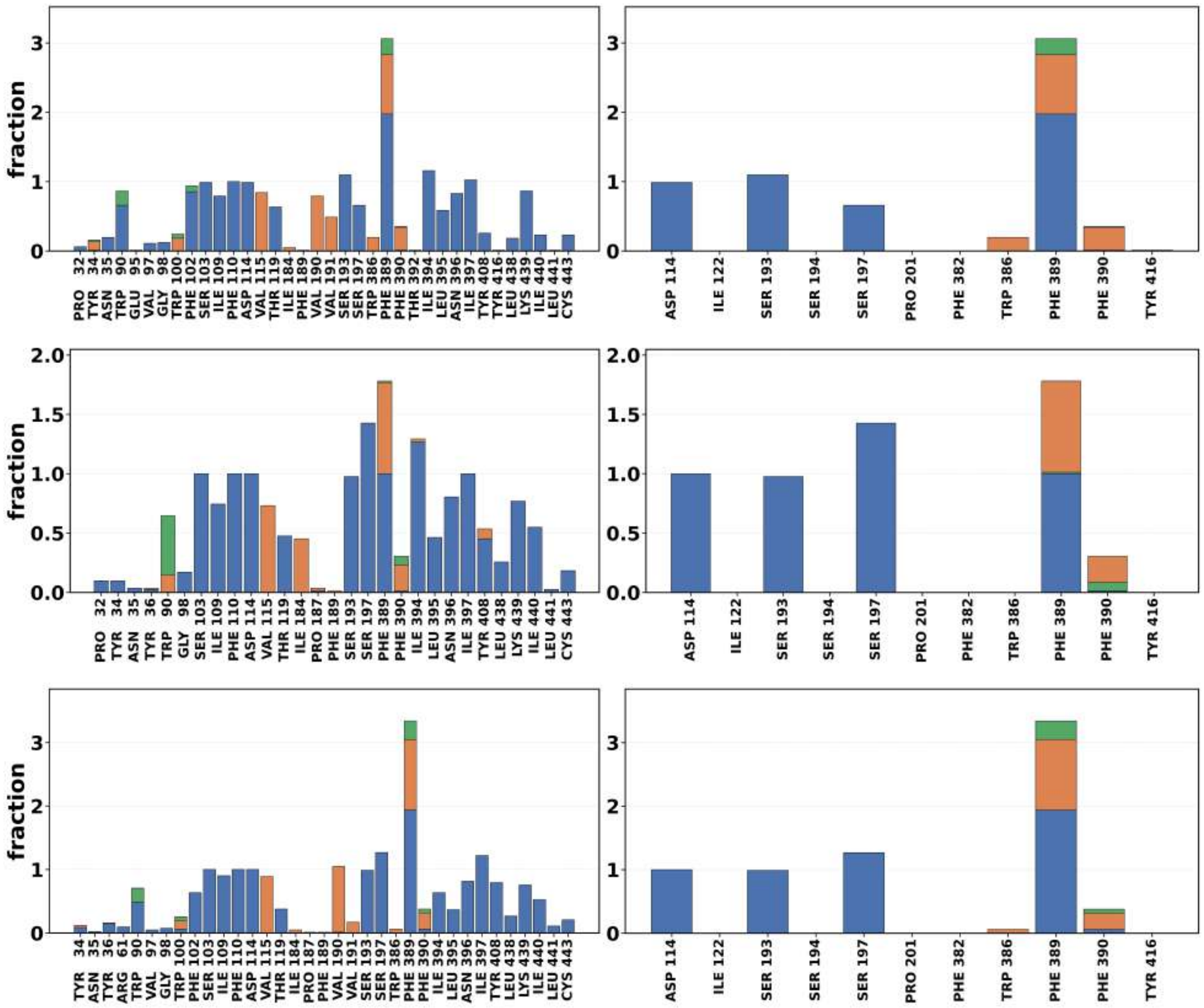

Figure S.13: PLIP interaction patterns resolved for D2R-dopamine complex via molecular-dynamics simulations. Each row corresponds to an independent MD run, arranged from run 1 (top) to run 3 (bottom). The left column displays all receptor residues involved in ligand interactions. The right column highlights residues belonging to functional microdomains: ionic microdomain (Asp114$^{3.32}$), the PIF connector (Ile122$^{3.40}$), the serine microdomain (Ser193$^{5.42}$, Ser194$^{5.43}$, and Ser197$^{5.46}$), the aromatic microdomain (Trp386$^{6.48}$, Phe389$^{6.51}$, Phe390$^{6.52}$, and His393$^{6.55}$), and Tyr416$^{7.43}$. Colors indicate the interaction type: hydrogen bonds (blue), salt bridges (red), hydrophobic interactions (orange), and π-stacking interactions (green).

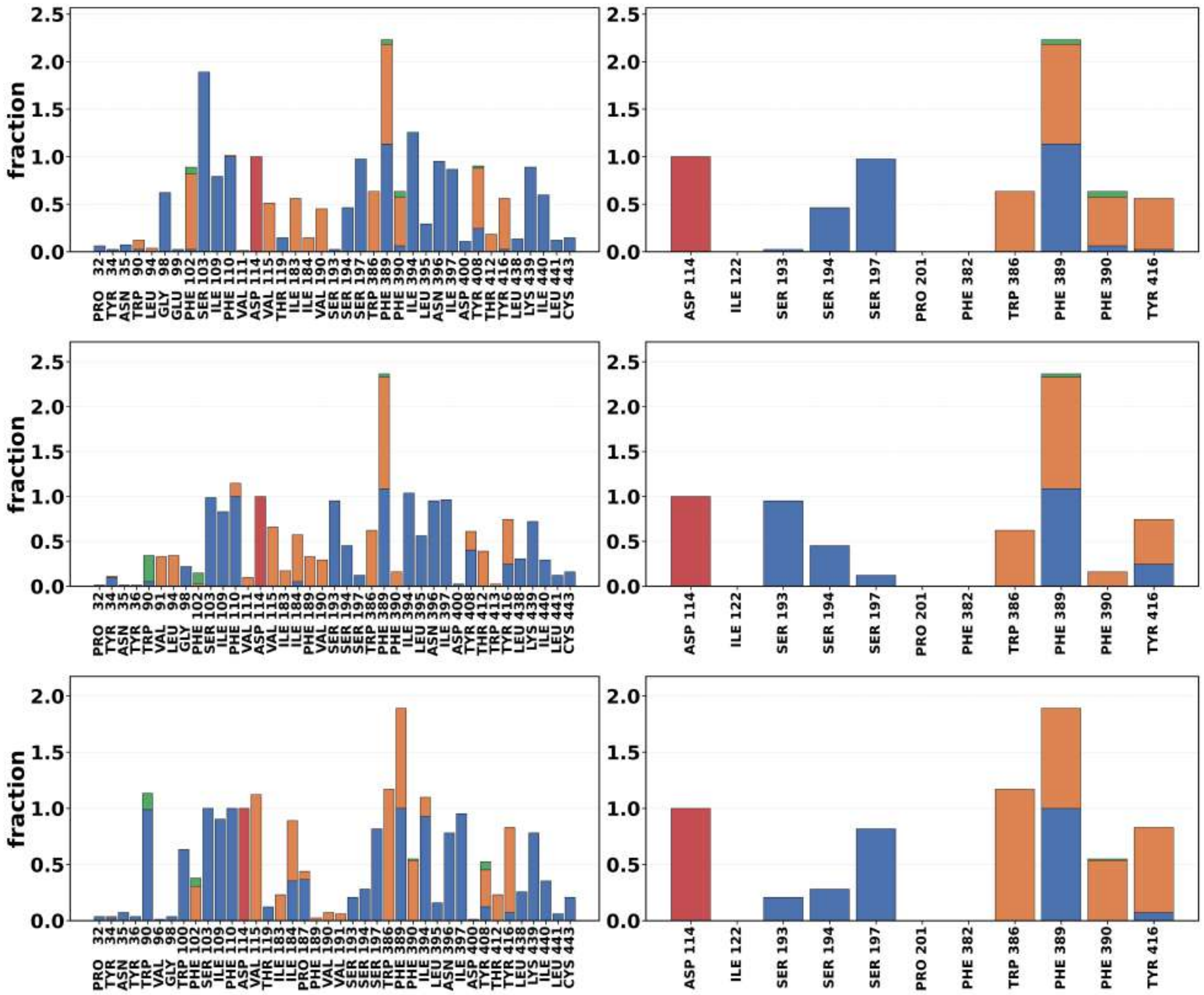

Figure S.14: PLIP interaction patterns resolved for D2R-rotigotine complex via molecular-dynamics simulations. Each row corresponds to an independent MD run, arranged from run 1 (top) to run 3 (bottom). The left column displays all receptor residues involved in ligand interactions. The right column highlights residues belonging to functional microdomains: ionic microdomain (Asp114$^{3.32}$), the PIF connector (Ile122$^{3.40}$), the serine microdomain (Ser193$^{5.42}$, Ser194$^{5.43}$, and Ser197$^{5.46}$), the aromatic microdomain (Trp386$^{6.48}$, Phe389$^{6.51}$, Phe390$^{6.52}$, and His393$^{6.55}$), and Tyr416$^{7.43}$. Colors indicate the interaction type: hydrogen bonds (blue), salt bridges (red), hydrophobic interactions (orange), and π-stacking interactions (green).

In this section, the molecular graph for each system and the distribution of ρ_{bcp} per residue are presented. Additionally, the electron density associated with water interactions is plotted, along with an illustrative image summarizing the main interactions. In the D2R systems docked with antipsychotics, three distinct clusters were identified.

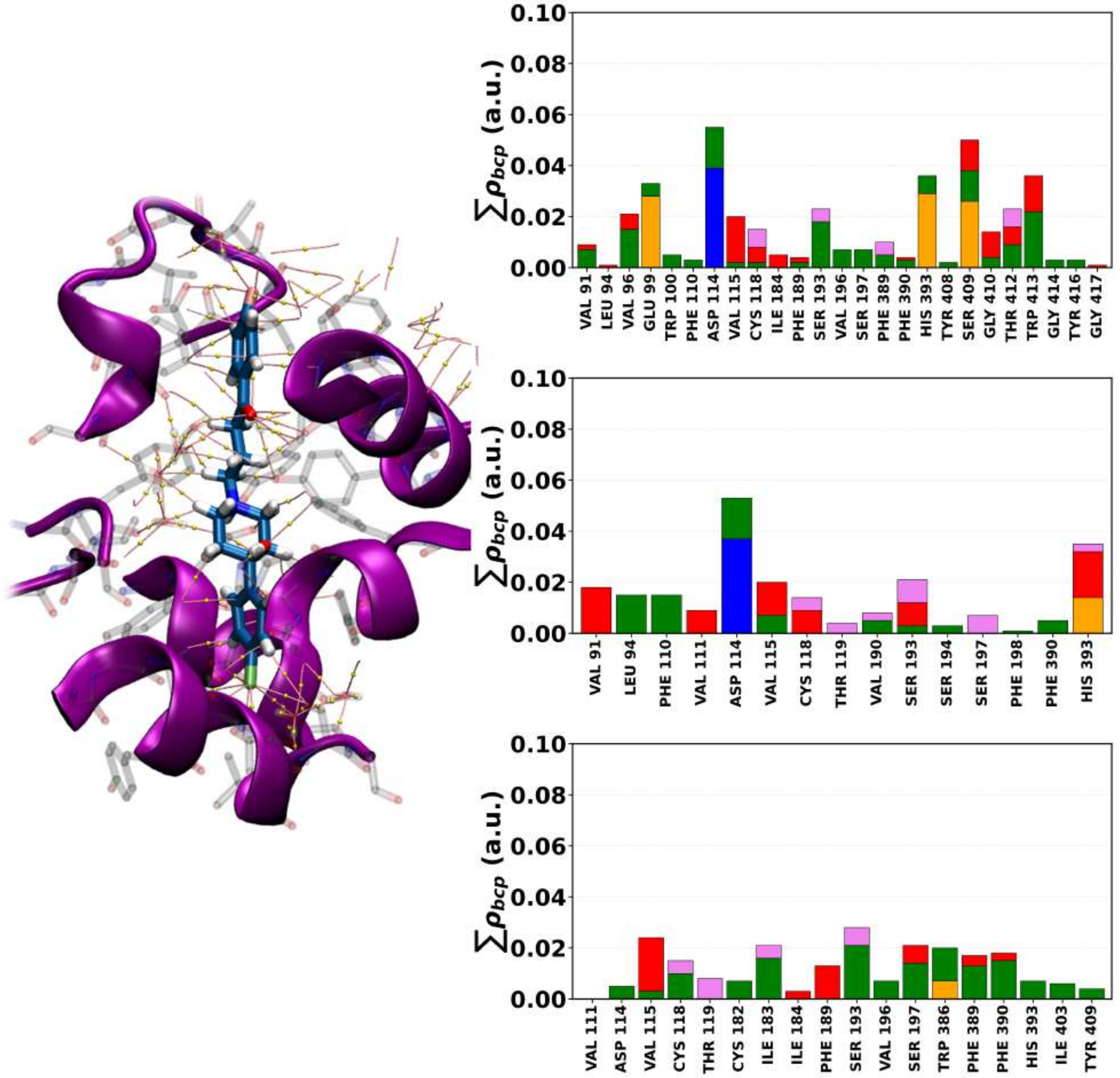

Figure S.15: QTAIM analysis of haloperidol in complex with D2R. On the left, the molecular graph is presented with bond critical points (yellow) and bond paths (pink) that indicate the presence of interactions. On the right, the intermolecular ρ_{bcp} per residue is shown, highlighting the nature of the interactions: charged-assisted hydrogen bond (blue), conventional hydrogen bond, non-conventional hydrogen bond (green), dihydrogen bond (red), and interactions between heteroatoms related to Lewis acid-base interactions (violet).

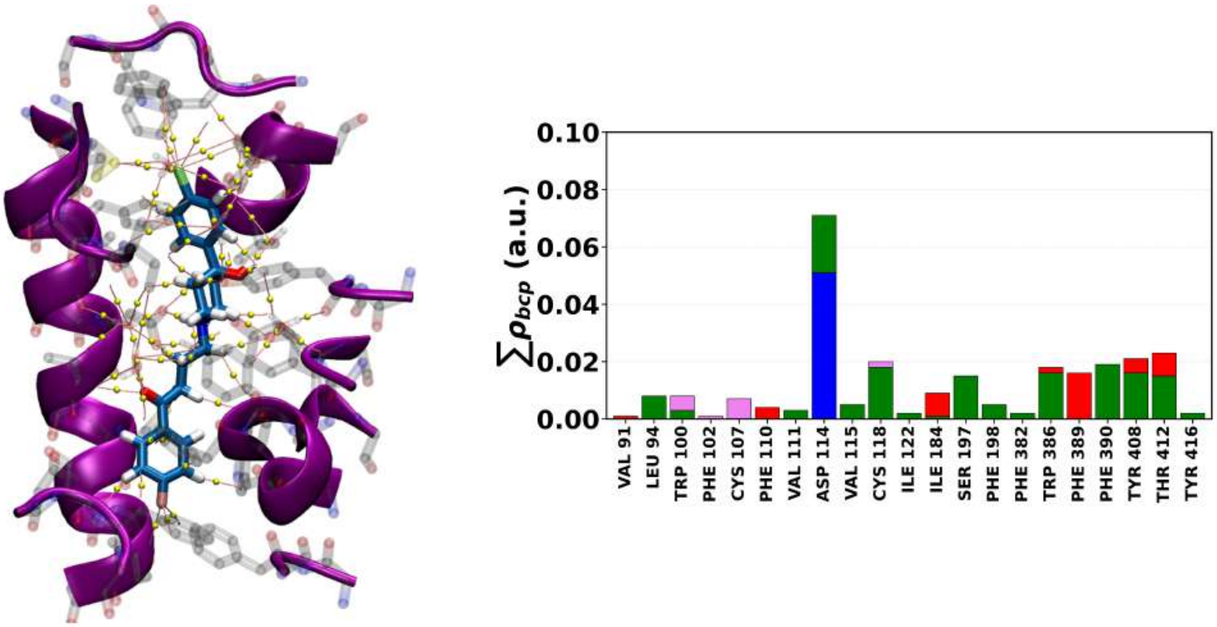

Figure S.16: QTAIM analysis of haloperidol (crystal) in complex with D2R. On the left, the molecular graph is presented with bond critical points (yellow) and bond paths (pink) that indicate the presence of interactions. On the right, the intermolecular ρ_{bcp} per residue is shown, highlighting the nature of the interactions: charged-assisted hydrogen bond (blue), conventional hydrogen bond, non-conventional hydrogen bond (green), dihydrogen bond (red), and interactions between heteroatoms related to Lewis acid-base interactions (violet).

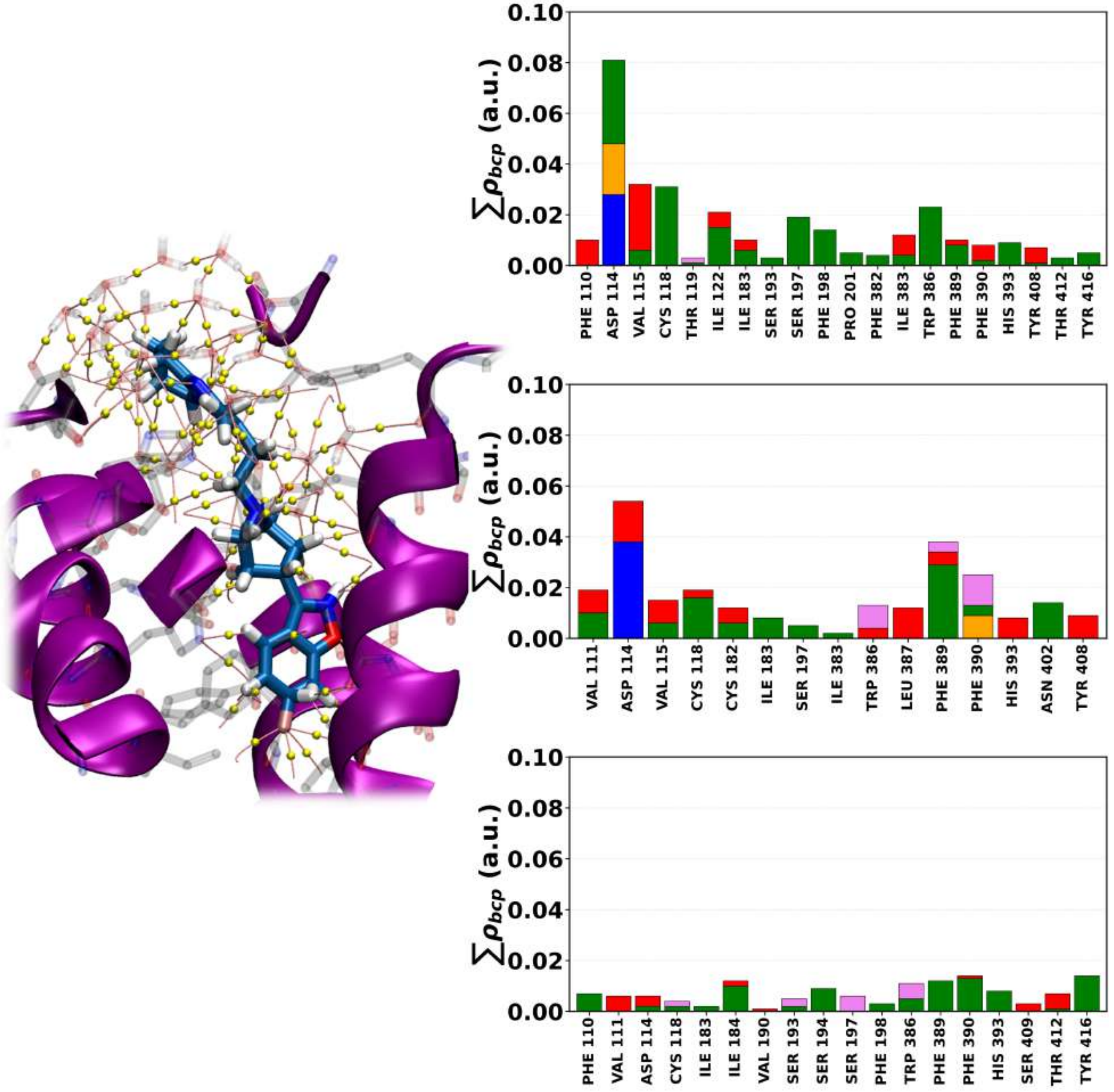

Figure S.17: QTAIM analysis of risperidone in complex with D2R. On the left, the molecular graph is presented with bond critical points (yellow) and bond paths (pink) that indicate the presence of interactions. On the right, the intermolecular ρ_{bcp} per residue is shown, highlighting the nature of the interactions: charged-assisted hydrogen bond (blue), conventional hydrogen bond, non-conventional hydrogen bond (green), dihydrogen bond (red), and interactions between heteroatoms related to Lewis acid-base interactions (violet).

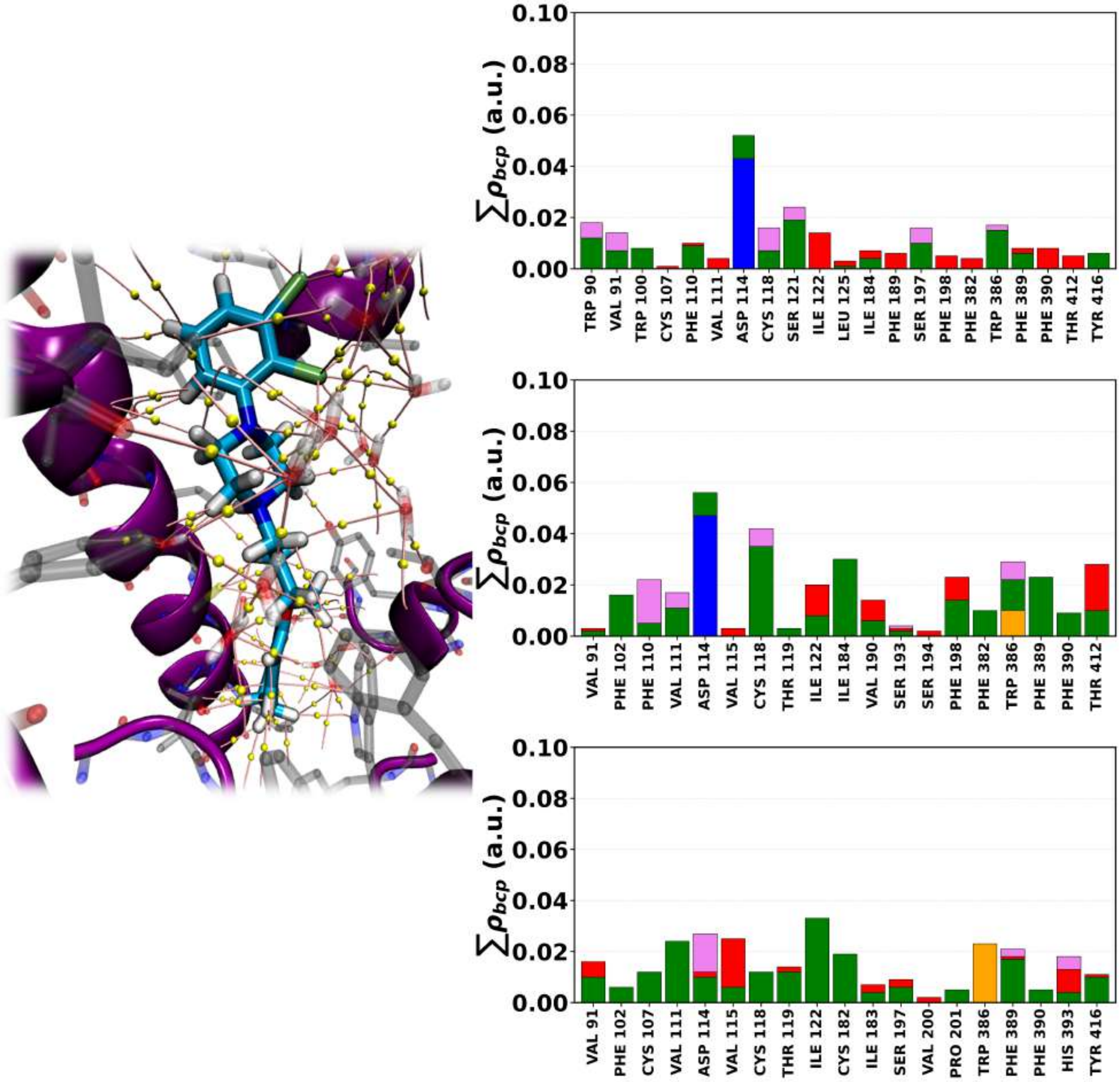

Figure S.18: QTAIM analysis of aripiprazol in complex with D2R. On the left, the molecular graph is presented with bond critical points (yellow) and bond paths (pink) that indicate the presence of interactions. On the right, the intermolecular ρ_{bcp} per residue is shown, highlighting the nature of the interactions: charged-assisted hydrogen bond (blue), conventional hydrogen bond, non-conventional hydrogen bond (green), dihydrogen bond (red), and interactions between heteroatoms related to Lewis acid-base interactions (violet).

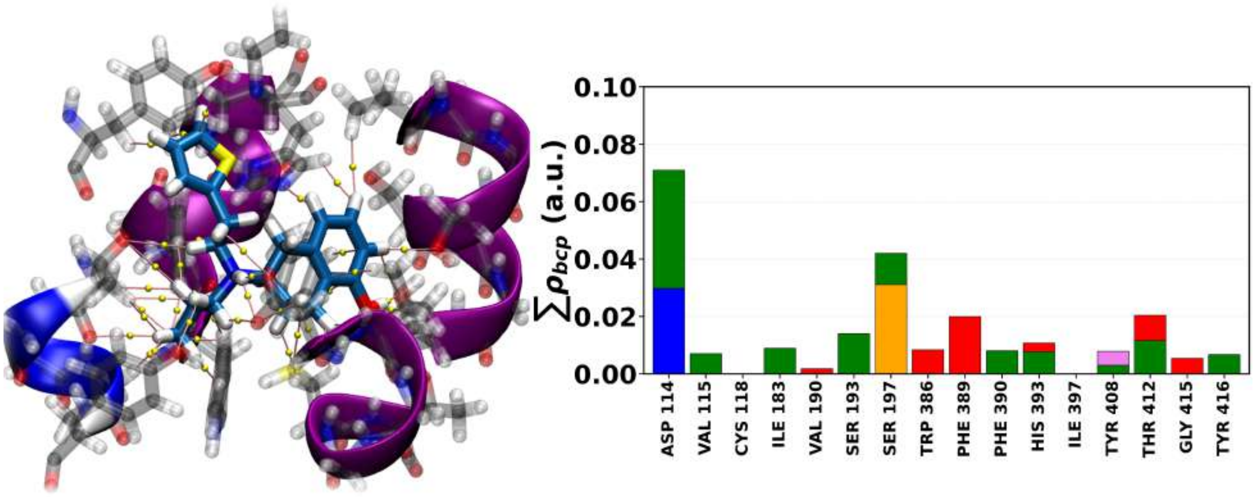

Figure S.19: QTAIM analysis of rotigotine in complex with D2R. On the left, the molecular graph is presented with bond critical points (yellow) and bond paths (pink) that indicate the presence of interactions. On the right, the intermolecular ρ_{bcp} per residue is shown, highlighting the nature of the interactions: charged-assisted hydrogen bond (blue), conventional hydrogen bond, non-conventional hydrogen bond (green), dihydrogen bond (red), and interactions between heteroatoms related to Lewis acid-base interactions (violet).

Table S2: QTAIM bond critical point descriptors for Asp114-ligand interaction ($N^{+}H\cdots O^{-}$).

Descriptor	Units	Interpretation
ρ_{BCP}	a.u.	Electron density at the bond critical point
$\nabla^2\rho_{\mathrm{BCP}}$	a.u.	Laplacian of the electron density
V_{BCP}	a.u.	Potential energy density
G_{BCP}	a.u.	Kinetic energy density
H_{BCP}	a.u.	Total energy density
BD	Dimensionless	Bond degree ($H_{\mathrm{BCP}}/\rho_{\mathrm{BCP}}$)

[a] H_{bcp} normalized by ρ_{bcp} to facilitate comparison. This quantity is also known as the bond degree.[?] .

These figures present a summary of main interactions and ρ_{bcp} distribution for each system.

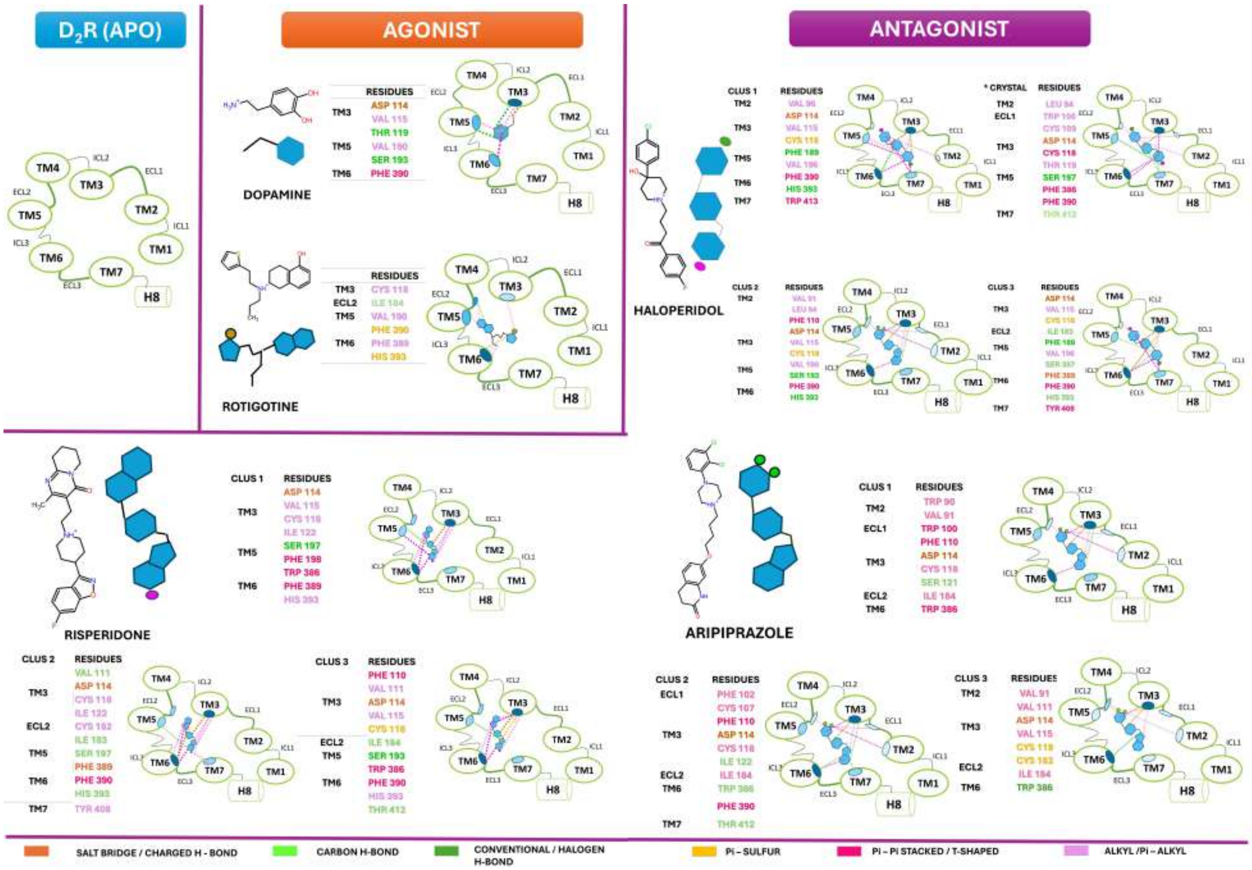

Figure S.20: 2-D binding models in APO, active (agonist bonded) and inactive (antagonist bonded) conformations of D2R. Blue circles represent the strenght of ligand interaction according to QTAIM results. Dashed lines represent interactions predicted with Biova Discovery Studio (vdW interactions are not displayed here).

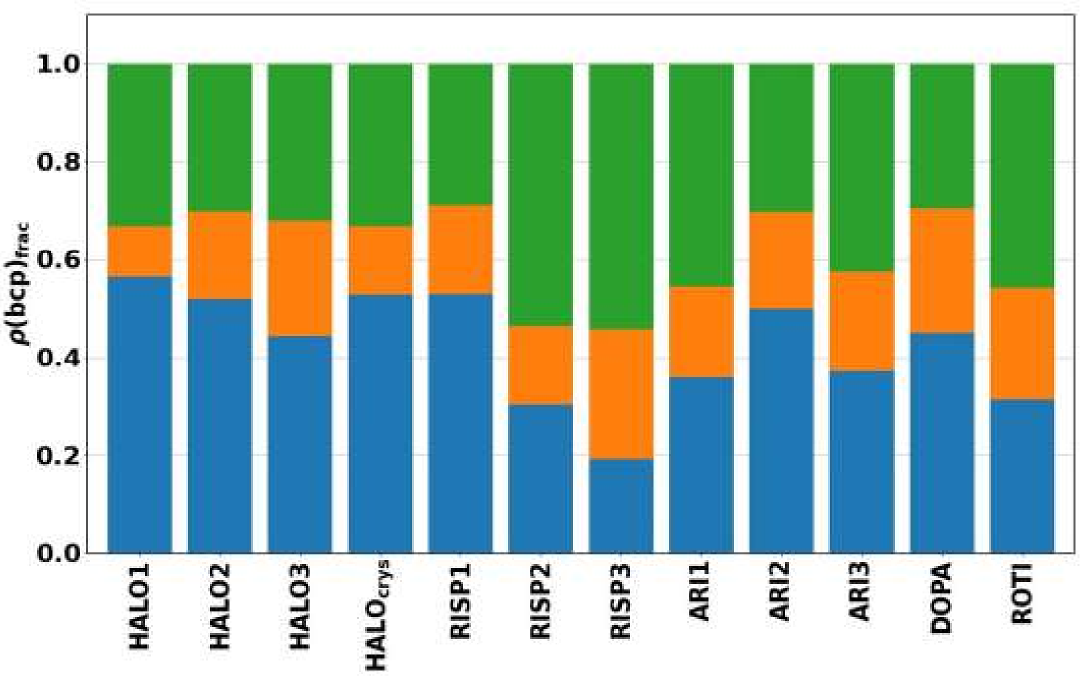

Figure S.21: Fractional $\sum \rho_{bcp}$ distribution of intermolecular interactions within the binding pocket, considering the ligand and water molecules. Only water molecules that mediate interactions between the binding pocket and the ligand are included: Protein-ligand interactions (blue), water-ligand interactions (orange), and protein-water interactions (green).

In this section, the XYZ coordinates of the optimized ligands are provided, along with the level of theory, basis set, and energy convergence criteria used in the optimization.

Haloperidol

```
50
haloperidol PBE0-D3/6-311G** -1.5786397877511229e+03
     C     0.7406106099    1.0787819953    0.0787199465
     C    -0.6135762093    0.8781477840    0.7255040200
     N    -1.3543040615   -0.2622504485    0.0889644218
     C    -0.5378750607   -1.5226546513    0.1321700663
     C     0.8074385455   -1.3200449118   -0.5331402052
     C     1.5873747860   -0.1956775455    0.1589053369
     C     2.9644791496    0.0433482632   -0.4236060164
     C     3.3462954832   -0.4053774439   -1.6858657034
     C     4.6143947442   -0.1380720110   -2.1841129155
     C     5.5121098135    0.5874012768   -1.4134931151
     C     5.1531127673    1.0455783532   -0.1511158390
     C     3.8847272489    0.7724517048    0.3338422223
    Cl     7.0925982201    0.9219880952   -2.0272507659
     O     1.6674130017   -0.4917511407    1.5501183853
     C    -2.7204461512   -0.4322381167    0.6877554368
     C    -3.5953439632   -1.3653597429   -0.1211595066
     C    -5.0211876092   -1.3899512905    0.4054703977
     C    -5.8698028332   -2.3486134146   -0.4235428289
     O    -5.3549264830   -2.9486909989   -1.3428711795
     C    -7.2963538233   -2.5136237624   -0.0771955353
     C    -8.0584556671   -3.3983305919   -0.8486771777
     C    -9.3979056686   -3.5985847155   -0.5764544645
     C    -9.9707146135   -2.9032000185    0.4791207126
     C    -9.2461638156   -2.0196503632    1.2641321697
```

C	-7.9045823513	-1.8288914100	0.9792185161
F	-11.2588591704	-3.0899718057	0.7482065329
H	1.2529863547	1.8883904858	0.6028019306
H	0.6394203331	1.3861441513	-0.9677325405
H	-1.2473810048	1.7620825633	0.6337998205
H	-0.5023954560	0.6231161804	1.7808063294
H	-1.1151808174	-2.3056849125	-0.3570178243
H	-0.4138720867	-1.7648236032	1.1891863900
H	0.6692617659	-1.1015283625	-1.5975301393
H	1.3619430553	-2.2607313748	-0.4715724033
H	2.6674037932	-0.9793978854	-2.3059688129
H	4.9082491493	-0.4933757371	-3.1642360978
H	5.8640418495	1.6075557488	0.4422825946
H	3.6082870004	1.1244726330	1.3222955951
H	2.4265959796	-1.0650538970	1.6922112557
H	-3.1560962988	0.5692439325	0.7360012901
H	-2.5662491733	-0.7844102435	1.7109001145
H	-3.2100138376	-2.3871178228	-0.1145872895
H	-3.6148389621	-1.0540374986	-1.1722565188
H	-5.4742146521	-0.3920739355	0.3721991432
H	-5.0487020564	-1.7068139081	1.4548735470
H	-7.5721302468	-3.9212475416	-1.6639395802
H	-10.0065896940	-4.2787073821	-1.1601455496
H	-9.7396414296	-1.5013261218	2.0772452938
H	-7.3381531224	-1.1378465084	1.5935482538
H	-1.4887873320	-0.0255920492	-0.8978077138

Risperidone

58

risperidone PBE0-D3/6-311G** -1.3612601497678374e+03

C	0.9425867619	2.3376677818	1.7815871637
C	1.5836485331	1.2930401862	0.9276434518
C	0.8955151068	0.3521845428	0.2062157529
C	1.6258346724	-0.6190134580	-0.5595542458
O	1.0972405491	-1.5326697007	-1.1944112660
N	3.0165795879	-0.4785325894	-0.5477756971
C	3.7592658496	-1.4807522940	-1.3340073259
C	5.1829169392	-1.0570508161	-1.6165860173
C	5.8485073969	-0.5820568250	-0.3371758368
C	5.1047452777	0.6395189255	0.1728085170
C	3.6110726987	0.4970953981	0.1920273303
N	2.9407595284	1.3496396389	0.9131139927
C	-0.5914627162	0.1930403492	0.1083234517
C	-1.0277071797	-1.0487256533	0.8837707624
N	-2.2100956696	-1.8010900684	0.3352215640
C	-3.4905997580	-1.0062306683	0.2505143452
C	-3.6710579308	-0.2743466148	-1.0649446754
C	-3.5161100594	-1.2030647028	-2.2757506021
C	-2.1474517494	-1.8900742785	-2.2123999350
C	-1.9162412999	-2.6234725176	-0.9080905882
C	-4.6426423255	-2.1880575620	-2.3936791332
N	-4.8858707166	-3.0535658844	-1.4459681868
O	-5.9647735406	-3.8242193426	-1.8340099553
C	-6.3703831306	-3.4046529662	-3.0447261643
C	-5.5648447696	-2.3493370964	-3.4733161929
C	-5.8010949920	-1.7478729290	-4.7152174921
C	-6.8404638948	-2.2322650736	-5.4784060996
C	-7.6231818663	-3.2967890009	-5.0037600631

C	-7.4244411989	-3.9169062910	-3.7876491803
F	-8.6131812207	-3.7265239021	-5.7798497295
H	1.4440593133	2.3604668561	2.7513079669
H	-0.1253633177	2.1839889614	1.9336140054
H	1.0939253964	3.3212395274	1.3266908440
H	3.1841713431	-1.6480035925	-2.2447665691
H	3.7363961357	-2.4195886933	-0.7705708317
H	5.1936288844	-0.2530233282	-2.3614894414
H	5.7106521290	-1.9069513196	-2.0560483016
H	6.8987302631	-0.3340003458	-0.5037285765
H	5.8249428092	-1.3818324396	0.4122396377
H	5.3222213544	1.5036092038	-0.4680079124
H	5.4044569018	0.9325505594	1.1805339270
H	-0.8261925065	0.0676285606	-0.9485224134
H	-1.1188521370	1.0791817641	0.4626521448
H	-1.2863890285	-0.7905053027	1.9141258183
H	-0.2162547902	-1.7762203994	0.9029853424
H	-3.4917054068	-0.3221678105	1.1013861008
H	-4.2963732893	-1.7320698762	0.3712666487
H	-2.9637003203	0.5530307780	-1.1562935663
H	-4.6716240809	0.1663751398	-1.0481137574
H	-3.5405085693	-0.5838018279	-3.1760739164
H	-1.3483011395	-1.1671306870	-2.3875342253
H	-2.0609580771	-2.6361595172	-3.0072607323
H	-0.8749404524	-2.9364259995	-0.8290720120
H	-2.5865631304	-3.4818136461	-0.8441149246
H	-5.1912885786	-0.9271932100	-5.0755019642
H	-7.0787304740	-1.8139937019	-6.4485847313
H	-8.0484295926	-4.7357741138	-3.4553972547

H	-2.3960785226	-2.5043321269	1.0493307497

Aripiprazole

58

aripiprazole PBE0-D3/6-311G** -2.1268880499676957e+03

C	1.5666917054	-1.1469608215	0.9918879177
C	0.5623955218	-1.6570511040	-0.0352172083
C	0.2207592205	-3.1132393300	0.1678155612
O	-0.8523181435	-3.6091237596	-0.0801009424
N	1.2662724249	-3.8816226458	0.6390342011
C	2.5553958426	-3.4221835145	0.8939358619
C	2.7633676597	-2.0500526247	1.0546777104
C	4.0574166242	-1.6093021175	1.3042967382
C	5.1215109401	-2.4897814908	1.4122147282
C	4.8914406769	-3.8541482695	1.2623831187
C	3.6126565923	-4.3290053374	0.9962352766
O	5.9874075374	-4.6598528707	1.3826105274
C	5.8142425102	-6.0429053116	1.2351042614
C	7.1827398059	-6.6665260401	1.4199977255
C	7.1487000340	-8.1847312558	1.2861717218
C	8.5330029623	-8.7601839861	1.5091573382
N	8.6059037934	-10.2375015517	1.2371273025
C	10.0224770205	-10.7399709504	1.1229740709
C	10.6720269733	-10.8348941000	2.4857008187
N	9.8759818214	-11.7052265206	3.3275039194
C	8.5557820440	-11.1717098245	3.5488515510
C	7.8369266908	-11.0856621745	2.2170860022
C	10.5457681355	-12.2913019457	4.4277070960
C	11.4453003701	-13.3417558329	4.1872807808

C	12.1221008401	-13.9361533180	5.2526302424
C	11.8857774717	-13.5088917962	6.5550929595
C	10.9808774701	-12.4896271890	6.7931614307
C	10.3224596564	-11.8754421847	5.7367319487
Cl	13.2361846618	-15.2211933397	4.9873246235
Cl	11.6711165736	-13.9227400411	2.5747015771
H	1.0875703110	-1.1139239679	1.9796731878
H	1.8689410873	-0.1240215192	0.7555662485
H	0.9828713698	-1.5690689460	-1.0450739947
H	-0.3731459808	-1.0976918632	-0.0210032345
H	1.0702448939	-4.8703640754	0.6908339072
H	4.2347054712	-0.5451835902	1.4260591398
H	6.1287872785	-2.1446928748	1.6102271479
H	3.4138623715	-5.3847597860	0.8541841198
H	5.4126172676	-6.2822145588	0.2391959813
H	5.1087151401	-6.4296901899	1.9855450967
H	7.5607149052	-6.3800460944	2.4070778683
H	7.8626907859	-6.2330384786	0.6794349473
H	6.7899500449	-8.4577928260	0.2845739587
H	6.4349905605	-8.5951740107	2.0080489948
H	8.8793322642	-8.5936829265	2.5290744636
H	9.2521754130	-8.2944111933	0.8309645978
H	10.5563443460	-10.0644780452	0.4529419543
H	9.9676667795	-11.7355567277	0.6778683852
H	10.8118813087	-9.8378217996	2.9364061361
H	11.6651387622	-11.2621338029	2.3437827690
H	8.5561484561	-10.1896950571	4.0541274877
H	7.9872759998	-11.8562788462	4.1820915104
H	6.8371788728	-10.6634696947	2.3129852484

```
H     7.7694605739  -12.0807347114    1.7736607172
H    12.4149321515  -13.9892040426    7.3686382203
H    10.7980116100  -12.1611504944    7.8094742837
H     9.6343407698  -11.0612081499    5.9338457578
H     8.1682317482  -10.3757704787    0.3247162392
```

Dopamine

```
23
dopamine PBE0-D3/6-311G** -5.1660687586077086e+02
C     0.0213052674    1.4557091843    0.5174617135
C    -0.1081005749    2.7519413272    1.0107336996
C    -0.7317703632    2.9723410195    2.2254289212
C    -1.2436142817    1.8916558092    2.9651797736
C    -1.1093702238    0.6069118752    2.4686335149
C    -0.4795256410    0.3823944045    1.2423395387
C    -0.3049691202   -1.0288460354    0.7467220965
C     0.9071482200   -1.6455439773    1.4206776828
N     1.0995683141   -3.0856530755    0.9860859633
O    -1.8589898740    2.0985078247    4.1392729998
O    -0.9187496354    4.1828935525    2.8045704625
H     0.4994135285    1.2976923544   -0.4438476721
H     0.2720211896    3.5930997808    0.4388801594
H    -1.5322070289   -0.2065639487    3.0505369224
H    -0.1734995631   -1.0310703761   -0.3411158587
H    -1.2004279065   -1.6205466426    0.9737087522
H     0.8026671724   -1.6603096487    2.5066235279
H     1.8257360571   -1.1162675585    1.1626657698
H     0.2773562121   -3.6474638454    1.2198974805
H     1.9118151864   -3.5171036785    1.4325394211
```

H	-1.8773974159	3.0481935399	4.3078119282
H	-0.6001705477	4.8900957253	2.2388248481
H	1.2297610287	-3.1460676106	-0.0266316452

Rotigotine

48

rotigotine PBE0-D3/6-311G** -1.2667445387146111e+03

C	-2.1690876777	0.4193221466	1.1386025917
C	-2.5878166316	-0.0105469638	-0.2616621010
C	-1.9657849549	0.8268819418	-1.3654936365
N	-0.4718620726	0.6758598770	-1.4285977057
C	-0.0867229952	-0.7375747742	-1.8082721791
C	0.6238791845	-1.4743637131	-0.6800800389
C	0.9839991836	-2.8587453989	-1.1056253705
C	2.2093339683	-3.3302090655	-1.4837615624
C	2.1823093380	-4.6946587180	-1.8770264902
C	0.9358357424	-5.2372932286	-1.7965912597
S	-0.2189921060	-4.0960341053	-1.2326680867
C	0.2376152332	1.7018629920	-2.2997875815
C	1.7365544248	1.6126804326	-2.0582710931
C	2.4775124005	2.4881620913	-3.0577200754
C	1.8838389790	3.8622572756	-3.1618827202
C	0.6111093536	4.1633366083	-2.6854300157
C	-0.2440292290	3.1140148905	-2.0110354244
C	0.0945229374	5.4529480463	-2.8146721450
C	0.8510597900	6.4456171599	-3.4138267455
C	2.1209336054	6.1619377388	-3.8991330304
C	2.6301271969	4.8759738317	-3.7786885779
O	3.8517605998	4.5193444414	-4.2381891882

H	-2.7032522182	-0.1610589714	1.8926157714
H	-2.3941754279	1.4750925962	1.3151343082
H	-1.1015142068	0.2651427518	1.3352671716
H	-3.6703882818	0.1053937413	-0.3684769698
H	-2.3910949887	-1.0762611579	-0.4171498234
H	-2.1779166333	1.8794045594	-1.1845101490
H	-2.3430660366	0.5610089645	-2.3554235352
H	0.5499904719	-0.6852424848	-2.6906173374
H	-1.0003479563	-1.2579117232	-2.0947582070
H	-0.0131977358	-1.4958642785	0.2115880469
H	1.5418168756	-0.9430615297	-0.4066667352
H	3.1073111184	-2.7232782297	-1.4685447168
H	3.0520495494	-5.2518736219	-2.1994934747
H	0.6239232178	-6.2465644388	-2.0221423581
H	-0.0040704849	1.4184994534	-3.3297788705
H	2.1042562896	0.5868372327	-2.1388301502
H	1.9455481104	1.9595184013	-1.0370645764
H	2.4764455077	2.0087130816	-4.0451657326
H	3.5297972159	2.5622893020	-2.7725454074
H	-1.2731502165	3.2559145285	-2.3488242569
H	-0.2341632362	3.2825035904	-0.9233235100
H	-0.9007580108	5.6767526365	-2.4445255258
H	0.4523261521	7.4489043381	-3.5115385345
H	2.7135154890	6.9376090150	-4.3756158154
H	4.2881618945	5.2824412818	-4.6253336355
H	-0.1271427286	0.8393174553	-0.4784635401

In this section, the coordinate files (PDB format) corresponding to the reduced models of each system, generated via GROMACS clustering, are provided.

Haloperidol (Cluster structure 1)

ATOM	1	N	GLY	A	76	-1.375	8.262	1.662	1.00	0.00	N
ATOM	2	CA	GLY	A	76	-0.594	7.311	0.969	1.00	0.00	C
ATOM	3	C	GLY	A	76	-1.313	6.192	0.254	1.00	0.00	C
ATOM	4	O	GLY	A	76	-1.126	5.925	-0.940	1.00	0.00	O
ATOM	5	H1	GLY	A	76	-1.275	9.124	1.182	1.00	0.00	H
ATOM	6	H2	GLY	A	76	-1.186	8.142	2.628	1.00	0.00	H
ATOM	7	HA2	GLY	A	76	-0.135	7.897	0.173	1.00	0.00	H
ATOM	8	HA3	GLY	A	76	0.142	6.846	1.685	1.00	0.00	H
ATOM	9	N	VAL	A	77	-2.279	5.682	1.015	1.00	0.00	N
ATOM	10	CA	VAL	A	77	-3.284	4.754	0.425	1.00	0.00	C
ATOM	11	CB	VAL	A	77	-3.983	4.085	1.543	1.00	0.00	C
ATOM	12	CG1	VAL	A	77	-5.267	3.237	1.075	1.00	0.00	C
ATOM	13	CG2	VAL	A	77	-2.969	3.069	2.152	1.00	0.00	C
ATOM	14	C	VAL	A	77	-4.254	5.332	-0.617	1.00	0.00	C
ATOM	15	O	VAL	A	77	-4.567	4.664	-1.555	1.00	0.00	O
ATOM	16	H	VAL	A	77	-2.430	5.991	1.949	1.00	0.00	H
ATOM	17	HA	VAL	A	77	-2.785	3.880	0.025	1.00	0.00	H
ATOM	18	HB	VAL	A	77	-4.223	4.810	2.331	1.00	0.00	H
ATOM	19	HG11	VAL	A	77	-5.129	2.739	0.086	1.00	0.00	H
ATOM	20	HG12	VAL	A	77	-6.125	3.941	1.023	1.00	0.00	H
ATOM	21	HG13	VAL	A	77	-5.442	2.425	1.820	1.00	0.00	H
ATOM	22	HG21	VAL	A	77	-1.893	3.376	2.233	1.00	0.00	H
ATOM	23	HG22	VAL	A	77	-2.985	2.179	1.489	1.00	0.00	H
ATOM	24	HG23	VAL	A	77	-3.357	2.710	3.117	1.00	0.00	H
ATOM	25	N	GLY	A	78	-4.699	6.658	-0.417	1.00	0.00	N
ATOM	26	CA	GLY	A	78	-5.668	7.245	-1.449	1.00	0.00	C

```
ATOM     27  C   GLY A  78      -4.974   7.305  -2.743  1.00  0.00           C
ATOM     28  O   GLY A  78      -5.497   6.854  -3.758  1.00  0.00           O
ATOM     29  H   GLY A  78      -4.390   7.199   0.377  1.00  0.00           H
ATOM     30  HA2 GLY A  78      -6.568   6.643  -1.575  1.00  0.00           H
ATOM     31  HA3 GLY A  78      -5.943   8.227  -1.097  1.00  0.00           H
ATOM     32  N   GLY A  79      -3.791   7.873  -2.729  1.00  0.00           N
ATOM     33  CA  GLY A  79      -2.950   7.953  -3.907  1.00  0.00           C
ATOM     34  C   GLY A  79      -2.458   6.641  -4.529  1.00  0.00           C
ATOM     35  O   GLY A  79      -2.378   6.336  -5.741  1.00  0.00           O
ATOM     36  H   GLY A  79      -3.453   8.270  -1.864  1.00  0.00           H
ATOM     37  HA2 GLY A  79      -3.607   8.382  -4.663  1.00  0.00           H
ATOM     38  HA3 GLY A  79      -2.051   8.578  -3.664  1.00  0.00           H
ATOM     39  N   ASP A  80      -2.138   5.719  -3.643  1.00  0.00           N
ATOM     40  CA  ASP A  80      -1.921   4.259  -3.869  1.00  0.00           C
ATOM     41  CB  ASP A  80      -0.447   4.169  -4.322  1.00  0.00           C
ATOM     42  CG  ASP A  80       0.081   2.730  -4.368  1.00  0.00           C
ATOM     43  OD1 ASP A  80       1.198   2.451  -4.885  1.00  0.00           O
ATOM     44  OD2 ASP A  80      -0.494   1.885  -3.700  1.00  0.00           O1-
ATOM     45  C   ASP A  80      -2.852   3.561  -4.806  1.00  0.00           C
ATOM     46  O   ASP A  80      -2.529   2.922  -5.804  1.00  0.00           O
ATOM     47  H   ASP A  80      -1.951   6.090  -2.739  1.00  0.00           H
ATOM     48  HA  ASP A  80      -2.059   3.721  -2.947  1.00  0.00           H
ATOM     49  HB2 ASP A  80       0.175   4.583  -3.502  1.00  0.00           H
ATOM     50  HB3 ASP A  80      -0.216   4.657  -5.289  1.00  0.00           H
ATOM     51  N   VAL A  81      -4.187   3.759  -4.494  1.00  0.00           N
ATOM     52  CA  VAL A  81      -5.299   3.280  -5.279  1.00  0.00           C
ATOM     53  CB  VAL A  81      -6.556   3.478  -4.480  1.00  0.00           C
ATOM     54  CG1 VAL A  81      -7.809   3.075  -5.237  1.00  0.00           C
ATOM     55  CG2 VAL A  81      -6.502   2.568  -3.224  1.00  0.00           C
```

ATOM	56	C	VAL	A	81	-5.355	3.860	-6.653	1.00	0.00	C
ATOM	57	O	VAL	A	81	-5.602	3.166	-7.644	1.00	0.00	O
ATOM	58	H	VAL	A	81	-4.521	4.294	-3.738	1.00	0.00	H
ATOM	59	HA	VAL	A	81	-5.206	2.227	-5.500	1.00	0.00	H
ATOM	60	HB	VAL	A	81	-6.635	4.549	-4.229	1.00	0.00	H
ATOM	61	HG11	VAL	A	81	-7.638	2.014	-5.511	1.00	0.00	H
ATOM	62	HG12	VAL	A	81	-7.993	3.770	-6.094	1.00	0.00	H
ATOM	63	HG13	VAL	A	81	-8.659	3.158	-4.535	1.00	0.00	H
ATOM	64	HG21	VAL	A	81	-5.658	2.859	-2.565	1.00	0.00	H
ATOM	65	HG22	VAL	A	81	-6.323	1.539	-3.637	1.00	0.00	H
ATOM	66	HG23	VAL	A	81	-7.434	2.683	-2.648	1.00	0.00	H
ATOM	67	N	GLY	A	82	-5.083	5.152	-6.849	1.00	0.00	N
ATOM	68	CA	GLY	A	82	-5.009	5.787	-8.156	1.00	0.00	C
ATOM	69	C	GLY	A	82	-3.929	5.197	-8.990	1.00	0.00	C
ATOM	70	O	GLY	A	82	-4.243	4.834	-10.059	1.00	0.00	O
ATOM	71	H	GLY	A	82	-4.917	5.727	-6.036	1.00	0.00	H
ATOM	72	HA2	GLY	A	82	-5.952	5.625	-8.677	1.00	0.00	H
ATOM	73	HA3	GLY	A	82	-4.826	6.860	-8.015	1.00	0.00	H
ATOM	74	N	GLY	A	83	-2.652	5.211	-8.543	1.00	0.00	N
ATOM	75	CA	GLY	A	83	-1.517	4.633	-9.278	1.00	0.00	C
ATOM	76	C	GLY	A	83	-1.659	3.190	-9.741	1.00	0.00	C
ATOM	77	O	GLY	A	83	-1.489	2.762	-10.869	1.00	0.00	O
ATOM	78	H	GLY	A	83	-2.467	5.645	-7.650	1.00	0.00	H
ATOM	79	HA2	GLY	A	83	-1.455	5.210	-10.201	1.00	0.00	H
ATOM	80	HA3	GLY	A	83	-0.609	4.713	-8.634	1.00	0.00	H
ATOM	81	N	CYS	A	84	-2.093	2.332	-8.748	1.00	0.00	N
ATOM	82	CA	CYS	A	84	-2.395	0.981	-8.922	1.00	0.00	C
ATOM	83	CB	CYS	A	84	-2.674	0.320	-7.619	1.00	0.00	C
ATOM	84	SG	CYS	A	84	-1.232	0.230	-6.510	1.00	0.00	S

ATOM	85	C	CYS	A	84	-3.598	0.797	-9.816	1.00	0.00	C
ATOM	86	O	CYS	A	84	-3.663	-0.215	-10.591	1.00	0.00	O
ATOM	87	H	CYS	A	84	-2.210	2.576	-7.786	1.00	0.00	H
ATOM	88	HA	CYS	A	84	-1.515	0.547	-9.375	1.00	0.00	H
ATOM	89	HB2	CYS	A	84	-3.070	-0.686	-7.851	1.00	0.00	H
ATOM	90	HB3	CYS	A	84	-3.507	0.755	-6.998	1.00	0.00	H
ATOM	91	HG	CYS	A	84	-1.521	1.485	-6.217	1.00	0.00	H
ATOM	92	N	THR	A	85	-4.639	1.620	-9.729	1.00	0.00	N
ATOM	93	CA	THR	A	85	-5.828	1.454	-10.573	1.00	0.00	C
ATOM	94	CB	THR	A	85	-7.053	2.006	-10.006	1.00	0.00	C
ATOM	95	OG1	THR	A	85	-7.312	1.364	-8.782	1.00	0.00	O
ATOM	96	CG2	THR	A	85	-8.393	1.884	-10.733	1.00	0.00	C
ATOM	97	C	THR	A	85	-5.725	2.075	-11.872	1.00	0.00	C
ATOM	98	O	THR	A	85	-6.247	1.533	-12.869	1.00	0.00	O
ATOM	99	H	THR	A	85	-4.602	2.481	-9.217	1.00	0.00	H
ATOM	100	HA	THR	A	85	-5.974	0.396	-10.706	1.00	0.00	H
ATOM	101	HB	THR	A	85	-7.002	3.115	-9.899	1.00	0.00	H
ATOM	102	HG21	THR	A	85	-8.800	0.852	-10.636	1.00	0.00	H
ATOM	103	HG22	THR	A	85	-8.296	2.278	-11.767	1.00	0.00	H
ATOM	104	HG23	THR	A	85	-9.036	2.601	-10.192	1.00	0.00	H
ATOM	105	HG1	THR	A	85	-6.997	1.957	-8.097	1.00	0.00	H
ATOM	106	N	GLY	A	86	-4.933	3.155	-12.051	1.00	0.00	N
ATOM	107	CA	GLY	A	86	-4.651	3.686	-13.388	1.00	0.00	C
ATOM	108	C	GLY	A	86	-3.751	2.744	-14.195	1.00	0.00	C
ATOM	109	O	GLY	A	86	-3.981	2.546	-15.403	1.00	0.00	O
ATOM	110	H	GLY	A	86	-4.526	3.607	-11.245	1.00	0.00	H
ATOM	111	HA2	GLY	A	86	-5.599	3.788	-13.917	1.00	0.00	H
ATOM	112	HA3	GLY	A	86	-4.153	4.654	-13.291	1.00	0.00	H
ATOM	113	HC	GLY	A	86	-2.917	2.252	-13.716	1.00	0.00	H

ATOM	114	N	GLY	A	147	-5.746	0.600	10.832	1.00	0.00	N
ATOM	115	CA	GLY	A	147	-4.595	1.465	11.225	1.00	0.00	C
ATOM	116	C	GLY	A	147	-3.401	1.629	10.337	1.00	0.00	C
ATOM	117	O	GLY	A	147	-2.262	1.178	10.656	1.00	0.00	O
ATOM	118	H1	GLY	A	147	-6.466	1.217	10.657	1.00	0.00	H
ATOM	119	H2	GLY	A	147	-5.701	-0.153	11.432	1.00	0.00	H
ATOM	120	HA2	GLY	A	147	-5.015	2.470	11.175	1.00	0.00	H
ATOM	121	HA3	GLY	A	147	-4.248	1.098	12.244	1.00	0.00	H
ATOM	122	N	CYS	A	148	-3.590	2.280	9.210	1.00	0.00	N
ATOM	123	CA	CYS	A	148	-2.575	2.646	8.293	1.00	0.00	C
ATOM	124	CB	CYS	A	148	-1.329	3.428	8.791	1.00	0.00	C
ATOM	125	SG	CYS	A	148	-1.884	5.026	9.522	1.00	0.00	S
ATOM	126	C	CYS	A	148	-2.147	1.510	7.338	1.00	0.00	C
ATOM	127	O	CYS	A	148	-1.040	1.586	6.811	1.00	0.00	O
ATOM	128	H	CYS	A	148	-4.516	2.544	8.948	1.00	0.00	H
ATOM	129	HA	CYS	A	148	-3.057	3.390	7.679	1.00	0.00	H
ATOM	130	HB2	CYS	A	148	-0.676	3.614	7.912	1.00	0.00	H
ATOM	131	HB3	CYS	A	148	-0.698	2.965	9.568	1.00	0.00	H
ATOM	132	HG	CYS	A	148	-2.663	5.669	8.648	1.00	0.00	H
ATOM	133	N	ILE	A	149	-3.026	0.542	7.003	1.00	0.00	N
ATOM	134	CA	ILE	A	149	-2.720	-0.407	5.957	1.00	0.00	C
ATOM	135	CB	ILE	A	149	-4.013	-0.725	5.232	1.00	0.00	C
ATOM	136	CG2	ILE	A	149	-3.865	-1.490	3.996	1.00	0.00	C
ATOM	137	CG1	ILE	A	149	-5.258	-1.165	6.135	1.00	0.00	C
ATOM	138	CD1	ILE	A	149	-6.594	-1.146	5.381	1.00	0.00	C
ATOM	139	C	ILE	A	149	-2.193	-1.778	6.473	1.00	0.00	C
ATOM	140	O	ILE	A	149	-1.321	-2.388	5.817	1.00	0.00	O
ATOM	141	H	ILE	A	149	-3.740	0.338	7.663	1.00	0.00	H
ATOM	142	HA	ILE	A	149	-2.017	0.061	5.284	1.00	0.00	H

ATOM	143	HB	ILE	A	149	-4.313	0.283	4.909	1.00	0.00	H
ATOM	144	HG12	ILE	A	149	-5.501	-0.594	7.059	1.00	0.00	H
ATOM	145	HG13	ILE	A	149	-5.071	-2.202	6.502	1.00	0.00	H
ATOM	146	HG21	ILE	A	149	-3.138	-0.926	3.385	1.00	0.00	H
ATOM	147	HG22	ILE	A	149	-4.823	-1.498	3.449	1.00	0.00	H
ATOM	148	HG23	ILE	A	149	-3.458	-2.499	4.226	1.00	0.00	H
ATOM	149	HD11	ILE	A	149	-6.591	-1.913	4.570	1.00	0.00	H
ATOM	150	HD12	ILE	A	149	-6.607	-0.195	4.805	1.00	0.00	H
ATOM	151	HD13	ILE	A	149	-7.437	-1.265	6.087	1.00	0.00	H
ATOM	152	N	ILE	A	150	-2.648	-2.254	7.618	1.00	0.00	N
ATOM	153	CA	ILE	A	150	-2.214	-3.516	8.260	1.00	0.00	C
ATOM	154	CB	ILE	A	150	-2.592	-3.596	9.723	1.00	0.00	C
ATOM	155	CG2	ILE	A	150	-2.043	-4.901	10.350	1.00	0.00	C
ATOM	156	CG1	ILE	A	150	-1.961	-2.365	10.424	1.00	0.00	C
ATOM	157	CD1	ILE	A	150	-0.373	-2.285	10.351	1.00	0.00	C
ATOM	158	C	ILE	A	150	-2.779	-4.785	7.566	1.00	0.00	C
ATOM	159	O	ILE	A	150	-2.060	-5.631	7.019	1.00	0.00	O
ATOM	160	H	ILE	A	150	-3.376	-1.706	8.027	1.00	0.00	H
ATOM	161	HA	ILE	A	150	-1.134	-3.549	8.172	1.00	0.00	H
ATOM	162	HB	ILE	A	150	-3.687	-3.533	9.872	1.00	0.00	H
ATOM	163	HG12	ILE	A	150	-2.264	-2.283	11.487	1.00	0.00	H
ATOM	164	HG13	ILE	A	150	-2.242	-1.406	9.948	1.00	0.00	H
ATOM	165	HG21	ILE	A	150	-2.612	-5.826	10.076	1.00	0.00	H
ATOM	166	HG22	ILE	A	150	-2.156	-4.842	11.457	1.00	0.00	H
ATOM	167	HG23	ILE	A	150	-0.974	-5.080	10.119	1.00	0.00	H
ATOM	168	HD11	ILE	A	150	0.163	-3.064	10.922	1.00	0.00	H
ATOM	169	HD12	ILE	A	150	0.033	-1.302	10.673	1.00	0.00	H
ATOM	170	HD13	ILE	A	150	-0.076	-2.326	9.278	1.00	0.00	H
ATOM	171	N	GLY	A	151	-4.081	-4.821	7.330	1.00	0.00	N

ATOM	172	CA	GLY	A	151	-4.681	-5.842	6.533	1.00	0.00	C
ATOM	173	C	GLY	A	151	-5.851	-5.428	5.754	1.00	0.00	C
ATOM	174	O	GLY	A	151	-5.938	-5.522	4.543	1.00	0.00	O
ATOM	175	H	GLY	A	151	-4.669	-4.102	7.727	1.00	0.00	H
ATOM	176	HA2	GLY	A	151	-3.933	-6.123	5.791	1.00	0.00	H
ATOM	177	HA3	GLY	A	151	-4.963	-6.694	7.203	1.00	0.00	H
ATOM	178	HC	GLY	A	151	-6.692	-5.012	6.289	1.00	0.00	H
ATOM	179	N	GLY	A	154	-12.091	-5.510	3.736	1.00	0.00	N
ATOM	180	CA	GLY	A	154	-12.714	-4.731	2.736	1.00	0.00	C
ATOM	181	C	GLY	A	154	-11.809	-4.116	1.630	1.00	0.00	C
ATOM	182	O	GLY	A	154	-12.160	-4.240	0.428	1.00	0.00	O
ATOM	183	H1	GLY	A	154	-12.400	-6.437	3.599	1.00	0.00	H
ATOM	184	H2	GLY	A	154	-12.179	-5.003	4.578	1.00	0.00	H
ATOM	185	HA2	GLY	A	154	-13.348	-5.459	2.230	1.00	0.00	H
ATOM	186	HA3	GLY	A	154	-13.273	-3.881	3.209	1.00	0.00	H
ATOM	187	N	PHE	A	155	-10.686	-3.601	2.055	1.00	0.00	N
ATOM	188	CA	PHE	A	155	-9.645	-3.039	1.263	1.00	0.00	C
ATOM	189	CB	PHE	A	155	-8.713	-2.240	2.182	1.00	0.00	C
ATOM	190	CG	PHE	A	155	-7.462	-1.825	1.489	1.00	0.00	C
ATOM	191	CD1	PHE	A	155	-7.414	-0.473	1.073	1.00	0.00	C
ATOM	192	CE1	PHE	A	155	-6.328	0.012	0.357	1.00	0.00	C
ATOM	193	CZ	PHE	A	155	-5.181	-0.869	0.054	1.00	0.00	C
ATOM	194	CD2	PHE	A	155	-6.353	-2.623	1.280	1.00	0.00	C
ATOM	195	CE2	PHE	A	155	-5.194	-2.149	0.662	1.00	0.00	C
ATOM	196	C	PHE	A	155	-9.052	-4.085	0.384	1.00	0.00	C
ATOM	197	O	PHE	A	155	-8.762	-3.879	-0.841	1.00	0.00	O
ATOM	198	H	PHE	A	155	-10.515	-3.572	3.041	1.00	0.00	H
ATOM	199	HA	PHE	A	155	-9.932	-2.246	0.583	1.00	0.00	H
ATOM	200	HB2	PHE	A	155	-8.305	-2.829	3.024	1.00	0.00	H

ATOM	201	HB3	PHE	A	155	-9.248	-1.321	2.545	1.00	0.00	H
ATOM	202	HD1	PHE	A	155	-8.157	0.219	1.437	1.00	0.00	H
ATOM	203	HD2	PHE	A	155	-6.276	-3.599	1.732	1.00	0.00	H
ATOM	204	HE1	PHE	A	155	-6.339	1.019	-0.067	1.00	0.00	H
ATOM	205	HE2	PHE	A	155	-4.348	-2.830	0.595	1.00	0.00	H
ATOM	206	HZ	PHE	A	155	-4.324	-0.513	-0.487	1.00	0.00	H
ATOM	207	N	GLY	A	156	-8.913	-5.352	0.987	1.00	0.00	N
ATOM	208	CA	GLY	A	156	-8.429	-6.542	0.339	1.00	0.00	C
ATOM	209	C	GLY	A	156	-9.363	-6.943	-0.790	1.00	0.00	C
ATOM	210	O	GLY	A	156	-8.928	-7.372	-1.847	1.00	0.00	O
ATOM	211	H	GLY	A	156	-9.176	-5.431	1.959	1.00	0.00	H
ATOM	212	HA2	GLY	A	156	-7.438	-6.339	-0.066	1.00	0.00	H
ATOM	213	HA3	GLY	A	156	-8.376	-7.358	1.061	1.00	0.00	H
ATOM	214	HC	GLY	A	156	-10.430	-6.847	-0.655	1.00	0.00	H
ATOM	215	N	GLY	A	158	-11.759	-4.669	-2.320	1.00	0.00	N
ATOM	216	CA	GLY	A	158	-11.980	-3.449	-3.071	1.00	0.00	C
ATOM	217	C	GLY	A	158	-10.896	-3.160	-4.062	1.00	0.00	C
ATOM	218	O	GLY	A	158	-11.150	-2.973	-5.239	1.00	0.00	O
ATOM	219	H1	GLY	A	158	-12.498	-5.290	-2.586	1.00	0.00	H
ATOM	220	H2	GLY	A	158	-11.628	-4.383	-1.369	1.00	0.00	H
ATOM	221	HA2	GLY	A	158	-12.900	-3.580	-3.641	1.00	0.00	H
ATOM	222	HA3	GLY	A	158	-12.061	-2.606	-2.362	1.00	0.00	H
ATOM	223	N	SER	A	159	-9.653	-3.262	-3.657	1.00	0.00	N
ATOM	224	CA	SER	A	159	-8.415	-3.196	-4.409	1.00	0.00	C
ATOM	225	CB	SER	A	159	-7.094	-3.127	-3.512	1.00	0.00	C
ATOM	226	OG	SER	A	159	-7.258	-2.008	-2.706	1.00	0.00	O
ATOM	227	C	SER	A	159	-8.247	-4.424	-5.317	1.00	0.00	C
ATOM	228	O	SER	A	159	-7.745	-4.326	-6.453	1.00	0.00	O
ATOM	229	H	SER	A	159	-9.516	-3.440	-2.686	1.00	0.00	H

ATOM	230	HA	SER	A	159	-8.420	-2.326	-5.057	1.00	0.00	H
ATOM	231	HB2	SER	A	159	-6.249	-3.168	-4.223	1.00	0.00	H
ATOM	232	HB3	SER	A	159	-7.026	-3.986	-2.816	1.00	0.00	H
ATOM	233	HG	SER	A	159	-7.297	-2.296	-1.792	1.00	0.00	H
ATOM	234	N	GLY	A	160	-8.704	-5.639	-4.913	1.00	0.00	N
ATOM	235	CA	GLY	A	160	-8.841	-6.791	-5.849	1.00	0.00	C
ATOM	236	C	GLY	A	160	-9.786	-6.495	-6.990	1.00	0.00	C
ATOM	237	O	GLY	A	160	-9.551	-6.891	-8.099	1.00	0.00	O
ATOM	238	H	GLY	A	160	-8.960	-5.766	-3.944	1.00	0.00	H
ATOM	239	HA2	GLY	A	160	-7.866	-6.997	-6.291	1.00	0.00	H
ATOM	240	HA3	GLY	A	160	-9.208	-7.651	-5.279	1.00	0.00	H
ATOM	241	N	GLY	A	161	-10.996	-5.889	-6.712	1.00	0.00	N
ATOM	242	CA	GLY	A	161	-12.032	-5.636	-7.730	1.00	0.00	C
ATOM	243	C	GLY	A	161	-11.598	-4.714	-8.903	1.00	0.00	C
ATOM	244	O	GLY	A	161	-11.802	-4.966	-10.073	1.00	0.00	O
ATOM	245	H	GLY	A	161	-11.181	-5.604	-5.761	1.00	0.00	H
ATOM	246	HA2	GLY	A	161	-12.250	-6.608	-8.172	1.00	0.00	H
ATOM	247	HA3	GLY	A	161	-12.909	-5.179	-7.239	1.00	0.00	H
ATOM	248	N	VAL	A	162	-10.934	-3.611	-8.516	1.00	0.00	N
ATOM	249	CA	VAL	A	162	-10.780	-2.432	-9.352	1.00	0.00	C
ATOM	250	CB	VAL	A	162	-11.045	-1.140	-8.558	1.00	0.00	C
ATOM	251	CG1	VAL	A	162	-12.521	-1.194	-7.988	1.00	0.00	C
ATOM	252	CG2	VAL	A	162	-10.014	-0.952	-7.428	1.00	0.00	C
ATOM	253	C	VAL	A	162	-9.498	-2.465	-9.978	1.00	0.00	C
ATOM	254	O	VAL	A	162	-9.169	-1.678	-10.879	1.00	0.00	O
ATOM	255	H	VAL	A	162	-10.765	-3.420	-7.556	1.00	0.00	H
ATOM	256	HA	VAL	A	162	-11.456	-2.517	-10.202	1.00	0.00	H
ATOM	257	HB	VAL	A	162	-10.923	-0.283	-9.261	1.00	0.00	H
ATOM	258	HG11	VAL	A	162	-12.914	-0.152	-7.968	1.00	0.00	H

ATOM	259	HG12	VAL	A	162	-12.624	-1.637	-6.975	1.00	0.00	H
ATOM	260	HG13	VAL	A	162	-13.096	-1.858	-8.676	1.00	0.00	H
ATOM	261	HG21	VAL	A	162	-10.301	-0.035	-6.864	1.00	0.00	H
ATOM	262	HG22	VAL	A	162	-8.997	-0.818	-7.850	1.00	0.00	H
ATOM	263	HG23	VAL	A	162	-10.056	-1.808	-6.738	1.00	0.00	H
ATOM	264	N	SER	A	163	-8.597	-3.455	-9.544	1.00	0.00	N
ATOM	265	CA	SER	A	163	-7.288	-3.613	-10.144	1.00	0.00	C
ATOM	266	CB	SER	A	163	-6.131	-3.255	-9.114	1.00	0.00	C
ATOM	267	OG	SER	A	163	-4.830	-3.312	-9.691	1.00	0.00	O
ATOM	268	C	SER	A	163	-7.117	-5.042	-10.618	1.00	0.00	C
ATOM	269	O	SER	A	163	-7.146	-5.243	-11.853	1.00	0.00	O
ATOM	270	H	SER	A	163	-8.851	-3.997	-8.739	1.00	0.00	H
ATOM	271	HA	SER	A	163	-7.245	-2.955	-10.989	1.00	0.00	H
ATOM	272	HB2	SER	A	163	-6.217	-4.009	-8.300	1.00	0.00	H
ATOM	273	HB3	SER	A	163	-6.383	-2.238	-8.738	1.00	0.00	H
ATOM	274	HG	SER	A	163	-4.665	-2.399	-9.963	1.00	0.00	H
ATOM	275	N	GLY	A	164	-7.006	-6.041	-9.707	1.00	0.00	N
ATOM	276	CA	GLY	A	164	-6.780	-7.426	-10.181	1.00	0.00	C
ATOM	277	C	GLY	A	164	-7.945	-8.009	-10.997	1.00	0.00	C
ATOM	278	O	GLY	A	164	-7.728	-8.799	-11.925	1.00	0.00	O
ATOM	279	H	GLY	A	164	-7.076	-5.843	-8.719	1.00	0.00	H
ATOM	280	HA2	GLY	A	164	-5.921	-7.392	-10.851	1.00	0.00	H
ATOM	281	HA3	GLY	A	164	-6.603	-8.070	-9.306	1.00	0.00	H
ATOM	282	HC	GLY	A	164	-8.960	-7.727	-10.759	1.00	0.00	H
ATOM	283	N	GLY	A	216	3.334	-8.841	-8.526	1.00	0.00	N
ATOM	284	CA	GLY	A	216	2.975	-7.814	-7.536	1.00	0.00	C
ATOM	285	C	GLY	A	216	1.724	-8.170	-6.771	1.00	0.00	C
ATOM	286	O	GLY	A	216	1.719	-8.075	-5.587	1.00	0.00	O
ATOM	287	H1	GLY	A	216	4.207	-9.211	-8.209	1.00	0.00	H

ATOM	288	H2	GLY	A	216	3.238	-8.390	-9.413	1.00	0.00	H
ATOM	289	HA2	GLY	A	216	3.787	-7.740	-6.812	1.00	0.00	H
ATOM	290	HA3	GLY	A	216	2.825	-6.862	-8.060	1.00	0.00	H
ATOM	291	N	TRP	A	217	0.619	-8.552	-7.413	1.00	0.00	N
ATOM	292	CA	TRP	A	217	-0.556	-8.995	-6.764	1.00	0.00	C
ATOM	293	CB	TRP	A	217	-1.340	-7.903	-6.037	1.00	0.00	C
ATOM	294	CG	TRP	A	217	-1.697	-6.696	-6.874	1.00	0.00	C
ATOM	295	CD1	TRP	A	217	-2.734	-6.588	-7.727	1.00	0.00	C
ATOM	296	NE1	TRP	A	217	-2.782	-5.342	-8.253	1.00	0.00	N
ATOM	297	CE2	TRP	A	217	-1.669	-4.634	-7.853	1.00	0.00	C
ATOM	298	CD2	TRP	A	217	-0.944	-5.462	-6.968	1.00	0.00	C
ATOM	299	CE3	TRP	A	217	0.213	-4.985	-6.305	1.00	0.00	C
ATOM	300	CZ3	TRP	A	217	0.624	-3.736	-6.667	1.00	0.00	C
ATOM	301	CZ2	TRP	A	217	-1.323	-3.369	-8.132	1.00	0.00	C
ATOM	302	CH2	TRP	A	217	-0.045	-2.955	-7.545	1.00	0.00	C
ATOM	303	C	TRP	A	217	-0.468	-10.192	-5.877	1.00	0.00	C
ATOM	304	O	TRP	A	217	-1.026	-10.222	-4.827	1.00	0.00	O
ATOM	305	H	TRP	A	217	0.540	-8.471	-8.409	1.00	0.00	H
ATOM	306	HA	TRP	A	217	-1.188	-9.225	-7.608	1.00	0.00	H
ATOM	307	HB2	TRP	A	217	-2.322	-8.358	-5.845	1.00	0.00	H
ATOM	308	HB3	TRP	A	217	-0.779	-7.514	-5.156	1.00	0.00	H
ATOM	309	HD1	TRP	A	217	-3.380	-7.447	-7.837	1.00	0.00	H
ATOM	310	HE1	TRP	A	217	-3.336	-4.941	-8.954	1.00	0.00	H
ATOM	311	HE3	TRP	A	217	0.882	-5.579	-5.703	1.00	0.00	H
ATOM	312	HZ2	TRP	A	217	-1.922	-2.692	-8.724	1.00	0.00	H
ATOM	313	HZ3	TRP	A	217	1.491	-3.374	-6.147	1.00	0.00	H
ATOM	314	HH2	TRP	A	217	0.266	-1.977	-7.880	1.00	0.00	H
ATOM	315	N	GLY	A	218	0.293	-11.205	-6.390	1.00	0.00	N
ATOM	316	CA	GLY	A	218	0.329	-12.539	-5.916	1.00	0.00	C

ATOM	317	C	GLY	A	218	1.356	-12.972	-4.881	1.00	0.00	C
ATOM	318	O	GLY	A	218	1.557	-14.188	-4.680	1.00	0.00	O
ATOM	319	H	GLY	A	218	0.883	-10.982	-7.179	1.00	0.00	H
ATOM	320	HA2	GLY	A	218	-0.609	-12.592	-5.363	1.00	0.00	H
ATOM	321	HA3	GLY	A	218	0.433	-13.240	-6.808	1.00	0.00	H
ATOM	322	N	GLY	A	219	2.205	-12.242	-4.081	1.00	0.00	N
ATOM	323	CA	GLY	A	219	2.370	-10.834	-3.970	1.00	0.00	C
ATOM	324	C	GLY	A	219	1.402	-10.301	-2.962	1.00	0.00	C
ATOM	325	O	GLY	A	219	0.907	-11.039	-2.083	1.00	0.00	O
ATOM	326	H	GLY	A	219	2.803	-12.791	-3.480	1.00	0.00	H
ATOM	327	HA2	GLY	A	219	3.390	-10.615	-3.655	1.00	0.00	H
ATOM	328	HA3	GLY	A	219	2.182	-10.361	-4.936	1.00	0.00	H
ATOM	329	N	PHE	A	220	1.099	-8.988	-2.947	1.00	0.00	N
ATOM	330	CA	PHE	A	220	0.398	-8.347	-1.855	1.00	0.00	C
ATOM	331	CB	PHE	A	220	0.318	-6.809	-2.223	1.00	0.00	C
ATOM	332	CG	PHE	A	220	1.660	-6.185	-1.922	1.00	0.00	C
ATOM	333	CD1	PHE	A	220	2.127	-5.763	-0.696	1.00	0.00	C
ATOM	334	CE1	PHE	A	220	3.339	-5.070	-0.570	1.00	0.00	C
ATOM	335	CZ	PHE	A	220	4.050	-4.574	-1.673	1.00	0.00	C
ATOM	336	CD2	PHE	A	220	2.403	-5.743	-3.058	1.00	0.00	C
ATOM	337	CE2	PHE	A	220	3.543	-5.002	-2.868	1.00	0.00	C
ATOM	338	C	PHE	A	220	-0.928	-8.841	-1.312	1.00	0.00	C
ATOM	339	O	PHE	A	220	-1.242	-8.720	-0.116	1.00	0.00	O
ATOM	340	H	PHE	A	220	1.501	-8.484	-3.698	1.00	0.00	H
ATOM	341	HA	PHE	A	220	1.057	-8.572	-1.043	1.00	0.00	H
ATOM	342	HB2	PHE	A	220	-0.405	-6.195	-1.641	1.00	0.00	H
ATOM	343	HB3	PHE	A	220	0.113	-6.661	-3.303	1.00	0.00	H
ATOM	344	HD1	PHE	A	220	1.484	-6.019	0.130	1.00	0.00	H
ATOM	345	HD2	PHE	A	220	2.086	-6.088	-4.027	1.00	0.00	H

ATOM	346	HE1	PHE	A	220	3.852	-4.949	0.376	1.00	0.00	H
ATOM	347	HE2	PHE	A	220	4.057	-4.699	-3.778	1.00	0.00	H
ATOM	348	HZ	PHE	A	220	5.037	-4.177	-1.547	1.00	0.00	H
ATOM	349	N	PHE	A	221	-1.681	-9.590	-2.196	1.00	0.00	N
ATOM	350	CA	PHE	A	221	-2.865	-10.345	-1.924	1.00	0.00	C
ATOM	351	CB	PHE	A	221	-4.126	-9.481	-1.658	1.00	0.00	C
ATOM	352	CG	PHE	A	221	-4.247	-8.357	-2.666	1.00	0.00	C
ATOM	353	CD1	PHE	A	221	-3.991	-7.036	-2.280	1.00	0.00	C
ATOM	354	CE1	PHE	A	221	-4.348	-6.068	-3.223	1.00	0.00	C
ATOM	355	CZ	PHE	A	221	-5.032	-6.386	-4.379	1.00	0.00	C
ATOM	356	CD2	PHE	A	221	-4.852	-8.754	-3.886	1.00	0.00	C
ATOM	357	CE2	PHE	A	221	-5.255	-7.692	-4.715	1.00	0.00	C
ATOM	358	C	PHE	A	221	-2.705	-11.415	-0.837	1.00	0.00	C
ATOM	359	O	PHE	A	221	-3.544	-11.640	0.047	1.00	0.00	O
ATOM	360	H	PHE	A	221	-1.235	-9.531	-3.091	1.00	0.00	H
ATOM	361	HA	PHE	A	221	-3.025	-10.809	-2.882	1.00	0.00	H
ATOM	362	HB2	PHE	A	221	-5.052	-10.100	-1.622	1.00	0.00	H
ATOM	363	HB3	PHE	A	221	-4.014	-9.002	-0.659	1.00	0.00	H
ATOM	364	HD1	PHE	A	221	-3.593	-6.690	-1.344	1.00	0.00	H
ATOM	365	HD2	PHE	A	221	-4.972	-9.791	-4.179	1.00	0.00	H
ATOM	366	HE1	PHE	A	221	-4.133	-5.029	-3.063	1.00	0.00	H
ATOM	367	HE2	PHE	A	221	-5.862	-7.875	-5.580	1.00	0.00	H
ATOM	368	HZ	PHE	A	221	-5.340	-5.591	-5.058	1.00	0.00	H
ATOM	369	N	GLY	A	222	-1.589	-12.116	-0.904	1.00	0.00	N
ATOM	370	CA	GLY	A	222	-1.105	-13.119	0.025	1.00	0.00	C
ATOM	371	C	GLY	A	222	-0.798	-12.518	1.291	1.00	0.00	C
ATOM	372	O	GLY	A	222	-1.119	-13.038	2.369	1.00	0.00	O
ATOM	373	H	GLY	A	222	-0.998	-11.931	-1.702	1.00	0.00	H
ATOM	374	HA2	GLY	A	222	-1.870	-13.875	0.201	1.00	0.00	H

ATOM	375	HA3	GLY	A	222	-0.215	-13.591	-0.415	1.00	0.00	H
ATOM	376	N	GLY	A	223	-0.059	-11.391	1.154	1.00	0.00	N
ATOM	377	CA	GLY	A	223	0.271	-10.559	2.316	1.00	0.00	C
ATOM	378	C	GLY	A	223	-0.941	-10.144	3.099	1.00	0.00	C
ATOM	379	O	GLY	A	223	-0.901	-10.196	4.328	1.00	0.00	O
ATOM	380	H	GLY	A	223	0.270	-11.120	0.238	1.00	0.00	H
ATOM	381	HA2	GLY	A	223	0.892	-11.152	2.987	1.00	0.00	H
ATOM	382	HA3	GLY	A	223	0.805	-9.664	1.963	1.00	0.00	H
ATOM	383	N	HIS	A	224	-2.031	-9.752	2.396	1.00	0.00	N
ATOM	384	CA	HIS	A	224	-3.306	-9.354	2.891	1.00	0.00	C
ATOM	385	CB	HIS	A	224	-3.222	-8.012	3.714	1.00	0.00	C
ATOM	386	ND1	HIS	A	224	-3.770	-5.676	2.933	1.00	0.00	N
ATOM	387	CG	HIS	A	224	-3.009	-6.818	2.797	1.00	0.00	C
ATOM	388	CE1	HIS	A	224	-3.125	-4.738	2.214	1.00	0.00	C
ATOM	389	NE2	HIS	A	224	-2.111	-5.232	1.496	1.00	0.00	N
ATOM	390	CD2	HIS	A	224	-1.990	-6.536	1.998	1.00	0.00	C
ATOM	391	C	HIS	A	224	-3.883	-10.465	3.783	1.00	0.00	C
ATOM	392	O	HIS	A	224	-4.375	-10.201	4.898	1.00	0.00	O
ATOM	393	H	HIS	A	224	-1.966	-9.657	1.406	1.00	0.00	H
ATOM	394	HA	HIS	A	224	-4.026	-9.143	2.111	1.00	0.00	H
ATOM	395	HB2	HIS	A	224	-2.449	-8.028	4.498	1.00	0.00	H
ATOM	396	HB3	HIS	A	224	-4.222	-7.826	4.164	1.00	0.00	H
ATOM	397	HD1	HIS	A	224	-4.586	-5.503	3.466	1.00	0.00	H
ATOM	398	HD2	HIS	A	224	-1.156	-7.159	1.703	1.00	0.00	H
ATOM	399	HE1	HIS	A	224	-3.487	-3.716	2.063	1.00	0.00	H
ATOM	400	N	GLY	A	225	-3.818	-11.750	3.267	1.00	0.00	N
ATOM	401	CA	GLY	A	225	-4.357	-12.821	3.988	1.00	0.00	C
ATOM	402	C	GLY	A	225	-3.609	-13.068	5.337	1.00	0.00	C
ATOM	403	O	GLY	A	225	-4.246	-13.249	6.386	1.00	0.00	O

ATOM	404	H	GLY	A	225	-3.382	-11.913	2.371	1.00	0.00	H
ATOM	405	HA2	GLY	A	225	-5.393	-12.548	4.187	1.00	0.00	H
ATOM	406	HA3	GLY	A	225	-4.298	-13.756	3.410	1.00	0.00	H
ATOM	407	HC	GLY	A	225	-2.529	-13.081	5.357	1.00	0.00	H
ATOM	408	N	GLY	A	233	4.110	-8.251	14.074	1.00	0.00	N
ATOM	409	CA	GLY	A	233	5.166	-8.766	13.193	1.00	0.00	C
ATOM	410	C	GLY	A	233	4.989	-8.331	11.729	1.00	0.00	C
ATOM	411	O	GLY	A	233	4.938	-9.090	10.801	1.00	0.00	O
ATOM	412	H1	GLY	A	233	4.578	-7.628	14.692	1.00	0.00	H
ATOM	413	H2	GLY	A	233	3.621	-9.060	14.382	1.00	0.00	H
ATOM	414	HA2	GLY	A	233	6.111	-8.353	13.546	1.00	0.00	H
ATOM	415	HA3	GLY	A	233	5.164	-9.862	13.237	1.00	0.00	H
ATOM	416	N	ILE	A	234	4.871	-6.982	11.448	1.00	0.00	N
ATOM	417	CA	ILE	A	234	4.781	-6.288	10.158	1.00	0.00	C
ATOM	418	CB	ILE	A	234	3.743	-5.115	10.315	1.00	0.00	C
ATOM	419	CG2	ILE	A	234	3.468	-4.348	8.963	1.00	0.00	C
ATOM	420	CG1	ILE	A	234	2.447	-5.425	11.082	1.00	0.00	C
ATOM	421	CD1	ILE	A	234	1.574	-6.364	10.277	1.00	0.00	C
ATOM	422	C	ILE	A	234	6.088	-5.751	9.527	1.00	0.00	C
ATOM	423	O	ILE	A	234	6.616	-4.756	10.085	1.00	0.00	O
ATOM	424	H	ILE	A	234	4.982	-6.357	12.222	1.00	0.00	H
ATOM	425	HA	ILE	A	234	4.391	-7.003	9.453	1.00	0.00	H
ATOM	426	HB	ILE	A	234	4.165	-4.405	11.069	1.00	0.00	H
ATOM	427	HG12	ILE	A	234	1.938	-4.442	11.156	1.00	0.00	H
ATOM	428	HG13	ILE	A	234	2.638	-5.772	12.118	1.00	0.00	H
ATOM	429	HG21	ILE	A	234	4.349	-3.817	8.546	1.00	0.00	H
ATOM	430	HG22	ILE	A	234	2.685	-3.577	9.100	1.00	0.00	H
ATOM	431	HG23	ILE	A	234	3.075	-5.072	8.227	1.00	0.00	H
ATOM	432	HD11	ILE	A	234	1.316	-6.000	9.265	1.00	0.00	H

ATOM	433	HD12	ILE	A	234	0.751	-6.726	10.907	1.00	0.00	H
ATOM	434	HD13	ILE	A	234	2.275	-7.216	10.091	1.00	0.00	H
ATOM	435	N	GLY	A	235	6.658	-6.247	8.493	1.00	0.00	N
ATOM	436	CA	GLY	A	235	7.535	-5.502	7.591	1.00	0.00	C
ATOM	437	C	GLY	A	235	8.795	-4.863	8.075	1.00	0.00	C
ATOM	438	O	GLY	A	235	8.890	-3.643	7.953	1.00	0.00	O
ATOM	439	H	GLY	A	235	6.490	-7.221	8.286	1.00	0.00	H
ATOM	440	HA2	GLY	A	235	7.938	-6.291	6.956	1.00	0.00	H
ATOM	441	HA3	GLY	A	235	6.896	-4.703	7.086	1.00	0.00	H
ATOM	442	HC	GLY	A	235	9.590	-5.448	8.514	1.00	0.00	H
ATOM	443	N	GLY	A	238	11.005	-6.441	4.164	1.00	0.00	N
ATOM	444	CA	GLY	A	238	10.178	-7.078	3.125	1.00	0.00	C
ATOM	445	C	GLY	A	238	9.203	-6.183	2.331	1.00	0.00	C
ATOM	446	O	GLY	A	238	9.140	-6.170	1.152	1.00	0.00	O
ATOM	447	H1	GLY	A	238	11.915	-6.491	3.819	1.00	0.00	H
ATOM	448	H2	GLY	A	238	10.665	-6.801	5.004	1.00	0.00	H
ATOM	449	HA2	GLY	A	238	10.900	-7.414	2.381	1.00	0.00	H
ATOM	450	HA3	GLY	A	238	9.587	-7.898	3.601	1.00	0.00	H
ATOM	451	N	TYR	A	239	8.451	-5.315	2.980	1.00	0.00	N
ATOM	452	CA	TYR	A	239	7.522	-4.269	2.429	1.00	0.00	C
ATOM	453	CB	TYR	A	239	6.186	-4.778	1.911	1.00	0.00	C
ATOM	454	CG	TYR	A	239	5.380	-5.437	3.113	1.00	0.00	C
ATOM	455	CD1	TYR	A	239	4.808	-4.575	4.026	1.00	0.00	C
ATOM	456	CE1	TYR	A	239	3.874	-4.963	5.029	1.00	0.00	C
ATOM	457	CZ	TYR	A	239	3.671	-6.342	5.151	1.00	0.00	C
ATOM	458	OH	TYR	A	239	2.970	-6.850	6.283	1.00	0.00	O
ATOM	459	CD2	TYR	A	239	5.081	-6.823	3.147	1.00	0.00	C
ATOM	460	CE2	TYR	A	239	4.311	-7.232	4.255	1.00	0.00	C
ATOM	461	C	TYR	A	239	8.192	-3.357	1.379	1.00	0.00	C

ATOM	462	O	TYR	A	239	7.817	-3.258	0.213	1.00	0.00	O
ATOM	463	H	TYR	A	239	8.551	-5.379	3.976	1.00	0.00	H
ATOM	464	HA	TYR	A	239	7.323	-3.661	3.302	1.00	0.00	H
ATOM	465	HB2	TYR	A	239	6.424	-5.647	1.240	1.00	0.00	H
ATOM	466	HB3	TYR	A	239	5.606	-3.966	1.432	1.00	0.00	H
ATOM	467	HD1	TYR	A	239	5.149	-3.553	3.841	1.00	0.00	H
ATOM	468	HD2	TYR	A	239	5.472	-7.476	2.396	1.00	0.00	H
ATOM	469	HE1	TYR	A	239	3.449	-4.234	5.694	1.00	0.00	H
ATOM	470	HE2	TYR	A	239	4.067	-8.274	4.426	1.00	0.00	H
ATOM	471	HH	TYR	A	239	2.957	-7.813	6.198	1.00	0.00	H
ATOM	472	N	GLY	A	240	9.266	-2.724	1.768	1.00	0.00	N
ATOM	473	CA	GLY	A	240	10.082	-1.913	0.900	1.00	0.00	C
ATOM	474	C	GLY	A	240	10.765	-2.590	-0.292	1.00	0.00	C
ATOM	475	O	GLY	A	240	10.735	-2.197	-1.492	1.00	0.00	O
ATOM	476	H	GLY	A	240	9.542	-2.808	2.736	1.00	0.00	H
ATOM	477	HA2	GLY	A	240	9.364	-1.229	0.447	1.00	0.00	H
ATOM	478	HA3	GLY	A	240	10.880	-1.415	1.515	1.00	0.00	H
ATOM	479	HC	GLY	A	240	11.326	-3.490	-0.087	1.00	0.00	H
TER	480		GLY	A	240						
HETATM	481	C1	HAL	B	274	0.439	-1.293	1.320	1.00	0.00	C
HETATM	482	C2	HAL	B	274	1.053	-1.828	-0.006	1.00	0.00	C
HETATM	483	N	HAL	B	274	0.037	-1.989	-1.076	1.00	0.00	N1+
HETATM	484	C3	HAL	B	274	-2.340	-2.599	-1.641	1.00	0.00	C
HETATM	485	C4	HAL	B	274	-1.905	-2.707	-3.140	1.00	0.00	C
HETATM	486	C5	HAL	B	274	-1.105	-2.912	-0.771	1.00	0.00	C
HETATM	487	C6	HAL	B	274	-0.629	-1.921	-3.454	1.00	0.00	C
HETATM	488	C7	HAL	B	274	0.506	-2.184	-2.469	1.00	0.00	C
HETATM	489	O1	HAL	B	274	-1.505	-4.078	-3.330	1.00	0.00	O
HETATM	490	C8	HAL	B	274	-3.009	-2.277	-4.071	1.00	0.00	C

HETATM	491	C9	HAL	B	274	-3.483	-3.074	-5.147	1.00	0.00	C
HETATM	492	C10	HAL	B	274	-3.670	-1.019	-3.776	1.00	0.00	C
HETATM	493	C11	HAL	B	274	-4.589	-2.639	-5.878	1.00	0.00	C
HETATM	494	C12	HAL	B	274	-4.735	-0.636	-4.600	1.00	0.00	C
HETATM	495	C13	HAL	B	274	-5.161	-1.369	-5.665	1.00	0.00	C
HETATM	496	Cl	HAL	B	274	-6.442	-0.797	-6.667	1.00	0.00	Cl
HETATM	497	C14	HAL	B	274	1.631	-1.060	2.326	1.00	0.00	C
HETATM	498	C15	HAL	B	274	1.194	-0.667	3.692	1.00	0.00	C
HETATM	499	O2	HAL	B	274	0.074	-0.199	3.833	1.00	0.00	O
HETATM	500	C16	HAL	B	274	2.095	-0.747	4.870	1.00	0.00	C
HETATM	501	C17	HAL	B	274	3.451	-0.820	4.731	1.00	0.00	C
HETATM	502	C18	HAL	B	274	1.463	-0.600	6.097	1.00	0.00	C
HETATM	503	C19	HAL	B	274	4.274	-0.880	5.903	1.00	0.00	C
HETATM	504	C20	HAL	B	274	2.266	-0.760	7.326	1.00	0.00	C
HETATM	505	C21	HAL	B	274	3.694	-0.901	7.143	1.00	0.00	C
HETATM	506	F	HAL	B	274	4.409	-0.987	8.217	1.00	0.00	F
HETATM	507	H	HAL	B	274	-1.218	-4.171	-4.243	1.00	0.00	H
HETATM	508	HC2	HAL	B	274	-0.300	-1.935	1.859	1.00	0.00	H
HETATM	509	HC3	HAL	B	274	-0.123	-0.350	1.194	1.00	0.00	H
HETATM	510	HC4	HAL	B	274	1.458	-2.836	0.194	1.00	0.00	H
HETATM	511	HC5	HAL	B	274	1.870	-1.180	-0.328	1.00	0.00	H
HETATM	512	HN	HAL	B	274	-0.336	-1.009	-1.128	1.00	0.00	H
HETATM	513	HC6	HAL	B	274	-3.141	-3.290	-1.281	1.00	0.00	H
HETATM	514	HC7	HAL	B	274	-2.656	-1.560	-1.423	1.00	0.00	H
HETATM	515	HC8	HAL	B	274	-1.384	-2.792	0.283	1.00	0.00	H
HETATM	516	HC9	HAL	B	274	-0.648	-3.887	-0.932	1.00	0.00	H
HETATM	517	HC10	HAL	B	274	-0.898	-0.842	-3.456	1.00	0.00	H
HETATM	518	HC11	HAL	B	274	-0.337	-2.203	-4.482	1.00	0.00	H
HETATM	519	HC12	HAL	B	274	0.877	-3.221	-2.569	1.00	0.00	H

HETATM	520	HC13	HAL	B	274	1.334	-1.490	-2.650	1.00	0.00	H
HETATM	521	HC	HAL	B	274	-2.982	-4.004	-5.328	1.00	0.00	H
HETATM	522	HC1	HAL	B	274	-3.198	-0.395	-3.035	1.00	0.00	H
HETATM	523	HC14	HAL	B	274	-4.856	-3.295	-6.687	1.00	0.00	H
HETATM	524	HC15	HAL	B	274	-5.289	0.280	-4.456	1.00	0.00	H
HETATM	525	HC16	HAL	B	274	2.388	-0.314	1.979	1.00	0.00	H
HETATM	526	HC17	HAL	B	274	2.174	-2.003	2.493	1.00	0.00	H
HETATM	527	HC18	HAL	B	274	3.921	-0.792	3.755	1.00	0.00	H
HETATM	528	HC19	HAL	B	274	0.389	-0.589	6.175	1.00	0.00	H
HETATM	529	HC20	HAL	B	274	5.340	-0.870	5.826	1.00	0.00	H
HETATM	530	HC21	HAL	B	274	1.865	-0.897	8.318	1.00	0.00	H
HETATM	531	OH2	TIP	X6377		-0.639	0.754	-1.422	1.00	0.00	O
HETATM	532	H1	TIP	X6377		-0.449	1.114	-2.290	1.00	0.00	H
HETATM	533	H2	TIP	X6377		-0.905	1.508	-0.893	1.00	0.00	H
HETATM	534	OH2	TIP	X8206		7.275	0.646	8.211	1.00	0.00	O
HETATM	535	H1	TIP	X8206		7.875	1.376	8.019	1.00	0.00	H
HETATM	536	H2	TIP	X8206		6.531	1.045	8.637	1.00	0.00	H
HETATM	537	OH2	TIP	X8259		3.736	2.273	6.374	1.00	0.00	O
HETATM	538	H1	TIP	X8259		3.924	2.970	5.755	1.00	0.00	H
HETATM	539	H2	TIP	X8259		3.336	1.585	5.849	1.00	0.00	H
HETATM	540	OH2	TIP	X	419	3.850	3.297	3.604	1.00	0.00	O
HETATM	541	H1	TIP	X	419	4.283	4.141	3.711	1.00	0.00	H
HETATM	542	H2	TIP	X	419	3.039	3.502	3.107	1.00	0.00	H
HETATM	543	OH2	TIP	X5829		1.716	0.042	-5.205	1.00	0.00	O
HETATM	544	H1	TIP	X5829		1.017	-0.482	-5.595	1.00	0.00	H
HETATM	545	H2	TIP	X5829		1.307	0.891	-5.009	1.00	0.00	H
HETATM	546	OH2	TIP	X5939		7.152	2.981	4.793	1.00	0.00	O
HETATM	547	H1	TIP	X5939		7.385	2.061	4.670	1.00	0.00	H
HETATM	548	H2	TIP	X5939		7.504	3.411	4.011	1.00	0.00	H

HETATM	549	OH2	TIP	X9200	4.721	0.984	1.672	1.00	0.00	O
HETATM	550	H1	TIP	X9200	4.358	1.336	0.863	1.00	0.00	H
HETATM	551	H2	TIP	X9200	4.488	1.631	2.337	1.00	0.00	H
HETATM	552	OH2	TIP	X9662	4.248	-1.222	0.099	1.00	0.00	O
HETATM	553	H1	TIP	X9662	4.916	-0.983	-0.558	1.00	0.00	H
HETATM	554	H2	TIP	X9662	3.984	-0.405	0.490	1.00	0.00	H
HETATM	555	OH2	TIP	X 730	5.868	-1.336	2.262	1.00	0.00	O
HETATM	556	H1	TIP	X 730	5.764	-0.387	2.230	1.00	0.00	H
HETATM	557	H2	TIP	X 730	5.400	-1.648	1.490	1.00	0.00	H
HETATM	558	OH2	TIP	X3644	-0.160	-4.761	5.084	1.00	0.00	O
HETATM	559	H1	TIP	X3644	-0.610	-5.435	5.603	1.00	0.00	H
HETATM	560	H2	TIP	X3644	-0.474	-3.927	5.437	1.00	0.00	H
HETATM	561	OH2	TIP	X6744	3.486	0.925	10.801	1.00	0.00	O
HETATM	562	H1	TIP	X6744	3.829	0.040	10.817	1.00	0.00	H
HETATM	563	H2	TIP	X6744	3.174	1.072	11.697	1.00	0.00	H
HETATM	564	OH2	TIP	X7837	-0.322	2.466	4.217	1.00	0.00	O
HETATM	565	H1	TIP	X7837	-0.001	1.563	4.232	1.00	0.00	H
HETATM	566	H2	TIP	X7837	-0.578	2.657	5.122	1.00	0.00	H
HETATM	567	OH2	TIP	X 983	7.186	-1.823	6.938	1.00	0.00	O
HETATM	568	H1	TIP	X 983	6.939	-1.082	7.487	1.00	0.00	H
HETATM	569	H2	TIP	X 983	7.277	-2.552	7.547	1.00	0.00	H
HETATM	570	OH2	TIP	X3979	-9.132	-0.076	-2.441	1.00	0.00	O
HETATM	571	H1	TIP	X3979	-9.735	-0.364	-3.118	1.00	0.00	H
HETATM	572	H2	TIP	X3979	-8.329	-0.578	-2.592	1.00	0.00	H
HETATM	573	OH2	TIP	X6055	7.526	-2.540	11.182	1.00	0.00	O
HETATM	574	H1	TIP	X6055	7.941	-2.947	11.948	1.00	0.00	H
HETATM	575	H2	TIP	X6055	7.389	-3.276	10.587	1.00	0.00	H
HETATM	576	OH2	TIP	X7729	0.623	-4.494	2.582	1.00	0.00	O
HETATM	577	H1	TIP	X7729	-0.231	-4.833	2.332	1.00	0.00	H

```
HETATM  578  H2  TIP X7729       0.492  -4.064   3.428  1.00  0.00           H
HETATM  579  OH2 TIP X8254       1.463   3.558   2.002  1.00  0.00           O
HETATM  580  H1  TIP X8254       0.568   3.542   2.352  1.00  0.00           H
HETATM  581  H2  TIP X8254       1.363   3.429   1.071  1.00  0.00           H
HETATM  582  OH2 TIP X2059       7.484   0.185   4.718  1.00  0.00           O
HETATM  583  H1  TIP X2059       7.138  -0.559   4.220  1.00  0.00           H
HETATM  584  H2  TIP X2059       7.532  -0.132   5.619  1.00  0.00           H
HETATM  585  OH2 TIP X4692       3.176   1.839  -0.234  1.00  0.00           O
HETATM  586  H1  TIP X4692       3.438   2.120  -1.107  1.00  0.00           H
HETATM  587  H2  TIP X4692       2.322   2.246  -0.094  1.00  0.00           H
HETATM  588  OH2 TIP X5919       4.812   2.235   8.858  1.00  0.00           O
HETATM  589  H1  TIP X5919       4.395   1.576   9.406  1.00  0.00           H
HETATM  590  H2  TIP X5919       4.391   2.171   8.020  1.00  0.00           H
HETATM  591  OH2 TIP X9531       4.898  -1.436  11.872  1.00  0.00           O
HETATM  592  H1  TIP X9531       4.426  -1.761  12.641  1.00  0.00           H
HETATM  593  H2  TIP X9531       5.745  -1.877  11.911  1.00  0.00           H
END
```

Haloperidol (Cluster structure 2)

```
ATOM      1  N   GLY A  56       5.680  11.089  -1.234  1.00  0.00           N
ATOM      2  CA  GLY A  56       5.191  10.557   0.046  1.00  0.00           C
ATOM      3  C   GLY A  56       6.092   9.513   0.673  1.00  0.00           C
ATOM      4  O   GLY A  56       6.275   9.378   1.885  1.00  0.00           O
ATOM      5  H1  GLY A  56       5.886  12.042  -1.054  1.00  0.00           H
ATOM      6  H2  GLY A  56       5.021  10.783  -1.908  1.00  0.00           H
ATOM      7  HA2 GLY A  56       5.166  11.393   0.745  1.00  0.00           H
ATOM      8  HA3 GLY A  56       4.190  10.119  -0.118  1.00  0.00           H
ATOM      9  N   VAL A  57       6.809   8.664  -0.076  1.00  0.00           N
ATOM     10  CA  VAL A  57       7.618   7.565   0.433  1.00  0.00           C
```

ATOM	11	CB	VAL	A	57	7.663	6.342	-0.610	1.00	0.00	C
ATOM	12	CG1	VAL	A	57	8.520	5.201	-0.103	1.00	0.00	C
ATOM	13	CG2	VAL	A	57	6.240	5.906	-0.963	1.00	0.00	C
ATOM	14	C	VAL	A	57	9.084	8.149	0.641	1.00	0.00	C
ATOM	15	O	VAL	A	57	9.838	7.595	1.448	1.00	0.00	O
ATOM	16	H	VAL	A	57	6.954	8.657	-1.055	1.00	0.00	H
ATOM	17	HA	VAL	A	57	7.272	7.122	1.362	1.00	0.00	H
ATOM	18	HB	VAL	A	57	8.137	6.845	-1.475	1.00	0.00	H
ATOM	19	HG11	VAL	A	57	8.490	4.467	-0.943	1.00	0.00	H
ATOM	20	HG12	VAL	A	57	8.042	4.760	0.798	1.00	0.00	H
ATOM	21	HG13	VAL	A	57	9.561	5.533	0.051	1.00	0.00	H
ATOM	22	HG21	VAL	A	57	5.704	6.738	-1.458	1.00	0.00	H
ATOM	23	HG22	VAL	A	57	5.686	5.763	-0.007	1.00	0.00	H
ATOM	24	HG23	VAL	A	57	6.247	5.060	-1.678	1.00	0.00	H
ATOM	25	N	GLY	A	58	9.465	9.183	-0.132	1.00	0.00	N
ATOM	26	CA	GLY	A	58	10.689	9.951	-0.117	1.00	0.00	C
ATOM	27	C	GLY	A	58	10.750	10.756	1.148	1.00	0.00	C
ATOM	28	O	GLY	A	58	11.803	10.784	1.806	1.00	0.00	O
ATOM	29	H	GLY	A	58	8.792	9.466	-0.830	1.00	0.00	H
ATOM	30	HA2	GLY	A	58	11.546	9.279	-0.165	1.00	0.00	H
ATOM	31	HA3	GLY	A	58	10.706	10.620	-0.975	1.00	0.00	H
ATOM	32	N	GLY	A	59	9.633	11.391	1.556	1.00	0.00	N
ATOM	33	CA	GLY	A	59	9.386	11.849	2.895	1.00	0.00	C
ATOM	34	C	GLY	A	59	9.425	10.595	3.847	1.00	0.00	C
ATOM	35	O	GLY	A	59	10.357	10.419	4.691	1.00	0.00	O
ATOM	36	H	GLY	A	59	8.914	11.558	0.867	1.00	0.00	H
ATOM	37	HA2	GLY	A	59	10.137	12.592	3.162	1.00	0.00	H
ATOM	38	HA3	GLY	A	59	8.408	12.290	2.975	1.00	0.00	H
ATOM	39	N	LEU	A	60	8.336	9.779	3.914	1.00	0.00	N

ATOM	40	CA	LEU	A	60	8.314	8.491	4.532	1.00	0.00	C
ATOM	41	CB	LEU	A	60	6.847	7.895	4.426	1.00	0.00	C
ATOM	42	CG	LEU	A	60	6.608	6.549	5.118	1.00	0.00	C
ATOM	43	CD1	LEU	A	60	5.119	6.202	4.996	1.00	0.00	C
ATOM	44	CD2	LEU	A	60	7.540	5.431	4.751	1.00	0.00	C
ATOM	45	C	LEU	A	60	8.887	8.360	5.959	1.00	0.00	C
ATOM	46	O	LEU	A	60	9.898	7.742	6.274	1.00	0.00	O
ATOM	47	H	LEU	A	60	7.528	10.056	3.403	1.00	0.00	H
ATOM	48	HA	LEU	A	60	8.823	7.853	3.837	1.00	0.00	H
ATOM	49	HB2	LEU	A	60	6.655	7.749	3.336	1.00	0.00	H
ATOM	50	HB3	LEU	A	60	6.135	8.647	4.819	1.00	0.00	H
ATOM	51	HG	LEU	A	60	6.771	6.784	6.184	1.00	0.00	H
ATOM	52	HD11	LEU	A	60	4.821	5.231	5.435	1.00	0.00	H
ATOM	53	HD12	LEU	A	60	4.967	6.094	3.890	1.00	0.00	H
ATOM	54	HD13	LEU	A	60	4.399	6.928	5.422	1.00	0.00	H
ATOM	55	HD21	LEU	A	60	7.238	4.909	3.820	1.00	0.00	H
ATOM	56	HD22	LEU	A	60	7.634	4.665	5.553	1.00	0.00	H
ATOM	57	HD23	LEU	A	60	8.562	5.818	4.560	1.00	0.00	H
ATOM	58	N	GLY	A	61	8.252	8.905	6.964	1.00	0.00	N
ATOM	59	CA	GLY	A	61	8.678	8.685	8.350	1.00	0.00	C
ATOM	60	C	GLY	A	61	9.860	9.600	8.852	1.00	0.00	C
ATOM	61	O	GLY	A	61	10.758	9.123	9.540	1.00	0.00	O
ATOM	62	H	GLY	A	61	7.450	9.491	6.781	1.00	0.00	H
ATOM	63	HA2	GLY	A	61	7.807	8.942	8.952	1.00	0.00	H
ATOM	64	HA3	GLY	A	61	8.997	7.635	8.471	1.00	0.00	H
ATOM	65	HC	GLY	A	61	9.880	10.648	8.590	1.00	0.00	H
ATOM	66	N	GLY	A	75	-5.158	11.151	2.855	1.00	0.00	N
ATOM	67	CA	GLY	A	75	-4.554	11.559	1.563	1.00	0.00	C
ATOM	68	C	GLY	A	75	-3.528	10.570	1.078	1.00	0.00	C

ATOM	69	O	GLY	A	75	-3.441	10.259	-0.095	1.00	0.00	O
ATOM	70	H1	GLY	A	75	-6.117	10.980	2.642	1.00	0.00	H
ATOM	71	H2	GLY	A	75	-4.869	11.854	3.502	1.00	0.00	H
ATOM	72	HA2	GLY	A	75	-5.340	11.599	0.809	1.00	0.00	H
ATOM	73	HA3	GLY	A	75	-4.087	12.541	1.698	1.00	0.00	H
ATOM	74	N	PHE	A	76	-2.720	10.044	2.019	1.00	0.00	N
ATOM	75	CA	PHE	A	76	-1.802	9.047	1.786	1.00	0.00	C
ATOM	76	CB	PHE	A	76	-0.725	9.603	0.792	1.00	0.00	C
ATOM	77	CG	PHE	A	76	0.612	8.888	0.753	1.00	0.00	C
ATOM	78	CD1	PHE	A	76	1.463	8.925	-0.435	1.00	0.00	C
ATOM	79	CE1	PHE	A	76	2.648	8.241	-0.392	1.00	0.00	C
ATOM	80	CZ	PHE	A	76	3.121	7.652	0.775	1.00	0.00	C
ATOM	81	CD2	PHE	A	76	1.079	8.294	1.850	1.00	0.00	C
ATOM	82	CE2	PHE	A	76	2.317	7.679	1.893	1.00	0.00	C
ATOM	83	C	PHE	A	76	-2.292	7.625	1.369	1.00	0.00	C
ATOM	84	O	PHE	A	76	-1.962	7.076	0.321	1.00	0.00	O
ATOM	85	H	PHE	A	76	-2.806	10.338	2.972	1.00	0.00	H
ATOM	86	HA	PHE	A	76	-1.304	8.881	2.719	1.00	0.00	H
ATOM	87	HB2	PHE	A	76	-0.504	10.627	1.162	1.00	0.00	H
ATOM	88	HB3	PHE	A	76	-1.165	9.649	-0.238	1.00	0.00	H
ATOM	89	HD1	PHE	A	76	1.123	9.451	-1.317	1.00	0.00	H
ATOM	90	HD2	PHE	A	76	0.552	8.370	2.789	1.00	0.00	H
ATOM	91	HE1	PHE	A	76	3.305	8.219	-1.246	1.00	0.00	H
ATOM	92	HE2	PHE	A	76	2.722	7.243	2.793	1.00	0.00	H
ATOM	93	HZ	PHE	A	76	4.118	7.248	0.854	1.00	0.00	H
ATOM	94	N	VAL	A	77	-3.346	7.095	2.046	1.00	0.00	N
ATOM	95	CA	VAL	A	77	-4.119	5.981	1.602	1.00	0.00	C
ATOM	96	CB	VAL	A	77	-5.120	5.572	2.719	1.00	0.00	C
ATOM	97	CG1	VAL	A	77	-6.203	4.575	2.235	1.00	0.00	C

ATOM	98	CG2	VAL	A	77	-4.331	4.739	3.694	1.00	0.00	C
ATOM	99	C	VAL	A	77	-4.870	6.333	0.236	1.00	0.00	C
ATOM	100	O	VAL	A	77	-4.700	5.623	-0.745	1.00	0.00	O
ATOM	101	H	VAL	A	77	-3.663	7.748	2.743	1.00	0.00	H
ATOM	102	HA	VAL	A	77	-3.503	5.117	1.417	1.00	0.00	H
ATOM	103	HB	VAL	A	77	-5.598	6.455	3.202	1.00	0.00	H
ATOM	104	HG11	VAL	A	77	-6.576	3.858	2.974	1.00	0.00	H
ATOM	105	HG12	VAL	A	77	-5.757	3.977	1.391	1.00	0.00	H
ATOM	106	HG13	VAL	A	77	-6.987	5.194	1.748	1.00	0.00	H
ATOM	107	HG21	VAL	A	77	-5.025	4.428	4.494	1.00	0.00	H
ATOM	108	HG22	VAL	A	77	-3.468	5.259	4.152	1.00	0.00	H
ATOM	109	HG23	VAL	A	77	-4.047	3.870	3.056	1.00	0.00	H
ATOM	110	N	GLY	A	78	-5.519	7.509	0.140	1.00	0.00	N
ATOM	111	CA	GLY	A	78	-6.189	7.964	-1.151	1.00	0.00	C
ATOM	112	C	GLY	A	78	-5.486	7.874	-2.487	1.00	0.00	C
ATOM	113	O	GLY	A	78	-5.918	7.328	-3.516	1.00	0.00	O
ATOM	114	H	GLY	A	78	-5.563	8.110	0.951	1.00	0.00	H
ATOM	115	HA2	GLY	A	78	-6.972	7.221	-1.302	1.00	0.00	H
ATOM	116	HA3	GLY	A	78	-6.513	9.036	-0.975	1.00	0.00	H
ATOM	117	N	GLY	A	79	-4.247	8.424	-2.558	1.00	0.00	N
ATOM	118	CA	GLY	A	79	-3.405	8.364	-3.697	1.00	0.00	C
ATOM	119	C	GLY	A	79	-2.862	7.008	-4.042	1.00	0.00	C
ATOM	120	O	GLY	A	79	-2.776	6.629	-5.190	1.00	0.00	O
ATOM	121	H	GLY	A	79	-3.901	8.910	-1.743	1.00	0.00	H
ATOM	122	HA2	GLY	A	79	-4.046	8.646	-4.533	1.00	0.00	H
ATOM	123	HA3	GLY	A	79	-2.543	9.063	-3.553	1.00	0.00	H
ATOM	124	N	ASP	A	80	-2.541	6.278	-2.951	1.00	0.00	N
ATOM	125	CA	ASP	A	80	-2.125	4.895	-3.031	1.00	0.00	C
ATOM	126	CB	ASP	A	80	-0.614	4.833	-3.405	1.00	0.00	C

ATOM	127	CG	ASP	A	80	0.010	3.535	-3.207	1.00	0.00	C
ATOM	128	OD1	ASP	A	80	0.989	3.260	-3.951	1.00	0.00	O
ATOM	129	OD2	ASP	A	80	-0.406	2.728	-2.357	1.00	0.00	O1-
ATOM	130	C	ASP	A	80	-3.098	3.985	-3.783	1.00	0.00	C
ATOM	131	O	ASP	A	80	-2.798	3.272	-4.756	1.00	0.00	O
ATOM	132	H	ASP	A	80	-2.895	6.545	-2.063	1.00	0.00	H
ATOM	133	HA	ASP	A	80	-2.256	4.604	-2.001	1.00	0.00	H
ATOM	134	HB2	ASP	A	80	-0.109	5.607	-2.782	1.00	0.00	H
ATOM	135	HB3	ASP	A	80	-0.487	5.121	-4.474	1.00	0.00	H
ATOM	136	N	VAL	A	81	-4.461	4.206	-3.373	1.00	0.00	N
ATOM	137	CA	VAL	A	81	-5.558	3.595	-4.065	1.00	0.00	C
ATOM	138	CB	VAL	A	81	-6.900	3.852	-3.439	1.00	0.00	C
ATOM	139	CG1	VAL	A	81	-8.140	3.204	-4.135	1.00	0.00	C
ATOM	140	CG2	VAL	A	81	-7.039	3.209	-2.021	1.00	0.00	C
ATOM	141	C	VAL	A	81	-5.644	4.028	-5.558	1.00	0.00	C
ATOM	142	O	VAL	A	81	-5.812	3.263	-6.465	1.00	0.00	O
ATOM	143	H	VAL	A	81	-4.698	4.876	-2.695	1.00	0.00	H
ATOM	144	HA	VAL	A	81	-5.385	2.529	-4.166	1.00	0.00	H
ATOM	145	HB	VAL	A	81	-6.964	4.953	-3.361	1.00	0.00	H
ATOM	146	HG11	VAL	A	81	-8.964	2.919	-3.446	1.00	0.00	H
ATOM	147	HG12	VAL	A	81	-7.797	2.214	-4.508	1.00	0.00	H
ATOM	148	HG13	VAL	A	81	-8.557	3.755	-4.993	1.00	0.00	H
ATOM	149	HG21	VAL	A	81	-7.154	2.095	-2.071	1.00	0.00	H
ATOM	150	HG22	VAL	A	81	-7.910	3.627	-1.469	1.00	0.00	H
ATOM	151	HG23	VAL	A	81	-6.155	3.341	-1.386	1.00	0.00	H
ATOM	152	N	GLY	A	82	-5.502	5.355	-5.737	1.00	0.00	N
ATOM	153	CA	GLY	A	82	-5.683	5.871	-7.097	1.00	0.00	C
ATOM	154	C	GLY	A	82	-4.685	5.385	-8.109	1.00	0.00	C
ATOM	155	O	GLY	A	82	-5.118	4.936	-9.185	1.00	0.00	O

ATOM	156	H	GLY	A	82	-5.280	5.969	-4.966	1.00	0.00	H
ATOM	157	HA2	GLY	A	82	-6.645	5.492	-7.441	1.00	0.00	H
ATOM	158	HA3	GLY	A	82	-5.648	6.981	-7.053	1.00	0.00	H
ATOM	159	N	GLY	A	83	-3.408	5.461	-7.787	1.00	0.00	N
ATOM	160	CA	GLY	A	83	-2.362	4.979	-8.592	1.00	0.00	C
ATOM	161	C	GLY	A	83	-2.319	3.445	-8.969	1.00	0.00	C
ATOM	162	O	GLY	A	83	-2.246	3.025	-10.134	1.00	0.00	O
ATOM	163	H	GLY	A	83	-3.167	5.893	-6.906	1.00	0.00	H
ATOM	164	HA2	GLY	A	83	-2.566	5.501	-9.527	1.00	0.00	H
ATOM	165	HA3	GLY	A	83	-1.366	5.210	-8.114	1.00	0.00	H
ATOM	166	N	CYS	A	84	-2.712	2.639	-7.960	1.00	0.00	N
ATOM	167	CA	CYS	A	84	-2.893	1.212	-8.062	1.00	0.00	C
ATOM	168	CB	CYS	A	84	-2.955	0.584	-6.693	1.00	0.00	C
ATOM	169	SG	CYS	A	84	-1.323	0.483	-5.917	1.00	0.00	S
ATOM	170	C	CYS	A	84	-4.119	0.811	-8.818	1.00	0.00	C
ATOM	171	O	CYS	A	84	-4.048	-0.079	-9.697	1.00	0.00	O
ATOM	172	H	CYS	A	84	-2.748	2.971	-7.024	1.00	0.00	H
ATOM	173	HA	CYS	A	84	-2.062	0.873	-8.647	1.00	0.00	H
ATOM	174	HB2	CYS	A	84	-3.309	-0.473	-6.746	1.00	0.00	H
ATOM	175	HB3	CYS	A	84	-3.682	1.144	-6.053	1.00	0.00	H
ATOM	176	HG	CYS	A	84	-1.210	1.726	-5.483	1.00	0.00	H
ATOM	177	N	THR	A	85	-5.186	1.573	-8.705	1.00	0.00	N
ATOM	178	CA	THR	A	85	-6.444	1.452	-9.479	1.00	0.00	C
ATOM	179	CB	THR	A	85	-7.541	2.306	-8.826	1.00	0.00	C
ATOM	180	OG1	THR	A	85	-7.729	1.880	-7.480	1.00	0.00	O
ATOM	181	CG2	THR	A	85	-8.906	2.016	-9.487	1.00	0.00	C
ATOM	182	C	THR	A	85	-6.178	1.702	-11.001	1.00	0.00	C
ATOM	183	O	THR	A	85	-6.666	0.985	-11.801	1.00	0.00	O
ATOM	184	H	THR	A	85	-5.083	2.329	-8.075	1.00	0.00	H

ATOM	185	HA	THR	A	85	-6.679	0.417	-9.323	1.00	0.00	H
ATOM	186	HB	THR	A	85	-7.306	3.398	-8.886	1.00	0.00	H
ATOM	187	HG21	THR	A	85	-9.136	0.921	-9.599	1.00	0.00	H
ATOM	188	HG22	THR	A	85	-8.941	2.442	-10.511	1.00	0.00	H
ATOM	189	HG23	THR	A	85	-9.732	2.484	-8.912	1.00	0.00	H
ATOM	190	HG1	THR	A	85	-7.035	2.241	-6.935	1.00	0.00	H
ATOM	191	N	GLY	A	86	-5.350	2.771	-11.273	1.00	0.00	N
ATOM	192	CA	GLY	A	86	-5.004	3.210	-12.655	1.00	0.00	C
ATOM	193	C	GLY	A	86	-4.002	2.111	-13.271	1.00	0.00	C
ATOM	194	O	GLY	A	86	-4.116	1.690	-14.388	1.00	0.00	O
ATOM	195	H	GLY	A	86	-4.958	3.283	-10.496	1.00	0.00	H
ATOM	196	HA2	GLY	A	86	-5.912	3.317	-13.248	1.00	0.00	H
ATOM	197	HA3	GLY	A	86	-4.487	4.139	-12.625	1.00	0.00	H
ATOM	198	HC	GLY	A	86	-3.197	1.739	-12.655	1.00	0.00	H
ATOM	199	N	GLY	A	155	-12.463	-1.308	2.802	1.00	0.00	N
ATOM	200	CA	GLY	A	155	-12.398	-0.535	1.624	1.00	0.00	C
ATOM	201	C	GLY	A	155	-11.198	-0.873	0.784	1.00	0.00	C
ATOM	202	O	GLY	A	155	-11.371	-1.029	-0.427	1.00	0.00	O
ATOM	203	H1	GLY	A	155	-13.298	-1.878	2.732	1.00	0.00	H
ATOM	204	H2	GLY	A	155	-12.364	-0.665	3.580	1.00	0.00	H
ATOM	205	HA2	GLY	A	155	-13.294	-0.761	1.046	1.00	0.00	H
ATOM	206	HA3	GLY	A	155	-12.357	0.538	1.881	1.00	0.00	H
ATOM	207	N	VAL	A	156	-10.064	-1.244	1.382	1.00	0.00	N
ATOM	208	CA	VAL	A	156	-9.046	-1.908	0.673	1.00	0.00	C
ATOM	209	CB	VAL	A	156	-7.782	-2.181	1.554	1.00	0.00	C
ATOM	210	CG1	VAL	A	156	-6.546	-2.708	0.755	1.00	0.00	C
ATOM	211	CG2	VAL	A	156	-7.346	-0.861	2.205	1.00	0.00	C
ATOM	212	C	VAL	A	156	-9.430	-3.208	0.036	1.00	0.00	C
ATOM	213	O	VAL	A	156	-8.903	-3.408	-1.085	1.00	0.00	O

```
ATOM    214  H   VAL A 156      -9.901  -1.075   2.362  1.00  0.00           H
ATOM    215  HA  VAL A 156      -8.676  -1.233  -0.084  1.00  0.00           H
ATOM    216  HB  VAL A 156      -8.045  -2.873   2.393  1.00  0.00           H
ATOM    217 HG11 VAL A 156      -6.471  -1.969  -0.060  1.00  0.00           H
ATOM    218 HG12 VAL A 156      -6.588  -3.740   0.360  1.00  0.00           H
ATOM    219 HG13 VAL A 156      -5.671  -2.712   1.451  1.00  0.00           H
ATOM    220 HG21 VAL A 156      -8.102  -0.552   2.956  1.00  0.00           H
ATOM    221 HG22 VAL A 156      -7.246  -0.003   1.510  1.00  0.00           H
ATOM    222 HG23 VAL A 156      -6.386  -1.073   2.697  1.00  0.00           H
ATOM    223  N   GLY A 157     -10.240  -4.130   0.679  1.00  0.00           N
ATOM    224  CA  GLY A 157     -10.711  -5.329   0.154  1.00  0.00           C
ATOM    225  C   GLY A 157     -11.620  -5.213  -1.081  1.00  0.00           C
ATOM    226  O   GLY A 157     -11.361  -5.771  -2.131  1.00  0.00           O
ATOM    227  H   GLY A 157     -10.517  -3.909   1.625  1.00  0.00           H
ATOM    228  HA2 GLY A 157      -9.804  -5.841  -0.166  1.00  0.00           H
ATOM    229  HA3 GLY A 157     -11.283  -5.896   0.931  1.00  0.00           H
ATOM    230  N   GLY A 158     -12.553  -4.238  -1.016  1.00  0.00           N
ATOM    231  CA  GLY A 158     -13.440  -3.794  -2.135  1.00  0.00           C
ATOM    232  C   GLY A 158     -12.628  -3.252  -3.295  1.00  0.00           C
ATOM    233  O   GLY A 158     -12.833  -3.583  -4.460  1.00  0.00           O
ATOM    234  H   GLY A 158     -12.661  -3.765  -0.130  1.00  0.00           H
ATOM    235  HA2 GLY A 158     -14.013  -4.646  -2.501  1.00  0.00           H
ATOM    236  HA3 GLY A 158     -14.104  -3.019  -1.756  1.00  0.00           H
ATOM    237  N   SER A 159     -11.613  -2.375  -2.966  1.00  0.00           N
ATOM    238  CA  SER A 159     -10.782  -1.651  -3.970  1.00  0.00           C
ATOM    239  CB  SER A 159      -9.914  -0.490  -3.256  1.00  0.00           C
ATOM    240  OG  SER A 159     -10.790   0.534  -2.787  1.00  0.00           O
ATOM    241  C   SER A 159      -9.851  -2.620  -4.750  1.00  0.00           C
ATOM    242  O   SER A 159      -9.578  -2.465  -5.933  1.00  0.00           O
```

```
ATOM    243  H   SER A 159     -11.419  -2.070  -2.029  1.00  0.00           H
ATOM    244  HA  SER A 159     -11.484  -1.273  -4.688  1.00  0.00           H
ATOM    245  HB2 SER A 159      -9.247   0.017  -3.997  1.00  0.00           H
ATOM    246  HB3 SER A 159      -9.348  -0.873  -2.384  1.00  0.00           H
ATOM    247  HG  SER A 159     -11.243   0.255  -2.001  1.00  0.00           H
ATOM    248  N   SER A 160      -9.450  -3.735  -4.082  1.00  0.00           N
ATOM    249  CA  SER A 160      -8.763  -4.866  -4.707  1.00  0.00           C
ATOM    250  CB  SER A 160      -8.330  -5.841  -3.619  1.00  0.00           C
ATOM    251  OG  SER A 160      -7.294  -5.308  -2.795  1.00  0.00           O
ATOM    252  C   SER A 160      -9.613  -5.492  -5.783  1.00  0.00           C
ATOM    253  O   SER A 160      -9.248  -5.668  -6.921  1.00  0.00           O
ATOM    254  H   SER A 160      -9.584  -3.738  -3.093  1.00  0.00           H
ATOM    255  HA  SER A 160      -7.821  -4.435  -5.025  1.00  0.00           H
ATOM    256  HB2 SER A 160      -7.956  -6.743  -4.132  1.00  0.00           H
ATOM    257  HB3 SER A 160      -9.150  -6.209  -2.965  1.00  0.00           H
ATOM    258  HG  SER A 160      -7.620  -4.937  -1.977  1.00  0.00           H
ATOM    259  N   GLY A 161     -10.802  -5.865  -5.326  1.00  0.00           N
ATOM    260  CA  GLY A 161     -11.854  -6.695  -5.976  1.00  0.00           C
ATOM    261  C   GLY A 161     -12.318  -6.019  -7.191  1.00  0.00           C
ATOM    262  O   GLY A 161     -12.614  -6.738  -8.200  1.00  0.00           O
ATOM    263  H   GLY A 161     -11.027  -5.537  -4.398  1.00  0.00           H
ATOM    264  HA2 GLY A 161     -11.468  -7.674  -6.260  1.00  0.00           H
ATOM    265  HA3 GLY A 161     -12.666  -6.819  -5.264  1.00  0.00           H
ATOM    266  N   GLY A 162     -12.472  -4.701  -7.201  1.00  0.00           N
ATOM    267  CA  GLY A 162     -12.891  -3.964  -8.374  1.00  0.00           C
ATOM    268  C   GLY A 162     -11.785  -3.403  -9.169  1.00  0.00           C
ATOM    269  O   GLY A 162     -11.989  -2.643 -10.081  1.00  0.00           O
ATOM    270  H   GLY A 162     -12.288  -4.188  -6.350  1.00  0.00           H
ATOM    271  HA2 GLY A 162     -13.393  -4.672  -9.033  1.00  0.00           H
```

ATOM	272	HA3	GLY	A	162	-13.570	-3.145	-8.047	1.00	0.00	H
ATOM	273	N	SER	A	163	-10.556	-3.920	-9.022	1.00	0.00	N
ATOM	274	CA	SER	A	163	-9.469	-3.540	-9.953	1.00	0.00	C
ATOM	275	CB	SER	A	163	-8.874	-2.146	-9.594	1.00	0.00	C
ATOM	276	OG	SER	A	163	-8.471	-2.067	-8.237	1.00	0.00	O
ATOM	277	C	SER	A	163	-8.412	-4.554	-10.087	1.00	0.00	C
ATOM	278	O	SER	A	163	-8.347	-5.079	-11.182	1.00	0.00	O
ATOM	279	H	SER	A	163	-10.328	-4.406	-8.182	1.00	0.00	H
ATOM	280	HA	SER	A	163	-9.909	-3.571	-10.948	1.00	0.00	H
ATOM	281	HB2	SER	A	163	-9.558	-1.283	-9.724	1.00	0.00	H
ATOM	282	HB3	SER	A	163	-7.954	-1.936	-10.175	1.00	0.00	H
ATOM	283	HG	SER	A	163	-9.303	-2.106	-7.746	1.00	0.00	H
ATOM	284	N	PHE	A	164	-7.592	-4.770	-9.018	1.00	0.00	N
ATOM	285	CA	PHE	A	164	-6.522	-5.705	-9.028	1.00	0.00	C
ATOM	286	CB	PHE	A	164	-5.852	-5.581	-7.638	1.00	0.00	C
ATOM	287	CG	PHE	A	164	-4.723	-6.551	-7.419	1.00	0.00	C
ATOM	288	CD1	PHE	A	164	-3.842	-7.001	-8.403	1.00	0.00	C
ATOM	289	CE1	PHE	A	164	-2.852	-7.899	-8.207	1.00	0.00	C
ATOM	290	CZ	PHE	A	164	-2.562	-8.381	-6.890	1.00	0.00	C
ATOM	291	CD2	PHE	A	164	-4.440	-6.932	-6.099	1.00	0.00	C
ATOM	292	CE2	PHE	A	164	-3.389	-7.909	-5.862	1.00	0.00	C
ATOM	293	C	PHE	A	164	-6.992	-7.128	-9.336	1.00	0.00	C
ATOM	294	O	PHE	A	164	-6.390	-7.921	-10.039	1.00	0.00	O
ATOM	295	H	PHE	A	164	-7.681	-4.234	-8.193	1.00	0.00	H
ATOM	296	HA	PHE	A	164	-5.738	-5.581	-9.752	1.00	0.00	H
ATOM	297	HB2	PHE	A	164	-6.543	-5.677	-6.773	1.00	0.00	H
ATOM	298	HB3	PHE	A	164	-5.438	-4.555	-7.568	1.00	0.00	H
ATOM	299	HD1	PHE	A	164	-4.149	-6.849	-9.443	1.00	0.00	H
ATOM	300	HD2	PHE	A	164	-5.092	-6.672	-5.283	1.00	0.00	H

ATOM	301	HE1	PHE	A	164	-2.178	-8.188	-8.992	1.00	0.00	H
ATOM	302	HE2	PHE	A	164	-3.295	-8.308	-4.856	1.00	0.00	H
ATOM	303	HZ	PHE	A	164	-1.705	-9.014	-6.701	1.00	0.00	H
ATOM	304	N	GLY	A	165	-8.215	-7.427	-8.841	1.00	0.00	N
ATOM	305	CA	GLY	A	165	-9.056	-8.530	-9.038	1.00	0.00	C
ATOM	306	C	GLY	A	165	-9.471	-8.735	-10.476	1.00	0.00	C
ATOM	307	O	GLY	A	165	-9.818	-9.888	-10.865	1.00	0.00	O
ATOM	308	H	GLY	A	165	-8.590	-6.728	-8.216	1.00	0.00	H
ATOM	309	HA2	GLY	A	165	-8.473	-9.400	-8.737	1.00	0.00	H
ATOM	310	HA3	GLY	A	165	-9.977	-8.420	-8.425	1.00	0.00	H
ATOM	311	HC	GLY	A	165	-9.472	-7.904	-11.166	1.00	0.00	H
ATOM	312	N	GLY	A	220	4.522	-7.708	-3.262	1.00	0.00	N
ATOM	313	CA	GLY	A	220	4.361	-6.621	-2.339	1.00	0.00	C
ATOM	314	C	GLY	A	220	3.008	-6.813	-1.548	1.00	0.00	C
ATOM	315	O	GLY	A	220	2.903	-6.364	-0.413	1.00	0.00	O
ATOM	316	H1	GLY	A	220	5.355	-8.193	-2.952	1.00	0.00	H
ATOM	317	H2	GLY	A	220	4.508	-7.277	-4.179	1.00	0.00	H
ATOM	318	HA2	GLY	A	220	5.219	-6.615	-1.667	1.00	0.00	H
ATOM	319	HA3	GLY	A	220	4.301	-5.678	-2.862	1.00	0.00	H
ATOM	320	N	PHE	A	221	1.962	-7.533	-2.066	1.00	0.00	N
ATOM	321	CA	PHE	A	221	0.657	-7.775	-1.387	1.00	0.00	C
ATOM	322	CB	PHE	A	221	-0.117	-6.488	-1.117	1.00	0.00	C
ATOM	323	CG	PHE	A	221	-0.343	-5.523	-2.253	1.00	0.00	C
ATOM	324	CD1	PHE	A	221	0.458	-4.339	-2.199	1.00	0.00	C
ATOM	325	CE1	PHE	A	221	0.251	-3.348	-3.128	1.00	0.00	C
ATOM	326	CZ	PHE	A	221	-0.799	-3.447	-4.062	1.00	0.00	C
ATOM	327	CD2	PHE	A	221	-1.273	-5.711	-3.263	1.00	0.00	C
ATOM	328	CE2	PHE	A	221	-1.535	-4.657	-4.160	1.00	0.00	C
ATOM	329	C	PHE	A	221	0.679	-8.652	-0.088	1.00	0.00	C

ATOM	330	O	PHE	A	221	-0.446	-8.965	0.379	1.00	0.00	O
ATOM	331	H	PHE	A	221	1.969	-7.814	-3.013	1.00	0.00	H
ATOM	332	HA	PHE	A	221	0.044	-8.368	-2.054	1.00	0.00	H
ATOM	333	HB2	PHE	A	221	-1.178	-6.710	-0.874	1.00	0.00	H
ATOM	334	HB3	PHE	A	221	0.273	-6.000	-0.201	1.00	0.00	H
ATOM	335	HD1	PHE	A	221	1.192	-4.331	-1.401	1.00	0.00	H
ATOM	336	HD2	PHE	A	221	-1.914	-6.575	-3.122	1.00	0.00	H
ATOM	337	HE1	PHE	A	221	0.864	-2.465	-3.146	1.00	0.00	H
ATOM	338	HE2	PHE	A	221	-2.425	-4.756	-4.767	1.00	0.00	H
ATOM	339	HZ	PHE	A	221	-0.863	-2.636	-4.779	1.00	0.00	H
ATOM	340	N	GLY	A	222	1.882	-8.842	0.532	1.00	0.00	N
ATOM	341	CA	GLY	A	222	1.995	-9.431	1.822	1.00	0.00	C
ATOM	342	C	GLY	A	222	1.405	-8.546	2.970	1.00	0.00	C
ATOM	343	O	GLY	A	222	0.715	-9.000	3.880	1.00	0.00	O
ATOM	344	H	GLY	A	222	2.724	-8.553	0.055	1.00	0.00	H
ATOM	345	HA2	GLY	A	222	1.419	-10.355	1.767	1.00	0.00	H
ATOM	346	HA3	GLY	A	222	3.053	-9.621	2.066	1.00	0.00	H
ATOM	347	N	GLY	A	223	1.635	-7.236	2.875	1.00	0.00	N
ATOM	348	CA	GLY	A	223	0.854	-6.243	3.639	1.00	0.00	C
ATOM	349	C	GLY	A	223	-0.622	-6.383	3.592	1.00	0.00	C
ATOM	350	O	GLY	A	223	-1.263	-6.338	4.624	1.00	0.00	O
ATOM	351	H	GLY	A	223	2.368	-6.912	2.260	1.00	0.00	H
ATOM	352	HA2	GLY	A	223	1.097	-6.409	4.689	1.00	0.00	H
ATOM	353	HA3	GLY	A	223	1.147	-5.230	3.269	1.00	0.00	H
ATOM	354	N	HIS	A	224	-1.283	-6.735	2.510	1.00	0.00	N
ATOM	355	CA	HIS	A	224	-2.719	-6.964	2.375	1.00	0.00	C
ATOM	356	CB	HIS	A	224	-3.452	-5.655	2.418	1.00	0.00	C
ATOM	357	ND1	HIS	A	224	-2.017	-4.070	0.835	1.00	0.00	N
ATOM	358	CG	HIS	A	224	-3.106	-4.869	1.122	1.00	0.00	C

ATOM	359	CE1	HIS	A	224	-2.198	-3.555	-0.404	1.00	0.00	C
ATOM	360	NE2	HIS	A	224	-3.299	-4.025	-0.965	1.00	0.00	N
ATOM	361	CD2	HIS	A	224	-3.856	-4.810	-0.002	1.00	0.00	C
ATOM	362	C	HIS	A	224	-3.238	-8.138	3.241	1.00	0.00	C
ATOM	363	O	HIS	A	224	-4.303	-8.136	3.921	1.00	0.00	O
ATOM	364	H	HIS	A	224	-0.674	-6.960	1.756	1.00	0.00	H
ATOM	365	HA	HIS	A	224	-2.880	-7.246	1.352	1.00	0.00	H
ATOM	366	HB2	HIS	A	224	-3.093	-5.040	3.277	1.00	0.00	H
ATOM	367	HB3	HIS	A	224	-4.533	-5.798	2.627	1.00	0.00	H
ATOM	368	HD1	HIS	A	224	-1.226	-3.879	1.414	1.00	0.00	H
ATOM	369	HD2	HIS	A	224	-4.779	-5.382	-0.099	1.00	0.00	H
ATOM	370	HE1	HIS	A	224	-1.492	-3.031	-1.039	1.00	0.00	H
ATOM	371	N	GLY	A	225	-2.477	-9.237	3.200	1.00	0.00	N
ATOM	372	CA	GLY	A	225	-2.706	-10.411	4.050	1.00	0.00	C
ATOM	373	C	GLY	A	225	-2.566	-10.184	5.542	1.00	0.00	C
ATOM	374	O	GLY	A	225	-3.482	-10.499	6.332	1.00	0.00	O
ATOM	375	H	GLY	A	225	-1.704	-9.260	2.550	1.00	0.00	H
ATOM	376	HA2	GLY	A	225	-3.751	-10.684	3.901	1.00	0.00	H
ATOM	377	HA3	GLY	A	225	-1.997	-11.209	3.740	1.00	0.00	H
ATOM	378	HC	GLY	A	225	-1.659	-9.743	5.929	1.00	0.00	H
TER	379		GLY	A	225						
HETATM	380	C1	HAL	B	274	-0.079	3.882	0.883	1.00	0.00	C
HETATM	381	C2	HAL	B	274	-0.146	2.403	1.066	1.00	0.00	C
HETATM	382	N	HAL	B	274	-1.157	1.741	0.128	1.00	0.00	N1+
HETATM	383	C3	HAL	B	274	-3.531	1.486	-0.605	1.00	0.00	C
HETATM	384	C4	HAL	B	274	-3.213	-0.033	-0.937	1.00	0.00	C
HETATM	385	C5	HAL	B	274	-2.562	2.000	0.488	1.00	0.00	C
HETATM	386	C6	HAL	B	274	-1.724	-0.233	-1.280	1.00	0.00	C
HETATM	387	C7	HAL	B	274	-0.924	0.296	-0.061	1.00	0.00	C

```
HETATM  388  O1  HAL B 274      -3.546  -0.737   0.251  1.00  0.00           O
HETATM  389  C8  HAL B 274      -4.085  -0.466  -2.106  1.00  0.00           C
HETATM  390  C9  HAL B 274      -5.428  -0.192  -2.064  1.00  0.00           C
HETATM  391  C10 HAL B 274      -3.568  -0.963  -3.325  1.00  0.00           C
HETATM  392  C11 HAL B 274      -6.268  -0.441  -3.178  1.00  0.00           C
HETATM  393  C12 HAL B 274      -4.370  -1.380  -4.390  1.00  0.00           C
HETATM  394  C13 HAL B 274      -5.728  -0.995  -4.344  1.00  0.00           C
HETATM  395 CL   HAL B 274      -6.684  -1.320  -5.714  1.00  0.00          CL
HETATM  396  C14 HAL B 274       1.190   4.509   1.578  1.00  0.00           C
HETATM  397  C15 HAL B 274       2.488   4.035   0.991  1.00  0.00           C
HETATM  398  O2  HAL B 274       2.630   4.184  -0.190  1.00  0.00           O
HETATM  399  C16 HAL B 274       3.610   3.535   1.852  1.00  0.00           C
HETATM  400  C17 HAL B 274       4.925   3.541   1.379  1.00  0.00           C
HETATM  401  C18 HAL B 274       3.408   3.155   3.135  1.00  0.00           C
HETATM  402  C19 HAL B 274       6.016   3.167   2.173  1.00  0.00           C
HETATM  403  C20 HAL B 274       4.415   2.730   4.021  1.00  0.00           C
HETATM  404  C21 HAL B 274       5.768   2.772   3.512  1.00  0.00           C
HETATM  405  F   HAL B 274       6.853   2.249   4.186  1.00  0.00           F
HETATM  406  H   HAL B 274      -3.383  -1.668   0.081  1.00  0.00           H
HETATM  407  HC2 HAL B 274      -1.084   4.236   1.190  1.00  0.00           H
HETATM  408  HC3 HAL B 274      -0.017   4.071  -0.203  1.00  0.00           H
HETATM  409  HC4 HAL B 274      -0.395   2.138   2.114  1.00  0.00           H
HETATM  410  HC5 HAL B 274       0.809   1.916   0.823  1.00  0.00           H
HETATM  411  HN  HAL B 274      -1.001   2.190  -0.794  1.00  0.00           H
HETATM  412  HC6 HAL B 274      -4.558   1.614  -0.229  1.00  0.00           H
HETATM  413  HC7 HAL B 274      -3.323   2.041  -1.549  1.00  0.00           H
HETATM  414  HC8 HAL B 274      -2.666   3.072   0.703  1.00  0.00           H
HETATM  415  HC9 HAL B 274      -2.608   1.461   1.447  1.00  0.00           H
HETATM  416 HC10 HAL B 274      -1.485   0.303  -2.205  1.00  0.00           H
```

HETATM	417	HC11	HAL	B	274	-1.533	-1.331	-1.399	1.00	0.00	H
HETATM	418	HC12	HAL	B	274	-1.140	-0.209	0.891	1.00	0.00	H
HETATM	419	HC13	HAL	B	274	0.128	0.171	-0.379	1.00	0.00	H
HETATM	420	HC	HAL	B	274	-5.817	0.392	-1.245	1.00	0.00	H
HETATM	421	HC1	HAL	B	274	-2.518	-1.231	-3.270	1.00	0.00	H
HETATM	422	HC14	HAL	B	274	-7.302	-0.151	-3.054	1.00	0.00	H
HETATM	423	HC15	HAL	B	274	-4.056	-1.897	-5.287	1.00	0.00	H
HETATM	424	HC16	HAL	B	274	1.180	4.320	2.680	1.00	0.00	H
HETATM	425	HC17	HAL	B	274	1.156	5.612	1.422	1.00	0.00	H
HETATM	426	HC18	HAL	B	274	5.046	3.832	0.351	1.00	0.00	H
HETATM	427	HC19	HAL	B	274	2.429	2.931	3.512	1.00	0.00	H
HETATM	428	HC20	HAL	B	274	7.052	3.114	1.871	1.00	0.00	H
HETATM	429	HC21	HAL	B	274	4.187	2.348	5.005	1.00	0.00	H
HETATM	430	OH2	TIP	X4007		10.935	2.653	5.307	1.00	0.00	O
HETATM	431	H1	TIP	X4007		10.417	3.090	5.977	1.00	0.00	H
HETATM	432	H2	TIP	X4007		11.318	1.902	5.749	1.00	0.00	H
HETATM	433	OH2	TIP	X6159		1.599	2.434	7.287	1.00	0.00	O
HETATM	434	H1	TIP	X6159		1.593	2.243	8.218	1.00	0.00	H
HETATM	435	H2	TIP	X6159		1.262	1.641	6.872	1.00	0.00	H
HETATM	436	OH2	TIP	X6241		10.950	1.165	2.443	1.00	0.00	O
HETATM	437	H1	TIP	X6241		10.385	0.397	2.277	1.00	0.00	H
HETATM	438	H2	TIP	X6241		10.356	1.805	2.853	1.00	0.00	H
HETATM	439	OH2	TIP	X9136		2.654	5.281	7.269	1.00	0.00	O
HETATM	440	H1	TIP	X9136		2.365	4.384	7.098	1.00	0.00	H
HETATM	441	H2	TIP	X9136		3.567	5.202	7.514	1.00	0.00	H
HETATM	442	OH2	TIP	X	112	5.298	4.676	7.878	1.00	0.00	O
HETATM	443	H1	TIP	X	112	6.136	4.913	8.288	1.00	0.00	H
HETATM	444	H2	TIP	X	112	5.045	3.853	8.312	1.00	0.00	H
HETATM	445	OH2	TIP	X1516		-2.127	-1.702	2.950	1.00	0.00	O

```
HETATM  446  H1  TIP X1516      -2.613  -0.890   3.147  1.00  0.00           H
HETATM  447  H2  TIP X1516      -2.480  -2.350   3.542  1.00  0.00           H
HETATM  448  OH2 TIP X1551       0.568   4.357   4.948  1.00  0.00           O
HETATM  449  H1  TIP X1551       0.949   5.225   5.042  1.00  0.00           H
HETATM  450  H2  TIP X1551       0.961   3.834   5.634  1.00  0.00           H
HETATM  451  OH2 TIP X1865      -3.565   0.690   3.784  1.00  0.00           O
HETATM  452  H1  TIP X1865      -2.943   1.416   3.816  1.00  0.00           H
HETATM  453  H2  TIP X1865      -4.387   1.069   4.103  1.00  0.00           H
HETATM  454  OH2 TIP X3199       1.569   6.920   5.005  1.00  0.00           O
HETATM  455  H1  TIP X3199       1.150   7.585   5.559  1.00  0.00           H
HETATM  456  H2  TIP X3199       2.259   6.540   5.574  1.00  0.00           H
HETATM  457  OH2 TIP X6753       3.336   3.980  -2.917  1.00  0.00           O
HETATM  458  H1  TIP X6753       2.524   3.798  -3.394  1.00  0.00           H
HETATM  459  H2  TIP X6753       3.032   4.362  -2.081  1.00  0.00           H
HETATM  460  OH2 TIP X6921       6.266  -0.260   6.635  1.00  0.00           O
HETATM  461  H1  TIP X6921       6.221   0.684   6.772  1.00  0.00           H
HETATM  462  H2  TIP X6921       5.902  -0.396   5.751  1.00  0.00           H
HETATM  463  OH2 TIP X8074      -1.461   2.613   4.301  1.00  0.00           O
HETATM  464  H1  TIP X8074      -0.903   1.957   4.740  1.00  0.00           H
HETATM  465  H2  TIP X8074      -0.938   3.411   4.317  1.00  0.00           H
HETATM  466  OH2 TIP X9899       0.520  -0.722   2.609  1.00  0.00           O
HETATM  467  H1  TIP X9899       0.779  -1.332   1.925  1.00  0.00           H
HETATM  468  H2  TIP X9899      -0.284  -1.092   2.980  1.00  0.00           H
HETATM  469  OH2 TIP X4055       3.254   0.251   1.667  1.00  0.00           O
HETATM  470  H1  TIP X4055       3.263   0.330   0.704  1.00  0.00           H
HETATM  471  H2  TIP X4055       2.335   0.337   1.915  1.00  0.00           H
HETATM  472  OH2 TIP X4700       5.568  -0.465   2.797  1.00  0.00           O
HETATM  473  H1  TIP X4700       4.820  -0.075   2.353  1.00  0.00           H
HETATM  474  H2  TIP X4700       6.319  -0.279   2.232  1.00  0.00           H
```

```
HETATM  475  OH2 TIP X6471       2.001  -2.257   0.098  1.00  0.00            O
HETATM  476  H1  TIP X6471       2.946  -2.352   0.140  1.00  0.00            H
HETATM  477  H2  TIP X6471       1.802  -2.150  -0.838  1.00  0.00            H
HETATM  478  OH2 TIP X 546       3.636  -1.680   4.851  1.00  0.00            O
HETATM  479  H1  TIP X 546       2.819  -1.307   4.546  1.00  0.00            H
HETATM  480  H2  TIP X 546       4.202  -1.683   4.085  1.00  0.00            H
HETATM  481  OH2 TIP X3340       2.631   1.255  -1.258  1.00  0.00            O
HETATM  482  H1  TIP X3340       2.834   2.155  -1.492  1.00  0.00            H
HETATM  483  H2  TIP X3340       2.159   0.911  -2.026  1.00  0.00            H
HETATM  484  OH2 TIP X8458       6.795   2.580  -1.620  1.00  0.00            O
HETATM  485  H1  TIP X8458       7.077   2.574  -2.531  1.00  0.00            H
HETATM  486  H2  TIP X8458       5.845   2.476  -1.648  1.00  0.00            H
HETATM  487  OH2 TIP X3789       1.101   1.139   4.788  1.00  0.00            O
HETATM  488  H1  TIP X3789       0.595   0.362   5.071  1.00  0.00            H
HETATM  489  H2  TIP X3789       1.397   0.924   3.911  1.00  0.00            H
HETATM  490  OH2 TIP X6384       7.493   0.958   0.577  1.00  0.00            O
HETATM  491  H1  TIP X6384       7.778   0.105   0.280  1.00  0.00            H
HETATM  492  H2  TIP X6384       7.280   1.437  -0.213  1.00  0.00            H
HETATM  493  OH2 TIP X7052       0.987   0.295  -3.214  1.00  0.00            O
HETATM  494  H1  TIP X7052       0.494   1.113  -3.339  1.00  0.00            H
HETATM  495  H2  TIP X7052       1.190   0.012  -4.108  1.00  0.00            H
HETATM  496  OH2 TIP X1729      -4.546  -6.089  -2.757  1.00  0.00            O
HETATM  497  H1  TIP X1729      -4.139  -5.464  -2.154  1.00  0.00            H
HETATM  498  H2  TIP X1729      -5.429  -5.760  -2.872  1.00  0.00            H
END
```

Haloperidol (Cluster structure 3)

```
ATOM      1  N   GLY A  76      -1.375   8.262   1.662  1.00  0.00            N
ATOM      2  CA  GLY A  76      -0.594   7.311   0.969  1.00  0.00            C
```

ATOM	3	C	GLY	A	76	-1.313	6.192	0.254	1.00	0.00	C
ATOM	4	O	GLY	A	76	-1.126	5.925	-0.940	1.00	0.00	O
ATOM	5	H1	GLY	A	76	-1.275	9.124	1.182	1.00	0.00	H
ATOM	6	H2	GLY	A	76	-1.186	8.142	2.628	1.00	0.00	H
ATOM	7	HA2	GLY	A	76	-0.135	7.897	0.173	1.00	0.00	H
ATOM	8	HA3	GLY	A	76	0.142	6.846	1.685	1.00	0.00	H
ATOM	9	N	VAL	A	77	-2.279	5.682	1.015	1.00	0.00	N
ATOM	10	CA	VAL	A	77	-3.284	4.754	0.425	1.00	0.00	C
ATOM	11	CB	VAL	A	77	-3.983	4.085	1.543	1.00	0.00	C
ATOM	12	CG1	VAL	A	77	-5.267	3.237	1.075	1.00	0.00	C
ATOM	13	CG2	VAL	A	77	-2.969	3.069	2.152	1.00	0.00	C
ATOM	14	C	VAL	A	77	-4.254	5.332	-0.617	1.00	0.00	C
ATOM	15	O	VAL	A	77	-4.567	4.664	-1.555	1.00	0.00	O
ATOM	16	H	VAL	A	77	-2.430	5.991	1.949	1.00	0.00	H
ATOM	17	HA	VAL	A	77	-2.785	3.880	0.025	1.00	0.00	H
ATOM	18	HB	VAL	A	77	-4.223	4.810	2.331	1.00	0.00	H
ATOM	19	HG11	VAL	A	77	-5.129	2.739	0.086	1.00	0.00	H
ATOM	20	HG12	VAL	A	77	-6.125	3.941	1.023	1.00	0.00	H
ATOM	21	HG13	VAL	A	77	-5.442	2.425	1.820	1.00	0.00	H
ATOM	22	HG21	VAL	A	77	-1.893	3.376	2.233	1.00	0.00	H
ATOM	23	HG22	VAL	A	77	-2.985	2.179	1.489	1.00	0.00	H
ATOM	24	HG23	VAL	A	77	-3.357	2.710	3.117	1.00	0.00	H
ATOM	25	N	GLY	A	78	-4.699	6.658	-0.417	1.00	0.00	N
ATOM	26	CA	GLY	A	78	-5.668	7.245	-1.449	1.00	0.00	C
ATOM	27	C	GLY	A	78	-4.974	7.305	-2.743	1.00	0.00	C
ATOM	28	O	GLY	A	78	-5.497	6.854	-3.758	1.00	0.00	O
ATOM	29	H	GLY	A	78	-4.390	7.199	0.377	1.00	0.00	H
ATOM	30	HA2	GLY	A	78	-6.568	6.643	-1.575	1.00	0.00	H
ATOM	31	HA3	GLY	A	78	-5.943	8.227	-1.097	1.00	0.00	H

ATOM	32	N	GLY	A	79	-3.791	7.873	-2.729	1.00	0.00	N
ATOM	33	CA	GLY	A	79	-2.950	7.953	-3.907	1.00	0.00	C
ATOM	34	C	GLY	A	79	-2.458	6.641	-4.529	1.00	0.00	C
ATOM	35	O	GLY	A	79	-2.378	6.336	-5.741	1.00	0.00	O
ATOM	36	H	GLY	A	79	-3.453	8.270	-1.864	1.00	0.00	H
ATOM	37	HA2	GLY	A	79	-3.607	8.382	-4.663	1.00	0.00	H
ATOM	38	HA3	GLY	A	79	-2.051	8.578	-3.664	1.00	0.00	H
ATOM	39	N	ASP	A	80	-2.138	5.719	-3.643	1.00	0.00	N
ATOM	40	CA	ASP	A	80	-1.921	4.259	-3.869	1.00	0.00	C
ATOM	41	CB	ASP	A	80	-0.447	4.169	-4.322	1.00	0.00	C
ATOM	42	CG	ASP	A	80	0.081	2.730	-4.368	1.00	0.00	C
ATOM	43	OD1	ASP	A	80	1.198	2.451	-4.885	1.00	0.00	O
ATOM	44	OD2	ASP	A	80	-0.494	1.885	-3.700	1.00	0.00	O1-
ATOM	45	C	ASP	A	80	-2.852	3.561	-4.806	1.00	0.00	C
ATOM	46	O	ASP	A	80	-2.529	2.922	-5.804	1.00	0.00	O
ATOM	47	H	ASP	A	80	-1.951	6.090	-2.739	1.00	0.00	H
ATOM	48	HA	ASP	A	80	-2.059	3.721	-2.947	1.00	0.00	H
ATOM	49	HB2	ASP	A	80	0.175	4.583	-3.502	1.00	0.00	H
ATOM	50	HB3	ASP	A	80	-0.216	4.657	-5.289	1.00	0.00	H
ATOM	51	N	VAL	A	81	-4.187	3.759	-4.494	1.00	0.00	N
ATOM	52	CA	VAL	A	81	-5.299	3.280	-5.279	1.00	0.00	C
ATOM	53	CB	VAL	A	81	-6.556	3.478	-4.480	1.00	0.00	C
ATOM	54	CG1	VAL	A	81	-7.809	3.075	-5.237	1.00	0.00	C
ATOM	55	CG2	VAL	A	81	-6.502	2.568	-3.224	1.00	0.00	C
ATOM	56	C	VAL	A	81	-5.355	3.860	-6.653	1.00	0.00	C
ATOM	57	O	VAL	A	81	-5.602	3.166	-7.644	1.00	0.00	O
ATOM	58	H	VAL	A	81	-4.521	4.294	-3.738	1.00	0.00	H
ATOM	59	HA	VAL	A	81	-5.206	2.227	-5.500	1.00	0.00	H
ATOM	60	HB	VAL	A	81	-6.635	4.549	-4.229	1.00	0.00	H

ATOM	61	HG11	VAL	A	81	-7.638	2.014	-5.511	1.00	0.00	H
ATOM	62	HG12	VAL	A	81	-7.993	3.770	-6.094	1.00	0.00	H
ATOM	63	HG13	VAL	A	81	-8.659	3.158	-4.535	1.00	0.00	H
ATOM	64	HG21	VAL	A	81	-5.658	2.859	-2.565	1.00	0.00	H
ATOM	65	HG22	VAL	A	81	-6.323	1.539	-3.637	1.00	0.00	H
ATOM	66	HG23	VAL	A	81	-7.434	2.683	-2.648	1.00	0.00	H
ATOM	67	N	GLY	A	82	-5.083	5.152	-6.849	1.00	0.00	N
ATOM	68	CA	GLY	A	82	-5.009	5.787	-8.156	1.00	0.00	C
ATOM	69	C	GLY	A	82	-3.929	5.197	-8.990	1.00	0.00	C
ATOM	70	O	GLY	A	82	-4.243	4.834	-10.059	1.00	0.00	O
ATOM	71	H	GLY	A	82	-4.917	5.727	-6.036	1.00	0.00	H
ATOM	72	HA2	GLY	A	82	-5.952	5.625	-8.677	1.00	0.00	H
ATOM	73	HA3	GLY	A	82	-4.826	6.860	-8.015	1.00	0.00	H
ATOM	74	N	GLY	A	83	-2.652	5.211	-8.543	1.00	0.00	N
ATOM	75	CA	GLY	A	83	-1.517	4.633	-9.278	1.00	0.00	C
ATOM	76	C	GLY	A	83	-1.659	3.190	-9.741	1.00	0.00	C
ATOM	77	O	GLY	A	83	-1.489	2.762	-10.869	1.00	0.00	O
ATOM	78	H	GLY	A	83	-2.467	5.645	-7.650	1.00	0.00	H
ATOM	79	HA2	GLY	A	83	-1.455	5.210	-10.201	1.00	0.00	H
ATOM	80	HA3	GLY	A	83	-0.609	4.713	-8.634	1.00	0.00	H
ATOM	81	N	CYS	A	84	-2.093	2.332	-8.748	1.00	0.00	N
ATOM	82	CA	CYS	A	84	-2.395	0.981	-8.922	1.00	0.00	C
ATOM	83	CB	CYS	A	84	-2.674	0.320	-7.619	1.00	0.00	C
ATOM	84	SG	CYS	A	84	-1.232	0.230	-6.510	1.00	0.00	S
ATOM	85	C	CYS	A	84	-3.598	0.797	-9.816	1.00	0.00	C
ATOM	86	O	CYS	A	84	-3.663	-0.215	-10.591	1.00	0.00	O
ATOM	87	H	CYS	A	84	-2.210	2.576	-7.786	1.00	0.00	H
ATOM	88	HA	CYS	A	84	-1.515	0.547	-9.375	1.00	0.00	H
ATOM	89	HB2	CYS	A	84	-3.070	-0.686	-7.851	1.00	0.00	H

ATOM	90	HB3	CYS	A	84	-3.507	0.755	-6.998	1.00	0.00	H
ATOM	91	HG	CYS	A	84	-1.521	1.485	-6.217	1.00	0.00	H
ATOM	92	N	THR	A	85	-4.639	1.620	-9.729	1.00	0.00	N
ATOM	93	CA	THR	A	85	-5.828	1.454	-10.573	1.00	0.00	C
ATOM	94	CB	THR	A	85	-7.053	2.006	-10.006	1.00	0.00	C
ATOM	95	OG1	THR	A	85	-7.312	1.364	-8.782	1.00	0.00	O
ATOM	96	CG2	THR	A	85	-8.393	1.884	-10.733	1.00	0.00	C
ATOM	97	C	THR	A	85	-5.725	2.075	-11.872	1.00	0.00	C
ATOM	98	O	THR	A	85	-6.247	1.533	-12.869	1.00	0.00	O
ATOM	99	H	THR	A	85	-4.602	2.481	-9.217	1.00	0.00	H
ATOM	100	HA	THR	A	85	-5.974	0.396	-10.706	1.00	0.00	H
ATOM	101	HB	THR	A	85	-7.002	3.115	-9.899	1.00	0.00	H
ATOM	102	HG21	THR	A	85	-8.800	0.852	-10.636	1.00	0.00	H
ATOM	103	HG22	THR	A	85	-8.296	2.278	-11.767	1.00	0.00	H
ATOM	104	HG23	THR	A	85	-9.036	2.601	-10.192	1.00	0.00	H
ATOM	105	HG1	THR	A	85	-6.997	1.957	-8.097	1.00	0.00	H
ATOM	106	N	GLY	A	86	-4.933	3.155	-12.051	1.00	0.00	N
ATOM	107	CA	GLY	A	86	-4.651	3.686	-13.388	1.00	0.00	C
ATOM	108	C	GLY	A	86	-3.751	2.744	-14.195	1.00	0.00	C
ATOM	109	O	GLY	A	86	-3.981	2.546	-15.403	1.00	0.00	O
ATOM	110	H	GLY	A	86	-4.526	3.607	-11.245	1.00	0.00	H
ATOM	111	HA2	GLY	A	86	-5.599	3.788	-13.917	1.00	0.00	H
ATOM	112	HA3	GLY	A	86	-4.153	4.654	-13.291	1.00	0.00	H
ATOM	113	HC	GLY	A	86	-2.917	2.252	-13.716	1.00	0.00	H
ATOM	114	N	GLY	A	147	-5.746	0.600	10.832	1.00	0.00	N
ATOM	115	CA	GLY	A	147	-4.595	1.465	11.225	1.00	0.00	C
ATOM	116	C	GLY	A	147	-3.401	1.629	10.337	1.00	0.00	C
ATOM	117	O	GLY	A	147	-2.262	1.178	10.656	1.00	0.00	O
ATOM	118	H1	GLY	A	147	-6.466	1.217	10.657	1.00	0.00	H

ATOM	119	H2	GLY	A	147	-5.701	-0.153	11.432	1.00	0.00	H
ATOM	120	HA2	GLY	A	147	-5.015	2.470	11.175	1.00	0.00	H
ATOM	121	HA3	GLY	A	147	-4.248	1.098	12.244	1.00	0.00	H
ATOM	122	N	CYS	A	148	-3.590	2.280	9.210	1.00	0.00	N
ATOM	123	CA	CYS	A	148	-2.575	2.646	8.293	1.00	0.00	C
ATOM	124	CB	CYS	A	148	-1.329	3.428	8.791	1.00	0.00	C
ATOM	125	SG	CYS	A	148	-1.884	5.026	9.522	1.00	0.00	S
ATOM	126	C	CYS	A	148	-2.147	1.510	7.338	1.00	0.00	C
ATOM	127	O	CYS	A	148	-1.040	1.586	6.811	1.00	0.00	O
ATOM	128	H	CYS	A	148	-4.516	2.544	8.948	1.00	0.00	H
ATOM	129	HA	CYS	A	148	-3.057	3.390	7.679	1.00	0.00	H
ATOM	130	HB2	CYS	A	148	-0.676	3.614	7.912	1.00	0.00	H
ATOM	131	HB3	CYS	A	148	-0.698	2.965	9.568	1.00	0.00	H
ATOM	132	HG	CYS	A	148	-2.663	5.669	8.648	1.00	0.00	H
ATOM	133	N	ILE	A	149	-3.026	0.542	7.003	1.00	0.00	N
ATOM	134	CA	ILE	A	149	-2.720	-0.407	5.957	1.00	0.00	C
ATOM	135	CB	ILE	A	149	-4.013	-0.725	5.232	1.00	0.00	C
ATOM	136	CG2	ILE	A	149	-3.865	-1.490	3.996	1.00	0.00	C
ATOM	137	CG1	ILE	A	149	-5.258	-1.165	6.135	1.00	0.00	C
ATOM	138	CD1	ILE	A	149	-6.594	-1.146	5.381	1.00	0.00	C
ATOM	139	C	ILE	A	149	-2.193	-1.778	6.473	1.00	0.00	C
ATOM	140	O	ILE	A	149	-1.321	-2.388	5.817	1.00	0.00	O
ATOM	141	H	ILE	A	149	-3.740	0.338	7.663	1.00	0.00	H
ATOM	142	HA	ILE	A	149	-2.017	0.061	5.284	1.00	0.00	H
ATOM	143	HB	ILE	A	149	-4.313	0.283	4.909	1.00	0.00	H
ATOM	144	HG12	ILE	A	149	-5.501	-0.594	7.059	1.00	0.00	H
ATOM	145	HG13	ILE	A	149	-5.071	-2.202	6.502	1.00	0.00	H
ATOM	146	HG21	ILE	A	149	-3.138	-0.926	3.385	1.00	0.00	H
ATOM	147	HG22	ILE	A	149	-4.823	-1.498	3.449	1.00	0.00	H

ATOM	148	HG23	ILE	A	149	-3.458	-2.499	4.226	1.00	0.00	H
ATOM	149	HD11	ILE	A	149	-6.591	-1.913	4.570	1.00	0.00	H
ATOM	150	HD12	ILE	A	149	-6.607	-0.195	4.805	1.00	0.00	H
ATOM	151	HD13	ILE	A	149	-7.437	-1.265	6.087	1.00	0.00	H
ATOM	152	N	ILE	A	150	-2.648	-2.254	7.618	1.00	0.00	N
ATOM	153	CA	ILE	A	150	-2.214	-3.516	8.260	1.00	0.00	C
ATOM	154	CB	ILE	A	150	-2.592	-3.596	9.723	1.00	0.00	C
ATOM	155	CG2	ILE	A	150	-2.043	-4.901	10.350	1.00	0.00	C
ATOM	156	CG1	ILE	A	150	-1.961	-2.365	10.424	1.00	0.00	C
ATOM	157	CD1	ILE	A	150	-0.373	-2.285	10.351	1.00	0.00	C
ATOM	158	C	ILE	A	150	-2.779	-4.785	7.566	1.00	0.00	C
ATOM	159	O	ILE	A	150	-2.060	-5.631	7.019	1.00	0.00	O
ATOM	160	H	ILE	A	150	-3.376	-1.706	8.027	1.00	0.00	H
ATOM	161	HA	ILE	A	150	-1.134	-3.549	8.172	1.00	0.00	H
ATOM	162	HB	ILE	A	150	-3.687	-3.533	9.872	1.00	0.00	H
ATOM	163	HG12	ILE	A	150	-2.264	-2.283	11.487	1.00	0.00	H
ATOM	164	HG13	ILE	A	150	-2.242	-1.406	9.948	1.00	0.00	H
ATOM	165	HG21	ILE	A	150	-2.612	-5.826	10.076	1.00	0.00	H
ATOM	166	HG22	ILE	A	150	-2.156	-4.842	11.457	1.00	0.00	H
ATOM	167	HG23	ILE	A	150	-0.974	-5.080	10.119	1.00	0.00	H
ATOM	168	HD11	ILE	A	150	0.163	-3.064	10.922	1.00	0.00	H
ATOM	169	HD12	ILE	A	150	0.033	-1.302	10.673	1.00	0.00	H
ATOM	170	HD13	ILE	A	150	-0.076	-2.326	9.278	1.00	0.00	H
ATOM	171	N	GLY	A	151	-4.081	-4.821	7.330	1.00	0.00	N
ATOM	172	CA	GLY	A	151	-4.681	-5.842	6.533	1.00	0.00	C
ATOM	173	C	GLY	A	151	-5.851	-5.428	5.754	1.00	0.00	C
ATOM	174	O	GLY	A	151	-5.938	-5.522	4.543	1.00	0.00	O
ATOM	175	H	GLY	A	151	-4.669	-4.102	7.727	1.00	0.00	H
ATOM	176	HA2	GLY	A	151	-3.933	-6.123	5.791	1.00	0.00	H

ATOM	177	HA3	GLY	A	151	-4.963	-6.694	7.203	1.00	0.00	H
ATOM	178	HC	GLY	A	151	-6.692	-5.012	6.289	1.00	0.00	H
ATOM	179	N	GLY	A	154	-12.091	-5.510	3.736	1.00	0.00	N
ATOM	180	CA	GLY	A	154	-12.714	-4.731	2.736	1.00	0.00	C
ATOM	181	C	GLY	A	154	-11.809	-4.116	1.630	1.00	0.00	C
ATOM	182	O	GLY	A	154	-12.160	-4.240	0.428	1.00	0.00	O
ATOM	183	H1	GLY	A	154	-12.400	-6.437	3.599	1.00	0.00	H
ATOM	184	H2	GLY	A	154	-12.179	-5.003	4.578	1.00	0.00	H
ATOM	185	HA2	GLY	A	154	-13.348	-5.459	2.230	1.00	0.00	H
ATOM	186	HA3	GLY	A	154	-13.273	-3.881	3.209	1.00	0.00	H
ATOM	187	N	PHE	A	155	-10.686	-3.601	2.055	1.00	0.00	N
ATOM	188	CA	PHE	A	155	-9.645	-3.039	1.263	1.00	0.00	C
ATOM	189	CB	PHE	A	155	-8.713	-2.240	2.182	1.00	0.00	C
ATOM	190	CG	PHE	A	155	-7.462	-1.825	1.489	1.00	0.00	C
ATOM	191	CD1	PHE	A	155	-7.414	-0.473	1.073	1.00	0.00	C
ATOM	192	CE1	PHE	A	155	-6.328	0.012	0.357	1.00	0.00	C
ATOM	193	CZ	PHE	A	155	-5.181	-0.869	0.054	1.00	0.00	C
ATOM	194	CD2	PHE	A	155	-6.353	-2.623	1.280	1.00	0.00	C
ATOM	195	CE2	PHE	A	155	-5.194	-2.149	0.662	1.00	0.00	C
ATOM	196	C	PHE	A	155	-9.052	-4.085	0.384	1.00	0.00	C
ATOM	197	O	PHE	A	155	-8.762	-3.879	-0.841	1.00	0.00	O
ATOM	198	H	PHE	A	155	-10.515	-3.572	3.041	1.00	0.00	H
ATOM	199	HA	PHE	A	155	-9.932	-2.246	0.583	1.00	0.00	H
ATOM	200	HB2	PHE	A	155	-8.305	-2.829	3.024	1.00	0.00	H
ATOM	201	HB3	PHE	A	155	-9.248	-1.321	2.545	1.00	0.00	H
ATOM	202	HD1	PHE	A	155	-8.157	0.219	1.437	1.00	0.00	H
ATOM	203	HD2	PHE	A	155	-6.276	-3.599	1.732	1.00	0.00	H
ATOM	204	HE1	PHE	A	155	-6.339	1.019	-0.067	1.00	0.00	H
ATOM	205	HE2	PHE	A	155	-4.348	-2.830	0.595	1.00	0.00	H

ATOM	206	HZ	PHE	A	155	-4.324	-0.513	-0.487	1.00	0.00	H
ATOM	207	N	GLY	A	156	-8.913	-5.352	0.987	1.00	0.00	N
ATOM	208	CA	GLY	A	156	-8.429	-6.542	0.339	1.00	0.00	C
ATOM	209	C	GLY	A	156	-9.363	-6.943	-0.790	1.00	0.00	C
ATOM	210	O	GLY	A	156	-8.928	-7.372	-1.847	1.00	0.00	O
ATOM	211	H	GLY	A	156	-9.176	-5.431	1.959	1.00	0.00	H
ATOM	212	HA2	GLY	A	156	-7.438	-6.339	-0.066	1.00	0.00	H
ATOM	213	HA3	GLY	A	156	-8.376	-7.358	1.061	1.00	0.00	H
ATOM	214	HC	GLY	A	156	-10.430	-6.847	-0.655	1.00	0.00	H
ATOM	215	N	GLY	A	158	-11.759	-4.669	-2.320	1.00	0.00	N
ATOM	216	CA	GLY	A	158	-11.980	-3.449	-3.071	1.00	0.00	C
ATOM	217	C	GLY	A	158	-10.896	-3.160	-4.062	1.00	0.00	C
ATOM	218	O	GLY	A	158	-11.150	-2.973	-5.239	1.00	0.00	O
ATOM	219	H1	GLY	A	158	-12.498	-5.290	-2.586	1.00	0.00	H
ATOM	220	H2	GLY	A	158	-11.628	-4.383	-1.369	1.00	0.00	H
ATOM	221	HA2	GLY	A	158	-12.900	-3.580	-3.641	1.00	0.00	H
ATOM	222	HA3	GLY	A	158	-12.061	-2.606	-2.362	1.00	0.00	H
ATOM	223	N	SER	A	159	-9.653	-3.262	-3.657	1.00	0.00	N
ATOM	224	CA	SER	A	159	-8.415	-3.196	-4.409	1.00	0.00	C
ATOM	225	CB	SER	A	159	-7.094	-3.127	-3.512	1.00	0.00	C
ATOM	226	OG	SER	A	159	-7.258	-2.008	-2.706	1.00	0.00	O
ATOM	227	C	SER	A	159	-8.247	-4.424	-5.317	1.00	0.00	C
ATOM	228	O	SER	A	159	-7.745	-4.326	-6.453	1.00	0.00	O
ATOM	229	H	SER	A	159	-9.516	-3.440	-2.686	1.00	0.00	H
ATOM	230	HA	SER	A	159	-8.420	-2.326	-5.057	1.00	0.00	H
ATOM	231	HB2	SER	A	159	-6.249	-3.168	-4.223	1.00	0.00	H
ATOM	232	HB3	SER	A	159	-7.026	-3.986	-2.816	1.00	0.00	H
ATOM	233	HG	SER	A	159	-7.297	-2.296	-1.792	1.00	0.00	H
ATOM	234	N	GLY	A	160	-8.704	-5.639	-4.913	1.00	0.00	N

ATOM	235	CA	GLY	A	160	-8.841	-6.791	-5.849	1.00	0.00	C
ATOM	236	C	GLY	A	160	-9.786	-6.495	-6.990	1.00	0.00	C
ATOM	237	O	GLY	A	160	-9.551	-6.891	-8.099	1.00	0.00	O
ATOM	238	H	GLY	A	160	-8.960	-5.766	-3.944	1.00	0.00	H
ATOM	239	HA2	GLY	A	160	-7.866	-6.997	-6.291	1.00	0.00	H
ATOM	240	HA3	GLY	A	160	-9.208	-7.651	-5.279	1.00	0.00	H
ATOM	241	N	GLY	A	161	-10.996	-5.889	-6.712	1.00	0.00	N
ATOM	242	CA	GLY	A	161	-12.032	-5.636	-7.730	1.00	0.00	C
ATOM	243	C	GLY	A	161	-11.598	-4.714	-8.903	1.00	0.00	C
ATOM	244	O	GLY	A	161	-11.802	-4.966	-10.073	1.00	0.00	O
ATOM	245	H	GLY	A	161	-11.181	-5.604	-5.761	1.00	0.00	H
ATOM	246	HA2	GLY	A	161	-12.250	-6.608	-8.172	1.00	0.00	H
ATOM	247	HA3	GLY	A	161	-12.909	-5.179	-7.239	1.00	0.00	H
ATOM	248	N	VAL	A	162	-10.934	-3.611	-8.516	1.00	0.00	N
ATOM	249	CA	VAL	A	162	-10.780	-2.432	-9.352	1.00	0.00	C
ATOM	250	CB	VAL	A	162	-11.045	-1.140	-8.558	1.00	0.00	C
ATOM	251	CG1	VAL	A	162	-12.521	-1.194	-7.988	1.00	0.00	C
ATOM	252	CG2	VAL	A	162	-10.014	-0.952	-7.428	1.00	0.00	C
ATOM	253	C	VAL	A	162	-9.498	-2.465	-9.978	1.00	0.00	C
ATOM	254	O	VAL	A	162	-9.169	-1.678	-10.879	1.00	0.00	O
ATOM	255	H	VAL	A	162	-10.765	-3.420	-7.556	1.00	0.00	H
ATOM	256	HA	VAL	A	162	-11.456	-2.517	-10.202	1.00	0.00	H
ATOM	257	HB	VAL	A	162	-10.923	-0.283	-9.261	1.00	0.00	H
ATOM	258	HG11	VAL	A	162	-12.914	-0.152	-7.968	1.00	0.00	H
ATOM	259	HG12	VAL	A	162	-12.624	-1.637	-6.975	1.00	0.00	H
ATOM	260	HG13	VAL	A	162	-13.096	-1.858	-8.676	1.00	0.00	H
ATOM	261	HG21	VAL	A	162	-10.301	-0.035	-6.864	1.00	0.00	H
ATOM	262	HG22	VAL	A	162	-8.997	-0.818	-7.850	1.00	0.00	H
ATOM	263	HG23	VAL	A	162	-10.056	-1.808	-6.738	1.00	0.00	H

ATOM	264	N	SER	A	163	-8.597	-3.455	-9.544	1.00	0.00	N
ATOM	265	CA	SER	A	163	-7.288	-3.613	-10.144	1.00	0.00	C
ATOM	266	CB	SER	A	163	-6.131	-3.255	-9.114	1.00	0.00	C
ATOM	267	OG	SER	A	163	-4.830	-3.312	-9.691	1.00	0.00	O
ATOM	268	C	SER	A	163	-7.117	-5.042	-10.618	1.00	0.00	C
ATOM	269	O	SER	A	163	-7.146	-5.243	-11.853	1.00	0.00	O
ATOM	270	H	SER	A	163	-8.851	-3.997	-8.739	1.00	0.00	H
ATOM	271	HA	SER	A	163	-7.245	-2.955	-10.989	1.00	0.00	H
ATOM	272	HB2	SER	A	163	-6.217	-4.009	-8.300	1.00	0.00	H
ATOM	273	HB3	SER	A	163	-6.383	-2.238	-8.738	1.00	0.00	H
ATOM	274	HG	SER	A	163	-4.665	-2.399	-9.963	1.00	0.00	H
ATOM	275	N	GLY	A	164	-7.006	-6.041	-9.707	1.00	0.00	N
ATOM	276	CA	GLY	A	164	-6.780	-7.426	-10.181	1.00	0.00	C
ATOM	277	C	GLY	A	164	-7.945	-8.009	-10.997	1.00	0.00	C
ATOM	278	O	GLY	A	164	-7.728	-8.799	-11.925	1.00	0.00	O
ATOM	279	H	GLY	A	164	-7.076	-5.843	-8.719	1.00	0.00	H
ATOM	280	HA2	GLY	A	164	-5.921	-7.392	-10.851	1.00	0.00	H
ATOM	281	HA3	GLY	A	164	-6.603	-8.070	-9.306	1.00	0.00	H
ATOM	282	HC	GLY	A	164	-8.960	-7.727	-10.759	1.00	0.00	H
ATOM	283	N	GLY	A	216	3.334	-8.841	-8.526	1.00	0.00	N
ATOM	284	CA	GLY	A	216	2.975	-7.814	-7.536	1.00	0.00	C
ATOM	285	C	GLY	A	216	1.724	-8.170	-6.771	1.00	0.00	C
ATOM	286	O	GLY	A	216	1.719	-8.075	-5.587	1.00	0.00	O
ATOM	287	H1	GLY	A	216	4.207	-9.211	-8.209	1.00	0.00	H
ATOM	288	H2	GLY	A	216	3.238	-8.390	-9.413	1.00	0.00	H
ATOM	289	HA2	GLY	A	216	3.787	-7.740	-6.812	1.00	0.00	H
ATOM	290	HA3	GLY	A	216	2.825	-6.862	-8.060	1.00	0.00	H
ATOM	291	N	TRP	A	217	0.619	-8.552	-7.413	1.00	0.00	N
ATOM	292	CA	TRP	A	217	-0.556	-8.995	-6.764	1.00	0.00	C

ATOM	293	CB	TRP	A	217	-1.340	-7.903	-6.037	1.00	0.00	C
ATOM	294	CG	TRP	A	217	-1.697	-6.696	-6.874	1.00	0.00	C
ATOM	295	CD1	TRP	A	217	-2.734	-6.588	-7.727	1.00	0.00	C
ATOM	296	NE1	TRP	A	217	-2.782	-5.342	-8.253	1.00	0.00	N
ATOM	297	CE2	TRP	A	217	-1.669	-4.634	-7.853	1.00	0.00	C
ATOM	298	CD2	TRP	A	217	-0.944	-5.462	-6.968	1.00	0.00	C
ATOM	299	CE3	TRP	A	217	0.213	-4.985	-6.305	1.00	0.00	C
ATOM	300	CZ3	TRP	A	217	0.624	-3.736	-6.667	1.00	0.00	C
ATOM	301	CZ2	TRP	A	217	-1.323	-3.369	-8.132	1.00	0.00	C
ATOM	302	CH2	TRP	A	217	-0.045	-2.955	-7.545	1.00	0.00	C
ATOM	303	C	TRP	A	217	-0.468	-10.192	-5.877	1.00	0.00	C
ATOM	304	O	TRP	A	217	-1.026	-10.222	-4.827	1.00	0.00	O
ATOM	305	H	TRP	A	217	0.540	-8.471	-8.409	1.00	0.00	H
ATOM	306	HA	TRP	A	217	-1.188	-9.225	-7.608	1.00	0.00	H
ATOM	307	HB2	TRP	A	217	-2.322	-8.358	-5.845	1.00	0.00	H
ATOM	308	HB3	TRP	A	217	-0.779	-7.514	-5.156	1.00	0.00	H
ATOM	309	HD1	TRP	A	217	-3.380	-7.447	-7.837	1.00	0.00	H
ATOM	310	HE1	TRP	A	217	-3.336	-4.941	-8.954	1.00	0.00	H
ATOM	311	HE3	TRP	A	217	0.882	-5.579	-5.703	1.00	0.00	H
ATOM	312	HZ2	TRP	A	217	-1.922	-2.692	-8.724	1.00	0.00	H
ATOM	313	HZ3	TRP	A	217	1.491	-3.374	-6.147	1.00	0.00	H
ATOM	314	HH2	TRP	A	217	0.266	-1.977	-7.880	1.00	0.00	H
ATOM	315	N	GLY	A	218	0.293	-11.205	-6.390	1.00	0.00	N
ATOM	316	CA	GLY	A	218	0.329	-12.539	-5.916	1.00	0.00	C
ATOM	317	C	GLY	A	218	1.356	-12.972	-4.881	1.00	0.00	C
ATOM	318	O	GLY	A	218	1.557	-14.188	-4.680	1.00	0.00	O
ATOM	319	H	GLY	A	218	0.883	-10.982	-7.179	1.00	0.00	H
ATOM	320	HA2	GLY	A	218	-0.609	-12.592	-5.363	1.00	0.00	H
ATOM	321	HA3	GLY	A	218	0.433	-13.240	-6.808	1.00	0.00	H

ATOM	322	N	GLY	A	219	2.205	-12.242	-4.081	1.00	0.00	N
ATOM	323	CA	GLY	A	219	2.370	-10.834	-3.970	1.00	0.00	C
ATOM	324	C	GLY	A	219	1.402	-10.301	-2.962	1.00	0.00	C
ATOM	325	O	GLY	A	219	0.907	-11.039	-2.083	1.00	0.00	O
ATOM	326	H	GLY	A	219	2.803	-12.791	-3.480	1.00	0.00	H
ATOM	327	HA2	GLY	A	219	3.390	-10.615	-3.655	1.00	0.00	H
ATOM	328	HA3	GLY	A	219	2.182	-10.361	-4.936	1.00	0.00	H
ATOM	329	N	PHE	A	220	1.099	-8.988	-2.947	1.00	0.00	N
ATOM	330	CA	PHE	A	220	0.398	-8.347	-1.855	1.00	0.00	C
ATOM	331	CB	PHE	A	220	0.318	-6.809	-2.223	1.00	0.00	C
ATOM	332	CG	PHE	A	220	1.660	-6.185	-1.922	1.00	0.00	C
ATOM	333	CD1	PHE	A	220	2.127	-5.763	-0.696	1.00	0.00	C
ATOM	334	CE1	PHE	A	220	3.339	-5.070	-0.570	1.00	0.00	C
ATOM	335	CZ	PHE	A	220	4.050	-4.574	-1.673	1.00	0.00	C
ATOM	336	CD2	PHE	A	220	2.403	-5.743	-3.058	1.00	0.00	C
ATOM	337	CE2	PHE	A	220	3.543	-5.002	-2.868	1.00	0.00	C
ATOM	338	C	PHE	A	220	-0.928	-8.841	-1.312	1.00	0.00	C
ATOM	339	O	PHE	A	220	-1.242	-8.720	-0.116	1.00	0.00	O
ATOM	340	H	PHE	A	220	1.501	-8.484	-3.698	1.00	0.00	H
ATOM	341	HA	PHE	A	220	1.057	-8.572	-1.043	1.00	0.00	H
ATOM	342	HB2	PHE	A	220	-0.405	-6.195	-1.641	1.00	0.00	H
ATOM	343	HB3	PHE	A	220	0.113	-6.661	-3.303	1.00	0.00	H
ATOM	344	HD1	PHE	A	220	1.484	-6.019	0.130	1.00	0.00	H
ATOM	345	HD2	PHE	A	220	2.086	-6.088	-4.027	1.00	0.00	H
ATOM	346	HE1	PHE	A	220	3.852	-4.949	0.376	1.00	0.00	H
ATOM	347	HE2	PHE	A	220	4.057	-4.699	-3.778	1.00	0.00	H
ATOM	348	HZ	PHE	A	220	5.037	-4.177	-1.547	1.00	0.00	H
ATOM	349	N	PHE	A	221	-1.681	-9.590	-2.196	1.00	0.00	N
ATOM	350	CA	PHE	A	221	-2.865	-10.345	-1.924	1.00	0.00	C

ATOM	351	CB	PHE	A	221	-4.126	-9.481	-1.658	1.00	0.00	C
ATOM	352	CG	PHE	A	221	-4.247	-8.357	-2.666	1.00	0.00	C
ATOM	353	CD1	PHE	A	221	-3.991	-7.036	-2.280	1.00	0.00	C
ATOM	354	CE1	PHE	A	221	-4.348	-6.068	-3.223	1.00	0.00	C
ATOM	355	CZ	PHE	A	221	-5.032	-6.386	-4.379	1.00	0.00	C
ATOM	356	CD2	PHE	A	221	-4.852	-8.754	-3.886	1.00	0.00	C
ATOM	357	CE2	PHE	A	221	-5.255	-7.692	-4.715	1.00	0.00	C
ATOM	358	C	PHE	A	221	-2.705	-11.415	-0.837	1.00	0.00	C
ATOM	359	O	PHE	A	221	-3.544	-11.640	0.047	1.00	0.00	O
ATOM	360	H	PHE	A	221	-1.235	-9.531	-3.091	1.00	0.00	H
ATOM	361	HA	PHE	A	221	-3.025	-10.809	-2.882	1.00	0.00	H
ATOM	362	HB2	PHE	A	221	-5.052	-10.100	-1.622	1.00	0.00	H
ATOM	363	HB3	PHE	A	221	-4.014	-9.002	-0.659	1.00	0.00	H
ATOM	364	HD1	PHE	A	221	-3.593	-6.690	-1.344	1.00	0.00	H
ATOM	365	HD2	PHE	A	221	-4.972	-9.791	-4.179	1.00	0.00	H
ATOM	366	HE1	PHE	A	221	-4.133	-5.029	-3.063	1.00	0.00	H
ATOM	367	HE2	PHE	A	221	-5.862	-7.875	-5.580	1.00	0.00	H
ATOM	368	HZ	PHE	A	221	-5.340	-5.591	-5.058	1.00	0.00	H
ATOM	369	N	GLY	A	222	-1.589	-12.116	-0.904	1.00	0.00	N
ATOM	370	CA	GLY	A	222	-1.105	-13.119	0.025	1.00	0.00	C
ATOM	371	C	GLY	A	222	-0.798	-12.518	1.291	1.00	0.00	C
ATOM	372	O	GLY	A	222	-1.119	-13.038	2.369	1.00	0.00	O
ATOM	373	H	GLY	A	222	-0.998	-11.931	-1.702	1.00	0.00	H
ATOM	374	HA2	GLY	A	222	-1.870	-13.875	0.201	1.00	0.00	H
ATOM	375	HA3	GLY	A	222	-0.215	-13.591	-0.415	1.00	0.00	H
ATOM	376	N	GLY	A	223	-0.059	-11.391	1.154	1.00	0.00	N
ATOM	377	CA	GLY	A	223	0.271	-10.559	2.316	1.00	0.00	C
ATOM	378	C	GLY	A	223	-0.941	-10.144	3.099	1.00	0.00	C
ATOM	379	O	GLY	A	223	-0.901	-10.196	4.328	1.00	0.00	O

ATOM	380	H	GLY	A	223	0.270	-11.120	0.238	1.00	0.00	H
ATOM	381	HA2	GLY	A	223	0.892	-11.152	2.987	1.00	0.00	H
ATOM	382	HA3	GLY	A	223	0.805	-9.664	1.963	1.00	0.00	H
ATOM	383	N	HIS	A	224	-2.031	-9.752	2.396	1.00	0.00	N
ATOM	384	CA	HIS	A	224	-3.306	-9.354	2.891	1.00	0.00	C
ATOM	385	CB	HIS	A	224	-3.222	-8.012	3.714	1.00	0.00	C
ATOM	386	ND1	HIS	A	224	-3.770	-5.676	2.933	1.00	0.00	N
ATOM	387	CG	HIS	A	224	-3.009	-6.818	2.797	1.00	0.00	C
ATOM	388	CE1	HIS	A	224	-3.125	-4.738	2.214	1.00	0.00	C
ATOM	389	NE2	HIS	A	224	-2.111	-5.232	1.496	1.00	0.00	N
ATOM	390	CD2	HIS	A	224	-1.990	-6.536	1.998	1.00	0.00	C
ATOM	391	C	HIS	A	224	-3.883	-10.465	3.783	1.00	0.00	C
ATOM	392	O	HIS	A	224	-4.375	-10.201	4.898	1.00	0.00	O
ATOM	393	H	HIS	A	224	-1.966	-9.657	1.406	1.00	0.00	H
ATOM	394	HA	HIS	A	224	-4.026	-9.143	2.111	1.00	0.00	H
ATOM	395	HB2	HIS	A	224	-2.449	-8.028	4.498	1.00	0.00	H
ATOM	396	HB3	HIS	A	224	-4.222	-7.826	4.164	1.00	0.00	H
ATOM	397	HD1	HIS	A	224	-4.586	-5.503	3.466	1.00	0.00	H
ATOM	398	HD2	HIS	A	224	-1.156	-7.159	1.703	1.00	0.00	H
ATOM	399	HE1	HIS	A	224	-3.487	-3.716	2.063	1.00	0.00	H
ATOM	400	N	GLY	A	225	-3.818	-11.750	3.267	1.00	0.00	N
ATOM	401	CA	GLY	A	225	-4.357	-12.821	3.988	1.00	0.00	C
ATOM	402	C	GLY	A	225	-3.609	-13.068	5.337	1.00	0.00	C
ATOM	403	O	GLY	A	225	-4.246	-13.249	6.386	1.00	0.00	O
ATOM	404	H	GLY	A	225	-3.382	-11.913	2.371	1.00	0.00	H
ATOM	405	HA2	GLY	A	225	-5.393	-12.548	4.187	1.00	0.00	H
ATOM	406	HA3	GLY	A	225	-4.298	-13.756	3.410	1.00	0.00	H
ATOM	407	HC	GLY	A	225	-2.529	-13.081	5.357	1.00	0.00	H
ATOM	408	N	GLY	A	233	4.110	-8.251	14.074	1.00	0.00	N

ATOM	409	CA	GLY	A	233	5.166	-8.766	13.193	1.00	0.00	C
ATOM	410	C	GLY	A	233	4.989	-8.331	11.729	1.00	0.00	C
ATOM	411	O	GLY	A	233	4.938	-9.090	10.801	1.00	0.00	O
ATOM	412	H1	GLY	A	233	4.578	-7.628	14.692	1.00	0.00	H
ATOM	413	H2	GLY	A	233	3.621	-9.060	14.382	1.00	0.00	H
ATOM	414	HA2	GLY	A	233	6.111	-8.353	13.546	1.00	0.00	H
ATOM	415	HA3	GLY	A	233	5.164	-9.862	13.237	1.00	0.00	H
ATOM	416	N	ILE	A	234	4.871	-6.982	11.448	1.00	0.00	N
ATOM	417	CA	ILE	A	234	4.781	-6.288	10.158	1.00	0.00	C
ATOM	418	CB	ILE	A	234	3.743	-5.115	10.315	1.00	0.00	C
ATOM	419	CG2	ILE	A	234	3.468	-4.348	8.963	1.00	0.00	C
ATOM	420	CG1	ILE	A	234	2.447	-5.425	11.082	1.00	0.00	C
ATOM	421	CD1	ILE	A	234	1.574	-6.364	10.277	1.00	0.00	C
ATOM	422	C	ILE	A	234	6.088	-5.751	9.527	1.00	0.00	C
ATOM	423	O	ILE	A	234	6.616	-4.756	10.085	1.00	0.00	O
ATOM	424	H	ILE	A	234	4.982	-6.357	12.222	1.00	0.00	H
ATOM	425	HA	ILE	A	234	4.391	-7.003	9.453	1.00	0.00	H
ATOM	426	HB	ILE	A	234	4.165	-4.405	11.069	1.00	0.00	H
ATOM	427	HG12	ILE	A	234	1.938	-4.442	11.156	1.00	0.00	H
ATOM	428	HG13	ILE	A	234	2.638	-5.772	12.118	1.00	0.00	H
ATOM	429	HG21	ILE	A	234	4.349	-3.817	8.546	1.00	0.00	H
ATOM	430	HG22	ILE	A	234	2.685	-3.577	9.100	1.00	0.00	H
ATOM	431	HG23	ILE	A	234	3.075	-5.072	8.227	1.00	0.00	H
ATOM	432	HD11	ILE	A	234	1.316	-6.000	9.265	1.00	0.00	H
ATOM	433	HD12	ILE	A	234	0.751	-6.726	10.907	1.00	0.00	H
ATOM	434	HD13	ILE	A	234	2.275	-7.216	10.091	1.00	0.00	H
ATOM	435	N	GLY	A	235	6.658	-6.247	8.493	1.00	0.00	N
ATOM	436	CA	GLY	A	235	7.535	-5.502	7.591	1.00	0.00	C
ATOM	437	C	GLY	A	235	8.795	-4.863	8.075	1.00	0.00	C

ATOM	438	O	GLY	A	235	8.890	-3.643	7.953	1.00	0.00	O
ATOM	439	H	GLY	A	235	6.490	-7.221	8.286	1.00	0.00	H
ATOM	440	HA2	GLY	A	235	7.938	-6.291	6.956	1.00	0.00	H
ATOM	441	HA3	GLY	A	235	6.896	-4.703	7.086	1.00	0.00	H
ATOM	442	HC	GLY	A	235	9.590	-5.448	8.514	1.00	0.00	H
ATOM	443	N	GLY	A	238	11.005	-6.441	4.164	1.00	0.00	N
ATOM	444	CA	GLY	A	238	10.178	-7.078	3.125	1.00	0.00	C
ATOM	445	C	GLY	A	238	9.203	-6.183	2.331	1.00	0.00	C
ATOM	446	O	GLY	A	238	9.140	-6.170	1.152	1.00	0.00	O
ATOM	447	H1	GLY	A	238	11.915	-6.491	3.819	1.00	0.00	H
ATOM	448	H2	GLY	A	238	10.665	-6.801	5.004	1.00	0.00	H
ATOM	449	HA2	GLY	A	238	10.900	-7.414	2.381	1.00	0.00	H
ATOM	450	HA3	GLY	A	238	9.587	-7.898	3.601	1.00	0.00	H
ATOM	451	N	TYR	A	239	8.451	-5.315	2.980	1.00	0.00	N
ATOM	452	CA	TYR	A	239	7.522	-4.269	2.429	1.00	0.00	C
ATOM	453	CB	TYR	A	239	6.186	-4.778	1.911	1.00	0.00	C
ATOM	454	CG	TYR	A	239	5.380	-5.437	3.113	1.00	0.00	C
ATOM	455	CD1	TYR	A	239	4.808	-4.575	4.026	1.00	0.00	C
ATOM	456	CE1	TYR	A	239	3.874	-4.963	5.029	1.00	0.00	C
ATOM	457	CZ	TYR	A	239	3.671	-6.342	5.151	1.00	0.00	C
ATOM	458	OH	TYR	A	239	2.970	-6.850	6.283	1.00	0.00	O
ATOM	459	CD2	TYR	A	239	5.081	-6.823	3.147	1.00	0.00	C
ATOM	460	CE2	TYR	A	239	4.311	-7.232	4.255	1.00	0.00	C
ATOM	461	C	TYR	A	239	8.192	-3.357	1.379	1.00	0.00	C
ATOM	462	O	TYR	A	239	7.817	-3.258	0.213	1.00	0.00	O
ATOM	463	H	TYR	A	239	8.551	-5.379	3.976	1.00	0.00	H
ATOM	464	HA	TYR	A	239	7.323	-3.661	3.302	1.00	0.00	H
ATOM	465	HB2	TYR	A	239	6.424	-5.647	1.240	1.00	0.00	H
ATOM	466	HB3	TYR	A	239	5.606	-3.966	1.432	1.00	0.00	H

ATOM	467	HD1	TYR	A	239	5.149	-3.553	3.841	1.00	0.00	H
ATOM	468	HD2	TYR	A	239	5.472	-7.476	2.396	1.00	0.00	H
ATOM	469	HE1	TYR	A	239	3.449	-4.234	5.694	1.00	0.00	H
ATOM	470	HE2	TYR	A	239	4.067	-8.274	4.426	1.00	0.00	H
ATOM	471	HH	TYR	A	239	2.957	-7.813	6.198	1.00	0.00	H
ATOM	472	N	GLY	A	240	9.266	-2.724	1.768	1.00	0.00	N
ATOM	473	CA	GLY	A	240	10.082	-1.913	0.900	1.00	0.00	C
ATOM	474	C	GLY	A	240	10.765	-2.590	-0.292	1.00	0.00	C
ATOM	475	O	GLY	A	240	10.735	-2.197	-1.492	1.00	0.00	O
ATOM	476	H	GLY	A	240	9.542	-2.808	2.736	1.00	0.00	H
ATOM	477	HA2	GLY	A	240	9.364	-1.229	0.447	1.00	0.00	H
ATOM	478	HA3	GLY	A	240	10.880	-1.415	1.515	1.00	0.00	H
ATOM	479	HC	GLY	A	240	11.326	-3.490	-0.087	1.00	0.00	H
TER	480		GLY	A	240						
HETATM	481	C1	HAL	B	274	0.439	-1.293	1.320	1.00	0.00	C
HETATM	482	C2	HAL	B	274	1.053	-1.828	-0.006	1.00	0.00	C
HETATM	483	N	HAL	B	274	0.037	-1.989	-1.076	1.00	0.00	N1+
HETATM	484	C3	HAL	B	274	-2.340	-2.599	-1.641	1.00	0.00	C
HETATM	485	C4	HAL	B	274	-1.905	-2.707	-3.140	1.00	0.00	C
HETATM	486	C5	HAL	B	274	-1.105	-2.912	-0.771	1.00	0.00	C
HETATM	487	C6	HAL	B	274	-0.629	-1.921	-3.454	1.00	0.00	C
HETATM	488	C7	HAL	B	274	0.506	-2.184	-2.469	1.00	0.00	C
HETATM	489	O1	HAL	B	274	-1.505	-4.078	-3.330	1.00	0.00	O
HETATM	490	C8	HAL	B	274	-3.009	-2.277	-4.071	1.00	0.00	C
HETATM	491	C9	HAL	B	274	-3.483	-3.074	-5.147	1.00	0.00	C
HETATM	492	C10	HAL	B	274	-3.670	-1.019	-3.776	1.00	0.00	C
HETATM	493	C11	HAL	B	274	-4.589	-2.639	-5.878	1.00	0.00	C
HETATM	494	C12	HAL	B	274	-4.735	-0.636	-4.600	1.00	0.00	C
HETATM	495	C13	HAL	B	274	-5.161	-1.369	-5.665	1.00	0.00	C

HETATM	496	CL	HAL	B	274	-6.442	-0.797	-6.667	1.00	0.00	CL
HETATM	497	C14	HAL	B	274	1.631	-1.060	2.326	1.00	0.00	C
HETATM	498	C15	HAL	B	274	1.194	-0.667	3.692	1.00	0.00	C
HETATM	499	O2	HAL	B	274	0.074	-0.199	3.833	1.00	0.00	O
HETATM	500	C16	HAL	B	274	2.095	-0.747	4.870	1.00	0.00	C
HETATM	501	C17	HAL	B	274	3.451	-0.820	4.731	1.00	0.00	C
HETATM	502	C18	HAL	B	274	1.463	-0.600	6.097	1.00	0.00	C
HETATM	503	C19	HAL	B	274	4.274	-0.880	5.903	1.00	0.00	C
HETATM	504	C20	HAL	B	274	2.266	-0.760	7.326	1.00	0.00	C
HETATM	505	C21	HAL	B	274	3.694	-0.901	7.143	1.00	0.00	C
HETATM	506	F	HAL	B	274	4.409	-0.987	8.217	1.00	0.00	F
HETATM	507	H	HAL	B	274	-1.218	-4.171	-4.243	1.00	0.00	H
HETATM	508	HC2	HAL	B	274	-0.300	-1.935	1.859	1.00	0.00	H
HETATM	509	HC3	HAL	B	274	-0.123	-0.350	1.194	1.00	0.00	H
HETATM	510	HC4	HAL	B	274	1.458	-2.836	0.194	1.00	0.00	H
HETATM	511	HC5	HAL	B	274	1.870	-1.180	-0.328	1.00	0.00	H
HETATM	512	HN	HAL	B	274	-0.336	-1.009	-1.128	1.00	0.00	H
HETATM	513	HC6	HAL	B	274	-3.141	-3.290	-1.281	1.00	0.00	H
HETATM	514	HC7	HAL	B	274	-2.656	-1.560	-1.423	1.00	0.00	H
HETATM	515	HC8	HAL	B	274	-1.384	-2.792	0.283	1.00	0.00	H
HETATM	516	HC9	HAL	B	274	-0.648	-3.887	-0.932	1.00	0.00	H
HETATM	517	HC10	HAL	B	274	-0.898	-0.842	-3.456	1.00	0.00	H
HETATM	518	HC11	HAL	B	274	-0.337	-2.203	-4.482	1.00	0.00	H
HETATM	519	HC12	HAL	B	274	0.877	-3.221	-2.569	1.00	0.00	H
HETATM	520	HC13	HAL	B	274	1.334	-1.490	-2.650	1.00	0.00	H
HETATM	521	HC	HAL	B	274	-2.982	-4.004	-5.328	1.00	0.00	H
HETATM	522	HC1	HAL	B	274	-3.198	-0.395	-3.035	1.00	0.00	H
HETATM	523	HC14	HAL	B	274	-4.856	-3.295	-6.687	1.00	0.00	H
HETATM	524	HC15	HAL	B	274	-5.289	0.280	-4.456	1.00	0.00	H

HETATM	525	HC16	HAL	B	274	2.388	-0.314	1.979	1.00	0.00	H
HETATM	526	HC17	HAL	B	274	2.174	-2.003	2.493	1.00	0.00	H
HETATM	527	HC18	HAL	B	274	3.921	-0.792	3.755	1.00	0.00	H
HETATM	528	HC19	HAL	B	274	0.389	-0.589	6.175	1.00	0.00	H
HETATM	529	HC20	HAL	B	274	5.340	-0.870	5.826	1.00	0.00	H
HETATM	530	HC21	HAL	B	274	1.865	-0.897	8.318	1.00	0.00	H
HETATM	531	OH2	TIP	X6377		-0.639	0.754	-1.422	1.00	0.00	O
HETATM	532	H1	TIP	X6377		-0.449	1.114	-2.290	1.00	0.00	H
HETATM	533	H2	TIP	X6377		-0.905	1.508	-0.893	1.00	0.00	H
HETATM	534	OH2	TIP	X8206		7.275	0.646	8.211	1.00	0.00	O
HETATM	535	H1	TIP	X8206		7.875	1.376	8.019	1.00	0.00	H
HETATM	536	H2	TIP	X8206		6.531	1.045	8.637	1.00	0.00	H
HETATM	537	OH2	TIP	X8259		3.736	2.273	6.374	1.00	0.00	O
HETATM	538	H1	TIP	X8259		3.924	2.970	5.755	1.00	0.00	H
HETATM	539	H2	TIP	X8259		3.336	1.585	5.849	1.00	0.00	H
HETATM	540	OH2	TIP	X	419	3.850	3.297	3.604	1.00	0.00	O
HETATM	541	H1	TIP	X	419	4.283	4.141	3.711	1.00	0.00	H
HETATM	542	H2	TIP	X	419	3.039	3.502	3.107	1.00	0.00	H
HETATM	543	OH2	TIP	X5829		1.716	0.042	-5.205	1.00	0.00	O
HETATM	544	H1	TIP	X5829		1.017	-0.482	-5.595	1.00	0.00	H
HETATM	545	H2	TIP	X5829		1.307	0.891	-5.009	1.00	0.00	H
HETATM	546	OH2	TIP	X5939		7.152	2.981	4.793	1.00	0.00	O
HETATM	547	H1	TIP	X5939		7.385	2.061	4.670	1.00	0.00	H
HETATM	548	H2	TIP	X5939		7.504	3.411	4.011	1.00	0.00	H
HETATM	549	OH2	TIP	X9200		4.721	0.984	1.672	1.00	0.00	O
HETATM	550	H1	TIP	X9200		4.358	1.336	0.863	1.00	0.00	H
HETATM	551	H2	TIP	X9200		4.488	1.631	2.337	1.00	0.00	H
HETATM	552	OH2	TIP	X9662		4.248	-1.222	0.099	1.00	0.00	O
HETATM	553	H1	TIP	X9662		4.916	-0.983	-0.558	1.00	0.00	H

HETATM	554	H2	TIP	X9662	3.984	-0.405	0.490	1.00	0.00	H
HETATM	555	OH2	TIP	X 730	5.868	-1.336	2.262	1.00	0.00	O
HETATM	556	H1	TIP	X 730	5.764	-0.387	2.230	1.00	0.00	H
HETATM	557	H2	TIP	X 730	5.400	-1.648	1.490	1.00	0.00	H
HETATM	558	OH2	TIP	X3644	-0.160	-4.761	5.084	1.00	0.00	O
HETATM	559	H1	TIP	X3644	-0.610	-5.435	5.603	1.00	0.00	H
HETATM	560	H2	TIP	X3644	-0.474	-3.927	5.437	1.00	0.00	H
HETATM	561	OH2	TIP	X6744	3.486	0.925	10.801	1.00	0.00	O
HETATM	562	H1	TIP	X6744	3.829	0.040	10.817	1.00	0.00	H
HETATM	563	H2	TIP	X6744	3.174	1.072	11.697	1.00	0.00	H
HETATM	564	OH2	TIP	X7837	-0.322	2.466	4.217	1.00	0.00	O
HETATM	565	H1	TIP	X7837	-0.001	1.563	4.232	1.00	0.00	H
HETATM	566	H2	TIP	X7837	-0.578	2.657	5.122	1.00	0.00	H
HETATM	567	OH2	TIP	X 983	7.186	-1.823	6.938	1.00	0.00	O
HETATM	568	H1	TIP	X 983	6.939	-1.082	7.487	1.00	0.00	H
HETATM	569	H2	TIP	X 983	7.277	-2.552	7.547	1.00	0.00	H
HETATM	570	OH2	TIP	X3979	-9.132	-0.076	-2.441	1.00	0.00	O
HETATM	571	H1	TIP	X3979	-9.735	-0.364	-3.118	1.00	0.00	H
HETATM	572	H2	TIP	X3979	-8.329	-0.578	-2.592	1.00	0.00	H
HETATM	573	OH2	TIP	X6055	7.526	-2.540	11.182	1.00	0.00	O
HETATM	574	H1	TIP	X6055	7.941	-2.947	11.948	1.00	0.00	H
HETATM	575	H2	TIP	X6055	7.389	-3.276	10.587	1.00	0.00	H
HETATM	576	OH2	TIP	X7729	0.623	-4.494	2.582	1.00	0.00	O
HETATM	577	H1	TIP	X7729	-0.231	-4.833	2.332	1.00	0.00	H
HETATM	578	H2	TIP	X7729	0.492	-4.064	3.428	1.00	0.00	H
HETATM	579	OH2	TIP	X8254	1.463	3.558	2.002	1.00	0.00	O
HETATM	580	H1	TIP	X8254	0.568	3.542	2.352	1.00	0.00	H
HETATM	581	H2	TIP	X8254	1.363	3.429	1.071	1.00	0.00	H
HETATM	582	OH2	TIP	X2059	7.484	0.185	4.718	1.00	0.00	O

```
HETATM  583  H1  TIP X2059       7.138  -0.559   4.220  1.00  0.00           H
HETATM  584  H2  TIP X2059       7.532  -0.132   5.619  1.00  0.00           H
HETATM  585  OH2 TIP X4692       3.176   1.839  -0.234  1.00  0.00           O
HETATM  586  H1  TIP X4692       3.438   2.120  -1.107  1.00  0.00           H
HETATM  587  H2  TIP X4692       2.322   2.246  -0.094  1.00  0.00           H
HETATM  588  OH2 TIP X5919       4.812   2.235   8.858  1.00  0.00           O
HETATM  589  H1  TIP X5919       4.395   1.576   9.406  1.00  0.00           H
HETATM  590  H2  TIP X5919       4.391   2.171   8.020  1.00  0.00           H
HETATM  591  OH2 TIP X9531       4.898  -1.436  11.872  1.00  0.00           O
HETATM  592  H1  TIP X9531       4.426  -1.761  12.641  1.00  0.00           H
HETATM  593  H2  TIP X9531       5.745  -1.877  11.911  1.00  0.00           H
END
```

Haloperidol (crystal)

```
ATOM      1  N   GLY A  56      -3.511 -23.889  10.936  1.00  0.00           N1+
ATOM      2  CA  GLY A  56      -2.964 -23.286  12.187  1.00  0.00           C
ATOM      3  C   GLY A  56      -3.843 -22.000  12.660  1.00  0.00           C
ATOM      4  O   GLY A  56      -3.984 -21.691  13.822  1.00  0.00           O
ATOM      5  H1  GLY A  56      -3.816 -24.777  11.236  1.00  0.00           H
ATOM      6  H2  GLY A  56      -2.792 -23.726  10.282  1.00  0.00           H
ATOM      7  HA2 GLY A  56      -2.993 -24.059  12.954  1.00  0.00           H
ATOM      8  HA3 GLY A  56      -1.957 -22.937  12.019  1.00  0.00           H
ATOM      9  N   VAL A  57      -4.456 -21.254  11.703  1.00  0.00           N
ATOM     10  CA  VAL A  57      -5.469 -20.325  12.126  1.00  0.00           C
ATOM     11  CB  VAL A  57      -5.929 -19.310  11.039  1.00  0.00           C
ATOM     12  CG1 VAL A  57      -6.546 -18.120  11.848  1.00  0.00           C
ATOM     13  CG2 VAL A  57      -4.756 -18.754  10.253  1.00  0.00           C
ATOM     14  C   VAL A  57      -6.648 -21.096  12.679  1.00  0.00           C
ATOM     15  O   VAL A  57      -7.241 -20.707  13.649  1.00  0.00           O
```

```
ATOM     16  H   VAL A  57      -4.163 -21.401  10.758  1.00  0.00           H
ATOM     17  HA  VAL A  57      -4.992 -19.737  12.892  1.00  0.00           H
ATOM     18  HB  VAL A  57      -6.591 -19.827  10.292  1.00  0.00           H
ATOM     19 HG11 VAL A  57      -7.093 -17.372  11.236  1.00  0.00           H
ATOM     20 HG12 VAL A  57      -5.701 -17.504  12.227  1.00  0.00           H
ATOM     21 HG13 VAL A  57      -7.175 -18.372  12.718  1.00  0.00           H
ATOM     22 HG21 VAL A  57      -4.377 -19.392   9.419  1.00  0.00           H
ATOM     23 HG22 VAL A  57      -4.003 -18.331  10.953  1.00  0.00           H
ATOM     24 HG23 VAL A  57      -4.977 -17.833   9.666  1.00  0.00           H
ATOM     25  N   GLY A  58      -7.167 -22.200  12.040  1.00  0.00           N
ATOM     26  CA  GLY A  58      -8.137 -23.076  12.745  1.00  0.00           C
ATOM     27  C   GLY A  58      -7.792 -23.431  14.130  1.00  0.00           C
ATOM     28  O   GLY A  58      -8.501 -23.095  15.046  1.00  0.00           O
ATOM     29  H   GLY A  58      -6.888 -22.414  11.093  1.00  0.00           H
ATOM     30  HA2 GLY A  58      -9.063 -22.510  12.845  1.00  0.00           H
ATOM     31  HA3 GLY A  58      -8.254 -23.999  12.130  1.00  0.00           H
ATOM     32  N   GLY A  59      -6.614 -24.023  14.349  1.00  0.00           N
ATOM     33  CA  GLY A  59      -6.076 -24.329  15.699  1.00  0.00           C
ATOM     34  C   GLY A  59      -5.980 -23.114  16.695  1.00  0.00           C
ATOM     35  O   GLY A  59      -6.481 -23.213  17.826  1.00  0.00           O
ATOM     36  H   GLY A  59      -6.055 -24.278  13.547  1.00  0.00           H
ATOM     37  HA2 GLY A  59      -6.796 -25.020  16.138  1.00  0.00           H
ATOM     38  HA3 GLY A  59      -5.064 -24.757  15.589  1.00  0.00           H
ATOM     39  N   LEU A  60      -5.451 -21.930  16.261  1.00  0.00           N
ATOM     40  CA  LEU A  60      -5.578 -20.721  17.012  1.00  0.00           C
ATOM     41  CB  LEU A  60      -4.889 -19.661  16.222  1.00  0.00           C
ATOM     42  CG  LEU A  60      -4.516 -18.237  16.804  1.00  0.00           C
ATOM     43  CD1 LEU A  60      -3.610 -18.387  18.044  1.00  0.00           C
ATOM     44  CD2 LEU A  60      -3.559 -17.440  15.862  1.00  0.00           C
```

ATOM	45	C	LEU	A	60	-6.946	-20.284	17.437	1.00	0.00	C
ATOM	46	O	LEU	A	60	-7.142	-19.739	18.515	1.00	0.00	O
ATOM	47	H	LEU	A	60	-5.196	-21.889	15.303	1.00	0.00	H
ATOM	48	HA	LEU	A	60	-4.999	-20.831	17.916	1.00	0.00	H
ATOM	49	HB2	LEU	A	60	-5.388	-19.533	15.237	1.00	0.00	H
ATOM	50	HB3	LEU	A	60	-3.891	-20.095	16.002	1.00	0.00	H
ATOM	51	HG	LEU	A	60	-5.442	-17.660	16.989	1.00	0.00	H
ATOM	52	HD11	LEU	A	60	-2.629	-18.859	17.838	1.00	0.00	H
ATOM	53	HD12	LEU	A	60	-4.062	-18.968	18.867	1.00	0.00	H
ATOM	54	HD13	LEU	A	60	-3.394	-17.330	18.297	1.00	0.00	H
ATOM	55	HD21	LEU	A	60	-3.878	-17.332	14.807	1.00	0.00	H
ATOM	56	HD22	LEU	A	60	-2.497	-17.777	15.903	1.00	0.00	H
ATOM	57	HD23	LEU	A	60	-3.615	-16.418	16.310	1.00	0.00	H
ATOM	58	N	GLY	A	61	-7.972	-20.455	16.516	1.00	0.00	N
ATOM	59	CA	GLY	A	61	-9.455	-20.273	16.834	1.00	0.00	C
ATOM	60	C	GLY	A	61	-10.007	-21.326	17.779	1.00	0.00	C
ATOM	61	O	GLY	A	61	-10.843	-21.004	18.670	1.00	0.00	O
ATOM	62	H	GLY	A	61	-7.721	-20.717	15.574	1.00	0.00	H
ATOM	63	HA2	GLY	A	61	-9.583	-19.321	17.350	1.00	0.00	H
ATOM	64	HA3	GLY	A	61	-9.997	-20.303	15.882	1.00	0.00	H
ATOM	65	HC	GLY	A	61	-9.684	-22.353	17.689	1.00	0.00	H
ATOM	66	N	GLY	A	65	-6.772	-18.523	22.140	1.00	0.00	N1+
ATOM	67	CA	GLY	A	65	-5.773	-17.638	22.570	1.00	0.00	C
ATOM	68	C	GLY	A	65	-4.421	-18.134	22.019	1.00	0.00	C
ATOM	69	O	GLY	A	65	-4.321	-19.310	21.633	1.00	0.00	O
ATOM	70	H1	GLY	A	65	-7.409	-17.972	21.549	1.00	0.00	H
ATOM	71	H2	GLY	A	65	-7.175	-18.937	22.991	1.00	0.00	H
ATOM	72	HA2	GLY	A	65	-6.015	-16.637	22.213	1.00	0.00	H
ATOM	73	HA3	GLY	A	65	-5.706	-17.619	23.652	1.00	0.00	H

ATOM	74	N	TRP	A	66	-3.325	-17.351	22.079	1.00	0.00	N
ATOM	75	CA	TRP	A	66	-1.962	-17.758	21.856	1.00	0.00	C
ATOM	76	CB	TRP	A	66	-1.099	-16.539	21.361	1.00	0.00	C
ATOM	77	CG	TRP	A	66	0.365	-16.739	21.097	1.00	0.00	C
ATOM	78	CD1	TRP	A	66	0.901	-17.660	20.256	1.00	0.00	C
ATOM	79	NE1	TRP	A	66	2.223	-17.785	20.499	1.00	0.00	N
ATOM	80	CE2	TRP	A	66	2.594	-16.916	21.477	1.00	0.00	C
ATOM	81	CD2	TRP	A	66	1.419	-16.291	21.942	1.00	0.00	C
ATOM	82	CE3	TRP	A	66	1.467	-15.434	23.039	1.00	0.00	C
ATOM	83	CZ3	TRP	A	66	2.714	-15.225	23.640	1.00	0.00	C
ATOM	84	CZ2	TRP	A	66	3.826	-16.499	21.890	1.00	0.00	C
ATOM	85	CH2	TRP	A	66	3.876	-15.716	23.040	1.00	0.00	C
ATOM	86	C	TRP	A	66	-1.308	-18.554	22.981	1.00	0.00	C
ATOM	87	O	TRP	A	66	-0.978	-17.998	24.007	1.00	0.00	O
ATOM	88	H	TRP	A	66	-3.421	-16.396	22.363	1.00	0.00	H
ATOM	89	HA	TRP	A	66	-2.034	-18.401	20.994	1.00	0.00	H
ATOM	90	HB2	TRP	A	66	-1.251	-15.711	22.090	1.00	0.00	H
ATOM	91	HB3	TRP	A	66	-1.510	-16.304	20.361	1.00	0.00	H
ATOM	92	HD1	TRP	A	66	0.362	-18.196	19.485	1.00	0.00	H
ATOM	93	HE1	TRP	A	66	2.691	-18.636	20.389	1.00	0.00	H
ATOM	94	HE3	TRP	A	66	0.571	-14.942	23.386	1.00	0.00	H
ATOM	95	HZ2	TRP	A	66	4.737	-16.891	21.446	1.00	0.00	H
ATOM	96	HZ3	TRP	A	66	2.848	-14.507	24.441	1.00	0.00	H
ATOM	97	HH2	TRP	A	66	4.849	-15.468	23.460	1.00	0.00	H
ATOM	98	N	GLY	A	67	-1.066	-19.849	22.775	1.00	0.00	N
ATOM	99	CA	GLY	A	67	-0.318	-20.705	23.720	1.00	0.00	C
ATOM	100	C	GLY	A	67	0.939	-21.439	23.251	1.00	0.00	C
ATOM	101	O	GLY	A	67	1.720	-21.858	24.104	1.00	0.00	O
ATOM	102	H	GLY	A	67	-1.414	-20.269	21.925	1.00	0.00	H

ATOM	103	HA2	GLY	A	67	0.082	-19.989	24.438	1.00	0.00	H
ATOM	104	HA3	GLY	A	67	-1.038	-21.468	24.133	1.00	0.00	H
ATOM	105	N	PHE	A	68	1.336	-21.591	21.941	1.00	0.00	N
ATOM	106	CA	PHE	A	68	2.555	-22.082	21.536	1.00	0.00	C
ATOM	107	CB	PHE	A	68	2.649	-22.579	20.064	1.00	0.00	C
ATOM	108	CG	PHE	A	68	1.863	-21.715	19.103	1.00	0.00	C
ATOM	109	CD1	PHE	A	68	2.521	-20.697	18.288	1.00	0.00	C
ATOM	110	CE1	PHE	A	68	1.835	-19.913	17.378	1.00	0.00	C
ATOM	111	CZ	PHE	A	68	0.497	-20.092	17.150	1.00	0.00	C
ATOM	112	CD2	PHE	A	68	0.522	-21.852	18.883	1.00	0.00	C
ATOM	113	CE2	PHE	A	68	-0.166	-21.048	18.013	1.00	0.00	C
ATOM	114	C	PHE	A	68	3.766	-21.141	21.848	1.00	0.00	C
ATOM	115	O	PHE	A	68	3.626	-19.936	21.925	1.00	0.00	O
ATOM	116	H	PHE	A	68	0.709	-21.308	21.227	1.00	0.00	H
ATOM	117	HA	PHE	A	68	2.808	-22.938	22.136	1.00	0.00	H
ATOM	118	HB2	PHE	A	68	2.218	-23.587	19.945	1.00	0.00	H
ATOM	119	HB3	PHE	A	68	3.713	-22.637	19.760	1.00	0.00	H
ATOM	120	HD1	PHE	A	68	3.563	-20.498	18.449	1.00	0.00	H
ATOM	121	HD2	PHE	A	68	-0.047	-22.592	19.434	1.00	0.00	H
ATOM	122	HE1	PHE	A	68	2.292	-18.990	17.054	1.00	0.00	H
ATOM	123	HE2	PHE	A	68	-1.226	-21.254	17.944	1.00	0.00	H
ATOM	124	HZ	PHE	A	68	-0.208	-19.551	16.555	1.00	0.00	H
ATOM	125	N	GLY	A	69	4.954	-21.623	22.041	1.00	0.00	N
ATOM	126	CA	GLY	A	69	6.160	-20.832	22.310	1.00	0.00	C
ATOM	127	C	GLY	A	69	6.576	-19.828	21.252	1.00	0.00	C
ATOM	128	O	GLY	A	69	6.302	-20.044	20.044	1.00	0.00	O
ATOM	129	H	GLY	A	69	5.058	-22.627	22.003	1.00	0.00	H
ATOM	130	HA2	GLY	A	69	5.908	-20.221	23.177	1.00	0.00	H
ATOM	131	HA3	GLY	A	69	7.007	-21.539	22.485	1.00	0.00	H

ATOM	132	HC	GLY	A	69	7.106	-18.931	21.535	1.00	0.00	H
ATOM	133	N	GLY	A	72	8.157	-20.620	17.643	1.00	0.00	N1+
ATOM	134	CA	GLY	A	72	7.163	-21.131	16.673	1.00	0.00	C
ATOM	135	C	GLY	A	72	6.223	-20.057	16.174	1.00	0.00	C
ATOM	136	O	GLY	A	72	5.664	-20.132	15.074	1.00	0.00	O
ATOM	137	H1	GLY	A	72	9.034	-20.724	17.184	1.00	0.00	H
ATOM	138	H2	GLY	A	72	7.933	-21.077	18.498	1.00	0.00	H
ATOM	139	HA2	GLY	A	72	7.703	-21.505	15.803	1.00	0.00	H
ATOM	140	HA3	GLY	A	72	6.584	-21.927	17.160	1.00	0.00	H
ATOM	141	N	CYS	A	73	5.953	-19.071	17.011	1.00	0.00	N
ATOM	142	CA	CYS	A	73	5.098	-17.945	16.661	1.00	0.00	C
ATOM	143	CB	CYS	A	73	4.735	-17.132	17.940	1.00	0.00	C
ATOM	144	SG	CYS	A	73	3.512	-15.768	17.563	1.00	0.00	S
ATOM	145	C	CYS	A	73	5.747	-17.122	15.644	1.00	0.00	C
ATOM	146	O	CYS	A	73	5.250	-16.813	14.530	1.00	0.00	O
ATOM	147	H	CYS	A	73	6.312	-19.133	17.939	1.00	0.00	H
ATOM	148	HA	CYS	A	73	4.228	-18.315	16.114	1.00	0.00	H
ATOM	149	HB2	CYS	A	73	5.585	-16.652	18.465	1.00	0.00	H
ATOM	150	HB3	CYS	A	73	4.240	-17.718	18.738	1.00	0.00	H
ATOM	151	N	GLY	A	74	6.923	-16.672	15.880	1.00	0.00	N
ATOM	152	CA	GLY	A	74	7.634	-15.773	14.983	1.00	0.00	C
ATOM	153	C	GLY	A	74	7.815	-16.302	13.594	1.00	0.00	C
ATOM	154	O	GLY	A	74	7.694	-15.621	12.531	1.00	0.00	O
ATOM	155	H	GLY	A	74	7.381	-16.956	16.734	1.00	0.00	H
ATOM	156	HA2	GLY	A	74	7.021	-14.877	14.884	1.00	0.00	H
ATOM	157	HA3	GLY	A	74	8.628	-15.552	15.423	1.00	0.00	H
ATOM	158	N	GLY	A	75	8.168	-17.610	13.466	1.00	0.00	N
ATOM	159	CA	GLY	A	75	8.250	-18.356	12.197	1.00	0.00	C
ATOM	160	C	GLY	A	75	6.912	-18.459	11.451	1.00	0.00	C

ATOM	161	O	GLY	A	75	6.787	-18.344	10.252	1.00	0.00	O
ATOM	162	H	GLY	A	75	8.393	-18.113	14.313	1.00	0.00	H
ATOM	163	HA2	GLY	A	75	8.929	-17.802	11.549	1.00	0.00	H
ATOM	164	HA3	GLY	A	75	8.616	-19.372	12.408	1.00	0.00	H
ATOM	165	N	PHE	A	76	5.867	-18.757	12.211	1.00	0.00	N
ATOM	166	CA	PHE	A	76	4.506	-18.848	11.730	1.00	0.00	C
ATOM	167	CB	PHE	A	76	3.460	-19.294	12.808	1.00	0.00	C
ATOM	168	CG	PHE	A	76	2.009	-19.269	12.454	1.00	0.00	C
ATOM	169	CD1	PHE	A	76	1.664	-20.022	11.336	1.00	0.00	C
ATOM	170	CE1	PHE	A	76	0.373	-20.290	11.056	1.00	0.00	C
ATOM	171	CZ	PHE	A	76	-0.675	-19.779	11.842	1.00	0.00	C
ATOM	172	CD2	PHE	A	76	1.013	-18.825	13.358	1.00	0.00	C
ATOM	173	CE2	PHE	A	76	-0.325	-19.089	13.063	1.00	0.00	C
ATOM	174	C	PHE	A	76	4.051	-17.586	11.045	1.00	0.00	C
ATOM	175	O	PHE	A	76	3.517	-17.493	9.945	1.00	0.00	O
ATOM	176	H	PHE	A	76	5.999	-18.912	13.190	1.00	0.00	H
ATOM	177	HA	PHE	A	76	4.509	-19.516	10.881	1.00	0.00	H
ATOM	178	HB2	PHE	A	76	3.718	-18.797	13.775	1.00	0.00	H
ATOM	179	HB3	PHE	A	76	3.662	-20.391	12.850	1.00	0.00	H
ATOM	180	HD1	PHE	A	76	2.441	-20.387	10.686	1.00	0.00	H
ATOM	181	HD2	PHE	A	76	1.342	-18.291	14.234	1.00	0.00	H
ATOM	182	HE1	PHE	A	76	0.174	-20.884	10.187	1.00	0.00	H
ATOM	183	HE2	PHE	A	76	-1.072	-18.809	13.783	1.00	0.00	H
ATOM	184	HZ	PHE	A	76	-1.713	-19.955	11.619	1.00	0.00	H
ATOM	185	N	VAL	A	77	4.285	-16.459	11.707	1.00	0.00	N
ATOM	186	CA	VAL	A	77	4.073	-15.078	11.311	1.00	0.00	C
ATOM	187	CB	VAL	A	77	4.344	-14.118	12.439	1.00	0.00	C
ATOM	188	CG1	VAL	A	77	4.277	-12.641	12.032	1.00	0.00	C
ATOM	189	CG2	VAL	A	77	3.359	-14.329	13.651	1.00	0.00	C

```
ATOM    190  C   VAL A  77       4.830 -14.672  10.047  1.00  0.00           C
ATOM    191  O   VAL A  77       4.373 -13.954   9.143  1.00  0.00           O
ATOM    192  H   VAL A  77       4.597 -16.495  12.659  1.00  0.00           H
ATOM    193  HA  VAL A  77       3.052 -14.885  11.020  1.00  0.00           H
ATOM    194  HB  VAL A  77       5.340 -14.341  12.865  1.00  0.00           H
ATOM    195 HG11 VAL A  77       4.993 -12.432  11.214  1.00  0.00           H
ATOM    196 HG12 VAL A  77       4.472 -11.996  12.930  1.00  0.00           H
ATOM    197 HG13 VAL A  77       3.253 -12.365  11.707  1.00  0.00           H
ATOM    198 HG21 VAL A  77       3.775 -13.774  14.516  1.00  0.00           H
ATOM    199 HG22 VAL A  77       3.207 -15.375  13.995  1.00  0.00           H
ATOM    200 HG23 VAL A  77       2.319 -14.048  13.379  1.00  0.00           H
ATOM    201  N   GLY A  78       6.026 -15.194   9.948  1.00  0.00           N
ATOM    202  CA  GLY A  78       6.906 -15.180   8.776  1.00  0.00           C
ATOM    203  C   GLY A  78       6.350 -15.967   7.594  1.00  0.00           C
ATOM    204  O   GLY A  78       6.517 -15.543   6.459  1.00  0.00           O
ATOM    205  H   GLY A  78       6.382 -15.658  10.771  1.00  0.00           H
ATOM    206  HA2 GLY A  78       6.996 -14.142   8.454  1.00  0.00           H
ATOM    207  HA3 GLY A  78       7.881 -15.604   9.060  1.00  0.00           H
ATOM    208  N   GLY A  79       5.653 -17.105   7.865  1.00  0.00           N
ATOM    209  CA  GLY A  79       5.068 -17.914   6.889  1.00  0.00           C
ATOM    210  C   GLY A  79       3.874 -17.284   6.247  1.00  0.00           C
ATOM    211  O   GLY A  79       3.700 -17.240   5.056  1.00  0.00           O
ATOM    212  H   GLY A  79       5.553 -17.381   8.831  1.00  0.00           H
ATOM    213  HA2 GLY A  79       5.824 -18.062   6.118  1.00  0.00           H
ATOM    214  HA3 GLY A  79       4.759 -18.882   7.334  1.00  0.00           H
ATOM    215  N   ASP A  80       3.010 -16.674   7.078  1.00  0.00           N
ATOM    216  CA  ASP A  80       1.961 -15.832   6.743  1.00  0.00           C
ATOM    217  CB  ASP A  80       1.357 -15.458   8.149  1.00  0.00           C
ATOM    218  CG  ASP A  80       0.283 -14.519   8.135  1.00  0.00           C
```

ATOM	219	OD1	ASP	A	80	-0.131	-14.073	7.046	1.00	0.00	O
ATOM	220	OD2	ASP	A	80	-0.211	-14.211	9.218	1.00	0.00	O1-
ATOM	221	C	ASP	A	80	2.404	-14.605	5.854	1.00	0.00	C
ATOM	222	O	ASP	A	80	1.918	-14.504	4.728	1.00	0.00	O
ATOM	223	H	ASP	A	80	3.142	-16.797	8.056	1.00	0.00	H
ATOM	224	HA	ASP	A	80	1.373	-16.365	6.018	1.00	0.00	H
ATOM	225	HB2	ASP	A	80	2.124	-14.934	8.742	1.00	0.00	H
ATOM	226	HB3	ASP	A	80	1.185	-16.330	8.820	1.00	0.00	H
ATOM	227	N	VAL	A	81	3.434	-13.878	6.237	1.00	0.00	N
ATOM	228	CA	VAL	A	81	3.852	-12.597	5.711	1.00	0.00	C
ATOM	229	CB	VAL	A	81	4.683	-11.848	6.762	1.00	0.00	C
ATOM	230	CG1	VAL	A	81	6.125	-12.142	6.796	1.00	0.00	C
ATOM	231	CG2	VAL	A	81	4.574	-10.312	6.438	1.00	0.00	C
ATOM	232	C	VAL	A	81	4.404	-12.799	4.305	1.00	0.00	C
ATOM	233	O	VAL	A	81	4.233	-12.019	3.375	1.00	0.00	O
ATOM	234	H	VAL	A	81	3.849	-14.207	7.089	1.00	0.00	H
ATOM	235	HA	VAL	A	81	2.979	-11.978	5.526	1.00	0.00	H
ATOM	236	HB	VAL	A	81	4.122	-11.873	7.715	1.00	0.00	H
ATOM	237	HG11	VAL	A	81	6.197	-13.233	6.924	1.00	0.00	H
ATOM	238	HG12	VAL	A	81	6.721	-11.734	7.636	1.00	0.00	H
ATOM	239	HG13	VAL	A	81	6.758	-11.740	5.959	1.00	0.00	H
ATOM	240	HG21	VAL	A	81	4.938	-9.702	7.307	1.00	0.00	H
ATOM	241	HG22	VAL	A	81	3.500	-10.023	6.341	1.00	0.00	H
ATOM	242	HG23	VAL	A	81	5.155	-10.185	5.508	1.00	0.00	H
ATOM	243	N	GLY	A	82	5.133	-13.917	4.135	1.00	0.00	N
ATOM	244	CA	GLY	A	82	5.700	-14.453	2.891	1.00	0.00	C
ATOM	245	C	GLY	A	82	4.694	-14.649	1.808	1.00	0.00	C
ATOM	246	O	GLY	A	82	4.889	-14.161	0.656	1.00	0.00	O
ATOM	247	H	GLY	A	82	5.313	-14.456	4.970	1.00	0.00	H

ATOM	248	HA2	GLY	A	82	6.411	-13.719	2.510	1.00	0.00	H
ATOM	249	HA3	GLY	A	82	6.189	-15.416	3.123	1.00	0.00	H
ATOM	250	N	GLY	A	83	3.653	-15.508	1.946	1.00	0.00	N
ATOM	251	CA	GLY	A	83	2.598	-15.763	0.972	1.00	0.00	C
ATOM	252	C	GLY	A	83	1.739	-14.554	0.650	1.00	0.00	C
ATOM	253	O	GLY	A	83	1.522	-14.334	-0.543	1.00	0.00	O
ATOM	254	H	GLY	A	83	3.604	-16.024	2.813	1.00	0.00	H
ATOM	255	HA2	GLY	A	83	3.096	-16.042	0.043	1.00	0.00	H
ATOM	256	HA3	GLY	A	83	1.948	-16.571	1.356	1.00	0.00	H
ATOM	257	N	CYS	A	84	1.302	-13.757	1.655	1.00	0.00	N
ATOM	258	CA	CYS	A	84	0.631	-12.521	1.370	1.00	0.00	C
ATOM	259	CB	CYS	A	84	0.027	-11.977	2.678	1.00	0.00	C
ATOM	260	SG	CYS	A	84	-1.087	-13.204	3.444	1.00	0.00	S
ATOM	261	C	CYS	A	84	1.406	-11.450	0.610	1.00	0.00	C
ATOM	262	O	CYS	A	84	0.942	-10.661	-0.145	1.00	0.00	O
ATOM	263	H	CYS	A	84	1.671	-13.815	2.582	1.00	0.00	H
ATOM	264	HA	CYS	A	84	-0.194	-12.737	0.700	1.00	0.00	H
ATOM	265	HB2	CYS	A	84	-0.491	-11.059	2.348	1.00	0.00	H
ATOM	266	HB3	CYS	A	84	0.839	-11.735	3.399	1.00	0.00	H
ATOM	267	HG	CYS	A	84	-0.142	-13.680	4.240	1.00	0.00	H
ATOM	268	N	THR	A	85	2.729	-11.277	0.870	1.00	0.00	N
ATOM	269	CA	THR	A	85	3.626	-10.374	0.093	1.00	0.00	C
ATOM	270	CB	THR	A	85	5.085	-10.588	0.475	1.00	0.00	C
ATOM	271	OG1	THR	A	85	5.291	-9.928	1.743	1.00	0.00	O
ATOM	272	CG2	THR	A	85	5.902	-9.724	-0.534	1.00	0.00	C
ATOM	273	C	THR	A	85	3.534	-10.746	-1.383	1.00	0.00	C
ATOM	274	O	THR	A	85	3.399	-9.911	-2.288	1.00	0.00	O
ATOM	275	H	THR	A	85	3.118	-11.892	1.546	1.00	0.00	H
ATOM	276	HA	THR	A	85	3.279	-9.372	0.233	1.00	0.00	H

ATOM	277	HB	THR	A	85	5.325	-11.671	0.522	1.00	0.00	H
ATOM	278	HG21	THR	A	85	6.009	-10.206	-1.534	1.00	0.00	H
ATOM	279	HG22	THR	A	85	6.950	-9.779	-0.141	1.00	0.00	H
ATOM	280	HG23	THR	A	85	5.573	-8.705	-0.780	1.00	0.00	H
ATOM	281	HG1	THR	A	85	4.920	-10.522	2.406	1.00	0.00	H
ATOM	282	N	GLY	A	86	3.597	-12.065	-1.627	1.00	0.00	N
ATOM	283	CA	GLY	A	86	3.487	-12.603	-2.962	1.00	0.00	C
ATOM	284	C	GLY	A	86	2.103	-12.394	-3.605	1.00	0.00	C
ATOM	285	O	GLY	A	86	2.054	-12.240	-4.845	1.00	0.00	O
ATOM	286	H	GLY	A	86	3.725	-12.700	-0.852	1.00	0.00	H
ATOM	287	HA2	GLY	A	86	4.213	-12.060	-3.566	1.00	0.00	H
ATOM	288	HA3	GLY	A	86	3.696	-13.686	-2.941	1.00	0.00	H
ATOM	289	N	GLY	A	87	0.934	-12.502	-2.848	1.00	0.00	N
ATOM	290	CA	GLY	A	87	-0.347	-12.139	-3.257	1.00	0.00	C
ATOM	291	C	GLY	A	87	-0.403	-10.796	-3.871	1.00	0.00	C
ATOM	292	O	GLY	A	87	-1.078	-10.668	-4.886	1.00	0.00	O
ATOM	293	H	GLY	A	87	1.026	-12.879	-1.916	1.00	0.00	H
ATOM	294	HA2	GLY	A	87	-0.645	-12.860	-4.018	1.00	0.00	H
ATOM	295	HA3	GLY	A	87	-1.041	-12.166	-2.388	1.00	0.00	H
ATOM	296	N	ILE	A	88	0.088	-9.776	-3.266	1.00	0.00	N
ATOM	297	CA	ILE	A	88	0.168	-8.491	-3.903	1.00	0.00	C
ATOM	298	CB	ILE	A	88	0.311	-7.375	-2.828	1.00	0.00	C
ATOM	299	CG2	ILE	A	88	1.679	-7.442	-2.078	1.00	0.00	C
ATOM	300	CG1	ILE	A	88	0.169	-6.021	-3.492	1.00	0.00	C
ATOM	301	CD1	ILE	A	88	-0.106	-4.835	-2.623	1.00	0.00	C
ATOM	302	C	ILE	A	88	1.122	-8.439	-5.055	1.00	0.00	C
ATOM	303	O	ILE	A	88	0.912	-7.844	-6.053	1.00	0.00	O
ATOM	304	H	ILE	A	88	0.272	-9.870	-2.296	1.00	0.00	H
ATOM	305	HA	ILE	A	88	-0.819	-8.318	-4.319	1.00	0.00	H

ATOM	306	HB	ILE	A	88	-0.524	-7.532	-2.109	1.00	0.00	H
ATOM	307	HG12	ILE	A	88	-0.547	-6.132	-4.341	1.00	0.00	H
ATOM	308	HG13	ILE	A	88	1.133	-5.797	-4.018	1.00	0.00	H
ATOM	309	HG21	ILE	A	88	1.824	-8.305	-1.415	1.00	0.00	H
ATOM	310	HG22	ILE	A	88	1.950	-6.492	-1.545	1.00	0.00	H
ATOM	311	HG23	ILE	A	88	2.457	-7.593	-2.859	1.00	0.00	H
ATOM	312	HD11	ILE	A	88	-1.195	-4.604	-2.515	1.00	0.00	H
ATOM	313	HD12	ILE	A	88	0.302	-3.930	-3.121	1.00	0.00	H
ATOM	314	HD13	ILE	A	88	0.373	-4.938	-1.637	1.00	0.00	H
ATOM	315	N	GLY	A	89	2.325	-9.108	-5.027	1.00	0.00	N
ATOM	316	CA	GLY	A	89	3.336	-8.930	-6.050	1.00	0.00	C
ATOM	317	C	GLY	A	89	2.835	-9.664	-7.321	1.00	0.00	C
ATOM	318	O	GLY	A	89	3.014	-9.161	-8.472	1.00	0.00	O
ATOM	319	H	GLY	A	89	2.510	-9.746	-4.266	1.00	0.00	H
ATOM	320	HA2	GLY	A	89	3.502	-7.868	-6.233	1.00	0.00	H
ATOM	321	HA3	GLY	A	89	4.262	-9.377	-5.740	1.00	0.00	H
ATOM	322	HC	GLY	A	89	2.329	-10.613	-7.225	1.00	0.00	H
ATOM	323	N	GLY	A	147	8.002	-12.062	23.442	1.00	0.00	N1+
ATOM	324	CA	GLY	A	147	6.671	-11.844	22.904	1.00	0.00	C
ATOM	325	C	GLY	A	147	6.810	-11.866	21.457	1.00	0.00	C
ATOM	326	O	GLY	A	147	7.864	-11.438	20.963	1.00	0.00	O
ATOM	327	H1	GLY	A	147	7.944	-12.939	23.990	1.00	0.00	H
ATOM	328	H2	GLY	A	147	8.226	-11.212	23.991	1.00	0.00	H
ATOM	329	HA2	GLY	A	147	5.959	-12.603	23.229	1.00	0.00	H
ATOM	330	HA3	GLY	A	147	6.311	-10.883	23.244	1.00	0.00	H
ATOM	331	N	CYS	A	148	5.789	-12.344	20.700	1.00	0.00	N
ATOM	332	CA	CYS	A	148	5.883	-12.585	19.288	1.00	0.00	C
ATOM	333	CB	CYS	A	148	4.691	-13.573	19.066	1.00	0.00	C
ATOM	334	SG	CYS	A	148	4.698	-14.260	17.391	1.00	0.00	S

ATOM	335	C	CYS	A	148	6.102	-11.418	18.327	1.00	0.00	C
ATOM	336	O	CYS	A	148	5.692	-10.301	18.631	1.00	0.00	O
ATOM	337	H	CYS	A	148	4.883	-12.473	21.114	1.00	0.00	H
ATOM	338	HA	CYS	A	148	6.779	-13.184	19.138	1.00	0.00	H
ATOM	339	HB2	CYS	A	148	3.770	-12.951	19.088	1.00	0.00	H
ATOM	340	HB3	CYS	A	148	4.631	-14.405	19.803	1.00	0.00	H
ATOM	341	N	GLY	A	149	6.843	-11.711	17.204	1.00	0.00	N
ATOM	342	CA	GLY	A	149	7.150	-10.725	16.160	1.00	0.00	C
ATOM	343	C	GLY	A	149	6.054	-9.932	15.580	1.00	0.00	C
ATOM	344	O	GLY	A	149	6.191	-8.806	15.175	1.00	0.00	O
ATOM	345	H	GLY	A	149	7.193	-12.652	17.093	1.00	0.00	H
ATOM	346	HA2	GLY	A	149	7.735	-9.960	16.670	1.00	0.00	H
ATOM	347	HA3	GLY	A	149	7.694	-11.275	15.335	1.00	0.00	H
ATOM	348	N	ILE	A	150	4.815	-10.511	15.478	1.00	0.00	N
ATOM	349	CA	ILE	A	150	3.717	-9.879	14.845	1.00	0.00	C
ATOM	350	CB	ILE	A	150	2.562	-10.853	15.021	1.00	0.00	C
ATOM	351	CG2	ILE	A	150	2.261	-11.029	16.555	1.00	0.00	C
ATOM	352	CG1	ILE	A	150	1.250	-10.426	14.310	1.00	0.00	C
ATOM	353	CD1	ILE	A	150	1.366	-10.393	12.774	1.00	0.00	C
ATOM	354	C	ILE	A	150	3.324	-8.426	15.314	1.00	0.00	C
ATOM	355	O	ILE	A	150	2.781	-7.612	14.588	1.00	0.00	O
ATOM	356	H	ILE	A	150	4.743	-11.379	15.946	1.00	0.00	H
ATOM	357	HA	ILE	A	150	3.978	-9.825	13.804	1.00	0.00	H
ATOM	358	HB	ILE	A	150	2.811	-11.891	14.692	1.00	0.00	H
ATOM	359	HG12	ILE	A	150	0.931	-9.468	14.782	1.00	0.00	H
ATOM	360	HG13	ILE	A	150	0.433	-11.097	14.636	1.00	0.00	H
ATOM	361	HG21	ILE	A	150	3.148	-11.302	17.157	1.00	0.00	H
ATOM	362	HG22	ILE	A	150	1.418	-11.762	16.513	1.00	0.00	H
ATOM	363	HG23	ILE	A	150	1.861	-10.111	17.037	1.00	0.00	H

ATOM	364	HD11	ILE	A	150	0.575	-9.712	12.395	1.00	0.00	H
ATOM	365	HD12	ILE	A	150	1.374	-11.390	12.270	1.00	0.00	H
ATOM	366	HD13	ILE	A	150	2.286	-9.844	12.482	1.00	0.00	H
ATOM	367	N	GLY	A	151	3.534	-8.148	16.608	1.00	0.00	N
ATOM	368	CA	GLY	A	151	3.320	-6.822	17.192	1.00	0.00	C
ATOM	369	C	GLY	A	151	4.499	-5.785	17.122	1.00	0.00	C
ATOM	370	O	GLY	A	151	4.302	-4.569	17.205	1.00	0.00	O
ATOM	371	H	GLY	A	151	3.856	-8.891	17.211	1.00	0.00	H
ATOM	372	HA2	GLY	A	151	2.536	-6.398	16.565	1.00	0.00	H
ATOM	373	HA3	GLY	A	151	3.046	-6.941	18.267	1.00	0.00	H
ATOM	374	HC	GLY	A	151	5.509	-6.146	16.998	1.00	0.00	H
ATOM	375	N	GLY	A	162	7.102	-2.297	2.905	1.00	0.00	N1+
ATOM	376	CA	GLY	A	162	7.699	-3.175	1.915	1.00	0.00	C
ATOM	377	C	GLY	A	162	6.763	-4.218	1.373	1.00	0.00	C
ATOM	378	O	GLY	A	162	6.760	-4.516	0.206	1.00	0.00	O
ATOM	379	H1	GLY	A	162	7.089	-1.391	2.489	1.00	0.00	H
ATOM	380	H2	GLY	A	162	7.578	-2.490	3.759	1.00	0.00	H
ATOM	381	HA2	GLY	A	162	7.979	-2.548	1.069	1.00	0.00	H
ATOM	382	HA3	GLY	A	162	8.576	-3.676	2.369	1.00	0.00	H
ATOM	383	N	SER	A	163	5.779	-4.674	2.162	1.00	0.00	N
ATOM	384	CA	SER	A	163	4.953	-5.805	1.802	1.00	0.00	C
ATOM	385	CB	SER	A	163	4.831	-6.883	2.930	1.00	0.00	C
ATOM	386	OG	SER	A	163	6.037	-7.402	3.475	1.00	0.00	O
ATOM	387	C	SER	A	163	3.565	-5.374	1.413	1.00	0.00	C
ATOM	388	O	SER	A	163	2.769	-6.142	0.904	1.00	0.00	O
ATOM	389	H	SER	A	163	5.851	-4.404	3.120	1.00	0.00	H
ATOM	390	HA	SER	A	163	5.413	-6.285	0.952	1.00	0.00	H
ATOM	391	HB2	SER	A	163	4.248	-7.712	2.478	1.00	0.00	H
ATOM	392	HB3	SER	A	163	4.263	-6.431	3.774	1.00	0.00	H

ATOM	393	HG	SER	A	163	6.461	-7.906	2.781	1.00	0.00	H
ATOM	394	N	PHE	A	164	3.337	-4.073	1.416	1.00	0.00	N
ATOM	395	CA	PHE	A	164	2.041	-3.534	1.052	1.00	0.00	C
ATOM	396	CB	PHE	A	164	1.107	-3.557	2.281	1.00	0.00	C
ATOM	397	CG	PHE	A	164	-0.183	-2.867	2.131	1.00	0.00	C
ATOM	398	CD1	PHE	A	164	-1.126	-3.295	1.121	1.00	0.00	C
ATOM	399	CE1	PHE	A	164	-2.406	-2.697	1.045	1.00	0.00	C
ATOM	400	CZ	PHE	A	164	-2.771	-1.628	1.880	1.00	0.00	C
ATOM	401	CD2	PHE	A	164	-0.537	-1.752	2.933	1.00	0.00	C
ATOM	402	CE2	PHE	A	164	-1.804	-1.117	2.813	1.00	0.00	C
ATOM	403	C	PHE	A	164	2.021	-2.232	0.274	1.00	0.00	C
ATOM	404	O	PHE	A	164	1.612	-2.262	-0.866	1.00	0.00	O
ATOM	405	H	PHE	A	164	4.042	-3.508	1.813	1.00	0.00	H
ATOM	406	HA	PHE	A	164	1.542	-4.163	0.330	1.00	0.00	H
ATOM	407	HB2	PHE	A	164	1.682	-2.997	3.057	1.00	0.00	H
ATOM	408	HB3	PHE	A	164	0.950	-4.625	2.525	1.00	0.00	H
ATOM	409	HD1	PHE	A	164	-1.000	-4.185	0.519	1.00	0.00	H
ATOM	410	HD2	PHE	A	164	0.138	-1.389	3.685	1.00	0.00	H
ATOM	411	HE1	PHE	A	164	-3.100	-2.960	0.261	1.00	0.00	H
ATOM	412	HE2	PHE	A	164	-2.208	-0.446	3.558	1.00	0.00	H
ATOM	413	HZ	PHE	A	164	-3.718	-1.154	1.709	1.00	0.00	H
ATOM	414	N	GLY	A	165	2.278	-1.085	1.002	1.00	0.00	N
ATOM	415	CA	GLY	A	165	2.180	0.296	0.503	1.00	0.00	C
ATOM	416	C	GLY	A	165	2.988	0.518	-0.800	1.00	0.00	C
ATOM	417	O	GLY	A	165	2.608	1.019	-1.885	1.00	0.00	O
ATOM	418	H	GLY	A	165	2.559	-1.206	1.965	1.00	0.00	H
ATOM	419	HA2	GLY	A	165	1.130	0.486	0.282	1.00	0.00	H
ATOM	420	HA3	GLY	A	165	2.559	0.981	1.267	1.00	0.00	H
ATOM	421	HC	GLY	A	165	4.019	0.198	-0.770	1.00	0.00	H

ATOM	422	N	GLY	A	212	-11.478	-5.447	-3.752	1.00	0.00	N1+
ATOM	423	CA	GLY	A	212	-11.659	-6.440	-2.752	1.00	0.00	C
ATOM	424	C	GLY	A	212	-10.386	-6.573	-1.848	1.00	0.00	C
ATOM	425	O	GLY	A	212	-10.354	-6.690	-0.637	1.00	0.00	O
ATOM	426	H1	GLY	A	212	-12.153	-4.727	-3.546	1.00	0.00	H
ATOM	427	H2	GLY	A	212	-11.508	-5.938	-4.632	1.00	0.00	H
ATOM	428	HA2	GLY	A	212	-12.515	-6.129	-2.152	1.00	0.00	H
ATOM	429	HA3	GLY	A	212	-11.842	-7.417	-3.205	1.00	0.00	H
ATOM	430	N	PHE	A	213	-9.153	-6.644	-2.453	1.00	0.00	N
ATOM	431	CA	PHE	A	213	-7.845	-6.849	-1.812	1.00	0.00	C
ATOM	432	CB	PHE	A	213	-6.837	-7.151	-2.966	1.00	0.00	C
ATOM	433	CG	PHE	A	213	-5.473	-7.644	-2.531	1.00	0.00	C
ATOM	434	CD1	PHE	A	213	-5.305	-8.977	-2.263	1.00	0.00	C
ATOM	435	CE1	PHE	A	213	-4.018	-9.477	-1.859	1.00	0.00	C
ATOM	436	CZ	PHE	A	213	-2.979	-8.555	-1.677	1.00	0.00	C
ATOM	437	CD2	PHE	A	213	-4.394	-6.792	-2.562	1.00	0.00	C
ATOM	438	CE2	PHE	A	213	-3.173	-7.231	-2.012	1.00	0.00	C
ATOM	439	C	PHE	A	213	-7.389	-5.630	-0.999	1.00	0.00	C
ATOM	440	O	PHE	A	213	-6.885	-5.660	0.132	1.00	0.00	O
ATOM	441	H	PHE	A	213	-9.104	-6.519	-3.441	1.00	0.00	H
ATOM	442	HA	PHE	A	213	-7.950	-7.665	-1.121	1.00	0.00	H
ATOM	443	HB2	PHE	A	213	-6.689	-6.266	-3.624	1.00	0.00	H
ATOM	444	HB3	PHE	A	213	-7.325	-7.924	-3.592	1.00	0.00	H
ATOM	445	HD1	PHE	A	213	-6.016	-9.693	-2.634	1.00	0.00	H
ATOM	446	HD2	PHE	A	213	-4.510	-5.738	-2.804	1.00	0.00	H
ATOM	447	HE1	PHE	A	213	-3.803	-10.537	-1.780	1.00	0.00	H
ATOM	448	HE2	PHE	A	213	-2.368	-6.501	-2.096	1.00	0.00	H
ATOM	449	HZ	PHE	A	213	-2.001	-8.891	-1.375	1.00	0.00	H
ATOM	450	N	GLY	A	214	-7.615	-4.423	-1.567	1.00	0.00	N

ATOM	451	CA	GLY	A	214	-7.552	-3.220	-0.698	1.00	0.00	C
ATOM	452	C	GLY	A	214	-8.602	-3.029	0.405	1.00	0.00	C
ATOM	453	O	GLY	A	214	-8.215	-2.730	1.557	1.00	0.00	O
ATOM	454	H	GLY	A	214	-7.820	-4.342	-2.553	1.00	0.00	H
ATOM	455	HA2	GLY	A	214	-6.633	-3.366	-0.131	1.00	0.00	H
ATOM	456	HA3	GLY	A	214	-7.575	-2.316	-1.366	1.00	0.00	H
ATOM	457	HC	GLY	A	214	-9.654	-3.150	0.191	1.00	0.00	H
ATOM	458	N	GLY	A	216	-10.146	-5.414	2.016	1.00	0.00	N1+
ATOM	459	CA	GLY	A	216	-9.794	-6.447	3.046	1.00	0.00	C
ATOM	460	C	GLY	A	216	-8.599	-6.055	3.998	1.00	0.00	C
ATOM	461	O	GLY	A	216	-8.544	-6.356	5.203	1.00	0.00	O
ATOM	462	H1	GLY	A	216	-11.032	-5.114	2.293	1.00	0.00	H
ATOM	463	H2	GLY	A	216	-9.902	-5.844	1.175	1.00	0.00	H
ATOM	464	HA2	GLY	A	216	-10.674	-6.548	3.681	1.00	0.00	H
ATOM	465	HA3	GLY	A	216	-9.528	-7.379	2.531	1.00	0.00	H
ATOM	466	N	TRP	A	217	-7.544	-5.436	3.384	1.00	0.00	N
ATOM	467	CA	TRP	A	217	-6.221	-5.337	4.001	1.00	0.00	C
ATOM	468	CB	TRP	A	217	-5.055	-5.912	3.122	1.00	0.00	C
ATOM	469	CG	TRP	A	217	-5.143	-7.348	2.799	1.00	0.00	C
ATOM	470	CD1	TRP	A	217	-5.187	-7.948	1.540	1.00	0.00	C
ATOM	471	NE1	TRP	A	217	-5.088	-9.307	1.706	1.00	0.00	N
ATOM	472	CE2	TRP	A	217	-5.095	-9.619	3.060	1.00	0.00	C
ATOM	473	CD2	TRP	A	217	-5.151	-8.385	3.771	1.00	0.00	C
ATOM	474	CE3	TRP	A	217	-5.066	-8.366	5.191	1.00	0.00	C
ATOM	475	CZ3	TRP	A	217	-5.111	-9.554	5.870	1.00	0.00	C
ATOM	476	CZ2	TRP	A	217	-5.212	-10.780	3.757	1.00	0.00	C
ATOM	477	CH2	TRP	A	217	-5.253	-10.723	5.140	1.00	0.00	C
ATOM	478	C	TRP	A	217	-5.967	-3.914	4.416	1.00	0.00	C
ATOM	479	O	TRP	A	217	-5.181	-3.778	5.376	1.00	0.00	O

ATOM	480	H	TRP	A	217	-7.518	-5.276	2.397	1.00	0.00	H
ATOM	481	HA	TRP	A	217	-6.171	-5.863	4.954	1.00	0.00	H
ATOM	482	HB2	TRP	A	217	-4.076	-5.821	3.638	1.00	0.00	H
ATOM	483	HB3	TRP	A	217	-5.018	-5.365	2.158	1.00	0.00	H
ATOM	484	HD1	TRP	A	217	-5.316	-7.324	0.672	1.00	0.00	H
ATOM	485	HE1	TRP	A	217	-5.443	-9.989	1.111	1.00	0.00	H
ATOM	486	HE3	TRP	A	217	-4.946	-7.439	5.733	1.00	0.00	H
ATOM	487	HZ2	TRP	A	217	-5.232	-11.735	3.245	1.00	0.00	H
ATOM	488	HZ3	TRP	A	217	-5.200	-9.535	6.948	1.00	0.00	H
ATOM	489	HH2	TRP	A	217	-5.435	-11.652	5.663	1.00	0.00	H
ATOM	490	N	GLY	A	218	-6.590	-2.949	3.824	1.00	0.00	N
ATOM	491	CA	GLY	A	218	-6.365	-1.537	4.313	1.00	0.00	C
ATOM	492	C	GLY	A	218	-6.838	-1.253	5.737	1.00	0.00	C
ATOM	493	O	GLY	A	218	-6.057	-0.575	6.454	1.00	0.00	O
ATOM	494	H	GLY	A	218	-7.214	-3.134	3.051	1.00	0.00	H
ATOM	495	HA2	GLY	A	218	-5.286	-1.383	4.342	1.00	0.00	H
ATOM	496	HA3	GLY	A	218	-6.882	-0.857	3.613	1.00	0.00	H
ATOM	497	N	GLY	A	219	-7.924	-1.778	6.290	1.00	0.00	N
ATOM	498	CA	GLY	A	219	-8.216	-1.662	7.730	1.00	0.00	C
ATOM	499	C	GLY	A	219	-7.318	-2.474	8.545	1.00	0.00	C
ATOM	500	O	GLY	A	219	-6.975	-2.074	9.647	1.00	0.00	O
ATOM	501	H	GLY	A	219	-8.576	-2.276	5.701	1.00	0.00	H
ATOM	502	HA2	GLY	A	219	-8.061	-0.630	8.045	1.00	0.00	H
ATOM	503	HA3	GLY	A	219	-9.264	-1.967	7.885	1.00	0.00	H
ATOM	504	N	PHE	A	220	-6.894	-3.650	8.101	1.00	0.00	N
ATOM	505	CA	PHE	A	220	-5.957	-4.490	8.893	1.00	0.00	C
ATOM	506	CB	PHE	A	220	-5.670	-5.907	8.374	1.00	0.00	C
ATOM	507	CG	PHE	A	220	-4.384	-6.578	8.946	1.00	0.00	C
ATOM	508	CD1	PHE	A	220	-4.271	-7.049	10.283	1.00	0.00	C

ATOM	509	CE1	PHE	A	220	-3.160	-7.837	10.669	1.00	0.00	C
ATOM	510	CZ	PHE	A	220	-2.177	-8.158	9.670	1.00	0.00	C
ATOM	511	CD2	PHE	A	220	-3.325	-6.904	8.100	1.00	0.00	C
ATOM	512	CE2	PHE	A	220	-2.268	-7.785	8.396	1.00	0.00	C
ATOM	513	C	PHE	A	220	-4.637	-3.690	9.077	1.00	0.00	C
ATOM	514	O	PHE	A	220	-4.400	-3.445	10.239	1.00	0.00	O
ATOM	515	H	PHE	A	220	-7.118	-3.933	7.172	1.00	0.00	H
ATOM	516	HA	PHE	A	220	-6.434	-4.570	9.849	1.00	0.00	H
ATOM	517	HB2	PHE	A	220	-5.495	-5.779	7.290	1.00	0.00	H
ATOM	518	HB3	PHE	A	220	-6.574	-6.493	8.676	1.00	0.00	H
ATOM	519	HD1	PHE	A	220	-5.003	-6.774	11.012	1.00	0.00	H
ATOM	520	HD2	PHE	A	220	-3.371	-6.462	7.110	1.00	0.00	H
ATOM	521	HE1	PHE	A	220	-3.190	-8.211	11.676	1.00	0.00	H
ATOM	522	HE2	PHE	A	220	-1.521	-7.991	7.631	1.00	0.00	H
ATOM	523	HZ	PHE	A	220	-1.342	-8.759	10.031	1.00	0.00	H
ATOM	524	N	PHE	A	221	-3.974	-3.255	7.994	1.00	0.00	N
ATOM	525	CA	PHE	A	221	-2.794	-2.421	8.103	1.00	0.00	C
ATOM	526	CB	PHE	A	221	-2.039	-2.197	6.725	1.00	0.00	C
ATOM	527	CG	PHE	A	221	-1.334	-3.483	6.323	1.00	0.00	C
ATOM	528	CD1	PHE	A	221	-0.175	-3.923	6.971	1.00	0.00	C
ATOM	529	CE1	PHE	A	221	0.410	-5.147	6.641	1.00	0.00	C
ATOM	530	CZ	PHE	A	221	-0.108	-5.888	5.554	1.00	0.00	C
ATOM	531	CD2	PHE	A	221	-1.854	-4.257	5.288	1.00	0.00	C
ATOM	532	CE2	PHE	A	221	-1.264	-5.425	4.904	1.00	0.00	C
ATOM	533	C	PHE	A	221	-3.064	-1.104	8.795	1.00	0.00	C
ATOM	534	O	PHE	A	221	-2.249	-0.829	9.716	1.00	0.00	O
ATOM	535	H	PHE	A	221	-4.269	-3.511	7.082	1.00	0.00	H
ATOM	536	HA	PHE	A	221	-2.171	-3.003	8.769	1.00	0.00	H
ATOM	537	HB2	PHE	A	221	-1.290	-1.381	6.774	1.00	0.00	H

ATOM	538	HB3	PHE	A	221	-2.739	-1.898	5.908	1.00	0.00	H
ATOM	539	HD1	PHE	A	221	0.331	-3.452	7.799	1.00	0.00	H
ATOM	540	HD2	PHE	A	221	-2.637	-3.871	4.648	1.00	0.00	H
ATOM	541	HE1	PHE	A	221	1.385	-5.419	7.022	1.00	0.00	H
ATOM	542	HE2	PHE	A	221	-1.876	-5.978	4.183	1.00	0.00	H
ATOM	543	HZ	PHE	A	221	0.516	-6.692	5.212	1.00	0.00	H
ATOM	544	N	GLY	A	222	-4.218	-0.397	8.599	1.00	0.00	N
ATOM	545	CA	GLY	A	222	-4.471	0.851	9.393	1.00	0.00	C
ATOM	546	C	GLY	A	222	-4.606	0.566	10.831	1.00	0.00	C
ATOM	547	O	GLY	A	222	-4.167	1.311	11.736	1.00	0.00	O
ATOM	548	H	GLY	A	222	-4.898	-0.714	7.922	1.00	0.00	H
ATOM	549	HA2	GLY	A	222	-3.624	1.530	9.295	1.00	0.00	H
ATOM	550	HA3	GLY	A	222	-5.386	1.309	8.999	1.00	0.00	H
ATOM	551	HC	GLY	A	222	-5.115	-0.341	11.123	1.00	0.00	H
ATOM	552	N	GLY	A	238	-12.712	-7.750	16.533	1.00	0.00	N1+
ATOM	553	CA	GLY	A	238	-12.405	-6.573	15.611	1.00	0.00	C
ATOM	554	C	GLY	A	238	-11.090	-6.784	14.760	1.00	0.00	C
ATOM	555	O	GLY	A	238	-11.125	-6.572	13.536	1.00	0.00	O
ATOM	556	H1	GLY	A	238	-13.539	-8.106	16.151	1.00	0.00	H
ATOM	557	H2	GLY	A	238	-12.597	-7.357	17.421	1.00	0.00	H
ATOM	558	HA2	GLY	A	238	-13.234	-6.477	14.910	1.00	0.00	H
ATOM	559	HA3	GLY	A	238	-12.283	-5.684	16.222	1.00	0.00	H
ATOM	560	N	TYR	A	239	-10.074	-7.213	15.576	1.00	0.00	N
ATOM	561	CA	TYR	A	239	-8.834	-7.676	14.996	1.00	0.00	C
ATOM	562	CB	TYR	A	239	-7.686	-7.833	16.027	1.00	0.00	C
ATOM	563	CG	TYR	A	239	-6.339	-8.352	15.528	1.00	0.00	C
ATOM	564	CD1	TYR	A	239	-5.294	-7.449	15.378	1.00	0.00	C
ATOM	565	CE1	TYR	A	239	-4.115	-7.774	14.719	1.00	0.00	C
ATOM	566	CZ	TYR	A	239	-3.824	-9.148	14.429	1.00	0.00	C

ATOM	567	OH	TYR	A	239	-2.581	-9.545	13.978	1.00	0.00	O
ATOM	568	CD2	TYR	A	239	-6.091	-9.656	15.190	1.00	0.00	C
ATOM	569	CE2	TYR	A	239	-4.854	-10.034	14.577	1.00	0.00	C
ATOM	570	C	TYR	A	239	-8.973	-8.938	14.037	1.00	0.00	C
ATOM	571	O	TYR	A	239	-8.514	-9.025	12.889	1.00	0.00	O
ATOM	572	H	TYR	A	239	-10.272	-7.478	16.507	1.00	0.00	H
ATOM	573	HA	TYR	A	239	-8.611	-6.912	14.253	1.00	0.00	H
ATOM	574	HB2	TYR	A	239	-7.977	-8.578	16.798	1.00	0.00	H
ATOM	575	HB3	TYR	A	239	-7.519	-6.846	16.521	1.00	0.00	H
ATOM	576	HD1	TYR	A	239	-5.432	-6.414	15.653	1.00	0.00	H
ATOM	577	HD2	TYR	A	239	-6.835	-10.449	15.240	1.00	0.00	H
ATOM	578	HE1	TYR	A	239	-3.382	-7.007	14.591	1.00	0.00	H
ATOM	579	HE2	TYR	A	239	-4.614	-11.053	14.309	1.00	0.00	H
ATOM	580	HH	TYR	A	239	-2.114	-8.704	13.988	1.00	0.00	H
ATOM	581	N	GLY	A	240	-9.738	-10.005	14.474	1.00	0.00	N
ATOM	582	CA	GLY	A	240	-9.887	-11.177	13.603	1.00	0.00	C
ATOM	583	C	GLY	A	240	-10.788	-11.068	12.445	1.00	0.00	C
ATOM	584	O	GLY	A	240	-10.659	-11.754	11.450	1.00	0.00	O
ATOM	585	H	GLY	A	240	-10.187	-9.978	15.378	1.00	0.00	H
ATOM	586	HA2	GLY	A	240	-8.912	-11.310	13.134	1.00	0.00	H
ATOM	587	HA3	GLY	A	240	-10.212	-12.037	14.254	1.00	0.00	H
ATOM	588	HC	GLY	A	240	-11.595	-10.351	12.480	1.00	0.00	H
ATOM	589	N	GLY	A	242	-10.950	-8.452	10.087	1.00	0.00	N1+
ATOM	590	CA	GLY	A	242	-10.215	-8.076	8.897	1.00	0.00	C
ATOM	591	C	GLY	A	242	-9.456	-9.300	8.264	1.00	0.00	C
ATOM	592	O	GLY	A	242	-9.646	-9.623	7.071	1.00	0.00	O
ATOM	593	H1	GLY	A	242	-11.910	-8.315	9.848	1.00	0.00	H
ATOM	594	H2	GLY	A	242	-10.507	-7.953	10.829	1.00	0.00	H
ATOM	595	HA2	GLY	A	242	-10.946	-7.697	8.183	1.00	0.00	H

ATOM	596	HA3	GLY	A	242	-9.471	-7.315	9.140	1.00	0.00	H
ATOM	597	N	THR	A	243	-8.738	-10.125	9.061	1.00	0.00	N
ATOM	598	CA	THR	A	243	-8.166	-11.384	8.613	1.00	0.00	C
ATOM	599	CB	THR	A	243	-7.180	-12.154	9.487	1.00	0.00	C
ATOM	600	OG1	THR	A	243	-5.982	-11.483	9.530	1.00	0.00	O
ATOM	601	CG2	THR	A	243	-6.857	-13.611	9.046	1.00	0.00	C
ATOM	602	C	THR	A	243	-9.185	-12.330	8.117	1.00	0.00	C
ATOM	603	O	THR	A	243	-8.995	-12.919	7.041	1.00	0.00	O
ATOM	604	H	THR	A	243	-8.766	-9.955	10.045	1.00	0.00	H
ATOM	605	HA	THR	A	243	-7.607	-11.126	7.720	1.00	0.00	H
ATOM	606	HB	THR	A	243	-7.620	-12.198	10.497	1.00	0.00	H
ATOM	607	HG21	THR	A	243	-7.825	-14.145	9.172	1.00	0.00	H
ATOM	608	HG22	THR	A	243	-6.076	-14.008	9.717	1.00	0.00	H
ATOM	609	HG23	THR	A	243	-6.520	-13.711	7.993	1.00	0.00	H
ATOM	610	HG1	THR	A	243	-6.085	-10.723	10.115	1.00	0.00	H
ATOM	611	N	GLY	A	244	-10.340	-12.592	8.797	1.00	0.00	N
ATOM	612	CA	GLY	A	244	-11.509	-13.401	8.273	1.00	0.00	C
ATOM	613	C	GLY	A	244	-12.002	-12.866	6.918	1.00	0.00	C
ATOM	614	O	GLY	A	244	-12.255	-13.628	5.982	1.00	0.00	O
ATOM	615	H	GLY	A	244	-10.422	-12.214	9.730	1.00	0.00	H
ATOM	616	HA2	GLY	A	244	-11.191	-14.433	8.124	1.00	0.00	H
ATOM	617	HA3	GLY	A	244	-12.312	-13.342	9.001	1.00	0.00	H
ATOM	618	HC	GLY	A	244	-12.129	-11.802	6.784	1.00	0.00	H
ATOM	619	N	GLY	A	246	-10.066	-11.264	4.568	1.00	0.00	N1+
ATOM	620	CA	GLY	A	246	-8.990	-11.530	3.617	1.00	0.00	C
ATOM	621	C	GLY	A	246	-9.181	-12.951	3.124	1.00	0.00	C
ATOM	622	O	GLY	A	246	-8.780	-13.325	2.044	1.00	0.00	O
ATOM	623	H1	GLY	A	246	-11.034	-11.271	4.281	1.00	0.00	H
ATOM	624	H2	GLY	A	246	-9.806	-11.074	5.517	1.00	0.00	H

ATOM	625	HA2	GLY	A	246	-8.030	-11.511	4.118	1.00	0.00	H
ATOM	626	HA3	GLY	A	246	-9.019	-10.837	2.795	1.00	0.00	H
ATOM	627	N	TYR	A	247	-9.571	-13.850	4.013	1.00	0.00	N
ATOM	628	CA	TYR	A	247	-9.801	-15.221	3.604	1.00	0.00	C
ATOM	629	CB	TYR	A	247	-9.709	-16.190	4.792	1.00	0.00	C
ATOM	630	CG	TYR	A	247	-8.298	-16.197	5.383	1.00	0.00	C
ATOM	631	CD1	TYR	A	247	-7.119	-16.022	4.723	1.00	0.00	C
ATOM	632	CE1	TYR	A	247	-5.854	-16.126	5.405	1.00	0.00	C
ATOM	633	CZ	TYR	A	247	-5.915	-16.405	6.764	1.00	0.00	C
ATOM	634	OH	TYR	A	247	-4.729	-16.516	7.557	1.00	0.00	O
ATOM	635	CD2	TYR	A	247	-8.333	-16.457	6.748	1.00	0.00	C
ATOM	636	CE2	TYR	A	247	-7.152	-16.506	7.508	1.00	0.00	C
ATOM	637	C	TYR	A	247	-11.167	-15.495	2.909	1.00	0.00	C
ATOM	638	O	TYR	A	247	-11.337	-16.450	2.233	1.00	0.00	O
ATOM	639	H	TYR	A	247	-9.798	-13.540	4.934	1.00	0.00	H
ATOM	640	HA	TYR	A	247	-9.105	-15.670	2.926	1.00	0.00	H
ATOM	641	HB2	TYR	A	247	-9.922	-17.257	4.620	1.00	0.00	H
ATOM	642	HB3	TYR	A	247	-10.384	-15.692	5.516	1.00	0.00	H
ATOM	643	HD1	TYR	A	247	-7.077	-15.878	3.654	1.00	0.00	H
ATOM	644	HD2	TYR	A	247	-9.308	-16.430	7.234	1.00	0.00	H
ATOM	645	HE1	TYR	A	247	-4.921	-15.923	4.904	1.00	0.00	H
ATOM	646	HE2	TYR	A	247	-7.169	-16.662	8.575	1.00	0.00	H
ATOM	647	HH	TYR	A	247	-3.977	-16.241	7.024	1.00	0.00	H
ATOM	648	N	GLY	A	248	-12.095	-14.578	3.040	1.00	0.00	N
ATOM	649	CA	GLY	A	248	-13.247	-14.497	2.184	1.00	0.00	C
ATOM	650	C	GLY	A	248	-12.834	-14.142	0.770	1.00	0.00	C
ATOM	651	O	GLY	A	248	-13.402	-14.657	-0.159	1.00	0.00	O
ATOM	652	H	GLY	A	248	-11.996	-13.896	3.778	1.00	0.00	H
ATOM	653	HA2	GLY	A	248	-13.743	-15.468	2.179	1.00	0.00	H

ATOM	654	HA3	GLY	A	248	-13.929	-13.730	2.558	1.00	0.00	H
ATOM	655	HC	GLY	A	248	-12.037	-13.434	0.597	1.00	0.00	H
TER	656		GLY	A	248						
HETATM	657	C1	GMJ	B	274	-1.275	-11.191	7.726	1.00	0.00	C
HETATM	658	C2	GMJ	B	274	-2.521	-11.923	8.285	1.00	0.00	C
HETATM	659	C3	GMJ	B	274	-1.674	-8.969	3.709	1.00	0.00	C
HETATM	660	C4	GMJ	B	274	-1.785	-8.188	2.511	1.00	0.00	C
HETATM	661	C5	GMJ	B	274	-0.648	-7.571	2.110	1.00	0.00	C
HETATM	662	C6	GMJ	B	274	0.600	-7.840	2.609	1.00	0.00	C
HETATM	663	C7	GMJ	B	274	0.688	-8.640	3.741	1.00	0.00	C
HETATM	664	C8	GMJ	B	274	-0.462	-9.081	4.449	1.00	0.00	C
HETATM	665	C9	GMJ	B	274	-0.264	-9.798	5.755	1.00	0.00	C
HETATM	666	C10	GMJ	B	274	-1.337	-10.722	6.282	1.00	0.00	C
HETATM	667	C11	GMJ	B	274	-3.176	-13.611	9.944	1.00	0.00	C
HETATM	668	C12	GMJ	B	274	-2.815	-14.375	11.375	1.00	0.00	C
HETATM	669	C13	GMJ	B	274	-2.705	-13.452	12.482	1.00	0.00	C
HETATM	670	C14	GMJ	B	274	-1.611	-12.298	12.094	1.00	0.00	C
HETATM	671	C15	GMJ	B	274	-2.073	-11.538	10.867	1.00	0.00	C
HETATM	672	C16	GMJ	B	274	-2.087	-13.963	13.791	1.00	0.00	C
HETATM	673	C17	GMJ	B	274	-0.925	-14.742	13.698	1.00	0.00	C
HETATM	674	C18	GMJ	B	274	-0.311	-15.286	14.898	1.00	0.00	C
HETATM	675	C19	GMJ	B	274	-0.865	-14.936	16.127	1.00	0.00	C
HETATM	676	C20	GMJ	B	274	-2.007	-14.128	16.207	1.00	0.00	C
HETATM	677	C21	GMJ	B	274	-2.543	-13.556	15.037	1.00	0.00	C
HETATM	678	F	GMJ	B	274	-0.683	-7.103	0.865	1.00	0.00	F
HETATM	679	N	GMJ	B	274	-2.207	-12.523	9.654	1.00	0.00	N1+
HETATM	680	O1	GMJ	B	274	0.784	-9.800	6.368	1.00	0.00	O
HETATM	681	O2	GMJ	B	274	-3.994	-12.848	12.598	1.00	0.00	O
HETATM	682	Cl	GMJ	B	274	-0.058	-15.537	17.569	1.00	0.00	Cl

HETATM	683	H1	GMJ	B	274	-0.434	-11.885	7.948	1.00	0.00	H
HETATM	684	H2	GMJ	B	274	-1.066	-10.285	8.318	1.00	0.00	H
HETATM	685	H3	GMJ	B	274	-2.871	-12.773	7.677	1.00	0.00	H
HETATM	686	H4	GMJ	B	274	-3.441	-11.353	8.501	1.00	0.00	H
HETATM	687	H5	GMJ	B	274	-2.556	-9.387	4.163	1.00	0.00	H
HETATM	688	H6	GMJ	B	274	-2.775	-8.166	2.062	1.00	0.00	H
HETATM	689	H7	GMJ	B	274	1.483	-7.475	2.098	1.00	0.00	H
HETATM	690	H8	GMJ	B	274	1.706	-8.854	4.034	1.00	0.00	H
HETATM	691	H9	GMJ	B	274	-2.319	-10.271	6.026	1.00	0.00	H
HETATM	692	H10	GMJ	B	274	-1.301	-11.634	5.661	1.00	0.00	H
HETATM	693	H11	GMJ	B	274	-4.116	-13.049	9.910	1.00	0.00	H
HETATM	694	H12	GMJ	B	274	-3.073	-14.339	9.126	1.00	0.00	H
HETATM	695	H13	GMJ	B	274	-1.923	-14.997	11.135	1.00	0.00	H
HETATM	696	H14	GMJ	B	274	-3.573	-15.173	11.477	1.00	0.00	H
HETATM	697	H15	GMJ	B	274	-0.616	-12.775	11.977	1.00	0.00	H
HETATM	698	H16	GMJ	B	274	-1.570	-11.581	12.926	1.00	0.00	H
HETATM	699	H17	GMJ	B	274	-1.343	-10.761	10.578	1.00	0.00	H
HETATM	700	H18	GMJ	B	274	-3.052	-11.055	10.962	1.00	0.00	H
HETATM	701	H19	GMJ	B	274	-0.492	-15.137	12.807	1.00	0.00	H
HETATM	702	H20	GMJ	B	274	0.501	-15.969	14.690	1.00	0.00	H
HETATM	703	H21	GMJ	B	274	-2.466	-13.828	17.136	1.00	0.00	H
HETATM	704	H22	GMJ	B	274	-3.251	-12.739	15.016	1.00	0.00	H
HETATM	705	H23	GMJ	B	274	-1.258	-12.953	9.561	1.00	0.00	H
HETATM	706	H24	GMJ	B	274	-4.574	-13.549	12.927	1.00	0.00	H
HETATM	707	OH2	TIP	X7605		1.850	-12.203	9.175	1.00	0.00	O
HETATM	708	H1	TIP	X7605		1.176	-12.874	9.282	1.00	0.00	H
HETATM	709	H2	TIP	X7605		2.675	-12.679	9.148	1.00	0.00	H
HETATM	710	OH2	TIP	X1365		1.075	-12.093	19.812	1.00	0.00	O
HETATM	711	H1	TIP	X1365		1.884	-12.011	20.318	1.00	0.00	H

```
HETATM  712  H2  TIP X1365       1.366 -12.358  18.932  1.00  0.00           H
HETATM  713  OH2 TIP X3707      -3.099 -11.815  18.627  1.00  0.00           O
HETATM  714  H1  TIP X3707      -3.428 -12.712  18.774  1.00  0.00           H
HETATM  715  H2  TIP X3707      -3.207 -11.387  19.479  1.00  0.00           H
HETATM  716  OH2 TIP X8198      -5.893 -15.255  13.576  1.00  0.00           O
HETATM  717  H1  TIP X8198      -6.000 -16.071  13.109  1.00  0.00           H
HETATM  718  H2  TIP X8198      -6.481 -15.311  14.323  1.00  0.00           H
HETATM  719  OH2 TIP X1123      -4.254 -14.504  19.107  1.00  0.00           O
HETATM  720  H1  TIP X1123      -5.153 -14.396  19.419  1.00  0.00           H
HETATM  721  H2  TIP X1123      -4.309 -15.183  18.436  1.00  0.00           H
HETATM  722  OH2 TIP X8014       1.921  -6.526   9.716  1.00  0.00           O
HETATM  723  H1  TIP X8014       2.728  -6.284   9.268  1.00  0.00           H
HETATM  724  H2  TIP X8014       1.777  -7.451   9.479  1.00  0.00           H
HETATM  725  OH2 TIP X 802      -2.475 -15.406   6.248  1.00  0.00           O
HETATM  726  H1  TIP X 802      -1.736 -15.073   6.752  1.00  0.00           H
HETATM  727  H2  TIP X 802      -2.289 -15.188   5.341  1.00  0.00           H
HETATM  728  OH2 TIP X1185      -1.717 -10.128  17.105  1.00  0.00           O
HETATM  729  H1  TIP X1185      -2.300 -10.703  17.601  1.00  0.00           H
HETATM  730  H2  TIP X1185      -2.224  -9.907  16.319  1.00  0.00           H
HETATM  731  OH2 TIP X2324       1.726  -9.310   8.837  1.00  0.00           O
HETATM  732  H1  TIP X2324       1.553  -9.351   7.902  1.00  0.00           H
HETATM  733  H2  TIP X2324       1.625 -10.209   9.158  1.00  0.00           H
HETATM  734  OH2 TIP X4298      -2.783 -13.565  21.052  1.00  0.00           O
HETATM  735  H1  TIP X4298      -2.862 -12.657  20.771  1.00  0.00           H
HETATM  736  H2  TIP X4298      -3.151 -14.082  20.330  1.00  0.00           H
HETATM  737  OH2 TIP X5181      -6.311  -9.519  11.401  1.00  0.00           O
HETATM  738  H1  TIP X5181      -5.607  -9.476  12.052  1.00  0.00           H
HETATM  739  H2  TIP X5181      -7.115  -9.536  11.919  1.00  0.00           H
END
```

Risperidone (Cluster structure 1)

ATOM	1	N	GLY	A	75	-6.075	12.269	5.641	1.00	0.00	N
ATOM	2	CA	GLY	A	75	-5.465	12.554	4.316	1.00	0.00	C
ATOM	3	C	GLY	A	75	-4.870	11.220	3.865	1.00	0.00	C
ATOM	4	O	GLY	A	75	-4.486	11.088	2.696	1.00	0.00	O
ATOM	5	H1	GLY	A	75	-7.071	12.471	5.502	1.00	0.00	H
ATOM	6	H2	GLY	A	75	-5.561	12.880	6.285	1.00	0.00	H
ATOM	7	HA2	GLY	A	75	-6.183	12.949	3.597	1.00	0.00	H
ATOM	8	HA3	GLY	A	75	-4.690	13.272	4.435	1.00	0.00	H
ATOM	9	N	PHE	A	76	-4.726	10.174	4.723	1.00	0.00	N
ATOM	10	CA	PHE	A	76	-4.033	8.912	4.499	1.00	0.00	C
ATOM	11	CB	PHE	A	76	-2.512	8.931	4.787	1.00	0.00	C
ATOM	12	CG	PHE	A	76	-1.723	7.686	4.707	1.00	0.00	C
ATOM	13	CD1	PHE	A	76	-0.483	7.637	4.101	1.00	0.00	C
ATOM	14	CE1	PHE	A	76	0.286	6.448	4.121	1.00	0.00	C
ATOM	15	CZ	PHE	A	76	-0.123	5.336	4.928	1.00	0.00	C
ATOM	16	CD2	PHE	A	76	-2.191	6.553	5.395	1.00	0.00	C
ATOM	17	CE2	PHE	A	76	-1.370	5.407	5.515	1.00	0.00	C
ATOM	18	C	PHE	A	76	-4.430	8.160	3.206	1.00	0.00	C
ATOM	19	O	PHE	A	76	-3.630	7.829	2.331	1.00	0.00	O
ATOM	20	H	PHE	A	76	-5.179	10.274	5.610	1.00	0.00	H
ATOM	21	HA	PHE	A	76	-4.392	8.269	5.303	1.00	0.00	H
ATOM	22	HB2	PHE	A	76	-2.397	9.414	5.771	1.00	0.00	H
ATOM	23	HB3	PHE	A	76	-2.079	9.723	4.120	1.00	0.00	H
ATOM	24	HD1	PHE	A	76	-0.116	8.543	3.622	1.00	0.00	H
ATOM	25	HD2	PHE	A	76	-3.153	6.614	5.875	1.00	0.00	H
ATOM	26	HE1	PHE	A	76	1.231	6.361	3.608	1.00	0.00	H
ATOM	27	HE2	PHE	A	76	-1.643	4.605	6.191	1.00	0.00	H
ATOM	28	HZ	PHE	A	76	0.479	4.461	5.084	1.00	0.00	H

ATOM	29	N	GLY	A	77	-5.744	7.895	3.003	1.00	0.00	N
ATOM	30	CA	GLY	A	77	-6.297	7.039	1.938	1.00	0.00	C
ATOM	31	C	GLY	A	77	-6.247	7.760	0.555	1.00	0.00	C
ATOM	32	O	GLY	A	77	-5.984	7.209	-0.497	1.00	0.00	O
ATOM	33	H	GLY	A	77	-6.403	8.322	3.638	1.00	0.00	H
ATOM	34	HA2	GLY	A	77	-5.696	6.131	1.888	1.00	0.00	H
ATOM	35	HA3	GLY	A	77	-7.333	6.804	2.165	1.00	0.00	H
ATOM	36	HC	GLY	A	77	-6.460	8.818	0.530	1.00	0.00	H
ATOM	37	N	GLY	A	79	-3.656	9.593	-0.305	1.00	0.00	N
ATOM	38	CA	GLY	A	79	-2.307	9.236	-0.720	1.00	0.00	C
ATOM	39	C	GLY	A	79	-2.077	7.757	-1.062	1.00	0.00	C
ATOM	40	O	GLY	A	79	-2.210	7.332	-2.196	1.00	0.00	O
ATOM	41	H1	GLY	A	79	-4.004	10.171	-1.024	1.00	0.00	H
ATOM	42	H2	GLY	A	79	-3.575	9.852	0.643	1.00	0.00	H
ATOM	43	HA2	GLY	A	79	-2.144	9.781	-1.650	1.00	0.00	H
ATOM	44	HA3	GLY	A	79	-1.597	9.521	0.086	1.00	0.00	H
ATOM	45	N	ASP	A	80	-1.882	6.880	-0.039	1.00	0.00	N
ATOM	46	CA	ASP	A	80	-2.023	5.449	0.041	1.00	0.00	C
ATOM	47	CB	ASP	A	80	-0.661	4.759	0.439	1.00	0.00	C
ATOM	48	CG	ASP	A	80	-0.868	3.336	0.823	1.00	0.00	C
ATOM	49	OD1	ASP	A	80	0.084	2.552	0.965	1.00	0.00	O
ATOM	50	OD2	ASP	A	80	-2.080	2.987	1.153	1.00	0.00	O1-
ATOM	51	C	ASP	A	80	-2.912	4.844	-1.024	1.00	0.00	C
ATOM	52	O	ASP	A	80	-2.430	4.400	-2.084	1.00	0.00	O
ATOM	53	H	ASP	A	80	-1.725	7.254	0.875	1.00	0.00	H
ATOM	54	HA	ASP	A	80	-2.741	5.378	0.857	1.00	0.00	H
ATOM	55	HB2	ASP	A	80	-0.144	5.340	1.235	1.00	0.00	H
ATOM	56	HB3	ASP	A	80	-0.007	4.769	-0.469	1.00	0.00	H
ATOM	57	N	VAL	A	81	-4.220	4.696	-0.797	1.00	0.00	N

ATOM	58	CA	VAL	A	81	-5.127	3.886	-1.581	1.00	0.00	C
ATOM	59	CB	VAL	A	81	-6.486	3.598	-0.913	1.00	0.00	C
ATOM	60	CG1	VAL	A	81	-7.198	2.516	-1.695	1.00	0.00	C
ATOM	61	CG2	VAL	A	81	-6.372	3.142	0.583	1.00	0.00	C
ATOM	62	C	VAL	A	81	-5.359	4.424	-2.977	1.00	0.00	C
ATOM	63	O	VAL	A	81	-5.285	3.627	-3.931	1.00	0.00	O
ATOM	64	H	VAL	A	81	-4.757	5.008	-0.032	1.00	0.00	H
ATOM	65	HA	VAL	A	81	-4.706	2.911	-1.815	1.00	0.00	H
ATOM	66	HB	VAL	A	81	-7.150	4.487	-0.884	1.00	0.00	H
ATOM	67	HG11	VAL	A	81	-6.592	1.579	-1.693	1.00	0.00	H
ATOM	68	HG12	VAL	A	81	-7.351	2.682	-2.775	1.00	0.00	H
ATOM	69	HG13	VAL	A	81	-8.193	2.290	-1.263	1.00	0.00	H
ATOM	70	HG21	VAL	A	81	-7.340	3.069	1.126	1.00	0.00	H
ATOM	71	HG22	VAL	A	81	-5.675	3.774	1.171	1.00	0.00	H
ATOM	72	HG23	VAL	A	81	-5.944	2.114	0.599	1.00	0.00	H
ATOM	73	N	GLY	A	82	-5.553	5.730	-3.194	1.00	0.00	N
ATOM	74	CA	GLY	A	82	-5.637	6.429	-4.393	1.00	0.00	C
ATOM	75	C	GLY	A	82	-4.365	6.290	-5.231	1.00	0.00	C
ATOM	76	O	GLY	A	82	-4.439	5.924	-6.406	1.00	0.00	O
ATOM	77	H	GLY	A	82	-5.654	6.293	-2.361	1.00	0.00	H
ATOM	78	HA2	GLY	A	82	-6.477	5.998	-4.937	1.00	0.00	H
ATOM	79	HA3	GLY	A	82	-5.801	7.504	-4.209	1.00	0.00	H
ATOM	80	N	GLY	A	83	-3.185	6.437	-4.727	1.00	0.00	N
ATOM	81	CA	GLY	A	83	-1.926	6.283	-5.456	1.00	0.00	C
ATOM	82	C	GLY	A	83	-1.714	4.871	-6.120	1.00	0.00	C
ATOM	83	O	GLY	A	83	-1.352	4.730	-7.282	1.00	0.00	O
ATOM	84	H	GLY	A	83	-3.127	6.678	-3.748	1.00	0.00	H
ATOM	85	HA2	GLY	A	83	-1.973	7.014	-6.263	1.00	0.00	H
ATOM	86	HA3	GLY	A	83	-1.081	6.458	-4.770	1.00	0.00	H

ATOM	87	N	CYS	A	84	-1.845	3.853	-5.314	1.00	0.00	N
ATOM	88	CA	CYS	A	84	-1.724	2.483	-5.704	1.00	0.00	C
ATOM	89	CB	CYS	A	84	-1.785	1.629	-4.469	1.00	0.00	C
ATOM	90	SG	CYS	A	84	-0.613	1.993	-3.109	1.00	0.00	S
ATOM	91	C	CYS	A	84	-2.850	2.015	-6.660	1.00	0.00	C
ATOM	92	O	CYS	A	84	-2.695	1.108	-7.509	1.00	0.00	O
ATOM	93	H	CYS	A	84	-2.050	3.904	-4.339	1.00	0.00	H
ATOM	94	HA	CYS	A	84	-0.747	2.492	-6.189	1.00	0.00	H
ATOM	95	HB2	CYS	A	84	-1.655	0.550	-4.694	1.00	0.00	H
ATOM	96	HB3	CYS	A	84	-2.787	1.711	-3.984	1.00	0.00	H
ATOM	97	HG	CYS	A	84	-1.018	3.245	-3.022	1.00	0.00	H
ATOM	98	N	THR	A	85	-4.072	2.623	-6.581	1.00	0.00	N
ATOM	99	CA	THR	A	85	-5.028	2.363	-7.668	1.00	0.00	C
ATOM	100	CB	THR	A	85	-6.506	2.679	-7.301	1.00	0.00	C
ATOM	101	OG1	THR	A	85	-6.824	1.905	-6.145	1.00	0.00	O
ATOM	102	CG2	THR	A	85	-7.548	2.125	-8.412	1.00	0.00	C
ATOM	103	C	THR	A	85	-4.662	3.118	-8.890	1.00	0.00	C
ATOM	104	O	THR	A	85	-4.798	2.551	-9.992	1.00	0.00	O
ATOM	105	H	THR	A	85	-4.175	3.366	-5.925	1.00	0.00	H
ATOM	106	HA	THR	A	85	-5.017	1.325	-7.976	1.00	0.00	H
ATOM	107	HB	THR	A	85	-6.710	3.765	-7.126	1.00	0.00	H
ATOM	108	HG21	THR	A	85	-7.378	1.039	-8.551	1.00	0.00	H
ATOM	109	HG22	THR	A	85	-7.270	2.601	-9.385	1.00	0.00	H
ATOM	110	HG23	THR	A	85	-8.578	2.344	-8.034	1.00	0.00	H
ATOM	111	HG1	THR	A	85	-6.281	2.258	-5.437	1.00	0.00	H
ATOM	112	N	GLY	A	86	-4.098	4.356	-8.816	1.00	0.00	N
ATOM	113	CA	GLY	A	86	-3.713	5.099	-10.036	1.00	0.00	C
ATOM	114	C	GLY	A	86	-2.514	4.385	-10.738	1.00	0.00	C
ATOM	115	O	GLY	A	86	-2.470	4.274	-11.917	1.00	0.00	O

ATOM	116	H	GLY	A	86	-3.940	4.775	-7.911	1.00	0.00	H
ATOM	117	HA2	GLY	A	86	-4.568	5.135	-10.712	1.00	0.00	H
ATOM	118	HA3	GLY	A	86	-3.407	6.099	-9.766	1.00	0.00	H
ATOM	119	N	GLY	A	87	-1.593	3.879	-9.931	1.00	0.00	N
ATOM	120	CA	GLY	A	87	-0.479	3.028	-10.418	1.00	0.00	C
ATOM	121	C	GLY	A	87	-1.009	1.742	-11.136	1.00	0.00	C
ATOM	122	O	GLY	A	87	-0.795	1.572	-12.338	1.00	0.00	O
ATOM	123	H	GLY	A	87	-1.652	4.081	-8.943	1.00	0.00	H
ATOM	124	HA2	GLY	A	87	0.102	3.612	-11.132	1.00	0.00	H
ATOM	125	HA3	GLY	A	87	0.133	2.722	-9.574	1.00	0.00	H
ATOM	126	N	ILE	A	88	-1.751	0.877	-10.472	1.00	0.00	N
ATOM	127	CA	ILE	A	88	-2.308	-0.278	-11.152	1.00	0.00	C
ATOM	128	C	ILE	A	88	-3.207	0.093	-12.381	1.00	0.00	C
ATOM	129	O	ILE	A	88	-3.092	-0.572	-13.408	1.00	0.00	O
ATOM	130	CB	ILE	A	88	-2.852	-1.204	-10.066	1.00	0.00	C
ATOM	131	CG2	ILE	A	88	-4.106	-0.575	-9.526	1.00	0.00	C
ATOM	132	CG1	ILE	A	88	-3.036	-2.651	-10.493	1.00	0.00	C
ATOM	133	CD1	ILE	A	88	-4.045	-2.806	-11.618	1.00	0.00	C
ATOM	134	H	ILE	A	88	-1.953	1.013	-9.486	1.00	0.00	H
ATOM	135	HA	ILE	A	88	-1.449	-0.787	-11.572	1.00	0.00	H
ATOM	136	HB	ILE	A	88	-2.127	-1.236	-9.250	1.00	0.00	H
ATOM	137	HG12	ILE	A	88	-2.088	-3.107	-10.755	1.00	0.00	H
ATOM	138	HG13	ILE	A	88	-3.345	-3.249	-9.643	1.00	0.00	H
ATOM	139	HG21	ILE	A	88	-4.853	-0.527	-10.295	1.00	0.00	H
ATOM	140	HG22	ILE	A	88	-4.478	-1.003	-8.602	1.00	0.00	H
ATOM	141	HG23	ILE	A	88	-3.899	0.453	-9.350	1.00	0.00	H
ATOM	142	HD11	ILE	A	88	-4.648	-1.936	-11.844	1.00	0.00	H
ATOM	143	HD12	ILE	A	88	-3.557	-3.125	-12.541	1.00	0.00	H
ATOM	144	HD13	ILE	A	88	-4.780	-3.522	-11.300	1.00	0.00	H

ATOM	145	N	GLY	A	89	-4.030	1.166	-12.340	1.00	0.00	N
ATOM	146	CA	GLY	A	89	-4.640	1.772	-13.497	1.00	0.00	C
ATOM	147	C	GLY	A	89	-3.691	2.091	-14.677	1.00	0.00	C
ATOM	148	O	GLY	A	89	-4.130	2.095	-15.810	1.00	0.00	O
ATOM	149	H	GLY	A	89	-4.231	1.570	-11.436	1.00	0.00	H
ATOM	150	HA2	GLY	A	89	-5.336	1.018	-13.863	1.00	0.00	H
ATOM	151	HA3	GLY	A	89	-5.137	2.721	-13.197	1.00	0.00	H
ATOM	152	HC	GLY	A	89	-2.649	2.308	-14.495	1.00	0.00	H
ATOM	153	N	GLY	A	148	-3.511	6.302	11.475	1.00	0.00	N
ATOM	154	CA	GLY	A	148	-2.768	5.715	10.359	1.00	0.00	C
ATOM	155	C	GLY	A	148	-2.903	4.180	10.334	1.00	0.00	C
ATOM	156	O	GLY	A	148	-1.944	3.481	10.380	1.00	0.00	O
ATOM	157	H1	GLY	A	148	-4.227	6.851	11.043	1.00	0.00	H
ATOM	158	H2	GLY	A	148	-2.810	6.710	12.061	1.00	0.00	H
ATOM	159	HA2	GLY	A	148	-3.182	6.115	9.433	1.00	0.00	H
ATOM	160	HA3	GLY	A	148	-1.712	5.971	10.454	1.00	0.00	H
ATOM	161	N	ILE	A	149	-4.122	3.708	10.153	1.00	0.00	N
ATOM	162	CA	ILE	A	149	-4.233	2.258	9.801	1.00	0.00	C
ATOM	163	CB	ILE	A	149	-5.405	2.008	8.759	1.00	0.00	C
ATOM	164	CG2	ILE	A	149	-5.311	3.046	7.597	1.00	0.00	C
ATOM	165	CG1	ILE	A	149	-5.297	0.562	8.101	1.00	0.00	C
ATOM	166	CD1	ILE	A	149	-5.993	-0.570	8.842	1.00	0.00	C
ATOM	167	C	ILE	A	149	-4.393	1.372	11.002	1.00	0.00	C
ATOM	168	O	ILE	A	149	-3.791	0.321	11.069	1.00	0.00	O
ATOM	169	H	ILE	A	149	-4.912	4.320	10.245	1.00	0.00	H
ATOM	170	HA	ILE	A	149	-3.400	1.836	9.279	1.00	0.00	H
ATOM	171	HB	ILE	A	149	-6.384	2.057	9.286	1.00	0.00	H
ATOM	172	HG12	ILE	A	149	-4.215	0.309	8.112	1.00	0.00	H
ATOM	173	HG13	ILE	A	149	-5.590	0.614	7.039	1.00	0.00	H

ATOM	174	HG21	ILE	A	149	-4.336	3.177	7.107	1.00	0.00	H
ATOM	175	HG22	ILE	A	149	-5.568	4.067	7.924	1.00	0.00	H
ATOM	176	HG23	ILE	A	149	-6.024	2.819	6.773	1.00	0.00	H
ATOM	177	HD11	ILE	A	149	-5.507	-0.770	9.821	1.00	0.00	H
ATOM	178	HD12	ILE	A	149	-5.993	-1.479	8.196	1.00	0.00	H
ATOM	179	HD13	ILE	A	149	-7.033	-0.346	9.138	1.00	0.00	H
ATOM	180	N	GLY	A	150	-5.198	1.736	12.019	1.00	0.00	N
ATOM	181	CA	GLY	A	150	-5.370	1.006	13.317	1.00	0.00	C
ATOM	182	C	GLY	A	150	-6.860	0.699	13.715	1.00	0.00	C
ATOM	183	O	GLY	A	150	-7.212	0.377	14.887	1.00	0.00	O
ATOM	184	H	GLY	A	150	-5.737	2.581	11.895	1.00	0.00	H
ATOM	185	HA2	GLY	A	150	-4.884	0.040	13.183	1.00	0.00	H
ATOM	186	HA3	GLY	A	150	-4.918	1.607	14.118	1.00	0.00	H
ATOM	187	HC	GLY	A	150	-7.620	0.766	12.951	1.00	0.00	H
ATOM	188	N	GLY	A	158	-12.349	-4.688	2.269	1.00	0.00	N
ATOM	189	CA	GLY	A	158	-12.680	-3.914	1.063	1.00	0.00	C
ATOM	190	C	GLY	A	158	-11.473	-3.517	0.210	1.00	0.00	C
ATOM	191	O	GLY	A	158	-11.392	-3.618	-1.025	1.00	0.00	O
ATOM	192	H1	GLY	A	158	-12.742	-5.581	2.112	1.00	0.00	H
ATOM	193	H2	GLY	A	158	-12.587	-4.095	3.023	1.00	0.00	H
ATOM	194	HA2	GLY	A	158	-13.292	-4.565	0.439	1.00	0.00	H
ATOM	195	HA3	GLY	A	158	-13.215	-2.997	1.369	1.00	0.00	H
ATOM	196	N	SER	A	159	-10.402	-3.095	0.936	1.00	0.00	N
ATOM	197	CA	SER	A	159	-9.066	-2.738	0.329	1.00	0.00	C
ATOM	198	CB	SER	A	159	-8.108	-2.380	1.451	1.00	0.00	C
ATOM	199	OG	SER	A	159	-6.931	-1.795	0.866	1.00	0.00	O
ATOM	200	C	SER	A	159	-8.408	-3.902	-0.389	1.00	0.00	C
ATOM	201	O	SER	A	159	-7.949	-3.787	-1.548	1.00	0.00	O
ATOM	202	H	SER	A	159	-10.502	-2.960	1.918	1.00	0.00	H

ATOM	203	HA	SER	A	159	-9.229	-1.874	-0.297	1.00	0.00	H
ATOM	204	HB2	SER	A	159	-7.887	-3.249	2.095	1.00	0.00	H
ATOM	205	HB3	SER	A	159	-8.490	-1.571	2.117	1.00	0.00	H
ATOM	206	HG	SER	A	159	-6.739	-1.150	1.544	1.00	0.00	H
ATOM	207	N	GLY	A	160	-8.484	-5.096	0.284	1.00	0.00	N
ATOM	208	CA	GLY	A	160	-8.106	-6.334	-0.240	1.00	0.00	C
ATOM	209	C	GLY	A	160	-8.990	-6.720	-1.485	1.00	0.00	C
ATOM	210	O	GLY	A	160	-8.475	-6.742	-2.593	1.00	0.00	O
ATOM	211	H	GLY	A	160	-8.840	-5.078	1.229	1.00	0.00	H
ATOM	212	HA2	GLY	A	160	-7.059	-6.234	-0.525	1.00	0.00	H
ATOM	213	HA3	GLY	A	160	-8.228	-7.135	0.496	1.00	0.00	H
ATOM	214	HC	GLY	A	160	-10.035	-6.962	-1.361	1.00	0.00	H
ATOM	215	N	GLY	A	162	-11.689	-5.318	-3.488	1.00	0.00	N
ATOM	216	CA	GLY	A	162	-12.068	-4.398	-4.552	1.00	0.00	C
ATOM	217	C	GLY	A	162	-10.927	-3.458	-4.984	1.00	0.00	C
ATOM	218	O	GLY	A	162	-10.555	-3.386	-6.196	1.00	0.00	O
ATOM	219	H1	GLY	A	162	-11.730	-6.222	-3.901	1.00	0.00	H
ATOM	220	H2	GLY	A	162	-12.243	-5.051	-2.706	1.00	0.00	H
ATOM	221	HA2	GLY	A	162	-12.331	-5.011	-5.414	1.00	0.00	H
ATOM	222	HA3	GLY	A	162	-12.915	-3.783	-4.214	1.00	0.00	H
ATOM	223	N	SER	A	163	-10.307	-2.779	-4.071	1.00	0.00	N
ATOM	224	CA	SER	A	163	-9.201	-1.809	-4.446	1.00	0.00	C
ATOM	225	CB	SER	A	163	-8.727	-0.802	-3.324	1.00	0.00	C
ATOM	226	OG	SER	A	163	-9.760	-0.003	-2.698	1.00	0.00	O
ATOM	227	C	SER	A	163	-7.909	-2.429	-4.951	1.00	0.00	C
ATOM	228	O	SER	A	163	-7.295	-1.877	-5.819	1.00	0.00	O
ATOM	229	H	SER	A	163	-10.598	-2.842	-3.111	1.00	0.00	H
ATOM	230	HA	SER	A	163	-9.623	-1.222	-5.248	1.00	0.00	H
ATOM	231	HB2	SER	A	163	-8.066	-0.071	-3.825	1.00	0.00	H

ATOM	232	HB3	SER	A	163	-8.200	-1.403	-2.570	1.00	0.00	H
ATOM	233	HG	SER	A	163	-10.379	0.229	-3.389	1.00	0.00	H
ATOM	234	N	PHE	A	164	-7.521	-3.566	-4.329	1.00	0.00	N
ATOM	235	CA	PHE	A	164	-6.321	-4.327	-4.721	1.00	0.00	C
ATOM	236	CB	PHE	A	164	-5.625	-4.769	-3.354	1.00	0.00	C
ATOM	237	CG	PHE	A	164	-4.573	-5.853	-3.526	1.00	0.00	C
ATOM	238	CD1	PHE	A	164	-3.497	-5.668	-4.404	1.00	0.00	C
ATOM	239	CE1	PHE	A	164	-2.599	-6.721	-4.624	1.00	0.00	C
ATOM	240	CZ	PHE	A	164	-2.607	-7.901	-3.922	1.00	0.00	C
ATOM	241	CD2	PHE	A	164	-4.564	-7.025	-2.791	1.00	0.00	C
ATOM	242	CE2	PHE	A	164	-3.578	-8.069	-2.899	1.00	0.00	C
ATOM	243	C	PHE	A	164	-6.721	-5.496	-5.710	1.00	0.00	C
ATOM	244	O	PHE	A	164	-6.049	-5.752	-6.738	1.00	0.00	O
ATOM	245	H	PHE	A	164	-8.112	-3.979	-3.654	1.00	0.00	H
ATOM	246	HA	PHE	A	164	-5.601	-3.805	-5.315	1.00	0.00	H
ATOM	247	HB2	PHE	A	164	-6.326	-5.257	-2.645	1.00	0.00	H
ATOM	248	HB3	PHE	A	164	-5.253	-3.843	-2.853	1.00	0.00	H
ATOM	249	HD1	PHE	A	164	-3.380	-4.717	-4.881	1.00	0.00	H
ATOM	250	HD2	PHE	A	164	-5.375	-7.206	-2.102	1.00	0.00	H
ATOM	251	HE1	PHE	A	164	-1.701	-6.493	-5.179	1.00	0.00	H
ATOM	252	HE2	PHE	A	164	-3.587	-8.963	-2.285	1.00	0.00	H
ATOM	253	HZ	PHE	A	164	-1.850	-8.668	-4.076	1.00	0.00	H
ATOM	254	N	GLY	A	165	-7.859	-6.145	-5.486	1.00	0.00	N
ATOM	255	CA	GLY	A	165	-8.241	-7.262	-6.339	1.00	0.00	C
ATOM	256	C	GLY	A	165	-8.609	-7.002	-7.845	1.00	0.00	C
ATOM	257	O	GLY	A	165	-8.271	-7.725	-8.774	1.00	0.00	O
ATOM	258	H	GLY	A	165	-8.459	-5.863	-4.724	1.00	0.00	H
ATOM	259	HA2	GLY	A	165	-7.314	-7.833	-6.390	1.00	0.00	H
ATOM	260	HA3	GLY	A	165	-9.118	-7.790	-5.873	1.00	0.00	H

ATOM	261	N	GLY	A	166	-9.342	-5.882	-8.127	1.00	0.00	N
ATOM	262	CA	GLY	A	166	-9.861	-5.563	-9.437	1.00	0.00	C
ATOM	263	C	GLY	A	166	-9.671	-4.099	-9.821	1.00	0.00	C
ATOM	264	O	GLY	A	166	-10.610	-3.428	-10.309	1.00	0.00	O
ATOM	265	H	GLY	A	166	-9.532	-5.238	-7.373	1.00	0.00	H
ATOM	266	HA2	GLY	A	166	-9.298	-6.164	-10.151	1.00	0.00	H
ATOM	267	HA3	GLY	A	166	-10.938	-5.797	-9.469	1.00	0.00	H
ATOM	268	N	PRO	A	167	-8.498	-3.476	-9.599	1.00	0.00	N
ATOM	269	CD	PRO	A	167	-8.453	-2.048	-9.720	1.00	0.00	C
ATOM	270	CA	PRO	A	167	-7.240	-4.021	-9.186	1.00	0.00	C
ATOM	271	CB	PRO	A	167	-6.392	-2.749	-8.934	1.00	0.00	C
ATOM	272	CG	PRO	A	167	-6.960	-1.841	-9.941	1.00	0.00	C
ATOM	273	C	PRO	A	167	-6.654	-4.945	-10.185	1.00	0.00	C
ATOM	274	O	PRO	A	167	-6.967	-4.995	-11.385	1.00	0.00	O
ATOM	275	HA	PRO	A	167	-7.455	-4.549	-8.258	1.00	0.00	H
ATOM	276	HB2	PRO	A	167	-5.314	-2.932	-9.102	1.00	0.00	H
ATOM	277	HB3	PRO	A	167	-6.476	-2.306	-7.916	1.00	0.00	H
ATOM	278	HG2	PRO	A	167	-6.750	-0.754	-9.856	1.00	0.00	H
ATOM	279	HG3	PRO	A	167	-6.719	-2.203	-10.967	1.00	0.00	H
ATOM	280	HD2	PRO	A	167	-8.850	-1.777	-8.708	1.00	0.00	H
ATOM	281	HD3	PRO	A	167	-9.030	-1.619	-10.578	1.00	0.00	H
ATOM	282	N	PHE	A	168	-5.762	-5.796	-9.660	1.00	0.00	N
ATOM	283	CA	PHE	A	168	-4.905	-6.686	-10.462	1.00	0.00	C
ATOM	284	CB	PHE	A	168	-3.466	-6.012	-10.911	1.00	0.00	C
ATOM	285	CG	PHE	A	168	-2.438	-6.891	-11.530	1.00	0.00	C
ATOM	286	CD1	PHE	A	168	-1.827	-6.601	-12.773	1.00	0.00	C
ATOM	287	CE1	PHE	A	168	-0.873	-7.404	-13.409	1.00	0.00	C
ATOM	288	CZ	PHE	A	168	-0.314	-8.449	-12.651	1.00	0.00	C
ATOM	289	CD2	PHE	A	168	-1.918	-7.978	-10.890	1.00	0.00	C

ATOM	290	CE2	PHE	A	168	-0.871	-8.804	-11.394	1.00	0.00	C
ATOM	291	C	PHE	A	168	-5.618	-7.513	-11.515	1.00	0.00	C
ATOM	292	O	PHE	A	168	-5.065	-7.737	-12.627	1.00	0.00	O
ATOM	293	H	PHE	A	168	-5.725	-5.730	-8.664	1.00	0.00	H
ATOM	294	HA	PHE	A	168	-4.519	-7.416	-9.772	1.00	0.00	H
ATOM	295	HB2	PHE	A	168	-3.002	-5.565	-9.997	1.00	0.00	H
ATOM	296	HB3	PHE	A	168	-3.723	-5.189	-11.611	1.00	0.00	H
ATOM	297	HD1	PHE	A	168	-2.177	-5.768	-13.364	1.00	0.00	H
ATOM	298	HD2	PHE	A	168	-2.297	-8.346	-9.945	1.00	0.00	H
ATOM	299	HE1	PHE	A	168	-0.649	-7.263	-14.444	1.00	0.00	H
ATOM	300	HE2	PHE	A	168	-0.474	-9.563	-10.724	1.00	0.00	H
ATOM	301	HZ	PHE	A	168	0.538	-8.969	-13.044	1.00	0.00	H
ATOM	302	N	GLY	A	169	-6.855	-8.077	-11.259	1.00	0.00	N
ATOM	303	CA	GLY	A	169	-7.567	-8.990	-12.204	1.00	0.00	C
ATOM	304	C	GLY	A	169	-7.970	-8.213	-13.399	1.00	0.00	C
ATOM	305	O	GLY	A	169	-7.999	-8.889	-14.441	1.00	0.00	O
ATOM	306	H	GLY	A	169	-7.304	-7.859	-10.381	1.00	0.00	H
ATOM	307	HA2	GLY	A	169	-6.927	-9.813	-12.523	1.00	0.00	H
ATOM	308	HA3	GLY	A	169	-8.435	-9.388	-11.693	1.00	0.00	H
ATOM	309	HC	GLY	A	169	-8.215	-7.162	-13.368	1.00	0.00	H
ATOM	310	N	GLY	A	212	7.171	-7.198	-9.860	1.00	0.00	N
ATOM	311	CA	GLY	A	212	7.597	-6.686	-8.595	1.00	0.00	C
ATOM	312	C	GLY	A	212	6.525	-6.150	-7.704	1.00	0.00	C
ATOM	313	O	GLY	A	212	6.374	-6.473	-6.527	1.00	0.00	O
ATOM	314	H1	GLY	A	212	7.346	-8.178	-9.843	1.00	0.00	H
ATOM	315	H2	GLY	A	212	7.548	-6.591	-10.553	1.00	0.00	H
ATOM	316	HA2	GLY	A	212	8.003	-7.547	-8.063	1.00	0.00	H
ATOM	317	HA3	GLY	A	212	8.354	-5.880	-8.771	1.00	0.00	H
ATOM	318	N	PHE	A	213	5.576	-5.431	-8.333	1.00	0.00	N

ATOM	319	CA	PHE	A	213	4.515	-4.677	-7.582	1.00	0.00	C
ATOM	320	CB	PHE	A	213	3.901	-3.628	-8.558	1.00	0.00	C
ATOM	321	CG	PHE	A	213	2.965	-2.772	-7.787	1.00	0.00	C
ATOM	322	CD1	PHE	A	213	3.560	-1.836	-6.784	1.00	0.00	C
ATOM	323	CE1	PHE	A	213	2.701	-0.832	-6.239	1.00	0.00	C
ATOM	324	CZ	PHE	A	213	1.362	-0.729	-6.550	1.00	0.00	C
ATOM	325	CD2	PHE	A	213	1.678	-2.560	-8.137	1.00	0.00	C
ATOM	326	CE2	PHE	A	213	0.903	-1.637	-7.494	1.00	0.00	C
ATOM	327	C	PHE	A	213	3.533	-5.670	-6.900	1.00	0.00	C
ATOM	328	O	PHE	A	213	3.210	-5.551	-5.744	1.00	0.00	O
ATOM	329	H	PHE	A	213	5.768	-5.103	-9.252	1.00	0.00	H
ATOM	330	HA	PHE	A	213	4.947	-4.050	-6.814	1.00	0.00	H
ATOM	331	HB2	PHE	A	213	3.393	-4.147	-9.390	1.00	0.00	H
ATOM	332	HB3	PHE	A	213	4.734	-3.058	-9.055	1.00	0.00	H
ATOM	333	HD1	PHE	A	213	4.628	-1.846	-6.603	1.00	0.00	H
ATOM	334	HD2	PHE	A	213	1.154	-3.008	-8.968	1.00	0.00	H
ATOM	335	HE1	PHE	A	213	3.225	-0.123	-5.608	1.00	0.00	H
ATOM	336	HE2	PHE	A	213	-0.135	-1.542	-7.789	1.00	0.00	H
ATOM	337	HZ	PHE	A	213	0.738	-0.019	-6.019	1.00	0.00	H
ATOM	338	N	ILE	A	214	3.051	-6.614	-7.677	1.00	0.00	N
ATOM	339	CA	ILE	A	214	2.053	-7.621	-7.291	1.00	0.00	C
ATOM	340	CB	ILE	A	214	0.579	-7.140	-7.198	1.00	0.00	C
ATOM	341	CG2	ILE	A	214	-0.262	-8.432	-7.269	1.00	0.00	C
ATOM	342	CG1	ILE	A	214	0.127	-6.105	-8.265	1.00	0.00	C
ATOM	343	CD1	ILE	A	214	-1.091	-5.245	-7.842	1.00	0.00	C
ATOM	344	C	ILE	A	214	2.630	-8.509	-6.090	1.00	0.00	C
ATOM	345	O	ILE	A	214	1.946	-8.881	-5.132	1.00	0.00	O
ATOM	346	H	ILE	A	214	3.473	-6.611	-8.584	1.00	0.00	H
ATOM	347	HA	ILE	A	214	2.039	-8.299	-8.120	1.00	0.00	H

ATOM	348	HB	ILE	A	214	0.425	-6.610	-6.225	1.00	0.00	H
ATOM	349	HG12	ILE	A	214	0.031	-6.610	-9.248	1.00	0.00	H
ATOM	350	HG13	ILE	A	214	1.009	-5.444	-8.460	1.00	0.00	H
ATOM	351	HG21	ILE	A	214	-1.309	-8.131	-7.464	1.00	0.00	H
ATOM	352	HG22	ILE	A	214	-0.288	-9.128	-6.406	1.00	0.00	H
ATOM	353	HG23	ILE	A	214	0.084	-8.904	-8.216	1.00	0.00	H
ATOM	354	HD11	ILE	A	214	-0.858	-4.644	-6.939	1.00	0.00	H
ATOM	355	HD12	ILE	A	214	-1.941	-5.902	-7.582	1.00	0.00	H
ATOM	356	HD13	ILE	A	214	-1.481	-4.522	-8.583	1.00	0.00	H
ATOM	357	N	GLY	A	215	3.953	-8.781	-6.162	1.00	0.00	N
ATOM	358	CA	GLY	A	215	4.725	-9.617	-5.217	1.00	0.00	C
ATOM	359	C	GLY	A	215	4.791	-9.040	-3.862	1.00	0.00	C
ATOM	360	O	GLY	A	215	4.801	-9.694	-2.862	1.00	0.00	O
ATOM	361	H	GLY	A	215	4.462	-8.375	-6.934	1.00	0.00	H
ATOM	362	HA2	GLY	A	215	4.221	-10.577	-5.109	1.00	0.00	H
ATOM	363	HA3	GLY	A	215	5.740	-9.741	-5.630	1.00	0.00	H
ATOM	364	N	GLY	A	216	4.887	-7.685	-3.779	1.00	0.00	N
ATOM	365	CA	GLY	A	216	4.873	-6.876	-2.554	1.00	0.00	C
ATOM	366	C	GLY	A	216	3.523	-6.776	-2.019	1.00	0.00	C
ATOM	367	O	GLY	A	216	3.348	-6.648	-0.825	1.00	0.00	O
ATOM	368	H	GLY	A	216	4.977	-7.184	-4.651	1.00	0.00	H
ATOM	369	HA2	GLY	A	216	5.490	-7.345	-1.787	1.00	0.00	H
ATOM	370	HA3	GLY	A	216	5.270	-5.883	-2.800	1.00	0.00	H
ATOM	371	N	TRP	A	217	2.486	-6.831	-2.906	1.00	0.00	N
ATOM	372	CA	TRP	A	217	1.120	-7.071	-2.580	1.00	0.00	C
ATOM	373	CB	TRP	A	217	0.460	-5.826	-1.889	1.00	0.00	C
ATOM	374	CG	TRP	A	217	0.879	-4.504	-2.493	1.00	0.00	C
ATOM	375	CD1	TRP	A	217	1.019	-4.201	-3.844	1.00	0.00	C
ATOM	376	NE1	TRP	A	217	1.434	-2.922	-4.018	1.00	0.00	N

ATOM	377	CE2	TRP	A	217	1.714	-2.429	-2.811	1.00	0.00	C
ATOM	378	CD2	TRP	A	217	1.444	-3.378	-1.818	1.00	0.00	C
ATOM	379	CE3	TRP	A	217	1.715	-3.132	-0.493	1.00	0.00	C
ATOM	380	CZ3	TRP	A	217	2.342	-1.962	-0.142	1.00	0.00	C
ATOM	381	CZ2	TRP	A	217	2.376	-1.313	-2.438	1.00	0.00	C
ATOM	382	CH2	TRP	A	217	2.769	-1.092	-1.139	1.00	0.00	C
ATOM	383	C	TRP	A	217	0.837	-8.367	-1.836	1.00	0.00	C
ATOM	384	O	TRP	A	217	0.219	-8.366	-0.803	1.00	0.00	O
ATOM	385	H	TRP	A	217	2.743	-6.826	-3.872	1.00	0.00	H
ATOM	386	HA	TRP	A	217	0.556	-7.172	-3.491	1.00	0.00	H
ATOM	387	HB2	TRP	A	217	-0.641	-5.926	-2.018	1.00	0.00	H
ATOM	388	HB3	TRP	A	217	0.661	-5.844	-0.796	1.00	0.00	H
ATOM	389	HD1	TRP	A	217	0.595	-4.849	-4.601	1.00	0.00	H
ATOM	390	HE1	TRP	A	217	1.684	-2.413	-4.821	1.00	0.00	H
ATOM	391	HE3	TRP	A	217	1.496	-3.842	0.285	1.00	0.00	H
ATOM	392	HZ2	TRP	A	217	2.484	-0.548	-3.187	1.00	0.00	H
ATOM	393	HZ3	TRP	A	217	2.471	-1.764	0.912	1.00	0.00	H
ATOM	394	HH2	TRP	A	217	3.385	-0.225	-0.933	1.00	0.00	H
ATOM	395	N	GLY	A	218	1.364	-9.556	-2.319	1.00	0.00	N
ATOM	396	CA	GLY	A	218	1.230	-10.922	-1.752	1.00	0.00	C
ATOM	397	C	GLY	A	218	2.551	-11.702	-1.313	1.00	0.00	C
ATOM	398	O	GLY	A	218	2.628	-12.815	-1.787	1.00	0.00	O
ATOM	399	H	GLY	A	218	1.909	-9.483	-3.166	1.00	0.00	H
ATOM	400	HA2	GLY	A	218	0.693	-10.744	-0.820	1.00	0.00	H
ATOM	401	HA3	GLY	A	218	0.701	-11.566	-2.492	1.00	0.00	H
ATOM	402	N	GLY	A	219	3.503	-11.271	-0.440	1.00	0.00	N
ATOM	403	CA	GLY	A	219	3.685	-9.974	0.075	1.00	0.00	C
ATOM	404	C	GLY	A	219	2.790	-9.541	1.205	1.00	0.00	C
ATOM	405	O	GLY	A	219	2.344	-10.371	2.040	1.00	0.00	O

ATOM	406	H	GLY	A	219	4.160	-11.969	-0.123	1.00	0.00	H
ATOM	407	HA2	GLY	A	219	4.684	-10.011	0.508	1.00	0.00	H
ATOM	408	HA3	GLY	A	219	3.575	-9.225	-0.760	1.00	0.00	H
ATOM	409	N	PHE	A	220	2.500	-8.248	1.359	1.00	0.00	N
ATOM	410	CA	PHE	A	220	2.061	-7.509	2.468	1.00	0.00	C
ATOM	411	CB	PHE	A	220	2.133	-6.037	2.059	1.00	0.00	C
ATOM	412	CG	PHE	A	220	1.536	-5.040	3.019	1.00	0.00	C
ATOM	413	CD1	PHE	A	220	0.234	-4.630	2.737	1.00	0.00	C
ATOM	414	CE1	PHE	A	220	-0.453	-3.764	3.619	1.00	0.00	C
ATOM	415	CZ	PHE	A	220	0.215	-3.397	4.888	1.00	0.00	C
ATOM	416	CD2	PHE	A	220	2.056	-4.887	4.320	1.00	0.00	C
ATOM	417	CE2	PHE	A	220	1.489	-3.907	5.192	1.00	0.00	C
ATOM	418	C	PHE	A	220	0.707	-7.917	2.939	1.00	0.00	C
ATOM	419	O	PHE	A	220	0.448	-8.004	4.123	1.00	0.00	O
ATOM	420	H	PHE	A	220	2.758	-7.584	0.654	1.00	0.00	H
ATOM	421	HA	PHE	A	220	2.774	-7.651	3.265	1.00	0.00	H
ATOM	422	HB2	PHE	A	220	1.691	-5.800	1.068	1.00	0.00	H
ATOM	423	HB3	PHE	A	220	3.206	-5.760	1.921	1.00	0.00	H
ATOM	424	HD1	PHE	A	220	-0.186	-4.796	1.770	1.00	0.00	H
ATOM	425	HD2	PHE	A	220	3.015	-5.371	4.477	1.00	0.00	H
ATOM	426	HE1	PHE	A	220	-1.451	-3.413	3.371	1.00	0.00	H
ATOM	427	HE2	PHE	A	220	2.031	-3.796	6.111	1.00	0.00	H
ATOM	428	HZ	PHE	A	220	-0.221	-2.611	5.495	1.00	0.00	H
ATOM	429	N	PHE	A	221	-0.202	-8.277	1.994	1.00	0.00	N
ATOM	430	CA	PHE	A	221	-1.563	-8.885	2.253	1.00	0.00	C
ATOM	431	CB	PHE	A	221	-2.631	-7.864	2.693	1.00	0.00	C
ATOM	432	CG	PHE	A	221	-2.936	-6.717	1.809	1.00	0.00	C
ATOM	433	CD1	PHE	A	221	-3.690	-5.707	2.317	1.00	0.00	C
ATOM	434	CE1	PHE	A	221	-4.018	-4.594	1.520	1.00	0.00	C

ATOM	435	CZ	PHE	A	221	-3.532	-4.405	0.257	1.00	0.00	C
ATOM	436	CD2	PHE	A	221	-2.503	-6.634	0.515	1.00	0.00	C
ATOM	437	CE2	PHE	A	221	-2.804	-5.445	-0.225	1.00	0.00	C
ATOM	438	C	PHE	A	221	-1.594	-10.073	3.144	1.00	0.00	C
ATOM	439	O	PHE	A	221	-2.564	-10.336	3.860	1.00	0.00	O
ATOM	440	H	PHE	A	221	0.189	-8.122	1.091	1.00	0.00	H
ATOM	441	HA	PHE	A	221	-1.809	-9.230	1.259	1.00	0.00	H
ATOM	442	HB2	PHE	A	221	-3.597	-8.332	2.926	1.00	0.00	H
ATOM	443	HB3	PHE	A	221	-2.184	-7.385	3.596	1.00	0.00	H
ATOM	444	HD1	PHE	A	221	-4.183	-5.789	3.272	1.00	0.00	H
ATOM	445	HD2	PHE	A	221	-1.906	-7.385	0.020	1.00	0.00	H
ATOM	446	HE1	PHE	A	221	-4.517	-3.724	1.932	1.00	0.00	H
ATOM	447	HE2	PHE	A	221	-2.366	-5.338	-1.205	1.00	0.00	H
ATOM	448	HZ	PHE	A	221	-3.712	-3.597	-0.424	1.00	0.00	H
ATOM	449	N	GLY	A	222	-0.593	-10.920	3.097	1.00	0.00	N
ATOM	450	CA	GLY	A	222	-0.586	-12.059	3.954	1.00	0.00	C
ATOM	451	C	GLY	A	222	-0.397	-11.701	5.345	1.00	0.00	C
ATOM	452	O	GLY	A	222	-1.118	-12.069	6.279	1.00	0.00	O
ATOM	453	H	GLY	A	222	0.171	-10.765	2.455	1.00	0.00	H
ATOM	454	HA2	GLY	A	222	-1.559	-12.546	3.884	1.00	0.00	H
ATOM	455	HA3	GLY	A	222	0.219	-12.746	3.624	1.00	0.00	H
ATOM	456	N	GLY	A	223	0.472	-10.770	5.575	1.00	0.00	N
ATOM	457	CA	GLY	A	223	0.723	-10.129	6.855	1.00	0.00	C
ATOM	458	C	GLY	A	223	-0.421	-9.315	7.316	1.00	0.00	C
ATOM	459	O	GLY	A	223	-0.718	-9.321	8.529	1.00	0.00	O
ATOM	460	H	GLY	A	223	1.028	-10.460	4.791	1.00	0.00	H
ATOM	461	HA2	GLY	A	223	0.875	-10.908	7.602	1.00	0.00	H
ATOM	462	HA3	GLY	A	223	1.620	-9.494	6.757	1.00	0.00	H
ATOM	463	N	HIS	A	224	-1.025	-8.514	6.461	1.00	0.00	N

ATOM	464	CA	HIS	A	224	-2.075	-7.627	6.765	1.00	0.00	C
ATOM	465	CB	HIS	A	224	-1.494	-6.349	7.399	1.00	0.00	C
ATOM	466	ND1	HIS	A	224	-3.143	-4.783	8.598	1.00	0.00	N
ATOM	467	CG	HIS	A	224	-2.589	-5.226	7.446	1.00	0.00	C
ATOM	468	CE1	HIS	A	224	-3.947	-3.766	8.263	1.00	0.00	C
ATOM	469	NE2	HIS	A	224	-3.975	-3.539	7.019	1.00	0.00	N
ATOM	470	CD2	HIS	A	224	-3.073	-4.394	6.456	1.00	0.00	C
ATOM	471	C	HIS	A	224	-3.335	-8.310	7.407	1.00	0.00	C
ATOM	472	O	HIS	A	224	-3.799	-7.888	8.468	1.00	0.00	O
ATOM	473	H	HIS	A	224	-0.754	-8.580	5.513	1.00	0.00	H
ATOM	474	HA	HIS	A	224	-2.517	-7.302	5.828	1.00	0.00	H
ATOM	475	HB2	HIS	A	224	-0.613	-6.027	6.785	1.00	0.00	H
ATOM	476	HB3	HIS	A	224	-1.092	-6.506	8.419	1.00	0.00	H
ATOM	477	HD1	HIS	A	224	-3.017	-5.218	9.502	1.00	0.00	H
ATOM	478	HD2	HIS	A	224	-2.749	-4.399	5.427	1.00	0.00	H
ATOM	479	HE1	HIS	A	224	-4.588	-3.276	8.993	1.00	0.00	H
ATOM	480	N	GLY	A	225	-3.888	-9.377	6.736	1.00	0.00	N
ATOM	481	CA	GLY	A	225	-4.860	-10.332	7.269	1.00	0.00	C
ATOM	482	C	GLY	A	225	-4.381	-11.116	8.503	1.00	0.00	C
ATOM	483	O	GLY	A	225	-5.171	-11.222	9.480	1.00	0.00	O
ATOM	484	H	GLY	A	225	-3.593	-9.515	5.780	1.00	0.00	H
ATOM	485	HA2	GLY	A	225	-5.715	-9.739	7.593	1.00	0.00	H
ATOM	486	HA3	GLY	A	225	-5.122	-11.055	6.476	1.00	0.00	H
ATOM	487	HC	GLY	A	225	-3.398	-11.562	8.532	1.00	0.00	H
ATOM	488	N	GLY	A	238	9.172	-6.774	8.447	1.00	0.00	N
ATOM	489	CA	GLY	A	238	8.840	-7.022	7.043	1.00	0.00	C
ATOM	490	C	GLY	A	238	8.071	-5.833	6.428	1.00	0.00	C
ATOM	491	O	GLY	A	238	8.382	-5.304	5.370	1.00	0.00	O
ATOM	492	H1	GLY	A	238	10.172	-6.737	8.469	1.00	0.00	H

ATOM	493	H2	GLY	A	238	8.657	-7.465	8.956	1.00	0.00	H
ATOM	494	HA2	GLY	A	238	9.773	-7.156	6.495	1.00	0.00	H
ATOM	495	HA3	GLY	A	238	8.217	-7.914	6.971	1.00	0.00	H
ATOM	496	N	TYR	A	239	7.045	-5.360	7.150	1.00	0.00	N
ATOM	497	CA	TYR	A	239	6.198	-4.212	6.826	1.00	0.00	C
ATOM	498	CB	TYR	A	239	4.762	-4.662	6.448	1.00	0.00	C
ATOM	499	CG	TYR	A	239	4.024	-5.059	7.761	1.00	0.00	C
ATOM	500	CD1	TYR	A	239	3.552	-4.067	8.686	1.00	0.00	C
ATOM	501	CE1	TYR	A	239	2.978	-4.430	9.920	1.00	0.00	C
ATOM	502	CZ	TYR	A	239	2.838	-5.732	10.227	1.00	0.00	C
ATOM	503	OH	TYR	A	239	2.362	-6.179	11.440	1.00	0.00	O
ATOM	504	CD2	TYR	A	239	3.780	-6.405	8.074	1.00	0.00	C
ATOM	505	CE2	TYR	A	239	3.189	-6.770	9.356	1.00	0.00	C
ATOM	506	C	TYR	A	239	6.748	-3.197	5.810	1.00	0.00	C
ATOM	507	O	TYR	A	239	6.249	-2.980	4.699	1.00	0.00	O
ATOM	508	H	TYR	A	239	6.940	-5.763	8.067	1.00	0.00	H
ATOM	509	HA	TYR	A	239	6.203	-3.659	7.749	1.00	0.00	H
ATOM	510	HB2	TYR	A	239	4.878	-5.579	5.835	1.00	0.00	H
ATOM	511	HB3	TYR	A	239	4.197	-3.812	5.973	1.00	0.00	H
ATOM	512	HD1	TYR	A	239	3.555	-3.003	8.472	1.00	0.00	H
ATOM	513	HD2	TYR	A	239	4.137	-7.259	7.504	1.00	0.00	H
ATOM	514	HE1	TYR	A	239	2.578	-3.648	10.552	1.00	0.00	H
ATOM	515	HE2	TYR	A	239	3.022	-7.812	9.531	1.00	0.00	H
ATOM	516	HH	TYR	A	239	2.793	-6.990	11.708	1.00	0.00	H
ATOM	517	N	GLY	A	240	7.866	-2.436	6.143	1.00	0.00	N
ATOM	518	CA	GLY	A	240	8.524	-1.437	5.310	1.00	0.00	C
ATOM	519	C	GLY	A	240	9.267	-1.917	4.079	1.00	0.00	C
ATOM	520	O	GLY	A	240	9.380	-1.229	3.075	1.00	0.00	O
ATOM	521	H	GLY	A	240	8.263	-2.593	7.058	1.00	0.00	H

ATOM	522	HA2	GLY	A	240	7.703	-0.844	4.906	1.00	0.00	H
ATOM	523	HA3	GLY	A	240	9.240	-0.861	5.954	1.00	0.00	H
ATOM	524	HC	GLY	A	240	9.712	-2.901	4.089	1.00	0.00	H
ATOM	525	N	GLY	A	242	8.506	-4.536	2.052	1.00	0.00	N
ATOM	526	CA	GLY	A	242	7.460	-4.614	1.053	1.00	0.00	C
ATOM	527	C	GLY	A	242	6.930	-3.307	0.613	1.00	0.00	C
ATOM	528	O	GLY	A	242	6.715	-3.092	-0.594	1.00	0.00	O
ATOM	529	H1	GLY	A	242	9.334	-4.884	1.614	1.00	0.00	H
ATOM	530	H2	GLY	A	242	8.129	-4.946	2.881	1.00	0.00	H
ATOM	531	HA2	GLY	A	242	7.905	-5.061	0.164	1.00	0.00	H
ATOM	532	HA3	GLY	A	242	6.631	-5.233	1.464	1.00	0.00	H
ATOM	533	N	THR	A	243	6.780	-2.407	1.583	1.00	0.00	N
ATOM	534	CA	THR	A	243	6.308	-1.040	1.274	1.00	0.00	C
ATOM	535	CB	THR	A	243	5.945	-0.209	2.468	1.00	0.00	C
ATOM	536	OG1	THR	A	243	5.073	-1.013	3.262	1.00	0.00	O
ATOM	537	CG2	THR	A	243	5.243	1.078	2.026	1.00	0.00	C
ATOM	538	C	THR	A	243	7.194	-0.246	0.279	1.00	0.00	C
ATOM	539	O	THR	A	243	6.748	0.315	-0.748	1.00	0.00	O
ATOM	540	H	THR	A	243	6.997	-2.560	2.551	1.00	0.00	H
ATOM	541	HA	THR	A	243	5.338	-1.111	0.829	1.00	0.00	H
ATOM	542	HB	THR	A	243	6.877	0.051	2.997	1.00	0.00	H
ATOM	543	HG21	THR	A	243	5.846	1.798	1.416	1.00	0.00	H
ATOM	544	HG22	THR	A	243	4.932	1.599	2.959	1.00	0.00	H
ATOM	545	HG23	THR	A	243	4.409	0.811	1.347	1.00	0.00	H
ATOM	546	HG1	THR	A	243	5.554	-1.640	3.797	1.00	0.00	H
ATOM	547	N	GLY	A	244	8.513	-0.377	0.577	1.00	0.00	N
ATOM	548	CA	GLY	A	244	9.704	0.086	-0.194	1.00	0.00	C
ATOM	549	C	GLY	A	244	9.667	-0.474	-1.525	1.00	0.00	C
ATOM	550	O	GLY	A	244	9.864	0.240	-2.538	1.00	0.00	O

ATOM	551	H	GLY	A	244	8.716	-0.855	1.443	1.00	0.00	H
ATOM	552	HA2	GLY	A	244	9.716	1.171	-0.299	1.00	0.00	H
ATOM	553	HA3	GLY	A	244	10.591	-0.238	0.353	1.00	0.00	H
ATOM	554	HC	GLY	A	244	9.466	-1.528	-1.651	1.00	0.00	H
ATOM	555	N	GLY	A	246	7.082	-2.240	-3.294	1.00	0.00	N
ATOM	556	CA	GLY	A	246	5.933	-1.708	-3.945	1.00	0.00	C
ATOM	557	C	GLY	A	246	6.044	-0.259	-4.360	1.00	0.00	C
ATOM	558	O	GLY	A	246	5.737	0.098	-5.481	1.00	0.00	O
ATOM	559	H1	GLY	A	246	8.009	-2.180	-3.690	1.00	0.00	H
ATOM	560	H2	GLY	A	246	6.899	-2.676	-2.418	1.00	0.00	H
ATOM	561	HA2	GLY	A	246	5.159	-1.883	-3.221	1.00	0.00	H
ATOM	562	HA3	GLY	A	246	5.814	-2.266	-4.852	1.00	0.00	H
ATOM	563	N	TYR	A	247	6.603	0.555	-3.458	1.00	0.00	N
ATOM	564	CA	TYR	A	247	6.985	1.976	-3.619	1.00	0.00	C
ATOM	565	CB	TYR	A	247	5.731	2.976	-3.738	1.00	0.00	C
ATOM	566	CG	TYR	A	247	4.963	2.891	-2.424	1.00	0.00	C
ATOM	567	CD1	TYR	A	247	3.570	2.517	-2.370	1.00	0.00	C
ATOM	568	CE1	TYR	A	247	2.796	2.521	-1.214	1.00	0.00	C
ATOM	569	CZ	TYR	A	247	3.357	3.123	-0.100	1.00	0.00	C
ATOM	570	OH	TYR	A	247	2.644	3.149	1.145	1.00	0.00	O
ATOM	571	CD2	TYR	A	247	5.513	3.368	-1.206	1.00	0.00	C
ATOM	572	CE2	TYR	A	247	4.651	3.578	-0.052	1.00	0.00	C
ATOM	573	C	TYR	A	247	7.913	2.236	-4.825	1.00	0.00	C
ATOM	574	O	TYR	A	247	7.729	3.276	-5.517	1.00	0.00	O
ATOM	575	H	TYR	A	247	6.733	0.237	-2.519	1.00	0.00	H
ATOM	576	HA	TYR	A	247	7.581	2.289	-2.786	1.00	0.00	H
ATOM	577	HB2	TYR	A	247	6.062	3.995	-4.025	1.00	0.00	H
ATOM	578	HB3	TYR	A	247	5.148	2.542	-4.576	1.00	0.00	H
ATOM	579	HD1	TYR	A	247	3.150	2.035	-3.250	1.00	0.00	H

ATOM	580	HD2	TYR	A	247	6.550	3.669	-1.196	1.00	0.00	H
ATOM	581	HE1	TYR	A	247	1.883	1.943	-1.060	1.00	0.00	H
ATOM	582	HE2	TYR	A	247	5.099	3.935	0.867	1.00	0.00	H
ATOM	583	HH	TYR	A	247	1.759	2.761	1.079	1.00	0.00	H
ATOM	584	N	GLY	A	248	8.867	1.329	-5.011	1.00	0.00	N
ATOM	585	CA	GLY	A	248	9.796	1.143	-6.126	1.00	0.00	C
ATOM	586	C	GLY	A	248	9.190	1.463	-7.547	1.00	0.00	C
ATOM	587	O	GLY	A	248	9.598	2.282	-8.327	1.00	0.00	O
ATOM	588	H	GLY	A	248	8.967	0.660	-4.261	1.00	0.00	H
ATOM	589	HA2	GLY	A	248	10.603	1.854	-5.949	1.00	0.00	H
ATOM	590	HA3	GLY	A	248	10.146	0.100	-6.142	1.00	0.00	H
ATOM	591	HC	GLY	A	248	8.325	0.891	-7.848	1.00	0.00	H
TER	592		GLY	A	248						
HETATM	593	C1	RIS	B	274	-0.376	0.132	2.685	1.00	0.00	C
HETATM	594	C2	RIS	B	274	-0.737	1.293	3.702	1.00	0.00	C
HETATM	595	C3	RIS	B	274	-0.258	1.050	5.132	1.00	0.00	C
HETATM	596	C4	RIS	B	274	2.110	1.920	4.524	1.00	0.00	C
HETATM	597	C5	RIS	B	274	1.050	1.198	5.490	1.00	0.00	C
HETATM	598	N1	RIS	B	274	1.596	0.870	6.743	1.00	0.00	N
HETATM	599	C6	RIS	B	274	-1.125	0.250	6.048	1.00	0.00	C
HETATM	600	C7	RIS	B	274	0.824	0.129	7.573	1.00	0.00	C
HETATM	601	N2	RIS	B	274	-0.528	-0.226	7.209	1.00	0.00	N
HETATM	602	O1	RIS	B	274	-2.359	-0.054	5.913	1.00	0.00	O
HETATM	603	C8	RIS	B	274	1.402	-0.301	8.834	1.00	0.00	C
HETATM	604	C9	RIS	B	274	-1.297	-1.170	8.080	1.00	0.00	C
HETATM	605	C10	RIS	B	274	0.449	-0.982	9.856	1.00	0.00	C
HETATM	606	C11	RIS	B	274	-0.499	-1.921	9.103	1.00	0.00	C
HETATM	607	N3	RIS	B	274	-1.383	0.132	1.483	1.00	0.00	N1+
HETATM	608	C12	RIS	B	274	-2.775	-0.350	1.482	1.00	0.00	C

HETATM	609	C13	RIS	B	274	-0.596	-0.455	0.332	1.00	0.00	C
HETATM	610	C14	RIS	B	274	-3.573	0.142	0.226	1.00	0.00	C
HETATM	611	C15	RIS	B	274	-1.469	-1.194	-0.686	1.00	0.00	C
HETATM	612	C16	RIS	B	274	-2.679	-0.215	-1.022	1.00	0.00	C
HETATM	613	C17	RIS	B	274	-3.548	-0.531	-2.272	1.00	0.00	C
HETATM	614	N4	RIS	B	274	-4.771	0.085	-2.354	1.00	0.00	N
HETATM	615	C18	RIS	B	274	-3.167	-1.184	-3.435	1.00	0.00	C
HETATM	616	O2	RIS	B	274	-5.242	-0.262	-3.658	1.00	0.00	O
HETATM	617	C19	RIS	B	274	-4.247	-0.907	-4.301	1.00	0.00	C
HETATM	618	C20	RIS	B	274	-2.073	-1.849	-3.996	1.00	0.00	C
HETATM	619	C21	RIS	B	274	-4.305	-1.451	-5.533	1.00	0.00	C
HETATM	620	C22	RIS	B	274	-2.079	-2.353	-5.301	1.00	0.00	C
HETATM	621	C23	RIS	B	274	-3.255	-2.135	-6.071	1.00	0.00	C
HETATM	622	F	RIS	B	274	-3.298	-2.876	-7.199	1.00	0.00	F
HETATM	623	H4	RIS	B	274	-5.231	0.652	-1.656	1.00	0.00	H
HETATM	624	H23	RIS	B	274	-3.376	-1.166	-6.556	1.00	0.00	H
HETATM	625	H1	RIS	B	274	-0.628	-0.823	3.186	1.00	0.00	H
HETATM	626	H2	RIS	B	274	0.639	0.304	2.303	1.00	0.00	H
HETATM	627	H3	RIS	B	274	-1.834	1.376	3.884	1.00	0.00	H
HETATM	628	H4	RIS	B	274	-0.318	2.286	3.425	1.00	0.00	H
HETATM	629	H5	RIS	B	274	3.122	1.858	4.978	1.00	0.00	H
HETATM	630	H6	RIS	B	274	1.854	2.992	4.621	1.00	0.00	H
HETATM	631	H7	RIS	B	274	2.143	1.654	3.436	1.00	0.00	H
HETATM	632	H8	RIS	B	274	1.811	0.625	9.279	1.00	0.00	H
HETATM	633	H9	RIS	B	274	2.153	-1.086	8.639	1.00	0.00	H
HETATM	634	H10	RIS	B	274	-2.000	-1.718	7.425	1.00	0.00	H
HETATM	635	H11	RIS	B	274	-1.792	-0.337	8.602	1.00	0.00	H
HETATM	636	H12	RIS	B	274	-0.252	-0.242	10.288	1.00	0.00	H
HETATM	637	H13	RIS	B	274	0.994	-1.497	10.677	1.00	0.00	H

HETATM	638	H14	RIS	B 274	0.039	-2.727	8.567	1.00	0.00	H
HETATM	639	H15	RIS	B 274	-1.174	-2.384	9.856	1.00	0.00	H
HETATM	640	H16	RIS	B 274	-3.110	0.204	2.373	1.00	0.00	H
HETATM	641	H17	RIS	B 274	-2.783	-1.381	1.837	1.00	0.00	H
HETATM	642	H18	RIS	B 274	0.053	-1.225	0.740	1.00	0.00	H
HETATM	643	H19	RIS	B 274	-0.034	0.434	-0.012	1.00	0.00	H
HETATM	644	H20	RIS	B 274	-3.750	1.228	0.173	1.00	0.00	H
HETATM	645	H21	RIS	B 274	-4.513	-0.449	0.094	1.00	0.00	H
HETATM	646	H22	RIS	B 274	-1.786	-2.153	-0.233	1.00	0.00	H
HETATM	647	H23	RIS	B 274	-0.780	-1.359	-1.556	1.00	0.00	H
HETATM	648	H24	RIS	B 274	-2.125	0.675	-1.378	1.00	0.00	H
HETATM	649	H25	RIS	B 274	-1.209	-1.982	-3.362	1.00	0.00	H
HETATM	650	H26	RIS	B 274	-5.121	-1.356	-6.254	1.00	0.00	H
HETATM	651	H27	RIS	B 274	-1.329	-2.941	-5.810	1.00	0.00	H
HETATM	652	H28	RIS	B 274	-1.477	1.141	1.285	1.00	0.00	H
HETATM	653	OH2	TIP	X1826	-4.153	-1.967	4.376	1.00	0.00	O
HETATM	654	H1	TIP	X1826	-3.432	-1.656	4.922	1.00	0.00	H
HETATM	655	H2	TIP	X1826	-4.739	-2.426	4.979	1.00	0.00	H
HETATM	656	OH2	TIP	X2333	0.486	0.446	13.586	1.00	0.00	O
HETATM	657	H1	TIP	X2333	0.880	1.032	14.236	1.00	0.00	H
HETATM	658	H2	TIP	X2333	-0.105	1.017	13.096	1.00	0.00	H
HETATM	659	OH2	TIP	X5640	-5.895	-0.439	2.960	1.00	0.00	O
HETATM	660	H1	TIP	X5640	-5.496	0.375	3.258	1.00	0.00	H
HETATM	661	H2	TIP	X5640	-5.524	-1.103	3.551	1.00	0.00	H
HETATM	662	OH2	TIP	X7470	2.243	-1.190	3.623	1.00	0.00	O
HETATM	663	H1	TIP	X7470	2.200	-2.121	3.827	1.00	0.00	H
HETATM	664	H2	TIP	X7470	3.175	-1.008	3.523	1.00	0.00	H
HETATM	665	OH2	TIP	X9631	6.321	1.410	6.591	1.00	0.00	O
HETATM	666	H1	TIP	X9631	7.269	1.304	6.622	1.00	0.00	H

HETATM	667	H2	TIP	X9631	6.061	1.568	7.494	1.00	0.00	H
HETATM	668	OH2	TIP	X1377	2.868	2.833	9.630	1.00	0.00	O
HETATM	669	H1	TIP	X1377	2.389	3.451	9.075	1.00	0.00	H
HETATM	670	H2	TIP	X1377	2.509	2.968	10.510	1.00	0.00	H
HETATM	671	OH2	TIP	X1460	2.723	-1.343	12.751	1.00	0.00	O
HETATM	672	H1	TIP	X1460	1.913	-0.885	12.935	1.00	0.00	H
HETATM	673	H2	TIP	X1460	2.484	-2.268	12.660	1.00	0.00	H
HETATM	674	OH2	TIP	X1795	5.462	-1.313	9.368	1.00	0.00	O
HETATM	675	H1	TIP	X1795	5.092	-1.156	8.492	1.00	0.00	H
HETATM	676	H2	TIP	X1795	5.176	-0.562	9.881	1.00	0.00	H
HETATM	677	OH2	TIP	X3662	4.231	-0.613	6.707	1.00	0.00	O
HETATM	678	H1	TIP	X3662	5.022	-0.107	6.524	1.00	0.00	H
HETATM	679	H2	TIP	X3662	3.553	0.052	6.825	1.00	0.00	H
HETATM	680	OH2	TIP	X6700	-0.997	2.759	7.992	1.00	0.00	O
HETATM	681	H1	TIP	X6700	-1.480	2.884	8.806	1.00	0.00	H
HETATM	682	H2	TIP	X6700	-0.615	1.891	8.050	1.00	0.00	H
HETATM	683	OH2	TIP	X8172	-4.432	1.626	4.060	1.00	0.00	O
HETATM	684	H1	TIP	X8172	-3.817	2.324	3.845	1.00	0.00	H
HETATM	685	H2	TIP	X8172	-4.068	1.213	4.843	1.00	0.00	H
HETATM	686	OH2	TIP	X 383	-0.329	2.850	12.818	1.00	0.00	O
HETATM	687	H1	TIP	X 383	-0.652	3.247	13.624	1.00	0.00	H
HETATM	688	H2	TIP	X 383	-0.959	3.101	12.147	1.00	0.00	H
HETATM	689	OH2	TIP	X 601	-0.791	-3.964	12.817	1.00	0.00	O
HETATM	690	H1	TIP	X 601	-1.358	-3.472	13.425	1.00	0.00	H
HETATM	691	H2	TIP	X 601	-1.384	-4.580	12.383	1.00	0.00	H
HETATM	692	OH2	TIP	X2639	1.132	4.783	7.801	1.00	0.00	O
HETATM	693	H1	TIP	X2639	0.917	5.259	6.989	1.00	0.00	H
HETATM	694	H2	TIP	X2639	0.382	4.216	7.955	1.00	0.00	H
HETATM	695	OH2	TIP	X9870	-3.236	4.124	3.312	1.00	0.00	O

```
HETATM  696  H1  TIP X9870      -2.803   4.970   3.352  1.00  0.00            H
HETATM  697  H2  TIP X9870      -2.921   3.728   2.495  1.00  0.00            H
HETATM  698  OH2 TIP X 221       4.436   3.651   6.077  1.00  0.00            O
HETATM  699  H1  TIP X 221       4.817   2.804   6.318  1.00  0.00            H
HETATM  700  H2  TIP X 221       4.563   3.704   5.127  1.00  0.00            H
HETATM  701  OH2 TIP X1639       5.288   1.557   9.143  1.00  0.00            O
HETATM  702  H1  TIP X1639       5.944   2.174   9.463  1.00  0.00            H
HETATM  703  H2  TIP X1639       4.455   1.986   9.332  1.00  0.00            H
HETATM  704  OH2 TIP X1693       2.140  -3.890  12.988  1.00  0.00            O
HETATM  705  H1  TIP X1693       1.183  -3.952  13.110  1.00  0.00            H
HETATM  706  H2  TIP X1693       2.364  -4.659  12.477  1.00  0.00            H
HETATM  707  OH2 TIP X5481      -3.875  -2.715  11.678  1.00  0.00            O
HETATM  708  H1  TIP X5481      -3.877  -1.789  11.427  1.00  0.00            H
HETATM  709  H2  TIP X5481      -4.600  -2.806  12.291  1.00  0.00            H
HETATM  710  OH2 TIP X7777      -2.949  -5.296  11.675  1.00  0.00            O
HETATM  711  H1  TIP X7777      -3.452  -5.802  12.316  1.00  0.00            H
HETATM  712  H2  TIP X7777      -3.476  -4.515  11.514  1.00  0.00            H
HETATM  713  OH2 TIP X3280       4.942   3.964   3.370  1.00  0.00            O
HETATM  714  H1  TIP X3280       4.726   4.877   3.213  1.00  0.00            H
HETATM  715  H2  TIP X3280       4.378   3.476   2.764  1.00  0.00            H
END
```

Risperidone (Cluster structure 2)

```
ATOM      1  N   GLY A  75      -6.241   8.981   5.171  1.00  0.00            N
ATOM      2  CA  GLY A  75      -5.631   9.515   3.923  1.00  0.00            C
ATOM      3  C   GLY A  75      -4.348   8.757   3.723  1.00  0.00            C
ATOM      4  O   GLY A  75      -4.029   8.477   2.588  1.00  0.00            O
ATOM      5  H1  GLY A  75      -7.130   8.581   4.864  1.00  0.00            H
ATOM      6  H2  GLY A  75      -6.272   9.786   5.799  1.00  0.00            H
```

ATOM	7	HA2	GLY	A	75	-6.286	9.400	3.059	1.00	0.00	H
ATOM	8	HA3	GLY	A	75	-5.430	10.558	4.059	1.00	0.00	H
ATOM	9	N	PHE	A	76	-3.522	8.546	4.773	1.00	0.00	N
ATOM	10	CA	PHE	A	76	-2.538	7.451	4.988	1.00	0.00	C
ATOM	11	CB	PHE	A	76	-1.098	7.954	4.820	1.00	0.00	C
ATOM	12	CG	PHE	A	76	-0.581	8.699	6.018	1.00	0.00	C
ATOM	13	CD1	PHE	A	76	0.190	8.103	7.046	1.00	0.00	C
ATOM	14	CE1	PHE	A	76	0.532	8.832	8.264	1.00	0.00	C
ATOM	15	CZ	PHE	A	76	0.282	10.213	8.245	1.00	0.00	C
ATOM	16	CD2	PHE	A	76	-0.780	10.080	6.095	1.00	0.00	C
ATOM	17	CE2	PHE	A	76	-0.379	10.799	7.218	1.00	0.00	C
ATOM	18	C	PHE	A	76	-2.742	6.224	4.016	1.00	0.00	C
ATOM	19	O	PHE	A	76	-1.940	5.916	3.156	1.00	0.00	O
ATOM	20	H	PHE	A	76	-3.729	9.042	5.628	1.00	0.00	H
ATOM	21	HA	PHE	A	76	-2.532	7.056	5.979	1.00	0.00	H
ATOM	22	HB2	PHE	A	76	-0.951	8.553	3.905	1.00	0.00	H
ATOM	23	HB3	PHE	A	76	-0.433	7.076	4.716	1.00	0.00	H
ATOM	24	HD1	PHE	A	76	0.547	7.090	6.962	1.00	0.00	H
ATOM	25	HD2	PHE	A	76	-1.300	10.640	5.325	1.00	0.00	H
ATOM	26	HE1	PHE	A	76	1.155	8.413	9.041	1.00	0.00	H
ATOM	27	HE2	PHE	A	76	-0.442	11.883	7.167	1.00	0.00	H
ATOM	28	HZ	PHE	A	76	0.667	10.819	9.049	1.00	0.00	H
ATOM	29	N	VAL	A	77	-3.848	5.491	4.162	1.00	0.00	N
ATOM	30	CA	VAL	A	77	-4.281	4.367	3.340	1.00	0.00	C
ATOM	31	CB	VAL	A	77	-5.462	3.649	3.911	1.00	0.00	C
ATOM	32	CG1	VAL	A	77	-5.867	2.425	3.028	1.00	0.00	C
ATOM	33	CG2	VAL	A	77	-5.208	3.188	5.351	1.00	0.00	C
ATOM	34	C	VAL	A	77	-4.531	4.796	1.930	1.00	0.00	C
ATOM	35	O	VAL	A	77	-4.045	4.222	0.943	1.00	0.00	O

ATOM	36	H	VAL	A	77	-4.385	5.893	4.895	1.00	0.00	H
ATOM	37	HA	VAL	A	77	-3.441	3.686	3.385	1.00	0.00	H
ATOM	38	HB	VAL	A	77	-6.305	4.367	3.928	1.00	0.00	H
ATOM	39	HG11	VAL	A	77	-6.114	2.644	1.962	1.00	0.00	H
ATOM	40	HG12	VAL	A	77	-6.715	1.865	3.462	1.00	0.00	H
ATOM	41	HG13	VAL	A	77	-5.076	1.653	2.973	1.00	0.00	H
ATOM	42	HG21	VAL	A	77	-5.230	4.015	6.099	1.00	0.00	H
ATOM	43	HG22	VAL	A	77	-4.245	2.636	5.395	1.00	0.00	H
ATOM	44	HG23	VAL	A	77	-6.002	2.504	5.735	1.00	0.00	H
ATOM	45	N	GLY	A	78	-5.337	5.898	1.665	1.00	0.00	N
ATOM	46	CA	GLY	A	78	-5.687	6.524	0.323	1.00	0.00	C
ATOM	47	C	GLY	A	78	-4.528	6.946	-0.495	1.00	0.00	C
ATOM	48	O	GLY	A	78	-4.406	6.709	-1.731	1.00	0.00	O
ATOM	49	H	GLY	A	78	-5.746	6.344	2.474	1.00	0.00	H
ATOM	50	HA2	GLY	A	78	-6.173	5.764	-0.288	1.00	0.00	H
ATOM	51	HA3	GLY	A	78	-6.346	7.387	0.540	1.00	0.00	H
ATOM	52	N	GLY	A	79	-3.445	7.583	0.057	1.00	0.00	N
ATOM	53	CA	GLY	A	79	-2.224	7.832	-0.641	1.00	0.00	C
ATOM	54	C	GLY	A	79	-1.524	6.593	-1.199	1.00	0.00	C
ATOM	55	O	GLY	A	79	-1.222	6.508	-2.388	1.00	0.00	O
ATOM	56	H	GLY	A	79	-3.516	7.895	1.015	1.00	0.00	H
ATOM	57	HA2	GLY	A	79	-2.511	8.438	-1.501	1.00	0.00	H
ATOM	58	HA3	GLY	A	79	-1.511	8.354	0.035	1.00	0.00	H
ATOM	59	N	ASP	A	80	-1.353	5.547	-0.349	1.00	0.00	N
ATOM	60	CA	ASP	A	80	-0.928	4.196	-0.802	1.00	0.00	C
ATOM	61	CB	ASP	A	80	0.572	3.997	-0.924	1.00	0.00	C
ATOM	62	CG	ASP	A	80	1.382	4.006	0.376	1.00	0.00	C
ATOM	63	OD1	ASP	A	80	0.733	3.649	1.386	1.00	0.00	O
ATOM	64	OD2	ASP	A	80	2.598	4.297	0.415	1.00	0.00	O1-

ATOM	65	C	ASP	A	80	-1.787	3.606	-1.871	1.00	0.00	C
ATOM	66	O	ASP	A	80	-1.229	2.939	-2.763	1.00	0.00	O
ATOM	67	H	ASP	A	80	-1.805	5.631	0.541	1.00	0.00	H
ATOM	68	HA	ASP	A	80	-1.033	3.469	-0.009	1.00	0.00	H
ATOM	69	HB2	ASP	A	80	0.857	4.832	-1.601	1.00	0.00	H
ATOM	70	HB3	ASP	A	80	0.724	3.048	-1.481	1.00	0.00	H
ATOM	71	N	VAL	A	81	-3.078	3.737	-1.779	1.00	0.00	N
ATOM	72	CA	VAL	A	81	-4.037	3.199	-2.726	1.00	0.00	C
ATOM	73	CB	VAL	A	81	-5.484	2.958	-2.174	1.00	0.00	C
ATOM	74	CG1	VAL	A	81	-6.584	2.519	-3.223	1.00	0.00	C
ATOM	75	CG2	VAL	A	81	-5.486	1.874	-1.063	1.00	0.00	C
ATOM	76	C	VAL	A	81	-4.075	3.937	-4.058	1.00	0.00	C
ATOM	77	O	VAL	A	81	-3.943	3.330	-5.157	1.00	0.00	O
ATOM	78	H	VAL	A	81	-3.432	4.219	-0.982	1.00	0.00	H
ATOM	79	HA	VAL	A	81	-3.785	2.189	-3.053	1.00	0.00	H
ATOM	80	HB	VAL	A	81	-5.808	3.965	-1.845	1.00	0.00	H
ATOM	81	HG11	VAL	A	81	-7.523	2.562	-2.632	1.00	0.00	H
ATOM	82	HG12	VAL	A	81	-6.460	1.483	-3.607	1.00	0.00	H
ATOM	83	HG13	VAL	A	81	-6.769	3.214	-4.071	1.00	0.00	H
ATOM	84	HG21	VAL	A	81	-4.937	0.988	-1.412	1.00	0.00	H
ATOM	85	HG22	VAL	A	81	-6.509	1.644	-0.684	1.00	0.00	H
ATOM	86	HG23	VAL	A	81	-4.846	2.234	-0.220	1.00	0.00	H
ATOM	87	N	GLY	A	82	-4.197	5.262	-3.995	1.00	0.00	N
ATOM	88	CA	GLY	A	82	-4.338	6.075	-5.245	1.00	0.00	C
ATOM	89	C	GLY	A	82	-3.050	5.956	-6.133	1.00	0.00	C
ATOM	90	O	GLY	A	82	-3.102	5.642	-7.330	1.00	0.00	O
ATOM	91	H	GLY	A	82	-4.194	5.723	-3.096	1.00	0.00	H
ATOM	92	HA2	GLY	A	82	-5.194	5.703	-5.808	1.00	0.00	H
ATOM	93	HA3	GLY	A	82	-4.477	7.113	-4.976	1.00	0.00	H

ATOM	94	N	GLY	A	83	-1.821	6.046	-5.557	1.00	0.00	N
ATOM	95	CA	GLY	A	83	-0.586	5.812	-6.254	1.00	0.00	C
ATOM	96	C	GLY	A	83	-0.539	4.433	-6.872	1.00	0.00	C
ATOM	97	O	GLY	A	83	-0.082	4.240	-7.997	1.00	0.00	O
ATOM	98	H	GLY	A	83	-1.773	6.293	-4.579	1.00	0.00	H
ATOM	99	HA2	GLY	A	83	-0.515	6.547	-7.056	1.00	0.00	H
ATOM	100	HA3	GLY	A	83	0.254	5.917	-5.554	1.00	0.00	H
ATOM	101	N	CYS	A	84	-0.898	3.300	-6.145	1.00	0.00	N
ATOM	102	CA	CYS	A	84	-1.001	1.998	-6.626	1.00	0.00	C
ATOM	103	CB	CYS	A	84	-1.161	0.914	-5.452	1.00	0.00	C
ATOM	104	SG	CYS	A	84	-0.957	-0.811	-6.057	1.00	0.00	S
ATOM	105	C	CYS	A	84	-2.017	1.856	-7.723	1.00	0.00	C
ATOM	106	O	CYS	A	84	-1.673	1.494	-8.879	1.00	0.00	O
ATOM	107	H	CYS	A	84	-1.343	3.526	-5.289	1.00	0.00	H
ATOM	108	HA	CYS	A	84	-0.110	1.797	-7.189	1.00	0.00	H
ATOM	109	HB2	CYS	A	84	-2.184	1.044	-5.037	1.00	0.00	H
ATOM	110	HB3	CYS	A	84	-0.463	1.038	-4.602	1.00	0.00	H
ATOM	111	HG	CYS	A	84	0.238	-1.095	-5.582	1.00	0.00	H
ATOM	112	N	GLY	A	85	-3.253	2.296	-7.491	1.00	0.00	N
ATOM	113	CA	GLY	A	85	-4.366	2.226	-8.466	1.00	0.00	C
ATOM	114	C	GLY	A	85	-4.059	2.882	-9.783	1.00	0.00	C
ATOM	115	O	GLY	A	85	-4.333	2.266	-10.787	1.00	0.00	O
ATOM	116	H	GLY	A	85	-3.444	2.706	-6.588	1.00	0.00	H
ATOM	117	HA2	GLY	A	85	-4.544	1.174	-8.689	1.00	0.00	H
ATOM	118	HA3	GLY	A	85	-5.249	2.705	-8.011	1.00	0.00	H
ATOM	119	HC	GLY	A	85	-3.612	3.864	-9.831	1.00	0.00	H
ATOM	120	N	GLY	A	147	-5.035	7.467	14.226	1.00	0.00	N
ATOM	121	CA	GLY	A	147	-4.014	6.458	14.268	1.00	0.00	C
ATOM	122	C	GLY	A	147	-4.086	5.589	12.953	1.00	0.00	C

ATOM	123	O	GLY	A	147	-5.186	5.251	12.462	1.00	0.00	O
ATOM	124	H1	GLY	A	147	-4.534	8.346	14.215	1.00	0.00	H
ATOM	125	H2	GLY	A	147	-5.649	7.250	15.000	1.00	0.00	H
ATOM	126	HA2	GLY	A	147	-3.049	6.956	14.362	1.00	0.00	H
ATOM	127	HA3	GLY	A	147	-4.166	5.793	15.107	1.00	0.00	H
ATOM	128	N	CYS	A	148	-2.858	5.256	12.422	1.00	0.00	N
ATOM	129	CA	CYS	A	148	-2.689	4.380	11.215	1.00	0.00	C
ATOM	130	CB	CYS	A	148	-1.181	4.342	10.880	1.00	0.00	C
ATOM	131	SG	CYS	A	148	-0.398	5.962	10.494	1.00	0.00	S
ATOM	132	C	CYS	A	148	-3.102	2.943	11.520	1.00	0.00	C
ATOM	133	O	CYS	A	148	-2.403	2.188	12.241	1.00	0.00	O
ATOM	134	H	CYS	A	148	-2.005	5.497	12.881	1.00	0.00	H
ATOM	135	HA	CYS	A	148	-3.372	4.742	10.469	1.00	0.00	H
ATOM	136	HB2	CYS	A	148	-0.988	3.678	10.007	1.00	0.00	H
ATOM	137	HB3	CYS	A	148	-0.675	3.840	11.731	1.00	0.00	H
ATOM	138	HG	CYS	A	148	-1.035	6.531	9.467	1.00	0.00	H
ATOM	139	N	ILE	A	149	-4.065	2.505	10.788	1.00	0.00	N
ATOM	140	CA	ILE	A	149	-4.651	1.129	10.794	1.00	0.00	C
ATOM	141	CB	ILE	A	149	-5.466	0.902	9.444	1.00	0.00	C
ATOM	142	CG2	ILE	A	149	-4.532	0.670	8.270	1.00	0.00	C
ATOM	143	CG1	ILE	A	149	-6.584	-0.152	9.413	1.00	0.00	C
ATOM	144	CD1	ILE	A	149	-6.132	-1.632	9.250	1.00	0.00	C
ATOM	145	C	ILE	A	149	-5.472	0.844	12.059	1.00	0.00	C
ATOM	146	O	ILE	A	149	-5.371	-0.248	12.588	1.00	0.00	O
ATOM	147	H	ILE	A	149	-4.479	3.063	10.071	1.00	0.00	H
ATOM	148	HA	ILE	A	149	-3.780	0.514	10.886	1.00	0.00	H
ATOM	149	HB	ILE	A	149	-5.988	1.865	9.235	1.00	0.00	H
ATOM	150	HG12	ILE	A	149	-7.183	0.051	8.499	1.00	0.00	H
ATOM	151	HG13	ILE	A	149	-7.298	0.079	10.239	1.00	0.00	H

ATOM	152	HG21	ILE	A	149	-3.867	-0.218	8.337	1.00	0.00	H
ATOM	153	HG22	ILE	A	149	-3.808	1.513	8.206	1.00	0.00	H
ATOM	154	HG23	ILE	A	149	-5.140	0.470	7.367	1.00	0.00	H
ATOM	155	HD11	ILE	A	149	-6.900	-2.425	9.391	1.00	0.00	H
ATOM	156	HD12	ILE	A	149	-5.471	-1.916	10.077	1.00	0.00	H
ATOM	157	HD13	ILE	A	149	-5.612	-1.838	8.282	1.00	0.00	H
ATOM	158	N	GLY	A	150	-6.101	1.834	12.666	1.00	0.00	N
ATOM	159	CA	GLY	A	150	-6.687	1.794	14.028	1.00	0.00	C
ATOM	160	C	GLY	A	150	-8.023	0.981	14.175	1.00	0.00	C
ATOM	161	O	GLY	A	150	-8.160	0.180	15.102	1.00	0.00	O
ATOM	162	H	GLY	A	150	-6.192	2.700	12.154	1.00	0.00	H
ATOM	163	HA2	GLY	A	150	-5.949	1.273	14.638	1.00	0.00	H
ATOM	164	HA3	GLY	A	150	-6.881	2.829	14.362	1.00	0.00	H
ATOM	165	HC	GLY	A	150	-8.822	1.124	13.463	1.00	0.00	H
ATOM	166	N	GLY	A	162	-12.004	-5.805	-3.154	1.00	0.00	N
ATOM	167	CA	GLY	A	162	-11.819	-4.789	-4.248	1.00	0.00	C
ATOM	168	C	GLY	A	162	-10.442	-4.748	-4.887	1.00	0.00	C
ATOM	169	O	GLY	A	162	-10.240	-4.790	-6.135	1.00	0.00	O
ATOM	170	H1	GLY	A	162	-12.639	-6.445	-3.532	1.00	0.00	H
ATOM	171	H2	GLY	A	162	-12.125	-5.264	-2.348	1.00	0.00	H
ATOM	172	HA2	GLY	A	162	-12.487	-5.081	-5.058	1.00	0.00	H
ATOM	173	HA3	GLY	A	162	-12.056	-3.795	-3.822	1.00	0.00	H
ATOM	174	N	SER	A	163	-9.288	-4.859	-4.167	1.00	0.00	N
ATOM	175	CA	SER	A	163	-7.942	-4.983	-4.676	1.00	0.00	C
ATOM	176	CB	SER	A	163	-6.798	-4.513	-3.721	1.00	0.00	C
ATOM	177	OG	SER	A	163	-6.886	-5.295	-2.475	1.00	0.00	O
ATOM	178	C	SER	A	163	-7.606	-6.279	-5.459	1.00	0.00	C
ATOM	179	O	SER	A	163	-6.975	-6.279	-6.526	1.00	0.00	O
ATOM	180	H	SER	A	163	-9.363	-4.954	-3.193	1.00	0.00	H

ATOM	181	HA	SER	A	163	-7.856	-4.264	-5.481	1.00	0.00		H
ATOM	182	HB2	SER	A	163	-6.909	-3.419	-3.505	1.00	0.00		H
ATOM	183	HB3	SER	A	163	-5.798	-4.691	-4.150	1.00	0.00		H
ATOM	184	HG	SER	A	163	-7.528	-4.772	-1.971	1.00	0.00		H
ATOM	185	N	GLY	A	164	-8.028	-7.465	-4.943	1.00	0.00		N
ATOM	186	CA	GLY	A	164	-7.964	-8.722	-5.614	1.00	0.00		C
ATOM	187	C	GLY	A	164	-8.828	-8.766	-6.912	1.00	0.00		C
ATOM	188	O	GLY	A	164	-8.316	-9.058	-7.975	1.00	0.00		O
ATOM	189	H	GLY	A	164	-8.417	-7.455	-4.011	1.00	0.00		H
ATOM	190	HA2	GLY	A	164	-6.918	-8.861	-5.886	1.00	0.00		H
ATOM	191	HA3	GLY	A	164	-8.319	-9.526	-4.953	1.00	0.00		H
ATOM	192	HC	GLY	A	164	-9.882	-8.537	-6.866	1.00	0.00		H
ATOM	193	N	GLY	A	213	3.887	-6.545	-10.028	1.00	0.00		N
ATOM	194	CA	GLY	A	213	3.415	-5.303	-9.333	1.00	0.00		C
ATOM	195	C	GLY	A	213	2.220	-5.614	-8.464	1.00	0.00		C
ATOM	196	O	GLY	A	213	2.037	-5.185	-7.328	1.00	0.00		O
ATOM	197	H1	GLY	A	213	4.805	-6.697	-9.656	1.00	0.00		H
ATOM	198	H2	GLY	A	213	3.738	-6.351	-10.999	1.00	0.00		H
ATOM	199	HA2	GLY	A	213	4.205	-4.908	-8.694	1.00	0.00		H
ATOM	200	HA3	GLY	A	213	3.145	-4.574	-10.091	1.00	0.00		H
ATOM	201	N	ILE	A	214	1.207	-6.412	-8.954	1.00	0.00		N
ATOM	202	CA	ILE	A	214	0.100	-6.986	-8.197	1.00	0.00		C
ATOM	203	CB	ILE	A	214	-1.151	-6.225	-8.104	1.00	0.00		C
ATOM	204	CG2	ILE	A	214	-1.048	-4.937	-7.362	1.00	0.00		C
ATOM	205	CG1	ILE	A	214	-2.291	-7.052	-7.452	1.00	0.00		C
ATOM	206	CD1	ILE	A	214	-2.702	-8.303	-8.207	1.00	0.00		C
ATOM	207	C	ILE	A	214	0.566	-7.668	-6.945	1.00	0.00		C
ATOM	208	O	ILE	A	214	0.327	-7.337	-5.759	1.00	0.00		O
ATOM	209	H	ILE	A	214	1.264	-6.832	-9.852	1.00	0.00		H

ATOM	210	HA	ILE	A	214	-0.225	-7.723	-8.920	1.00	0.00	H
ATOM	211	HB	ILE	A	214	-1.513	-5.997	-9.132	1.00	0.00	H
ATOM	212	HG12	ILE	A	214	-3.160	-6.369	-7.500	1.00	0.00	H
ATOM	213	HG13	ILE	A	214	-2.015	-7.304	-6.394	1.00	0.00	H
ATOM	214	HG21	ILE	A	214	-0.695	-5.154	-6.335	1.00	0.00	H
ATOM	215	HG22	ILE	A	214	-2.017	-4.407	-7.368	1.00	0.00	H
ATOM	216	HG23	ILE	A	214	-0.302	-4.311	-7.906	1.00	0.00	H
ATOM	217	HD11	ILE	A	214	-3.710	-8.565	-7.808	1.00	0.00	H
ATOM	218	HD12	ILE	A	214	-1.938	-9.109	-8.135	1.00	0.00	H
ATOM	219	HD13	ILE	A	214	-2.831	-8.003	-9.266	1.00	0.00	H
ATOM	220	N	GLY	A	215	1.465	-8.628	-7.228	1.00	0.00	N
ATOM	221	CA	GLY	A	215	2.250	-9.267	-6.180	1.00	0.00	C
ATOM	222	C	GLY	A	215	2.874	-8.366	-5.084	1.00	0.00	C
ATOM	223	O	GLY	A	215	2.797	-8.524	-3.860	1.00	0.00	O
ATOM	224	H	GLY	A	215	1.599	-8.912	-8.188	1.00	0.00	H
ATOM	225	HA2	GLY	A	215	1.517	-9.875	-5.650	1.00	0.00	H
ATOM	226	HA3	GLY	A	215	3.081	-9.847	-6.657	1.00	0.00	H
ATOM	227	N	GLY	A	216	3.651	-7.315	-5.545	1.00	0.00	N
ATOM	228	CA	GLY	A	216	4.240	-6.183	-4.765	1.00	0.00	C
ATOM	229	C	GLY	A	216	3.144	-5.428	-3.869	1.00	0.00	C
ATOM	230	O	GLY	A	216	3.380	-5.181	-2.664	1.00	0.00	O
ATOM	231	H	GLY	A	216	3.845	-7.313	-6.536	1.00	0.00	H
ATOM	232	HA2	GLY	A	216	4.999	-6.609	-4.109	1.00	0.00	H
ATOM	233	HA3	GLY	A	216	4.663	-5.456	-5.448	1.00	0.00	H
ATOM	234	N	TRP	A	217	1.941	-5.071	-4.392	1.00	0.00	N
ATOM	235	CA	TRP	A	217	0.874	-4.378	-3.717	1.00	0.00	C
ATOM	236	CB	TRP	A	217	1.290	-2.919	-3.295	1.00	0.00	C
ATOM	237	CG	TRP	A	217	1.791	-2.001	-4.398	1.00	0.00	C
ATOM	238	CD1	TRP	A	217	1.949	-2.055	-5.722	1.00	0.00	C

ATOM	239	NE1	TRP	A	217	2.510	-0.878	-6.122	1.00	0.00	N
ATOM	240	CE2	TRP	A	217	2.606	0.058	-5.165	1.00	0.00	C
ATOM	241	CD2	TRP	A	217	2.143	-0.615	-4.007	1.00	0.00	C
ATOM	242	CE3	TRP	A	217	2.176	0.133	-2.813	1.00	0.00	C
ATOM	243	CZ3	TRP	A	217	2.612	1.421	-2.809	1.00	0.00	C
ATOM	244	CZ2	TRP	A	217	2.919	1.471	-5.220	1.00	0.00	C
ATOM	245	CH2	TRP	A	217	2.871	2.135	-4.008	1.00	0.00	C
ATOM	246	C	TRP	A	217	0.205	-5.136	-2.506	1.00	0.00	C
ATOM	247	O	TRP	A	217	-0.286	-4.582	-1.500	1.00	0.00	O
ATOM	248	H	TRP	A	217	1.709	-5.210	-5.351	1.00	0.00	H
ATOM	249	HA	TRP	A	217	0.123	-4.200	-4.469	1.00	0.00	H
ATOM	250	HB2	TRP	A	217	0.399	-2.493	-2.804	1.00	0.00	H
ATOM	251	HB3	TRP	A	217	2.124	-2.983	-2.560	1.00	0.00	H
ATOM	252	HD1	TRP	A	217	1.891	-2.986	-6.283	1.00	0.00	H
ATOM	253	HE1	TRP	A	217	2.284	-0.549	-7.010	1.00	0.00	H
ATOM	254	HE3	TRP	A	217	1.880	-0.332	-1.889	1.00	0.00	H
ATOM	255	HZ2	TRP	A	217	3.007	1.898	-6.219	1.00	0.00	H
ATOM	256	HZ3	TRP	A	217	2.495	1.913	-1.854	1.00	0.00	H
ATOM	257	HH2	TRP	A	217	3.066	3.199	-3.941	1.00	0.00	H
ATOM	258	N	LEU	A	218	0.030	-6.505	-2.755	1.00	0.00	N
ATOM	259	CA	LEU	A	218	-0.604	-7.466	-1.942	1.00	0.00	C
ATOM	260	CB	LEU	A	218	-2.172	-7.553	-2.192	1.00	0.00	C
ATOM	261	CG	LEU	A	218	-2.545	-7.926	-3.640	1.00	0.00	C
ATOM	262	CD1	LEU	A	218	-2.054	-9.304	-4.077	1.00	0.00	C
ATOM	263	CD2	LEU	A	218	-4.090	-7.721	-3.936	1.00	0.00	C
ATOM	264	C	LEU	A	218	-0.365	-7.396	-0.451	1.00	0.00	C
ATOM	265	O	LEU	A	218	-1.323	-7.158	0.297	1.00	0.00	O
ATOM	266	H	LEU	A	218	0.455	-6.830	-3.598	1.00	0.00	H
ATOM	267	HA	LEU	A	218	-0.131	-8.352	-2.339	1.00	0.00	H

ATOM	268	HB2	LEU	A	218	-2.672	-6.586	-1.945	1.00	0.00	H
ATOM	269	HB3	LEU	A	218	-2.537	-8.285	-1.429	1.00	0.00	H
ATOM	270	HG	LEU	A	218	-2.022	-7.156	-4.243	1.00	0.00	H
ATOM	271	HD11	LEU	A	218	-2.283	-9.528	-5.140	1.00	0.00	H
ATOM	272	HD12	LEU	A	218	-2.499	-10.149	-3.509	1.00	0.00	H
ATOM	273	HD13	LEU	A	218	-0.964	-9.499	-3.953	1.00	0.00	H
ATOM	274	HD21	LEU	A	218	-4.381	-7.931	-4.975	1.00	0.00	H
ATOM	275	HD22	LEU	A	218	-4.370	-6.687	-3.611	1.00	0.00	H
ATOM	276	HD23	LEU	A	218	-4.616	-8.424	-3.250	1.00	0.00	H
ATOM	277	N	GLY	A	219	0.802	-7.771	0.097	1.00	0.00	N
ATOM	278	CA	GLY	A	219	1.396	-7.016	1.154	1.00	0.00	C
ATOM	279	C	GLY	A	219	0.573	-6.534	2.371	1.00	0.00	C
ATOM	280	O	GLY	A	219	0.132	-7.343	3.203	1.00	0.00	O
ATOM	281	H	GLY	A	219	1.267	-8.600	-0.244	1.00	0.00	H
ATOM	282	HA2	GLY	A	219	2.048	-7.771	1.593	1.00	0.00	H
ATOM	283	HA3	GLY	A	219	1.894	-6.093	0.707	1.00	0.00	H
ATOM	284	N	PHE	A	220	0.261	-5.225	2.483	1.00	0.00	N
ATOM	285	CA	PHE	A	220	-0.594	-4.693	3.568	1.00	0.00	C
ATOM	286	CB	PHE	A	220	-0.636	-3.150	3.548	1.00	0.00	C
ATOM	287	CG	PHE	A	220	0.426	-2.596	4.397	1.00	0.00	C
ATOM	288	CD1	PHE	A	220	1.653	-2.258	3.905	1.00	0.00	C
ATOM	289	CE1	PHE	A	220	2.702	-1.753	4.740	1.00	0.00	C
ATOM	290	CZ	PHE	A	220	2.478	-1.604	6.101	1.00	0.00	C
ATOM	291	CD2	PHE	A	220	0.192	-2.465	5.736	1.00	0.00	C
ATOM	292	CE2	PHE	A	220	1.205	-2.018	6.574	1.00	0.00	C
ATOM	293	C	PHE	A	220	-2.041	-5.237	3.573	1.00	0.00	C
ATOM	294	O	PHE	A	220	-2.593	-5.523	4.596	1.00	0.00	O
ATOM	295	H	PHE	A	220	0.542	-4.559	1.787	1.00	0.00	H
ATOM	296	HA	PHE	A	220	-0.122	-5.075	4.470	1.00	0.00	H

ATOM	297	HB2	PHE	A	220	-1.604	-2.730	3.901	1.00	0.00	H
ATOM	298	HB3	PHE	A	220	-0.526	-2.743	2.521	1.00	0.00	H
ATOM	299	HD1	PHE	A	220	1.858	-2.362	2.856	1.00	0.00	H
ATOM	300	HD2	PHE	A	220	-0.736	-2.932	6.055	1.00	0.00	H
ATOM	301	HE1	PHE	A	220	3.662	-1.476	4.336	1.00	0.00	H
ATOM	302	HE2	PHE	A	220	1.138	-2.019	7.655	1.00	0.00	H
ATOM	303	HZ	PHE	A	220	3.171	-1.161	6.802	1.00	0.00	H
ATOM	304	N	PHE	A	221	-2.713	-5.428	2.431	1.00	0.00	N
ATOM	305	CA	PHE	A	221	-4.080	-5.993	2.333	1.00	0.00	C
ATOM	306	CB	PHE	A	221	-5.216	-5.134	2.847	1.00	0.00	C
ATOM	307	CG	PHE	A	221	-5.340	-3.811	2.174	1.00	0.00	C
ATOM	308	CD1	PHE	A	221	-5.231	-2.679	3.011	1.00	0.00	C
ATOM	309	CE1	PHE	A	221	-5.522	-1.367	2.470	1.00	0.00	C
ATOM	310	CZ	PHE	A	221	-5.853	-1.247	1.159	1.00	0.00	C
ATOM	311	CD2	PHE	A	221	-5.594	-3.614	0.784	1.00	0.00	C
ATOM	312	CE2	PHE	A	221	-5.693	-2.362	0.283	1.00	0.00	C
ATOM	313	C	PHE	A	221	-4.206	-7.424	2.792	1.00	0.00	C
ATOM	314	O	PHE	A	221	-5.132	-7.736	3.601	1.00	0.00	O
ATOM	315	H	PHE	A	221	-2.299	-5.138	1.574	1.00	0.00	H
ATOM	316	HA	PHE	A	221	-4.274	-6.151	1.277	1.00	0.00	H
ATOM	317	HB2	PHE	A	221	-6.235	-5.512	2.595	1.00	0.00	H
ATOM	318	HB3	PHE	A	221	-5.242	-5.009	3.947	1.00	0.00	H
ATOM	319	HD1	PHE	A	221	-5.150	-2.852	4.060	1.00	0.00	H
ATOM	320	HD2	PHE	A	221	-5.673	-4.456	0.115	1.00	0.00	H
ATOM	321	HE1	PHE	A	221	-5.615	-0.572	3.189	1.00	0.00	H
ATOM	322	HE2	PHE	A	221	-5.867	-2.229	-0.788	1.00	0.00	H
ATOM	323	HZ	PHE	A	221	-6.059	-0.282	0.734	1.00	0.00	H
ATOM	324	N	GLY	A	222	-3.299	-8.305	2.503	1.00	0.00	N
ATOM	325	CA	GLY	A	222	-3.120	-9.665	3.009	1.00	0.00	C

ATOM	326	C	GLY	A	222	-2.759	-9.705	4.488	1.00	0.00	C
ATOM	327	O	GLY	A	222	-3.279	-10.526	5.301	1.00	0.00	O
ATOM	328	H	GLY	A	222	-2.612	-8.006	1.826	1.00	0.00	H
ATOM	329	HA2	GLY	A	222	-4.072	-10.183	2.894	1.00	0.00	H
ATOM	330	HA3	GLY	A	222	-2.323	-10.157	2.434	1.00	0.00	H
ATOM	331	N	GLY	A	223	-1.910	-8.803	5.005	1.00	0.00	N
ATOM	332	CA	GLY	A	223	-1.729	-8.717	6.480	1.00	0.00	C
ATOM	333	C	GLY	A	223	-2.829	-8.228	7.273	1.00	0.00	C
ATOM	334	O	GLY	A	223	-3.207	-8.773	8.325	1.00	0.00	O
ATOM	335	H	GLY	A	223	-1.395	-8.184	4.395	1.00	0.00	H
ATOM	336	HA2	GLY	A	223	-1.645	-9.744	6.836	1.00	0.00	H
ATOM	337	HA3	GLY	A	223	-0.808	-8.087	6.649	1.00	0.00	H
ATOM	338	N	HIS	A	224	-3.513	-7.146	6.800	1.00	0.00	N
ATOM	339	CA	HIS	A	224	-4.846	-6.705	7.182	1.00	0.00	C
ATOM	340	CB	HIS	A	224	-4.825	-5.649	8.288	1.00	0.00	C
ATOM	341	ND1	HIS	A	224	-3.449	-3.535	8.553	1.00	0.00	N
ATOM	342	CG	HIS	A	224	-4.072	-4.440	7.719	1.00	0.00	C
ATOM	343	CE1	HIS	A	224	-3.024	-2.496	7.780	1.00	0.00	C
ATOM	344	NE2	HIS	A	224	-3.415	-2.645	6.511	1.00	0.00	N
ATOM	345	CD2	HIS	A	224	-4.023	-3.856	6.481	1.00	0.00	C
ATOM	346	C	HIS	A	224	-5.921	-7.737	7.430	1.00	0.00	C
ATOM	347	O	HIS	A	224	-6.530	-7.820	8.496	1.00	0.00	O
ATOM	348	H	HIS	A	224	-3.156	-6.614	6.027	1.00	0.00	H
ATOM	349	HA	HIS	A	224	-5.207	-6.203	6.297	1.00	0.00	H
ATOM	350	HB2	HIS	A	224	-4.295	-6.114	9.143	1.00	0.00	H
ATOM	351	HB3	HIS	A	224	-5.845	-5.312	8.580	1.00	0.00	H
ATOM	352	HD1	HIS	A	224	-3.195	-3.699	9.512	1.00	0.00	H
ATOM	353	HD2	HIS	A	224	-4.514	-4.206	5.574	1.00	0.00	H
ATOM	354	HE1	HIS	A	224	-2.621	-1.658	8.345	1.00	0.00	H

ATOM	355	N	GLY	A	225	-6.407	-8.537	6.454	1.00	0.00	N
ATOM	356	CA	GLY	A	225	-7.460	-9.585	6.573	1.00	0.00	C
ATOM	357	C	GLY	A	225	-7.112	-10.908	7.421	1.00	0.00	C
ATOM	358	O	GLY	A	225	-7.994	-11.505	8.042	1.00	0.00	O
ATOM	359	H	GLY	A	225	-6.009	-8.409	5.535	1.00	0.00	H
ATOM	360	HA2	GLY	A	225	-8.248	-9.082	7.133	1.00	0.00	H
ATOM	361	HA3	GLY	A	225	-7.750	-9.920	5.557	1.00	0.00	H
ATOM	362	HC	GLY	A	225	-6.097	-11.276	7.446	1.00	0.00	H
ATOM	363	N	GLY	A	232	-2.265	-5.135	16.807	1.00	0.00	N
ATOM	364	CA	GLY	A	232	-2.164	-4.111	17.809	1.00	0.00	C
ATOM	365	C	GLY	A	232	-0.932	-3.286	17.792	1.00	0.00	C
ATOM	366	O	GLY	A	232	-0.677	-2.375	18.575	1.00	0.00	O
ATOM	367	H1	GLY	A	232	-3.010	-4.869	16.205	1.00	0.00	H
ATOM	368	H2	GLY	A	232	-2.235	-6.005	17.288	1.00	0.00	H
ATOM	369	HA2	GLY	A	232	-2.966	-3.408	17.582	1.00	0.00	H
ATOM	370	HA3	GLY	A	232	-2.267	-4.592	18.817	1.00	0.00	H
ATOM	371	N	ASN	A	233	-0.068	-3.646	16.867	1.00	0.00	N
ATOM	372	CA	ASN	A	233	1.092	-2.845	16.478	1.00	0.00	C
ATOM	373	CB	ASN	A	233	1.473	-2.952	15.015	1.00	0.00	C
ATOM	374	CG	ASN	A	233	0.330	-2.548	14.078	1.00	0.00	C
ATOM	375	OD1	ASN	A	233	0.286	-3.339	13.132	1.00	0.00	O
ATOM	376	ND2	ASN	A	233	-0.553	-1.562	14.232	1.00	0.00	N
ATOM	377	C	ASN	A	233	2.340	-3.222	17.307	1.00	0.00	C
ATOM	378	O	ASN	A	233	3.231	-2.371	17.383	1.00	0.00	O
ATOM	379	H	ASN	A	233	-0.123	-4.412	16.233	1.00	0.00	H
ATOM	380	HA	ASN	A	233	0.928	-1.819	16.745	1.00	0.00	H
ATOM	381	HB2	ASN	A	233	1.737	-3.972	14.659	1.00	0.00	H
ATOM	382	HB3	ASN	A	233	2.362	-2.280	14.862	1.00	0.00	H
ATOM	383	HD21	ASN	A	233	-0.409	-0.954	15.030	1.00	0.00	H

ATOM	384	HD22	ASN	A	233	-1.357	-1.530	13.660	1.00	0.00	H
ATOM	385	N	GLY	A	234	2.376	-4.393	17.895	1.00	0.00	N
ATOM	386	CA	GLY	A	234	3.486	-4.790	18.769	1.00	0.00	C
ATOM	387	C	GLY	A	234	4.553	-5.547	17.960	1.00	0.00	C
ATOM	388	O	GLY	A	234	4.329	-6.580	17.346	1.00	0.00	O
ATOM	389	H	GLY	A	234	1.617	-5.042	17.742	1.00	0.00	H
ATOM	390	HA2	GLY	A	234	3.931	-3.895	19.204	1.00	0.00	H
ATOM	391	HA3	GLY	A	234	3.111	-5.441	19.552	1.00	0.00	H
ATOM	392	HC	GLY	A	234	5.555	-5.144	17.933	1.00	0.00	H
ATOM	393	N	GLY	A	238	8.191	-7.114	9.126	1.00	0.00	N
ATOM	394	CA	GLY	A	238	7.960	-6.984	7.645	1.00	0.00	C
ATOM	395	C	GLY	A	238	7.589	-5.564	7.282	1.00	0.00	C
ATOM	396	O	GLY	A	238	7.470	-5.328	6.070	1.00	0.00	O
ATOM	397	H1	GLY	A	238	9.159	-7.347	9.198	1.00	0.00	H
ATOM	398	H2	GLY	A	238	7.467	-7.731	9.428	1.00	0.00	H
ATOM	399	HA2	GLY	A	238	8.873	-7.236	7.105	1.00	0.00	H
ATOM	400	HA3	GLY	A	238	7.156	-7.661	7.371	1.00	0.00	H
ATOM	401	N	TYR	A	239	7.456	-4.689	8.251	1.00	0.00	N
ATOM	402	CA	TYR	A	239	7.019	-3.352	8.058	1.00	0.00	C
ATOM	403	CB	TYR	A	239	5.475	-3.277	7.736	1.00	0.00	C
ATOM	404	CG	TYR	A	239	4.512	-3.771	8.815	1.00	0.00	C
ATOM	405	CD1	TYR	A	239	3.992	-2.902	9.812	1.00	0.00	C
ATOM	406	CE1	TYR	A	239	2.951	-3.398	10.680	1.00	0.00	C
ATOM	407	CZ	TYR	A	239	2.454	-4.734	10.547	1.00	0.00	C
ATOM	408	OH	TYR	A	239	1.350	-5.220	11.358	1.00	0.00	O
ATOM	409	CD2	TYR	A	239	4.085	-5.079	8.812	1.00	0.00	C
ATOM	410	CE2	TYR	A	239	3.043	-5.580	9.590	1.00	0.00	C
ATOM	411	C	TYR	A	239	7.842	-2.462	7.121	1.00	0.00	C
ATOM	412	O	TYR	A	239	7.246	-1.735	6.370	1.00	0.00	O

ATOM	413	H	TYR	A	239	7.742	-4.845	9.204	1.00	0.00	H
ATOM	414	HA	TYR	A	239	7.092	-2.805	8.986	1.00	0.00	H
ATOM	415	HB2	TYR	A	239	5.303	-3.825	6.787	1.00	0.00	H
ATOM	416	HB3	TYR	A	239	5.103	-2.246	7.507	1.00	0.00	H
ATOM	417	HD1	TYR	A	239	4.199	-1.847	9.669	1.00	0.00	H
ATOM	418	HD2	TYR	A	239	4.451	-5.724	8.030	1.00	0.00	H
ATOM	419	HE1	TYR	A	239	2.483	-2.835	11.488	1.00	0.00	H
ATOM	420	HE2	TYR	A	239	2.662	-6.597	9.503	1.00	0.00	H
ATOM	421	HH	TYR	A	239	1.024	-4.507	11.916	1.00	0.00	H
ATOM	422	N	GLY	A	240	9.192	-2.522	7.110	1.00	0.00	N
ATOM	423	CA	GLY	A	240	9.939	-1.920	6.061	1.00	0.00	C
ATOM	424	C	GLY	A	240	9.834	-2.624	4.730	1.00	0.00	C
ATOM	425	O	GLY	A	240	9.828	-2.023	3.637	1.00	0.00	O
ATOM	426	H	GLY	A	240	9.676	-3.000	7.856	1.00	0.00	H
ATOM	427	HA2	GLY	A	240	9.507	-0.928	5.925	1.00	0.00	H
ATOM	428	HA3	GLY	A	240	11.012	-1.875	6.351	1.00	0.00	H
ATOM	429	HC	GLY	A	240	9.760	-3.701	4.724	1.00	0.00	H
ATOM	430	N	GLY	A	242	7.577	-4.531	3.185	1.00	0.00	N
ATOM	431	CA	GLY	A	242	6.325	-4.352	2.498	1.00	0.00	C
ATOM	432	C	GLY	A	242	6.243	-2.945	1.884	1.00	0.00	C
ATOM	433	O	GLY	A	242	5.860	-2.869	0.726	1.00	0.00	O
ATOM	434	H1	GLY	A	242	8.058	-5.263	2.682	1.00	0.00	H
ATOM	435	H2	GLY	A	242	7.334	-4.662	4.157	1.00	0.00	H
ATOM	436	HA2	GLY	A	242	6.262	-5.100	1.708	1.00	0.00	H
ATOM	437	HA3	GLY	A	242	5.497	-4.472	3.197	1.00	0.00	H
ATOM	438	N	THR	A	243	6.639	-1.782	2.603	1.00	0.00	N
ATOM	439	CA	THR	A	243	6.929	-0.499	1.996	1.00	0.00	C
ATOM	440	CB	THR	A	243	7.264	0.511	3.054	1.00	0.00	C
ATOM	441	OG1	THR	A	243	6.320	0.360	4.080	1.00	0.00	O

```
ATOM    442  CG2 THR A 243       7.096   1.942   2.513  1.00  0.00           C
ATOM    443  C   THR A 243       8.019  -0.397   0.884  1.00  0.00           C
ATOM    444  O   THR A 243       7.832   0.297  -0.134  1.00  0.00           O
ATOM    445  H   THR A 243       6.964  -1.861   3.543  1.00  0.00           H
ATOM    446  HA  THR A 243       5.984  -0.181   1.581  1.00  0.00           H
ATOM    447  HB  THR A 243       8.295   0.399   3.417  1.00  0.00           H
ATOM    448 HG21 THR A 243       7.850   2.168   1.721  1.00  0.00           H
ATOM    449 HG22 THR A 243       7.216   2.714   3.295  1.00  0.00           H
ATOM    450 HG23 THR A 243       6.183   2.047   1.891  1.00  0.00           H
ATOM    451  HG1 THR A 243       5.480   0.167   3.644  1.00  0.00           H
ATOM    452  N   GLY A 244       9.137  -1.104   1.131  1.00  0.00           N
ATOM    453  CA  GLY A 244      10.277  -1.270   0.255  1.00  0.00           C
ATOM    454  C   GLY A 244      10.064  -1.983  -1.109  1.00  0.00           C
ATOM    455  O   GLY A 244      10.517  -1.448  -2.113  1.00  0.00           O
ATOM    456  H   GLY A 244       9.186  -1.570   2.026  1.00  0.00           H
ATOM    457  HA2 GLY A 244      10.532  -0.244  -0.009  1.00  0.00           H
ATOM    458  HA3 GLY A 244      11.079  -1.830   0.804  1.00  0.00           H
ATOM    459  HC  GLY A 244       9.533  -2.921  -1.170  1.00  0.00           H
TER     460      GLY A 244
HETATM  461  C1  RIS B 274       0.270   0.985   4.050  1.00  0.00           C
HETATM  462  C2  RIS B 274       0.150   2.168   5.084  1.00  0.00           C
HETATM  463  C3  RIS B 274       0.603   1.789   6.529  1.00  0.00           C
HETATM  464  C4  RIS B 274       2.800   2.845   6.026  1.00  0.00           C
HETATM  465  C5  RIS B 274       1.862   2.207   6.941  1.00  0.00           C
HETATM  466  N1  RIS B 274       2.327   1.837   8.196  1.00  0.00           N
HETATM  467  C6  RIS B 274      -0.300   1.107   7.456  1.00  0.00           C
HETATM  468  C7  RIS B 274       1.550   1.234   9.140  1.00  0.00           C
HETATM  469  N2  RIS B 274       0.233   0.893   8.685  1.00  0.00           N
HETATM  470  O1  RIS B 274      -1.494   0.896   7.191  1.00  0.00           O
```

HETATM	471	C8	RIS	B	274	2.170	0.550	10.369	1.00	0.00	C
HETATM	472	C9	RIS	B	274	-0.567	0.309	9.678	1.00	0.00	C
HETATM	473	C10	RIS	B	274	1.145	0.401	11.486	1.00	0.00	C
HETATM	474	C11	RIS	B	274	-0.031	-0.407	10.906	1.00	0.00	C
HETATM	475	N3	RIS	B	274	-0.186	1.363	2.625	1.00	0.00	N1+
HETATM	476	C12	RIS	B	274	-1.715	1.670	2.466	1.00	0.00	C
HETATM	477	C13	RIS	B	274	0.267	0.372	1.586	1.00	0.00	C
HETATM	478	C14	RIS	B	274	-2.434	0.526	1.933	1.00	0.00	C
HETATM	479	C15	RIS	B	274	-0.573	0.486	0.297	1.00	0.00	C
HETATM	480	C16	RIS	B	274	-2.056	0.158	0.459	1.00	0.00	C
HETATM	481	C17	RIS	B	274	-2.350	-1.257	-0.038	1.00	0.00	C
HETATM	482	N4	RIS	B	274	-2.225	-2.297	0.787	1.00	0.00	N
HETATM	483	C18	RIS	B	274	-2.735	-1.576	-1.334	1.00	0.00	C
HETATM	484	O2	RIS	B	274	-2.505	-3.395	-0.027	1.00	0.00	O
HETATM	485	C19	RIS	B	274	-2.754	-2.995	-1.286	1.00	0.00	C
HETATM	486	C20	RIS	B	274	-2.911	-0.950	-2.606	1.00	0.00	C
HETATM	487	C21	RIS	B	274	-3.078	-3.741	-2.403	1.00	0.00	C
HETATM	488	C22	RIS	B	274	-3.116	-1.736	-3.731	1.00	0.00	C
HETATM	489	C23	RIS	B	274	-3.177	-3.084	-3.614	1.00	0.00	C
HETATM	490	F	RIS	B	274	-3.408	-3.799	-4.719	1.00	0.00	F
HETATM	491	H1	RIS	B	274	-0.315	0.081	4.267	1.00	0.00	H
HETATM	492	H2	RIS	B	274	1.326	0.691	4.025	1.00	0.00	H
HETATM	493	H3	RIS	B	274	-0.883	2.543	5.064	1.00	0.00	H
HETATM	494	H4	RIS	B	274	0.758	3.028	4.706	1.00	0.00	H
HETATM	495	H5	RIS	B	274	2.368	3.780	5.615	1.00	0.00	H
HETATM	496	H6	RIS	B	274	3.101	2.189	5.186	1.00	0.00	H
HETATM	497	H7	RIS	B	274	3.760	3.022	6.555	1.00	0.00	H
HETATM	498	H8	RIS	B	274	3.098	1.075	10.657	1.00	0.00	H
HETATM	499	H9	RIS	B	274	2.467	-0.386	9.879	1.00	0.00	H

HETATM	500	H10	RIS	B	274	-1.136	-0.465	9.117	1.00	0.00	H
HETATM	501	H11	RIS	B	274	-1.211	1.177	9.955	1.00	0.00	H
HETATM	502	H12	RIS	B	274	0.823	1.375	11.864	1.00	0.00	H
HETATM	503	H13	RIS	B	274	1.563	-0.203	12.323	1.00	0.00	H
HETATM	504	H14	RIS	B	274	0.312	-1.455	10.738	1.00	0.00	H
HETATM	505	H15	RIS	B	274	-0.834	-0.537	11.676	1.00	0.00	H
HETATM	506	H16	RIS	B	274	-1.883	2.626	1.964	1.00	0.00	H
HETATM	507	H17	RIS	B	274	-2.005	1.750	3.524	1.00	0.00	H
HETATM	508	H18	RIS	B	274	0.288	-0.689	1.855	1.00	0.00	H
HETATM	509	H19	RIS	B	274	1.329	0.656	1.455	1.00	0.00	H
HETATM	510	H20	RIS	B	274	-3.493	0.833	1.907	1.00	0.00	H
HETATM	511	H21	RIS	B	274	-2.324	-0.419	2.509	1.00	0.00	H
HETATM	512	H22	RIS	B	274	-0.164	-0.215	-0.452	1.00	0.00	H
HETATM	513	H23	RIS	B	274	-0.529	1.501	-0.148	1.00	0.00	H
HETATM	514	H24	RIS	B	274	-2.626	0.838	-0.211	1.00	0.00	H
HETATM	515	H25	RIS	B	274	-2.943	0.132	-2.664	1.00	0.00	H
HETATM	516	H26	RIS	B	274	-3.048	-4.819	-2.415	1.00	0.00	H
HETATM	517	H27	RIS	B	274	-3.068	-1.286	-4.713	1.00	0.00	H
HETATM	518	H28	RIS	B	274	0.253	2.273	2.361	1.00	0.00	H
HETATM	519	OH2	TIP	X2644		0.760	-3.074	0.526	1.00	0.00	O
HETATM	520	H1	TIP	X2644		1.674	-2.935	0.323	1.00	0.00	H
HETATM	521	H2	TIP	X2644		0.419	-3.577	-0.219	1.00	0.00	H
HETATM	522	OH2	TIP	X4071		0.754	4.984	3.480	1.00	0.00	O
HETATM	523	H1	TIP	X4071		0.890	4.594	2.633	1.00	0.00	H
HETATM	524	H2	TIP	X4071		0.037	5.597	3.365	1.00	0.00	H
HETATM	525	OH2	TIP	X4261		-2.585	-0.591	4.859	1.00	0.00	O
HETATM	526	H1	TIP	X4261		-2.392	-0.098	5.657	1.00	0.00	H
HETATM	527	H2	TIP	X4261		-2.882	-1.436	5.172	1.00	0.00	H
HETATM	528	OH2	TIP	X5159		6.201	0.084	10.239	1.00	0.00	O

HETATM	529	H1	TIP	X5159	5.871	0.503	11.046	1.00	0.00	H
HETATM	530	H2	TIP	X5159	6.044	0.728	9.554	1.00	0.00	H
HETATM	531	OH2	TIP	X8828	3.875	0.616	2.627	1.00	0.00	O
HETATM	532	H1	TIP	X8828	3.891	0.636	1.663	1.00	0.00	H
HETATM	533	H2	TIP	X8828	3.944	1.522	2.885	1.00	0.00	H
HETATM	534	OH2	TIP	X9855	6.059	3.379	5.419	1.00	0.00	O
HETATM	535	H1	TIP	X9855	5.643	2.818	4.754	1.00	0.00	H
HETATM	536	H2	TIP	X9855	6.337	4.164	4.940	1.00	0.00	H
HETATM	537	OH2	TIP	X4450	3.584	6.067	10.292	1.00	0.00	O
HETATM	538	H1	TIP	X4450	4.404	5.633	10.084	1.00	0.00	H
HETATM	539	H2	TIP	X4450	3.837	6.958	10.520	1.00	0.00	H
HETATM	540	OH2	TIP	X5594	5.815	4.555	10.005	1.00	0.00	O
HETATM	541	H1	TIP	X5594	6.533	4.959	9.519	1.00	0.00	H
HETATM	542	H2	TIP	X5594	5.646	3.730	9.537	1.00	0.00	H
HETATM	543	OH2	TIP	X2425	6.074	0.641	6.784	1.00	0.00	O
HETATM	544	H1	TIP	X2425	6.603	-0.146	6.890	1.00	0.00	H
HETATM	545	H2	TIP	X2425	5.964	0.732	5.828	1.00	0.00	H
HETATM	546	OH2	TIP	X5128	3.448	3.234	2.913	1.00	0.00	O
HETATM	547	H1	TIP	X5128	2.894	3.334	2.140	1.00	0.00	H
HETATM	548	H2	TIP	X5128	3.236	3.977	3.465	1.00	0.00	H
HETATM	549	OH2	TIP	X5307	5.126	1.280	12.637	1.00	0.00	O
HETATM	550	H1	TIP	X5307	4.625	0.673	13.179	1.00	0.00	H
HETATM	551	H2	TIP	X5307	4.646	2.096	12.658	1.00	0.00	H
HETATM	552	OH2	TIP	X7504	-3.437	-2.135	11.717	1.00	0.00	O
HETATM	553	H1	TIP	X7504	-3.970	-2.909	11.856	1.00	0.00	H
HETATM	554	H2	TIP	X7504	-4.066	-1.406	11.738	1.00	0.00	H
HETATM	555	OH2	TIP	X 804	1.080	2.711	14.308	1.00	0.00	O
HETATM	556	H1	TIP	X 804	1.702	2.943	13.622	1.00	0.00	H
HETATM	557	H2	TIP	X 804	1.575	2.794	15.122	1.00	0.00	H

```
HETATM  558  OH2 TIP X2616      -6.665  -0.442  -5.455  1.00  0.00           O
HETATM  559  H1  TIP X2616      -6.523   0.367  -5.958  1.00  0.00           H
HETATM  560  H2  TIP X2616      -7.285  -0.952  -5.970  1.00  0.00           H
HETATM  561  OH2 TIP X7325       5.150   2.203   8.852  1.00  0.00           O
HETATM  562  H1  TIP X7325       4.201   2.088   8.831  1.00  0.00           H
HETATM  563  H2  TIP X7325       5.438   1.992   7.969  1.00  0.00           H
HETATM  564  OH2 TIP X1623      -0.501   0.878  15.904  1.00  0.00           O
HETATM  565  H1  TIP X1623      -0.093   1.430  15.238  1.00  0.00           H
HETATM  566  H2  TIP X1623      -1.407   1.159  15.935  1.00  0.00           H
HETATM  567  OH2 TIP X2815       3.726   1.862   0.256  1.00  0.00           O
HETATM  568  H1  TIP X2815       3.265   2.695   0.301  1.00  0.00           H
HETATM  569  H2  TIP X2815       4.498   2.042  -0.274  1.00  0.00           H
HETATM  570  OH2 TIP X3211       4.048  -0.217  15.010  1.00  0.00           O
HETATM  571  H1  TIP X3211       4.830  -0.306  15.547  1.00  0.00           H
HETATM  572  H2  TIP X3211       3.441   0.291  15.557  1.00  0.00           H
HETATM  573  OH2 TIP X6981       3.383   3.279  12.396  1.00  0.00           O
HETATM  574  H1  TIP X6981       3.859   3.955  12.881  1.00  0.00           H
HETATM  575  H2  TIP X6981       3.589   3.450  11.479  1.00  0.00           H
HETATM  576  OH2 TIP X8133      -5.926  -0.987  -2.877  1.00  0.00           O
HETATM  577  H1  TIP X8133      -6.206  -0.943  -3.793  1.00  0.00           H
HETATM  578  H2  TIP X8133      -5.074  -1.423  -2.921  1.00  0.00           H
HETATM  579  OH2 TIP X1631      -5.414  -6.870  -0.675  1.00  0.00           O
HETATM  580  H1  TIP X1631      -5.739  -6.158  -1.235  1.00  0.00           H
HETATM  581  H2  TIP X1631      -5.542  -7.655  -1.206  1.00  0.00           H
END
```

Risperidone (Cluster structure 3)

```
ATOM      1  N   GLY A  75      -4.793  13.157   5.669  1.00  0.00           N
ATOM      2  CA  GLY A  75      -3.849  12.758   4.655  1.00  0.00           C
```

ATOM	3	C	GLY	A	75	-3.399	11.353	4.781	1.00	0.00	C
ATOM	4	O	GLY	A	75	-3.595	10.629	3.803	1.00	0.00	O
ATOM	5	H1	GLY	A	75	-5.655	13.347	5.191	1.00	0.00	H
ATOM	6	H2	GLY	A	75	-4.335	13.862	6.217	1.00	0.00	H
ATOM	7	HA2	GLY	A	75	-4.358	12.831	3.694	1.00	0.00	H
ATOM	8	HA3	GLY	A	75	-2.971	13.434	4.704	1.00	0.00	H
ATOM	9	N	PHE	A	76	-2.899	10.954	5.942	1.00	0.00	N
ATOM	10	CA	PHE	A	76	-2.691	9.616	6.423	1.00	0.00	C
ATOM	11	CB	PHE	A	76	-1.645	9.656	7.576	1.00	0.00	C
ATOM	12	CG	PHE	A	76	-1.171	8.276	8.172	1.00	0.00	C
ATOM	13	CD1	PHE	A	76	0.110	7.665	7.970	1.00	0.00	C
ATOM	14	CE1	PHE	A	76	0.413	6.488	8.626	1.00	0.00	C
ATOM	15	CZ	PHE	A	76	-0.595	5.871	9.425	1.00	0.00	C
ATOM	16	CD2	PHE	A	76	-2.062	7.646	9.052	1.00	0.00	C
ATOM	17	CE2	PHE	A	76	-1.806	6.489	9.647	1.00	0.00	C
ATOM	18	C	PHE	A	76	-2.367	8.510	5.415	1.00	0.00	C
ATOM	19	O	PHE	A	76	-1.437	8.451	4.639	1.00	0.00	O
ATOM	20	H	PHE	A	76	-2.821	11.646	6.660	1.00	0.00	H
ATOM	21	HA	PHE	A	76	-3.684	9.333	6.749	1.00	0.00	H
ATOM	22	HB2	PHE	A	76	-1.892	10.321	8.440	1.00	0.00	H
ATOM	23	HB3	PHE	A	76	-0.722	10.090	7.153	1.00	0.00	H
ATOM	24	HD1	PHE	A	76	0.807	8.123	7.293	1.00	0.00	H
ATOM	25	HD2	PHE	A	76	-3.040	8.097	9.183	1.00	0.00	H
ATOM	26	HE1	PHE	A	76	1.352	6.016	8.406	1.00	0.00	H
ATOM	27	HE2	PHE	A	76	-2.583	6.033	10.243	1.00	0.00	H
ATOM	28	HZ	PHE	A	76	-0.407	4.860	9.763	1.00	0.00	H
ATOM	29	N	VAL	A	77	-3.301	7.581	5.405	1.00	0.00	N
ATOM	30	CA	VAL	A	77	-3.309	6.353	4.658	1.00	0.00	C
ATOM	31	CB	VAL	A	77	-3.876	5.210	5.451	1.00	0.00	C

ATOM	32	CG1	VAL	A	77	-2.788	4.914	6.507	1.00	0.00	C
ATOM	33	CG2	VAL	A	77	-5.233	5.524	6.202	1.00	0.00	C
ATOM	34	C	VAL	A	77	-4.253	6.506	3.458	1.00	0.00	C
ATOM	35	O	VAL	A	77	-4.293	5.704	2.515	1.00	0.00	O
ATOM	36	H	VAL	A	77	-4.136	7.667	5.951	1.00	0.00	H
ATOM	37	HA	VAL	A	77	-2.335	6.103	4.259	1.00	0.00	H
ATOM	38	HB	VAL	A	77	-3.987	4.312	4.790	1.00	0.00	H
ATOM	39	HG11	VAL	A	77	-2.574	5.842	7.057	1.00	0.00	H
ATOM	40	HG12	VAL	A	77	-1.888	4.553	5.971	1.00	0.00	H
ATOM	41	HG13	VAL	A	77	-3.073	4.154	7.260	1.00	0.00	H
ATOM	42	HG21	VAL	A	77	-5.443	4.635	6.830	1.00	0.00	H
ATOM	43	HG22	VAL	A	77	-6.075	5.861	5.568	1.00	0.00	H
ATOM	44	HG23	VAL	A	77	-4.972	6.412	6.834	1.00	0.00	H
ATOM	45	N	GLY	A	78	-4.928	7.653	3.349	1.00	0.00	N
ATOM	46	CA	GLY	A	78	-5.797	8.085	2.241	1.00	0.00	C
ATOM	47	C	GLY	A	78	-4.924	8.122	0.951	1.00	0.00	C
ATOM	48	O	GLY	A	78	-5.290	7.556	-0.075	1.00	0.00	O
ATOM	49	H	GLY	A	78	-4.831	8.301	4.117	1.00	0.00	H
ATOM	50	HA2	GLY	A	78	-6.640	7.403	2.132	1.00	0.00	H
ATOM	51	HA3	GLY	A	78	-6.167	9.072	2.434	1.00	0.00	H
ATOM	52	N	GLY	A	79	-3.776	8.814	1.142	1.00	0.00	N
ATOM	53	CA	GLY	A	79	-2.729	9.003	0.209	1.00	0.00	C
ATOM	54	C	GLY	A	79	-2.108	7.640	-0.225	1.00	0.00	C
ATOM	55	O	GLY	A	79	-1.842	7.403	-1.424	1.00	0.00	O
ATOM	56	H	GLY	A	79	-3.654	9.242	2.049	1.00	0.00	H
ATOM	57	HA2	GLY	A	79	-3.164	9.508	-0.653	1.00	0.00	H
ATOM	58	HA3	GLY	A	79	-1.927	9.606	0.643	1.00	0.00	H
ATOM	59	N	ASP	A	80	-1.915	6.700	0.732	1.00	0.00	N
ATOM	60	CA	ASP	A	80	-1.796	5.224	0.555	1.00	0.00	C

ATOM	61	CB	ASP	A	80	-0.406	4.621	0.495	1.00	0.00	C
ATOM	62	CG	ASP	A	80	0.549	4.817	1.642	1.00	0.00	C
ATOM	63	OD1	ASP	A	80	0.126	4.932	2.833	1.00	0.00	O
ATOM	64	OD2	ASP	A	80	1.791	4.747	1.391	1.00	0.00	O1-
ATOM	65	C	ASP	A	80	-2.718	4.580	-0.456	1.00	0.00	C
ATOM	66	O	ASP	A	80	-2.292	4.029	-1.491	1.00	0.00	O
ATOM	67	H	ASP	A	80	-2.209	6.899	1.671	1.00	0.00	H
ATOM	68	HA	ASP	A	80	-2.216	4.767	1.436	1.00	0.00	H
ATOM	69	HB2	ASP	A	80	0.156	4.919	-0.418	1.00	0.00	H
ATOM	70	HB3	ASP	A	80	-0.539	3.528	0.532	1.00	0.00	H
ATOM	71	N	GLY	A	81	-4.063	4.765	-0.230	1.00	0.00	N
ATOM	72	CA	GLY	A	81	-5.167	4.339	-1.021	1.00	0.00	C
ATOM	73	C	GLY	A	81	-5.219	4.976	-2.415	1.00	0.00	C
ATOM	74	O	GLY	A	81	-5.402	4.290	-3.424	1.00	0.00	O
ATOM	75	H	GLY	A	81	-4.299	5.272	0.611	1.00	0.00	H
ATOM	76	HA2	GLY	A	81	-5.035	3.265	-1.155	1.00	0.00	H
ATOM	77	HA3	GLY	A	81	-6.115	4.577	-0.501	1.00	0.00	H
ATOM	78	HC	GLY	A	81	-5.093	6.044	-2.515	1.00	0.00	H
ATOM	79	N	GLY	A	83	-2.838	6.745	-4.146	1.00	0.00	N
ATOM	80	CA	GLY	A	83	-1.681	6.408	-5.039	1.00	0.00	C
ATOM	81	C	GLY	A	83	-1.758	4.923	-5.508	1.00	0.00	C
ATOM	82	O	GLY	A	83	-1.521	4.528	-6.682	1.00	0.00	O
ATOM	83	H1	GLY	A	83	-3.341	7.425	-4.667	1.00	0.00	H
ATOM	84	H2	GLY	A	83	-2.407	6.936	-3.271	1.00	0.00	H
ATOM	85	HA2	GLY	A	83	-1.713	7.055	-5.916	1.00	0.00	H
ATOM	86	HA3	GLY	A	83	-0.761	6.554	-4.488	1.00	0.00	H
ATOM	87	N	CYS	A	84	-2.176	3.965	-4.630	1.00	0.00	N
ATOM	88	CA	CYS	A	84	-2.287	2.581	-5.133	1.00	0.00	C
ATOM	89	CB	CYS	A	84	-2.418	1.657	-3.941	1.00	0.00	C

ATOM	90	SG	CYS	A	84	-0.951	1.660	-2.881	1.00	0.00	S
ATOM	91	C	CYS	A	84	-3.451	2.430	-6.194	1.00	0.00	C
ATOM	92	O	CYS	A	84	-3.343	1.601	-7.104	1.00	0.00	O
ATOM	93	H	CYS	A	84	-2.383	4.130	-3.658	1.00	0.00	H
ATOM	94	HA	CYS	A	84	-1.342	2.331	-5.592	1.00	0.00	H
ATOM	95	HB2	CYS	A	84	-2.808	0.675	-4.282	1.00	0.00	H
ATOM	96	HB3	CYS	A	84	-3.316	2.048	-3.397	1.00	0.00	H
ATOM	97	HG	CYS	A	84	-1.112	2.870	-2.355	1.00	0.00	H
ATOM	98	N	GLY	A	85	-4.592	3.192	-6.036	1.00	0.00	N
ATOM	99	CA	GLY	A	85	-5.631	3.124	-7.109	1.00	0.00	C
ATOM	100	C	GLY	A	85	-5.030	3.634	-8.480	1.00	0.00	C
ATOM	101	O	GLY	A	85	-5.116	2.894	-9.456	1.00	0.00	O
ATOM	102	H	GLY	A	85	-4.720	3.776	-5.222	1.00	0.00	H
ATOM	103	HA2	GLY	A	85	-5.961	2.090	-7.213	1.00	0.00	H
ATOM	104	HA3	GLY	A	85	-6.459	3.760	-6.845	1.00	0.00	H
ATOM	105	HC	GLY	A	85	-4.559	4.603	-8.555	1.00	0.00	H
ATOM	106	N	GLY	A	148	-6.724	7.429	13.670	1.00	0.00	N
ATOM	107	CA	GLY	A	148	-5.891	7.865	12.585	1.00	0.00	C
ATOM	108	C	GLY	A	148	-5.927	6.894	11.378	1.00	0.00	C
ATOM	109	O	GLY	A	148	-5.341	7.214	10.294	1.00	0.00	O
ATOM	110	H1	GLY	A	148	-7.444	8.120	13.749	1.00	0.00	H
ATOM	111	H2	GLY	A	148	-6.098	7.230	14.425	1.00	0.00	H
ATOM	112	HA2	GLY	A	148	-6.281	8.831	12.265	1.00	0.00	H
ATOM	113	HA3	GLY	A	148	-4.849	7.947	12.922	1.00	0.00	H
ATOM	114	N	ILE	A	149	-6.576	5.709	11.410	1.00	0.00	N
ATOM	115	CA	ILE	A	149	-6.803	4.900	10.225	1.00	0.00	C
ATOM	116	CB	ILE	A	149	-8.226	4.347	10.165	1.00	0.00	C
ATOM	117	CG2	ILE	A	149	-9.269	5.536	10.079	1.00	0.00	C
ATOM	118	CG1	ILE	A	149	-8.562	3.247	9.088	1.00	0.00	C

ATOM	119	CD1	ILE	A	149	-8.444	3.681	7.614	1.00	0.00	C
ATOM	120	C	ILE	A	149	-5.758	3.751	10.310	1.00	0.00	C
ATOM	121	O	ILE	A	149	-5.178	3.272	9.314	1.00	0.00	O
ATOM	122	H	ILE	A	149	-6.970	5.349	12.238	1.00	0.00	H
ATOM	123	HA	ILE	A	149	-6.644	5.532	9.364	1.00	0.00	H
ATOM	124	HB	ILE	A	149	-8.463	3.771	11.093	1.00	0.00	H
ATOM	125	HG12	ILE	A	149	-9.596	2.880	9.237	1.00	0.00	H
ATOM	126	HG13	ILE	A	149	-7.903	2.363	9.258	1.00	0.00	H
ATOM	127	HG21	ILE	A	149	-9.155	6.357	10.832	1.00	0.00	H
ATOM	128	HG22	ILE	A	149	-10.242	5.038	10.283	1.00	0.00	H
ATOM	129	HG23	ILE	A	149	-9.324	6.077	9.108	1.00	0.00	H
ATOM	130	HD11	ILE	A	149	-8.451	2.825	6.913	1.00	0.00	H
ATOM	131	HD12	ILE	A	149	-7.554	4.339	7.552	1.00	0.00	H
ATOM	132	HD13	ILE	A	149	-9.176	4.497	7.415	1.00	0.00	H
ATOM	133	N	ILE	A	150	-5.441	3.324	11.560	1.00	0.00	N
ATOM	134	CA	ILE	A	150	-4.518	2.190	11.862	1.00	0.00	C
ATOM	135	CB	ILE	A	150	-4.220	2.012	13.294	1.00	0.00	C
ATOM	136	CG2	ILE	A	150	-2.997	1.018	13.536	1.00	0.00	C
ATOM	137	CG1	ILE	A	150	-3.831	3.407	14.011	1.00	0.00	C
ATOM	138	CD1	ILE	A	150	-2.578	4.136	13.382	1.00	0.00	C
ATOM	139	C	ILE	A	150	-4.842	0.862	11.184	1.00	0.00	C
ATOM	140	O	ILE	A	150	-4.092	0.282	10.424	1.00	0.00	O
ATOM	141	H	ILE	A	150	-5.889	3.757	12.329	1.00	0.00	H
ATOM	142	HA	ILE	A	150	-3.594	2.451	11.366	1.00	0.00	H
ATOM	143	HB	ILE	A	150	-5.034	1.609	13.937	1.00	0.00	H
ATOM	144	HG12	ILE	A	150	-3.624	3.111	15.075	1.00	0.00	H
ATOM	145	HG13	ILE	A	150	-4.750	4.025	14.126	1.00	0.00	H
ATOM	146	HG21	ILE	A	150	-2.235	1.178	12.750	1.00	0.00	H
ATOM	147	HG22	ILE	A	150	-3.406	-0.022	13.487	1.00	0.00	H

ATOM	148	HG23	ILE	A	150	-2.544	1.167	14.537	1.00	0.00	H
ATOM	149	HD11	ILE	A	150	-2.818	4.203	12.295	1.00	0.00	H
ATOM	150	HD12	ILE	A	150	-1.695	3.455	13.388	1.00	0.00	H
ATOM	151	HD13	ILE	A	150	-2.311	5.099	13.862	1.00	0.00	H
ATOM	152	N	GLY	A	151	-6.110	0.460	11.371	1.00	0.00	N
ATOM	153	CA	GLY	A	151	-6.640	-0.619	10.601	1.00	0.00	C
ATOM	154	C	GLY	A	151	-7.677	-0.273	9.624	1.00	0.00	C
ATOM	155	O	GLY	A	151	-7.391	0.033	8.487	1.00	0.00	O
ATOM	156	H	GLY	A	151	-6.689	0.922	12.057	1.00	0.00	H
ATOM	157	HA2	GLY	A	151	-5.808	-0.982	9.997	1.00	0.00	H
ATOM	158	HA3	GLY	A	151	-7.041	-1.394	11.309	1.00	0.00	H
ATOM	159	HC	GLY	A	151	-8.715	-0.291	9.923	1.00	0.00	H
ATOM	160	N	GLY	A	155	-12.551	-0.369	5.148	1.00	0.00	N
ATOM	161	CA	GLY	A	155	-12.019	0.608	4.150	1.00	0.00	C
ATOM	162	C	GLY	A	155	-10.804	-0.066	3.450	1.00	0.00	C
ATOM	163	O	GLY	A	155	-10.484	0.136	2.287	1.00	0.00	O
ATOM	164	H1	GLY	A	155	-13.486	-0.560	4.815	1.00	0.00	H
ATOM	165	H2	GLY	A	155	-12.417	0.101	6.032	1.00	0.00	H
ATOM	166	HA2	GLY	A	155	-12.786	0.884	3.426	1.00	0.00	H
ATOM	167	HA3	GLY	A	155	-11.686	1.486	4.660	1.00	0.00	H
ATOM	168	N	VAL	A	156	-10.097	-0.994	4.163	1.00	0.00	N
ATOM	169	CA	VAL	A	156	-9.056	-1.727	3.512	1.00	0.00	C
ATOM	170	CB	VAL	A	156	-8.247	-2.571	4.494	1.00	0.00	C
ATOM	171	CG1	VAL	A	156	-7.377	-3.655	3.873	1.00	0.00	C
ATOM	172	CG2	VAL	A	156	-7.568	-1.614	5.470	1.00	0.00	C
ATOM	173	C	VAL	A	156	-9.631	-2.644	2.416	1.00	0.00	C
ATOM	174	O	VAL	A	156	-9.075	-2.854	1.320	1.00	0.00	O
ATOM	175	H	VAL	A	156	-10.351	-1.158	5.106	1.00	0.00	H
ATOM	176	HA	VAL	A	156	-8.465	-0.965	3.053	1.00	0.00	H

ATOM	177	HB	VAL	A	156	-9.006	-3.036	5.180	1.00	0.00	H
ATOM	178	HG11	VAL	A	156	-7.019	-4.154	4.796	1.00	0.00	H
ATOM	179	HG12	VAL	A	156	-6.502	-3.190	3.388	1.00	0.00	H
ATOM	180	HG13	VAL	A	156	-7.925	-4.454	3.313	1.00	0.00	H
ATOM	181	HG21	VAL	A	156	-8.218	-0.917	6.027	1.00	0.00	H
ATOM	182	HG22	VAL	A	156	-6.756	-1.025	5.000	1.00	0.00	H
ATOM	183	HG23	VAL	A	156	-7.034	-2.251	6.210	1.00	0.00	H
ATOM	184	N	GLY	A	157	-10.792	-3.261	2.672	1.00	0.00	N
ATOM	185	CA	GLY	A	157	-11.406	-4.247	1.818	1.00	0.00	C
ATOM	186	C	GLY	A	157	-12.040	-3.582	0.595	1.00	0.00	C
ATOM	187	O	GLY	A	157	-11.922	-3.949	-0.572	1.00	0.00	O
ATOM	188	H	GLY	A	157	-11.273	-3.018	3.526	1.00	0.00	H
ATOM	189	HA2	GLY	A	157	-10.633	-4.942	1.490	1.00	0.00	H
ATOM	190	HA3	GLY	A	157	-12.183	-4.779	2.368	1.00	0.00	H
ATOM	191	N	GLY	A	158	-12.679	-2.415	0.825	1.00	0.00	N
ATOM	192	CA	GLY	A	158	-13.085	-1.351	-0.088	1.00	0.00	C
ATOM	193	C	GLY	A	158	-11.952	-1.068	-1.054	1.00	0.00	C
ATOM	194	O	GLY	A	158	-12.124	-1.217	-2.181	1.00	0.00	O
ATOM	195	H	GLY	A	158	-12.916	-2.250	1.793	1.00	0.00	H
ATOM	196	HA2	GLY	A	158	-13.972	-1.657	-0.642	1.00	0.00	H
ATOM	197	HA3	GLY	A	158	-13.305	-0.455	0.482	1.00	0.00	H
ATOM	198	N	SER	A	159	-10.775	-0.657	-0.572	1.00	0.00	N
ATOM	199	CA	SER	A	159	-9.599	-0.282	-1.399	1.00	0.00	C
ATOM	200	CB	SER	A	159	-8.451	0.404	-0.547	1.00	0.00	C
ATOM	201	OG	SER	A	159	-8.250	-0.286	0.721	1.00	0.00	O
ATOM	202	C	SER	A	159	-9.044	-1.470	-2.091	1.00	0.00	C
ATOM	203	O	SER	A	159	-8.646	-1.312	-3.271	1.00	0.00	O
ATOM	204	H	SER	A	159	-10.600	-0.494	0.396	1.00	0.00	H
ATOM	205	HA	SER	A	159	-9.920	0.370	-2.207	1.00	0.00	H

ATOM	206	HB2	SER	A	159	-8.685	1.466	-0.368	1.00	0.00	H
ATOM	207	HB3	SER	A	159	-7.522	0.380	-1.154	1.00	0.00	H
ATOM	208	HG	SER	A	159	-9.013	-0.152	1.296	1.00	0.00	H
ATOM	209	N	SER	A	160	-9.073	-2.700	-1.487	1.00	0.00	N
ATOM	210	CA	SER	A	160	-8.636	-4.008	-2.086	1.00	0.00	C
ATOM	211	CB	SER	A	160	-8.770	-5.254	-1.223	1.00	0.00	C
ATOM	212	OG	SER	A	160	-7.961	-5.172	-0.124	1.00	0.00	O
ATOM	213	C	SER	A	160	-9.429	-4.208	-3.380	1.00	0.00	C
ATOM	214	O	SER	A	160	-8.890	-4.535	-4.422	1.00	0.00	O
ATOM	215	H	SER	A	160	-9.371	-2.761	-0.539	1.00	0.00	H
ATOM	216	HA	SER	A	160	-7.622	-3.888	-2.446	1.00	0.00	H
ATOM	217	HB2	SER	A	160	-8.455	-6.067	-1.911	1.00	0.00	H
ATOM	218	HB3	SER	A	160	-9.845	-5.403	-0.932	1.00	0.00	H
ATOM	219	HG	SER	A	160	-8.158	-4.297	0.223	1.00	0.00	H
ATOM	220	N	GLY	A	161	-10.776	-4.099	-3.303	1.00	0.00	N
ATOM	221	CA	GLY	A	161	-11.683	-4.327	-4.448	1.00	0.00	C
ATOM	222	C	GLY	A	161	-11.408	-3.511	-5.634	1.00	0.00	C
ATOM	223	O	GLY	A	161	-11.281	-4.058	-6.753	1.00	0.00	O
ATOM	224	H	GLY	A	161	-11.184	-3.848	-2.414	1.00	0.00	H
ATOM	225	HA2	GLY	A	161	-11.529	-5.352	-4.786	1.00	0.00	H
ATOM	226	HA3	GLY	A	161	-12.721	-4.147	-4.089	1.00	0.00	H
ATOM	227	N	GLY	A	162	-10.998	-2.257	-5.502	1.00	0.00	N
ATOM	228	CA	GLY	A	162	-11.047	-1.256	-6.478	1.00	0.00	C
ATOM	229	C	GLY	A	162	-9.761	-1.032	-7.169	1.00	0.00	C
ATOM	230	O	GLY	A	162	-9.703	-0.266	-8.116	1.00	0.00	O
ATOM	231	H	GLY	A	162	-10.611	-1.998	-4.606	1.00	0.00	H
ATOM	232	HA2	GLY	A	162	-11.756	-1.600	-7.231	1.00	0.00	H
ATOM	233	HA3	GLY	A	162	-11.378	-0.298	-6.013	1.00	0.00	H
ATOM	234	N	SER	A	163	-8.727	-1.725	-6.660	1.00	0.00	N

ATOM	235	CA	SER	A	163	-7.401	-1.640	-7.226	1.00	0.00	C
ATOM	236	CB	SER	A	163	-6.570	-0.705	-6.367	1.00	0.00	C
ATOM	237	OG	SER	A	163	-6.536	-1.142	-5.010	1.00	0.00	O
ATOM	238	C	SER	A	163	-6.861	-2.996	-7.581	1.00	0.00	C
ATOM	239	O	SER	A	163	-6.895	-3.541	-8.643	1.00	0.00	O
ATOM	240	H	SER	A	163	-8.898	-2.455	-6.007	1.00	0.00	H
ATOM	241	HA	SER	A	163	-7.415	-1.090	-8.156	1.00	0.00	H
ATOM	242	HB2	SER	A	163	-7.034	0.293	-6.378	1.00	0.00	H
ATOM	243	HB3	SER	A	163	-5.509	-0.637	-6.695	1.00	0.00	H
ATOM	244	HG	SER	A	163	-7.413	-1.138	-4.612	1.00	0.00	H
ATOM	245	N	PHE	A	164	-6.435	-3.663	-6.461	1.00	0.00	N
ATOM	246	CA	PHE	A	164	-5.861	-5.005	-6.356	1.00	0.00	C
ATOM	247	CB	PHE	A	164	-5.457	-5.238	-4.881	1.00	0.00	C
ATOM	248	CG	PHE	A	164	-5.007	-6.584	-4.527	1.00	0.00	C
ATOM	249	CD1	PHE	A	164	-4.006	-7.316	-5.199	1.00	0.00	C
ATOM	250	CE1	PHE	A	164	-3.408	-8.442	-4.620	1.00	0.00	C
ATOM	251	CZ	PHE	A	164	-3.870	-9.037	-3.445	1.00	0.00	C
ATOM	252	CD2	PHE	A	164	-5.512	-7.218	-3.391	1.00	0.00	C
ATOM	253	CE2	PHE	A	164	-4.956	-8.414	-2.871	1.00	0.00	C
ATOM	254	C	PHE	A	164	-6.767	-6.125	-6.919	1.00	0.00	C
ATOM	255	O	PHE	A	164	-6.286	-7.018	-7.602	1.00	0.00	O
ATOM	256	H	PHE	A	164	-6.552	-3.195	-5.588	1.00	0.00	H
ATOM	257	HA	PHE	A	164	-4.981	-5.026	-6.984	1.00	0.00	H
ATOM	258	HB2	PHE	A	164	-6.254	-4.819	-4.236	1.00	0.00	H
ATOM	259	HB3	PHE	A	164	-4.551	-4.616	-4.821	1.00	0.00	H
ATOM	260	HD1	PHE	A	164	-3.472	-6.965	-6.064	1.00	0.00	H
ATOM	261	HD2	PHE	A	164	-6.354	-6.743	-2.919	1.00	0.00	H
ATOM	262	HE1	PHE	A	164	-2.467	-8.751	-5.047	1.00	0.00	H
ATOM	263	HE2	PHE	A	164	-5.398	-8.911	-2.029	1.00	0.00	H

ATOM	264	HZ	PHE	A	164	-3.539	-9.952	-2.968	1.00	0.00	H
ATOM	265	N	GLY	A	165	-8.130	-6.070	-6.700	1.00	0.00	N
ATOM	266	CA	GLY	A	165	-9.192	-6.929	-7.204	1.00	0.00	C
ATOM	267	C	GLY	A	165	-9.448	-6.735	-8.696	1.00	0.00	C
ATOM	268	O	GLY	A	165	-9.781	-7.683	-9.417	1.00	0.00	O
ATOM	269	H	GLY	A	165	-8.437	-5.319	-6.098	1.00	0.00	H
ATOM	270	HA2	GLY	A	165	-8.873	-7.961	-7.055	1.00	0.00	H
ATOM	271	HA3	GLY	A	165	-10.119	-6.715	-6.655	1.00	0.00	H
ATOM	272	HC	GLY	A	165	-9.336	-5.752	-9.130	1.00	0.00	H
ATOM	273	N	GLY	A	216	4.486	-8.171	-4.933	1.00	0.00	N
ATOM	274	CA	GLY	A	216	4.504	-7.194	-3.876	1.00	0.00	C
ATOM	275	C	GLY	A	216	3.280	-7.163	-2.973	1.00	0.00	C
ATOM	276	O	GLY	A	216	3.412	-6.969	-1.757	1.00	0.00	O
ATOM	277	H1	GLY	A	216	5.200	-8.821	-4.714	1.00	0.00	H
ATOM	278	H2	GLY	A	216	4.445	-7.656	-5.778	1.00	0.00	H
ATOM	279	HA2	GLY	A	216	5.334	-7.491	-3.235	1.00	0.00	H
ATOM	280	HA3	GLY	A	216	4.635	-6.180	-4.322	1.00	0.00	H
ATOM	281	N	TRP	A	217	2.087	-7.506	-3.538	1.00	0.00	N
ATOM	282	CA	TRP	A	217	0.820	-7.761	-2.855	1.00	0.00	C
ATOM	283	CB	TRP	A	217	0.300	-6.506	-2.196	1.00	0.00	C
ATOM	284	CG	TRP	A	217	0.010	-5.306	-3.063	1.00	0.00	C
ATOM	285	CD1	TRP	A	217	-1.220	-5.075	-3.628	1.00	0.00	C
ATOM	286	NE1	TRP	A	217	-1.167	-4.014	-4.445	1.00	0.00	N
ATOM	287	CE2	TRP	A	217	0.094	-3.470	-4.307	1.00	0.00	C
ATOM	288	CD2	TRP	A	217	0.848	-4.234	-3.419	1.00	0.00	C
ATOM	289	CE3	TRP	A	217	2.091	-3.942	-3.073	1.00	0.00	C
ATOM	290	CZ3	TRP	A	217	2.643	-2.819	-3.595	1.00	0.00	C
ATOM	291	CZ2	TRP	A	217	0.667	-2.351	-4.919	1.00	0.00	C
ATOM	292	CH2	TRP	A	217	1.952	-1.998	-4.484	1.00	0.00	C

ATOM	293	C	TRP	A	217	0.760	-8.907	-1.885	1.00	0.00	C
ATOM	294	O	TRP	A	217	0.139	-8.795	-0.847	1.00	0.00	O
ATOM	295	H	TRP	A	217	1.963	-7.682	-4.514	1.00	0.00	H
ATOM	296	HA	TRP	A	217	0.136	-8.030	-3.656	1.00	0.00	H
ATOM	297	HB2	TRP	A	217	-0.693	-6.776	-1.790	1.00	0.00	H
ATOM	298	HB3	TRP	A	217	0.883	-6.217	-1.298	1.00	0.00	H
ATOM	299	HD1	TRP	A	217	-2.069	-5.737	-3.597	1.00	0.00	H
ATOM	300	HE1	TRP	A	217	-1.906	-3.567	-4.911	1.00	0.00	H
ATOM	301	HE3	TRP	A	217	2.606	-4.569	-2.373	1.00	0.00	H
ATOM	302	HZ2	TRP	A	217	0.223	-1.731	-5.698	1.00	0.00	H
ATOM	303	HZ3	TRP	A	217	3.663	-2.608	-3.338	1.00	0.00	H
ATOM	304	HH2	TRP	A	217	2.371	-1.083	-4.870	1.00	0.00	H
ATOM	305	N	GLY	A	218	1.354	-10.047	-2.287	1.00	0.00	N
ATOM	306	CA	GLY	A	218	1.321	-11.336	-1.652	1.00	0.00	C
ATOM	307	C	GLY	A	218	2.627	-11.992	-1.091	1.00	0.00	C
ATOM	308	O	GLY	A	218	2.608	-13.224	-1.108	1.00	0.00	O
ATOM	309	H	GLY	A	218	1.890	-9.989	-3.141	1.00	0.00	H
ATOM	310	HA2	GLY	A	218	0.751	-11.104	-0.753	1.00	0.00	H
ATOM	311	HA3	GLY	A	218	0.863	-12.086	-2.368	1.00	0.00	H
ATOM	312	N	GLY	A	219	3.692	-11.285	-0.631	1.00	0.00	N
ATOM	313	CA	GLY	A	219	3.739	-9.835	-0.351	1.00	0.00	C
ATOM	314	C	GLY	A	219	2.910	-9.454	0.905	1.00	0.00	C
ATOM	315	O	GLY	A	219	2.738	-10.308	1.743	1.00	0.00	O
ATOM	316	H	GLY	A	219	4.541	-11.805	-0.463	1.00	0.00	H
ATOM	317	HA2	GLY	A	219	4.779	-9.568	-0.165	1.00	0.00	H
ATOM	318	HA3	GLY	A	219	3.339	-9.293	-1.209	1.00	0.00	H
ATOM	319	N	PHE	A	220	2.372	-8.163	0.852	1.00	0.00	N
ATOM	320	CA	PHE	A	220	1.921	-7.484	2.053	1.00	0.00	C
ATOM	321	CB	PHE	A	220	1.467	-5.992	1.865	1.00	0.00	C

ATOM	322	CG	PHE	A	220	2.566	-5.142	1.279	1.00	0.00	C
ATOM	323	CD1	PHE	A	220	3.908	-5.486	1.370	1.00	0.00	C
ATOM	324	CE1	PHE	A	220	4.854	-4.734	0.616	1.00	0.00	C
ATOM	325	CZ	PHE	A	220	4.448	-3.591	-0.041	1.00	0.00	C
ATOM	326	CD2	PHE	A	220	2.163	-3.982	0.532	1.00	0.00	C
ATOM	327	CE2	PHE	A	220	3.110	-3.165	-0.074	1.00	0.00	C
ATOM	328	C	PHE	A	220	0.722	-8.145	2.711	1.00	0.00	C
ATOM	329	O	PHE	A	220	0.577	-8.065	3.917	1.00	0.00	O
ATOM	330	H	PHE	A	220	2.634	-7.654	0.048	1.00	0.00	H
ATOM	331	HA	PHE	A	220	2.699	-7.523	2.786	1.00	0.00	H
ATOM	332	HB2	PHE	A	220	1.283	-5.300	2.718	1.00	0.00	H
ATOM	333	HB3	PHE	A	220	0.678	-5.994	1.073	1.00	0.00	H
ATOM	334	HD1	PHE	A	220	4.280	-6.416	1.768	1.00	0.00	H
ATOM	335	HD2	PHE	A	220	1.129	-3.698	0.398	1.00	0.00	H
ATOM	336	HE1	PHE	A	220	5.889	-4.990	0.719	1.00	0.00	H
ATOM	337	HE2	PHE	A	220	2.819	-2.222	-0.511	1.00	0.00	H
ATOM	338	HZ	PHE	A	220	5.178	-3.052	-0.630	1.00	0.00	H
ATOM	339	N	PHE	A	221	-0.107	-8.836	1.933	1.00	0.00	N
ATOM	340	CA	PHE	A	221	-1.178	-9.591	2.405	1.00	0.00	C
ATOM	341	CB	PHE	A	221	-2.493	-8.800	2.830	1.00	0.00	C
ATOM	342	CG	PHE	A	221	-2.845	-7.679	1.885	1.00	0.00	C
ATOM	343	CD1	PHE	A	221	-2.869	-7.776	0.497	1.00	0.00	C
ATOM	344	CE1	PHE	A	221	-3.094	-6.658	-0.286	1.00	0.00	C
ATOM	345	CZ	PHE	A	221	-3.504	-5.423	0.266	1.00	0.00	C
ATOM	346	CD2	PHE	A	221	-3.223	-6.417	2.416	1.00	0.00	C
ATOM	347	CE2	PHE	A	221	-3.460	-5.317	1.653	1.00	0.00	C
ATOM	348	C	PHE	A	221	-0.770	-10.749	3.482	1.00	0.00	C
ATOM	349	O	PHE	A	221	-1.477	-10.935	4.490	1.00	0.00	O
ATOM	350	H	PHE	A	221	0.075	-9.070	0.983	1.00	0.00	H

ATOM	351	HA	PHE	A	221	-1.396	-10.151	1.491	1.00	0.00	H
ATOM	352	HB2	PHE	A	221	-3.323	-9.528	2.724	1.00	0.00	H
ATOM	353	HB3	PHE	A	221	-2.314	-8.374	3.848	1.00	0.00	H
ATOM	354	HD1	PHE	A	221	-2.678	-8.763	0.074	1.00	0.00	H
ATOM	355	HD2	PHE	A	221	-3.201	-6.261	3.491	1.00	0.00	H
ATOM	356	HE1	PHE	A	221	-2.901	-6.715	-1.352	1.00	0.00	H
ATOM	357	HE2	PHE	A	221	-3.686	-4.395	2.158	1.00	0.00	H
ATOM	358	HZ	PHE	A	221	-3.788	-4.596	-0.368	1.00	0.00	H
ATOM	359	N	GLY	A	222	0.346	-11.451	3.282	1.00	0.00	N
ATOM	360	CA	GLY	A	222	0.763	-12.495	4.225	1.00	0.00	C
ATOM	361	C	GLY	A	222	1.077	-11.873	5.629	1.00	0.00	C
ATOM	362	O	GLY	A	222	0.583	-12.363	6.610	1.00	0.00	O
ATOM	363	H	GLY	A	222	0.913	-11.261	2.468	1.00	0.00	H
ATOM	364	HA2	GLY	A	222	-0.050	-13.216	4.315	1.00	0.00	H
ATOM	365	HA3	GLY	A	222	1.661	-12.980	3.861	1.00	0.00	H
ATOM	366	N	GLY	A	223	1.939	-10.789	5.642	1.00	0.00	N
ATOM	367	CA	GLY	A	223	2.157	-9.958	6.857	1.00	0.00	C
ATOM	368	C	GLY	A	223	0.888	-9.226	7.384	1.00	0.00	C
ATOM	369	O	GLY	A	223	0.676	-9.092	8.617	1.00	0.00	O
ATOM	370	H	GLY	A	223	2.438	-10.554	4.796	1.00	0.00	H
ATOM	371	HA2	GLY	A	223	2.461	-10.648	7.644	1.00	0.00	H
ATOM	372	HA3	GLY	A	223	2.925	-9.202	6.632	1.00	0.00	H
ATOM	373	N	HIS	A	224	-0.046	-8.814	6.456	1.00	0.00	N
ATOM	374	CA	HIS	A	224	-1.320	-8.207	6.838	1.00	0.00	C
ATOM	375	CB	HIS	A	224	-1.296	-6.857	7.661	1.00	0.00	C
ATOM	376	ND1	HIS	A	224	-0.273	-4.531	7.644	1.00	0.00	N
ATOM	377	CG	HIS	A	224	-0.529	-5.725	6.901	1.00	0.00	C
ATOM	378	CE1	HIS	A	224	0.252	-3.629	6.758	1.00	0.00	C
ATOM	379	NE2	HIS	A	224	0.371	-4.159	5.548	1.00	0.00	N

ATOM	380	CD2	HIS	A	224	-0.115	-5.460	5.667	1.00	0.00	C
ATOM	381	C	HIS	A	224	-2.202	-9.242	7.633	1.00	0.00	C
ATOM	382	O	HIS	A	224	-2.679	-9.047	8.730	1.00	0.00	O
ATOM	383	H	HIS	A	224	0.250	-8.814	5.500	1.00	0.00	H
ATOM	384	HA	HIS	A	224	-1.730	-7.936	5.882	1.00	0.00	H
ATOM	385	HB2	HIS	A	224	-0.752	-7.066	8.617	1.00	0.00	H
ATOM	386	HB3	HIS	A	224	-2.297	-6.433	7.853	1.00	0.00	H
ATOM	387	HD1	HIS	A	224	-0.405	-4.460	8.630	1.00	0.00	H
ATOM	388	HD2	HIS	A	224	-0.151	-6.011	4.735	1.00	0.00	H
ATOM	389	HE1	HIS	A	224	0.569	-2.690	7.200	1.00	0.00	H
ATOM	390	N	GLY	A	225	-2.294	-10.476	7.145	1.00	0.00	N
ATOM	391	CA	GLY	A	225	-3.104	-11.522	7.751	1.00	0.00	C
ATOM	392	C	GLY	A	225	-2.397	-12.165	8.884	1.00	0.00	C
ATOM	393	O	GLY	A	225	-3.037	-12.403	9.924	1.00	0.00	O
ATOM	394	H	GLY	A	225	-1.774	-10.699	6.309	1.00	0.00	H
ATOM	395	HA2	GLY	A	225	-4.019	-11.078	8.143	1.00	0.00	H
ATOM	396	HA3	GLY	A	225	-3.340	-12.271	6.984	1.00	0.00	H
ATOM	397	HC	GLY	A	225	-1.350	-12.421	8.808	1.00	0.00	H
ATOM	398	N	GLY	A	239	7.724	-3.750	8.336	1.00	0.00	N
ATOM	399	CA	GLY	A	239	6.558	-3.513	7.491	1.00	0.00	C
ATOM	400	C	GLY	A	239	6.691	-2.320	6.530	1.00	0.00	C
ATOM	401	O	GLY	A	239	6.467	-2.370	5.298	1.00	0.00	O
ATOM	402	H1	GLY	A	239	8.086	-4.620	8.035	1.00	0.00	H
ATOM	403	H2	GLY	A	239	7.416	-3.561	9.257	1.00	0.00	H
ATOM	404	HA2	GLY	A	239	6.456	-4.402	6.868	1.00	0.00	H
ATOM	405	HA3	GLY	A	239	5.676	-3.344	8.136	1.00	0.00	H
ATOM	406	N	SER	A	240	7.088	-1.172	7.055	1.00	0.00	N
ATOM	407	CA	SER	A	240	7.467	-0.010	6.219	1.00	0.00	C
ATOM	408	CB	SER	A	240	7.670	1.329	7.035	1.00	0.00	C

ATOM	409	OG	SER	A	240	8.578	1.215	8.123	1.00	0.00	O
ATOM	410	C	SER	A	240	8.715	-0.152	5.331	1.00	0.00	C
ATOM	411	O	SER	A	240	8.725	0.421	4.220	1.00	0.00	O
ATOM	412	H	SER	A	240	7.126	-1.111	8.044	1.00	0.00	H
ATOM	413	HA	SER	A	240	6.652	0.274	5.568	1.00	0.00	H
ATOM	414	HB2	SER	A	240	6.736	1.611	7.553	1.00	0.00	H
ATOM	415	HB3	SER	A	240	8.105	2.103	6.364	1.00	0.00	H
ATOM	416	HG	SER	A	240	8.143	0.577	8.679	1.00	0.00	H
ATOM	417	N	GLY	A	241	9.822	-0.833	5.730	1.00	0.00	N
ATOM	418	CA	GLY	A	241	10.946	-1.352	5.013	1.00	0.00	C
ATOM	419	C	GLY	A	241	10.435	-2.227	3.806	1.00	0.00	C
ATOM	420	O	GLY	A	241	10.905	-2.022	2.654	1.00	0.00	O
ATOM	421	H	GLY	A	241	9.858	-1.006	6.724	1.00	0.00	H
ATOM	422	HA2	GLY	A	241	11.544	-0.509	4.667	1.00	0.00	H
ATOM	423	HA3	GLY	A	241	11.547	-1.991	5.647	1.00	0.00	H
ATOM	424	N	GLY	A	242	9.385	-3.021	4.020	1.00	0.00	N
ATOM	425	CA	GLY	A	242	8.796	-3.807	2.938	1.00	0.00	C
ATOM	426	C	GLY	A	242	8.099	-2.961	1.898	1.00	0.00	C
ATOM	427	O	GLY	A	242	8.197	-2.999	0.650	1.00	0.00	O
ATOM	428	H	GLY	A	242	8.989	-3.080	4.947	1.00	0.00	H
ATOM	429	HA2	GLY	A	242	9.611	-4.324	2.431	1.00	0.00	H
ATOM	430	HA3	GLY	A	242	8.073	-4.517	3.371	1.00	0.00	H
ATOM	431	N	THR	A	243	7.314	-1.962	2.533	1.00	0.00	N
ATOM	432	CA	THR	A	243	6.425	-1.054	1.829	1.00	0.00	C
ATOM	433	CB	THR	A	243	5.570	-0.140	2.795	1.00	0.00	C
ATOM	434	OG1	THR	A	243	4.753	-0.874	3.711	1.00	0.00	O
ATOM	435	CG2	THR	A	243	4.572	0.782	2.009	1.00	0.00	C
ATOM	436	C	THR	A	243	7.281	-0.129	0.997	1.00	0.00	C
ATOM	437	O	THR	A	243	6.864	0.168	-0.100	1.00	0.00	O

ATOM	438	H	THR	A	243	7.246	-2.014	3.525	1.00	0.00	H
ATOM	439	HA	THR	A	243	5.705	-1.556	1.196	1.00	0.00	H
ATOM	440	HB	THR	A	243	6.246	0.474	3.424	1.00	0.00	H
ATOM	441	HG21	THR	A	243	4.050	0.193	1.232	1.00	0.00	H
ATOM	442	HG22	THR	A	243	5.147	1.604	1.517	1.00	0.00	H
ATOM	443	HG23	THR	A	243	3.767	1.230	2.623	1.00	0.00	H
ATOM	444	HG1	THR	A	243	5.347	-1.359	4.286	1.00	0.00	H
ATOM	445	N	GLY	A	244	8.443	0.375	1.566	1.00	0.00	N
ATOM	446	CA	GLY	A	244	9.485	1.062	0.825	1.00	0.00	C
ATOM	447	C	GLY	A	244	10.019	0.261	-0.330	1.00	0.00	C
ATOM	448	O	GLY	A	244	10.065	0.770	-1.468	1.00	0.00	O
ATOM	449	H	GLY	A	244	8.572	0.256	2.561	1.00	0.00	H
ATOM	450	HA2	GLY	A	244	9.042	1.965	0.405	1.00	0.00	H
ATOM	451	HA3	GLY	A	244	10.313	1.300	1.514	1.00	0.00	H
ATOM	452	HC	GLY	A	244	10.362	-0.751	-0.175	1.00	0.00	H
ATOM	453	N	GLY	A	246	8.490	-2.243	-1.944	1.00	0.00	N
ATOM	454	CA	GLY	A	246	7.391	-2.432	-2.823	1.00	0.00	C
ATOM	455	C	GLY	A	246	7.012	-1.215	-3.632	1.00	0.00	C
ATOM	456	O	GLY	A	246	6.530	-1.207	-4.789	1.00	0.00	O
ATOM	457	H1	GLY	A	246	9.423	-2.178	-2.325	1.00	0.00	H
ATOM	458	H2	GLY	A	246	8.358	-2.172	-0.948	1.00	0.00	H
ATOM	459	HA2	GLY	A	246	6.560	-2.816	-2.261	1.00	0.00	H
ATOM	460	HA3	GLY	A	246	7.697	-3.088	-3.627	1.00	0.00	H
ATOM	461	N	TYR	A	247	7.157	-0.047	-2.950	1.00	0.00	N
ATOM	462	CA	TYR	A	247	7.220	1.252	-3.545	1.00	0.00	C
ATOM	463	CB	TYR	A	247	5.782	1.717	-4.052	1.00	0.00	C
ATOM	464	CG	TYR	A	247	4.803	1.914	-2.871	1.00	0.00	C
ATOM	465	CD1	TYR	A	247	3.959	0.912	-2.517	1.00	0.00	C
ATOM	466	CE1	TYR	A	247	3.079	1.047	-1.493	1.00	0.00	C

ATOM	467	CZ	TYR	A	247	2.998	2.279	-0.787	1.00	0.00	C
ATOM	468	OH	TYR	A	247	1.956	2.528	0.090	1.00	0.00	O
ATOM	469	CD2	TYR	A	247	4.685	3.195	-2.247	1.00	0.00	C
ATOM	470	CE2	TYR	A	247	3.736	3.354	-1.215	1.00	0.00	C
ATOM	471	C	TYR	A	247	8.385	1.380	-4.575	1.00	0.00	C
ATOM	472	O	TYR	A	247	8.167	1.826	-5.684	1.00	0.00	O
ATOM	473	H	TYR	A	247	7.463	-0.042	-2.006	1.00	0.00	H
ATOM	474	HA	TYR	A	247	7.467	1.936	-2.756	1.00	0.00	H
ATOM	475	HB2	TYR	A	247	5.896	2.727	-4.506	1.00	0.00	H
ATOM	476	HB3	TYR	A	247	5.291	1.146	-4.849	1.00	0.00	H
ATOM	477	HD1	TYR	A	247	4.026	-0.013	-3.064	1.00	0.00	H
ATOM	478	HD2	TYR	A	247	5.404	3.955	-2.555	1.00	0.00	H
ATOM	479	HE1	TYR	A	247	2.370	0.264	-1.235	1.00	0.00	H
ATOM	480	HE2	TYR	A	247	3.806	4.234	-0.585	1.00	0.00	H
ATOM	481	HH	TYR	A	247	2.071	3.415	0.401	1.00	0.00	H
ATOM	482	N	GLY	A	248	9.628	0.985	-4.094	1.00	0.00	N
ATOM	483	CA	GLY	A	248	10.689	0.554	-4.955	1.00	0.00	C
ATOM	484	C	GLY	A	248	10.240	-0.284	-6.192	1.00	0.00	C
ATOM	485	O	GLY	A	248	10.325	0.202	-7.339	1.00	0.00	O
ATOM	486	H	GLY	A	248	9.790	1.003	-3.097	1.00	0.00	H
ATOM	487	HA2	GLY	A	248	11.128	1.477	-5.333	1.00	0.00	H
ATOM	488	HA3	GLY	A	248	11.412	-0.061	-4.383	1.00	0.00	H
ATOM	489	HC	GLY	A	248	9.856	-1.284	-6.056	1.00	0.00	H
TER	490		GLY	A	248						
HETATM	491	C1	RIS	B	274	1.078	0.866	5.381	1.00	0.00	C
HETATM	492	C2	RIS	B	274	0.038	1.194	6.496	1.00	0.00	C
HETATM	493	C3	RIS	B	274	0.779	1.728	7.798	1.00	0.00	C
HETATM	494	C4	RIS	B	274	-1.470	1.640	9.071	1.00	0.00	C
HETATM	495	C5	RIS	B	274	-0.045	2.004	8.928	1.00	0.00	C

HETATM	496	N1	RIS	B	274	0.489	2.397	10.152	1.00	0.00	N
HETATM	497	C6	RIS	B	274	2.145	1.825	7.923	1.00	0.00	C
HETATM	498	C7	RIS	B	274	1.823	2.635	10.262	1.00	0.00	C
HETATM	499	N2	RIS	B	274	2.617	2.361	9.142	1.00	0.00	N
HETATM	500	O1	RIS	B	274	3.016	1.598	7.133	1.00	0.00	O
HETATM	501	C8	RIS	B	274	2.274	3.166	11.571	1.00	0.00	C
HETATM	502	C9	RIS	B	274	4.095	2.344	9.353	1.00	0.00	C
HETATM	503	C10	RIS	B	274	3.760	2.961	11.858	1.00	0.00	C
HETATM	504	C11	RIS	B	274	4.659	3.096	10.565	1.00	0.00	C
HETATM	505	N3	RIS	B	274	0.315	0.306	4.149	1.00	0.00	N1+
HETATM	506	C12	RIS	B	274	-0.198	-1.102	4.312	1.00	0.00	C
HETATM	507	C13	RIS	B	274	0.890	0.696	2.732	1.00	0.00	C
HETATM	508	C14	RIS	B	274	-0.317	-1.825	2.924	1.00	0.00	C
HETATM	509	C15	RIS	B	274	-0.179	0.448	1.636	1.00	0.00	C
HETATM	510	C16	RIS	B	274	-0.949	-0.904	1.823	1.00	0.00	C
HETATM	511	C17	RIS	B	274	-1.314	-1.520	0.498	1.00	0.00	C
HETATM	512	N4	RIS	B	274	-0.313	-1.756	-0.310	1.00	0.00	N
HETATM	513	C18	RIS	B	274	-2.562	-1.779	-0.040	1.00	0.00	C
HETATM	514	O2	RIS	B	274	-0.927	-2.133	-1.506	1.00	0.00	O
HETATM	515	C19	RIS	B	274	-2.316	-2.038	-1.358	1.00	0.00	C
HETATM	516	C20	RIS	B	274	-3.846	-1.995	0.581	1.00	0.00	C
HETATM	517	C21	RIS	B	274	-3.450	-2.313	-2.195	1.00	0.00	C
HETATM	518	C22	RIS	B	274	-4.942	-2.133	-0.310	1.00	0.00	C
HETATM	519	C23	RIS	B	274	-4.679	-2.275	-1.652	1.00	0.00	C
HETATM	520	F	RIS	B	274	-5.617	-2.452	-2.530	1.00	0.00	F
HETATM	521	H1	RIS	B	274	1.812	0.069	5.549	1.00	0.00	H
HETATM	522	H2	RIS	B	274	1.575	1.746	4.986	1.00	0.00	H
HETATM	523	H3	RIS	B	274	-0.548	0.296	6.790	1.00	0.00	H
HETATM	524	H4	RIS	B	274	-0.559	2.081	6.216	1.00	0.00	H

HETATM	525	H5	RIS	B	274	-1.847	0.807	8.425	1.00	0.00	H
HETATM	526	H6	RIS	B	274	-2.108	2.532	8.891	1.00	0.00	H
HETATM	527	H7	RIS	B	274	-1.774	1.331	10.099	1.00	0.00	H
HETATM	528	H8	RIS	B	274	1.739	2.612	12.363	1.00	0.00	H
HETATM	529	H9	RIS	B	274	2.083	4.263	11.689	1.00	0.00	H
HETATM	530	H10	RIS	B	274	4.514	2.770	8.423	1.00	0.00	H
HETATM	531	H11	RIS	B	274	4.222	1.248	9.463	1.00	0.00	H
HETATM	532	H12	RIS	B	274	3.776	1.909	12.196	1.00	0.00	H
HETATM	533	H13	RIS	B	274	4.090	3.690	12.628	1.00	0.00	H
HETATM	534	H14	RIS	B	274	4.694	4.166	10.277	1.00	0.00	H
HETATM	535	H15	RIS	B	274	5.754	3.009	10.743	1.00	0.00	H
HETATM	536	H16	RIS	B	274	-1.167	-0.920	4.802	1.00	0.00	H
HETATM	537	H17	RIS	B	274	0.549	-1.511	5.017	1.00	0.00	H
HETATM	538	H18	RIS	B	274	1.734	0.002	2.618	1.00	0.00	H
HETATM	539	H19	RIS	B	274	1.179	1.754	2.809	1.00	0.00	H
HETATM	540	H20	RIS	B	274	-0.970	-2.704	3.087	1.00	0.00	H
HETATM	541	H21	RIS	B	274	0.697	-2.202	2.651	1.00	0.00	H
HETATM	542	H22	RIS	B	274	0.326	0.606	0.656	1.00	0.00	H
HETATM	543	H23	RIS	B	274	-0.870	1.318	1.586	1.00	0.00	H
HETATM	544	H24	RIS	B	274	-1.875	-0.485	2.296	1.00	0.00	H
HETATM	545	H25	RIS	B	274	-3.968	-1.911	1.647	1.00	0.00	H
HETATM	546	H26	RIS	B	274	-3.336	-2.695	-3.200	1.00	0.00	H
HETATM	547	H27	RIS	B	274	-5.906	-2.222	0.170	1.00	0.00	H
HETATM	548	H28	RIS	B	274	-0.608	0.763	3.960	1.00	0.00	H
HETATM	549	OH2	TIP	X1348		0.037	-0.768	13.550	1.00	0.00	O
HETATM	550	H1	TIP	X1348		-0.708	-1.308	13.799	1.00	0.00	H
HETATM	551	H2	TIP	X1348		0.357	-1.175	12.738	1.00	0.00	H
HETATM	552	OH2	TIP	X1381		4.286	5.876	5.418	1.00	0.00	O
HETATM	553	H1	TIP	X1381		3.733	5.102	5.288	1.00	0.00	H

HETATM	554	H2	TIP	X1381	4.216	6.363	4.600	1.00	0.00	H
HETATM	555	OH2	TIP	X6771	-0.139	-1.775	9.020	1.00	0.00	O
HETATM	556	H1	TIP	X6771	-0.811	-1.966	9.658	1.00	0.00	H
HETATM	557	H2	TIP	X6771	0.549	-1.327	9.513	1.00	0.00	H
HETATM	558	OH2	TIP	X7829	6.992	6.426	9.869	1.00	0.00	O
HETATM	559	H1	TIP	X7829	6.304	6.498	10.528	1.00	0.00	H
HETATM	560	H2	TIP	X7829	6.647	5.822	9.215	1.00	0.00	H
HETATM	561	OH2	TIP	X9249	-5.111	-0.914	7.190	1.00	0.00	O
HETATM	562	H1	TIP	X9249	-5.145	-0.306	6.448	1.00	0.00	H
HETATM	563	H2	TIP	X9249	-5.814	-0.623	7.761	1.00	0.00	H
HETATM	564	OH2	TIP	X 4	6.508	-0.024	12.401	1.00	0.00	O
HETATM	565	H1	TIP	X 4	5.585	-0.185	12.584	1.00	0.00	H
HETATM	566	H2	TIP	X 4	6.800	0.555	13.095	1.00	0.00	H
HETATM	567	OH2	TIP	X1249	-5.955	1.335	-3.728	1.00	0.00	O
HETATM	568	H1	TIP	X1249	-6.736	1.779	-4.065	1.00	0.00	H
HETATM	569	H2	TIP	X1249	-6.041	0.427	-4.048	1.00	0.00	H
HETATM	570	OH2	TIP	X7701	-3.882	-2.533	-5.505	1.00	0.00	O
HETATM	571	H1	TIP	X7701	-3.354	-1.803	-5.854	1.00	0.00	H
HETATM	572	H2	TIP	X7701	-4.705	-2.146	-5.250	1.00	0.00	H
HETATM	573	OH2	TIP	X8147	-1.583	2.842	3.334	1.00	0.00	O
HETATM	574	H1	TIP	X8147	-2.380	2.769	2.805	1.00	0.00	H
HETATM	575	H2	TIP	X8147	-1.240	3.707	3.138	1.00	0.00	H
HETATM	576	OH2	TIP	X8284	-2.614	-2.096	10.468	1.00	0.00	O
HETATM	577	H1	TIP	X8284	-3.180	-1.348	10.284	1.00	0.00	H
HETATM	578	H2	TIP	X8284	-2.488	-2.081	11.410	1.00	0.00	H
HETATM	579	OH2	TIP	X8507	5.157	2.567	5.647	1.00	0.00	O
HETATM	580	H1	TIP	X8507	4.795	3.033	4.885	1.00	0.00	H
HETATM	581	H2	TIP	X8507	4.387	2.175	6.070	1.00	0.00	H
HETATM	582	OH2	TIP	X3638	0.862	4.509	5.440	1.00	0.00	O

HETATM	583	H1	TIP	X3638	0.981	5.321	5.933	1.00	0.00	H
HETATM	584	H2	TIP	X3638	0.481	4.802	4.604	1.00	0.00	H
HETATM	585	OH2	TIP	X4276	2.413	-0.632	10.065	1.00	0.00	O
HETATM	586	H1	TIP	X4276	2.706	0.212	9.739	1.00	0.00	H
HETATM	587	H2	TIP	X4276	2.992	-0.820	10.800	1.00	0.00	H
HETATM	588	OH2	TIP	X5511	7.365	4.355	13.790	1.00	0.00	O
HETATM	589	H1	TIP	X5511	7.490	5.128	14.325	1.00	0.00	H
HETATM	590	H2	TIP	X5511	7.087	3.680	14.400	1.00	0.00	H
HETATM	591	OH2	TIP	X5597	0.748	3.124	14.834	1.00	0.00	O
HETATM	592	H1	TIP	X5597	0.497	3.360	15.722	1.00	0.00	H
HETATM	593	H2	TIP	X5597	-0.075	3.155	14.351	1.00	0.00	H
HETATM	594	OH2	TIP	X5776	-2.281	-1.955	6.965	1.00	0.00	O
HETATM	595	H1	TIP	X5776	-2.882	-1.226	7.031	1.00	0.00	H
HETATM	596	H2	TIP	X5776	-1.707	-1.871	7.721	1.00	0.00	H
HETATM	597	OH2	TIP	X6779	-3.882	-2.260	4.472	1.00	0.00	O
HETATM	598	H1	TIP	X6779	-3.326	-2.135	5.236	1.00	0.00	H
HETATM	599	H2	TIP	X6779	-4.467	-2.980	4.720	1.00	0.00	H
HETATM	600	OH2	TIP	X8881	2.557	6.985	12.351	1.00	0.00	O
HETATM	601	H1	TIP	X8881	3.431	7.092	11.969	1.00	0.00	H
HETATM	602	H2	TIP	X8881	2.009	7.621	11.881	1.00	0.00	H
HETATM	603	OH2	TIP	X2852	6.572	-1.050	9.756	1.00	0.00	O
HETATM	604	H1	TIP	X2852	6.666	-0.567	10.571	1.00	0.00	H
HETATM	605	H2	TIP	X2852	6.446	-1.955	10.046	1.00	0.00	H
HETATM	606	OH2	TIP	X4147	-7.042	1.078	2.741	1.00	0.00	O
HETATM	607	H1	TIP	X4147	-7.088	0.888	1.796	1.00	0.00	H
HETATM	608	H2	TIP	X4147	-6.205	0.729	3.015	1.00	0.00	H
HETATM	609	OH2	TIP	X9063	3.004	3.682	3.897	1.00	0.00	O
HETATM	610	H1	TIP	X9063	3.024	4.164	3.070	1.00	0.00	H
HETATM	611	H2	TIP	X9063	2.158	3.884	4.279	1.00	0.00	H

HETATM	612	OH2	TIP	X 287	5.231	6.555	12.142	1.00	0.00	O
HETATM	613	H1	TIP	X 287	5.356	5.700	12.554	1.00	0.00	H
HETATM	614	H2	TIP	X 287	5.133	7.157	12.870	1.00	0.00	H
HETATM	615	OH2	TIP	X 373	7.920	3.593	9.461	1.00	0.00	O
HETATM	616	H1	TIP	X 373	8.042	4.349	8.896	1.00	0.00	H
HETATM	617	H2	TIP	X 373	8.107	2.842	8.910	1.00	0.00	H
HETATM	618	OH2	TIP	X2541	6.051	2.946	15.823	1.00	0.00	O
HETATM	619	H1	TIP	X2541	6.060	2.838	16.777	1.00	0.00	H
HETATM	620	H2	TIP	X2541	5.134	3.142	15.623	1.00	0.00	H
HETATM	621	OH2	TIP	X2786	-4.711	1.887	7.107	1.00	0.00	O
HETATM	622	H1	TIP	X2786	-4.513	2.560	7.767	1.00	0.00	H
HETATM	623	H2	TIP	X2786	-5.502	1.452	7.422	1.00	0.00	H
HETATM	624	OH2	TIP	X3447	-4.399	0.144	3.280	1.00	0.00	O
HETATM	625	H1	TIP	X3447	-3.829	0.643	3.871	1.00	0.00	H
HETATM	626	H2	TIP	X3447	-4.274	-0.766	3.544	1.00	0.00	H
HETATM	627	OH2	TIP	X8042	-4.731	0.842	-1.162	1.00	0.00	O
HETATM	628	H1	TIP	X8042	-4.746	-0.105	-1.029	1.00	0.00	H
HETATM	629	H2	TIP	X8042	-5.200	1.001	-1.985	1.00	0.00	H
HETATM	630	OH2	TIP	X8176	1.763	0.331	15.426	1.00	0.00	O
HETATM	631	H1	TIP	X8176	1.422	-0.068	14.625	1.00	0.00	H
HETATM	632	H2	TIP	X8176	1.729	1.279	15.252	1.00	0.00	H
HETATM	633	OH2	TIP	X8244	3.421	3.377	15.832	1.00	0.00	O
HETATM	634	H1	TIP	X8244	2.788	3.368	16.554	1.00	0.00	H
HETATM	635	H2	TIP	X8244	2.977	3.839	15.129	1.00	0.00	H
HETATM	636	OH2	TIP	X1306	-2.917	1.017	5.171	1.00	0.00	O
HETATM	637	H1	TIP	X1306	-3.420	1.183	5.959	1.00	0.00	H
HETATM	638	H2	TIP	X1306	-2.564	1.870	4.925	1.00	0.00	H
HETATM	639	OH2	TIP	X1614	5.098	5.209	8.045	1.00	0.00	O
HETATM	640	H1	TIP	X1614	5.466	4.479	7.535	1.00	0.00	H

```
HETATM  641  H2  TIP X1614       4.638   5.744   7.389  1.00  0.00           H
HETATM  642  OH2 TIP X2068      -3.241   1.644   1.302  1.00  0.00           O
HETATM  643  H1  TIP X2068      -3.748   0.945   1.720  1.00  0.00           H
HETATM  644  H2  TIP X2068      -3.365   1.509   0.374  1.00  0.00           H
HETATM  645  OH2 TIP X4239       3.550  -0.684  12.774  1.00  0.00           O
HETATM  646  H1  TIP X4239       3.792  -1.378  13.382  1.00  0.00           H
HETATM  647  H2  TIP X4239       3.004  -0.094  13.286  1.00  0.00           H
END
```

Aripiprazole (Cluster structure 1)

```
ATOM      1  N   GLY A  55       8.031   9.377  -0.203  1.00  0.00           N
ATOM      2  CA  GLY A  55       7.284  10.135   0.798  1.00  0.00           C
ATOM      3  C   GLY A  55       7.406   9.737   2.311  1.00  0.00           C
ATOM      4  O   GLY A  55       8.466   9.940   2.911  1.00  0.00           O
ATOM      5  H1  GLY A  55       8.706   9.993  -0.525  1.00  0.00           H
ATOM      6  H2  GLY A  55       7.354   8.897  -0.704  1.00  0.00           H
ATOM      7  HA2 GLY A  55       7.753  11.117   0.744  1.00  0.00           H
ATOM      8  HA3 GLY A  55       6.193  10.103   0.534  1.00  0.00           H
ATOM      9  N   TRP A  56       6.315   9.248   2.912  1.00  0.00           N
ATOM     10  CA  TRP A  56       6.194   9.025   4.378  1.00  0.00           C
ATOM     11  CB  TRP A  56       5.991  10.402   5.218  1.00  0.00           C
ATOM     12  CG  TRP A  56       4.562  10.745   5.583  1.00  0.00           C
ATOM     13  CD1 TRP A  56       3.771  11.788   5.299  1.00  0.00           C
ATOM     14  NE1 TRP A  56       2.608  11.601   5.876  1.00  0.00           N
ATOM     15  CE2 TRP A  56       2.625  10.446   6.621  1.00  0.00           C
ATOM     16  CD2 TRP A  56       3.928   9.926   6.518  1.00  0.00           C
ATOM     17  CE3 TRP A  56       4.318   8.800   7.219  1.00  0.00           C
ATOM     18  CZ3 TRP A  56       3.401   8.247   8.131  1.00  0.00           C
ATOM     19  CZ2 TRP A  56       1.717   9.895   7.574  1.00  0.00           C
```

ATOM	20	CH2	TRP	A	56	2.105	8.832	8.352	1.00	0.00	C
ATOM	21	C	TRP	A	56	7.310	8.172	4.890	1.00	0.00	C
ATOM	22	O	TRP	A	56	8.033	8.388	5.865	1.00	0.00	O
ATOM	23	H	TRP	A	56	5.519	9.209	2.328	1.00	0.00	H
ATOM	24	HA	TRP	A	56	5.302	8.412	4.472	1.00	0.00	H
ATOM	25	HB2	TRP	A	56	6.591	10.420	6.156	1.00	0.00	H
ATOM	26	HB3	TRP	A	56	6.332	11.241	4.581	1.00	0.00	H
ATOM	27	HD1	TRP	A	56	3.992	12.577	4.608	1.00	0.00	H
ATOM	28	HE1	TRP	A	56	1.956	12.287	6.082	1.00	0.00	H
ATOM	29	HE3	TRP	A	56	5.286	8.334	7.249	1.00	0.00	H
ATOM	30	HZ2	TRP	A	56	0.840	10.479	7.824	1.00	0.00	H
ATOM	31	HZ3	TRP	A	56	3.710	7.494	8.844	1.00	0.00	H
ATOM	32	HH2	TRP	A	56	1.459	8.427	9.120	1.00	0.00	H
ATOM	33	N	VAL	A	57	7.568	7.146	4.138	1.00	0.00	N
ATOM	34	CA	VAL	A	57	8.680	6.309	4.529	1.00	0.00	C
ATOM	35	CB	VAL	A	57	8.668	5.032	3.826	1.00	0.00	C
ATOM	36	CG1	VAL	A	57	9.878	4.084	3.995	1.00	0.00	C
ATOM	37	CG2	VAL	A	57	7.460	4.226	4.414	1.00	0.00	C
ATOM	38	C	VAL	A	57	10.066	6.923	4.567	1.00	0.00	C
ATOM	39	O	VAL	A	57	10.750	6.871	5.581	1.00	0.00	O
ATOM	40	H	VAL	A	57	7.105	6.809	3.319	1.00	0.00	H
ATOM	41	HA	VAL	A	57	8.546	5.969	5.538	1.00	0.00	H
ATOM	42	HB	VAL	A	57	8.461	5.199	2.748	1.00	0.00	H
ATOM	43	HG11	VAL	A	57	9.620	3.022	3.763	1.00	0.00	H
ATOM	44	HG12	VAL	A	57	10.087	3.988	5.079	1.00	0.00	H
ATOM	45	HG13	VAL	A	57	10.829	4.360	3.495	1.00	0.00	H
ATOM	46	HG21	VAL	A	57	7.643	3.737	5.402	1.00	0.00	H
ATOM	47	HG22	VAL	A	57	7.283	3.308	3.810	1.00	0.00	H
ATOM	48	HG23	VAL	A	57	6.540	4.836	4.511	1.00	0.00	H

ATOM	49	N	GLY	A	58	10.493	7.704	3.535	1.00	0.00	N
ATOM	50	CA	GLY	A	58	11.881	8.306	3.513	1.00	0.00	C
ATOM	51	C	GLY	A	58	12.071	9.536	4.456	1.00	0.00	C
ATOM	52	O	GLY	A	58	13.163	9.706	4.989	1.00	0.00	O
ATOM	53	H	GLY	A	58	9.866	7.886	2.764	1.00	0.00	H
ATOM	54	HA2	GLY	A	58	12.561	7.537	3.879	1.00	0.00	H
ATOM	55	HA3	GLY	A	58	12.102	8.614	2.484	1.00	0.00	H
ATOM	56	N	GLY	A	59	11.036	10.266	4.744	1.00	0.00	N
ATOM	57	CA	GLY	A	59	11.088	11.187	5.894	1.00	0.00	C
ATOM	58	C	GLY	A	59	11.118	10.414	7.226	1.00	0.00	C
ATOM	59	O	GLY	A	59	11.933	10.596	8.147	1.00	0.00	O
ATOM	60	H	GLY	A	59	10.199	10.203	4.182	1.00	0.00	H
ATOM	61	HA2	GLY	A	59	11.998	11.782	5.817	1.00	0.00	H
ATOM	62	HA3	GLY	A	59	10.207	11.827	5.878	1.00	0.00	H
ATOM	63	N	LEU	A	60	10.193	9.385	7.405	1.00	0.00	N
ATOM	64	CA	LEU	A	60	10.148	8.497	8.548	1.00	0.00	C
ATOM	65	CB	LEU	A	60	9.402	9.212	9.750	1.00	0.00	C
ATOM	66	CG	LEU	A	60	7.897	9.455	9.505	1.00	0.00	C
ATOM	67	CD1	LEU	A	60	7.102	8.093	9.702	1.00	0.00	C
ATOM	68	CD2	LEU	A	60	7.324	10.524	10.391	1.00	0.00	C
ATOM	69	C	LEU	A	60	11.383	7.869	8.997	1.00	0.00	C
ATOM	70	O	LEU	A	60	11.727	7.732	10.179	1.00	0.00	O
ATOM	71	H	LEU	A	60	9.630	9.132	6.628	1.00	0.00	H
ATOM	72	HA	LEU	A	60	9.603	7.647	8.165	1.00	0.00	H
ATOM	73	HB2	LEU	A	60	9.910	10.122	10.099	1.00	0.00	H
ATOM	74	HB3	LEU	A	60	9.344	8.556	10.654	1.00	0.00	H
ATOM	75	HG	LEU	A	60	7.548	9.678	8.479	1.00	0.00	H
ATOM	76	HD11	LEU	A	60	6.059	8.285	10.069	1.00	0.00	H
ATOM	77	HD12	LEU	A	60	7.675	7.452	10.402	1.00	0.00	H

ATOM	78	HD13	LEU	A	60	7.038	7.500	8.769	1.00	0.00	H
ATOM	79	HD21	LEU	A	60	7.551	10.317	11.460	1.00	0.00	H
ATOM	80	HD22	LEU	A	60	6.221	10.589	10.240	1.00	0.00	H
ATOM	81	HD23	LEU	A	60	7.636	11.524	10.036	1.00	0.00	H
ATOM	82	N	GLY	A	61	12.131	7.491	7.972	1.00	0.00	N
ATOM	83	CA	GLY	A	61	13.427	6.862	8.173	1.00	0.00	C
ATOM	84	C	GLY	A	61	14.546	7.722	8.720	1.00	0.00	C
ATOM	85	O	GLY	A	61	15.565	7.178	9.126	1.00	0.00	O
ATOM	86	H	GLY	A	61	11.795	7.642	7.032	1.00	0.00	H
ATOM	87	HA2	GLY	A	61	13.251	6.125	8.956	1.00	0.00	H
ATOM	88	HA3	GLY	A	61	13.760	6.424	7.196	1.00	0.00	H
ATOM	89	HC	GLY	A	61	14.447	8.797	8.752	1.00	0.00	H
ATOM	90	N	GLY	A	65	10.333	8.377	15.282	1.00	0.00	N
ATOM	91	CA	GLY	A	65	9.642	8.633	16.554	1.00	0.00	C
ATOM	92	C	GLY	A	65	8.140	8.532	16.482	1.00	0.00	C
ATOM	93	O	GLY	A	65	7.451	8.994	17.376	1.00	0.00	O
ATOM	94	H1	GLY	A	65	10.844	7.540	15.429	1.00	0.00	H
ATOM	95	H2	GLY	A	65	10.748	9.243	15.035	1.00	0.00	H
ATOM	96	HA2	GLY	A	65	9.958	7.853	17.246	1.00	0.00	H
ATOM	97	HA3	GLY	A	65	9.923	9.645	16.904	1.00	0.00	H
ATOM	98	N	TRP	A	66	7.574	8.153	15.279	1.00	0.00	N
ATOM	99	CA	TRP	A	66	6.120	8.032	15.250	1.00	0.00	C
ATOM	100	CB	TRP	A	66	5.890	7.113	14.042	1.00	0.00	C
ATOM	101	CG	TRP	A	66	4.437	6.695	13.834	1.00	0.00	C
ATOM	102	CD1	TRP	A	66	3.289	6.852	14.509	1.00	0.00	C
ATOM	103	NE1	TRP	A	66	2.259	6.372	13.761	1.00	0.00	N
ATOM	104	CE2	TRP	A	66	2.685	5.799	12.629	1.00	0.00	C
ATOM	105	CD2	TRP	A	66	4.057	6.110	12.587	1.00	0.00	C
ATOM	106	CE3	TRP	A	66	4.832	5.734	11.394	1.00	0.00	C

ATOM	107	CZ3	TRP	A	66	4.112	5.146	10.372	1.00	0.00	C
ATOM	108	CZ2	TRP	A	66	1.999	5.278	11.567	1.00	0.00	C
ATOM	109	CH2	TRP	A	66	2.749	4.907	10.462	1.00	0.00	C
ATOM	110	C	TRP	A	66	5.462	9.477	15.127	1.00	0.00	C
ATOM	111	O	TRP	A	66	4.309	9.703	15.508	1.00	0.00	O
ATOM	112	H	TRP	A	66	8.085	7.702	14.557	1.00	0.00	H
ATOM	113	HA	TRP	A	66	5.786	7.575	16.184	1.00	0.00	H
ATOM	114	HB2	TRP	A	66	6.429	6.163	14.202	1.00	0.00	H
ATOM	115	HB3	TRP	A	66	6.137	7.622	13.083	1.00	0.00	H
ATOM	116	HD1	TRP	A	66	3.327	7.317	15.495	1.00	0.00	H
ATOM	117	HE1	TRP	A	66	1.358	6.260	14.126	1.00	0.00	H
ATOM	118	HE3	TRP	A	66	5.910	5.852	11.316	1.00	0.00	H
ATOM	119	HZ2	TRP	A	66	0.927	5.309	11.589	1.00	0.00	H
ATOM	120	HZ3	TRP	A	66	4.721	4.809	9.540	1.00	0.00	H
ATOM	121	HH2	TRP	A	66	2.186	4.491	9.621	1.00	0.00	H
ATOM	122	N	GLY	A	67	6.280	10.479	14.664	1.00	0.00	N
ATOM	123	CA	GLY	A	67	6.003	11.982	14.775	1.00	0.00	C
ATOM	124	C	GLY	A	67	5.486	12.580	13.488	1.00	0.00	C
ATOM	125	O	GLY	A	67	6.290	12.634	12.560	1.00	0.00	O
ATOM	126	H	GLY	A	67	7.139	10.201	14.211	1.00	0.00	H
ATOM	127	HA2	GLY	A	67	5.220	12.148	15.514	1.00	0.00	H
ATOM	128	HA3	GLY	A	67	6.942	12.460	15.066	1.00	0.00	H
ATOM	129	HC	GLY	A	67	4.472	12.938	13.389	1.00	0.00	H
ATOM	130	N	GLY	A	72	-3.865	12.404	9.558	1.00	0.00	N
ATOM	131	CA	GLY	A	72	-3.238	12.237	8.209	1.00	0.00	C
ATOM	132	C	GLY	A	72	-3.067	10.756	7.792	1.00	0.00	C
ATOM	133	O	GLY	A	72	-2.684	10.464	6.668	1.00	0.00	O
ATOM	134	H1	GLY	A	72	-4.732	12.829	9.362	1.00	0.00	H
ATOM	135	H2	GLY	A	72	-3.147	12.804	10.102	1.00	0.00	H

ATOM	136	HA2	GLY	A	72	-3.905	12.697	7.480	1.00	0.00	H
ATOM	137	HA3	GLY	A	72	-2.255	12.717	8.223	1.00	0.00	H
ATOM	138	N	CYS	A	73	-3.331	9.855	8.696	1.00	0.00	N
ATOM	139	CA	CYS	A	73	-3.259	8.393	8.474	1.00	0.00	C
ATOM	140	CB	CYS	A	73	-1.853	7.909	8.333	1.00	0.00	C
ATOM	141	SG	CYS	A	73	-1.642	6.202	8.888	1.00	0.00	S
ATOM	142	C	CYS	A	73	-4.171	8.102	7.212	1.00	0.00	C
ATOM	143	O	CYS	A	73	-3.716	7.601	6.159	1.00	0.00	O
ATOM	144	H	CYS	A	73	-3.607	10.087	9.627	1.00	0.00	H
ATOM	145	HA	CYS	A	73	-3.799	7.958	9.308	1.00	0.00	H
ATOM	146	HB2	CYS	A	73	-1.067	8.535	8.795	1.00	0.00	H
ATOM	147	HB3	CYS	A	73	-1.636	7.833	7.244	1.00	0.00	H
ATOM	148	HG	CYS	A	73	-0.366	5.838	8.733	1.00	0.00	H
ATOM	149	N	GLY	A	74	-5.426	8.531	7.301	1.00	0.00	N
ATOM	150	CA	GLY	A	74	-6.507	8.425	6.346	1.00	0.00	C
ATOM	151	C	GLY	A	74	-6.213	8.826	4.922	1.00	0.00	C
ATOM	152	O	GLY	A	74	-6.482	8.059	4.017	1.00	0.00	O
ATOM	153	H	GLY	A	74	-5.659	9.001	8.164	1.00	0.00	H
ATOM	154	HA2	GLY	A	74	-6.716	7.357	6.289	1.00	0.00	H
ATOM	155	HA3	GLY	A	74	-7.369	9.038	6.718	1.00	0.00	H
ATOM	156	N	GLY	A	75	-5.671	10.034	4.710	1.00	0.00	N
ATOM	157	CA	GLY	A	75	-5.330	10.395	3.339	1.00	0.00	C
ATOM	158	C	GLY	A	75	-4.141	9.681	2.757	1.00	0.00	C
ATOM	159	O	GLY	A	75	-4.096	9.366	1.579	1.00	0.00	O
ATOM	160	H	GLY	A	75	-5.504	10.673	5.475	1.00	0.00	H
ATOM	161	HA2	GLY	A	75	-6.178	10.085	2.728	1.00	0.00	H
ATOM	162	HA3	GLY	A	75	-5.148	11.492	3.301	1.00	0.00	H
ATOM	163	N	PHE	A	76	-3.132	9.504	3.619	1.00	0.00	N
ATOM	164	CA	PHE	A	76	-1.974	8.644	3.412	1.00	0.00	C

ATOM	165	CB	PHE	A	76	-1.010	9.454	2.482	1.00	0.00	C
ATOM	166	CG	PHE	A	76	0.418	8.721	2.382	1.00	0.00	C
ATOM	167	CD1	PHE	A	76	0.782	8.068	1.241	1.00	0.00	C
ATOM	168	CE1	PHE	A	76	2.093	7.468	1.151	1.00	0.00	C
ATOM	169	CZ	PHE	A	76	2.890	7.419	2.330	1.00	0.00	C
ATOM	170	CD2	PHE	A	76	1.256	8.896	3.444	1.00	0.00	C
ATOM	171	CE2	PHE	A	76	2.424	8.095	3.496	1.00	0.00	C
ATOM	172	C	PHE	A	76	-2.291	7.205	2.962	1.00	0.00	C
ATOM	173	O	PHE	A	76	-1.940	6.813	1.830	1.00	0.00	O
ATOM	174	H	PHE	A	76	-3.313	9.892	4.518	1.00	0.00	H
ATOM	175	HA	PHE	A	76	-1.500	8.506	4.370	1.00	0.00	H
ATOM	176	HB2	PHE	A	76	-0.945	10.461	2.948	1.00	0.00	H
ATOM	177	HB3	PHE	A	76	-1.414	9.468	1.452	1.00	0.00	H
ATOM	178	HD1	PHE	A	76	0.077	8.008	0.421	1.00	0.00	H
ATOM	179	HD2	PHE	A	76	0.821	9.429	4.277	1.00	0.00	H
ATOM	180	HE1	PHE	A	76	2.595	7.141	0.254	1.00	0.00	H
ATOM	181	HE2	PHE	A	76	3.034	8.081	4.395	1.00	0.00	H
ATOM	182	HZ	PHE	A	76	3.892	7.010	2.299	1.00	0.00	H
ATOM	183	N	VAL	A	77	-3.166	6.507	3.751	1.00	0.00	N
ATOM	184	CA	VAL	A	77	-3.694	5.156	3.358	1.00	0.00	C
ATOM	185	CB	VAL	A	77	-4.422	4.362	4.387	1.00	0.00	C
ATOM	186	CG1	VAL	A	77	-4.973	2.986	3.834	1.00	0.00	C
ATOM	187	CG2	VAL	A	77	-3.401	3.914	5.481	1.00	0.00	C
ATOM	188	C	VAL	A	77	-4.417	5.057	2.018	1.00	0.00	C
ATOM	189	O	VAL	A	77	-4.144	4.117	1.174	1.00	0.00	O
ATOM	190	H	VAL	A	77	-3.442	6.966	4.593	1.00	0.00	H
ATOM	191	HA	VAL	A	77	-2.798	4.613	3.157	1.00	0.00	H
ATOM	192	HB	VAL	A	77	-5.203	4.935	4.943	1.00	0.00	H
ATOM	193	HG11	VAL	A	77	-4.210	2.308	3.382	1.00	0.00	H

ATOM	194	HG12	VAL	A	77	-5.734	3.189	3.062	1.00	0.00	H
ATOM	195	HG13	VAL	A	77	-5.368	2.387	4.691	1.00	0.00	H
ATOM	196	HG21	VAL	A	77	-2.533	3.393	5.038	1.00	0.00	H
ATOM	197	HG22	VAL	A	77	-3.891	3.181	6.158	1.00	0.00	H
ATOM	198	HG23	VAL	A	77	-3.030	4.719	6.148	1.00	0.00	H
ATOM	199	N	GLY	A	78	-5.347	5.885	1.795	1.00	0.00	N
ATOM	200	CA	GLY	A	78	-6.065	6.119	0.523	1.00	0.00	C
ATOM	201	C	GLY	A	78	-5.214	6.413	-0.643	1.00	0.00	C
ATOM	202	O	GLY	A	78	-5.502	5.842	-1.706	1.00	0.00	O
ATOM	203	H	GLY	A	78	-5.633	6.454	2.579	1.00	0.00	H
ATOM	204	HA2	GLY	A	78	-6.561	5.185	0.261	1.00	0.00	H
ATOM	205	HA3	GLY	A	78	-6.780	6.956	0.694	1.00	0.00	H
ATOM	206	N	GLY	A	79	-4.169	7.283	-0.439	1.00	0.00	N
ATOM	207	CA	GLY	A	79	-3.260	7.618	-1.544	1.00	0.00	C
ATOM	208	C	GLY	A	79	-2.436	6.361	-1.891	1.00	0.00	C
ATOM	209	O	GLY	A	79	-2.174	5.971	-3.047	1.00	0.00	O
ATOM	210	H	GLY	A	79	-4.026	7.693	0.473	1.00	0.00	H
ATOM	211	HA2	GLY	A	79	-3.832	7.953	-2.410	1.00	0.00	H
ATOM	212	HA3	GLY	A	79	-2.590	8.402	-1.234	1.00	0.00	H
ATOM	213	N	ASP	A	80	-2.220	5.601	-0.881	1.00	0.00	N
ATOM	214	CA	ASP	A	80	-1.721	4.222	-0.978	1.00	0.00	C
ATOM	215	CB	ASP	A	80	-0.205	4.240	-1.282	1.00	0.00	C
ATOM	216	CG	ASP	A	80	0.456	2.884	-1.172	1.00	0.00	C
ATOM	217	OD1	ASP	A	80	1.537	2.623	-1.756	1.00	0.00	O
ATOM	218	OD2	ASP	A	80	-0.024	2.037	-0.330	1.00	0.00	O1-
ATOM	219	C	ASP	A	80	-2.554	3.321	-1.892	1.00	0.00	C
ATOM	220	O	ASP	A	80	-2.093	2.812	-2.884	1.00	0.00	O
ATOM	221	H	ASP	A	80	-2.242	5.975	0.035	1.00	0.00	H
ATOM	222	HA	ASP	A	80	-1.846	3.628	-0.089	1.00	0.00	H

ATOM	223	HB2	ASP	A	80	0.308	4.864	-0.522	1.00	0.00	H
ATOM	224	HB3	ASP	A	80	-0.117	4.813	-2.232	1.00	0.00	H
ATOM	225	N	GLY	A	81	-3.884	3.189	-1.565	1.00	0.00	N
ATOM	226	CA	GLY	A	81	-4.782	2.375	-2.335	1.00	0.00	C
ATOM	227	C	GLY	A	81	-4.912	3.018	-3.757	1.00	0.00	C
ATOM	228	O	GLY	A	81	-4.851	2.366	-4.822	1.00	0.00	O
ATOM	229	H	GLY	A	81	-4.240	3.680	-0.758	1.00	0.00	H
ATOM	230	HA2	GLY	A	81	-4.394	1.357	-2.381	1.00	0.00	H
ATOM	231	HA3	GLY	A	81	-5.761	2.359	-1.888	1.00	0.00	H
ATOM	232	N	GLY	A	82	-5.031	4.347	-3.903	1.00	0.00	N
ATOM	233	CA	GLY	A	82	-5.145	5.156	-5.140	1.00	0.00	C
ATOM	234	C	GLY	A	82	-3.934	4.883	-5.948	1.00	0.00	C
ATOM	235	O	GLY	A	82	-4.111	4.484	-7.133	1.00	0.00	O
ATOM	236	H	GLY	A	82	-5.045	4.874	-3.042	1.00	0.00	H
ATOM	237	HA2	GLY	A	82	-6.038	4.909	-5.714	1.00	0.00	H
ATOM	238	HA3	GLY	A	82	-5.193	6.196	-4.863	1.00	0.00	H
ATOM	239	N	GLY	A	83	-2.686	4.929	-5.394	1.00	0.00	N
ATOM	240	CA	GLY	A	83	-1.504	4.530	-6.088	1.00	0.00	C
ATOM	241	C	GLY	A	83	-1.463	3.126	-6.695	1.00	0.00	C
ATOM	242	O	GLY	A	83	-1.196	2.845	-7.884	1.00	0.00	O
ATOM	243	H	GLY	A	83	-2.595	5.262	-4.445	1.00	0.00	H
ATOM	244	HA2	GLY	A	83	-1.483	5.202	-6.946	1.00	0.00	H
ATOM	245	HA3	GLY	A	83	-0.622	4.628	-5.397	1.00	0.00	H
ATOM	246	N	CYS	A	84	-1.801	2.170	-5.722	1.00	0.00	N
ATOM	247	CA	CYS	A	84	-1.838	0.740	-5.960	1.00	0.00	C
ATOM	248	CB	CYS	A	84	-2.106	-0.036	-4.671	1.00	0.00	C
ATOM	249	SG	CYS	A	84	-0.685	0.078	-3.481	1.00	0.00	S
ATOM	250	C	CYS	A	84	-2.873	0.310	-7.011	1.00	0.00	C
ATOM	251	O	CYS	A	84	-2.520	-0.549	-7.795	1.00	0.00	O

ATOM	252	H	CYS	A	84	-2.069	2.609	-4.859	1.00	0.00	H
ATOM	253	HA	CYS	A	84	-0.887	0.429	-6.357	1.00	0.00	H
ATOM	254	HB2	CYS	A	84	-2.342	-1.091	-4.942	1.00	0.00	H
ATOM	255	HB3	CYS	A	84	-3.058	0.337	-4.216	1.00	0.00	H
ATOM	256	HG	CYS	A	84	-0.841	1.318	-3.083	1.00	0.00	H
ATOM	257	N	GLY	A	85	-4.082	0.834	-7.004	1.00	0.00	N
ATOM	258	CA	GLY	A	85	-5.128	0.528	-7.950	1.00	0.00	C
ATOM	259	C	GLY	A	85	-4.853	1.051	-9.320	1.00	0.00	C
ATOM	260	O	GLY	A	85	-5.130	0.500	-10.373	1.00	0.00	O
ATOM	261	H	GLY	A	85	-4.294	1.504	-6.278	1.00	0.00	H
ATOM	262	HA2	GLY	A	85	-5.184	-0.557	-8.032	1.00	0.00	H
ATOM	263	HA3	GLY	A	85	-6.079	0.954	-7.580	1.00	0.00	H
ATOM	264	N	GLY	A	86	-4.140	2.181	-9.381	1.00	0.00	N
ATOM	265	CA	GLY	A	86	-3.687	2.823	-10.637	1.00	0.00	C
ATOM	266	C	GLY	A	86	-2.632	2.046	-11.373	1.00	0.00	C
ATOM	267	O	GLY	A	86	-2.612	1.851	-12.607	1.00	0.00	O
ATOM	268	H	GLY	A	86	-3.893	2.628	-8.510	1.00	0.00	H
ATOM	269	HA2	GLY	A	86	-4.544	2.870	-11.309	1.00	0.00	H
ATOM	270	HA3	GLY	A	86	-3.301	3.826	-10.387	1.00	0.00	H
ATOM	271	N	SER	A	87	-1.722	1.593	-10.495	1.00	0.00	N
ATOM	272	CA	SER	A	87	-0.811	0.512	-10.885	1.00	0.00	C
ATOM	273	CB	SER	A	87	0.159	0.188	-9.718	1.00	0.00	C
ATOM	274	OG	SER	A	87	0.773	1.293	-9.142	1.00	0.00	O
ATOM	275	C	SER	A	87	-1.428	-0.743	-11.476	1.00	0.00	C
ATOM	276	O	SER	A	87	-1.187	-1.193	-12.650	1.00	0.00	O
ATOM	277	H	SER	A	87	-1.701	2.025	-9.599	1.00	0.00	H
ATOM	278	HA	SER	A	87	-0.146	0.897	-11.651	1.00	0.00	H
ATOM	279	HB2	SER	A	87	1.029	-0.418	-10.038	1.00	0.00	H
ATOM	280	HB3	SER	A	87	-0.372	-0.430	-8.957	1.00	0.00	H

ATOM	281	HG	SER	A	87	0.089	1.846	-8.762	1.00	0.00	H
ATOM	282	N	ILE	A	88	-2.218	-1.406	-10.667	1.00	0.00	N
ATOM	283	CA	ILE	A	88	-2.925	-2.590	-11.081	1.00	0.00	C
ATOM	284	C	ILE	A	88	-3.684	-2.418	-12.396	1.00	0.00	C
ATOM	285	O	ILE	A	88	-3.461	-3.180	-13.341	1.00	0.00	O
ATOM	286	CB	ILE	A	88	-3.852	-2.967	-9.943	1.00	0.00	C
ATOM	287	CG2	ILE	A	88	-4.849	-2.036	-9.586	1.00	0.00	C
ATOM	288	CG1	ILE	A	88	-4.653	-4.095	-10.326	1.00	0.00	C
ATOM	289	CD1	ILE	A	88	-5.957	-4.503	-9.745	1.00	0.00	C
ATOM	290	H	ILE	A	88	-2.362	-1.076	-9.714	1.00	0.00	H
ATOM	291	HA	ILE	A	88	-2.188	-3.382	-11.228	1.00	0.00	H
ATOM	292	HB	ILE	A	88	-3.252	-3.114	-9.036	1.00	0.00	H
ATOM	293	HG12	ILE	A	88	-4.846	-4.188	-11.373	1.00	0.00	H
ATOM	294	HG13	ILE	A	88	-3.800	-4.618	-10.002	1.00	0.00	H
ATOM	295	HG21	ILE	A	88	-5.622	-1.790	-10.294	1.00	0.00	H
ATOM	296	HG22	ILE	A	88	-5.337	-2.338	-8.691	1.00	0.00	H
ATOM	297	HG23	ILE	A	88	-4.153	-1.269	-9.381	1.00	0.00	H
ATOM	298	HD11	ILE	A	88	-6.731	-3.809	-10.069	1.00	0.00	H
ATOM	299	HD12	ILE	A	88	-6.075	-5.502	-10.142	1.00	0.00	H
ATOM	300	HD13	ILE	A	88	-6.038	-4.640	-8.689	1.00	0.00	H
ATOM	301	N	GLY	A	89	-4.577	-1.422	-12.430	1.00	0.00	N
ATOM	302	CA	GLY	A	89	-5.345	-1.075	-13.639	1.00	0.00	C
ATOM	303	C	GLY	A	89	-4.478	-0.952	-14.817	1.00	0.00	C
ATOM	304	O	GLY	A	89	-4.667	-1.349	-15.972	1.00	0.00	O
ATOM	305	H	GLY	A	89	-4.732	-0.884	-11.589	1.00	0.00	H
ATOM	306	HA2	GLY	A	89	-6.057	-1.871	-13.856	1.00	0.00	H
ATOM	307	HA3	GLY	A	89	-5.869	-0.129	-13.446	1.00	0.00	H
ATOM	308	N	GLY	A	90	-3.318	-0.245	-14.602	1.00	0.00	N
ATOM	309	CA	GLY	A	90	-2.363	-0.009	-15.651	1.00	0.00	C

ATOM	310	C	GLY	A	90	-1.892	-1.395	-16.239	1.00	0.00	C
ATOM	311	O	GLY	A	90	-1.882	-1.600	-17.463	1.00	0.00	O
ATOM	312	H	GLY	A	90	-3.131	0.120	-13.679	1.00	0.00	H
ATOM	313	HA2	GLY	A	90	-2.838	0.607	-16.414	1.00	0.00	H
ATOM	314	HA3	GLY	A	90	-1.489	0.496	-15.267	1.00	0.00	H
ATOM	315	N	LEU	A	91	-1.474	-2.193	-15.299	1.00	0.00	N
ATOM	316	CA	LEU	A	91	-0.942	-3.512	-15.647	1.00	0.00	C
ATOM	317	CB	LEU	A	91	-0.371	-4.300	-14.330	1.00	0.00	C
ATOM	318	CG	LEU	A	91	0.410	-5.625	-14.647	1.00	0.00	C
ATOM	319	CD1	LEU	A	91	1.615	-5.531	-15.549	1.00	0.00	C
ATOM	320	CD2	LEU	A	91	0.989	-6.021	-13.264	1.00	0.00	C
ATOM	321	C	LEU	A	91	-1.951	-4.404	-16.439	1.00	0.00	C
ATOM	322	O	LEU	A	91	-1.618	-5.077	-17.341	1.00	0.00	O
ATOM	323	H	LEU	A	91	-1.603	-2.004	-14.331	1.00	0.00	H
ATOM	324	HA	LEU	A	91	-0.154	-3.289	-16.342	1.00	0.00	H
ATOM	325	HB2	LEU	A	91	-1.303	-4.494	-13.753	1.00	0.00	H
ATOM	326	HB3	LEU	A	91	0.333	-3.629	-13.794	1.00	0.00	H
ATOM	327	HG	LEU	A	91	-0.331	-6.350	-15.053	1.00	0.00	H
ATOM	328	HD11	LEU	A	91	2.241	-6.438	-15.527	1.00	0.00	H
ATOM	329	HD12	LEU	A	91	2.286	-4.762	-15.108	1.00	0.00	H
ATOM	330	HD13	LEU	A	91	1.308	-5.219	-16.576	1.00	0.00	H
ATOM	331	HD21	LEU	A	91	1.890	-5.443	-12.936	1.00	0.00	H
ATOM	332	HD22	LEU	A	91	1.380	-7.039	-13.429	1.00	0.00	H
ATOM	333	HD23	LEU	A	91	0.317	-6.060	-12.390	1.00	0.00	H
ATOM	334	N	GLY	A	92	-3.276	-4.325	-16.110	1.00	0.00	N
ATOM	335	CA	GLY	A	92	-4.353	-5.012	-16.822	1.00	0.00	C
ATOM	336	C	GLY	A	92	-4.636	-4.444	-18.208	1.00	0.00	C
ATOM	337	O	GLY	A	92	-4.766	-5.235	-19.126	1.00	0.00	O
ATOM	338	H	GLY	A	92	-3.529	-3.751	-15.318	1.00	0.00	H

ATOM	339	HA2	GLY	A	92	-4.027	-6.042	-16.966	1.00	0.00	H
ATOM	340	HA3	GLY	A	92	-5.274	-4.951	-16.218	1.00	0.00	H
ATOM	341	HC	GLY	A	92	-4.718	-3.379	-18.371	1.00	0.00	H
ATOM	342	N	GLY	A	149	-6.455	3.423	9.212	1.00	0.00	N
ATOM	343	CA	GLY	A	149	-6.864	2.092	8.623	1.00	0.00	C
ATOM	344	C	GLY	A	149	-5.646	1.461	7.889	1.00	0.00	C
ATOM	345	O	GLY	A	149	-5.695	1.338	6.679	1.00	0.00	O
ATOM	346	H1	GLY	A	149	-6.547	3.273	10.195	1.00	0.00	H
ATOM	347	H2	GLY	A	149	-7.024	4.075	8.714	1.00	0.00	H
ATOM	348	HA2	GLY	A	149	-7.201	1.420	9.412	1.00	0.00	H
ATOM	349	HA3	GLY	A	149	-7.656	2.258	7.916	1.00	0.00	H
ATOM	350	N	ILE	A	150	-4.506	1.136	8.632	1.00	0.00	N
ATOM	351	CA	ILE	A	150	-3.284	0.582	8.096	1.00	0.00	C
ATOM	352	CB	ILE	A	150	-2.094	1.525	8.213	1.00	0.00	C
ATOM	353	CG2	ILE	A	150	-1.830	1.902	9.703	1.00	0.00	C
ATOM	354	CG1	ILE	A	150	-0.830	1.041	7.445	1.00	0.00	C
ATOM	355	CD1	ILE	A	150	0.204	2.130	7.339	1.00	0.00	C
ATOM	356	C	ILE	A	150	-2.986	-0.817	8.662	1.00	0.00	C
ATOM	357	O	ILE	A	150	-2.119	-1.510	8.146	1.00	0.00	O
ATOM	358	H	ILE	A	150	-4.716	1.525	9.530	1.00	0.00	H
ATOM	359	HA	ILE	A	150	-3.404	0.347	7.048	1.00	0.00	H
ATOM	360	HB	ILE	A	150	-2.366	2.443	7.665	1.00	0.00	H
ATOM	361	HG12	ILE	A	150	-1.169	0.853	6.406	1.00	0.00	H
ATOM	362	HG13	ILE	A	150	-0.400	0.092	7.810	1.00	0.00	H
ATOM	363	HG21	ILE	A	150	-1.520	1.025	10.310	1.00	0.00	H
ATOM	364	HG22	ILE	A	150	-2.797	2.312	10.055	1.00	0.00	H
ATOM	365	HG23	ILE	A	150	-1.066	2.698	9.856	1.00	0.00	H
ATOM	366	HD11	ILE	A	150	0.962	1.851	6.572	1.00	0.00	H
ATOM	367	HD12	ILE	A	150	0.708	2.382	8.304	1.00	0.00	H

ATOM	368	HD13	ILE	A	150	-0.209	3.113	7.074	1.00	0.00	H
ATOM	369	N	GLY	A	151	-3.841	-1.279	9.614	1.00	0.00	N
ATOM	370	CA	GLY	A	151	-3.905	-2.674	10.004	1.00	0.00	C
ATOM	371	C	GLY	A	151	-5.285	-3.236	10.295	1.00	0.00	C
ATOM	372	O	GLY	A	151	-6.257	-2.511	10.516	1.00	0.00	O
ATOM	373	H	GLY	A	151	-4.458	-0.622	10.069	1.00	0.00	H
ATOM	374	HA2	GLY	A	151	-3.556	-3.224	9.130	1.00	0.00	H
ATOM	375	HA3	GLY	A	151	-3.266	-2.820	10.908	1.00	0.00	H
ATOM	376	HC	GLY	A	151	-5.420	-4.307	10.309	1.00	0.00	H
ATOM	377	N	GLY	A	154	-9.736	-6.204	7.367	1.00	0.00	N
ATOM	378	CA	GLY	A	154	-10.328	-5.498	6.242	1.00	0.00	C
ATOM	379	C	GLY	A	154	-9.304	-4.701	5.526	1.00	0.00	C
ATOM	380	O	GLY	A	154	-9.275	-4.516	4.319	1.00	0.00	O
ATOM	381	H1	GLY	A	154	-9.839	-7.189	7.157	1.00	0.00	H
ATOM	382	H2	GLY	A	154	-10.178	-5.821	8.194	1.00	0.00	H
ATOM	383	HA2	GLY	A	154	-10.744	-6.221	5.541	1.00	0.00	H
ATOM	384	HA3	GLY	A	154	-11.119	-4.840	6.617	1.00	0.00	H
ATOM	385	N	PHE	A	155	-8.186	-4.355	6.243	1.00	0.00	N
ATOM	386	CA	PHE	A	155	-7.041	-3.820	5.592	1.00	0.00	C
ATOM	387	CB	PHE	A	155	-6.081	-3.063	6.498	1.00	0.00	C
ATOM	388	CG	PHE	A	155	-4.878	-2.342	5.772	1.00	0.00	C
ATOM	389	CD1	PHE	A	155	-3.568	-2.716	5.824	1.00	0.00	C
ATOM	390	CE1	PHE	A	155	-2.530	-2.099	5.135	1.00	0.00	C
ATOM	391	CZ	PHE	A	155	-2.823	-1.124	4.194	1.00	0.00	C
ATOM	392	CD2	PHE	A	155	-5.191	-1.325	4.859	1.00	0.00	C
ATOM	393	CE2	PHE	A	155	-4.208	-0.752	4.039	1.00	0.00	C
ATOM	394	C	PHE	A	155	-6.254	-4.807	4.663	1.00	0.00	C
ATOM	395	O	PHE	A	155	-5.904	-4.477	3.534	1.00	0.00	O
ATOM	396	H	PHE	A	155	-8.260	-4.367	7.232	1.00	0.00	H

ATOM	397	HA	PHE	A	155	-7.391	-3.065	4.908	1.00	0.00	H
ATOM	398	HB2	PHE	A	155	-5.642	-3.768	7.245	1.00	0.00	H
ATOM	399	HB3	PHE	A	155	-6.653	-2.352	7.157	1.00	0.00	H
ATOM	400	HD1	PHE	A	155	-3.219	-3.450	6.548	1.00	0.00	H
ATOM	401	HD2	PHE	A	155	-6.198	-0.920	4.769	1.00	0.00	H
ATOM	402	HE1	PHE	A	155	-1.503	-2.341	5.309	1.00	0.00	H
ATOM	403	HE2	PHE	A	155	-4.506	-0.020	3.297	1.00	0.00	H
ATOM	404	HZ	PHE	A	155	-2.043	-0.698	3.581	1.00	0.00	H
ATOM	405	N	GLY	A	156	-6.000	-6.056	5.192	1.00	0.00	N
ATOM	406	CA	GLY	A	156	-5.367	-7.065	4.356	1.00	0.00	C
ATOM	407	C	GLY	A	156	-6.060	-7.402	3.086	1.00	0.00	C
ATOM	408	O	GLY	A	156	-5.501	-7.428	1.990	1.00	0.00	O
ATOM	409	H	GLY	A	156	-6.250	-6.267	6.147	1.00	0.00	H
ATOM	410	HA2	GLY	A	156	-4.419	-6.631	4.037	1.00	0.00	H
ATOM	411	HA3	GLY	A	156	-5.246	-7.994	4.968	1.00	0.00	H
ATOM	412	HC	GLY	A	156	-7.113	-7.637	3.127	1.00	0.00	H
ATOM	413	N	GLY	A	162	-7.929	-5.028	-4.318	1.00	0.00	N
ATOM	414	CA	GLY	A	162	-7.996	-3.963	-5.342	1.00	0.00	C
ATOM	415	C	GLY	A	162	-6.809	-3.757	-6.219	1.00	0.00	C
ATOM	416	O	GLY	A	162	-6.885	-3.042	-7.251	1.00	0.00	O
ATOM	417	H1	GLY	A	162	-8.573	-5.704	-4.610	1.00	0.00	H
ATOM	418	H2	GLY	A	162	-7.922	-4.557	-3.460	1.00	0.00	H
ATOM	419	HA2	GLY	A	162	-8.760	-4.294	-6.045	1.00	0.00	H
ATOM	420	HA3	GLY	A	162	-8.236	-3.003	-4.809	1.00	0.00	H
ATOM	421	N	SER	A	163	-5.680	-4.345	-5.837	1.00	0.00	N
ATOM	422	CA	SER	A	163	-4.474	-4.305	-6.587	1.00	0.00	C
ATOM	423	CB	SER	A	163	-3.358	-3.692	-5.670	1.00	0.00	C
ATOM	424	OG	SER	A	163	-3.072	-4.466	-4.456	1.00	0.00	O
ATOM	425	C	SER	A	163	-4.062	-5.700	-7.187	1.00	0.00	C

ATOM	426	O	SER	A	163	-4.054	-5.935	-8.400	1.00	0.00	O
ATOM	427	H	SER	A	163	-5.781	-4.882	-5.010	1.00	0.00	H
ATOM	428	HA	SER	A	163	-4.599	-3.775	-7.514	1.00	0.00	H
ATOM	429	HB2	SER	A	163	-3.726	-2.689	-5.353	1.00	0.00	H
ATOM	430	HB3	SER	A	163	-2.464	-3.641	-6.353	1.00	0.00	H
ATOM	431	HG	SER	A	163	-3.761	-4.393	-3.788	1.00	0.00	H
ATOM	432	N	PHE	A	164	-3.827	-6.685	-6.309	1.00	0.00	N
ATOM	433	CA	PHE	A	164	-3.231	-7.958	-6.725	1.00	0.00	C
ATOM	434	CB	PHE	A	164	-2.512	-8.614	-5.487	1.00	0.00	C
ATOM	435	CG	PHE	A	164	-1.799	-9.909	-5.617	1.00	0.00	C
ATOM	436	CD1	PHE	A	164	-0.845	-9.982	-6.604	1.00	0.00	C
ATOM	437	CE1	PHE	A	164	-0.135	-11.191	-6.848	1.00	0.00	C
ATOM	438	CZ	PHE	A	164	-0.513	-12.380	-6.249	1.00	0.00	C
ATOM	439	CD2	PHE	A	164	-1.945	-11.016	-4.791	1.00	0.00	C
ATOM	440	CE2	PHE	A	164	-1.344	-12.275	-5.077	1.00	0.00	C
ATOM	441	C	PHE	A	164	-4.303	-8.991	-7.294	1.00	0.00	C
ATOM	442	O	PHE	A	164	-3.945	-9.782	-8.140	1.00	0.00	O
ATOM	443	H	PHE	A	164	-4.055	-6.484	-5.355	1.00	0.00	H
ATOM	444	HA	PHE	A	164	-2.481	-7.756	-7.461	1.00	0.00	H
ATOM	445	HB2	PHE	A	164	-3.225	-8.804	-4.670	1.00	0.00	H
ATOM	446	HB3	PHE	A	164	-1.768	-7.860	-5.112	1.00	0.00	H
ATOM	447	HD1	PHE	A	164	-0.442	-9.078	-7.041	1.00	0.00	H
ATOM	448	HD2	PHE	A	164	-2.561	-10.867	-3.912	1.00	0.00	H
ATOM	449	HE1	PHE	A	164	0.655	-11.319	-7.560	1.00	0.00	H
ATOM	450	HE2	PHE	A	164	-1.443	-13.192	-4.516	1.00	0.00	H
ATOM	451	HZ	PHE	A	164	-0.028	-13.311	-6.433	1.00	0.00	H
ATOM	452	N	GLY	A	165	-5.544	-8.960	-6.849	1.00	0.00	N
ATOM	453	CA	GLY	A	165	-6.594	-9.801	-7.375	1.00	0.00	C
ATOM	454	C	GLY	A	165	-6.958	-9.734	-8.815	1.00	0.00	C

ATOM	455	O	GLY	A	165	-7.327	-10.727	-9.425	1.00	0.00	O
ATOM	456	H	GLY	A	165	-5.771	-8.317	-6.104	1.00	0.00	H
ATOM	457	HA2	GLY	A	165	-6.166	-10.801	-7.298	1.00	0.00	H
ATOM	458	HA3	GLY	A	165	-7.529	-9.610	-6.757	1.00	0.00	H
ATOM	459	HC	GLY	A	165	-6.895	-8.788	-9.333	1.00	0.00	H
ATOM	460	N	GLY	A	212	7.474	-11.719	-13.355	1.00	0.00	N
ATOM	461	CA	GLY	A	212	7.604	-10.454	-12.616	1.00	0.00	C
ATOM	462	C	GLY	A	212	6.398	-9.991	-11.815	1.00	0.00	C
ATOM	463	O	GLY	A	212	6.604	-9.446	-10.725	1.00	0.00	O
ATOM	464	H1	GLY	A	212	8.086	-12.339	-12.906	1.00	0.00	H
ATOM	465	H2	GLY	A	212	7.495	-11.463	-14.301	1.00	0.00	H
ATOM	466	HA2	GLY	A	212	8.368	-10.647	-11.863	1.00	0.00	H
ATOM	467	HA3	GLY	A	212	7.877	-9.653	-13.345	1.00	0.00	H
ATOM	468	N	PHE	A	213	5.165	-10.260	-12.169	1.00	0.00	N
ATOM	469	CA	PHE	A	213	4.038	-9.931	-11.410	1.00	0.00	C
ATOM	470	CB	PHE	A	213	2.766	-9.558	-12.291	1.00	0.00	C
ATOM	471	CG	PHE	A	213	1.449	-9.553	-11.508	1.00	0.00	C
ATOM	472	CD1	PHE	A	213	0.418	-10.498	-11.848	1.00	0.00	C
ATOM	473	CE1	PHE	A	213	-0.813	-10.469	-11.162	1.00	0.00	C
ATOM	474	CZ	PHE	A	213	-0.985	-9.496	-10.125	1.00	0.00	C
ATOM	475	CD2	PHE	A	213	1.174	-8.521	-10.587	1.00	0.00	C
ATOM	476	CE2	PHE	A	213	0.010	-8.502	-9.848	1.00	0.00	C
ATOM	477	C	PHE	A	213	3.751	-10.954	-10.292	1.00	0.00	C
ATOM	478	O	PHE	A	213	3.340	-10.611	-9.181	1.00	0.00	O
ATOM	479	H	PHE	A	213	5.059	-10.592	-13.103	1.00	0.00	H
ATOM	480	HA	PHE	A	213	4.168	-9.004	-10.858	1.00	0.00	H
ATOM	481	HB2	PHE	A	213	2.577	-10.225	-13.156	1.00	0.00	H
ATOM	482	HB3	PHE	A	213	2.971	-8.548	-12.721	1.00	0.00	H
ATOM	483	HD1	PHE	A	213	0.556	-11.222	-12.646	1.00	0.00	H

ATOM	484	HD2	PHE	A	213	1.876	-7.689	-10.568	1.00	0.00	H
ATOM	485	HE1	PHE	A	213	-1.556	-11.152	-11.510	1.00	0.00	H
ATOM	486	HE2	PHE	A	213	-0.230	-7.690	-9.178	1.00	0.00	H
ATOM	487	HZ	PHE	A	213	-1.937	-9.431	-9.618	1.00	0.00	H
ATOM	488	N	GLY	A	214	4.043	-12.214	-10.602	1.00	0.00	N
ATOM	489	CA	GLY	A	214	4.038	-13.287	-9.541	1.00	0.00	C
ATOM	490	C	GLY	A	214	4.974	-13.000	-8.378	1.00	0.00	C
ATOM	491	O	GLY	A	214	4.551	-13.005	-7.205	1.00	0.00	O
ATOM	492	H	GLY	A	214	4.270	-12.455	-11.556	1.00	0.00	H
ATOM	493	HA2	GLY	A	214	3.036	-13.332	-9.113	1.00	0.00	H
ATOM	494	HA3	GLY	A	214	4.324	-14.231	-10.021	1.00	0.00	H
ATOM	495	HC	GLY	A	214	6.016	-12.790	-8.570	1.00	0.00	H
ATOM	496	N	GLY	A	216	6.980	-10.851	-5.867	1.00	0.00	N
ATOM	497	CA	GLY	A	216	6.779	-10.734	-4.458	1.00	0.00	C
ATOM	498	C	GLY	A	216	5.253	-10.700	-4.126	1.00	0.00	C
ATOM	499	O	GLY	A	216	4.861	-10.666	-2.952	1.00	0.00	O
ATOM	500	H1	GLY	A	216	7.438	-11.742	-6.001	1.00	0.00	H
ATOM	501	H2	GLY	A	216	7.445	-9.998	-6.144	1.00	0.00	H
ATOM	502	HA2	GLY	A	216	7.251	-11.596	-3.987	1.00	0.00	H
ATOM	503	HA3	GLY	A	216	7.220	-9.812	-4.083	1.00	0.00	H
ATOM	504	N	TRP	A	217	4.381	-10.902	-5.118	1.00	0.00	N
ATOM	505	CA	TRP	A	217	2.968	-11.068	-4.963	1.00	0.00	C
ATOM	506	CB	TRP	A	217	2.215	-9.740	-4.806	1.00	0.00	C
ATOM	507	CG	TRP	A	217	2.787	-8.591	-5.525	1.00	0.00	C
ATOM	508	CD1	TRP	A	217	2.784	-8.350	-6.877	1.00	0.00	C
ATOM	509	NE1	TRP	A	217	3.337	-7.081	-7.091	1.00	0.00	N
ATOM	510	CE2	TRP	A	217	3.604	-6.427	-5.952	1.00	0.00	C
ATOM	511	CD2	TRP	A	217	3.333	-7.384	-4.946	1.00	0.00	C
ATOM	512	CE3	TRP	A	217	3.445	-6.907	-3.597	1.00	0.00	C

ATOM	513	CZ3	TRP	A	217	4.069	-5.593	-3.338	1.00	0.00	C
ATOM	514	CZ2	TRP	A	217	4.287	-5.237	-5.766	1.00	0.00	C
ATOM	515	CH2	TRP	A	217	4.538	-4.826	-4.438	1.00	0.00	C
ATOM	516	C	TRP	A	217	2.474	-12.179	-3.972	1.00	0.00	C
ATOM	517	O	TRP	A	217	1.550	-12.034	-3.156	1.00	0.00	O
ATOM	518	H	TRP	A	217	4.677	-10.785	-6.061	1.00	0.00	H
ATOM	519	HA	TRP	A	217	2.734	-11.418	-5.962	1.00	0.00	H
ATOM	520	HB2	TRP	A	217	1.139	-9.878	-5.018	1.00	0.00	H
ATOM	521	HB3	TRP	A	217	2.211	-9.510	-3.713	1.00	0.00	H
ATOM	522	HD1	TRP	A	217	2.547	-9.110	-7.600	1.00	0.00	H
ATOM	523	HE1	TRP	A	217	3.146	-6.472	-7.823	1.00	0.00	H
ATOM	524	HE3	TRP	A	217	3.092	-7.507	-2.758	1.00	0.00	H
ATOM	525	HZ2	TRP	A	217	4.462	-4.644	-6.652	1.00	0.00	H
ATOM	526	HZ3	TRP	A	217	4.233	-5.241	-2.343	1.00	0.00	H
ATOM	527	HH2	TRP	A	217	4.948	-3.840	-4.287	1.00	0.00	H
ATOM	528	N	GLY	A	218	3.199	-13.324	-4.057	1.00	0.00	N
ATOM	529	CA	GLY	A	218	3.140	-14.522	-3.193	1.00	0.00	C
ATOM	530	C	GLY	A	218	3.482	-14.258	-1.745	1.00	0.00	C
ATOM	531	O	GLY	A	218	2.555	-13.955	-0.996	1.00	0.00	O
ATOM	532	H	GLY	A	218	3.869	-13.362	-4.812	1.00	0.00	H
ATOM	533	HA2	GLY	A	218	2.107	-14.871	-3.197	1.00	0.00	H
ATOM	534	HA3	GLY	A	218	3.836	-15.275	-3.601	1.00	0.00	H
ATOM	535	N	GLY	A	219	4.718	-14.292	-1.189	1.00	0.00	N
ATOM	536	CA	GLY	A	219	5.363	-13.044	-0.797	1.00	0.00	C
ATOM	537	C	GLY	A	219	4.638	-12.165	0.250	1.00	0.00	C
ATOM	538	O	GLY	A	219	4.418	-12.477	1.414	1.00	0.00	O
ATOM	539	H	GLY	A	219	5.188	-15.174	-1.047	1.00	0.00	H
ATOM	540	HA2	GLY	A	219	6.280	-13.367	-0.305	1.00	0.00	H
ATOM	541	HA3	GLY	A	219	5.527	-12.423	-1.710	1.00	0.00	H

ATOM	542	N	PHE	A	220	4.269	-10.971	-0.212	1.00	0.00	N
ATOM	543	CA	PHE	A	220	3.533	-9.984	0.470	1.00	0.00	C
ATOM	544	CB	PHE	A	220	3.612	-8.622	-0.269	1.00	0.00	C
ATOM	545	CG	PHE	A	220	2.948	-7.486	0.460	1.00	0.00	C
ATOM	546	CD1	PHE	A	220	3.512	-6.909	1.605	1.00	0.00	C
ATOM	547	CE1	PHE	A	220	2.942	-5.769	2.248	1.00	0.00	C
ATOM	548	CZ	PHE	A	220	2.039	-5.089	1.552	1.00	0.00	C
ATOM	549	CD2	PHE	A	220	1.976	-6.692	-0.184	1.00	0.00	C
ATOM	550	CE2	PHE	A	220	1.449	-5.586	0.373	1.00	0.00	C
ATOM	551	C	PHE	A	220	2.046	-10.371	0.810	1.00	0.00	C
ATOM	552	O	PHE	A	220	1.464	-10.076	1.895	1.00	0.00	O
ATOM	553	H	PHE	A	220	4.538	-10.701	-1.122	1.00	0.00	H
ATOM	554	HA	PHE	A	220	4.118	-9.828	1.361	1.00	0.00	H
ATOM	555	HB2	PHE	A	220	3.059	-8.812	-1.218	1.00	0.00	H
ATOM	556	HB3	PHE	A	220	4.669	-8.348	-0.464	1.00	0.00	H
ATOM	557	HD1	PHE	A	220	4.296	-7.411	2.149	1.00	0.00	H
ATOM	558	HD2	PHE	A	220	1.655	-6.900	-1.191	1.00	0.00	H
ATOM	559	HE1	PHE	A	220	3.464	-5.372	3.108	1.00	0.00	H
ATOM	560	HE2	PHE	A	220	0.664	-5.052	-0.133	1.00	0.00	H
ATOM	561	HZ	PHE	A	220	1.522	-4.228	1.967	1.00	0.00	H
ATOM	562	N	PHE	A	221	1.379	-11.037	-0.106	1.00	0.00	N
ATOM	563	CA	PHE	A	221	0.083	-11.757	0.024	1.00	0.00	C
ATOM	564	CB	PHE	A	221	-1.067	-10.731	0.135	1.00	0.00	C
ATOM	565	CG	PHE	A	221	-0.926	-9.515	-0.746	1.00	0.00	C
ATOM	566	CD1	PHE	A	221	-1.535	-8.281	-0.298	1.00	0.00	C
ATOM	567	CE1	PHE	A	221	-1.351	-7.091	-1.026	1.00	0.00	C
ATOM	568	CZ	PHE	A	221	-0.854	-7.173	-2.343	1.00	0.00	C
ATOM	569	CD2	PHE	A	221	-0.419	-9.587	-2.053	1.00	0.00	C
ATOM	570	CE2	PHE	A	221	-0.431	-8.345	-2.851	1.00	0.00	C

ATOM	571	C	PHE	A	221	0.044	-12.734	1.211	1.00	0.00	C
ATOM	572	O	PHE	A	221	-0.790	-12.726	2.166	1.00	0.00	O
ATOM	573	H	PHE	A	221	1.785	-11.211	-1.003	1.00	0.00	H
ATOM	574	HA	PHE	A	221	0.018	-12.329	-0.878	1.00	0.00	H
ATOM	575	HB2	PHE	A	221	-2.025	-11.271	-0.027	1.00	0.00	H
ATOM	576	HB3	PHE	A	221	-1.225	-10.388	1.177	1.00	0.00	H
ATOM	577	HD1	PHE	A	221	-1.978	-8.213	0.692	1.00	0.00	H
ATOM	578	HD2	PHE	A	221	-0.129	-10.495	-2.567	1.00	0.00	H
ATOM	579	HE1	PHE	A	221	-1.731	-6.129	-0.718	1.00	0.00	H
ATOM	580	HE2	PHE	A	221	0.075	-8.293	-3.813	1.00	0.00	H
ATOM	581	HZ	PHE	A	221	-0.853	-6.264	-2.938	1.00	0.00	H
ATOM	582	N	GLY	A	222	1.041	-13.652	1.230	1.00	0.00	N
ATOM	583	CA	GLY	A	222	1.258	-14.681	2.270	1.00	0.00	C
ATOM	584	C	GLY	A	222	1.516	-13.999	3.598	1.00	0.00	C
ATOM	585	O	GLY	A	222	0.950	-14.254	4.684	1.00	0.00	O
ATOM	586	H	GLY	A	222	1.697	-13.631	0.462	1.00	0.00	H
ATOM	587	HA2	GLY	A	222	0.379	-15.319	2.358	1.00	0.00	H
ATOM	588	HA3	GLY	A	222	2.116	-15.280	1.994	1.00	0.00	H
ATOM	589	HC	GLY	A	222	2.261	-13.217	3.608	1.00	0.00	H
ATOM	590	N	GLY	A	242	10.201	-7.368	0.664	1.00	0.00	N
ATOM	591	CA	GLY	A	242	9.220	-7.815	-0.264	1.00	0.00	C
ATOM	592	C	GLY	A	242	8.494	-6.714	-1.068	1.00	0.00	C
ATOM	593	O	GLY	A	242	8.307	-6.753	-2.297	1.00	0.00	O
ATOM	594	H1	GLY	A	242	11.073	-7.689	0.319	1.00	0.00	H
ATOM	595	H2	GLY	A	242	9.849	-7.587	1.565	1.00	0.00	H
ATOM	596	HA2	GLY	A	242	9.788	-8.401	-0.987	1.00	0.00	H
ATOM	597	HA3	GLY	A	242	8.441	-8.409	0.269	1.00	0.00	H
ATOM	598	N	THR	A	243	8.238	-5.534	-0.493	1.00	0.00	N
ATOM	599	CA	THR	A	243	7.647	-4.422	-1.192	1.00	0.00	C

ATOM	600	CB	THR	A	243	7.132	-3.363	-0.243	1.00	0.00	C
ATOM	601	OG1	THR	A	243	6.213	-3.902	0.729	1.00	0.00	O
ATOM	602	CG2	THR	A	243	6.294	-2.289	-0.811	1.00	0.00	C
ATOM	603	C	THR	A	243	8.579	-3.814	-2.196	1.00	0.00	C
ATOM	604	O	THR	A	243	8.242	-3.468	-3.405	1.00	0.00	O
ATOM	605	H	THR	A	243	8.490	-5.356	0.463	1.00	0.00	H
ATOM	606	HA	THR	A	243	6.742	-4.727	-1.711	1.00	0.00	H
ATOM	607	HB	THR	A	243	7.966	-2.973	0.350	1.00	0.00	H
ATOM	608	HG21	THR	A	243	6.755	-1.629	-1.567	1.00	0.00	H
ATOM	609	HG22	THR	A	243	5.747	-1.647	-0.088	1.00	0.00	H
ATOM	610	HG23	THR	A	243	5.568	-2.823	-1.464	1.00	0.00	H
ATOM	611	HG1	THR	A	243	6.764	-4.427	1.304	1.00	0.00	H
ATOM	612	N	GLY	A	244	9.908	-3.698	-1.841	1.00	0.00	N
ATOM	613	CA	GLY	A	244	11.117	-3.280	-2.655	1.00	0.00	C
ATOM	614	C	GLY	A	244	11.318	-4.170	-3.855	1.00	0.00	C
ATOM	615	O	GLY	A	244	11.391	-3.672	-4.962	1.00	0.00	O
ATOM	616	H	GLY	A	244	10.108	-3.930	-0.879	1.00	0.00	H
ATOM	617	HA2	GLY	A	244	10.969	-2.269	-3.035	1.00	0.00	H
ATOM	618	HA3	GLY	A	244	11.987	-3.328	-1.999	1.00	0.00	H
ATOM	619	HC	GLY	A	244	11.396	-5.240	-3.733	1.00	0.00	H
ATOM	620	N	GLY	A	246	8.961	-6.196	-4.924	1.00	0.00	N
ATOM	621	CA	GLY	A	246	7.739	-6.031	-5.715	1.00	0.00	C
ATOM	622	C	GLY	A	246	7.892	-4.879	-6.673	1.00	0.00	C
ATOM	623	O	GLY	A	246	7.523	-4.912	-7.850	1.00	0.00	O
ATOM	624	H1	GLY	A	246	9.864	-6.467	-5.286	1.00	0.00	H
ATOM	625	H2	GLY	A	246	8.753	-5.996	-3.969	1.00	0.00	H
ATOM	626	HA2	GLY	A	246	6.934	-5.774	-5.039	1.00	0.00	H
ATOM	627	HA3	GLY	A	246	7.450	-6.914	-6.255	1.00	0.00	H
ATOM	628	N	TYR	A	247	8.532	-3.790	-6.245	1.00	0.00	N

ATOM	629	CA	TYR	A	247	8.997	-2.692	-7.058	1.00	0.00	C
ATOM	630	CB	TYR	A	247	7.716	-1.820	-7.457	1.00	0.00	C
ATOM	631	CG	TYR	A	247	7.177	-1.169	-6.223	1.00	0.00	C
ATOM	632	CD1	TYR	A	247	7.926	-0.271	-5.453	1.00	0.00	C
ATOM	633	CE1	TYR	A	247	7.344	0.592	-4.469	1.00	0.00	C
ATOM	634	CZ	TYR	A	247	6.035	0.418	-4.180	1.00	0.00	C
ATOM	635	OH	TYR	A	247	5.521	1.084	-3.065	1.00	0.00	O
ATOM	636	CD2	TYR	A	247	5.814	-1.359	-5.865	1.00	0.00	C
ATOM	637	CE2	TYR	A	247	5.275	-0.584	-4.796	1.00	0.00	C
ATOM	638	C	TYR	A	247	9.989	-3.070	-8.182	1.00	0.00	C
ATOM	639	O	TYR	A	247	9.932	-2.528	-9.276	1.00	0.00	O
ATOM	640	H	TYR	A	247	8.707	-3.789	-5.256	1.00	0.00	H
ATOM	641	HA	TYR	A	247	9.645	-2.106	-6.430	1.00	0.00	H
ATOM	642	HB2	TYR	A	247	8.056	-0.950	-8.056	1.00	0.00	H
ATOM	643	HB3	TYR	A	247	6.920	-2.425	-7.920	1.00	0.00	H
ATOM	644	HD1	TYR	A	247	8.946	-0.019	-5.718	1.00	0.00	H
ATOM	645	HD2	TYR	A	247	5.156	-1.934	-6.494	1.00	0.00	H
ATOM	646	HE1	TYR	A	247	7.982	1.322	-3.988	1.00	0.00	H
ATOM	647	HE2	TYR	A	247	4.214	-0.721	-4.668	1.00	0.00	H
ATOM	648	HH	TYR	A	247	4.579	0.917	-3.141	1.00	0.00	H
ATOM	649	N	GLY	A	248	11.004	-3.938	-7.874	1.00	0.00	N
ATOM	650	CA	GLY	A	248	12.145	-4.311	-8.741	1.00	0.00	C
ATOM	651	C	GLY	A	248	11.723	-5.037	-10.025	1.00	0.00	C
ATOM	652	O	GLY	A	248	11.702	-6.278	-10.039	1.00	0.00	O
ATOM	653	H	GLY	A	248	10.970	-4.368	-6.961	1.00	0.00	H
ATOM	654	HA2	GLY	A	248	12.634	-3.386	-9.047	1.00	0.00	H
ATOM	655	HA3	GLY	A	248	12.820	-4.961	-8.170	1.00	0.00	H
ATOM	656	N	GLY	A	249	11.523	-4.303	-11.173	1.00	0.00	N
ATOM	657	CA	GLY	A	249	11.126	-4.798	-12.414	1.00	0.00	C

ATOM	658	C	GLY	A	249	9.719	-4.359	-12.713	1.00	0.00	C
ATOM	659	O	GLY	A	249	9.198	-4.664	-13.776	1.00	0.00	O
ATOM	660	H	GLY	A	249	11.680	-3.307	-11.109	1.00	0.00	H
ATOM	661	HA2	GLY	A	249	11.810	-4.410	-13.169	1.00	0.00	H
ATOM	662	HA3	GLY	A	249	11.159	-5.894	-12.421	1.00	0.00	H
ATOM	663	N	SER	A	250	9.082	-3.510	-11.871	1.00	0.00	N
ATOM	664	CA	SER	A	250	7.725	-3.012	-12.140	1.00	0.00	C
ATOM	665	CB	SER	A	250	6.858	-3.577	-10.985	1.00	0.00	C
ATOM	666	OG	SER	A	250	5.474	-3.248	-10.988	1.00	0.00	O
ATOM	667	C	SER	A	250	7.808	-1.506	-12.223	1.00	0.00	C
ATOM	668	O	SER	A	250	7.356	-0.929	-13.206	1.00	0.00	O
ATOM	669	H	SER	A	250	9.591	-3.303	-11.035	1.00	0.00	H
ATOM	670	HA	SER	A	250	7.360	-3.328	-13.106	1.00	0.00	H
ATOM	671	HB2	SER	A	250	7.227	-3.359	-9.945	1.00	0.00	H
ATOM	672	HB3	SER	A	250	6.874	-4.684	-10.872	1.00	0.00	H
ATOM	673	HG	SER	A	250	5.346	-2.324	-10.728	1.00	0.00	H
ATOM	674	N	GLY	A	251	8.375	-0.817	-11.292	1.00	0.00	N
ATOM	675	CA	GLY	A	251	8.228	0.662	-11.194	1.00	0.00	C
ATOM	676	C	GLY	A	251	9.464	1.378	-11.768	1.00	0.00	C
ATOM	677	O	GLY	A	251	9.440	2.527	-12.133	1.00	0.00	O
ATOM	678	H	GLY	A	251	8.939	-1.297	-10.605	1.00	0.00	H
ATOM	679	HA2	GLY	A	251	7.354	0.968	-11.769	1.00	0.00	H
ATOM	680	HA3	GLY	A	251	8.113	0.932	-10.148	1.00	0.00	H
ATOM	681	HC	GLY	A	251	10.391	0.829	-11.847	1.00	0.00	H
TER	682		GLY	A	251						
HETATM	683	C1	ARI	B	274	1.754	-2.298	-1.190	1.00	0.00	C
HETATM	684	C2	ARI	B	274	2.642	-2.380	-2.490	1.00	0.00	C
HETATM	685	O1	ARI	B	274	1.955	-1.810	-3.594	1.00	0.00	O
HETATM	686	C3	ARI	B	274	1.396	-2.641	-4.559	1.00	0.00	C

HETATM	687	C4	ARI	B	274	-0.067	-5.120	-7.745	1.00	0.00	C
HETATM	688	C5	ARI	B	274	0.318	-4.148	-6.613	1.00	0.00	C
HETATM	689	C6	ARI	B	274	-0.248	-4.311	-9.036	1.00	0.00	C
HETATM	690	C7	ARI	B	274	1.012	-3.018	-6.935	1.00	0.00	C
HETATM	691	C8	ARI	B	274	0.030	-4.554	-5.348	1.00	0.00	C
HETATM	692	C9	ARI	B	274	0.696	-3.166	-9.328	1.00	0.00	C
HETATM	693	N1	ARI	B	274	1.303	-2.552	-8.222	1.00	0.00	N
HETATM	694	C10	ARI	B	274	1.536	-2.233	-5.880	1.00	0.00	C
HETATM	695	C11	ARI	B	274	0.506	-3.708	-4.297	1.00	0.00	C
HETATM	696	O2	ARI	B	274	0.916	-2.773	-10.445	1.00	0.00	O
HETATM	697	C12	ARI	B	274	1.684	-0.872	-0.586	1.00	0.00	C
HETATM	698	C13	ARI	B	274	1.406	-0.911	0.896	1.00	0.00	C
HETATM	699	N2	ARI	B	274	1.356	0.473	1.472	1.00	0.00	N1+
HETATM	700	C14	ARI	B	274	0.573	0.661	2.743	1.00	0.00	C
HETATM	701	C15	ARI	B	274	2.806	1.090	1.653	1.00	0.00	C
HETATM	702	C16	ARI	B	274	0.449	2.082	3.195	1.00	0.00	C
HETATM	703	C17	ARI	B	274	2.672	2.553	2.143	1.00	0.00	C
HETATM	704	N3	ARI	B	274	1.872	2.522	3.421	1.00	0.00	N
HETATM	705	C18	ARI	B	274	2.017	3.699	4.235	1.00	0.00	C
HETATM	706	C19	ARI	B	274	0.913	4.531	4.535	1.00	0.00	C
HETATM	707	C20	ARI	B	274	3.250	3.821	4.935	1.00	0.00	C
HETATM	708	C21	ARI	B	274	1.075	5.659	5.381	1.00	0.00	C
HETATM	709	C22	ARI	B	274	3.384	4.927	5.721	1.00	0.00	C
HETATM	710	CL1	ARI	B	274	4.517	2.660	4.804	1.00	0.00	CL
HETATM	711	C23	ARI	B	274	2.309	5.863	5.889	1.00	0.00	C
HETATM	712	CL2	ARI	B	274	4.798	5.286	6.771	1.00	0.00	CL
HETATM	713	H	ARI	B	274	2.003	-1.921	-8.516	1.00	0.00	H
HETATM	714	HC2	ARI	B	274	2.194	-2.994	-0.454	1.00	0.00	H
HETATM	715	HC3	ARI	B	274	0.748	-2.736	-1.328	1.00	0.00	H

HETATM	716	HC4	ARI	B	274	2.934	-3.426	-2.679	1.00	0.00	H
HETATM	717	HC5	ARI	B	274	3.506	-1.732	-2.186	1.00	0.00	H
HETATM	718	HC6	ARI	B	274	-0.987	-5.652	-7.405	1.00	0.00	H
HETATM	719	HC7	ARI	B	274	0.801	-5.808	-7.841	1.00	0.00	H
HETATM	720	HC8	ARI	B	274	-1.340	-4.064	-9.123	1.00	0.00	H
HETATM	721	HC9	ARI	B	274	-0.081	-5.056	-9.842	1.00	0.00	H
HETATM	722	HC	ARI	B	274	-0.505	-5.461	-5.097	1.00	0.00	H
HETATM	723	HC1	ARI	B	274	2.076	-1.370	-6.250	1.00	0.00	H
HETATM	724	HC10	ARI	B	274	0.146	-3.862	-3.286	1.00	0.00	H
HETATM	725	HC11	ARI	B	274	0.964	-0.201	-1.104	1.00	0.00	H
HETATM	726	HC12	ARI	B	274	2.685	-0.447	-0.778	1.00	0.00	H
HETATM	727	HC13	ARI	B	274	2.242	-1.437	1.373	1.00	0.00	H
HETATM	728	HC14	ARI	B	274	0.395	-1.244	1.154	1.00	0.00	H
HETATM	729	HN	ARI	B	274	0.851	1.114	0.835	1.00	0.00	H
HETATM	730	HC15	ARI	B	274	1.114	0.135	3.538	1.00	0.00	H
HETATM	731	HC16	ARI	B	274	-0.386	0.236	2.437	1.00	0.00	H
HETATM	732	HC17	ARI	B	274	3.280	1.041	0.664	1.00	0.00	H
HETATM	733	HC18	ARI	B	274	3.287	0.402	2.361	1.00	0.00	H
HETATM	734	HC19	ARI	B	274	-0.152	2.123	4.129	1.00	0.00	H
HETATM	735	HC20	ARI	B	274	-0.041	2.709	2.429	1.00	0.00	H
HETATM	736	HC21	ARI	B	274	2.027	3.147	1.451	1.00	0.00	H
HETATM	737	HC22	ARI	B	274	3.637	3.064	2.336	1.00	0.00	H
HETATM	738	HC23	ARI	B	274	0.117	4.596	3.797	1.00	0.00	H
HETATM	739	HC24	ARI	B	274	0.237	6.336	5.469	1.00	0.00	H
HETATM	740	HC25	ARI	B	274	2.436	6.772	6.471	1.00	0.00	H
HETATM	741	OH2	TIP	X2357		4.254	0.486	7.850	1.00	0.00	O
HETATM	742	H1	TIP	X2357		4.764	0.178	8.595	1.00	0.00	H
HETATM	743	H2	TIP	X2357		3.539	1.000	8.240	1.00	0.00	H
HETATM	744	OH2	TIP	X3716		6.096	0.288	2.169	1.00	0.00	O

```
HETATM  745  H1  TIP X3716       6.243   0.567   3.076  1.00  0.00           H
HETATM  746  H2  TIP X3716       6.636   0.885   1.649  1.00  0.00           H
HETATM  747  OH2 TIP X5907       2.055  -1.939 -12.810  1.00  0.00           O
HETATM  748  H1  TIP X5907       1.224  -1.973 -13.276  1.00  0.00           H
HETATM  749  H2  TIP X5907       1.857  -2.283 -11.944  1.00  0.00           H
HETATM  750  OH2 TIP X8486       0.979  -2.430   6.403  1.00  0.00           O
HETATM  751  H1  TIP X8486       1.636  -1.946   5.893  1.00  0.00           H
HETATM  752  H2  TIP X8486       1.310  -2.416   7.299  1.00  0.00           H
HETATM  753  OH2 TIP X8539       3.109  -0.660   5.499  1.00  0.00           O
HETATM  754  H1  TIP X8539       3.036  -0.022   4.800  1.00  0.00           H
HETATM  755  H2  TIP X8539       3.428  -0.171   6.246  1.00  0.00           H
HETATM  756  OH2 TIP X9909       6.522   3.015   8.094  1.00  0.00           O
HETATM  757  H1  TIP X9909       5.591   3.002   7.864  1.00  0.00           H
HETATM  758  H2  TIP X9909       6.533   3.149   9.041  1.00  0.00           H
HETATM  759  OH2 TIP X 465       3.684  -5.256  -9.739  1.00  0.00           O
HETATM  760  H1  TIP X 465       3.998  -4.382  -9.978  1.00  0.00           H
HETATM  761  H2  TIP X 465       4.463  -5.760  -9.535  1.00  0.00           H
HETATM  762  OH2 TIP X5304       7.028   0.545   4.984  1.00  0.00           O
HETATM  763  H1  TIP X5304       7.923   0.298   5.203  1.00  0.00           H
HETATM  764  H2  TIP X5304       6.506   0.247   5.744  1.00  0.00           H
HETATM  765  OH2 TIP X6060       7.331   2.309   0.604  1.00  0.00           O
HETATM  766  H1  TIP X6060       6.694   2.390  -0.108  1.00  0.00           H
HETATM  767  H2  TIP X6060       7.429   3.199   0.935  1.00  0.00           H
HETATM  768  OH2 TIP X8569       3.989  -0.005 -12.387  1.00  0.00           O
HETATM  769  H1  TIP X8569       4.506  -0.019 -13.195  1.00  0.00           H
HETATM  770  H2  TIP X8569       3.279  -0.622 -12.545  1.00  0.00           H
HETATM  771  OH2 TIP X9931      -1.139  -3.612   0.946  1.00  0.00           O
HETATM  772  H1  TIP X9931      -1.969  -4.085   1.077  1.00  0.00           H
HETATM  773  H2  TIP X9931      -0.747  -3.581   1.813  1.00  0.00           H
```

HETATM	774	OH2	TIP	X2303	5.331	-2.521	3.092	1.00	0.00	O
HETATM	775	H1	TIP	X2303	5.353	-3.050	2.302	1.00	0.00	H
HETATM	776	H2	TIP	X2303	5.823	-1.732	2.854	1.00	0.00	H
HETATM	777	OH2	TIP	X2691	5.738	4.426	1.175	1.00	0.00	O
HETATM	778	H1	TIP	X2691	5.522	5.344	0.931	1.00	0.00	H
HETATM	779	H2	TIP	X2691	5.220	3.897	0.572	1.00	0.00	H
HETATM	780	OH2	TIP	X7607	8.823	4.507	7.669	1.00	0.00	O
HETATM	781	H1	TIP	X7607	9.145	4.592	8.571	1.00	0.00	H
HETATM	782	H2	TIP	X7607	7.866	4.447	7.759	1.00	0.00	H
HETATM	783	OH2	TIP	X8139	4.286	-3.162	-13.918	1.00	0.00	O
HETATM	784	H1	TIP	X8139	3.462	-2.924	-13.482	1.00	0.00	H
HETATM	785	H2	TIP	X8139	4.891	-3.333	-13.204	1.00	0.00	H
HETATM	786	OH2	TIP	X3101	4.741	-0.831	-9.759	1.00	0.00	O
HETATM	787	H1	TIP	X3101	4.177	-0.509	-9.054	1.00	0.00	H
HETATM	788	H2	TIP	X3101	4.397	-0.399	-10.541	1.00	0.00	H
HETATM	789	OH2	TIP	X8875	3.998	2.979	-0.840	1.00	0.00	O
HETATM	790	H1	TIP	X8875	4.532	2.317	-1.282	1.00	0.00	H
HETATM	791	H2	TIP	X8875	3.256	3.098	-1.408	1.00	0.00	H
HETATM	792	OH2	TIP	X2274	2.716	1.383	-5.754	1.00	0.00	O
HETATM	793	H1	TIP	X2274	2.084	2.078	-5.568	1.00	0.00	H
HETATM	794	H2	TIP	X2274	2.938	1.028	-4.890	1.00	0.00	H
HETATM	795	OH2	TIP	X2412	2.840	0.713	-2.899	1.00	0.00	O
HETATM	796	H1	TIP	X2412	2.213	1.278	-2.437	1.00	0.00	H
HETATM	797	H2	TIP	X2412	2.304	-0.024	-3.205	1.00	0.00	H
HETATM	798	OH2	TIP	X4044	-0.064	-3.495	3.593	1.00	0.00	O
HETATM	799	H1	TIP	X4044	-0.149	-4.453	3.605	1.00	0.00	H
HETATM	800	H2	TIP	X4044	0.437	-3.289	4.389	1.00	0.00	H
HETATM	801	OH2	TIP	X4593	2.939	0.367	-8.200	1.00	0.00	O
HETATM	802	H1	TIP	X4593	2.943	0.828	-7.356	1.00	0.00	H

```
HETATM  803  H2  TIP X4593       2.231   0.759  -8.697  1.00  0.00           H
HETATM  804  OH2 TIP X1519      -2.455   0.989   0.124  1.00  0.00           O
HETATM  805  H1  TIP X1519      -1.629   1.242  -0.290  1.00  0.00           H
HETATM  806  H2  TIP X1519      -3.077   1.658  -0.139  1.00  0.00           H
END
```

Aripiprazole (Cluster structure 2)

```
ATOM      1  N   GLY A  56       6.959  10.440   2.241  1.00  0.00           N
ATOM      2  CA  GLY A  56       6.868  10.332   3.681  1.00  0.00           C
ATOM      3  C   GLY A  56       7.900   9.461   4.409  1.00  0.00           C
ATOM      4  O   GLY A  56       8.501   9.921   5.317  1.00  0.00           O
ATOM      5  H1  GLY A  56       7.219  11.368   2.063  1.00  0.00           H
ATOM      6  H2  GLY A  56       6.168   9.979   1.892  1.00  0.00           H
ATOM      7  HA2 GLY A  56       7.080  11.345   4.024  1.00  0.00           H
ATOM      8  HA3 GLY A  56       5.847   9.951   3.949  1.00  0.00           H
ATOM      9  N   VAL A  57       8.226   8.233   3.959  1.00  0.00           N
ATOM     10  CA  VAL A  57       9.068   7.243   4.672  1.00  0.00           C
ATOM     11  CB  VAL A  57       8.985   5.851   4.108  1.00  0.00           C
ATOM     12  CG1 VAL A  57       9.824   4.767   4.918  1.00  0.00           C
ATOM     13  CG2 VAL A  57       7.459   5.434   4.247  1.00  0.00           C
ATOM     14  C   VAL A  57      10.514   7.672   4.542  1.00  0.00           C
ATOM     15  O   VAL A  57      11.220   7.685   5.533  1.00  0.00           O
ATOM     16  H   VAL A  57       7.855   7.837   3.120  1.00  0.00           H
ATOM     17  HA  VAL A  57       8.712   7.067   5.682  1.00  0.00           H
ATOM     18  HB  VAL A  57       9.189   5.732   3.026  1.00  0.00           H
ATOM     19 HG11 VAL A  57       9.770   4.946   6.018  1.00  0.00           H
ATOM     20 HG12 VAL A  57      10.898   4.925   4.731  1.00  0.00           H
ATOM     21 HG13 VAL A  57       9.640   3.715   4.619  1.00  0.00           H
ATOM     22 HG21 VAL A  57       7.153   5.301   5.305  1.00  0.00           H
```

ATOM	23	HG22	VAL	A	57	7.310	4.520	3.638	1.00	0.00	H
ATOM	24	HG23	VAL	A	57	6.782	6.209	3.781	1.00	0.00	H
ATOM	25	N	GLY	A	58	10.964	8.106	3.364	1.00	0.00	N
ATOM	26	CA	GLY	A	58	12.282	8.703	3.200	1.00	0.00	C
ATOM	27	C	GLY	A	58	12.589	9.874	4.054	1.00	0.00	C
ATOM	28	O	GLY	A	58	13.651	9.926	4.656	1.00	0.00	O
ATOM	29	H	GLY	A	58	10.367	8.017	2.554	1.00	0.00	H
ATOM	30	HA2	GLY	A	58	12.993	7.943	3.526	1.00	0.00	H
ATOM	31	HA3	GLY	A	58	12.398	8.988	2.123	1.00	0.00	H
ATOM	32	HC	GLY	A	58	11.876	10.680	4.144	1.00	0.00	H
ATOM	33	N	GLY	A	67	8.274	10.604	14.540	1.00	0.00	N
ATOM	34	CA	GLY	A	67	7.591	11.755	13.998	1.00	0.00	C
ATOM	35	C	GLY	A	67	6.795	11.356	12.782	1.00	0.00	C
ATOM	36	O	GLY	A	67	7.040	10.474	11.965	1.00	0.00	O
ATOM	37	H1	GLY	A	67	7.882	10.464	15.466	1.00	0.00	H
ATOM	38	H2	GLY	A	67	9.265	10.806	14.449	1.00	0.00	H
ATOM	39	HA2	GLY	A	67	6.916	12.157	14.753	1.00	0.00	H
ATOM	40	HA3	GLY	A	67	8.323	12.512	13.717	1.00	0.00	H
ATOM	41	N	PHE	A	68	5.663	12.025	12.598	1.00	0.00	N
ATOM	42	CA	PHE	A	68	4.663	11.578	11.674	1.00	0.00	C
ATOM	43	CB	PHE	A	68	4.096	10.163	12.007	1.00	0.00	C
ATOM	44	CG	PHE	A	68	3.238	9.651	10.888	1.00	0.00	C
ATOM	45	CD1	PHE	A	68	1.941	9.322	11.164	1.00	0.00	C
ATOM	46	CE1	PHE	A	68	1.179	8.671	10.217	1.00	0.00	C
ATOM	47	CZ	PHE	A	68	1.784	8.267	8.981	1.00	0.00	C
ATOM	48	CD2	PHE	A	68	3.893	9.339	9.676	1.00	0.00	C
ATOM	49	CE2	PHE	A	68	3.130	8.607	8.725	1.00	0.00	C
ATOM	50	C	PHE	A	68	3.543	12.634	11.564	1.00	0.00	C
ATOM	51	O	PHE	A	68	3.212	13.031	10.478	1.00	0.00	O

ATOM	52	H	PHE	A	68	5.369	12.697	13.269	1.00	0.00	H
ATOM	53	HA	PHE	A	68	5.097	11.625	10.684	1.00	0.00	H
ATOM	54	HB2	PHE	A	68	3.483	10.154	12.931	1.00	0.00	H
ATOM	55	HB3	PHE	A	68	4.917	9.458	12.208	1.00	0.00	H
ATOM	56	HD1	PHE	A	68	1.428	9.444	12.111	1.00	0.00	H
ATOM	57	HD2	PHE	A	68	4.917	9.663	9.577	1.00	0.00	H
ATOM	58	HE1	PHE	A	68	0.135	8.515	10.424	1.00	0.00	H
ATOM	59	HE2	PHE	A	68	3.543	8.473	7.737	1.00	0.00	H
ATOM	60	HZ	PHE	A	68	1.150	7.780	8.245	1.00	0.00	H
ATOM	61	N	GLY	A	69	2.949	13.001	12.712	1.00	0.00	N
ATOM	62	CA	GLY	A	69	1.862	13.884	13.006	1.00	0.00	C
ATOM	63	C	GLY	A	69	0.649	13.082	13.417	1.00	0.00	C
ATOM	64	O	GLY	A	69	-0.299	13.031	12.575	1.00	0.00	O
ATOM	65	H	GLY	A	69	3.350	12.567	13.531	1.00	0.00	H
ATOM	66	HA2	GLY	A	69	2.157	14.548	13.819	1.00	0.00	H
ATOM	67	HA3	GLY	A	69	1.615	14.474	12.122	1.00	0.00	H
ATOM	68	HC	GLY	A	69	0.592	12.581	14.372	1.00	0.00	H
ATOM	69	N	GLY	A	72	-4.457	11.562	12.933	1.00	0.00	N
ATOM	70	CA	GLY	A	72	-5.024	12.204	11.736	1.00	0.00	C
ATOM	71	C	GLY	A	72	-5.177	11.229	10.680	1.00	0.00	C
ATOM	72	O	GLY	A	72	-6.247	10.768	10.397	1.00	0.00	O
ATOM	73	H1	GLY	A	72	-5.171	11.645	13.651	1.00	0.00	H
ATOM	74	H2	GLY	A	72	-3.557	12.009	13.082	1.00	0.00	H
ATOM	75	HA2	GLY	A	72	-6.011	12.616	11.946	1.00	0.00	H
ATOM	76	HA3	GLY	A	72	-4.346	13.009	11.436	1.00	0.00	H
ATOM	77	N	CYS	A	73	-4.024	10.764	10.218	1.00	0.00	N
ATOM	78	CA	CYS	A	73	-3.942	9.570	9.361	1.00	0.00	C
ATOM	79	CB	CYS	A	73	-2.474	9.024	9.199	1.00	0.00	C
ATOM	80	SG	CYS	A	73	-2.149	7.281	8.555	1.00	0.00	S

ATOM	81	C	CYS	A	73	-4.632	9.592	8.016	1.00	0.00	C
ATOM	82	O	CYS	A	73	-4.876	10.647	7.447	1.00	0.00	O
ATOM	83	H	CYS	A	73	-3.115	11.084	10.458	1.00	0.00	H
ATOM	84	HA	CYS	A	73	-4.397	8.783	9.942	1.00	0.00	H
ATOM	85	HB2	CYS	A	73	-2.107	9.244	10.231	1.00	0.00	H
ATOM	86	HB3	CYS	A	73	-1.919	9.776	8.572	1.00	0.00	H
ATOM	87	HG	CYS	A	73	-0.832	7.061	8.511	1.00	0.00	H
ATOM	88	N	GLY	A	74	-5.016	8.339	7.548	1.00	0.00	N
ATOM	89	CA	GLY	A	74	-5.882	8.087	6.421	1.00	0.00	C
ATOM	90	C	GLY	A	74	-5.161	8.205	5.059	1.00	0.00	C
ATOM	91	O	GLY	A	74	-4.910	7.202	4.372	1.00	0.00	O
ATOM	92	H	GLY	A	74	-4.657	7.534	8.041	1.00	0.00	H
ATOM	93	HA2	GLY	A	74	-6.232	7.060	6.528	1.00	0.00	H
ATOM	94	HA3	GLY	A	74	-6.717	8.804	6.429	1.00	0.00	H
ATOM	95	N	GLY	A	75	-4.825	9.437	4.656	1.00	0.00	N
ATOM	96	CA	GLY	A	75	-4.343	9.716	3.289	1.00	0.00	C
ATOM	97	C	GLY	A	75	-3.057	8.896	3.054	1.00	0.00	C
ATOM	98	O	GLY	A	75	-2.916	8.288	1.986	1.00	0.00	O
ATOM	99	H	GLY	A	75	-4.904	10.202	5.310	1.00	0.00	H
ATOM	100	HA2	GLY	A	75	-5.106	9.448	2.558	1.00	0.00	H
ATOM	101	HA3	GLY	A	75	-4.114	10.763	3.198	1.00	0.00	H
ATOM	102	N	PHE	A	76	-2.081	8.792	3.978	1.00	0.00	N
ATOM	103	CA	PHE	A	76	-0.854	7.974	3.782	1.00	0.00	C
ATOM	104	CB	PHE	A	76	0.154	8.812	2.994	1.00	0.00	C
ATOM	105	CG	PHE	A	76	1.528	8.286	3.111	1.00	0.00	C
ATOM	106	CD1	PHE	A	76	2.177	7.597	2.096	1.00	0.00	C
ATOM	107	CE1	PHE	A	76	3.439	6.999	2.316	1.00	0.00	C
ATOM	108	CZ	PHE	A	76	4.042	7.184	3.594	1.00	0.00	C
ATOM	109	CD2	PHE	A	76	2.131	8.358	4.357	1.00	0.00	C

ATOM	110	CE2	PHE	A	76	3.393	7.835	4.632	1.00	0.00	C
ATOM	111	C	PHE	A	76	-1.079	6.483	3.222	1.00	0.00	C
ATOM	112	O	PHE	A	76	-0.528	6.066	2.217	1.00	0.00	O
ATOM	113	H	PHE	A	76	-2.310	9.177	4.860	1.00	0.00	H
ATOM	114	HA	PHE	A	76	-0.399	7.869	4.758	1.00	0.00	H
ATOM	115	HB2	PHE	A	76	0.076	9.826	3.433	1.00	0.00	H
ATOM	116	HB3	PHE	A	76	-0.039	8.917	1.901	1.00	0.00	H
ATOM	117	HD1	PHE	A	76	1.729	7.392	1.131	1.00	0.00	H
ATOM	118	HD2	PHE	A	76	1.542	8.752	5.173	1.00	0.00	H
ATOM	119	HE1	PHE	A	76	3.904	6.314	1.619	1.00	0.00	H
ATOM	120	HE2	PHE	A	76	3.849	7.869	5.609	1.00	0.00	H
ATOM	121	HZ	PHE	A	76	4.914	6.624	3.877	1.00	0.00	H
ATOM	122	N	VAL	A	77	-2.005	5.773	3.972	1.00	0.00	N
ATOM	123	CA	VAL	A	77	-2.621	4.471	3.624	1.00	0.00	C
ATOM	124	CB	VAL	A	77	-3.561	4.027	4.745	1.00	0.00	C
ATOM	125	CG1	VAL	A	77	-4.139	2.576	4.419	1.00	0.00	C
ATOM	126	CG2	VAL	A	77	-2.730	3.898	6.009	1.00	0.00	C
ATOM	127	C	VAL	A	77	-3.342	4.523	2.287	1.00	0.00	C
ATOM	128	O	VAL	A	77	-3.007	3.722	1.486	1.00	0.00	O
ATOM	129	H	VAL	A	77	-2.274	6.226	4.818	1.00	0.00	H
ATOM	130	HA	VAL	A	77	-1.836	3.745	3.526	1.00	0.00	H
ATOM	131	HB	VAL	A	77	-4.346	4.803	4.840	1.00	0.00	H
ATOM	132	HG11	VAL	A	77	-4.872	2.349	5.217	1.00	0.00	H
ATOM	133	HG12	VAL	A	77	-3.338	1.834	4.663	1.00	0.00	H
ATOM	134	HG13	VAL	A	77	-4.636	2.471	3.437	1.00	0.00	H
ATOM	135	HG21	VAL	A	77	-1.926	3.127	5.985	1.00	0.00	H
ATOM	136	HG22	VAL	A	77	-3.375	3.604	6.863	1.00	0.00	H
ATOM	137	HG23	VAL	A	77	-2.307	4.868	6.307	1.00	0.00	H
ATOM	138	N	GLY	A	78	-4.160	5.537	2.054	1.00	0.00	N

ATOM	139	CA	GLY	A	78	-4.871	5.768	0.834	1.00	0.00	C
ATOM	140	C	GLY	A	78	-3.923	5.795	-0.368	1.00	0.00	C
ATOM	141	O	GLY	A	78	-4.194	5.147	-1.348	1.00	0.00	O
ATOM	142	H	GLY	A	78	-4.294	6.204	2.800	1.00	0.00	H
ATOM	143	HA2	GLY	A	78	-5.582	4.951	0.709	1.00	0.00	H
ATOM	144	HA3	GLY	A	78	-5.395	6.729	0.883	1.00	0.00	H
ATOM	145	N	GLY	A	79	-2.768	6.458	-0.338	1.00	0.00	N
ATOM	146	CA	GLY	A	79	-1.634	6.369	-1.309	1.00	0.00	C
ATOM	147	C	GLY	A	79	-1.115	4.975	-1.384	1.00	0.00	C
ATOM	148	O	GLY	A	79	-1.094	4.407	-2.476	1.00	0.00	O
ATOM	149	H	GLY	A	79	-2.643	7.098	0.433	1.00	0.00	H
ATOM	150	HA2	GLY	A	79	-1.972	6.646	-2.307	1.00	0.00	H
ATOM	151	HA3	GLY	A	79	-0.852	7.049	-0.972	1.00	0.00	H
ATOM	152	N	ASP	A	80	-0.816	4.403	-0.216	1.00	0.00	N
ATOM	153	CA	ASP	A	80	-0.197	3.125	-0.207	1.00	0.00	C
ATOM	154	CB	ASP	A	80	1.287	3.185	-0.442	1.00	0.00	C
ATOM	155	CG	ASP	A	80	2.013	1.758	-0.251	1.00	0.00	C
ATOM	156	OD1	ASP	A	80	3.090	1.677	-0.832	1.00	0.00	O
ATOM	157	OD2	ASP	A	80	1.504	0.905	0.525	1.00	0.00	O1-
ATOM	158	C	ASP	A	80	-1.073	2.069	-1.087	1.00	0.00	C
ATOM	159	O	ASP	A	80	-0.645	1.457	-2.031	1.00	0.00	O
ATOM	160	H	ASP	A	80	-0.996	4.790	0.686	1.00	0.00	H
ATOM	161	HA	ASP	A	80	-0.414	2.817	0.794	1.00	0.00	H
ATOM	162	HB2	ASP	A	80	1.681	3.859	0.348	1.00	0.00	H
ATOM	163	HB3	ASP	A	80	1.455	3.551	-1.488	1.00	0.00	H
ATOM	164	N	VAL	A	81	-2.359	1.921	-0.784	1.00	0.00	N
ATOM	165	CA	VAL	A	81	-3.215	1.051	-1.576	1.00	0.00	C
ATOM	166	CB	VAL	A	81	-4.518	0.806	-0.815	1.00	0.00	C
ATOM	167	CG1	VAL	A	81	-5.495	-0.079	-1.558	1.00	0.00	C

ATOM	168	CG2	VAL	A	81	-4.062	0.110	0.454	1.00	0.00	C
ATOM	169	C	VAL	A	81	-3.561	1.537	-2.979	1.00	0.00	C
ATOM	170	O	VAL	A	81	-3.663	0.768	-3.939	1.00	0.00	O
ATOM	171	H	VAL	A	81	-2.845	2.417	-0.084	1.00	0.00	H
ATOM	172	HA	VAL	A	81	-2.784	0.057	-1.540	1.00	0.00	H
ATOM	173	HB	VAL	A	81	-4.944	1.801	-0.560	1.00	0.00	H
ATOM	174	HG11	VAL	A	81	-5.101	-1.115	-1.690	1.00	0.00	H
ATOM	175	HG12	VAL	A	81	-5.707	0.168	-2.631	1.00	0.00	H
ATOM	176	HG13	VAL	A	81	-6.454	-0.232	-1.009	1.00	0.00	H
ATOM	177	HG21	VAL	A	81	-4.953	-0.088	1.087	1.00	0.00	H
ATOM	178	HG22	VAL	A	81	-3.506	0.784	1.136	1.00	0.00	H
ATOM	179	HG23	VAL	A	81	-3.499	-0.841	0.409	1.00	0.00	H
ATOM	180	N	GLY	A	82	-3.747	2.870	-3.156	1.00	0.00	N
ATOM	181	CA	GLY	A	82	-3.994	3.463	-4.435	1.00	0.00	C
ATOM	182	C	GLY	A	82	-2.850	3.196	-5.513	1.00	0.00	C
ATOM	183	O	GLY	A	82	-3.128	2.770	-6.619	1.00	0.00	O
ATOM	184	H	GLY	A	82	-3.709	3.472	-2.346	1.00	0.00	H
ATOM	185	HA2	GLY	A	82	-4.914	2.992	-4.781	1.00	0.00	H
ATOM	186	HA3	GLY	A	82	-4.092	4.556	-4.342	1.00	0.00	H
ATOM	187	N	GLY	A	83	-1.576	3.264	-5.099	1.00	0.00	N
ATOM	188	CA	GLY	A	83	-0.299	2.883	-5.776	1.00	0.00	C
ATOM	189	C	GLY	A	83	-0.304	1.518	-6.231	1.00	0.00	C
ATOM	190	O	GLY	A	83	-0.229	1.182	-7.419	1.00	0.00	O
ATOM	191	H	GLY	A	83	-1.459	3.638	-4.168	1.00	0.00	H
ATOM	192	HA2	GLY	A	83	-0.161	3.484	-6.675	1.00	0.00	H
ATOM	193	HA3	GLY	A	83	0.513	3.064	-5.051	1.00	0.00	H
ATOM	194	N	CYS	A	84	-0.425	0.616	-5.247	1.00	0.00	N
ATOM	195	CA	CYS	A	84	-0.664	-0.823	-5.489	1.00	0.00	C
ATOM	196	CB	CYS	A	84	-0.669	-1.570	-4.143	1.00	0.00	C

ATOM	197	SG	CYS	A	84	0.969	-2.003	-3.538	1.00	0.00	S
ATOM	198	C	CYS	A	84	-1.825	-1.263	-6.346	1.00	0.00	C
ATOM	199	O	CYS	A	84	-1.651	-2.134	-7.154	1.00	0.00	O
ATOM	200	H	CYS	A	84	-0.277	0.872	-4.300	1.00	0.00	H
ATOM	201	HA	CYS	A	84	0.162	-1.212	-6.072	1.00	0.00	H
ATOM	202	HB2	CYS	A	84	-1.155	-2.569	-4.309	1.00	0.00	H
ATOM	203	HB3	CYS	A	84	-1.266	-1.010	-3.389	1.00	0.00	H
ATOM	204	HG	CYS	A	84	1.297	-0.734	-3.403	1.00	0.00	H
ATOM	205	N	THR	A	85	-2.981	-0.593	-6.229	1.00	0.00	N
ATOM	206	CA	THR	A	85	-4.181	-0.770	-7.055	1.00	0.00	C
ATOM	207	CB	THR	A	85	-5.434	-0.119	-6.517	1.00	0.00	C
ATOM	208	OG1	THR	A	85	-5.556	-0.536	-5.196	1.00	0.00	O
ATOM	209	CG2	THR	A	85	-6.737	-0.458	-7.223	1.00	0.00	C
ATOM	210	C	THR	A	85	-4.036	-0.314	-8.519	1.00	0.00	C
ATOM	211	O	THR	A	85	-4.488	-0.956	-9.442	1.00	0.00	O
ATOM	212	H	THR	A	85	-3.116	0.090	-5.527	1.00	0.00	H
ATOM	213	HA	THR	A	85	-4.330	-1.841	-7.052	1.00	0.00	H
ATOM	214	HB	THR	A	85	-5.226	0.967	-6.441	1.00	0.00	H
ATOM	215	HG21	THR	A	85	-6.758	0.232	-8.104	1.00	0.00	H
ATOM	216	HG22	THR	A	85	-7.565	-0.220	-6.519	1.00	0.00	H
ATOM	217	HG23	THR	A	85	-6.799	-1.498	-7.621	1.00	0.00	H
ATOM	218	HG1	THR	A	85	-4.789	-0.131	-4.753	1.00	0.00	H
ATOM	219	N	GLY	A	86	-3.454	0.892	-8.687	1.00	0.00	N
ATOM	220	CA	GLY	A	86	-3.131	1.451	-9.955	1.00	0.00	C
ATOM	221	C	GLY	A	86	-2.045	0.702	-10.769	1.00	0.00	C
ATOM	222	O	GLY	A	86	-2.181	0.388	-11.962	1.00	0.00	O
ATOM	223	H	GLY	A	86	-3.234	1.431	-7.862	1.00	0.00	H
ATOM	224	HA2	GLY	A	86	-4.058	1.371	-10.523	1.00	0.00	H
ATOM	225	HA3	GLY	A	86	-2.785	2.502	-9.827	1.00	0.00	H

ATOM	226	N	GLY	A	87	-1.000	0.247	-9.981	1.00	0.00	N
ATOM	227	CA	GLY	A	87	0.010	-0.670	-10.483	1.00	0.00	C
ATOM	228	C	GLY	A	87	-0.535	-2.031	-10.946	1.00	0.00	C
ATOM	229	O	GLY	A	87	-0.213	-2.523	-12.040	1.00	0.00	O
ATOM	230	H	GLY	A	87	-0.938	0.564	-9.024	1.00	0.00	H
ATOM	231	HA2	GLY	A	87	0.438	-0.189	-11.363	1.00	0.00	H
ATOM	232	HA3	GLY	A	87	0.762	-0.848	-9.692	1.00	0.00	H
ATOM	233	N	ILE	A	88	-1.443	-2.673	-10.197	1.00	0.00	N
ATOM	234	CA	ILE	A	88	-2.205	-3.840	-10.686	1.00	0.00	C
ATOM	235	C	ILE	A	88	-3.140	-3.520	-11.871	1.00	0.00	C
ATOM	236	O	ILE	A	88	-3.104	-4.277	-12.845	1.00	0.00	O
ATOM	237	CB	ILE	A	88	-2.909	-4.565	-9.532	1.00	0.00	C
ATOM	238	CG2	ILE	A	88	-3.828	-3.639	-8.813	1.00	0.00	C
ATOM	239	CG1	ILE	A	88	-3.809	-5.717	-9.907	1.00	0.00	C
ATOM	240	CD1	ILE	A	88	-3.180	-6.882	-10.604	1.00	0.00	C
ATOM	241	H	ILE	A	88	-1.669	-2.331	-9.267	1.00	0.00	H
ATOM	242	HA	ILE	A	88	-1.469	-4.545	-11.078	1.00	0.00	H
ATOM	243	HB	ILE	A	88	-2.172	-4.922	-8.821	1.00	0.00	H
ATOM	244	HG12	ILE	A	88	-4.221	-6.072	-8.972	1.00	0.00	H
ATOM	245	HG13	ILE	A	88	-4.651	-5.376	-10.509	1.00	0.00	H
ATOM	246	HG21	ILE	A	88	-4.678	-3.335	-9.411	1.00	0.00	H
ATOM	247	HG22	ILE	A	88	-4.195	-4.057	-7.895	1.00	0.00	H
ATOM	248	HG23	ILE	A	88	-3.272	-2.777	-8.556	1.00	0.00	H
ATOM	249	HD11	ILE	A	88	-2.506	-7.470	-9.999	1.00	0.00	H
ATOM	250	HD12	ILE	A	88	-4.034	-7.519	-10.769	1.00	0.00	H
ATOM	251	HD13	ILE	A	88	-2.711	-6.621	-11.553	1.00	0.00	H
ATOM	252	N	GLY	A	89	-3.956	-2.433	-11.837	1.00	0.00	N
ATOM	253	CA	GLY	A	89	-4.645	-1.970	-13.032	1.00	0.00	C
ATOM	254	C	GLY	A	89	-3.746	-1.890	-14.329	1.00	0.00	C

ATOM	255	O	GLY	A	89	-4.204	-2.321	-15.388	1.00	0.00	O
ATOM	256	H	GLY	A	89	-4.086	-1.938	-10.966	1.00	0.00	H
ATOM	257	HA2	GLY	A	89	-5.417	-2.717	-13.217	1.00	0.00	H
ATOM	258	HA3	GLY	A	89	-5.058	-0.963	-12.856	1.00	0.00	H
ATOM	259	HC	GLY	A	89	-2.753	-1.467	-14.288	1.00	0.00	H
ATOM	260	N	GLY	A	149	-4.158	3.274	11.748	1.00	0.00	N
ATOM	261	CA	GLY	A	149	-4.372	1.944	11.198	1.00	0.00	C
ATOM	262	C	GLY	A	149	-3.534	1.826	9.914	1.00	0.00	C
ATOM	263	O	GLY	A	149	-4.107	1.994	8.812	1.00	0.00	O
ATOM	264	H1	GLY	A	149	-3.725	3.114	12.656	1.00	0.00	H
ATOM	265	H2	GLY	A	149	-5.074	3.718	11.720	1.00	0.00	H
ATOM	266	HA2	GLY	A	149	-4.083	1.186	11.926	1.00	0.00	H
ATOM	267	HA3	GLY	A	149	-5.412	1.814	10.949	1.00	0.00	H
ATOM	268	N	ILE	A	150	-2.207	1.577	9.970	1.00	0.00	N
ATOM	269	CA	ILE	A	150	-1.558	1.487	8.642	1.00	0.00	C
ATOM	270	CB	ILE	A	150	-0.072	1.286	8.827	1.00	0.00	C
ATOM	271	CG2	ILE	A	150	0.646	1.093	7.477	1.00	0.00	C
ATOM	272	CG1	ILE	A	150	0.507	2.497	9.582	1.00	0.00	C
ATOM	273	CD1	ILE	A	150	0.267	3.913	9.027	1.00	0.00	C
ATOM	274	C	ILE	A	150	-2.016	0.256	7.761	1.00	0.00	C
ATOM	275	O	ILE	A	150	-2.127	0.391	6.536	1.00	0.00	O
ATOM	276	H	ILE	A	150	-1.680	1.318	10.778	1.00	0.00	H
ATOM	277	HA	ILE	A	150	-1.712	2.379	8.056	1.00	0.00	H
ATOM	278	HB	ILE	A	150	0.092	0.352	9.397	1.00	0.00	H
ATOM	279	HG12	ILE	A	150	1.563	2.217	9.737	1.00	0.00	H
ATOM	280	HG13	ILE	A	150	0.092	2.483	10.610	1.00	0.00	H
ATOM	281	HG21	ILE	A	150	0.314	0.131	7.010	1.00	0.00	H
ATOM	282	HG22	ILE	A	150	1.733	1.130	7.710	1.00	0.00	H
ATOM	283	HG23	ILE	A	150	0.353	1.887	6.756	1.00	0.00	H

ATOM	284	HD11	ILE	A	150	-0.810	4.199	8.962	1.00	0.00	H
ATOM	285	HD12	ILE	A	150	0.602	3.925	7.958	1.00	0.00	H
ATOM	286	HD13	ILE	A	150	0.724	4.715	9.649	1.00	0.00	H
ATOM	287	N	GLY	A	151	-2.345	-0.950	8.393	1.00	0.00	N
ATOM	288	CA	GLY	A	151	-2.554	-2.163	7.654	1.00	0.00	C
ATOM	289	C	GLY	A	151	-3.700	-2.142	6.664	1.00	0.00	C
ATOM	290	O	GLY	A	151	-3.478	-2.653	5.560	1.00	0.00	O
ATOM	291	H	GLY	A	151	-2.438	-0.967	9.399	1.00	0.00	H
ATOM	292	HA2	GLY	A	151	-1.651	-2.291	7.058	1.00	0.00	H
ATOM	293	HA3	GLY	A	151	-2.724	-3.000	8.371	1.00	0.00	H
ATOM	294	HC	GLY	A	151	-4.657	-1.705	6.908	1.00	0.00	H
ATOM	295	N	GLY	A	155	-10.034	-3.294	3.429	1.00	0.00	N
ATOM	296	CA	GLY	A	155	-9.730	-2.477	2.253	1.00	0.00	C
ATOM	297	C	GLY	A	155	-8.683	-3.194	1.336	1.00	0.00	C
ATOM	298	O	GLY	A	155	-8.946	-3.289	0.095	1.00	0.00	O
ATOM	299	H1	GLY	A	155	-11.001	-3.529	3.326	1.00	0.00	H
ATOM	300	H2	GLY	A	155	-9.701	-2.752	4.202	1.00	0.00	H
ATOM	301	HA2	GLY	A	155	-10.657	-2.324	1.700	1.00	0.00	H
ATOM	302	HA3	GLY	A	155	-9.311	-1.526	2.565	1.00	0.00	H
ATOM	303	N	VAL	A	156	-7.513	-3.672	1.817	1.00	0.00	N
ATOM	304	CA	VAL	A	156	-6.589	-4.497	1.046	1.00	0.00	C
ATOM	305	CB	VAL	A	156	-5.426	-4.850	2.019	1.00	0.00	C
ATOM	306	CG1	VAL	A	156	-4.515	-5.929	1.336	1.00	0.00	C
ATOM	307	CG2	VAL	A	156	-4.659	-3.576	2.270	1.00	0.00	C
ATOM	308	C	VAL	A	156	-7.161	-5.840	0.401	1.00	0.00	C
ATOM	309	O	VAL	A	156	-6.866	-6.085	-0.761	1.00	0.00	O
ATOM	310	H	VAL	A	156	-7.365	-3.425	2.774	1.00	0.00	H
ATOM	311	HA	VAL	A	156	-6.232	-3.876	0.235	1.00	0.00	H
ATOM	312	HB	VAL	A	156	-5.903	-5.293	2.916	1.00	0.00	H

ATOM	313	HG11	VAL	A	156	-4.928	-6.910	1.019	1.00	0.00	H
ATOM	314	HG12	VAL	A	156	-3.728	-6.185	2.077	1.00	0.00	H
ATOM	315	HG13	VAL	A	156	-3.979	-5.411	0.505	1.00	0.00	H
ATOM	316	HG21	VAL	A	156	-3.983	-3.731	3.143	1.00	0.00	H
ATOM	317	HG22	VAL	A	156	-5.265	-2.683	2.480	1.00	0.00	H
ATOM	318	HG23	VAL	A	156	-3.896	-3.306	1.510	1.00	0.00	H
ATOM	319	N	GLY	A	157	-7.908	-6.615	1.107	1.00	0.00	N
ATOM	320	CA	GLY	A	157	-8.701	-7.711	0.672	1.00	0.00	C
ATOM	321	C	GLY	A	157	-9.762	-7.356	-0.400	1.00	0.00	C
ATOM	322	O	GLY	A	157	-9.841	-7.946	-1.522	1.00	0.00	O
ATOM	323	H	GLY	A	157	-7.934	-6.419	2.097	1.00	0.00	H
ATOM	324	HA2	GLY	A	157	-7.994	-8.404	0.217	1.00	0.00	H
ATOM	325	HA3	GLY	A	157	-9.235	-8.159	1.534	1.00	0.00	H
ATOM	326	N	GLY	A	158	-10.528	-6.298	-0.135	1.00	0.00	N
ATOM	327	CA	GLY	A	158	-11.407	-5.713	-1.201	1.00	0.00	C
ATOM	328	C	GLY	A	158	-10.745	-5.271	-2.516	1.00	0.00	C
ATOM	329	O	GLY	A	158	-11.064	-5.795	-3.596	1.00	0.00	O
ATOM	330	H	GLY	A	158	-10.517	-5.888	0.788	1.00	0.00	H
ATOM	331	HA2	GLY	A	158	-12.057	-6.533	-1.505	1.00	0.00	H
ATOM	332	HA3	GLY	A	158	-11.938	-4.838	-0.757	1.00	0.00	H
ATOM	333	N	SER	A	159	-9.759	-4.461	-2.410	1.00	0.00	N
ATOM	334	CA	SER	A	159	-8.881	-4.084	-3.497	1.00	0.00	C
ATOM	335	CB	SER	A	159	-7.817	-3.016	-3.137	1.00	0.00	C
ATOM	336	OG	SER	A	159	-6.965	-3.419	-2.064	1.00	0.00	O
ATOM	337	C	SER	A	159	-8.171	-5.229	-4.138	1.00	0.00	C
ATOM	338	O	SER	A	159	-8.104	-5.338	-5.383	1.00	0.00	O
ATOM	339	H	SER	A	159	-9.600	-4.107	-1.500	1.00	0.00	H
ATOM	340	HA	SER	A	159	-9.476	-3.591	-4.252	1.00	0.00	H
ATOM	341	HB2	SER	A	159	-8.405	-2.153	-2.775	1.00	0.00	H

ATOM	342	HB3	SER	A	159	-7.301	-2.665	-4.057	1.00	0.00	H
ATOM	343	HG	SER	A	159	-7.427	-3.296	-1.227	1.00	0.00	H
ATOM	344	N	SER	A	160	-7.720	-6.180	-3.343	1.00	0.00	N
ATOM	345	CA	SER	A	160	-7.162	-7.389	-3.921	1.00	0.00	C
ATOM	346	CB	SER	A	160	-6.696	-8.461	-2.856	1.00	0.00	C
ATOM	347	OG	SER	A	160	-5.490	-7.906	-2.227	1.00	0.00	O
ATOM	348	C	SER	A	160	-8.056	-8.278	-4.853	1.00	0.00	C
ATOM	349	O	SER	A	160	-7.633	-8.600	-5.947	1.00	0.00	O
ATOM	350	H	SER	A	160	-7.863	-6.069	-2.360	1.00	0.00	H
ATOM	351	HA	SER	A	160	-6.272	-7.217	-4.502	1.00	0.00	H
ATOM	352	HB2	SER	A	160	-6.548	-9.456	-3.305	1.00	0.00	H
ATOM	353	HB3	SER	A	160	-7.400	-8.737	-2.034	1.00	0.00	H
ATOM	354	HG	SER	A	160	-5.771	-7.135	-1.714	1.00	0.00	H
ATOM	355	N	GLY	A	161	-9.314	-8.498	-4.444	1.00	0.00	N
ATOM	356	CA	GLY	A	161	-10.348	-9.154	-5.214	1.00	0.00	C
ATOM	357	C	GLY	A	161	-10.695	-8.488	-6.469	1.00	0.00	C
ATOM	358	O	GLY	A	161	-10.835	-9.121	-7.499	1.00	0.00	O
ATOM	359	H	GLY	A	161	-9.561	-8.178	-3.518	1.00	0.00	H
ATOM	360	HA2	GLY	A	161	-9.955	-10.127	-5.510	1.00	0.00	H
ATOM	361	HA3	GLY	A	161	-11.254	-9.243	-4.570	1.00	0.00	H
ATOM	362	N	GLY	A	162	-10.835	-7.189	-6.402	1.00	0.00	N
ATOM	363	CA	GLY	A	162	-11.342	-6.497	-7.611	1.00	0.00	C
ATOM	364	C	GLY	A	162	-10.234	-5.950	-8.517	1.00	0.00	C
ATOM	365	O	GLY	A	162	-10.414	-5.277	-9.519	1.00	0.00	O
ATOM	366	H	GLY	A	162	-10.607	-6.678	-5.561	1.00	0.00	H
ATOM	367	HA2	GLY	A	162	-11.889	-7.235	-8.198	1.00	0.00	H
ATOM	368	HA3	GLY	A	162	-11.986	-5.665	-7.292	1.00	0.00	H
ATOM	369	N	SER	A	163	-8.962	-6.236	-8.138	1.00	0.00	N
ATOM	370	CA	SER	A	163	-7.806	-5.897	-9.008	1.00	0.00	C

ATOM	371	CB	SER	A	163	-7.196	-4.427	-8.833	1.00	0.00	C
ATOM	372	OG	SER	A	163	-6.483	-4.368	-7.624	1.00	0.00	O
ATOM	373	C	SER	A	163	-6.793	-6.998	-9.137	1.00	0.00	C
ATOM	374	O	SER	A	163	-6.511	-7.451	-10.254	1.00	0.00	O
ATOM	375	H	SER	A	163	-8.918	-6.479	-7.168	1.00	0.00	H
ATOM	376	HA	SER	A	163	-8.221	-5.843	-10.000	1.00	0.00	H
ATOM	377	HB2	SER	A	163	-7.939	-3.602	-8.892	1.00	0.00	H
ATOM	378	HB3	SER	A	163	-6.503	-4.324	-9.703	1.00	0.00	H
ATOM	379	HG	SER	A	163	-7.114	-4.451	-6.895	1.00	0.00	H
ATOM	380	N	PHE	A	164	-6.123	-7.417	-8.015	1.00	0.00	N
ATOM	381	CA	PHE	A	164	-5.042	-8.453	-7.927	1.00	0.00	C
ATOM	382	CB	PHE	A	164	-4.282	-8.476	-6.592	1.00	0.00	C
ATOM	383	CG	PHE	A	164	-3.161	-9.444	-6.518	1.00	0.00	C
ATOM	384	CD1	PHE	A	164	-3.114	-10.434	-5.484	1.00	0.00	C
ATOM	385	CE1	PHE	A	164	-2.094	-11.290	-5.269	1.00	0.00	C
ATOM	386	CZ	PHE	A	164	-0.960	-11.069	-6.102	1.00	0.00	C
ATOM	387	CD2	PHE	A	164	-1.988	-9.246	-7.247	1.00	0.00	C
ATOM	388	CE2	PHE	A	164	-0.907	-10.109	-7.121	1.00	0.00	C
ATOM	389	C	PHE	A	164	-5.477	-9.808	-8.429	1.00	0.00	C
ATOM	390	O	PHE	A	164	-4.741	-10.481	-9.203	1.00	0.00	O
ATOM	391	H	PHE	A	164	-6.211	-6.904	-7.161	1.00	0.00	H
ATOM	392	HA	PHE	A	164	-4.369	-8.236	-8.735	1.00	0.00	H
ATOM	393	HB2	PHE	A	164	-4.867	-8.778	-5.696	1.00	0.00	H
ATOM	394	HB3	PHE	A	164	-3.818	-7.488	-6.314	1.00	0.00	H
ATOM	395	HD1	PHE	A	164	-4.032	-10.475	-4.913	1.00	0.00	H
ATOM	396	HD2	PHE	A	164	-1.961	-8.498	-8.022	1.00	0.00	H
ATOM	397	HE1	PHE	A	164	-2.121	-12.047	-4.484	1.00	0.00	H
ATOM	398	HE2	PHE	A	164	-0.066	-9.820	-7.742	1.00	0.00	H
ATOM	399	HZ	PHE	A	164	-0.086	-11.714	-6.034	1.00	0.00	H

ATOM	400	N	GLY	A	165	-6.724	-10.207	-8.137	1.00	0.00	N
ATOM	401	CA	GLY	A	165	-7.388	-11.383	-8.554	1.00	0.00	C
ATOM	402	C	GLY	A	165	-7.588	-11.619	-10.084	1.00	0.00	C
ATOM	403	O	GLY	A	165	-7.652	-12.831	-10.501	1.00	0.00	O
ATOM	404	H	GLY	A	165	-7.257	-9.588	-7.543	1.00	0.00	H
ATOM	405	HA2	GLY	A	165	-6.698	-12.161	-8.228	1.00	0.00	H
ATOM	406	HA3	GLY	A	165	-8.409	-11.431	-8.084	1.00	0.00	H
ATOM	407	HC	GLY	A	165	-7.670	-10.789	-10.770	1.00	0.00	H
ATOM	408	N	GLY	A	212	6.674	-11.660	-12.190	1.00	0.00	N
ATOM	409	CA	GLY	A	212	7.218	-10.695	-11.229	1.00	0.00	C
ATOM	410	C	GLY	A	212	6.230	-10.078	-10.283	1.00	0.00	C
ATOM	411	O	GLY	A	212	6.552	-10.086	-9.085	1.00	0.00	O
ATOM	412	H1	GLY	A	212	7.062	-12.531	-11.934	1.00	0.00	H
ATOM	413	H2	GLY	A	212	6.778	-11.235	-13.075	1.00	0.00	H
ATOM	414	HA2	GLY	A	212	7.895	-11.261	-10.589	1.00	0.00	H
ATOM	415	HA3	GLY	A	212	7.728	-9.887	-11.803	1.00	0.00	H
ATOM	416	N	PHE	A	213	5.071	-9.635	-10.832	1.00	0.00	N
ATOM	417	CA	PHE	A	213	4.014	-9.087	-10.033	1.00	0.00	C
ATOM	418	CB	PHE	A	213	2.881	-8.546	-10.889	1.00	0.00	C
ATOM	419	CG	PHE	A	213	2.160	-7.449	-10.215	1.00	0.00	C
ATOM	420	CD1	PHE	A	213	2.748	-6.180	-10.066	1.00	0.00	C
ATOM	421	CE1	PHE	A	213	1.979	-5.090	-9.492	1.00	0.00	C
ATOM	422	CZ	PHE	A	213	0.611	-5.341	-9.112	1.00	0.00	C
ATOM	423	CD2	PHE	A	213	0.804	-7.626	-9.930	1.00	0.00	C
ATOM	424	CE2	PHE	A	213	0.075	-6.560	-9.404	1.00	0.00	C
ATOM	425	C	PHE	A	213	3.510	-10.112	-8.995	1.00	0.00	C
ATOM	426	O	PHE	A	213	3.205	-9.894	-7.801	1.00	0.00	O
ATOM	427	H	PHE	A	213	5.026	-9.564	-11.820	1.00	0.00	H
ATOM	428	HA	PHE	A	213	4.442	-8.278	-9.453	1.00	0.00	H

ATOM	429	HB2	PHE	A	213	2.219	-9.358	-11.272	1.00	0.00	H
ATOM	430	HB3	PHE	A	213	3.427	-8.049	-11.739	1.00	0.00	H
ATOM	431	HD1	PHE	A	213	3.745	-6.034	-10.423	1.00	0.00	H
ATOM	432	HD2	PHE	A	213	0.168	-8.489	-10.018	1.00	0.00	H
ATOM	433	HE1	PHE	A	213	2.366	-4.084	-9.397	1.00	0.00	H
ATOM	434	HE2	PHE	A	213	-0.893	-6.866	-9.045	1.00	0.00	H
ATOM	435	HZ	PHE	A	213	-0.080	-4.641	-8.660	1.00	0.00	H
ATOM	436	N	GLY	A	214	3.368	-11.403	-9.547	1.00	0.00	N
ATOM	437	CA	GLY	A	214	2.947	-12.634	-8.864	1.00	0.00	C
ATOM	438	C	GLY	A	214	3.817	-12.861	-7.563	1.00	0.00	C
ATOM	439	O	GLY	A	214	3.311	-12.826	-6.430	1.00	0.00	O
ATOM	440	H	GLY	A	214	3.582	-11.491	-10.530	1.00	0.00	H
ATOM	441	HA2	GLY	A	214	1.894	-12.533	-8.600	1.00	0.00	H
ATOM	442	HA3	GLY	A	214	3.093	-13.485	-9.514	1.00	0.00	H
ATOM	443	HC	GLY	A	214	4.876	-13.051	-7.663	1.00	0.00	H
ATOM	444	N	GLY	A	216	6.133	-10.885	-6.117	1.00	0.00	N
ATOM	445	CA	GLY	A	216	6.017	-9.822	-5.121	1.00	0.00	C
ATOM	446	C	GLY	A	216	4.822	-10.036	-4.200	1.00	0.00	C
ATOM	447	O	GLY	A	216	4.866	-9.831	-2.991	1.00	0.00	O
ATOM	448	H1	GLY	A	216	6.994	-11.350	-5.902	1.00	0.00	H
ATOM	449	H2	GLY	A	216	5.984	-10.433	-6.998	1.00	0.00	H
ATOM	450	HA2	GLY	A	216	6.920	-9.832	-4.511	1.00	0.00	H
ATOM	451	HA3	GLY	A	216	5.904	-8.862	-5.634	1.00	0.00	H
ATOM	452	N	TRP	A	217	3.679	-10.313	-4.868	1.00	0.00	N
ATOM	453	CA	TRP	A	217	2.370	-10.482	-4.302	1.00	0.00	C
ATOM	454	CB	TRP	A	217	1.622	-9.231	-3.730	1.00	0.00	C
ATOM	455	CG	TRP	A	217	1.864	-7.989	-4.556	1.00	0.00	C
ATOM	456	CD1	TRP	A	217	1.304	-7.689	-5.778	1.00	0.00	C
ATOM	457	NE1	TRP	A	217	1.760	-6.482	-6.228	1.00	0.00	N

ATOM	458	CE2	TRP	A	217	2.661	-5.932	-5.282	1.00	0.00	C
ATOM	459	CD2	TRP	A	217	2.728	-6.879	-4.211	1.00	0.00	C
ATOM	460	CE3	TRP	A	217	3.605	-6.621	-3.135	1.00	0.00	C
ATOM	461	CZ3	TRP	A	217	4.305	-5.384	-3.132	1.00	0.00	C
ATOM	462	CZ2	TRP	A	217	3.281	-4.730	-5.218	1.00	0.00	C
ATOM	463	CH2	TRP	A	217	4.158	-4.475	-4.181	1.00	0.00	C
ATOM	464	C	TRP	A	217	2.256	-11.686	-3.347	1.00	0.00	C
ATOM	465	O	TRP	A	217	1.722	-11.505	-2.264	1.00	0.00	O
ATOM	466	H	TRP	A	217	3.604	-10.333	-5.870	1.00	0.00	H
ATOM	467	HA	TRP	A	217	1.819	-10.828	-5.168	1.00	0.00	H
ATOM	468	HB2	TRP	A	217	0.539	-9.462	-3.728	1.00	0.00	H
ATOM	469	HB3	TRP	A	217	2.059	-9.054	-2.723	1.00	0.00	H
ATOM	470	HD1	TRP	A	217	0.617	-8.285	-6.357	1.00	0.00	H
ATOM	471	HE1	TRP	A	217	1.746	-6.154	-7.134	1.00	0.00	H
ATOM	472	HE3	TRP	A	217	3.758	-7.338	-2.340	1.00	0.00	H
ATOM	473	HZ2	TRP	A	217	3.268	-4.005	-6.022	1.00	0.00	H
ATOM	474	HZ3	TRP	A	217	5.015	-5.254	-2.330	1.00	0.00	H
ATOM	475	HH2	TRP	A	217	4.864	-3.655	-4.119	1.00	0.00	H
ATOM	476	N	GLY	A	218	2.643	-12.885	-3.808	1.00	0.00	N
ATOM	477	CA	GLY	A	218	2.472	-14.201	-3.146	1.00	0.00	C
ATOM	478	C	GLY	A	218	3.718	-14.799	-2.515	1.00	0.00	C
ATOM	479	O	GLY	A	218	3.810	-16.062	-2.644	1.00	0.00	O
ATOM	480	H	GLY	A	218	3.103	-12.892	-4.707	1.00	0.00	H
ATOM	481	HA2	GLY	A	218	1.795	-14.028	-2.310	1.00	0.00	H
ATOM	482	HA3	GLY	A	218	2.071	-14.913	-3.899	1.00	0.00	H
ATOM	483	N	GLY	A	219	4.764	-14.214	-1.900	1.00	0.00	N
ATOM	484	CA	GLY	A	219	4.992	-12.855	-1.652	1.00	0.00	C
ATOM	485	C	GLY	A	219	4.239	-12.331	-0.436	1.00	0.00	C
ATOM	486	O	GLY	A	219	3.869	-13.188	0.441	1.00	0.00	O

ATOM	487	H	GLY	A	219	5.483	-14.843	-1.572	1.00	0.00	H
ATOM	488	HA2	GLY	A	219	6.061	-12.764	-1.458	1.00	0.00	H
ATOM	489	HA3	GLY	A	219	4.689	-12.248	-2.527	1.00	0.00	H
ATOM	490	N	PHE	A	220	3.969	-11.000	-0.364	1.00	0.00	N
ATOM	491	CA	PHE	A	220	3.387	-10.278	0.841	1.00	0.00	C
ATOM	492	CB	PHE	A	220	3.207	-8.844	0.265	1.00	0.00	C
ATOM	493	CG	PHE	A	220	2.849	-7.880	1.375	1.00	0.00	C
ATOM	494	CD1	PHE	A	220	1.595	-7.301	1.307	1.00	0.00	C
ATOM	495	CE1	PHE	A	220	1.168	-6.415	2.301	1.00	0.00	C
ATOM	496	CZ	PHE	A	220	2.128	-5.954	3.274	1.00	0.00	C
ATOM	497	CD2	PHE	A	220	3.770	-7.452	2.300	1.00	0.00	C
ATOM	498	CE2	PHE	A	220	3.383	-6.515	3.301	1.00	0.00	C
ATOM	499	C	PHE	A	220	2.125	-10.882	1.347	1.00	0.00	C
ATOM	500	O	PHE	A	220	1.928	-10.886	2.548	1.00	0.00	O
ATOM	501	H	PHE	A	220	4.245	-10.436	-1.142	1.00	0.00	H
ATOM	502	HA	PHE	A	220	4.057	-10.126	1.661	1.00	0.00	H
ATOM	503	HB2	PHE	A	220	2.438	-8.932	-0.521	1.00	0.00	H
ATOM	504	HB3	PHE	A	220	4.128	-8.519	-0.266	1.00	0.00	H
ATOM	505	HD1	PHE	A	220	0.862	-7.684	0.612	1.00	0.00	H
ATOM	506	HD2	PHE	A	220	4.756	-7.890	2.325	1.00	0.00	H
ATOM	507	HE1	PHE	A	220	0.224	-5.893	2.181	1.00	0.00	H
ATOM	508	HE2	PHE	A	220	4.022	-6.400	4.164	1.00	0.00	H
ATOM	509	HZ	PHE	A	220	1.831	-5.332	4.098	1.00	0.00	H
ATOM	510	N	PHE	A	221	1.272	-11.436	0.492	1.00	0.00	N
ATOM	511	CA	PHE	A	221	0.120	-12.209	0.892	1.00	0.00	C
ATOM	512	CB	PHE	A	221	-1.010	-11.248	1.430	1.00	0.00	C
ATOM	513	CG	PHE	A	221	-1.487	-10.211	0.438	1.00	0.00	C
ATOM	514	CD1	PHE	A	221	-1.690	-10.565	-0.914	1.00	0.00	C
ATOM	515	CE1	PHE	A	221	-1.808	-9.560	-1.879	1.00	0.00	C

ATOM	516	CZ	PHE	A	221	-2.053	-8.224	-1.455	1.00	0.00	C
ATOM	517	CD2	PHE	A	221	-1.832	-8.926	0.843	1.00	0.00	C
ATOM	518	CE2	PHE	A	221	-2.050	-7.938	-0.102	1.00	0.00	C
ATOM	519	C	PHE	A	221	0.346	-13.372	1.920	1.00	0.00	C
ATOM	520	O	PHE	A	221	-0.489	-13.675	2.772	1.00	0.00	O
ATOM	521	H	PHE	A	221	1.396	-11.497	-0.494	1.00	0.00	H
ATOM	522	HA	PHE	A	221	-0.281	-12.708	0.019	1.00	0.00	H
ATOM	523	HB2	PHE	A	221	-2.007	-11.618	1.743	1.00	0.00	H
ATOM	524	HB3	PHE	A	221	-0.588	-10.738	2.322	1.00	0.00	H
ATOM	525	HD1	PHE	A	221	-1.514	-11.563	-1.252	1.00	0.00	H
ATOM	526	HD2	PHE	A	221	-1.784	-8.565	1.851	1.00	0.00	H
ATOM	527	HE1	PHE	A	221	-1.875	-9.784	-2.937	1.00	0.00	H
ATOM	528	HE2	PHE	A	221	-2.196	-6.895	0.173	1.00	0.00	H
ATOM	529	HZ	PHE	A	221	-2.216	-7.421	-2.166	1.00	0.00	H
ATOM	530	N	GLY	A	222	1.462	-14.095	1.766	1.00	0.00	N
ATOM	531	CA	GLY	A	222	1.730	-15.323	2.588	1.00	0.00	C
ATOM	532	C	GLY	A	222	2.204	-14.868	3.976	1.00	0.00	C
ATOM	533	O	GLY	A	222	2.074	-15.609	4.969	1.00	0.00	O
ATOM	534	H	GLY	A	222	2.143	-13.807	1.078	1.00	0.00	H
ATOM	535	HA2	GLY	A	222	0.834	-15.936	2.681	1.00	0.00	H
ATOM	536	HA3	GLY	A	222	2.503	-15.893	2.111	1.00	0.00	H
ATOM	537	HC	GLY	A	222	2.656	-13.894	4.093	1.00	0.00	H
ATOM	538	N	GLY	A	242	9.958	-7.149	-0.122	1.00	0.00	N
ATOM	539	CA	GLY	A	242	8.807	-7.477	-0.937	1.00	0.00	C
ATOM	540	C	GLY	A	242	8.176	-6.233	-1.585	1.00	0.00	C
ATOM	541	O	GLY	A	242	8.054	-6.166	-2.775	1.00	0.00	O
ATOM	542	H1	GLY	A	242	10.736	-7.583	-0.578	1.00	0.00	H
ATOM	543	H2	GLY	A	242	9.695	-7.386	0.814	1.00	0.00	H
ATOM	544	HA2	GLY	A	242	9.154	-8.138	-1.731	1.00	0.00	H

ATOM	545	HA3	GLY	A	242	8.047	-7.968	-0.318	1.00	0.00	H
ATOM	546	N	THR	A	243	7.853	-5.191	-0.781	1.00	0.00	N
ATOM	547	CA	THR	A	243	7.482	-3.828	-1.264	1.00	0.00	C
ATOM	548	CB	THR	A	243	7.205	-2.705	-0.164	1.00	0.00	C
ATOM	549	OG1	THR	A	243	6.623	-3.299	1.013	1.00	0.00	O
ATOM	550	CG2	THR	A	243	6.241	-1.650	-0.683	1.00	0.00	C
ATOM	551	C	THR	A	243	8.422	-3.166	-2.270	1.00	0.00	C
ATOM	552	O	THR	A	243	8.001	-2.444	-3.179	1.00	0.00	O
ATOM	553	H	THR	A	243	7.778	-5.322	0.200	1.00	0.00	H
ATOM	554	HA	THR	A	243	6.558	-4.018	-1.794	1.00	0.00	H
ATOM	555	HB	THR	A	243	8.174	-2.251	0.148	1.00	0.00	H
ATOM	556	HG21	THR	A	243	6.469	-1.212	-1.683	1.00	0.00	H
ATOM	557	HG22	THR	A	243	6.260	-0.725	-0.076	1.00	0.00	H
ATOM	558	HG23	THR	A	243	5.197	-2.019	-0.641	1.00	0.00	H
ATOM	559	HG1	THR	A	243	7.294	-3.904	1.336	1.00	0.00	H
ATOM	560	N	GLY	A	244	9.758	-3.254	-2.065	1.00	0.00	N
ATOM	561	CA	GLY	A	244	10.922	-2.896	-2.806	1.00	0.00	C
ATOM	562	C	GLY	A	244	10.926	-3.512	-4.146	1.00	0.00	C
ATOM	563	O	GLY	A	244	11.146	-2.938	-5.216	1.00	0.00	O
ATOM	564	H	GLY	A	244	9.983	-3.674	-1.174	1.00	0.00	H
ATOM	565	HA2	GLY	A	244	10.923	-1.814	-2.937	1.00	0.00	H
ATOM	566	HA3	GLY	A	244	11.818	-3.217	-2.249	1.00	0.00	H
ATOM	567	HC	GLY	A	244	10.716	-4.570	-4.196	1.00	0.00	H
TER	568		GLY	A	244						
HETATM	569	C1	ARI	B	274	2.688	-3.717	0.387	1.00	0.00	C
HETATM	570	C2	ARI	B	274	1.646	-4.385	-0.553	1.00	0.00	C
HETATM	571	O1	ARI	B	274	0.271	-4.337	-0.184	1.00	0.00	O
HETATM	572	C3	ARI	B	274	-0.648	-4.574	-1.113	1.00	0.00	C
HETATM	573	C4	ARI	B	274	-3.590	-4.436	-4.371	1.00	0.00	C

HETATM	574	C5	ARI	B	274	-2.628	-4.488	-3.141	1.00	0.00	C
HETATM	575	C6	ARI	B	274	-3.013	-4.840	-5.759	1.00	0.00	C
HETATM	576	C7	ARI	B	274	-1.367	-4.893	-3.446	1.00	0.00	C
HETATM	577	C8	ARI	B	274	-2.973	-4.073	-1.852	1.00	0.00	C
HETATM	578	C9	ARI	B	274	-1.811	-5.681	-5.732	1.00	0.00	C
HETATM	579	N1	ARI	B	274	-1.056	-5.610	-4.624	1.00	0.00	N
HETATM	580	C10	ARI	B	274	-0.393	-5.029	-2.410	1.00	0.00	C
HETATM	581	C11	ARI	B	274	-1.956	-3.984	-0.882	1.00	0.00	C
HETATM	582	O2	ARI	B	274	-1.401	-6.246	-6.720	1.00	0.00	O
HETATM	583	C12	ARI	B	274	2.485	-2.225	0.760	1.00	0.00	C
HETATM	584	C13	ARI	B	274	3.544	-1.771	1.795	1.00	0.00	C
HETATM	585	N2	ARI	B	274	3.240	-0.375	2.213	1.00	0.00	N1+
HETATM	586	C14	ARI	B	274	2.252	-0.446	3.358	1.00	0.00	C
HETATM	587	C15	ARI	B	274	4.443	0.414	2.475	1.00	0.00	C
HETATM	588	C16	ARI	B	274	1.905	0.933	3.947	1.00	0.00	C
HETATM	589	C17	ARI	B	274	4.027	1.837	2.980	1.00	0.00	C
HETATM	590	N3	ARI	B	274	3.110	1.807	4.142	1.00	0.00	N
HETATM	591	C18	ARI	B	274	3.134	2.780	5.147	1.00	0.00	C
HETATM	592	C19	ARI	B	274	4.304	2.810	5.863	1.00	0.00	C
HETATM	593	C20	ARI	B	274	2.178	3.790	5.403	1.00	0.00	C
HETATM	594	C21	ARI	B	274	4.497	3.792	6.878	1.00	0.00	C
HETATM	595	C22	ARI	B	274	2.384	4.862	6.272	1.00	0.00	C
HETATM	596	CL1	ARI	B	274	0.825	3.812	4.260	1.00	0.00	CL
HETATM	597	C23	ARI	B	274	3.592	4.809	7.095	1.00	0.00	C
HETATM	598	CL2	ARI	B	274	1.262	6.150	6.477	1.00	0.00	CL
HETATM	599	H	ARI	B	274	-0.211	-6.137	-4.585	1.00	0.00	H
HETATM	600	HC2	ARI	B	274	3.733	-3.803	0.026	1.00	0.00	H
HETATM	601	HC3	ARI	B	274	2.710	-4.146	1.420	1.00	0.00	H
HETATM	602	HC4	ARI	B	274	1.748	-5.485	-0.634	1.00	0.00	H

```
HETATM  603  HC5 ARI B 274       1.774  -3.947  -1.565  1.00  0.00           H
HETATM  604  HC6 ARI B 274      -4.192  -3.513  -4.445  1.00  0.00           H
HETATM  605  HC7 ARI B 274      -4.391  -5.147  -4.041  1.00  0.00           H
HETATM  606  HC8 ARI B 274      -2.687  -3.987  -6.406  1.00  0.00           H
HETATM  607  HC9 ARI B 274      -3.810  -5.382  -6.303  1.00  0.00           H
HETATM  608  HC  ARI B 274      -3.956  -3.790  -1.515  1.00  0.00           H
HETATM  609  HC1 ARI B 274       0.507  -5.529  -2.703  1.00  0.00           H
HETATM  610 HC10 ARI B 274      -2.258  -3.661   0.104  1.00  0.00           H
HETATM  611 HC11 ARI B 274       1.460  -2.107   1.168  1.00  0.00           H
HETATM  612 HC12 ARI B 274       2.483  -1.619  -0.166  1.00  0.00           H
HETATM  613 HC13 ARI B 274       4.618  -1.749   1.537  1.00  0.00           H
HETATM  614 HC14 ARI B 274       3.416  -2.356   2.717  1.00  0.00           H
HETATM  615  HN  ARI B 274       2.704   0.118   1.459  1.00  0.00           H
HETATM  616 HC15 ARI B 274       2.776  -0.996   4.156  1.00  0.00           H
HETATM  617 HC16 ARI B 274       1.404  -0.998   2.954  1.00  0.00           H
HETATM  618 HC17 ARI B 274       5.046   0.505   1.564  1.00  0.00           H
HETATM  619 HC18 ARI B 274       4.916  -0.112   3.321  1.00  0.00           H
HETATM  620 HC19 ARI B 274       1.361   0.815   4.900  1.00  0.00           H
HETATM  621 HC20 ARI B 274       1.210   1.409   3.222  1.00  0.00           H
HETATM  622 HC21 ARI B 274       3.695   2.430   2.101  1.00  0.00           H
HETATM  623 HC22 ARI B 274       4.986   2.263   3.314  1.00  0.00           H
HETATM  624 HC23 ARI B 274       5.091   2.106   5.653  1.00  0.00           H
HETATM  625 HC24 ARI B 274       5.377   3.668   7.501  1.00  0.00           H
HETATM  626 HC25 ARI B 274       3.836   5.457   7.935  1.00  0.00           H
HETATM  627  OH2 TIP X2153       8.909   4.922   8.835  1.00  0.00           O
HETATM  628  H1  TIP X2153       9.712   5.423   8.941  1.00  0.00           H
HETATM  629  H2  TIP X2153       8.683   4.999   7.904  1.00  0.00           H
HETATM  630  OH2 TIP X4556       7.385   0.940   3.350  1.00  0.00           O
HETATM  631  H1  TIP X4556       7.585   0.004   3.470  1.00  0.00           H
```

HETATM	632	H2	TIP	X4556	7.919	1.382	4.008	1.00	0.00	H
HETATM	633	OH2	TIP	X8972	5.334	6.652	9.435	1.00	0.00	O
HETATM	634	H1	TIP	X8972	6.258	6.748	9.220	1.00	0.00	H
HETATM	635	H2	TIP	X8972	5.132	7.377	10.022	1.00	0.00	H
HETATM	636	OH2	TIP	X2076	8.728	0.803	5.855	1.00	0.00	O
HETATM	637	H1	TIP	X2076	8.647	-0.126	6.030	1.00	0.00	H
HETATM	638	H2	TIP	X2076	8.014	1.214	6.327	1.00	0.00	H
HETATM	639	OH2	TIP	X1100	6.655	1.335	7.714	1.00	0.00	O
HETATM	640	H1	TIP	X1100	5.839	0.841	7.848	1.00	0.00	H
HETATM	641	H2	TIP	X1100	6.934	1.575	8.593	1.00	0.00	H
HETATM	642	OH2	TIP	X1259	3.545	-3.237	5.738	1.00	0.00	O
HETATM	643	H1	TIP	X1259	3.595	-2.885	6.617	1.00	0.00	H
HETATM	644	H2	TIP	X1259	2.629	-3.155	5.499	1.00	0.00	H
HETATM	645	OH2	TIP	X4778	6.889	1.946	10.571	1.00	0.00	O
HETATM	646	H1	TIP	X4778	7.497	2.673	10.448	1.00	0.00	H
HETATM	647	H2	TIP	X4778	7.429	1.207	10.832	1.00	0.00	H
HETATM	648	OH2	TIP	X6596	-0.638	-0.526	4.677	1.00	0.00	O
HETATM	649	H1	TIP	X6596	-1.262	-0.099	5.250	1.00	0.00	H
HETATM	650	H2	TIP	X6596	-0.842	-0.205	3.799	1.00	0.00	H
HETATM	651	OH2	TIP	X8875	4.109	1.854	10.469	1.00	0.00	O
HETATM	652	H1	TIP	X8875	3.916	2.795	10.560	1.00	0.00	H
HETATM	653	H2	TIP	X8875	5.043	1.829	10.233	1.00	0.00	H
HETATM	654	OH2	TIP	X 276	-0.922	0.475	2.065	1.00	0.00	O
HETATM	655	H1	TIP	X 276	-0.095	0.901	1.816	1.00	0.00	H
HETATM	656	H2	TIP	X 276	-1.342	0.271	1.242	1.00	0.00	H
HETATM	657	OH2	TIP	X3250	-0.735	-3.161	2.275	1.00	0.00	O
HETATM	658	H1	TIP	X3250	-0.206	-3.400	1.517	1.00	0.00	H
HETATM	659	H2	TIP	X3250	-0.687	-2.207	2.299	1.00	0.00	H
HETATM	660	OH2	TIP	X5210	8.048	1.913	0.895	1.00	0.00	O

```
HETATM  661  H1  TIP X5210       7.893   1.649   1.813  1.00  0.00           H
HETATM  662  H2  TIP X5210       7.225   2.292   0.616  1.00  0.00           H
HETATM  663  OH2 TIP X5759       8.099  -1.685   3.616  1.00  0.00           O
HETATM  664  H1  TIP X5759       7.538  -2.344   4.006  1.00  0.00           H
HETATM  665  H2  TIP X5759       8.973  -1.858   3.971  1.00  0.00           H
HETATM  666  OH2 TIP X3610       0.906  -3.101   5.041  1.00  0.00           O
HETATM  667  H1  TIP X3610       0.150  -3.680   5.121  1.00  0.00           H
HETATM  668  H2  TIP X3610       0.534  -2.218   5.003  1.00  0.00           H
HETATM  669  OH2 TIP X6562      -0.300  -1.063  -0.324  1.00  0.00           O
HETATM  670  H1  TIP X6562       0.221  -0.340  -0.668  1.00  0.00           H
HETATM  671  H2  TIP X6562      -0.098  -1.798  -0.910  1.00  0.00           H
HETATM  672  OH2 TIP X8198       6.125  -3.342   5.010  1.00  0.00           O
HETATM  673  H1  TIP X8198       5.185  -3.197   5.097  1.00  0.00           H
HETATM  674  H2  TIP X8198       6.380  -3.747   5.844  1.00  0.00           H
HETATM  675  OH2 TIP X 723       5.497   2.434  -0.160  1.00  0.00           O
HETATM  676  H1  TIP X 723       5.261   3.158  -0.726  1.00  0.00           H
HETATM  677  H2  TIP X 723       4.743   1.869  -0.161  1.00  0.00           H
HETATM  678  OH2 TIP X3503       4.028   4.624  10.783  1.00  0.00           O
HETATM  679  H1  TIP X3503       4.313   5.203  10.075  1.00  0.00           H
HETATM  680  H2  TIP X3503       3.355   5.124  11.240  1.00  0.00           H
HETATM  681  OH2 TIP X4247       4.032   0.413   8.084  1.00  0.00           O
HETATM  682  H1  TIP X4247       3.824   0.894   8.899  1.00  0.00           H
HETATM  683  H2  TIP X4247       3.957   1.077   7.394  1.00  0.00           H
END
```

Aripiprazole (Cluster structure 3)

```
ATOM      1  N   GLY A  56       5.963   8.715   0.872  1.00  0.00           N1+
ATOM      2  CA  GLY A  56       5.375   7.927   1.907  1.00  0.00           C
ATOM      3  C   GLY A  56       6.149   7.792   3.213  1.00  0.00           C
```

ATOM	4	O	GLY	A	56	6.749	8.753	3.728	1.00	0.00	O
ATOM	5	H1	GLY	A	56	5.387	9.511	0.768	1.00	0.00	H
ATOM	6	H2	GLY	A	56	6.193	8.089	0.143	1.00	0.00	H
ATOM	7	HA2	GLY	A	56	4.473	8.480	2.170	1.00	0.00	H
ATOM	8	HA3	GLY	A	56	5.180	6.894	1.515	1.00	0.00	H
ATOM	9	N	VAL	A	57	6.132	6.633	3.892	1.00	0.00	N
ATOM	10	CA	VAL	A	57	6.801	6.272	5.167	1.00	0.00	C
ATOM	11	CB	VAL	A	57	6.029	5.160	5.952	1.00	0.00	C
ATOM	12	CG1	VAL	A	57	4.720	5.727	6.485	1.00	0.00	C
ATOM	13	CG2	VAL	A	57	5.661	3.980	5.011	1.00	0.00	C
ATOM	14	C	VAL	A	57	8.187	5.822	4.883	1.00	0.00	C
ATOM	15	O	VAL	A	57	8.934	5.537	5.845	1.00	0.00	O
ATOM	16	H	VAL	A	57	5.597	5.901	3.504	1.00	0.00	H
ATOM	17	HA	VAL	A	57	6.897	7.170	5.756	1.00	0.00	H
ATOM	18	HB	VAL	A	57	6.612	4.881	6.854	1.00	0.00	H
ATOM	19	HG11	VAL	A	57	4.232	5.005	7.189	1.00	0.00	H
ATOM	20	HG12	VAL	A	57	4.038	5.970	5.642	1.00	0.00	H
ATOM	21	HG13	VAL	A	57	4.992	6.689	6.963	1.00	0.00	H
ATOM	22	HG21	VAL	A	57	5.198	3.197	5.658	1.00	0.00	H
ATOM	23	HG22	VAL	A	57	6.508	3.429	4.567	1.00	0.00	H
ATOM	24	HG23	VAL	A	57	4.946	4.284	4.214	1.00	0.00	H
ATOM	25	N	GLY	A	58	8.658	5.833	3.658	1.00	0.00	N
ATOM	26	CA	GLY	A	58	10.062	6.003	3.322	1.00	0.00	C
ATOM	27	C	GLY	A	58	10.575	7.327	3.826	1.00	0.00	C
ATOM	28	O	GLY	A	58	11.503	7.326	4.615	1.00	0.00	O
ATOM	29	H	GLY	A	58	8.002	5.716	2.899	1.00	0.00	H
ATOM	30	HA2	GLY	A	58	10.636	5.206	3.795	1.00	0.00	H
ATOM	31	HA3	GLY	A	58	10.178	5.959	2.236	1.00	0.00	H
ATOM	32	HC	GLY	A	58	10.131	8.254	3.496	1.00	0.00	H

ATOM	33	N	GLY	A	67	7.337	9.111	14.047	1.00	0.00	N1+
ATOM	34	CA	GLY	A	67	6.508	10.290	13.725	1.00	0.00	C
ATOM	35	C	GLY	A	67	5.499	9.974	12.758	1.00	0.00	C
ATOM	36	O	GLY	A	67	5.714	9.254	11.790	1.00	0.00	O
ATOM	37	H1	GLY	A	67	7.166	8.915	15.023	1.00	0.00	H
ATOM	38	H2	GLY	A	67	8.270	9.339	13.733	1.00	0.00	H
ATOM	39	HA2	GLY	A	67	5.977	10.633	14.613	1.00	0.00	H
ATOM	40	HA3	GLY	A	67	7.186	11.076	13.356	1.00	0.00	H
ATOM	41	N	PHE	A	68	4.277	10.587	12.888	1.00	0.00	N
ATOM	42	CA	PHE	A	68	3.221	10.673	11.923	1.00	0.00	C
ATOM	43	CB	PHE	A	68	2.097	9.572	12.084	1.00	0.00	C
ATOM	44	CG	PHE	A	68	2.611	8.192	11.567	1.00	0.00	C
ATOM	45	CD1	PHE	A	68	2.783	8.003	10.158	1.00	0.00	C
ATOM	46	CE1	PHE	A	68	3.273	6.784	9.637	1.00	0.00	C
ATOM	47	CZ	PHE	A	68	3.342	5.653	10.456	1.00	0.00	C
ATOM	48	CD2	PHE	A	68	2.634	7.032	12.341	1.00	0.00	C
ATOM	49	CE2	PHE	A	68	3.041	5.826	11.840	1.00	0.00	C
ATOM	50	C	PHE	A	68	2.608	12.035	11.806	1.00	0.00	C
ATOM	51	O	PHE	A	68	2.779	12.728	10.865	1.00	0.00	O
ATOM	52	H	PHE	A	68	4.110	11.095	13.722	1.00	0.00	H
ATOM	53	HA	PHE	A	68	3.751	10.662	10.976	1.00	0.00	H
ATOM	54	HB2	PHE	A	68	1.232	9.881	11.462	1.00	0.00	H
ATOM	55	HB3	PHE	A	68	1.706	9.477	13.107	1.00	0.00	H
ATOM	56	HD1	PHE	A	68	2.802	8.802	9.441	1.00	0.00	H
ATOM	57	HD2	PHE	A	68	2.429	7.171	13.394	1.00	0.00	H
ATOM	58	HE1	PHE	A	68	3.657	6.709	8.624	1.00	0.00	H
ATOM	59	HE2	PHE	A	68	2.980	5.029	12.572	1.00	0.00	H
ATOM	60	HZ	PHE	A	68	3.587	4.673	10.127	1.00	0.00	H
ATOM	61	N	GLY	A	69	1.871	12.513	12.857	1.00	0.00	N

ATOM	62	CA	GLY	A	69	1.137	13.790	12.797	1.00	0.00	C
ATOM	63	C	GLY	A	69	-0.244	13.447	12.261	1.00	0.00	C
ATOM	64	O	GLY	A	69	-0.563	12.437	11.647	1.00	0.00	O
ATOM	65	H	GLY	A	69	1.829	11.969	13.707	1.00	0.00	H
ATOM	66	HA2	GLY	A	69	1.081	14.277	13.771	1.00	0.00	H
ATOM	67	HA3	GLY	A	69	1.633	14.451	12.120	1.00	0.00	H
ATOM	68	HC	GLY	A	69	-1.026	14.170	12.441	1.00	0.00	H
ATOM	69	N	GLY	A	72	-3.476	12.123	8.190	1.00	0.00	N1+
ATOM	70	CA	GLY	A	72	-2.382	11.612	7.351	1.00	0.00	C
ATOM	71	C	GLY	A	72	-2.765	10.244	6.842	1.00	0.00	C
ATOM	72	O	GLY	A	72	-2.768	9.962	5.626	1.00	0.00	O
ATOM	73	H1	GLY	A	72	-3.808	12.951	7.707	1.00	0.00	H
ATOM	74	H2	GLY	A	72	-3.069	12.221	9.115	1.00	0.00	H
ATOM	75	HA2	GLY	A	72	-2.203	12.280	6.509	1.00	0.00	H
ATOM	76	HA3	GLY	A	72	-1.484	11.542	7.949	1.00	0.00	H
ATOM	77	N	CYS	A	73	-3.039	9.315	7.823	1.00	0.00	N
ATOM	78	CA	CYS	A	73	-2.956	7.951	7.558	1.00	0.00	C
ATOM	79	CB	CYS	A	73	-1.450	7.517	7.459	1.00	0.00	C
ATOM	80	SG	CYS	A	73	-0.369	7.922	8.806	1.00	0.00	S
ATOM	81	C	CYS	A	73	-3.878	7.461	6.471	1.00	0.00	C
ATOM	82	O	CYS	A	73	-3.567	6.608	5.686	1.00	0.00	O
ATOM	83	H	CYS	A	73	-3.265	9.715	8.701	1.00	0.00	H
ATOM	84	HA	CYS	A	73	-3.288	7.544	8.499	1.00	0.00	H
ATOM	85	HB2	CYS	A	73	-0.995	8.160	6.661	1.00	0.00	H
ATOM	86	HB3	CYS	A	73	-1.257	6.459	7.205	1.00	0.00	H
ATOM	87	N	GLY	A	74	-5.161	7.936	6.394	1.00	0.00	N
ATOM	88	CA	GLY	A	74	-6.235	7.552	5.440	1.00	0.00	C
ATOM	89	C	GLY	A	74	-5.945	7.800	3.931	1.00	0.00	C
ATOM	90	O	GLY	A	74	-6.076	6.892	3.117	1.00	0.00	O

ATOM	91	H	GLY	A	74	-5.410	8.639	7.075	1.00	0.00	H
ATOM	92	HA2	GLY	A	74	-6.326	6.470	5.539	1.00	0.00	H
ATOM	93	HA3	GLY	A	74	-7.157	8.105	5.711	1.00	0.00	H
ATOM	94	N	GLY	A	75	-5.387	9.010	3.645	1.00	0.00	N
ATOM	95	CA	GLY	A	75	-5.026	9.395	2.265	1.00	0.00	C
ATOM	96	C	GLY	A	75	-3.666	8.896	2.018	1.00	0.00	C
ATOM	97	O	GLY	A	75	-3.310	8.482	0.873	1.00	0.00	O
ATOM	98	H	GLY	A	75	-5.215	9.662	4.397	1.00	0.00	H
ATOM	99	HA2	GLY	A	75	-5.710	8.976	1.527	1.00	0.00	H
ATOM	100	HA3	GLY	A	75	-5.061	10.472	2.193	1.00	0.00	H
ATOM	101	N	PHE	A	76	-2.772	8.907	3.015	1.00	0.00	N
ATOM	102	CA	PHE	A	76	-1.449	8.230	3.037	1.00	0.00	C
ATOM	103	CB	PHE	A	76	-0.316	9.019	2.345	1.00	0.00	C
ATOM	104	CG	PHE	A	76	-0.285	10.360	2.935	1.00	0.00	C
ATOM	105	CD1	PHE	A	76	-0.560	11.549	2.172	1.00	0.00	C
ATOM	106	CE1	PHE	A	76	-0.486	12.798	2.707	1.00	0.00	C
ATOM	107	CZ	PHE	A	76	-0.059	12.896	4.043	1.00	0.00	C
ATOM	108	CD2	PHE	A	76	0.155	10.517	4.262	1.00	0.00	C
ATOM	109	CE2	PHE	A	76	0.249	11.775	4.798	1.00	0.00	C
ATOM	110	C	PHE	A	76	-1.504	6.750	2.627	1.00	0.00	C
ATOM	111	O	PHE	A	76	-0.811	6.364	1.721	1.00	0.00	O
ATOM	112	H	PHE	A	76	-2.951	9.295	3.918	1.00	0.00	H
ATOM	113	HA	PHE	A	76	-1.175	8.182	4.075	1.00	0.00	H
ATOM	114	HB2	PHE	A	76	-0.471	9.123	1.260	1.00	0.00	H
ATOM	115	HB3	PHE	A	76	0.672	8.504	2.450	1.00	0.00	H
ATOM	116	HD1	PHE	A	76	-0.786	11.465	1.121	1.00	0.00	H
ATOM	117	HD2	PHE	A	76	0.385	9.589	4.786	1.00	0.00	H
ATOM	118	HE1	PHE	A	76	-0.676	13.714	2.177	1.00	0.00	H
ATOM	119	HE2	PHE	A	76	0.663	11.893	5.801	1.00	0.00	H

```
ATOM    120  HZ  PHE A  76      -0.061  13.857   4.530  1.00  0.00           H
ATOM    121  N   VAL A  77      -2.287   5.908   3.228  1.00  0.00           N
ATOM    122  CA  VAL A  77      -2.486   4.534   2.962  1.00  0.00           C
ATOM    123  CB  VAL A  77      -3.063   3.846   4.199  1.00  0.00           C
ATOM    124  CG1 VAL A  77      -3.633   2.465   4.092  1.00  0.00           C
ATOM    125  CG2 VAL A  77      -1.953   3.789   5.230  1.00  0.00           C
ATOM    126  C   VAL A  77      -3.429   4.255   1.689  1.00  0.00           C
ATOM    127  O   VAL A  77      -3.372   3.211   1.038  1.00  0.00           O
ATOM    128  H   VAL A  77      -2.562   6.190   4.151  1.00  0.00           H
ATOM    129  HA  VAL A  77      -1.505   4.139   2.712  1.00  0.00           H
ATOM    130  HB  VAL A  77      -3.847   4.535   4.579  1.00  0.00           H
ATOM    131 HG11 VAL A  77      -3.822   2.033   5.100  1.00  0.00           H
ATOM    132 HG12 VAL A  77      -2.844   1.756   3.752  1.00  0.00           H
ATOM    133 HG13 VAL A  77      -4.627   2.429   3.608  1.00  0.00           H
ATOM    134 HG21 VAL A  77      -1.380   4.729   5.088  1.00  0.00           H
ATOM    135 HG22 VAL A  77      -1.239   2.943   5.064  1.00  0.00           H
ATOM    136 HG23 VAL A  77      -2.326   3.664   6.265  1.00  0.00           H
ATOM    137  N   GLY A  78      -4.366   5.198   1.357  1.00  0.00           N
ATOM    138  CA  GLY A  78      -5.084   5.171   0.038  1.00  0.00           C
ATOM    139  C   GLY A  78      -4.129   5.365  -1.043  1.00  0.00           C
ATOM    140  O   GLY A  78      -4.245   4.747  -2.079  1.00  0.00           O
ATOM    141  H   GLY A  78      -4.580   5.932   2.018  1.00  0.00           H
ATOM    142  HA2 GLY A  78      -5.574   4.212  -0.131  1.00  0.00           H
ATOM    143  HA3 GLY A  78      -5.829   5.966   0.059  1.00  0.00           H
ATOM    144  N   GLY A  79      -3.048   6.188  -0.905  1.00  0.00           N
ATOM    145  CA  GLY A  79      -1.996   6.389  -1.774  1.00  0.00           C
ATOM    146  C   GLY A  79      -1.326   5.084  -2.144  1.00  0.00           C
ATOM    147  O   GLY A  79      -0.993   4.900  -3.363  1.00  0.00           O
ATOM    148  H   GLY A  79      -3.014   6.723  -0.049  1.00  0.00           H
```

ATOM	149	HA2	GLY	A	79	-2.414	6.853	-2.668	1.00	0.00	H
ATOM	150	HA3	GLY	A	79	-1.234	7.047	-1.323	1.00	0.00	H
ATOM	151	N	ASP	A	80	-1.145	4.179	-1.172	1.00	0.00	N
ATOM	152	CA	ASP	A	80	-0.818	2.756	-1.389	1.00	0.00	C
ATOM	153	CB	ASP	A	80	0.732	2.601	-1.515	1.00	0.00	C
ATOM	154	CG	ASP	A	80	1.387	2.761	-0.134	1.00	0.00	C
ATOM	155	OD1	ASP	A	80	0.665	3.098	0.830	1.00	0.00	O
ATOM	156	OD2	ASP	A	80	2.619	2.557	-0.054	1.00	0.00	O1-
ATOM	157	C	ASP	A	80	-1.517	2.068	-2.540	1.00	0.00	C
ATOM	158	O	ASP	A	80	-0.922	1.685	-3.558	1.00	0.00	O
ATOM	159	H	ASP	A	80	-1.244	4.454	-0.209	1.00	0.00	H
ATOM	160	HA	ASP	A	80	-1.123	2.108	-0.584	1.00	0.00	H
ATOM	161	HB2	ASP	A	80	1.008	3.441	-2.183	1.00	0.00	H
ATOM	162	HB3	ASP	A	80	1.019	1.590	-1.879	1.00	0.00	H
ATOM	163	N	VAL	A	81	-2.836	1.995	-2.387	1.00	0.00	N
ATOM	164	CA	VAL	A	81	-3.751	1.345	-3.312	1.00	0.00	C
ATOM	165	CB	VAL	A	81	-5.141	1.325	-2.603	1.00	0.00	C
ATOM	166	CG1	VAL	A	81	-6.021	0.404	-3.482	1.00	0.00	C
ATOM	167	CG2	VAL	A	81	-4.959	0.771	-1.180	1.00	0.00	C
ATOM	168	C	VAL	A	81	-3.755	1.963	-4.694	1.00	0.00	C
ATOM	169	O	VAL	A	81	-3.762	1.271	-5.738	1.00	0.00	O
ATOM	170	H	VAL	A	81	-3.233	2.441	-1.589	1.00	0.00	H
ATOM	171	HA	VAL	A	81	-3.417	0.323	-3.379	1.00	0.00	H
ATOM	172	HB	VAL	A	81	-5.597	2.332	-2.710	1.00	0.00	H
ATOM	173	HG11	VAL	A	81	-6.323	1.004	-4.365	1.00	0.00	H
ATOM	174	HG12	VAL	A	81	-6.951	0.028	-3.031	1.00	0.00	H
ATOM	175	HG13	VAL	A	81	-5.462	-0.477	-3.887	1.00	0.00	H
ATOM	176	HG21	VAL	A	81	-5.954	0.727	-0.678	1.00	0.00	H
ATOM	177	HG22	VAL	A	81	-4.473	1.526	-0.526	1.00	0.00	H

ATOM	178	HG23	VAL	A	81	-4.470	-0.237	-1.190	1.00	0.00	H
ATOM	179	N	GLY	A	82	-3.690	3.342	-4.665	1.00	0.00	N
ATOM	180	CA	GLY	A	82	-3.702	4.091	-5.838	1.00	0.00	C
ATOM	181	C	GLY	A	82	-2.488	3.966	-6.727	1.00	0.00	C
ATOM	182	O	GLY	A	82	-2.626	3.619	-7.928	1.00	0.00	O
ATOM	183	H	GLY	A	82	-3.633	3.816	-3.775	1.00	0.00	H
ATOM	184	HA2	GLY	A	82	-4.535	3.674	-6.404	1.00	0.00	H
ATOM	185	HA3	GLY	A	82	-3.835	5.179	-5.593	1.00	0.00	H
ATOM	186	N	GLY	A	83	-1.326	4.216	-6.154	1.00	0.00	N
ATOM	187	CA	GLY	A	83	-0.022	3.989	-6.801	1.00	0.00	C
ATOM	188	C	GLY	A	83	0.394	2.593	-7.259	1.00	0.00	C
ATOM	189	O	GLY	A	83	0.832	2.275	-8.385	1.00	0.00	O
ATOM	190	H	GLY	A	83	-1.331	4.586	-5.214	1.00	0.00	H
ATOM	191	HA2	GLY	A	83	-0.140	4.509	-7.752	1.00	0.00	H
ATOM	192	HA3	GLY	A	83	0.778	4.382	-6.102	1.00	0.00	H
ATOM	193	N	CYS	A	84	0.075	1.535	-6.464	1.00	0.00	N
ATOM	194	CA	CYS	A	84	0.055	0.154	-6.891	1.00	0.00	C
ATOM	195	CB	CYS	A	84	-0.068	-0.850	-5.671	1.00	0.00	C
ATOM	196	SG	CYS	A	84	1.202	-0.512	-4.413	1.00	0.00	S
ATOM	197	C	CYS	A	84	-0.926	-0.244	-7.955	1.00	0.00	C
ATOM	198	O	CYS	A	84	-0.592	-0.848	-8.960	1.00	0.00	O
ATOM	199	H	CYS	A	84	-0.275	1.669	-5.548	1.00	0.00	H
ATOM	200	HA	CYS	A	84	1.055	-0.072	-7.250	1.00	0.00	H
ATOM	201	HB2	CYS	A	84	-0.039	-1.892	-6.064	1.00	0.00	H
ATOM	202	HB3	CYS	A	84	-1.028	-0.675	-5.136	1.00	0.00	H
ATOM	203	HG	CYS	A	84	0.525	0.543	-3.982	1.00	0.00	H
ATOM	204	N	THR	A	85	-2.200	0.182	-7.779	1.00	0.00	N
ATOM	205	CA	THR	A	85	-3.147	-0.057	-8.831	1.00	0.00	C
ATOM	206	CB	THR	A	85	-4.616	0.466	-8.587	1.00	0.00	C

ATOM	207	OG1	THR	A	85	-5.114	-0.085	-7.382	1.00	0.00	O
ATOM	208	CG2	THR	A	85	-5.583	0.058	-9.690	1.00	0.00	C
ATOM	209	C	THR	A	85	-2.849	0.551	-10.209	1.00	0.00	C
ATOM	210	O	THR	A	85	-2.926	-0.109	-11.241	1.00	0.00	O
ATOM	211	H	THR	A	85	-2.616	0.628	-6.983	1.00	0.00	H
ATOM	212	HA	THR	A	85	-3.237	-1.115	-8.986	1.00	0.00	H
ATOM	213	HB	THR	A	85	-4.660	1.558	-8.397	1.00	0.00	H
ATOM	214	HG21	THR	A	85	-5.753	-1.027	-9.773	1.00	0.00	H
ATOM	215	HG22	THR	A	85	-5.284	0.385	-10.715	1.00	0.00	H
ATOM	216	HG23	THR	A	85	-6.499	0.531	-9.283	1.00	0.00	H
ATOM	217	HG1	THR	A	85	-4.490	0.274	-6.730	1.00	0.00	H
ATOM	218	N	GLY	A	86	-2.372	1.815	-10.203	1.00	0.00	N
ATOM	219	CA	GLY	A	86	-1.813	2.483	-11.382	1.00	0.00	C
ATOM	220	C	GLY	A	86	-0.638	1.790	-12.101	1.00	0.00	C
ATOM	221	O	GLY	A	86	-0.667	1.559	-13.340	1.00	0.00	O
ATOM	222	H	GLY	A	86	-2.402	2.330	-9.335	1.00	0.00	H
ATOM	223	HA2	GLY	A	86	-2.626	2.469	-12.107	1.00	0.00	H
ATOM	224	HA3	GLY	A	86	-1.481	3.507	-11.085	1.00	0.00	H
ATOM	225	N	GLY	A	87	0.359	1.356	-11.342	1.00	0.00	N
ATOM	226	CA	GLY	A	87	1.544	0.663	-11.888	1.00	0.00	C
ATOM	227	C	GLY	A	87	1.106	-0.654	-12.514	1.00	0.00	C
ATOM	228	O	GLY	A	87	1.344	-1.004	-13.662	1.00	0.00	O
ATOM	229	H	GLY	A	87	0.306	1.506	-10.345	1.00	0.00	H
ATOM	230	HA2	GLY	A	87	2.025	1.283	-12.644	1.00	0.00	H
ATOM	231	HA3	GLY	A	87	2.239	0.467	-11.083	1.00	0.00	H
ATOM	232	N	ILE	A	88	0.445	-1.508	-11.784	1.00	0.00	N
ATOM	233	CA	ILE	A	88	-0.177	-2.661	-12.410	1.00	0.00	C
ATOM	234	C	ILE	A	88	-0.970	-2.414	-13.658	1.00	0.00	C
ATOM	235	O	ILE	A	88	-0.771	-3.132	-14.643	1.00	0.00	O

ATOM	236	CB	ILE	A	88	-1.033	-3.312	-11.416	1.00	0.00	C
ATOM	237	CG2	ILE	A	88	-1.785	-2.540	-10.409	1.00	0.00	C
ATOM	238	CG1	ILE	A	88	-2.164	-4.105	-11.950	1.00	0.00	C
ATOM	239	CD1	ILE	A	88	-2.087	-5.076	-10.871	1.00	0.00	C
ATOM	240	H	ILE	A	88	0.248	-1.308	-10.808	1.00	0.00	H
ATOM	241	HA	ILE	A	88	0.597	-3.366	-12.704	1.00	0.00	H
ATOM	242	HB	ILE	A	88	-0.215	-3.802	-10.886	1.00	0.00	H
ATOM	243	HG12	ILE	A	88	-3.139	-3.612	-11.997	1.00	0.00	H
ATOM	244	HG13	ILE	A	88	-2.156	-4.398	-12.983	1.00	0.00	H
ATOM	245	HG21	ILE	A	88	-2.874	-2.546	-10.379	1.00	0.00	H
ATOM	246	HG22	ILE	A	88	-1.442	-2.833	-9.425	1.00	0.00	H
ATOM	247	HG23	ILE	A	88	-1.532	-1.582	-10.695	1.00	0.00	H
ATOM	248	HD11	ILE	A	88	-2.863	-4.660	-10.271	1.00	0.00	H
ATOM	249	HD12	ILE	A	88	-2.076	-6.068	-11.209	1.00	0.00	H
ATOM	250	HD13	ILE	A	88	-1.375	-5.123	-10.096	1.00	0.00	H
ATOM	251	N	GLY	A	89	-1.863	-1.421	-13.569	1.00	0.00	N
ATOM	252	CA	GLY	A	89	-2.700	-1.024	-14.682	1.00	0.00	C
ATOM	253	C	GLY	A	89	-1.903	-0.924	-15.911	1.00	0.00	C
ATOM	254	O	GLY	A	89	-2.276	-1.313	-17.008	1.00	0.00	O
ATOM	255	H	GLY	A	89	-1.957	-0.929	-12.692	1.00	0.00	H
ATOM	256	HA2	GLY	A	89	-3.476	-1.772	-14.842	1.00	0.00	H
ATOM	257	HA3	GLY	A	89	-3.159	-0.057	-14.448	1.00	0.00	H
ATOM	258	HC	GLY	A	89	-0.922	-0.477	-15.841	1.00	0.00	H
ATOM	259	N	GLY	A	147	-6.657	5.989	12.697	1.00	0.00	N1+
ATOM	260	CA	GLY	A	147	-6.175	5.840	11.323	1.00	0.00	C
ATOM	261	C	GLY	A	147	-4.711	5.338	11.401	1.00	0.00	C
ATOM	262	O	GLY	A	147	-4.101	5.438	12.455	1.00	0.00	O
ATOM	263	H1	GLY	A	147	-7.426	5.326	12.769	1.00	0.00	H
ATOM	264	H2	GLY	A	147	-6.859	6.982	12.788	1.00	0.00	H

ATOM	265	HA2	GLY	A	147	-6.807	5.144	10.772	1.00	0.00	H
ATOM	266	HA3	GLY	A	147	-6.188	6.791	10.827	1.00	0.00	H
ATOM	267	N	CYS	A	148	-4.063	4.853	10.330	1.00	0.00	N
ATOM	268	CA	CYS	A	148	-2.620	4.662	10.151	1.00	0.00	C
ATOM	269	CB	CYS	A	148	-1.705	5.811	10.701	1.00	0.00	C
ATOM	270	SG	CYS	A	148	-0.246	6.078	9.727	1.00	0.00	S
ATOM	271	C	CYS	A	148	-2.201	3.257	10.468	1.00	0.00	C
ATOM	272	O	CYS	A	148	-1.094	3.088	11.047	1.00	0.00	O
ATOM	273	H	CYS	A	148	-4.637	4.792	9.510	1.00	0.00	H
ATOM	274	HA	CYS	A	148	-2.557	4.697	9.071	1.00	0.00	H
ATOM	275	HB2	CYS	A	148	-1.419	5.523	11.731	1.00	0.00	H
ATOM	276	HB3	CYS	A	148	-2.319	6.704	10.888	1.00	0.00	H
ATOM	277	N	ILE	A	149	-2.981	2.233	10.104	1.00	0.00	N
ATOM	278	CA	ILE	A	149	-2.723	0.793	10.222	1.00	0.00	C
ATOM	279	CB	ILE	A	149	-3.357	-0.087	9.150	1.00	0.00	C
ATOM	280	CG2	ILE	A	149	-4.881	-0.173	9.330	1.00	0.00	C
ATOM	281	CG1	ILE	A	149	-2.850	0.493	7.694	1.00	0.00	C
ATOM	282	CD1	ILE	A	149	-1.343	0.209	7.453	1.00	0.00	C
ATOM	283	C	ILE	A	149	-2.990	0.214	11.605	1.00	0.00	C
ATOM	284	O	ILE	A	149	-2.229	-0.612	12.098	1.00	0.00	O
ATOM	285	H	ILE	A	149	-3.824	2.392	9.596	1.00	0.00	H
ATOM	286	HA	ILE	A	149	-1.664	0.575	10.142	1.00	0.00	H
ATOM	287	HB	ILE	A	149	-2.935	-1.102	9.284	1.00	0.00	H
ATOM	288	HG12	ILE	A	149	-3.011	1.589	7.760	1.00	0.00	H
ATOM	289	HG13	ILE	A	149	-3.543	-0.005	6.985	1.00	0.00	H
ATOM	290	HG21	ILE	A	149	-5.386	0.805	9.197	1.00	0.00	H
ATOM	291	HG22	ILE	A	149	-5.179	-0.561	10.326	1.00	0.00	H
ATOM	292	HG23	ILE	A	149	-5.347	-0.939	8.668	1.00	0.00	H
ATOM	293	HD11	ILE	A	149	-1.212	-0.886	7.565	1.00	0.00	H

ATOM	294	HD12	ILE	A	149	-0.647	0.866	8.001	1.00	0.00	H
ATOM	295	HD13	ILE	A	149	-0.995	0.485	6.424	1.00	0.00	H
ATOM	296	N	GLY	A	150	-4.098	0.735	12.211	1.00	0.00	N
ATOM	297	CA	GLY	A	150	-4.514	0.502	13.595	1.00	0.00	C
ATOM	298	C	GLY	A	150	-5.757	-0.335	13.550	1.00	0.00	C
ATOM	299	O	GLY	A	150	-5.915	-1.309	14.292	1.00	0.00	O
ATOM	300	H	GLY	A	150	-4.685	1.337	11.652	1.00	0.00	H
ATOM	301	HA2	GLY	A	150	-3.734	-0.004	14.164	1.00	0.00	H
ATOM	302	HA3	GLY	A	150	-4.724	1.449	14.065	1.00	0.00	H
ATOM	303	HC	GLY	A	150	-6.537	-0.078	12.848	1.00	0.00	H
ATOM	304	N	GLY	A	162	-10.410	-8.795	-2.424	1.00	0.00	N1+
ATOM	305	CA	GLY	A	162	-10.734	-7.825	-3.443	1.00	0.00	C
ATOM	306	C	GLY	A	162	-9.537	-7.318	-4.347	1.00	0.00	C
ATOM	307	O	GLY	A	162	-9.658	-7.066	-5.510	1.00	0.00	O
ATOM	308	H1	GLY	A	162	-10.854	-9.621	-2.709	1.00	0.00	H
ATOM	309	H2	GLY	A	162	-10.546	-8.326	-1.574	1.00	0.00	H
ATOM	310	HA2	GLY	A	162	-11.412	-8.367	-4.102	1.00	0.00	H
ATOM	311	HA3	GLY	A	162	-11.188	-6.925	-2.974	1.00	0.00	H
ATOM	312	N	SER	A	163	-8.350	-7.089	-3.731	1.00	0.00	N
ATOM	313	CA	SER	A	163	-7.241	-6.669	-4.414	1.00	0.00	C
ATOM	314	CB	SER	A	163	-6.240	-5.752	-3.541	1.00	0.00	C
ATOM	315	OG	SER	A	163	-6.840	-4.499	-3.194	1.00	0.00	O
ATOM	316	C	SER	A	163	-6.403	-7.740	-5.028	1.00	0.00	C
ATOM	317	O	SER	A	163	-5.720	-7.516	-6.019	1.00	0.00	O
ATOM	318	H	SER	A	163	-8.309	-7.048	-2.741	1.00	0.00	H
ATOM	319	HA	SER	A	163	-7.448	-5.952	-5.206	1.00	0.00	H
ATOM	320	HB2	SER	A	163	-5.337	-5.445	-4.126	1.00	0.00	H
ATOM	321	HB3	SER	A	163	-5.941	-6.271	-2.609	1.00	0.00	H
ATOM	322	HG	SER	A	163	-7.551	-4.703	-2.578	1.00	0.00	H

ATOM	323	N	GLY	A	164	-6.578	-9.004	-4.587	1.00	0.00	N
ATOM	324	CA	GLY	A	164	-6.116	-10.212	-5.223	1.00	0.00	C
ATOM	325	C	GLY	A	164	-6.814	-10.540	-6.537	1.00	0.00	C
ATOM	326	O	GLY	A	164	-6.178	-11.044	-7.419	1.00	0.00	O
ATOM	327	H	GLY	A	164	-7.086	-9.115	-3.721	1.00	0.00	H
ATOM	328	HA2	GLY	A	164	-5.066	-10.042	-5.460	1.00	0.00	H
ATOM	329	HA3	GLY	A	164	-6.256	-11.065	-4.530	1.00	0.00	H
ATOM	330	N	GLY	A	165	-8.105	-10.155	-6.665	1.00	0.00	N
ATOM	331	CA	GLY	A	165	-8.993	-10.345	-7.781	1.00	0.00	C
ATOM	332	C	GLY	A	165	-8.596	-9.621	-9.109	1.00	0.00	C
ATOM	333	O	GLY	A	165	-8.977	-10.010	-10.226	1.00	0.00	O
ATOM	334	H	GLY	A	165	-8.498	-9.669	-5.871	1.00	0.00	H
ATOM	335	HA2	GLY	A	165	-8.937	-11.415	-7.983	1.00	0.00	H
ATOM	336	HA3	GLY	A	165	-10.014	-9.999	-7.509	1.00	0.00	H
ATOM	337	N	VAL	A	166	-7.827	-8.517	-8.978	1.00	0.00	N
ATOM	338	CA	VAL	A	166	-7.737	-7.482	-9.990	1.00	0.00	C
ATOM	339	CB	VAL	A	166	-8.307	-6.210	-9.448	1.00	0.00	C
ATOM	340	CG1	VAL	A	166	-9.703	-6.490	-8.950	1.00	0.00	C
ATOM	341	CG2	VAL	A	166	-7.515	-5.596	-8.336	1.00	0.00	C
ATOM	342	C	VAL	A	166	-6.362	-7.151	-10.642	1.00	0.00	C
ATOM	343	O	VAL	A	166	-6.266	-6.226	-11.472	1.00	0.00	O
ATOM	344	H	VAL	A	166	-7.409	-8.221	-8.127	1.00	0.00	H
ATOM	345	HA	VAL	A	166	-8.422	-7.749	-10.780	1.00	0.00	H
ATOM	346	HB	VAL	A	166	-8.483	-5.495	-10.257	1.00	0.00	H
ATOM	347	HG11	VAL	A	166	-10.235	-5.530	-8.754	1.00	0.00	H
ATOM	348	HG12	VAL	A	166	-9.651	-6.990	-7.955	1.00	0.00	H
ATOM	349	HG13	VAL	A	166	-10.252	-7.162	-9.642	1.00	0.00	H
ATOM	350	HG21	VAL	A	166	-6.545	-5.200	-8.691	1.00	0.00	H
ATOM	351	HG22	VAL	A	166	-7.317	-6.372	-7.564	1.00	0.00	H

ATOM	352	HG23	VAL	A	166	-8.059	-4.695	-7.969	1.00	0.00	H
ATOM	353	N	PRO	A	167	-5.242	-7.781	-10.411	1.00	0.00	N
ATOM	354	CD	PRO	A	167	-3.997	-7.064	-10.607	1.00	0.00	C
ATOM	355	CA	PRO	A	167	-5.004	-9.079	-9.713	1.00	0.00	C
ATOM	356	CB	PRO	A	167	-3.474	-9.132	-9.550	1.00	0.00	C
ATOM	357	CG	PRO	A	167	-3.055	-7.646	-9.592	1.00	0.00	C
ATOM	358	C	PRO	A	167	-5.415	-10.246	-10.628	1.00	0.00	C
ATOM	359	O	PRO	A	167	-5.587	-10.022	-11.796	1.00	0.00	O
ATOM	360	HA	PRO	A	167	-5.550	-9.038	-8.788	1.00	0.00	H
ATOM	361	HB2	PRO	A	167	-2.932	-9.713	-10.341	1.00	0.00	H
ATOM	362	HB3	PRO	A	167	-3.214	-9.710	-8.642	1.00	0.00	H
ATOM	363	HG2	PRO	A	167	-3.232	-7.088	-8.649	1.00	0.00	H
ATOM	364	HG3	PRO	A	167	-1.996	-7.614	-9.945	1.00	0.00	H
ATOM	365	HD2	PRO	A	167	-4.007	-5.953	-10.615	1.00	0.00	H
ATOM	366	HD3	PRO	A	167	-3.600	-7.320	-11.617	1.00	0.00	H
ATOM	367	N	GLY	A	168	-5.826	-11.362	-10.014	1.00	0.00	N
ATOM	368	CA	GLY	A	168	-6.239	-12.649	-10.496	1.00	0.00	C
ATOM	369	C	GLY	A	168	-6.904	-12.708	-11.848	1.00	0.00	C
ATOM	370	O	GLY	A	168	-6.362	-13.209	-12.791	1.00	0.00	O
ATOM	371	H	GLY	A	168	-5.850	-11.282	-9.007	1.00	0.00	H
ATOM	372	HA2	GLY	A	168	-5.304	-13.193	-10.630	1.00	0.00	H
ATOM	373	HA3	GLY	A	168	-6.933	-13.109	-9.741	1.00	0.00	H
ATOM	374	HC	GLY	A	168	-7.892	-12.289	-11.969	1.00	0.00	H
ATOM	375	N	GLY	A	216	6.405	-8.788	-8.286	1.00	0.00	N1+
ATOM	376	CA	GLY	A	216	6.088	-7.635	-7.534	1.00	0.00	C
ATOM	377	C	GLY	A	216	4.794	-7.798	-6.823	1.00	0.00	C
ATOM	378	O	GLY	A	216	4.613	-7.229	-5.760	1.00	0.00	O
ATOM	379	H1	GLY	A	216	7.260	-9.176	-7.887	1.00	0.00	H
ATOM	380	H2	GLY	A	216	6.417	-8.507	-9.267	1.00	0.00	H

ATOM	381	HA2	GLY	A	216	6.875	-7.486	-6.795	1.00	0.00	H
ATOM	382	HA3	GLY	A	216	6.027	-6.762	-8.202	1.00	0.00	H
ATOM	383	N	TRP	A	217	3.841	-8.526	-7.440	1.00	0.00	N
ATOM	384	CA	TRP	A	217	2.623	-8.872	-6.750	1.00	0.00	C
ATOM	385	CB	TRP	A	217	1.780	-7.610	-6.208	1.00	0.00	C
ATOM	386	CG	TRP	A	217	1.520	-6.432	-7.100	1.00	0.00	C
ATOM	387	CD1	TRP	A	217	0.327	-6.351	-7.839	1.00	0.00	C
ATOM	388	NE1	TRP	A	217	0.246	-5.209	-8.549	1.00	0.00	N
ATOM	389	CE2	TRP	A	217	1.338	-4.457	-8.269	1.00	0.00	C
ATOM	390	CD2	TRP	A	217	2.159	-5.214	-7.387	1.00	0.00	C
ATOM	391	CE3	TRP	A	217	3.290	-4.543	-6.941	1.00	0.00	C
ATOM	392	CZ3	TRP	A	217	3.629	-3.245	-7.404	1.00	0.00	C
ATOM	393	CZ2	TRP	A	217	1.703	-3.219	-8.708	1.00	0.00	C
ATOM	394	CH2	TRP	A	217	2.819	-2.567	-8.224	1.00	0.00	C
ATOM	395	C	TRP	A	217	2.855	-9.879	-5.641	1.00	0.00	C
ATOM	396	O	TRP	A	217	2.283	-9.827	-4.569	1.00	0.00	O
ATOM	397	H	TRP	A	217	3.975	-8.702	-8.410	1.00	0.00	H
ATOM	398	HA	TRP	A	217	1.951	-9.350	-7.457	1.00	0.00	H
ATOM	399	HB2	TRP	A	217	0.760	-7.954	-5.929	1.00	0.00	H
ATOM	400	HB3	TRP	A	217	2.273	-7.236	-5.279	1.00	0.00	H
ATOM	401	HD1	TRP	A	217	-0.327	-7.199	-7.747	1.00	0.00	H
ATOM	402	HE1	TRP	A	217	-0.568	-4.791	-8.875	1.00	0.00	H
ATOM	403	HE3	TRP	A	217	3.889	-4.933	-6.127	1.00	0.00	H
ATOM	404	HZ2	TRP	A	217	1.046	-2.587	-9.289	1.00	0.00	H
ATOM	405	HZ3	TRP	A	217	4.506	-2.785	-6.995	1.00	0.00	H
ATOM	406	HH2	TRP	A	217	3.019	-1.564	-8.519	1.00	0.00	H
ATOM	407	N	GLY	A	218	3.668	-10.948	-5.855	1.00	0.00	N
ATOM	408	CA	GLY	A	218	3.940	-12.177	-5.083	1.00	0.00	C
ATOM	409	C	GLY	A	218	5.177	-12.199	-4.098	1.00	0.00	C

ATOM	410	O	GLY	A	218	5.570	-13.364	-4.017	1.00	0.00	O
ATOM	411	H	GLY	A	218	4.192	-10.889	-6.717	1.00	0.00	H
ATOM	412	HA2	GLY	A	218	3.071	-12.248	-4.428	1.00	0.00	H
ATOM	413	HA3	GLY	A	218	4.071	-13.026	-5.797	1.00	0.00	H
ATOM	414	N	GLY	A	219	5.874	-11.172	-3.441	1.00	0.00	N
ATOM	415	CA	GLY	A	219	5.571	-9.760	-3.443	1.00	0.00	C
ATOM	416	C	GLY	A	219	4.661	-9.446	-2.240	1.00	0.00	C
ATOM	417	O	GLY	A	219	4.638	-10.244	-1.302	1.00	0.00	O
ATOM	418	H	GLY	A	219	6.682	-11.454	-2.904	1.00	0.00	H
ATOM	419	HA2	GLY	A	219	6.500	-9.193	-3.382	1.00	0.00	H
ATOM	420	HA3	GLY	A	219	5.046	-9.494	-4.351	1.00	0.00	H
ATOM	421	N	PHE	A	220	4.025	-8.223	-2.288	1.00	0.00	N
ATOM	422	CA	PHE	A	220	3.216	-7.622	-1.276	1.00	0.00	C
ATOM	423	CB	PHE	A	220	2.459	-6.336	-1.842	1.00	0.00	C
ATOM	424	CG	PHE	A	220	3.262	-5.163	-1.990	1.00	0.00	C
ATOM	425	CD1	PHE	A	220	3.339	-4.398	-3.233	1.00	0.00	C
ATOM	426	CE1	PHE	A	220	4.247	-3.319	-3.358	1.00	0.00	C
ATOM	427	CZ	PHE	A	220	4.868	-2.859	-2.204	1.00	0.00	C
ATOM	428	CD2	PHE	A	220	4.013	-4.768	-0.850	1.00	0.00	C
ATOM	429	CE2	PHE	A	220	4.763	-3.564	-0.964	1.00	0.00	C
ATOM	430	C	PHE	A	220	2.039	-8.571	-0.870	1.00	0.00	C
ATOM	431	O	PHE	A	220	1.530	-8.571	0.250	1.00	0.00	O
ATOM	432	H	PHE	A	220	4.207	-7.680	-3.106	1.00	0.00	H
ATOM	433	HA	PHE	A	220	3.795	-7.311	-0.418	1.00	0.00	H
ATOM	434	HB2	PHE	A	220	1.712	-5.988	-1.093	1.00	0.00	H
ATOM	435	HB3	PHE	A	220	1.856	-6.596	-2.734	1.00	0.00	H
ATOM	436	HD1	PHE	A	220	2.888	-4.864	-4.107	1.00	0.00	H
ATOM	437	HD2	PHE	A	220	4.205	-5.393	0.020	1.00	0.00	H
ATOM	438	HE1	PHE	A	220	4.262	-2.811	-4.316	1.00	0.00	H

ATOM	439	HE2	PHE	A	220	5.410	-3.385	-0.127	1.00	0.00	H
ATOM	440	HZ	PHE	A	220	5.569	-2.054	-2.371	1.00	0.00	H
ATOM	441	N	PHE	A	221	1.552	-9.256	-1.847	1.00	0.00	N
ATOM	442	CA	PHE	A	221	0.570	-10.382	-1.790	1.00	0.00	C
ATOM	443	CB	PHE	A	221	-0.914	-9.809	-1.669	1.00	0.00	C
ATOM	444	CG	PHE	A	221	-1.439	-9.222	-2.990	1.00	0.00	C
ATOM	445	CD1	PHE	A	221	-1.960	-7.953	-3.066	1.00	0.00	C
ATOM	446	CE1	PHE	A	221	-2.329	-7.422	-4.358	1.00	0.00	C
ATOM	447	CZ	PHE	A	221	-2.327	-8.225	-5.521	1.00	0.00	C
ATOM	448	CD2	PHE	A	221	-1.502	-10.063	-4.119	1.00	0.00	C
ATOM	449	CE2	PHE	A	221	-1.803	-9.534	-5.394	1.00	0.00	C
ATOM	450	C	PHE	A	221	0.719	-11.455	-0.680	1.00	0.00	C
ATOM	451	O	PHE	A	221	-0.240	-12.031	-0.222	1.00	0.00	O
ATOM	452	H	PHE	A	221	2.017	-9.153	-2.721	1.00	0.00	H
ATOM	453	HA	PHE	A	221	0.611	-10.965	-2.698	1.00	0.00	H
ATOM	454	HB2	PHE	A	221	-1.620	-10.604	-1.372	1.00	0.00	H
ATOM	455	HB3	PHE	A	221	-0.886	-9.028	-0.875	1.00	0.00	H
ATOM	456	HD1	PHE	A	221	-2.120	-7.266	-2.243	1.00	0.00	H
ATOM	457	HD2	PHE	A	221	-1.037	-11.030	-4.172	1.00	0.00	H
ATOM	458	HE1	PHE	A	221	-2.683	-6.399	-4.294	1.00	0.00	H
ATOM	459	HE2	PHE	A	221	-1.800	-10.106	-6.306	1.00	0.00	H
ATOM	460	HZ	PHE	A	221	-2.475	-7.731	-6.456	1.00	0.00	H
ATOM	461	N	GLY	A	222	1.968	-11.639	-0.206	1.00	0.00	N
ATOM	462	CA	GLY	A	222	2.144	-12.724	0.836	1.00	0.00	C
ATOM	463	C	GLY	A	222	1.611	-12.232	2.233	1.00	0.00	C
ATOM	464	O	GLY	A	222	0.858	-12.935	2.886	1.00	0.00	O
ATOM	465	H	GLY	A	222	2.746	-11.082	-0.529	1.00	0.00	H
ATOM	466	HA2	GLY	A	222	1.583	-13.608	0.531	1.00	0.00	H
ATOM	467	HA3	GLY	A	222	3.193	-12.951	0.925	1.00	0.00	H

ATOM	468	N	GLY	A	223	1.913	-11.012	2.521	1.00	0.00	N
ATOM	469	CA	GLY	A	223	1.367	-10.220	3.673	1.00	0.00	C
ATOM	470	C	GLY	A	223	-0.134	-10.027	3.472	1.00	0.00	C
ATOM	471	O	GLY	A	223	-0.912	-9.898	4.430	1.00	0.00	O
ATOM	472	H	GLY	A	223	2.576	-10.548	1.917	1.00	0.00	H
ATOM	473	HA2	GLY	A	223	1.541	-10.741	4.614	1.00	0.00	H
ATOM	474	HA3	GLY	A	223	1.859	-9.262	3.692	1.00	0.00	H
ATOM	475	N	HIS	A	224	-0.680	-10.040	2.243	1.00	0.00	N
ATOM	476	CA	HIS	A	224	-2.111	-9.930	1.930	1.00	0.00	C
ATOM	477	CB	HIS	A	224	-2.800	-8.654	2.449	1.00	0.00	C
ATOM	478	ND1	HIS	A	224	-2.750	-6.698	0.833	1.00	0.00	N
ATOM	479	CG	HIS	A	224	-2.222	-7.355	1.933	1.00	0.00	C
ATOM	480	CE1	HIS	A	224	-2.049	-5.512	0.704	1.00	0.00	C
ATOM	481	NE2	HIS	A	224	-1.100	-5.414	1.605	1.00	0.00	N
ATOM	482	CD2	HIS	A	224	-1.223	-6.558	2.423	1.00	0.00	C
ATOM	483	C	HIS	A	224	-2.764	-11.167	2.518	1.00	0.00	C
ATOM	484	O	HIS	A	224	-3.768	-11.050	3.239	1.00	0.00	O
ATOM	485	H	HIS	A	224	-0.055	-9.913	1.478	1.00	0.00	H
ATOM	486	HA	HIS	A	224	-2.285	-9.996	0.864	1.00	0.00	H
ATOM	487	HB2	HIS	A	224	-2.858	-8.490	3.548	1.00	0.00	H
ATOM	488	HB3	HIS	A	224	-3.827	-8.539	2.040	1.00	0.00	H
ATOM	489	HD1	HIS	A	224	-3.589	-6.929	0.347	1.00	0.00	H
ATOM	490	HD2	HIS	A	224	-0.555	-6.600	3.274	1.00	0.00	H
ATOM	491	HE1	HIS	A	224	-2.166	-4.769	-0.078	1.00	0.00	H
ATOM	492	N	GLY	A	225	-2.125	-12.340	2.375	1.00	0.00	N
ATOM	493	CA	GLY	A	225	-2.613	-13.563	3.112	1.00	0.00	C
ATOM	494	C	GLY	A	225	-2.495	-13.474	4.584	1.00	0.00	C
ATOM	495	O	GLY	A	225	-3.466	-13.689	5.271	1.00	0.00	O
ATOM	496	H	GLY	A	225	-1.316	-12.403	1.773	1.00	0.00	H

ATOM	497	HA2	GLY	A	225	-3.682	-13.670	2.929	1.00	0.00	H
ATOM	498	HA3	GLY	A	225	-2.034	-14.422	2.728	1.00	0.00	H
ATOM	499	HC	GLY	A	225	-1.550	-13.219	5.041	1.00	0.00	H
ATOM	500	N	GLY	A	246	9.257	-5.209	-4.261	1.00	0.00	N1+
ATOM	501	CA	GLY	A	246	8.184	-4.662	-5.110	1.00	0.00	C
ATOM	502	C	GLY	A	246	8.404	-3.250	-5.637	1.00	0.00	C
ATOM	503	O	GLY	A	246	8.306	-2.978	-6.827	1.00	0.00	O
ATOM	504	H1	GLY	A	246	10.197	-5.318	-4.613	1.00	0.00	H
ATOM	505	H2	GLY	A	246	9.021	-5.469	-3.321	1.00	0.00	H
ATOM	506	HA2	GLY	A	246	7.361	-4.632	-4.408	1.00	0.00	H
ATOM	507	HA3	GLY	A	246	7.979	-5.347	-5.914	1.00	0.00	H
ATOM	508	N	TYR	A	247	8.673	-2.337	-4.706	1.00	0.00	N
ATOM	509	CA	TYR	A	247	8.878	-0.914	-4.923	1.00	0.00	C
ATOM	510	CB	TYR	A	247	7.484	-0.095	-5.075	1.00	0.00	C
ATOM	511	CG	TYR	A	247	6.707	0.315	-3.867	1.00	0.00	C
ATOM	512	CD1	TYR	A	247	5.339	0.213	-3.819	1.00	0.00	C
ATOM	513	CE1	TYR	A	247	4.654	0.630	-2.621	1.00	0.00	C
ATOM	514	CZ	TYR	A	247	5.408	1.036	-1.501	1.00	0.00	C
ATOM	515	OH	TYR	A	247	4.914	1.386	-0.231	1.00	0.00	O
ATOM	516	CD2	TYR	A	247	7.437	0.902	-2.832	1.00	0.00	C
ATOM	517	CE2	TYR	A	247	6.769	1.178	-1.651	1.00	0.00	C
ATOM	518	C	TYR	A	247	9.991	-0.535	-5.916	1.00	0.00	C
ATOM	519	O	TYR	A	247	9.762	0.072	-7.002	1.00	0.00	O
ATOM	520	H	TYR	A	247	8.616	-2.594	-3.745	1.00	0.00	H
ATOM	521	HA	TYR	A	247	9.255	-0.595	-3.966	1.00	0.00	H
ATOM	522	HB2	TYR	A	247	7.565	0.752	-5.795	1.00	0.00	H
ATOM	523	HB3	TYR	A	247	6.979	-0.878	-5.661	1.00	0.00	H
ATOM	524	HD1	TYR	A	247	4.758	-0.118	-4.658	1.00	0.00	H
ATOM	525	HD2	TYR	A	247	8.516	0.964	-2.831	1.00	0.00	H

```
ATOM    526  HE1 TYR A 247       3.585   0.678  -2.681  1.00  0.00           H
ATOM    527  HE2 TYR A 247       7.412   1.577  -0.886  1.00  0.00           H
ATOM    528  HH  TYR A 247       4.058   1.786  -0.370  1.00  0.00           H
ATOM    529  N   GLY A 248      11.210  -0.967  -5.525  1.00  0.00           N
ATOM    530  CA  GLY A 248      12.418  -0.991  -6.415  1.00  0.00           C
ATOM    531  C   GLY A 248      12.099  -1.663  -7.725  1.00  0.00           C
ATOM    532  O   GLY A 248      12.168  -1.057  -8.767  1.00  0.00           O
ATOM    533  H   GLY A 248      11.315  -1.294  -4.575  1.00  0.00           H
ATOM    534  HA2 GLY A 248      12.739   0.028  -6.633  1.00  0.00           H
ATOM    535  HA3 GLY A 248      13.205  -1.534  -5.898  1.00  0.00           H
ATOM    536  HC  GLY A 248      11.804  -2.702  -7.732  1.00  0.00           H
TER     537      GLY A 248
HETATM  538  C1  ARI B 274       1.162  -1.775  -0.797  1.00  0.00           C
HETATM  539  C2  ARI B 274       0.341  -3.097  -0.630  1.00  0.00           C
HETATM  540  O1  ARI B 274      -0.031  -3.846  -1.755  1.00  0.00           O
HETATM  541  C3  ARI B 274      -1.050  -3.432  -2.587  1.00  0.00           C
HETATM  542  C4  ARI B 274      -4.213  -2.505  -5.415  1.00  0.00           C
HETATM  543  C5  ARI B 274      -3.198  -2.879  -4.352  1.00  0.00           C
HETATM  544  C6  ARI B 274      -4.280  -3.505  -6.644  1.00  0.00           C
HETATM  545  C7  ARI B 274      -2.054  -3.560  -4.793  1.00  0.00           C
HETATM  546  C8  ARI B 274      -3.266  -2.444  -2.993  1.00  0.00           C
HETATM  547  C9  ARI B 274      -2.883  -3.965  -7.027  1.00  0.00           C
HETATM  548  N1  ARI B 274      -1.903  -3.898  -6.142  1.00  0.00           N
HETATM  549  C10 ARI B 274      -1.015  -3.866  -3.929  1.00  0.00           C
HETATM  550  C11 ARI B 274      -2.232  -2.769  -2.170  1.00  0.00           C
HETATM  551  O2  ARI B 274      -2.600  -4.418  -8.160  1.00  0.00           O
HETATM  552  C12 ARI B 274       2.092  -1.286   0.301  1.00  0.00           C
HETATM  553  C13 ARI B 274       1.435  -1.190   1.686  1.00  0.00           C
HETATM  554  N2  ARI B 274       2.329  -0.759   2.821  1.00  0.00           N1+
```

HETATM	555	C14	ARI	B	274	2.881	0.612	2.724	1.00	0.00	C
HETATM	556	C15	ARI	B	274	1.590	-0.971	4.218	1.00	0.00	C
HETATM	557	C16	ARI	B	274	3.625	1.018	4.024	1.00	0.00	C
HETATM	558	C17	ARI	B	274	2.479	-0.520	5.403	1.00	0.00	C
HETATM	559	N3	ARI	B	274	2.760	0.861	5.215	1.00	0.00	N
HETATM	560	C18	ARI	B	274	2.168	1.904	5.958	1.00	0.00	C
HETATM	561	C19	ARI	B	274	2.306	1.781	7.373	1.00	0.00	C
HETATM	562	C20	ARI	B	274	1.637	3.112	5.508	1.00	0.00	C
HETATM	563	C21	ARI	B	274	1.969	2.825	8.275	1.00	0.00	C
HETATM	564	C22	ARI	B	274	1.445	4.221	6.396	1.00	0.00	C
HETATM	565	CL1	ARI	B	274	1.285	3.452	3.826	1.00	0.00	CL
HETATM	566	C23	ARI	B	274	1.585	4.049	7.771	1.00	0.00	C
HETATM	567	CL2	ARI	B	274	0.899	5.801	5.923	1.00	0.00	CL
HETATM	568	H	ARI	B	274	-0.999	-4.241	-6.320	1.00	0.00	H
HETATM	569	HC2	ARI	B	274	0.367	-1.057	-1.112	1.00	0.00	H
HETATM	570	HC3	ARI	B	274	1.805	-1.840	-1.700	1.00	0.00	H
HETATM	571	HC4	ARI	B	274	-0.573	-2.873	-0.040	1.00	0.00	H
HETATM	572	HC5	ARI	B	274	0.999	-3.679	0.051	1.00	0.00	H
HETATM	573	HC6	ARI	B	274	-3.787	-1.550	-5.765	1.00	0.00	H
HETATM	574	HC7	ARI	B	274	-5.176	-2.370	-4.870	1.00	0.00	H
HETATM	575	HC8	ARI	B	274	-4.715	-3.071	-7.561	1.00	0.00	H
HETATM	576	HC9	ARI	B	274	-4.846	-4.425	-6.371	1.00	0.00	H
HETATM	577	HC	ARI	B	274	-4.193	-2.000	-2.668	1.00	0.00	H
HETATM	578	HC1	ARI	B	274	-0.135	-4.417	-4.219	1.00	0.00	H
HETATM	579	HC10	ARI	B	274	-2.354	-2.572	-1.119	1.00	0.00	H
HETATM	580	HC11	ARI	B	274	2.463	-0.289	-0.004	1.00	0.00	H
HETATM	581	HC12	ARI	B	274	2.932	-2.006	0.310	1.00	0.00	H
HETATM	582	HC13	ARI	B	274	1.043	-2.166	2.000	1.00	0.00	H
HETATM	583	HC14	ARI	B	274	0.619	-0.461	1.741	1.00	0.00	H

HETATM	584	HN	ARI	B	274	3.120	-1.448	2.824	1.00	0.00	H
HETATM	585	HC15	ARI	B	274	2.062	1.301	2.527	1.00	0.00	H
HETATM	586	HC16	ARI	B	274	3.573	0.616	1.867	1.00	0.00	H
HETATM	587	HC17	ARI	B	274	1.251	-2.010	4.216	1.00	0.00	H
HETATM	588	HC18	ARI	B	274	0.743	-0.271	4.179	1.00	0.00	H
HETATM	589	HC19	ARI	B	274	3.831	2.102	3.960	1.00	0.00	H
HETATM	590	HC20	ARI	B	274	4.583	0.485	4.205	1.00	0.00	H
HETATM	591	HC21	ARI	B	274	3.412	-1.133	5.421	1.00	0.00	H
HETATM	592	HC22	ARI	B	274	1.921	-0.799	6.328	1.00	0.00	H
HETATM	593	HC23	ARI	B	274	2.775	0.885	7.763	1.00	0.00	H
HETATM	594	HC24	ARI	B	274	1.854	2.661	9.329	1.00	0.00	H
HETATM	595	HC25	ARI	B	274	1.398	4.817	8.511	1.00	0.00	H
HETATM	596	OH2	TIP	X1822		1.317	2.806	12.700	1.00	0.00	O
HETATM	597	H1	TIP	X1822		0.618	2.880	12.047	1.00	0.00	H
HETATM	598	H2	TIP	X1822		2.124	3.007	12.216	1.00	0.00	H
HETATM	599	OH2	TIP	X2803		4.093	3.990	1.785	1.00	0.00	O
HETATM	600	H1	TIP	X2803		3.420	4.654	1.886	1.00	0.00	H
HETATM	601	H2	TIP	X2803		3.772	3.429	1.068	1.00	0.00	H
HETATM	602	OH2	TIP	X9062		2.837	-4.584	5.779	1.00	0.00	O
HETATM	603	H1	TIP	X9062		1.905	-4.351	5.813	1.00	0.00	H
HETATM	604	H2	TIP	X9062		3.083	-4.661	6.702	1.00	0.00	H
HETATM	605	OH2	TIP	X9507		1.973	5.684	0.932	1.00	0.00	O
HETATM	606	H1	TIP	X9507		1.602	4.806	0.825	1.00	0.00	H
HETATM	607	H2	TIP	X9507		1.333	6.148	1.481	1.00	0.00	H
HETATM	608	OH2	TIP	X9847		4.768	1.829	9.844	1.00	0.00	O
HETATM	609	H1	TIP	X9847		4.180	1.079	9.913	1.00	0.00	H
HETATM	610	H2	TIP	X9847		5.275	1.660	9.047	1.00	0.00	H
HETATM	611	OH2	TIP	X2552		6.207	-3.723	5.721	1.00	0.00	O
HETATM	612	H1	TIP	X2552		6.858	-3.176	5.298	1.00	0.00	H

HETATM	613	H2	TIP	X2552	5.761	-4.168	4.989	1.00	0.00	H
HETATM	614	OH2	TIP	X3111	2.609	0.215	10.173	1.00	0.00	O
HETATM	615	H1	TIP	X3111	2.365	-0.398	9.475	1.00	0.00	H
HETATM	616	H2	TIP	X3111	2.110	-0.087	10.934	1.00	0.00	H
HETATM	617	OH2	TIP	X4711	1.669	-5.791	1.930	1.00	0.00	O
HETATM	618	H1	TIP	X4711	0.824	-5.371	1.792	1.00	0.00	H
HETATM	619	H2	TIP	X4711	1.624	-6.581	1.376	1.00	0.00	H
HETATM	620	OH2	TIP	X 187	4.076	2.808	12.347	1.00	0.00	O
HETATM	621	H1	TIP	X 187	4.282	2.579	11.435	1.00	0.00	H
HETATM	622	H2	TIP	X 187	4.664	2.278	12.859	1.00	0.00	H
HETATM	623	OH2	TIP	X 433	7.633	1.067	4.123	1.00	0.00	O
HETATM	624	H1	TIP	X 433	8.418	1.610	4.118	1.00	0.00	H
HETATM	625	H2	TIP	X 433	7.078	1.446	3.455	1.00	0.00	H
HETATM	626	OH2	TIP	X3274	-2.756	-2.003	1.449	1.00	0.00	O
HETATM	627	H1	TIP	X3274	-2.387	-2.215	2.307	1.00	0.00	H
HETATM	628	H2	TIP	X3274	-2.547	-1.080	1.317	1.00	0.00	H
HETATM	629	OH2	TIP	X 424	2.170	-1.884	8.516	1.00	0.00	O
HETATM	630	H1	TIP	X 424	1.965	-2.807	8.699	1.00	0.00	H
HETATM	631	H2	TIP	X 424	3.105	-1.887	8.349	1.00	0.00	H
HETATM	632	OH2	TIP	X 856	-0.572	1.594	2.581	1.00	0.00	O
HETATM	633	H1	TIP	X 856	-0.942	2.228	3.199	1.00	0.00	H
HETATM	634	H2	TIP	X 856	-0.081	2.106	1.959	1.00	0.00	H
HETATM	635	OH2	TIP	X3761	4.767	-2.448	7.634	1.00	0.00	O
HETATM	636	H1	TIP	X3761	5.140	-1.611	7.350	1.00	0.00	H
HETATM	637	H2	TIP	X3761	5.006	-3.068	6.930	1.00	0.00	H
HETATM	638	OH2	TIP	X5455	6.269	1.942	1.942	1.00	0.00	O
HETATM	639	H1	TIP	X5455	6.040	2.851	2.112	1.00	0.00	H
HETATM	640	H2	TIP	X5455	5.897	1.763	1.076	1.00	0.00	H
HETATM	641	OH2	TIP	X1534	-1.514	-3.434	3.741	1.00	0.00	O

```
HETATM  642  H1  TIP X1534      -1.467  -3.987   2.975  1.00  0.00           H
HETATM  643  H2  TIP X1534      -2.026  -3.935   4.371  1.00  0.00           H
HETATM  644  OH2 TIP X1287       0.122  -3.898   5.780  1.00  0.00           O
HETATM  645  H1  TIP X1287      -0.299  -3.675   4.946  1.00  0.00           H
HETATM  646  H2  TIP X1287      -0.562  -4.333   6.269  1.00  0.00           H
HETATM  647  OH2 TIP X1548      -2.455   0.557   0.834  1.00  0.00           O
HETATM  648  H1  TIP X1548      -1.654   0.889   1.245  1.00  0.00           H
HETATM  649  H2  TIP X1548      -3.100   1.250   0.959  1.00  0.00           H
HETATM  650  OH2 TIP X1612       5.920  -0.149   6.342  1.00  0.00           O
HETATM  651  H1  TIP X1612       5.934  -0.531   5.471  1.00  0.00           H
HETATM  652  H2  TIP X1612       6.757   0.342   6.394  1.00  0.00           H
HETATM  653  OH2 TIP X2089       4.627  -2.591   2.859  1.00  0.00           O
HETATM  654  H1  TIP X2089       5.544  -2.524   2.610  1.00  0.00           H
HETATM  655  H2  TIP X2089       4.463  -3.526   2.947  1.00  0.00           H
HETATM  656  OH2 TIP X3523       4.000  -5.150   3.337  1.00  0.00           O
HETATM  657  H1  TIP X3523       3.806  -5.071   4.271  1.00  0.00           H
HETATM  658  H2  TIP X3523       3.178  -5.452   2.944  1.00  0.00           H
END
```

Dopamine (Cluster structure)

```
ATOM      1  N   GLY A 113       1.776  10.416  13.755  1.00  0.00           N
ATOM      2  CA  GLY A 113       2.747   9.986  12.706  1.00  0.00           C
ATOM      3  C   GLY A 113       2.918   8.469  12.623  1.00  0.00           C
ATOM      4  O   GLY A 113       2.919   7.940  11.550  1.00  0.00           O
ATOM      5  H1  GLY A 113       1.032  10.819  13.244  1.00  0.00           H
ATOM      6  H2  GLY A 113       2.332  10.901  14.413  1.00  0.00           H
ATOM      7  HA2 GLY A 113       2.354  10.308  11.741  1.00  0.00           H
ATOM      8  HA3 GLY A 113       3.717  10.448  12.927  1.00  0.00           H
ATOM      9  N   ASP A 114       3.076   7.768  13.716  1.00  0.00           N
```

ATOM	10	CA	ASP	A	114	3.393	6.323	13.852	1.00	0.00	C
ATOM	11	CB	ASP	A	114	3.895	6.020	15.280	1.00	0.00	C
ATOM	12	CG	ASP	A	114	4.282	4.583	15.387	1.00	0.00	C
ATOM	13	OD1	ASP	A	114	4.731	3.929	14.440	1.00	0.00	O
ATOM	14	OD2	ASP	A	114	4.020	4.063	16.502	1.00	0.00	O1-
ATOM	15	C	ASP	A	114	2.253	5.413	13.298	1.00	0.00	C
ATOM	16	O	ASP	A	114	2.368	4.467	12.501	1.00	0.00	O
ATOM	17	H	ASP	A	114	2.978	8.149	14.635	1.00	0.00	H
ATOM	18	HA	ASP	A	114	4.235	6.037	13.243	1.00	0.00	H
ATOM	19	HB2	ASP	A	114	3.120	6.322	16.020	1.00	0.00	H
ATOM	20	HB3	ASP	A	114	4.831	6.572	15.519	1.00	0.00	H
ATOM	21	N	VAL	A	115	1.035	5.867	13.700	1.00	0.00	N
ATOM	22	CA	VAL	A	115	-0.156	5.231	13.211	1.00	0.00	C
ATOM	23	CB	VAL	A	115	-1.303	5.481	14.086	1.00	0.00	C
ATOM	24	CG1	VAL	A	115	-2.654	4.916	13.610	1.00	0.00	C
ATOM	25	CG2	VAL	A	115	-1.052	4.939	15.533	1.00	0.00	C
ATOM	26	C	VAL	A	115	-0.537	5.481	11.727	1.00	0.00	C
ATOM	27	O	VAL	A	115	-0.883	4.580	10.989	1.00	0.00	O
ATOM	28	H	VAL	A	115	0.945	6.689	14.264	1.00	0.00	H
ATOM	29	HA	VAL	A	115	0.015	4.157	13.173	1.00	0.00	H
ATOM	30	HB	VAL	A	115	-1.406	6.574	14.285	1.00	0.00	H
ATOM	31	HG11	VAL	A	115	-3.005	5.386	12.666	1.00	0.00	H
ATOM	32	HG12	VAL	A	115	-3.459	5.019	14.360	1.00	0.00	H
ATOM	33	HG13	VAL	A	115	-2.571	3.824	13.421	1.00	0.00	H
ATOM	34	HG21	VAL	A	115	-0.612	3.907	15.528	1.00	0.00	H
ATOM	35	HG22	VAL	A	115	-2.003	5.022	16.091	1.00	0.00	H
ATOM	36	HG23	VAL	A	115	-0.302	5.559	16.085	1.00	0.00	H
ATOM	37	N	GLY	A	116	-0.377	6.788	11.278	1.00	0.00	N
ATOM	38	CA	GLY	A	116	-0.560	7.244	9.907	1.00	0.00	C

ATOM	39	C	GLY	A	116	0.380	6.656	8.896	1.00	0.00	C
ATOM	40	O	GLY	A	116	0.010	6.178	7.833	1.00	0.00	O
ATOM	41	H	GLY	A	116	-0.111	7.481	11.963	1.00	0.00	H
ATOM	42	HA2	GLY	A	116	-1.550	6.901	9.608	1.00	0.00	H
ATOM	43	HA3	GLY	A	116	-0.463	8.353	9.892	1.00	0.00	H
ATOM	44	N	GLY	A	117	1.673	6.552	9.227	1.00	0.00	N
ATOM	45	CA	GLY	A	117	2.552	5.997	8.253	1.00	0.00	C
ATOM	46	C	GLY	A	117	2.484	4.459	8.146	1.00	0.00	C
ATOM	47	O	GLY	A	117	2.733	3.883	7.151	1.00	0.00	O
ATOM	48	H	GLY	A	117	2.011	6.854	10.129	1.00	0.00	H
ATOM	49	HA2	GLY	A	117	2.224	6.410	7.299	1.00	0.00	H
ATOM	50	HA3	GLY	A	117	3.597	6.273	8.488	1.00	0.00	H
ATOM	51	N	CYS	A	118	2.172	3.778	9.249	1.00	0.00	N
ATOM	52	CA	CYS	A	118	2.032	2.413	9.191	1.00	0.00	C
ATOM	53	CB	CYS	A	118	2.036	1.655	10.525	1.00	0.00	C
ATOM	54	SG	CYS	A	118	3.763	1.728	11.176	1.00	0.00	S
ATOM	55	C	CYS	A	118	0.764	2.029	8.546	1.00	0.00	C
ATOM	56	O	CYS	A	118	0.730	1.164	7.742	1.00	0.00	O
ATOM	57	H	CYS	A	118	2.025	4.228	10.133	1.00	0.00	H
ATOM	58	HA	CYS	A	118	2.823	1.921	8.643	1.00	0.00	H
ATOM	59	HB2	CYS	A	118	1.858	0.567	10.401	1.00	0.00	H
ATOM	60	HB3	CYS	A	118	1.343	2.204	11.193	1.00	0.00	H
ATOM	61	HG	CYS	A	118	3.672	2.905	11.801	1.00	0.00	H
ATOM	62	N	THR	A	119	-0.230	2.865	8.772	1.00	0.00	N
ATOM	63	CA	THR	A	119	-1.579	2.805	8.198	1.00	0.00	C
ATOM	64	CB	THR	A	119	-2.590	3.621	8.804	1.00	0.00	C
ATOM	65	OG1	THR	A	119	-2.866	3.112	10.099	1.00	0.00	O
ATOM	66	CG2	THR	A	119	-3.816	3.452	8.058	1.00	0.00	C
ATOM	67	C	THR	A	119	-1.610	3.035	6.722	1.00	0.00	C

ATOM	68	O	THR	A	119	-2.134	2.269	5.892	1.00	0.00	O
ATOM	69	H	THR	A	119	-0.092	3.592	9.436	1.00	0.00	H
ATOM	70	HA	THR	A	119	-1.900	1.766	8.228	1.00	0.00	H
ATOM	71	HB	THR	A	119	-2.192	4.649	8.926	1.00	0.00	H
ATOM	72	HG21	THR	A	119	-4.737	3.754	8.604	1.00	0.00	H
ATOM	73	HG22	THR	A	119	-3.920	2.392	7.724	1.00	0.00	H
ATOM	74	HG23	THR	A	119	-3.738	4.093	7.153	1.00	0.00	H
ATOM	75	HG1	THR	A	119	-2.115	3.497	10.563	1.00	0.00	H
ATOM	76	N	GLY	A	120	-0.910	4.060	6.259	1.00	0.00	N
ATOM	77	CA	GLY	A	120	-0.702	4.453	4.859	1.00	0.00	C
ATOM	78	C	GLY	A	120	0.033	3.366	4.041	1.00	0.00	C
ATOM	79	O	GLY	A	120	-0.335	3.164	2.903	1.00	0.00	O
ATOM	80	H	GLY	A	120	-0.465	4.643	6.954	1.00	0.00	H
ATOM	81	HA2	GLY	A	120	-1.686	4.600	4.414	1.00	0.00	H
ATOM	82	HA3	GLY	A	120	-0.109	5.372	4.829	1.00	0.00	H
ATOM	83	HC	GLY	A	120	0.849	2.804	4.470	1.00	0.00	H
ATOM	84	N	GLY	A	188	-7.424	0.468	23.117	1.00	0.00	N
ATOM	85	CA	GLY	A	188	-8.744	0.492	22.599	1.00	0.00	C
ATOM	86	C	GLY	A	188	-8.796	1.015	21.187	1.00	0.00	C
ATOM	87	O	GLY	A	188	-9.338	0.374	20.259	1.00	0.00	O
ATOM	88	H1	GLY	A	188	-7.196	-0.504	23.278	1.00	0.00	H
ATOM	89	H2	GLY	A	188	-7.406	1.122	23.888	1.00	0.00	H
ATOM	90	HA2	GLY	A	188	-9.104	-0.537	22.594	1.00	0.00	H
ATOM	91	HA3	GLY	A	188	-9.382	1.129	23.235	1.00	0.00	H
ATOM	92	N	PHE	A	189	-8.081	2.173	20.957	1.00	0.00	N
ATOM	93	CA	PHE	A	189	-7.876	2.866	19.711	1.00	0.00	C
ATOM	94	CB	PHE	A	189	-7.116	4.210	19.782	1.00	0.00	C
ATOM	95	CG	PHE	A	189	-6.757	4.671	18.321	1.00	0.00	C
ATOM	96	CD1	PHE	A	189	-7.653	5.363	17.584	1.00	0.00	C

ATOM	97	CE1	PHE	A	189	-7.297	5.722	16.300	1.00	0.00	C
ATOM	98	CZ	PHE	A	189	-5.998	5.485	15.847	1.00	0.00	C
ATOM	99	CD2	PHE	A	189	-5.450	4.336	17.924	1.00	0.00	C
ATOM	100	CE2	PHE	A	189	-5.034	4.787	16.656	1.00	0.00	C
ATOM	101	C	PHE	A	189	-7.266	1.897	18.684	1.00	0.00	C
ATOM	102	O	PHE	A	189	-7.714	1.787	17.569	1.00	0.00	O
ATOM	103	H	PHE	A	189	-7.726	2.609	21.781	1.00	0.00	H
ATOM	104	HA	PHE	A	189	-8.817	3.122	19.247	1.00	0.00	H
ATOM	105	HB2	PHE	A	189	-6.219	4.195	20.431	1.00	0.00	H
ATOM	106	HB3	PHE	A	189	-7.883	4.951	20.107	1.00	0.00	H
ATOM	107	HD1	PHE	A	189	-8.658	5.668	17.829	1.00	0.00	H
ATOM	108	HD2	PHE	A	189	-4.743	3.871	18.595	1.00	0.00	H
ATOM	109	HE1	PHE	A	189	-8.086	6.203	15.737	1.00	0.00	H
ATOM	110	HE2	PHE	A	189	-4.027	4.645	16.283	1.00	0.00	H
ATOM	111	HZ	PHE	A	189	-5.641	5.997	14.965	1.00	0.00	H
ATOM	112	N	VAL	A	190	-6.204	1.146	19.076	1.00	0.00	N
ATOM	113	CA	VAL	A	190	-5.608	0.128	18.253	1.00	0.00	C
ATOM	114	CB	VAL	A	190	-4.434	-0.501	19.051	1.00	0.00	C
ATOM	115	CG1	VAL	A	190	-3.674	-1.388	18.053	1.00	0.00	C
ATOM	116	CG2	VAL	A	190	-3.500	0.590	19.430	1.00	0.00	C
ATOM	117	C	VAL	A	190	-6.589	-0.919	17.750	1.00	0.00	C
ATOM	118	O	VAL	A	190	-6.636	-1.353	16.601	1.00	0.00	O
ATOM	119	H	VAL	A	190	-5.831	1.443	19.949	1.00	0.00	H
ATOM	120	HA	VAL	A	190	-5.260	0.660	17.369	1.00	0.00	H
ATOM	121	HB	VAL	A	190	-4.951	-0.933	19.926	1.00	0.00	H
ATOM	122	HG11	VAL	A	190	-3.456	-0.849	17.106	1.00	0.00	H
ATOM	123	HG12	VAL	A	190	-4.188	-2.298	17.702	1.00	0.00	H
ATOM	124	HG13	VAL	A	190	-2.745	-1.681	18.588	1.00	0.00	H
ATOM	125	HG21	VAL	A	190	-3.256	1.390	18.690	1.00	0.00	H

ATOM	126	HG22	VAL	A	190	-2.559	0.171	19.843	1.00	0.00	H
ATOM	127	HG23	VAL	A	190	-3.847	1.208	20.281	1.00	0.00	H
ATOM	128	N	GLY	A	191	-7.319	-1.484	18.758	1.00	0.00	N
ATOM	129	CA	GLY	A	191	-8.333	-2.482	18.423	1.00	0.00	C
ATOM	130	C	GLY	A	191	-9.465	-2.192	17.473	1.00	0.00	C
ATOM	131	O	GLY	A	191	-9.757	-2.992	16.580	1.00	0.00	O
ATOM	132	H	GLY	A	191	-7.160	-1.216	19.719	1.00	0.00	H
ATOM	133	HA2	GLY	A	191	-7.754	-3.192	17.833	1.00	0.00	H
ATOM	134	HA3	GLY	A	191	-8.782	-2.850	19.407	1.00	0.00	H
ATOM	135	N	GLY	A	192	-10.127	-1.060	17.638	1.00	0.00	N
ATOM	136	CA	GLY	A	192	-11.221	-0.724	16.797	1.00	0.00	C
ATOM	137	C	GLY	A	192	-10.654	-0.189	15.436	1.00	0.00	C
ATOM	138	O	GLY	A	192	-11.184	-0.556	14.384	1.00	0.00	O
ATOM	139	H	GLY	A	192	-9.851	-0.425	18.373	1.00	0.00	H
ATOM	140	HA2	GLY	A	192	-11.837	-1.613	16.661	1.00	0.00	H
ATOM	141	HA3	GLY	A	192	-11.820	0.063	17.233	1.00	0.00	H
ATOM	142	N	SER	A	193	-9.623	0.599	15.403	1.00	0.00	N
ATOM	143	CA	SER	A	193	-8.908	1.041	14.171	1.00	0.00	C
ATOM	144	CB	SER	A	193	-7.774	2.110	14.515	1.00	0.00	C
ATOM	145	OG	SER	A	193	-6.606	1.571	15.129	1.00	0.00	O
ATOM	146	C	SER	A	193	-8.259	0.004	13.179	1.00	0.00	C
ATOM	147	O	SER	A	193	-8.376	0.079	11.980	1.00	0.00	O
ATOM	148	H	SER	A	193	-9.290	0.868	16.307	1.00	0.00	H
ATOM	149	HA	SER	A	193	-9.676	1.550	13.577	1.00	0.00	H
ATOM	150	HB2	SER	A	193	-8.278	2.781	15.230	1.00	0.00	H
ATOM	151	HB3	SER	A	193	-7.536	2.710	13.605	1.00	0.00	H
ATOM	152	HG	SER	A	193	-6.785	1.592	16.066	1.00	0.00	H
ATOM	153	N	SER	A	194	-7.549	-1.021	13.680	1.00	0.00	N
ATOM	154	CA	SER	A	194	-6.884	-2.027	12.897	1.00	0.00	C

ATOM	155	CB	SER	A	194	-5.901	-2.812	13.750	1.00	0.00	C
ATOM	156	OG	SER	A	194	-6.492	-3.525	14.810	1.00	0.00	O
ATOM	157	C	SER	A	194	-7.819	-2.880	12.104	1.00	0.00	C
ATOM	158	O	SER	A	194	-7.430	-3.475	11.132	1.00	0.00	O
ATOM	159	H	SER	A	194	-7.369	-1.037	14.666	1.00	0.00	H
ATOM	160	HA	SER	A	194	-6.221	-1.473	12.238	1.00	0.00	H
ATOM	161	HB2	SER	A	194	-5.138	-2.103	14.107	1.00	0.00	H
ATOM	162	HB3	SER	A	194	-5.360	-3.616	13.184	1.00	0.00	H
ATOM	163	HG	SER	A	194	-6.545	-2.828	15.461	1.00	0.00	H
ATOM	164	N	GLY	A	195	-9.144	-2.885	12.329	1.00	0.00	N
ATOM	165	CA	GLY	A	195	-10.136	-3.487	11.380	1.00	0.00	C
ATOM	166	C	GLY	A	195	-10.150	-2.667	10.083	1.00	0.00	C
ATOM	167	O	GLY	A	195	-10.168	-3.180	8.939	1.00	0.00	O
ATOM	168	H	GLY	A	195	-9.488	-2.462	13.179	1.00	0.00	H
ATOM	169	HA2	GLY	A	195	-9.866	-4.518	11.153	1.00	0.00	H
ATOM	170	HA3	GLY	A	195	-11.112	-3.457	11.837	1.00	0.00	H
ATOM	171	N	GLY	A	196	-10.103	-1.364	10.273	1.00	0.00	N
ATOM	172	CA	GLY	A	196	-10.059	-0.560	9.048	1.00	0.00	C
ATOM	173	C	GLY	A	196	-8.635	-0.429	8.387	1.00	0.00	C
ATOM	174	O	GLY	A	196	-8.463	-0.553	7.225	1.00	0.00	O
ATOM	175	H	GLY	A	196	-10.098	-0.950	11.194	1.00	0.00	H
ATOM	176	HA2	GLY	A	196	-10.692	-1.091	8.337	1.00	0.00	H
ATOM	177	HA3	GLY	A	196	-10.420	0.459	9.266	1.00	0.00	H
ATOM	178	N	SER	A	197	-7.531	-0.307	9.173	1.00	0.00	N
ATOM	179	CA	SER	A	197	-6.181	-0.125	8.689	1.00	0.00	C
ATOM	180	CB	SER	A	197	-5.301	0.612	9.738	1.00	0.00	C
ATOM	181	OG	SER	A	197	-5.969	1.818	10.169	1.00	0.00	O
ATOM	182	C	SER	A	197	-5.365	-1.346	8.361	1.00	0.00	C
ATOM	183	O	SER	A	197	-4.245	-1.243	7.897	1.00	0.00	O

ATOM	184	H	SER	A	197	-7.540	-0.302	10.175	1.00	0.00	H
ATOM	185	HA	SER	A	197	-6.173	0.487	7.794	1.00	0.00	H
ATOM	186	HB2	SER	A	197	-4.273	0.841	9.384	1.00	0.00	H
ATOM	187	HB3	SER	A	197	-5.241	0.106	10.738	1.00	0.00	H
ATOM	188	HG	SER	A	197	-5.322	2.498	10.345	1.00	0.00	H
ATOM	189	N	GLY	A	198	-5.872	-2.518	8.716	1.00	0.00	N
ATOM	190	CA	GLY	A	198	-5.128	-3.713	8.486	1.00	0.00	C
ATOM	191	C	GLY	A	198	-6.138	-4.754	7.922	1.00	0.00	C
ATOM	192	O	GLY	A	198	-6.007	-5.243	6.860	1.00	0.00	O
ATOM	193	H	GLY	A	198	-6.783	-2.565	9.149	1.00	0.00	H
ATOM	194	HA2	GLY	A	198	-4.299	-3.514	7.807	1.00	0.00	H
ATOM	195	HA3	GLY	A	198	-4.728	-4.100	9.403	1.00	0.00	H
ATOM	196	HC	GLY	A	198	-6.988	-5.037	8.526	1.00	0.00	H
ATOM	197	N	GLY	A	388	0.382	-10.807	14.973	1.00	0.00	N
ATOM	198	CA	GLY	A	388	0.915	-10.911	16.372	1.00	0.00	C
ATOM	199	C	GLY	A	388	0.820	-9.656	17.173	1.00	0.00	C
ATOM	200	O	GLY	A	388	0.457	-9.802	18.336	1.00	0.00	O
ATOM	201	H1	GLY	A	388	-0.407	-11.397	14.968	1.00	0.00	H
ATOM	202	H2	GLY	A	388	1.177	-10.915	14.400	1.00	0.00	H
ATOM	203	HA2	GLY	A	388	0.294	-11.628	16.909	1.00	0.00	H
ATOM	204	HA3	GLY	A	388	1.971	-11.235	16.298	1.00	0.00	H
ATOM	205	N	PHE	A	389	1.120	-8.420	16.710	1.00	0.00	N
ATOM	206	CA	PHE	A	389	0.838	-7.123	17.324	1.00	0.00	C
ATOM	207	CB	PHE	A	389	1.376	-6.022	16.423	1.00	0.00	C
ATOM	208	CG	PHE	A	389	1.416	-4.639	16.989	1.00	0.00	C
ATOM	209	CD1	PHE	A	389	2.398	-4.228	17.937	1.00	0.00	C
ATOM	210	CE1	PHE	A	389	2.459	-2.929	18.438	1.00	0.00	C
ATOM	211	CZ	PHE	A	389	1.506	-1.947	18.045	1.00	0.00	C
ATOM	212	CD2	PHE	A	389	0.505	-3.610	16.554	1.00	0.00	C

```
ATOM    213  CE2 PHE A 389       0.514  -2.327  17.125  1.00  0.00           C
ATOM    214  C   PHE A 389      -0.618  -6.865  17.622  1.00  0.00           C
ATOM    215  O   PHE A 389      -0.998  -6.355  18.679  1.00  0.00           O
ATOM    216  H   PHE A 389       1.388  -8.473  15.747  1.00  0.00           H
ATOM    217  HA  PHE A 389       1.471  -7.056  18.205  1.00  0.00           H
ATOM    218  HB2 PHE A 389       0.774  -6.012  15.499  1.00  0.00           H
ATOM    219  HB3 PHE A 389       2.441  -6.246  16.196  1.00  0.00           H
ATOM    220  HD1 PHE A 389       3.142  -4.955  18.222  1.00  0.00           H
ATOM    221  HD2 PHE A 389      -0.333  -3.864  15.911  1.00  0.00           H
ATOM    222  HE1 PHE A 389       3.337  -2.678  19.023  1.00  0.00           H
ATOM    223  HE2 PHE A 389      -0.109  -1.524  16.768  1.00  0.00           H
ATOM    224  HZ  PHE A 389       1.568  -0.990  18.515  1.00  0.00           H
ATOM    225  N   PHE A 390      -1.548  -7.099  16.615  1.00  0.00           N
ATOM    226  CA  PHE A 390      -2.992  -6.677  16.774  1.00  0.00           C
ATOM    227  CB  PHE A 390      -3.795  -6.441  15.393  1.00  0.00           C
ATOM    228  CG  PHE A 390      -3.117  -5.308  14.523  1.00  0.00           C
ATOM    229  CD1 PHE A 390      -2.848  -4.054  15.120  1.00  0.00           C
ATOM    230  CE1 PHE A 390      -2.312  -2.975  14.350  1.00  0.00           C
ATOM    231  CZ  PHE A 390      -1.956  -3.230  12.999  1.00  0.00           C
ATOM    232  CD2 PHE A 390      -2.642  -5.519  13.252  1.00  0.00           C
ATOM    233  CE2 PHE A 390      -2.116  -4.522  12.446  1.00  0.00           C
ATOM    234  C   PHE A 390      -3.828  -7.703  17.575  1.00  0.00           C
ATOM    235  O   PHE A 390      -4.734  -7.468  18.376  1.00  0.00           O
ATOM    236  H   PHE A 390      -1.323  -7.634  15.808  1.00  0.00           H
ATOM    237  HA  PHE A 390      -3.072  -5.755  17.333  1.00  0.00           H
ATOM    238  HB2 PHE A 390      -4.857  -6.113  15.495  1.00  0.00           H
ATOM    239  HB3 PHE A 390      -3.762  -7.329  14.721  1.00  0.00           H
ATOM    240  HD1 PHE A 390      -3.227  -3.859  16.104  1.00  0.00           H
ATOM    241  HD2 PHE A 390      -2.797  -6.556  12.965  1.00  0.00           H
```

ATOM	242	HE1	PHE	A	390	-2.129	-1.984	14.720	1.00	0.00	H
ATOM	243	HE2	PHE	A	390	-1.816	-4.699	11.430	1.00	0.00	H
ATOM	244	HZ	PHE	A	390	-1.531	-2.529	12.288	1.00	0.00	H
ATOM	245	N	GLY	A	391	-3.527	-9.010	17.503	1.00	0.00	N
ATOM	246	CA	GLY	A	391	-3.958	-10.025	18.522	1.00	0.00	C
ATOM	247	C	GLY	A	391	-3.545	-9.648	19.932	1.00	0.00	C
ATOM	248	O	GLY	A	391	-4.364	-9.594	20.894	1.00	0.00	O
ATOM	249	H	GLY	A	391	-2.978	-9.329	16.717	1.00	0.00	H
ATOM	250	HA2	GLY	A	391	-5.047	-10.077	18.519	1.00	0.00	H
ATOM	251	HA3	GLY	A	391	-3.510	-10.986	18.250	1.00	0.00	H
ATOM	252	HC	GLY	A	391	-2.508	-9.416	20.122	1.00	0.00	H
ATOM	253	N	GLY	A	411	7.581	-7.030	17.762	1.00	0.00	N
ATOM	254	CA	GLY	A	411	6.387	-6.944	16.912	1.00	0.00	C
ATOM	255	C	GLY	A	411	6.343	-5.634	16.158	1.00	0.00	C
ATOM	256	O	GLY	A	411	5.785	-5.594	15.034	1.00	0.00	O
ATOM	257	H1	GLY	A	411	8.116	-7.789	17.381	1.00	0.00	H
ATOM	258	H2	GLY	A	411	7.229	-7.059	18.701	1.00	0.00	H
ATOM	259	HA2	GLY	A	411	6.419	-7.753	16.182	1.00	0.00	H
ATOM	260	HA3	GLY	A	411	5.499	-7.030	17.543	1.00	0.00	H
ATOM	261	N	THR	A	412	6.861	-4.548	16.800	1.00	0.00	N
ATOM	262	CA	THR	A	412	7.054	-3.121	16.165	1.00	0.00	C
ATOM	263	CB	THR	A	412	7.254	-1.973	17.197	1.00	0.00	C
ATOM	264	OG1	THR	A	412	6.041	-1.664	17.858	1.00	0.00	O
ATOM	265	CG2	THR	A	412	7.777	-0.626	16.573	1.00	0.00	C
ATOM	266	C	THR	A	412	8.084	-3.127	15.163	1.00	0.00	C
ATOM	267	O	THR	A	412	7.905	-2.564	14.097	1.00	0.00	O
ATOM	268	H	THR	A	412	7.203	-4.738	17.719	1.00	0.00	H
ATOM	269	HA	THR	A	412	6.143	-2.774	15.707	1.00	0.00	H
ATOM	270	HB	THR	A	412	7.949	-2.301	17.990	1.00	0.00	H

ATOM	271	HG21	THR	A	412	8.689	-0.870	15.983	1.00	0.00	H
ATOM	272	HG22	THR	A	412	8.041	0.052	17.422	1.00	0.00	H
ATOM	273	HG23	THR	A	412	6.929	-0.225	15.993	1.00	0.00	H
ATOM	274	HG1	THR	A	412	6.194	-2.006	18.741	1.00	0.00	H
ATOM	275	N	GLY	A	413	9.317	-3.721	15.511	1.00	0.00	N
ATOM	276	CA	GLY	A	413	10.355	-3.664	14.576	1.00	0.00	C
ATOM	277	C	GLY	A	413	10.160	-4.526	13.278	1.00	0.00	C
ATOM	278	O	GLY	A	413	10.442	-4.086	12.167	1.00	0.00	O
ATOM	279	H	GLY	A	413	9.445	-4.172	16.406	1.00	0.00	H
ATOM	280	HA2	GLY	A	413	10.360	-2.616	14.277	1.00	0.00	H
ATOM	281	HA3	GLY	A	413	11.316	-3.995	15.034	1.00	0.00	H
ATOM	282	HC	GLY	A	413	9.769	-5.529	13.365	1.00	0.00	H
ATOM	283	N	GLY	A	415	7.176	-5.177	11.913	1.00	0.00	N
ATOM	284	CA	GLY	A	415	6.261	-4.374	11.189	1.00	0.00	C
ATOM	285	C	GLY	A	415	6.947	-3.306	10.403	1.00	0.00	C
ATOM	286	O	GLY	A	415	6.861	-3.325	9.150	1.00	0.00	O
ATOM	287	H1	GLY	A	415	7.908	-5.692	11.446	1.00	0.00	H
ATOM	288	H2	GLY	A	415	7.067	-5.212	12.904	1.00	0.00	H
ATOM	289	HA2	GLY	A	415	5.686	-3.890	11.953	1.00	0.00	H
ATOM	290	HA3	GLY	A	415	5.613	-4.920	10.526	1.00	0.00	H
ATOM	291	N	TYR	A	416	7.815	-2.383	11.020	1.00	0.00	N
ATOM	292	CA	TYR	A	416	8.539	-1.310	10.335	1.00	0.00	C
ATOM	293	CB	TYR	A	416	9.463	-0.521	11.221	1.00	0.00	C
ATOM	294	CG	TYR	A	416	8.913	0.486	12.227	1.00	0.00	C
ATOM	295	CD1	TYR	A	416	7.912	1.395	11.787	1.00	0.00	C
ATOM	296	CE1	TYR	A	416	7.406	2.404	12.643	1.00	0.00	C
ATOM	297	CZ	TYR	A	416	7.932	2.500	13.860	1.00	0.00	C
ATOM	298	OH	TYR	A	416	7.394	3.464	14.808	1.00	0.00	O
ATOM	299	CD2	TYR	A	416	9.418	0.655	13.499	1.00	0.00	C

ATOM	300	CE2	TYR	A	416	8.972	1.615	14.370	1.00	0.00	C
ATOM	301	C	TYR	A	416	9.530	-1.849	9.292	1.00	0.00	C
ATOM	302	O	TYR	A	416	9.750	-1.245	8.261	1.00	0.00	O
ATOM	303	H	TYR	A	416	7.816	-2.479	12.007	1.00	0.00	H
ATOM	304	HA	TYR	A	416	7.911	-0.661	9.726	1.00	0.00	H
ATOM	305	HB2	TYR	A	416	10.075	0.186	10.623	1.00	0.00	H
ATOM	306	HB3	TYR	A	416	10.101	-1.221	11.803	1.00	0.00	H
ATOM	307	HD1	TYR	A	416	7.340	1.216	10.884	1.00	0.00	H
ATOM	308	HD2	TYR	A	416	10.182	-0.041	13.793	1.00	0.00	H
ATOM	309	HE1	TYR	A	416	6.570	3.055	12.430	1.00	0.00	H
ATOM	310	HE2	TYR	A	416	9.413	1.698	15.345	1.00	0.00	H
ATOM	311	HH	TYR	A	416	6.530	3.687	14.458	1.00	0.00	H
ATOM	312	N	GLY	A	417	10.236	-2.961	9.487	1.00	0.00	N
ATOM	313	CA	GLY	A	417	11.075	-3.667	8.510	1.00	0.00	C
ATOM	314	C	GLY	A	417	10.277	-4.152	7.319	1.00	0.00	C
ATOM	315	O	GLY	A	417	10.640	-4.025	6.167	1.00	0.00	O
ATOM	316	H	GLY	A	417	10.189	-3.365	10.412	1.00	0.00	H
ATOM	317	HA2	GLY	A	417	11.826	-2.969	8.140	1.00	0.00	H
ATOM	318	HA3	GLY	A	417	11.545	-4.524	9.005	1.00	0.00	H
ATOM	319	HC	GLY	A	417	9.331	-4.639	7.504	1.00	0.00	H
TER	320		GLY	A	417						
HETATM	321	C1	DOP	B	444	1.154	0.789	15.694	1.00	0.00	C
HETATM	322	C2	DOP	B	444	-0.293	0.754	15.027	1.00	0.00	C
HETATM	323	C3	DOP	B	444	-1.370	1.131	15.846	1.00	0.00	C
HETATM	324	C4	DOP	B	444	-2.649	1.374	15.307	1.00	0.00	C
HETATM	325	C5	DOP	B	444	-2.863	1.070	13.957	1.00	0.00	C
HETATM	326	C6	DOP	B	444	-0.506	0.737	13.611	1.00	0.00	C
HETATM	327	C7	DOP	B	444	-1.806	0.762	13.123	1.00	0.00	C
HETATM	328	O1	DOP	B	444	-4.224	1.187	13.503	1.00	0.00	O

HETATM	329	O2	DOP	B 444	-1.899	0.475	11.725	1.00	0.00	O
HETATM	330	C8	DOP	B 444	2.040	1.807	14.924	1.00	0.00	C
HETATM	331	N	DOP	B 444	3.504	1.659	15.202	1.00	0.00	N1+
HETATM	332	H1	DOP	B 444	-4.769	1.391	14.273	1.00	0.00	H
HETATM	333	H2	DOP	B 444	-2.692	0.898	11.366	1.00	0.00	H
HETATM	334	H3	DOP	B 444	4.027	1.062	14.543	1.00	0.00	H
HETATM	335	H4	DOP	B 444	3.665	1.363	16.193	1.00	0.00	H
HETATM	336	H5	DOP	B 444	1.485	-0.268	15.608	1.00	0.00	H
HETATM	337	H6	DOP	B 444	1.183	1.087	16.760	1.00	0.00	H
HETATM	338	H7	DOP	B 444	-1.220	1.289	16.902	1.00	0.00	H
HETATM	339	H8	DOP	B 444	-3.470	1.777	15.870	1.00	0.00	H
HETATM	340	H9	DOP	B 444	0.328	0.668	12.930	1.00	0.00	H
HETATM	341	H10	DOP	B 444	1.872	2.830	15.326	1.00	0.00	H
HETATM	342	H11	DOP	B 444	1.981	1.717	13.836	1.00	0.00	H
HETATM	343	H12	DOP	B 444	3.978	2.572	15.061	1.00	0.00	H
HETATM	344	OH2	TIP	X5369	3.057	-2.430	14.947	1.00	0.00	O
HETATM	345	H1	TIP	X5369	3.204	-3.292	14.576	1.00	0.00	H
HETATM	346	H2	TIP	X5369	2.325	-2.556	15.560	1.00	0.00	H
HETATM	347	OH2	TIP	X9400	6.141	2.730	17.448	1.00	0.00	O
HETATM	348	H1	TIP	X9400	5.336	3.210	17.226	1.00	0.00	H
HETATM	349	H2	TIP	X9400	6.823	3.088	16.871	1.00	0.00	H
HETATM	350	OH2	TIP	X4965	4.406	0.621	17.660	1.00	0.00	O
HETATM	351	H1	TIP	X4965	5.022	1.314	17.895	1.00	0.00	H
HETATM	352	H2	TIP	X4965	4.927	-0.165	17.583	1.00	0.00	H
HETATM	353	OH2	TIP	X 290	2.004	3.615	18.310	1.00	0.00	O
HETATM	354	H1	TIP	X 290	2.589	3.824	17.579	1.00	0.00	H
HETATM	355	H2	TIP	X 290	2.589	3.454	19.052	1.00	0.00	H
HETATM	356	OH2	TIP	X 785	4.706	-0.540	14.034	1.00	0.00	O
HETATM	357	H1	TIP	X 785	5.495	-0.856	13.596	1.00	0.00	H

```
HETATM  358  H2  TIP X 785       4.289  -1.342  14.370  1.00  0.00           H
END
```

Rotigotine (Cluster structure)

```
ATOM      1  CA  LEU A 113      -1.294  -5.714  17.624  1.00  0.00           C
ATOM      2  C   LEU A 113      -1.436  -4.231  17.324  1.00  0.00           C
ATOM      3  O   LEU A 113      -1.468  -3.803  16.155  1.00  0.00           O
ATOM      4  HA1 LEU A 113      -0.728  -6.026  16.757  1.00  0.00           H
ATOM      5  HA2 LEU A 113      -2.263  -6.211  17.676  1.00  0.00           H
ATOM      6  HA3 LEU A 113      -0.746  -5.889  18.550  1.00  0.00           H
ATOM      7  N   ASP A 114      -1.488  -3.332  18.359  1.00  0.00           N
ATOM      8  CA  ASP A 114      -1.563  -1.920  18.323  1.00  0.00           C
ATOM      9  CB  ASP A 114      -1.703  -1.380  19.787  1.00  0.00           C
ATOM     10  CG  ASP A 114      -2.119   0.109  19.898  1.00  0.00           C
ATOM     11  OD1 ASP A 114      -2.703   0.717  18.939  1.00  0.00           O
ATOM     12  OD2 ASP A 114      -1.865   0.724  20.959  1.00  0.00           O1-
ATOM     13  C   ASP A 114      -0.319  -1.269  17.658  1.00  0.00           C
ATOM     14  O   ASP A 114      -0.440  -0.453  16.702  1.00  0.00           O
ATOM     15  H   ASP A 114      -1.382  -3.764  19.246  1.00  0.00           H
ATOM     16  HA  ASP A 114      -2.434  -1.597  17.790  1.00  0.00           H
ATOM     17  HB2 ASP A 114      -2.554  -1.857  20.307  1.00  0.00           H
ATOM     18  HB3 ASP A 114      -0.772  -1.589  20.344  1.00  0.00           H
ATOM     19  N   VAL A 115       0.914  -1.526  18.053  1.00  0.00           N
ATOM     20  CA  VAL A 115       2.115  -1.236  17.403  1.00  0.00           C
ATOM     21  CB  VAL A 115       3.352  -1.622  18.212  1.00  0.00           C
ATOM     22  CG1 VAL A 115       4.546  -1.078  17.432  1.00  0.00           C
ATOM     23  CG2 VAL A 115       3.363  -0.961  19.618  1.00  0.00           C
ATOM     24  C   VAL A 115       2.124  -1.867  15.992  1.00  0.00           C
ATOM     25  O   VAL A 115       2.620  -1.309  14.990  1.00  0.00           O
```

ATOM	26	H	VAL	A	115	1.050	-1.968	18.942	1.00	0.00	H
ATOM	27	HA	VAL	A	115	2.187	-0.182	17.221	1.00	0.00	H
ATOM	28	HB	VAL	A	115	3.380	-2.724	18.281	1.00	0.00	H
ATOM	29	HG11	VAL	A	115	4.361	-0.050	17.059	1.00	0.00	H
ATOM	30	HG12	VAL	A	115	4.760	-1.714	16.540	1.00	0.00	H
ATOM	31	HG13	VAL	A	115	5.510	-1.085	17.988	1.00	0.00	H
ATOM	32	HG21	VAL	A	115	2.819	-1.601	20.349	1.00	0.00	H
ATOM	33	HG22	VAL	A	115	2.945	0.081	19.515	1.00	0.00	H
ATOM	34	HG23	VAL	A	115	4.411	-0.836	19.983	1.00	0.00	H
ATOM	35	N	MET	A	116	1.712	-3.157	15.863	1.00	0.00	N
ATOM	36	CA	MET	A	116	1.838	-3.881	14.589	1.00	0.00	C
ATOM	37	C	MET	A	116	0.997	-3.236	13.521	1.00	0.00	C
ATOM	38	O	MET	A	116	1.591	-3.034	12.443	1.00	0.00	O
ATOM	39	H	MET	A	116	1.597	-3.823	16.592	1.00	0.00	H
ATOM	40	HA2	MET	A	116	2.852	-3.963	14.197	1.00	0.00	H
ATOM	41	HA3	MET	A	116	1.498	-4.888	14.831	1.00	0.00	H
ATOM	42	N	MET	A	117	-0.263	-2.987	13.724	1.00	0.00	N
ATOM	43	CA	MET	A	117	-1.147	-2.505	12.710	1.00	0.00	C
ATOM	44	C	MET	A	117	-0.798	-1.081	12.262	1.00	0.00	C
ATOM	45	O	MET	A	117	-0.812	-0.832	11.050	1.00	0.00	O
ATOM	46	H	MET	A	117	-0.665	-3.159	14.617	1.00	0.00	H
ATOM	47	HA2	MET	A	117	-0.934	-3.098	11.832	1.00	0.00	H
ATOM	48	HA3	MET	A	117	-2.166	-2.547	13.096	1.00	0.00	H
ATOM	49	N	CYS	A	118	-0.388	-0.136	13.173	1.00	0.00	N
ATOM	50	CA	CYS	A	118	0.079	1.183	12.903	1.00	0.00	C
ATOM	51	CB	CYS	A	118	0.391	1.857	14.219	1.00	0.00	C
ATOM	52	SG	CYS	A	118	0.720	3.676	14.165	1.00	0.00	S
ATOM	53	C	CYS	A	118	1.288	1.183	12.075	1.00	0.00	C
ATOM	54	O	CYS	A	118	1.435	1.790	11.032	1.00	0.00	O

ATOM	55	H	CYS	A	118	-0.221	-0.462	14.095	1.00	0.00	H
ATOM	56	HA	CYS	A	118	-0.680	1.784	12.409	1.00	0.00	H
ATOM	57	HB2	CYS	A	118	-0.498	1.728	14.888	1.00	0.00	H
ATOM	58	HB3	CYS	A	118	1.149	1.311	14.806	1.00	0.00	H
ATOM	59	HG	CYS	A	118	-0.513	4.085	13.898	1.00	0.00	H
ATOM	60	N	THR	A	119	2.325	0.383	12.481	1.00	0.00	N
ATOM	61	CA	THR	A	119	3.582	0.228	11.768	1.00	0.00	C
ATOM	62	CB	THR	A	119	4.545	-0.706	12.416	1.00	0.00	C
ATOM	63	OG1	THR	A	119	4.716	-0.176	13.702	1.00	0.00	O
ATOM	64	CG2	THR	A	119	5.923	-0.693	11.749	1.00	0.00	C
ATOM	65	C	THR	A	119	3.401	-0.318	10.373	1.00	0.00	C
ATOM	66	O	THR	A	119	3.957	0.088	9.375	1.00	0.00	O
ATOM	67	H	THR	A	119	2.211	-0.035	13.374	1.00	0.00	H
ATOM	68	HA	THR	A	119	4.014	1.192	11.553	1.00	0.00	H
ATOM	69	HB	THR	A	119	4.174	-1.741	12.501	1.00	0.00	H
ATOM	70	HG21	THR	A	119	6.500	0.249	11.934	1.00	0.00	H
ATOM	71	HG22	THR	A	119	5.814	-0.731	10.641	1.00	0.00	H
ATOM	72	HG23	THR	A	119	6.510	-1.568	12.092	1.00	0.00	H
ATOM	73	HG1	THR	A	119	3.968	-0.430	14.230	1.00	0.00	H
ATOM	74	N	ALA	A	120	2.532	-1.329	10.247	1.00	0.00	N
ATOM	75	CA	ALA	A	120	2.092	-1.805	8.960	1.00	0.00	C
ATOM	76	H	ALA	A	120	2.159	-1.774	11.071	1.00	0.00	H
ATOM	77	HA1	ALA	A	120	2.924	-2.177	8.378	1.00	0.00	H
ATOM	78	HA2	ALA	A	120	1.618	-0.985	8.420	1.00	0.00	H
ATOM	79	HA3	ALA	A	120	1.371	-2.609	9.105	1.00	0.00	H
ATOM	80	CA	CYS	A	182	-3.181	0.445	30.359	1.00	0.00	C
ATOM	81	C	CYS	A	182	-2.283	0.869	29.108	1.00	0.00	C
ATOM	82	O	CYS	A	182	-2.751	0.770	27.966	1.00	0.00	O
ATOM	83	HA1	CYS	A	182	-3.441	-0.602	30.305	1.00	0.00	H

ATOM	84	HA2	CYS	A	182	-4.094	1.041	30.362	1.00	0.00	H
ATOM	85	HA3	CYS	A	182	-2.624	0.628	31.278	1.00	0.00	H
ATOM	86	N	ILE	A	183	-1.029	1.363	29.257	1.00	0.00	N
ATOM	87	CA	ILE	A	183	-0.103	1.775	28.190	1.00	0.00	C
ATOM	88	CB	ILE	A	183	0.183	3.269	28.125	1.00	0.00	C
ATOM	89	CG2	ILE	A	183	-1.127	3.937	27.828	1.00	0.00	C
ATOM	90	CG1	ILE	A	183	0.552	3.704	29.539	1.00	0.00	C
ATOM	91	CD1	ILE	A	183	2.081	3.787	29.732	1.00	0.00	C
ATOM	92	C	ILE	A	183	1.178	0.962	28.206	1.00	0.00	C
ATOM	93	O	ILE	A	183	1.430	0.146	29.103	1.00	0.00	O
ATOM	94	H	ILE	A	183	-0.767	1.577	30.208	1.00	0.00	H
ATOM	95	HA	ILE	A	183	-0.476	1.440	27.236	1.00	0.00	H
ATOM	96	HB	ILE	A	183	0.984	3.678	27.493	1.00	0.00	H
ATOM	97	HG12	ILE	A	183	0.062	4.696	29.564	1.00	0.00	H
ATOM	98	HG13	ILE	A	183	0.048	3.256	30.402	1.00	0.00	H
ATOM	99	HG21	ILE	A	183	-1.509	3.636	26.828	1.00	0.00	H
ATOM	100	HG22	ILE	A	183	-1.065	5.050	27.768	1.00	0.00	H
ATOM	101	HG23	ILE	A	183	-1.888	3.646	28.573	1.00	0.00	H
ATOM	102	HD11	ILE	A	183	2.526	4.513	29.028	1.00	0.00	H
ATOM	103	HD12	ILE	A	183	2.312	4.087	30.781	1.00	0.00	H
ATOM	104	HD13	ILE	A	183	2.501	2.766	29.641	1.00	0.00	H
ATOM	105	N	ILE	A	184	2.013	1.188	27.106	1.00	0.00	N
ATOM	106	CA	ILE	A	184	3.359	0.761	27.063	1.00	0.00	C
ATOM	107	H	ILE	A	184	1.787	1.748	26.320	1.00	0.00	H
ATOM	108	HA1	ILE	A	184	3.435	-0.269	27.415	1.00	0.00	H
ATOM	109	HA2	ILE	A	184	3.963	1.406	27.701	1.00	0.00	H
ATOM	110	HA3	ILE	A	184	3.724	0.820	26.038	1.00	0.00	H
ATOM	111	CA	PHE	A	189	11.094	1.483	23.737	1.00	0.00	C
ATOM	112	C	PHE	A	189	10.152	2.402	22.929	1.00	0.00	C

ATOM	113	O	PHE	A	189	10.025	2.329	21.724	1.00	0.00	O
ATOM	114	HA1	PHE	A	189	11.787	1.026	23.055	1.00	0.00	H
ATOM	115	HA2	PHE	A	189	10.512	0.712	24.243	1.00	0.00	H
ATOM	116	HA3	PHE	A	189	11.638	2.071	24.476	1.00	0.00	H
ATOM	117	N	VAL	A	190	9.434	3.343	23.597	1.00	0.00	N
ATOM	118	CA	VAL	A	190	8.511	4.275	22.939	1.00	0.00	C
ATOM	119	CB	VAL	A	190	7.677	4.998	23.963	1.00	0.00	C
ATOM	120	CG1	VAL	A	190	6.783	6.053	23.448	1.00	0.00	C
ATOM	121	CG2	VAL	A	190	6.868	3.954	24.743	1.00	0.00	C
ATOM	122	C	VAL	A	190	9.350	5.224	22.072	1.00	0.00	C
ATOM	123	O	VAL	A	190	8.895	5.449	20.930	1.00	0.00	O
ATOM	124	H	VAL	A	190	9.434	3.376	24.603	1.00	0.00	H
ATOM	125	HA	VAL	A	190	7.822	3.722	22.319	1.00	0.00	H
ATOM	126	HB	VAL	A	190	8.424	5.458	24.638	1.00	0.00	H
ATOM	127	HG11	VAL	A	190	6.107	5.573	22.706	1.00	0.00	H
ATOM	128	HG12	VAL	A	190	7.407	6.805	22.924	1.00	0.00	H
ATOM	129	HG13	VAL	A	190	6.349	6.569	24.336	1.00	0.00	H
ATOM	130	HG21	VAL	A	190	7.406	3.000	24.895	1.00	0.00	H
ATOM	131	HG22	VAL	A	190	5.910	3.697	24.248	1.00	0.00	H
ATOM	132	HG23	VAL	A	190	6.680	4.318	25.778	1.00	0.00	H
ATOM	133	N	VAL	A	191	10.561	5.755	22.528	1.00	0.00	N
ATOM	134	CA	VAL	A	191	11.508	6.333	21.487	1.00	0.00	C
ATOM	135	C	VAL	A	191	11.862	5.449	20.311	1.00	0.00	C
ATOM	136	O	VAL	A	191	11.709	5.818	19.140	1.00	0.00	O
ATOM	137	H	VAL	A	191	10.860	5.757	23.482	1.00	0.00	H
ATOM	138	HA2	VAL	A	191	11.042	7.185	21.007	1.00	0.00	H
ATOM	139	HA3	VAL	A	191	12.405	6.528	22.074	1.00	0.00	H
ATOM	140	N	TYR	A	192	12.317	4.237	20.552	1.00	0.00	N
ATOM	141	CA	TYR	A	192	12.990	3.409	19.563	1.00	0.00	C

ATOM	142	C	TYR	A	192	11.916	2.989	18.595	1.00	0.00	C
ATOM	143	O	TYR	A	192	12.138	2.881	17.380	1.00	0.00	O
ATOM	144	H	TYR	A	192	12.474	3.988	21.513	1.00	0.00	H
ATOM	145	HA2	TYR	A	192	13.662	3.984	18.944	1.00	0.00	H
ATOM	146	HA3	TYR	A	192	13.525	2.614	20.082	1.00	0.00	H
ATOM	147	N	SER	A	193	10.726	2.690	19.187	1.00	0.00	N
ATOM	148	CA	SER	A	193	9.502	2.380	18.393	1.00	0.00	C
ATOM	149	CB	SER	A	193	8.268	1.880	19.126	1.00	0.00	C
ATOM	150	OG	SER	A	193	7.144	1.563	18.294	1.00	0.00	O
ATOM	151	C	SER	A	193	9.082	3.485	17.521	1.00	0.00	C
ATOM	152	O	SER	A	193	8.824	3.307	16.342	1.00	0.00	O
ATOM	153	H	SER	A	193	10.604	2.508	20.165	1.00	0.00	H
ATOM	154	HA	SER	A	193	9.843	1.685	17.653	1.00	0.00	H
ATOM	155	HB2	SER	A	193	8.422	1.072	19.869	1.00	0.00	H
ATOM	156	HB3	SER	A	193	7.990	2.725	19.793	1.00	0.00	H
ATOM	157	HG	SER	A	193	7.389	0.692	17.975	1.00	0.00	H
ATOM	158	N	SER	A	194	9.060	4.732	17.997	1.00	0.00	N
ATOM	159	CA	SER	A	194	8.740	5.873	17.231	1.00	0.00	C
ATOM	160	CB	SER	A	194	8.796	7.300	18.024	1.00	0.00	C
ATOM	161	OG	SER	A	194	7.883	7.296	19.180	1.00	0.00	O
ATOM	162	C	SER	A	194	9.626	6.077	15.994	1.00	0.00	C
ATOM	163	O	SER	A	194	9.230	6.327	14.889	1.00	0.00	O
ATOM	164	H	SER	A	194	9.131	4.943	18.977	1.00	0.00	H
ATOM	165	HA	SER	A	194	7.763	5.689	16.815	1.00	0.00	H
ATOM	166	HB2	SER	A	194	9.823	7.412	18.441	1.00	0.00	H
ATOM	167	HB3	SER	A	194	8.502	8.143	17.365	1.00	0.00	H
ATOM	168	HG	SER	A	194	8.189	6.629	19.801	1.00	0.00	H
ATOM	169	N	ILE	A	195	10.950	5.763	16.227	1.00	0.00	N
ATOM	170	CA	ILE	A	195	11.932	5.832	15.234	1.00	0.00	C

ATOM	171	C	ILE	A	195	11.695	4.882	14.103	1.00	0.00	C
ATOM	172	O	ILE	A	195	11.701	5.278	12.929	1.00	0.00	O
ATOM	173	H	ILE	A	195	11.187	5.403	17.124	1.00	0.00	H
ATOM	174	HA2	ILE	A	195	11.877	6.774	14.714	1.00	0.00	H
ATOM	175	HA3	ILE	A	195	12.860	5.639	15.772	1.00	0.00	H
ATOM	176	N	VAL	A	196	11.389	3.670	14.428	1.00	0.00	N
ATOM	177	CA	VAL	A	196	11.141	2.695	13.331	1.00	0.00	C
ATOM	178	C	VAL	A	196	9.747	2.756	12.762	1.00	0.00	C
ATOM	179	O	VAL	A	196	9.598	2.500	11.546	1.00	0.00	O
ATOM	180	H	VAL	A	196	11.365	3.371	15.374	1.00	0.00	H
ATOM	181	HA2	VAL	A	196	11.821	2.853	12.504	1.00	0.00	H
ATOM	182	HA3	VAL	A	196	11.291	1.722	13.799	1.00	0.00	H
ATOM	183	N	SER	A	197	8.729	2.994	13.708	1.00	0.00	N
ATOM	184	CA	SER	A	197	7.304	2.973	13.262	1.00	0.00	C
ATOM	185	CB	SER	A	197	6.238	2.657	14.414	1.00	0.00	C
ATOM	186	OG	SER	A	197	6.334	3.523	15.577	1.00	0.00	O
ATOM	187	C	SER	A	197	6.833	4.183	12.606	1.00	0.00	C
ATOM	188	O	SER	A	197	5.850	4.073	11.876	1.00	0.00	O
ATOM	189	H	SER	A	197	8.950	3.108	14.664	1.00	0.00	H
ATOM	190	HA	SER	A	197	7.313	2.164	12.537	1.00	0.00	H
ATOM	191	HB2	SER	A	197	5.254	2.714	13.920	1.00	0.00	H
ATOM	192	HB3	SER	A	197	6.353	1.604	14.749	1.00	0.00	H
ATOM	193	HG	SER	A	197	7.136	3.283	16.056	1.00	0.00	H
ATOM	194	N	PHE	A	198	7.482	5.349	12.868	1.00	0.00	N
ATOM	195	CA	PHE	A	198	7.172	6.621	12.235	1.00	0.00	C
ATOM	196	H	PHE	A	198	8.276	5.332	13.475	1.00	0.00	H
ATOM	197	HA1	PHE	A	198	6.458	6.370	11.467	1.00	0.00	H
ATOM	198	HA2	PHE	A	198	8.068	7.064	11.799	1.00	0.00	H
ATOM	199	HA3	PHE	A	198	6.737	7.314	12.955	1.00	0.00	H

ATOM	200	CA	CYS	A	385	-2.763	12.564	11.814	1.00	0.00	C
ATOM	201	C	CYS	A	385	-1.699	11.622	12.353	1.00	0.00	C
ATOM	202	O	CYS	A	385	-1.986	10.650	13.046	1.00	0.00	O
ATOM	203	HA1	CYS	A	385	-3.310	12.867	12.693	1.00	0.00	H
ATOM	204	HA2	CYS	A	385	-3.416	12.049	11.109	1.00	0.00	H
ATOM	205	HA3	CYS	A	385	-2.307	13.425	11.325	1.00	0.00	H
ATOM	206	N	TRP	A	386	-0.412	12.017	12.122	1.00	0.00	N
ATOM	207	CA	TRP	A	386	0.633	11.274	12.833	1.00	0.00	C
ATOM	208	CB	TRP	A	386	1.301	10.155	11.929	1.00	0.00	C
ATOM	209	CG	TRP	A	386	0.393	9.005	11.551	1.00	0.00	C
ATOM	210	CD1	TRP	A	386	-0.045	8.744	10.282	1.00	0.00	C
ATOM	211	NE1	TRP	A	386	-1.007	7.745	10.417	1.00	0.00	N
ATOM	212	CE2	TRP	A	386	-1.076	7.244	11.697	1.00	0.00	C
ATOM	213	CD2	TRP	A	386	-0.116	8.001	12.419	1.00	0.00	C
ATOM	214	CE3	TRP	A	386	-0.075	7.719	13.809	1.00	0.00	C
ATOM	215	CZ3	TRP	A	386	-0.841	6.667	14.348	1.00	0.00	C
ATOM	216	CZ2	TRP	A	386	-1.883	6.224	12.241	1.00	0.00	C
ATOM	217	CH2	TRP	A	386	-1.681	5.911	13.576	1.00	0.00	C
ATOM	218	C	TRP	A	386	1.696	12.158	13.405	1.00	0.00	C
ATOM	219	O	TRP	A	386	2.190	11.793	14.488	1.00	0.00	O
ATOM	220	H	TRP	A	386	-0.258	12.818	11.555	1.00	0.00	H
ATOM	221	HA	TRP	A	386	0.248	10.821	13.739	1.00	0.00	H
ATOM	222	HB2	TRP	A	386	1.617	10.620	10.960	1.00	0.00	H
ATOM	223	HB3	TRP	A	386	2.222	9.742	12.406	1.00	0.00	H
ATOM	224	HD1	TRP	A	386	0.240	9.418	9.483	1.00	0.00	H
ATOM	225	HE1	TRP	A	386	-1.410	7.518	9.567	1.00	0.00	H
ATOM	226	HE3	TRP	A	386	0.496	8.409	14.396	1.00	0.00	H
ATOM	227	HZ2	TRP	A	386	-2.533	5.594	11.644	1.00	0.00	H
ATOM	228	HZ3	TRP	A	386	-0.855	6.486	15.408	1.00	0.00	H

ATOM	229	HH2	TRP	A	386	-2.204	5.079	14.043	1.00	0.00	H
ATOM	230	N	LEU	A	387	2.074	13.275	12.802	1.00	0.00	N
ATOM	231	CA	LEU	A	387	3.178	14.114	13.210	1.00	0.00	C
ATOM	232	C	LEU	A	387	3.061	14.886	14.537	1.00	0.00	C
ATOM	233	O	LEU	A	387	3.931	14.757	15.344	1.00	0.00	O
ATOM	234	H	LEU	A	387	1.626	13.630	11.984	1.00	0.00	H
ATOM	235	HA2	LEU	A	387	3.948	13.372	13.381	1.00	0.00	H
ATOM	236	HA3	LEU	A	387	3.309	14.893	12.459	1.00	0.00	H
ATOM	237	N	PRO	A	388	1.954	15.550	14.889	1.00	0.00	N
ATOM	238	CA	PRO	A	388	1.915	16.152	16.233	1.00	0.00	C
ATOM	239	C	PRO	A	388	1.873	15.108	17.334	1.00	0.00	C
ATOM	240	O	PRO	A	388	2.543	15.279	18.367	1.00	0.00	O
ATOM	241	H	PRO	A	388	1.172	15.636	14.255	1.00	0.00	H
ATOM	242	HA2	PRO	A	388	2.830	16.721	16.387	1.00	0.00	H
ATOM	243	HA3	PRO	A	388	1.016	16.766	16.282	1.00	0.00	H
ATOM	244	N	PHE	A	389	1.234	14.001	17.064	1.00	0.00	N
ATOM	245	CA	PHE	A	389	1.243	12.836	17.911	1.00	0.00	C
ATOM	246	CB	PHE	A	389	0.390	11.785	17.194	1.00	0.00	C
ATOM	247	CG	PHE	A	389	-0.012	10.595	18.041	1.00	0.00	C
ATOM	248	CD1	PHE	A	389	-0.674	9.598	17.298	1.00	0.00	C
ATOM	249	CE1	PHE	A	389	-1.401	8.592	17.942	1.00	0.00	C
ATOM	250	CZ	PHE	A	389	-1.278	8.450	19.331	1.00	0.00	C
ATOM	251	CD2	PHE	A	389	0.002	10.485	19.434	1.00	0.00	C
ATOM	252	CE2	PHE	A	389	-0.517	9.331	20.052	1.00	0.00	C
ATOM	253	C	PHE	A	389	2.613	12.145	18.192	1.00	0.00	C
ATOM	254	O	PHE	A	389	2.906	11.726	19.372	1.00	0.00	O
ATOM	255	H	PHE	A	389	0.722	13.866	16.209	1.00	0.00	H
ATOM	256	HA	PHE	A	389	0.726	13.123	18.813	1.00	0.00	H
ATOM	257	HB2	PHE	A	389	-0.572	12.168	16.782	1.00	0.00	H

ATOM	258	HB3	PHE	A	389	0.985	11.375	16.352	1.00	0.00	H
ATOM	259	HD1	PHE	A	389	-0.729	9.917	16.277	1.00	0.00	H
ATOM	260	HD2	PHE	A	389	0.575	11.270	19.916	1.00	0.00	H
ATOM	261	HE1	PHE	A	389	-1.813	7.779	17.355	1.00	0.00	H
ATOM	262	HE2	PHE	A	389	-0.260	9.186	21.099	1.00	0.00	H
ATOM	263	HZ	PHE	A	389	-1.861	7.653	19.762	1.00	0.00	H
ATOM	264	N	PHE	A	390	3.473	11.965	17.155	1.00	0.00	N
ATOM	265	CA	PHE	A	390	4.784	11.315	17.308	1.00	0.00	C
ATOM	266	CB	PHE	A	390	5.210	10.502	16.000	1.00	0.00	C
ATOM	267	CG	PHE	A	390	4.597	9.133	15.932	1.00	0.00	C
ATOM	268	CD1	PHE	A	390	3.767	8.869	14.811	1.00	0.00	C
ATOM	269	CE1	PHE	A	390	3.176	7.603	14.626	1.00	0.00	C
ATOM	270	CZ	PHE	A	390	3.535	6.557	15.549	1.00	0.00	C
ATOM	271	CD2	PHE	A	390	4.877	8.121	16.809	1.00	0.00	C
ATOM	272	CE2	PHE	A	390	4.446	6.812	16.621	1.00	0.00	C
ATOM	273	C	PHE	A	390	5.861	12.312	17.691	1.00	0.00	C
ATOM	274	O	PHE	A	390	6.861	12.010	18.303	1.00	0.00	O
ATOM	275	H	PHE	A	390	3.225	12.196	16.207	1.00	0.00	H
ATOM	276	HA	PHE	A	390	4.788	10.691	18.183	1.00	0.00	H
ATOM	277	HB2	PHE	A	390	4.946	11.072	15.080	1.00	0.00	H
ATOM	278	HB3	PHE	A	390	6.329	10.462	16.088	1.00	0.00	H
ATOM	279	HD1	PHE	A	390	3.603	9.722	14.162	1.00	0.00	H
ATOM	280	HD2	PHE	A	390	5.610	8.273	17.591	1.00	0.00	H
ATOM	281	HE1	PHE	A	390	2.442	7.403	13.861	1.00	0.00	H
ATOM	282	HE2	PHE	A	390	4.710	6.000	17.285	1.00	0.00	H
ATOM	283	HZ	PHE	A	390	3.176	5.541	15.429	1.00	0.00	H
ATOM	284	N	ILE	A	391	5.606	13.592	17.441	1.00	0.00	N
ATOM	285	CA	ILE	A	391	6.260	14.680	18.119	1.00	0.00	C
ATOM	286	C	ILE	A	391	6.013	14.708	19.661	1.00	0.00	C

ATOM	287	O	ILE	A	391	6.966	14.801	20.475	1.00	0.00	O
ATOM	288	H	ILE	A	391	4.876	13.826	16.801	1.00	0.00	H
ATOM	289	HA2	ILE	A	391	7.339	14.513	18.105	1.00	0.00	H
ATOM	290	HA3	ILE	A	391	5.884	15.571	17.616	1.00	0.00	H
ATOM	291	N	THR	A	392	4.778	14.546	19.958	1.00	0.00	N
ATOM	292	CA	THR	A	392	4.323	14.472	21.290	1.00	0.00	C
ATOM	293	C	THR	A	392	4.852	13.263	22.088	1.00	0.00	C
ATOM	294	O	THR	A	392	5.221	13.446	23.237	1.00	0.00	O
ATOM	295	H	THR	A	392	4.135	14.417	19.202	1.00	0.00	H
ATOM	296	HA2	THR	A	392	4.867	15.325	21.680	1.00	0.00	H
ATOM	297	HA3	THR	A	392	3.236	14.421	21.353	1.00	0.00	H
ATOM	298	N	HSD	A	393	5.098	12.138	21.434	1.00	0.00	N
ATOM	299	CA	HSD	A	393	5.930	11.107	22.096	1.00	0.00	C
ATOM	300	CB	HSD	A	393	6.097	9.832	21.191	1.00	0.00	C
ATOM	301	ND1	HSD	A	393	3.629	9.490	20.613	1.00	0.00	N
ATOM	302	CG	HSD	A	393	4.881	8.961	20.889	1.00	0.00	C
ATOM	303	CE1	HSD	A	393	2.850	8.417	20.488	1.00	0.00	C
ATOM	304	NE2	HSD	A	393	3.517	7.276	20.619	1.00	0.00	N
ATOM	305	CD2	HSD	A	393	4.758	7.689	20.901	1.00	0.00	C
ATOM	306	C	HSD	A	393	7.330	11.561	22.525	1.00	0.00	C
ATOM	307	O	HSD	A	393	7.736	11.346	23.594	1.00	0.00	O
ATOM	308	H	HSD	A	393	4.687	12.086	20.537	1.00	0.00	H
ATOM	309	HA	HSD	A	393	5.397	10.793	22.979	1.00	0.00	H
ATOM	310	HB2	HSD	A	393	6.700	10.146	20.319	1.00	0.00	H
ATOM	311	HB3	HSD	A	393	6.764	9.167	21.785	1.00	0.00	H
ATOM	312	HD1	HSD	A	393	3.322	10.402	20.338	1.00	0.00	H
ATOM	313	HD2	HSD	A	393	5.497	6.930	21.156	1.00	0.00	H
ATOM	314	HE1	HSD	A	393	1.813	8.388	20.149	1.00	0.00	H
ATOM	315	N	ILE	A	394	8.096	12.052	21.566	1.00	0.00	N

ATOM	316	CA	ILE	A	394	9.498	12.413	21.656	1.00	0.00	C
ATOM	317	H	ILE	A	394	7.876	12.005	20.590	1.00	0.00	H
ATOM	318	HA1	ILE	A	394	10.146	11.648	22.063	1.00	0.00	H
ATOM	319	HA2	ILE	A	394	9.579	13.296	22.290	1.00	0.00	H
ATOM	320	HA3	ILE	A	394	9.849	12.653	20.653	1.00	0.00	H
ATOM	321	CA	LEU	A	407	-4.246	14.746	24.972	1.00	0.00	C
ATOM	322	C	LEU	A	407	-4.020	13.455	24.161	1.00	0.00	C
ATOM	323	O	LEU	A	407	-4.399	13.257	22.973	1.00	0.00	O
ATOM	324	HA1	LEU	A	407	-4.903	15.334	24.339	1.00	0.00	H
ATOM	325	HA2	LEU	A	407	-3.304	15.267	25.145	1.00	0.00	H
ATOM	326	HA3	LEU	A	407	-4.721	14.527	25.928	1.00	0.00	H
ATOM	327	N	TYR	A	408	-3.578	12.321	24.784	1.00	0.00	N
ATOM	328	CA	TYR	A	408	-3.413	11.069	24.088	1.00	0.00	C
ATOM	329	CB	TYR	A	408	-2.804	9.954	24.913	1.00	0.00	C
ATOM	330	CG	TYR	A	408	-1.302	10.230	25.047	1.00	0.00	C
ATOM	331	CD1	TYR	A	408	-0.512	9.399	24.241	1.00	0.00	C
ATOM	332	CE1	TYR	A	408	0.809	9.513	24.297	1.00	0.00	C
ATOM	333	CZ	TYR	A	408	1.440	10.502	25.122	1.00	0.00	C
ATOM	334	OH	TYR	A	408	2.869	10.544	25.240	1.00	0.00	O
ATOM	335	CD2	TYR	A	408	-0.742	11.203	25.955	1.00	0.00	C
ATOM	336	CE2	TYR	A	408	0.709	11.290	25.975	1.00	0.00	C
ATOM	337	C	TYR	A	408	-4.753	10.498	23.496	1.00	0.00	C
ATOM	338	O	TYR	A	408	-4.636	9.962	22.385	1.00	0.00	O
ATOM	339	H	TYR	A	408	-3.228	12.380	25.720	1.00	0.00	H
ATOM	340	HA	TYR	A	408	-2.891	11.276	23.171	1.00	0.00	H
ATOM	341	HB2	TYR	A	408	-3.225	9.955	25.941	1.00	0.00	H
ATOM	342	HB3	TYR	A	408	-2.889	8.968	24.416	1.00	0.00	H
ATOM	343	HD1	TYR	A	408	-0.971	8.710	23.543	1.00	0.00	H
ATOM	344	HD2	TYR	A	408	-1.331	11.920	26.496	1.00	0.00	H

ATOM	345	HE1	TYR	A	408	1.479	8.907	23.698	1.00	0.00	H
ATOM	346	HE2	TYR	A	408	1.160	12.065	26.578	1.00	0.00	H
ATOM	347	HH	TYR	A	408	3.213	9.677	25.022	1.00	0.00	H
ATOM	348	N	SER	A	409	-5.831	10.568	24.264	1.00	0.00	N
ATOM	349	CA	SER	A	409	-7.140	10.115	23.714	1.00	0.00	C
ATOM	350	H	SER	A	409	-5.829	10.837	25.224	1.00	0.00	H
ATOM	351	HA1	SER	A	409	-7.048	9.043	23.526	1.00	0.00	H
ATOM	352	HA2	SER	A	409	-7.360	10.642	22.785	1.00	0.00	H
ATOM	353	HA3	SER	A	409	-7.936	10.303	24.435	1.00	0.00	H
ATOM	354	CA	PHE	A	411	-4.676	12.065	18.884	1.00	0.00	C
ATOM	355	C	PHE	A	411	-4.987	10.642	18.493	1.00	0.00	C
ATOM	356	O	PHE	A	411	-4.947	10.348	17.304	1.00	0.00	O
ATOM	357	HA1	PHE	A	411	-4.811	12.601	17.951	1.00	0.00	H
ATOM	358	HA2	PHE	A	411	-3.653	12.159	19.247	1.00	0.00	H
ATOM	359	HA3	PHE	A	411	-5.367	12.423	19.647	1.00	0.00	H
ATOM	360	N	THR	A	412	-5.266	9.746	19.418	1.00	0.00	N
ATOM	361	CA	THR	A	412	-5.657	8.367	19.171	1.00	0.00	C
ATOM	362	CB	THR	A	412	-5.870	7.562	20.436	1.00	0.00	C
ATOM	363	OG1	THR	A	412	-4.618	7.466	21.090	1.00	0.00	O
ATOM	364	CG2	THR	A	412	-6.452	6.122	20.237	1.00	0.00	C
ATOM	365	C	THR	A	412	-6.820	8.205	18.294	1.00	0.00	C
ATOM	366	O	THR	A	412	-6.825	7.530	17.255	1.00	0.00	O
ATOM	367	H	THR	A	412	-5.425	9.947	20.387	1.00	0.00	H
ATOM	368	HA	THR	A	412	-4.795	7.979	18.621	1.00	0.00	H
ATOM	369	HB	THR	A	412	-6.531	8.131	21.121	1.00	0.00	H
ATOM	370	HG21	THR	A	412	-7.457	6.184	19.773	1.00	0.00	H
ATOM	371	HG22	THR	A	412	-6.458	5.629	21.244	1.00	0.00	H
ATOM	372	HG23	THR	A	412	-5.909	5.567	19.437	1.00	0.00	H
ATOM	373	HG1	THR	A	412	-4.511	8.391	21.323	1.00	0.00	H

ATOM	374	N	TRP	A	413	-7.938	8.851	18.721	1.00	0.00	N
ATOM	375	CA	TRP	A	413	-9.137	9.000	17.887	1.00	0.00	C
ATOM	376	C	TRP	A	413	-8.896	9.684	16.560	1.00	0.00	C
ATOM	377	O	TRP	A	413	-9.450	9.238	15.591	1.00	0.00	O
ATOM	378	H	TRP	A	413	-7.951	9.404	19.556	1.00	0.00	H
ATOM	379	HA2	TRP	A	413	-9.414	8.008	17.602	1.00	0.00	H
ATOM	380	HA3	TRP	A	413	-9.857	9.580	18.465	1.00	0.00	H
ATOM	381	N	LEU	A	414	-7.995	10.661	16.469	1.00	0.00	N
ATOM	382	CA	LEU	A	414	-7.604	11.175	15.108	1.00	0.00	C
ATOM	383	C	LEU	A	414	-6.806	10.133	14.315	1.00	0.00	C
ATOM	384	O	LEU	A	414	-6.852	10.154	13.067	1.00	0.00	O
ATOM	385	H	LEU	A	414	-7.491	10.923	17.293	1.00	0.00	H
ATOM	386	HA2	LEU	A	414	-8.454	11.592	14.591	1.00	0.00	H
ATOM	387	HA3	LEU	A	414	-6.912	12.008	15.230	1.00	0.00	H
ATOM	388	N	GLY	A	415	-6.143	9.136	14.920	1.00	0.00	N
ATOM	389	CA	GLY	A	415	-5.536	8.037	14.204	1.00	0.00	C
ATOM	390	C	GLY	A	415	-6.580	7.071	13.635	1.00	0.00	C
ATOM	391	O	GLY	A	415	-6.364	6.516	12.538	1.00	0.00	O
ATOM	392	H	GLY	A	415	-6.135	9.005	15.911	1.00	0.00	H
ATOM	393	HA2	GLY	A	415	-4.849	7.499	14.840	1.00	0.00	H
ATOM	394	HA3	GLY	A	415	-5.042	8.420	13.310	1.00	0.00	H
ATOM	395	N	TYR	A	416	-7.768	6.993	14.283	1.00	0.00	N
ATOM	396	CA	TYR	A	416	-8.843	6.078	13.908	1.00	0.00	C
ATOM	397	CB	TYR	A	416	-9.553	5.492	15.175	1.00	0.00	C
ATOM	398	CG	TYR	A	416	-8.571	4.633	15.984	1.00	0.00	C
ATOM	399	CD1	TYR	A	416	-7.845	3.627	15.417	1.00	0.00	C
ATOM	400	CE1	TYR	A	416	-7.083	2.701	16.164	1.00	0.00	C
ATOM	401	CZ	TYR	A	416	-6.946	2.854	17.568	1.00	0.00	C
ATOM	402	OH	TYR	A	416	-5.941	2.144	18.294	1.00	0.00	O

ATOM	403	CD2	TYR	A	416	-8.616	4.631	17.350	1.00	0.00	C
ATOM	404	CE2	TYR	A	416	-7.844	3.724	18.155	1.00	0.00	C
ATOM	405	C	TYR	A	416	-9.836	6.660	12.878	1.00	0.00	C
ATOM	406	O	TYR	A	416	-10.513	6.088	12.069	1.00	0.00	O
ATOM	407	H	TYR	A	416	-7.873	7.461	15.151	1.00	0.00	H
ATOM	408	HA	TYR	A	416	-8.387	5.239	13.424	1.00	0.00	H
ATOM	409	HB2	TYR	A	416	-9.801	6.396	15.772	1.00	0.00	H
ATOM	410	HB3	TYR	A	416	-10.465	4.958	14.854	1.00	0.00	H
ATOM	411	HD1	TYR	A	416	-7.735	3.472	14.350	1.00	0.00	H
ATOM	412	HD2	TYR	A	416	-9.334	5.307	17.806	1.00	0.00	H
ATOM	413	HE1	TYR	A	416	-6.404	2.001	15.681	1.00	0.00	H
ATOM	414	HE2	TYR	A	416	-7.825	3.809	19.235	1.00	0.00	H
ATOM	415	HH	TYR	A	416	-5.330	1.685	17.723	1.00	0.00	H
ATOM	416	N	VAL	A	417	-9.802	8.065	12.875	1.00	0.00	N
ATOM	417	CA	VAL	A	417	-10.302	8.902	11.831	1.00	0.00	C
ATOM	418	H	VAL	A	417	-9.355	8.540	13.625	1.00	0.00	H
ATOM	419	HA1	VAL	A	417	-11.302	8.610	11.563	1.00	0.00	H
ATOM	420	HA2	VAL	A	417	-9.655	8.812	10.958	1.00	0.00	H
ATOM	421	HA3	VAL	A	417	-10.309	9.937	12.173	1.00	0.00	H
TER	422		VAL	A	417						
HETATM	423	C1	ROT	B	444	-1.867	4.456	21.012	1.00	0.00	C
HETATM	424	N	ROT	B	444	-1.702	3.618	19.716	1.00	0.00	N1+
HETATM	425	C2	ROT	B	444	-2.859	3.847	18.749	1.00	0.00	C
HETATM	426	C3	ROT	B	444	-2.767	5.239	18.070	1.00	0.00	C
HETATM	427	C4	ROT	B	444	-3.672	5.488	16.921	1.00	0.00	C
HETATM	428	C5	ROT	B	444	-0.266	3.471	19.074	1.00	0.00	C
HETATM	429	C6	ROT	B	444	-0.027	2.268	18.108	1.00	0.00	C
HETATM	430	C7	ROT	B	444	0.792	3.452	20.150	1.00	0.00	C
HETATM	431	C8	ROT	B	444	1.293	2.502	17.366	1.00	0.00	C

HETATM	432	C9	ROT	B	444	2.160	3.434	19.477	1.00	0.00	C
HETATM	433	C10	ROT	B	444	2.358	2.872	18.210	1.00	0.00	C
HETATM	434	C11	ROT	B	444	3.208	4.017	20.197	1.00	0.00	C
HETATM	435	C12	ROT	B	444	3.674	2.892	17.652	1.00	0.00	C
HETATM	436	C13	ROT	B	444	4.524	4.001	19.665	1.00	0.00	C
HETATM	437	C14	ROT	B	444	4.742	3.500	18.399	1.00	0.00	C
HETATM	438	O	ROT	B	444	3.884	2.358	16.300	1.00	0.00	O
HETATM	439	C15	ROT	B	444	-1.799	3.670	22.287	1.00	0.00	C
HETATM	440	C16	ROT	B	444	-1.813	4.577	23.514	1.00	0.00	C
HETATM	441	C17	ROT	B	444	-2.838	4.987	24.321	1.00	0.00	C
HETATM	442	S	ROT	B	444	-0.313	5.257	24.101	1.00	0.00	S
HETATM	443	C18	ROT	B	444	-2.479	5.910	25.285	1.00	0.00	C
HETATM	444	C19	ROT	B	444	-1.117	6.119	25.306	1.00	0.00	C
HETATM	445	H1	ROT	B	444	-2.862	4.929	20.981	1.00	0.00	H
HETATM	446	H2	ROT	B	444	-1.059	5.207	20.955	1.00	0.00	H
HETATM	447	H3	ROT	B	444	-1.965	2.667	20.032	1.00	0.00	H
HETATM	448	H4	ROT	B	444	-3.767	3.727	19.346	1.00	0.00	H
HETATM	449	H5	ROT	B	444	-2.872	2.916	18.154	1.00	0.00	H
HETATM	450	H6	ROT	B	444	-1.702	5.502	17.888	1.00	0.00	H
HETATM	451	H7	ROT	B	444	-3.118	5.976	18.824	1.00	0.00	H
HETATM	452	H8	ROT	B	444	-4.727	5.391	17.260	1.00	0.00	H
HETATM	453	H9	ROT	B	444	-3.532	4.682	16.166	1.00	0.00	H
HETATM	454	H10	ROT	B	444	-3.616	6.496	16.442	1.00	0.00	H
HETATM	455	H11	ROT	B	444	-0.040	4.404	18.508	1.00	0.00	H
HETATM	456	H12	ROT	B	444	-0.788	2.315	17.305	1.00	0.00	H
HETATM	457	H13	ROT	B	444	0.016	1.324	18.689	1.00	0.00	H
HETATM	458	H14	ROT	B	444	0.676	2.561	20.792	1.00	0.00	H
HETATM	459	H15	ROT	B	444	0.773	4.292	20.870	1.00	0.00	H
HETATM	460	H16	ROT	B	444	1.139	3.427	16.787	1.00	0.00	H

HETATM	461	H17	ROT	B 444	1.532	1.657	16.689	1.00	0.00	H
HETATM	462	H18	ROT	B 444	3.133	4.557	21.135	1.00	0.00	H
HETATM	463	H19	ROT	B 444	5.282	4.471	20.278	1.00	0.00	H
HETATM	464	H20	ROT	B 444	5.767	3.548	18.067	1.00	0.00	H
HETATM	465	H21	ROT	B 444	4.789	2.619	16.096	1.00	0.00	H
HETATM	466	H22	ROT	B 444	-0.907	3.018	22.396	1.00	0.00	H
HETATM	467	H23	ROT	B 444	-2.716	3.032	22.276	1.00	0.00	H
HETATM	468	H24	ROT	B 444	-3.842	4.573	24.315	1.00	0.00	H
HETATM	469	H25	ROT	B 444	-3.162	6.406	25.961	1.00	0.00	H
HETATM	470	H26	ROT	B 444	-0.489	6.690	25.973	1.00	0.00	H
HETATM	471	OH2	TIP	X1573	-4.559	1.668	21.294	1.00	0.00	O
HETATM	472	H1	TIP	X1573	-4.978	1.803	20.430	1.00	0.00	H
HETATM	473	H2	TIP	X1573	-3.680	1.343	21.090	1.00	0.00	H
HETATM	474	OH2	TIP	X3380	-2.752	5.927	30.117	1.00	0.00	O
HETATM	475	H1	TIP	X3380	-2.806	5.193	30.735	1.00	0.00	H
HETATM	476	H2	TIP	X3380	-3.287	5.648	29.375	1.00	0.00	H
HETATM	477	OH2	TIP	X4433	0.445	1.408	24.336	1.00	0.00	O
HETATM	478	H1	TIP	X4433	0.524	0.635	23.788	1.00	0.00	H
HETATM	479	H2	TIP	X4433	-0.492	1.629	24.313	1.00	0.00	H
HETATM	480	OH2	TIP	X5714	-2.345	0.458	23.464	1.00	0.00	O
HETATM	481	H1	TIP	X5714	-2.012	0.936	22.716	1.00	0.00	H
HETATM	482	H2	TIP	X5714	-2.650	-0.362	23.112	1.00	0.00	H
HETATM	483	OH2	TIP	X7169	0.510	9.128	28.350	1.00	0.00	O
HETATM	484	H1	TIP	X7169	0.743	9.936	28.824	1.00	0.00	H
HETATM	485	H2	TIP	X7169	-0.406	9.249	28.080	1.00	0.00	H
HETATM	486	OH2	TIP	X 898	1.566	6.650	27.324	1.00	0.00	O
HETATM	487	H1	TIP	X 898	1.424	7.586	27.472	1.00	0.00	H
HETATM	488	H2	TIP	X 898	1.493	6.259	28.193	1.00	0.00	H
HETATM	489	OH2	TIP	X2732	-5.364	5.971	28.487	1.00	0.00	O

HETATM	490	H1	TIP	X2732	-5.973	6.425	27.905	1.00	0.00	H
HETATM	491	H2	TIP	X2732	-5.097	5.195	28.001	1.00	0.00	H
HETATM	492	OH2	TIP	X3408	-6.858	4.968	24.215	1.00	0.00	O
HETATM	493	H1	TIP	X3408	-6.574	4.088	23.979	1.00	0.00	H
HETATM	494	H2	TIP	X3408	-7.069	4.901	25.145	1.00	0.00	H
HETATM	495	OH2	TIP	X9269	-4.127	0.956	16.764	1.00	0.00	O
HETATM	496	H1	TIP	X9269	-3.373	0.948	17.359	1.00	0.00	H
HETATM	497	H2	TIP	X9269	-3.765	1.207	15.919	1.00	0.00	H
HETATM	498	OH2	TIP	X7090	2.928	5.598	25.323	1.00	0.00	O
HETATM	499	H1	TIP	X7090	2.335	4.924	24.976	1.00	0.00	H
HETATM	500	H2	TIP	X7090	2.453	5.980	26.060	1.00	0.00	H
HETATM	501	OH2	TIP	X8123	-3.187	1.932	14.032	1.00	0.00	O
HETATM	502	H1	TIP	X8123	-3.620	2.763	14.204	1.00	0.00	H
HETATM	503	H2	TIP	X8123	-3.429	1.697	13.145	1.00	0.00	H
HETATM	504	OH2	TIP	X2167	-5.355	7.171	24.184	1.00	0.00	O
HETATM	505	H1	TIP	X2167	-6.072	6.544	24.264	1.00	0.00	H
HETATM	506	H2	TIP	X2167	-4.810	6.845	23.490	1.00	0.00	H
HETATM	507	OH2	TIP	X2817	-2.093	8.448	28.926	1.00	0.00	O
HETATM	508	H1	TIP	X2817	-2.200	7.548	28.658	1.00	0.00	H
HETATM	509	H2	TIP	X2817	-2.054	8.432	29.879	1.00	0.00	H
HETATM	510	OH2	TIP	X3430	-5.681	8.449	26.878	1.00	0.00	O
HETATM	511	H1	TIP	X3430	-5.413	9.366	27.003	1.00	0.00	H
HETATM	512	H2	TIP	X3430	-5.245	8.169	26.075	1.00	0.00	H
HETATM	513	OH2	TIP	X5522	-3.900	1.342	25.671	1.00	0.00	O
HETATM	514	H1	TIP	X5522	-3.392	1.046	26.420	1.00	0.00	H
HETATM	515	H2	TIP	X5522	-3.537	0.869	24.928	1.00	0.00	H
HETATM	516	OH2	TIP	X6133	-5.386	2.593	23.694	1.00	0.00	O
HETATM	517	H1	TIP	X6133	-5.231	2.188	22.847	1.00	0.00	H
HETATM	518	H2	TIP	X6133	-4.767	2.152	24.294	1.00	0.00	H

```
HETATM  519  OH2 TIP X6374      -4.146   4.128  26.930  1.00  0.00           O
HETATM  520  H1  TIP X6374      -3.783   4.653  26.225  1.00  0.00           H
HETATM  521  H2  TIP X6374      -4.052   3.220  26.625  1.00  0.00           H
HETATM  522  OH2 TIP X1906       1.440   6.297  18.433  1.00  0.00           O
HETATM  523  H1  TIP X1906       1.683   6.595  17.560  1.00  0.00           H
HETATM  524  H2  TIP X1906       2.246   6.376  18.944  1.00  0.00           H
HETATM  525  OH2 TIP X5890      -4.746   4.279  13.383  1.00  0.00           O
HETATM  526  H1  TIP X5890      -4.447   4.144  12.466  1.00  0.00           H
HETATM  527  H2  TIP X5890      -5.610   3.898  13.404  1.00  0.00           H
HETATM  528  OH2 TIP X1543       0.659   0.164  21.919  1.00  0.00           O
HETATM  529  H1  TIP X1543       1.168  -0.484  21.426  1.00  0.00           H
HETATM  530  H2  TIP X1543      -0.124   0.284  21.387  1.00  0.00           H
END
```
